# From a shared origin to divergent frames: A computational analysis of media representations of Kampo Medicine and Traditional Chinese Medicine in *Asahi Shimbun*

**DOI:** 10.1371/journal.pone.0351860

**Published:** 2026-06-30

**Authors:** Xi Chen, Jieqiong Wang, Xinying Liu

**Affiliations:** 1 School of Japanese Culture and Economics, Xi’an International Studies University, Xi’an, Shaanxi, China; 2 Graduate School, Xi’an International Studies University, Xi’an, Shaanxi, China; 3 School of Literature and History, Longdong University, Qingyang, Gansu, China; 4 School of Foreign Languages, Shanghai University, Shanghai, China; PLOS ONE, UNITED KINGDOM OF GREAT BRITAIN AND NORTHERN IRELAND

## Abstract

Based on a corpus of news articles published in *Asahi Shimbun* over four decades (1984–2025), this study examines how Kampo medicine and Traditional Chinese Medicine (TCM), two medical traditions sharing a common historical origin, are discursively represented in Japanese mainstream media. Using topic modeling and word embedding analysis, it analyzes long-term patterns at two levels: thematic structures identified through topic modeling and semantic associations derived from word embeddings. The results show that *Asahi Shimbun* consistently differentiates Kampo medicine and TCM through sustained thematic and semantic organization. At the thematic level, coverage is structured across six domains, within which Kampo medicine is more stably integrated into core public concerns related to health practices and material supply, while TCM is more often situated in culturally oriented, comparative, or externally referenced contexts. Diachronically, these distinctions persist while adjusting to changing social demands and policy priorities. At the semantic level, Kampo medicine and TCM form distinct and relatively stable semantic neighborhoods. Kampo medicine is closely associated with concrete therapeutic entities, while TCM is framed within abstract classificatory, institutional, and system-oriented concepts. Together, thematic differentiation and semantic stabilization reinforce a clear discursive boundary between the two traditions. These findings reveal how Japanese mainstream media differentially position homologous medical knowledge over the long term, highlighting their role in shaping meaning within the modern nation-state context. The study applies recontextualization to long-term media analysis and shows how agenda-setting and framing contribute to the localization and othering of homologous knowledge. By linking macro-level thematic structures with micro-level semantic patterns, it further clarifies how media discourse shapes knowledge boundaries and cultural identity, extending the application of recontextualization theory in health and cross-cultural communication research.

## Introduction

The cross-border circulation of traditional knowledge systems is not merely a process of information transfer. Rather, such knowledge is continuously reinterpreted, reorganized, and endowed with new meanings within the public discourse and audience expectations of the receiving society. This dynamic process is commonly conceptualized as recontextualization [1–4]. In contemporary societies, news media function as a primary source of public information and actively participate in the reconstruction and boundary-making of knowledge objects through agenda-setting and framing practices, thereby shaping public understandings of their origins, attributes, and social value [5–9].

Within the East Asian context, the transnational circulation of medical knowledge provides a particularly illustrative case. Historically, the region has been characterized by long-standing patterns of knowledge mobility grounded in the shared use of Sinitic script, within which Traditional Chinese Medicine (TCM) developed as a systematized medical tradition with strong cross-regional transmissibility [10]. From the 5th to 6th centuries onward, Chinese medical texts, prescriptions, and therapeutic principles have entered Japan through both official and non-official channels. Over time, these bodies of knowledge were selectively absorbed and adapted, becoming a major source for the formation of Japan’s traditional medical system [11].

Following the Meiji Restoration (1868), Japan institutionalized a classificatory framework distinguishing Kampo medicine from Rangaku (Dutch studies in Western medicine) [12], gradually consolidating Kampo as a category of indigenous traditional medicine. In parallel, TCM came to be positioned as an externally originating system, leading to the increasingly distinct knowledge boundaries between the two. As a result, TCM and Kampo medicine constitute a paradigmatic case of a “shared-origin yet divergent-development” knowledge system. While this process has been extensively examined in medical history and sociology [10,11,13–17], far less attention has been paid to its representation in contemporary media discourse. In public communication, East Asian traditional medicine is often treated as a homogeneous category, and empirical analyses of internal differentiation remain scarce. Although Japan maintains deep historical ties to TCM while operating within a modern biomedical system, it remains unclear how mainstream media have represented and differentiated Kampo medicine and TCM over time.

Building on this gap, this study draws on a corpus of articles published over four decades in *Asahi Shimbun*, one of Japan’s most influential national newspapers, and applies text-mining approaches to examine media representations of traditional medicine. Latent Dirichlet Allocation (LDA) is employed to identify the major thematic structures and trace their distribution and longitudinal shifts. In parallel, a Word2Vec model is used to examine the semantic neighborhoods of Kampo medicine and TCM, capturing differences in how the two are narratively framed.

By integrating thematic and semantic analyses, this study provides an empirical account of how medical knowledge with a shared historical origin is represented in contemporary media discourse and offers interpretive implications for recontextualization. It addresses the following research questions:

(1) What are the dominant themes through which Japanese mainstream media frame Kampo medicine and TCM, and how have the distribution and relative salience changed over the past four decades?
(2) Within these thematic contexts, what kinds of semantic field structures emerge around Kampo medicine and TCM, and how do they contribute to the construction of interpretive pathways and knowledge boundaries?

The remainder of the paper is organized as follows. The next section reviews the literature and outlines the research design, followed by the presentation of results, discussion, and conclusion.

## Literature review

### From traditional Chinese medicine to Kampo medicine: A historical overview

Although traditional medical systems across East Asia differ in their specific prescriptions and diagnostic practices, they broadly share theoretical foundations derived from TCM [18]. When this body of knowledge was transmitted to Japan, it gradually evolved into Kampo medicine with distinct local characteristics, following a trajectory of shared origin yet divergent development [19].

Existing research indicates that the transmission of TCM to Japan can be traced back to the fifth and sixth centuries, with the Korean Peninsula serving as a major intermediary [20]. During this period, classical texts such as *Shanghan Lun* and *Huangdi Neijing* were introduced into Japan and became central to medical education among aristocracy and official institutions [14]. At this early stage, Chinese medicine and indigenous Japanese practices exhibited a high degree of conceptual and practical homogeneity, and their influence was largely confined to state institutions and Buddhist communities [11].

From the Kamakura period (1185–1333) onward, as Buddhist monks played an important role in the dissemination of medical knowledge, TCM spread more widely across Japanese society [12]. This expansion was accompanied by selective appropriation and adaptation. During the Edo period (1603–1868), within a relatively closed environment, multiple local schools emerged based on traditional Chinese medical theory, alongside systematic reinterpretation and reorganization of medical texts, prescriptions, and theoretical frameworks. This period marked the beginning of sustained reconstruction of traditional medicine within the Japanese context [21]. At the same time, the introduction of European medicine altered Japan’s medical epistemology, and China ceased to serve as the sole external reference for medical knowledge [22].

Following the Meiji Restoration, Japan adopted Western medical systems, particularly German medicine, and formal education in traditional medicine was abolished [23]. However, from the early twentieth century onward, physicians promoted the remodernization of traditional medicine by emphasizing a clinical framework centered on formula-symptom correspondence. Through these efforts, Kampo medicine gradually regained clinical legitimacy within a modern medical context. From the latter half of the twentieth century, Kampo formulas were incorporated into the national health insurance system and medical education, becoming an institutionalized component of Japan’s contemporary healthcare system [15,24,25].

As a result, TCM and Kampo medicine have become conceptually differentiated, reflecting divergent processes of institutionalization and localization in China and Japan. These differences are increasingly salient in institutional arrangements, theoretical articulation, and cultural identity [26].

Taken together, historical research has provided a clear account of how Kampo medicine evolved from the transmission of TCM to localized reorganization and eventual institutional differentiation. However, far less attention has been paid to how Kampo medicine and TCM are represented and reproduced in contemporary media. Large-scale, text-based analyses of how these two forms of medical knowledge are discursively differentiated in modern media remain limited.

### Kampo medicine and traditional chinese medicine in media discourse

Communication research recognizes that media discourse actively shapes public understandings of social reality through agenda-setting and framing processes [27]. Agenda-setting theory emphasizes how reporting frequency, continuity, and prominence shape public perceptions of issue importance [6,28], whereas framing theory focuses on how media guide interpretation through problem definition, causal attribution, and value evaluation [8,9].

Traditional medicine is widely regarded as a topic whose social meaning depends heavily on media discourse, given its entanglement with therapeutic efficacy, scientific evidence, and cultural identity [29]. Existing studies show that media coverage influences public assessments of credibility through the selection of expert voices, modes of risk presentation, and narrative tone [30].

Media practices also vary across cultural contexts in their framing of the efficacy, risks, and social value of traditional medicine. Research on TCM has largely compared Chinese and Western media. Chinese official media tend to frame TCM positively, emphasizing its scientific legitimacy and value as cultural heritage, whereas Western media often adopt a more cautious or skeptical stance, foregrounding issues of evidentiary standards and regulatory oversight [31–34]. By comparison, Kampo medicine is more frequently positioned as an integral part of the modern healthcare system and understood as operating alongside or as a complement to biomedical practice [35–38]. Despite these differences, existing research remains largely confined to macro-level, cross-regional comparisons. Within such approaches, TCM and Kampo medicine are often treated as equivalent instances of “traditional medicine” or “complementary and alternative medicine” [11,38] with limited attention to how medical knowledge sharing a common historical origin is differentiated and represented over time within a single media context.

### Media discourse analysis from a text-mining perspective

With the development of digital humanities and natural language processing, text-mining techniques have become an important approach for analyzing large-scale and longitudinal media corpora. Compared with traditional approaches based on manual coding and qualitative interpretation, text-mining enables the identification of latent discursive structures across extended time spans and large textual datasets, offering new empirical avenues for examining agenda dynamics and framing patterns [39–42].

Among existing approaches, Latent Dirichlet Allocation (LDA) has been widely used to identify latent topic structures in news reporting [43]. By modeling documents as mixtures of multiple topics, LDA extracts major thematic patterns from large corpora and allows their distribution and temporal variation to be examined [44]. It is therefore well suited to analyzing how media organize issues and allocate salience over time and has been broadly applied in communication studies [45].

However, LDA relies on bag-of-words assumptions and operates primarily at the document level, limiting its ability to capture fine-grained semantic relationships [46]. While effective in identifying what topics are covered, it is less capable of explaining how concepts are semantically associated or framed within that discourse.

To address this limitation, recent studies have increasingly introduced word embedding models to examine semantic structure at a more granular level. By learning patterns of word co-occurrence, these models represent words as continuous vectors, with semantically related terms located in close proximity. Among them, Word2Vec, proposed by Mikolov et al. [47,48], is widely used to analyze semantic neighborhoods and conceptual associations. In media discourse research, examining semantic neighborhoods of a keyword reveals stable patterns of co-occurrence, providing insights into discursive positioning and framing tendencies.

Despite these advances, most studies tend to rely on either topic models or word embedding models in isolation, with limited integration of thematic and semantic analysis. For knowledge systems such as Kampo medicine and TCM, which share a common historical origin but have diverged in modern contexts, single-layer approaches are insufficient to capture the complexity of media framing. This study therefore combines LDA and Word2Vec to examine the differentiated representation in Japanese mainstream media from two complementary perspectives: thematic structure and semantic proximity.

## Research design

### Analytical framework

This study conducts a multi-level text analysis of long-term coverage related to Kampo medicine and TCM in Japanese mainstream media. The research design is organized around two complementary analytical dimensions: (1) topic-level thematic structures in news discourse, and (2) semantic-level associations surrounding the key concepts of Kampo medicine and TCM.

At the topic level, LDA is applied to identify thematic structures in news discourse and examine their distribution and longitudinal changes across different periods. This analysis characterizes the overall thematic environments in which Kampo medicine and TCM are embedded.

At the semantic level, Word2Vec is used to analyze the semantic proximity and clustering patterns of terms associated with Kampo medicine and TCM within a shared semantic space. This approach enables a comparison of their semantic positions and patterns of representation, complementing topic-level analysis by capturing differences in conceptual associations.

**Fig 1:**
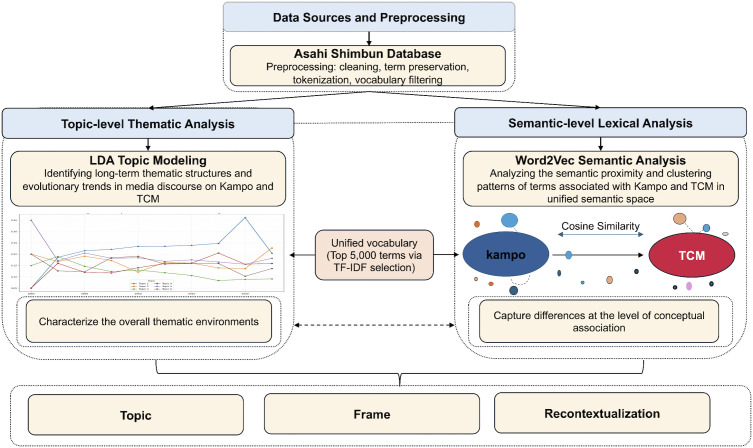
Analytical framework.

Although these procedures are methodologically independent, they are based on the same dataset and research focus. By integrating thematic and semantic analyses, the study reveals how medical knowledge with a shared historical origin is represented across multiple dimensions in contemporary media discourse, providing an empirical basis for understanding its discursive construction and recontextualization. The overall research workflow is presented in Fig 1.

### Data sources and data preprocessing

The data were retrieved from *Asahi Shimbun*’s official database, *Kikuzo II Visual* [49]. As a nationally influential newspaper, *Asahi Shimbun* provides extensive coverage of social, cultural, and policy issues, making it a suitable source for analyzing media discourse in Japan [50].

A keyword-based retrieval strategy was employed to construct the analytical corpus. The database was queried using the original-language keywords “漢方” (Kampo),“中医 AND 中国” (TCM AND China), “中国 AND 伝統医学” (China AND traditional medicine),and “漢医” (Kan’i; Kampo medical practice). The retrieved articles were subsequently screened and organized to form a curated corpus for analysis.

Duplicate articles were identified and removed based on exact matches of titles and publication dates, ensuring that each news item was uniquely represented in the corpus and retaining only the first occurrence. To ensure data quality, three researchers manually inspected a random sample of 250 articles, verifying title-content consistency, the accuracy of deduplication, and the completeness of article structure (e.g., title and body).

The final corpus consists of 5,602 articles published between August 5, 1984, and October 27, 2025, totaling approximately 7.59 million Japanese characters and an average length of 1,355 characters. This curated corpus constitutes the dataset used for all subsequent analyses.

As the source data are third-party copyrighted materials, the full-text corpus cannot be publicly shared. Instead, a minimal dataset and all analytical outputs required for reproducibility are provided as Supporting Information. In addition, a small set of representative news excerpts with English translations is included to illustrate the nature of the source data.

All texts underwent a standardized preprocessing procedure to support topic modeling and semantic analysis. This procedure consisted of four main steps: text cleaning, terminology protection, tokenization, and lexical feature selection. Preprocessing, vectorization, and modeling were implemented in Python (version 3.9.18), using the pandas (1.5.3), scikit-learn (1.2.2), and gensim (4.3.2) libraries. The specific processing steps are as follows:

First, non-semantic elements, including URLs, email addresses, telephone numbers, image captions and advertising labels, were removed to reduce noise.

Second, a terminology protection list was constructed to preserve domain-specific compound expressions. Based on the *East Asian Traditional Medicine Terminology* published by the Faculty of Medicine at Keio University, the list contains 5,572 entries covering diseases, therapies, medicinal substances, and diagnostic concepts. These terms were preserved as single units during tokenization.

For tokenization, multiple Japanese morphological analyzers were evaluated, and SudachiPy (developed by the National Institute for Japanese Language and Linguistics) was adopted, using C mode for relatively coarse-grained segmentation. Only nominal tokens were retained, and lemmatized base forms were used when available. The results were further refined using the terminology list and a customized stop-word list.

Finally, a document-term matrix weighted by TF-IDF was constructed to identify discriminative terms. The top 5,000 terms were retained as a controlled vocabulary which was used as input for both topic modeling and word embedding analyses to reduce the influence of high-frequency general terms.

### Topic modeling with LDA

This study employs Latent Dirichlet Allocation (LDA) to model topic-level thematic distributions in the preprocessed news corpus. LDA identifies latent topics in large text collections and estimates document-level topic composition, making it suitable for analyzing the formation and longitudinal evolution of thematic structures in news reporting [43,44].

The input model was constructed following the preprocessing procedures described in Sect 3.2. Using the filtered vocabulary, each document was represented as a bag-of-words (BoW), which served as the input corpus for LDA.

Selecting the number of topics (k) is a critical step in LDA modeling. Prior research has emphasized that there is no mathematically “correct” value for k; instead, the selection largely depends on research objectives and semantic interpretability [51]. Relying on a single statistical criterion may result in topics that are difficult to distinguish or interpret at the semantic level [52]. This study therefore adopts a two-staged approach combining quantitative metrics with semantic evaluation to determine k.

**Fig 2:**
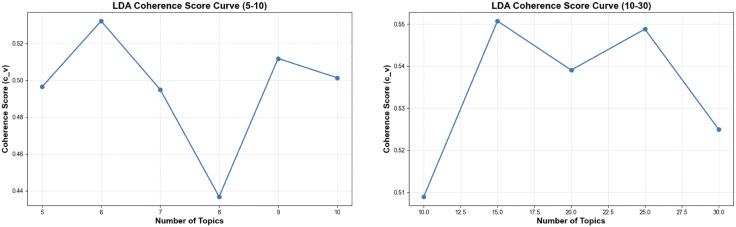
Coherence score across different numbers of topics.

In the first stage, topic coherence (c_v) was used to compare models across a broad range of topic numbers. LDA models were trained with k ∈ {10, 15, 20, 25, 30}, and coherence scores were calculated for each configuration, with k = 15 yielding the highest score, as shown in Fig 2.

However, pyLDAvis visualization revealed substantial topic overlap at k = 15, reducing inter-topic separability. The second stage therefore narrowed the search to k ∈ {5, 6, 7, 8, 9, 10}.

**Fig 3:**
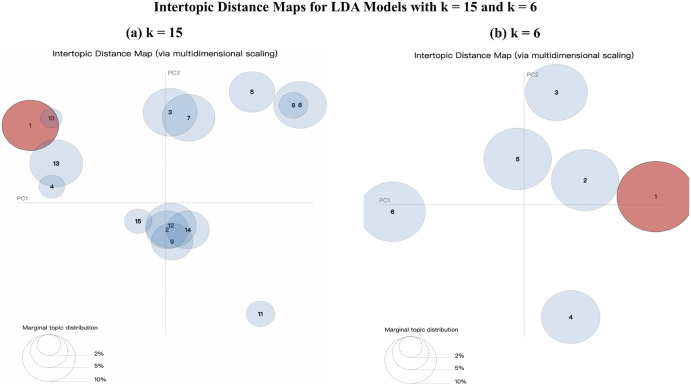
Intertopic distance map from pyLDAvis for LDA models with k = 15 and k = 6. For clearer visualization, only the intertopic distance maps are displayed. (a) shows the LDA model with 15 topics, and (b) shows the model with 6 topics. The numbers in the circles represent topic IDs, and circle size indicates the proportion of tokens in each topic.

Within this range, coherence scores and topic separation were jointly evaluated. The results show that k = 6 achieves a balance between coherence and topic distinctiveness, with relatively clear and well-separated structures in the visualization presented in Fig 3. Accordingly, k = 6 was selected as the final model.

The final LDA model was implemented using Gensim, with alpha = “auto” and eta = “auto” to reduce the influence of prior assumptions on topic distributions. Model training employed commonly used settings for news corpora, including passes = 10, iterations = 300, and chunksize = 2000, and a fixed random seed (random_state = 42) to ensure reproducibility. For each topic, the top 20 highest-weighted terms were extracted and interpreted together with representative documents exhibiting the highest topic probabilities. Topic labels were assigned based on a set of interpretive criteria, including the dominant semantic domain of each topic, the coverage of core high-frequency terms, consistency with representative articles, and clear differentiation from other topics. Three researchers independently interpreted and assigned topic labels. Discrepancies were subsequently discussed and resolved through iterative refinement until consensus was reached, ensuring the consistency and interpretive validity of the labeling process.

Based on the document-topic distributions, the study further examined longitudinal changes in topic prevalence. Documents were grouped by publication year, and topic frequencies and proportions were calculated for each decade. Time-series visualizations were then generated to compare topic trajectories across the full study period.

### Semantic analysis with Word2Vec

This study employs word embedding analysis to examine how Kampo medicine and TCM are semantically organized and positioned in Japanese media discourse, complementing topic modeling by capturing fine-grained conceptual associations. To this end, a Word2Vec model was trained on the news corpus to learn distributed representations of lexical items based on contextual co-occurrence, enabling analysis of semantic proximity within a shared vector space.

To ensure consistency with the topic modeling and reduce the influence of high-frequency general terms, the same controlled vocabulary derived during preprocessing was used. Specifically, the top 5,000 discriminative terms (based on TF-IDF) were retained, and tokenized documents were filtered accordingly for model training.

Word embeddings were trained using the Gensim implementation of Word2Vec with a skip-gram architecture (sg = 1). Following standard parameter ranges established in the original Word2Vec framework [47], preliminary comparisons were conducted to identify configurations suitable for the present news corpus. Vector dimensionality was evaluated at 100, 200, and 300, and context window sizes at 4, 6, and 8. Based on these comparisons, a vector size of 200 and a window size of 6 were selected to balance semantic representation and stability and to capture mid-range contextual relations in news text. The remaining parameters followed standard practice [47]: a minimum frequency of 3, 10 negative samples, and 10 training epochs.

To operationalize the concept of TCM as a unified concept, an anchor-based semantic center approach was adopted. A set of related terms- “TCM,” “Kan’i,” “Chinese medicine,” “Chinese medical science,” “traditional Chinese medicine,” “TCM pharmacy,” “Chinese herbs,” and “Chinese medicine and pharmaceuticals”-was selected as anchor words. The corresponding vectors were averaged to construct a composite representation, referred to as the “TCM semantic center”, which was used to measure the semantic proximity and identify its core neighborhood.

Cosine similarity between “漢方” (Kampo medicine) and “the TCM semantic center” was calculated to assess their proximity in the embedding space.

To compare semantic neighborhood structures, the top 30 most similar terms were extracted for both “漢方” (Kampo medicine) and “the TCM semantic center”. Their vectors were projected into a low-dimensional space for visualization. Principal component analysis (PCA) reduced the vectors to 30, followed by t-SNE mapping. A fixed random seed (random_state = 42) was used to ensure reproducibility.

## Results

### Topic structure of media coverage of Kampo Medicine and TCM

**Fig 4:**
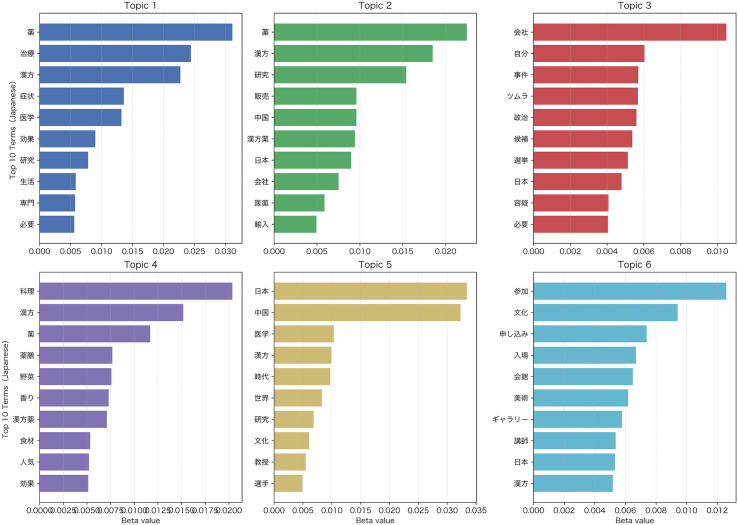
Top terms and beta values for the six topics identified by the LDA model. To facilitate visualization, only the top 10 original Japanese terms are displayed per topic. The full list of the top 15 terms with English glosses can be found in Table 1.

Fig 4 presents the top 10 highest-probability terms for each of the six topics identified by the LDA model. Terms are displayed in Japanese as they appear in the corpus, while topic labels and English glosses are provided in Table 1. Based on these terms and representative high-probability articles, the thematic characteristics of the six topics are summarized below.

**Table 1:** Topics identified by the LDA Model and their key terms.

Proportion	Topic Label	Top 15 Terms (Japanese terms with English glosses)
21.85%	medical events and health information	薬 (medicine), 治療 (treatment), 漢方 (Kampo), 症状 (symptoms), 医学 (medical science), 効果 (effects), 研究 (research), 生活 (daily life), 専門 (specialty), 必要 (necessity), 教授 (professor), 漢方薬 (Kampo medicine), 痛み (pain), 精神 (mental), 感染 (infection)
21.13%	raw material supply and industrial structure	薬 (medicine), 漢方 (Kampo), 研究 (research), 販売 (sales), 中国 (China), 漢方薬 (Kampo medicine), 会社 (company), 医薬 (pharmaceuticals), 輸入 (import), 製造 (manufacturing), 市場 (market), 製薬 (drug production), 農薬 (agrochemicals), 国内 (domestic), 農家 (farmers)
9.46%	political-economic and metaphorical uses	会社 (company), 事件 (incident), ツムラ (Tsumura), 政治 (politics), 候補 (candidate), 選挙 (election), 容疑 (suspicion), 自民 (LDP), 社長 (company president), 役員 (executive), 行政 (administration), 経営 (management), 民主 (Democratic Party), 議員 (legislator), 職員 (staff)
16.67%	dietary therapy and food-related practices	料理 (cuisine), 漢方 (Kampo), 薬 (medicine), 薬膳 (medicinal cuisine), 野菜 (vegetables), 香り (aroma), 食材 (ingredients), 人気 (popularity), 効果 (effects), 温泉 (hot springs), カレー (curry), 栄養 (nutrition), スープ (soup), 食事 (meals), 入浴 (bathing)
17.72%	international interaction	日本 (Japan), 中国 (China), 医学 (medical science), 漢方 (Kampo), 世界 (world), 研究 (research), 文化 (culture), 教授 (professor), 北京 (Beijing), アジア (Asia), 台湾 (Taiwan), 江戸 (Edo), 伝統 (tradition), 漢方薬 (Kampo medicine), 帰国 (return home)
13.17%	lifestyle and public information	参加 (participation), 文化 (culture), 申し込み (registration), 入場 (admission), 会館 (hall), 美術 (art), ギャラリー (gallery), 講師 (lecturer), 記念 (commemoration), 定員 (capacity), 募集 (recruitment), 主催 (organizer), 会場 (venue), 福祉 (welfare), 県立 (prefectural)

#### Topic 1: Medical events and health information.

Topic 1 is the most prominent topic in the corpus, with an average contribution of 21.85%. High-probability terms in this topic focus strongly on concrete symptoms, medical explanations, and treatment approaches, including terms such as 薬 (medicine), 治療 (treatment), 症状 (symptoms), and 効果 (effects).

Representative articles are primarily health information or medical guidance texts addressing common conditions such as menopausal disorders, cold sensitivity, pain and cognitive decline. These articles typically present symptom explanations followed by treatment options, often combining traditional and biomedical approaches.

#### Topic 2: Raw material supply and industrial structure.

Topic 2 accounts for 21.13% of the corpus. Terms cluster around raw material, production, and supply chains. Terms such as 中国 (China), 輸入 (imports), and 価格上昇 (price increases) contrast with 国内 (domestic), 国産 (domestically produced) and 栽培 (cultivation), indicating competing concerns over sourcing and production. Articles frequently report on domestic cultivation of medicinal crops, including local government projects, pharmaceutical industry involvement, and policies related to regional development.

#### Topic 3: Political-economy and metaphorical usage.

Topic 3 accounts for 9.46% of the corpus and is characterized by terms associated more with corporate, political, and social events than with medical or health issues per se. Although terms such as 薬 (medicine) still appear, they primarily function as indicators of corporate affiliation or industry background.

Articles within this topic fall into two main categories: reports on Kampo-related companies (e.g., Tsumura) and broader political or social commentary. In the latter, “Kampo” is often used metaphorically or rhetorically to frame policy effects, reform strategies, or administrative measures, often to describe gradual reform or delayed but cumulative effects.

#### Topic 4: Dietary therapy and food-related practices.

Topic 4 has an average contribution of 16.67% and is defined by high-probability terms related to food, cooking, and everyday life in connection with traditional medicine, including 料理 (cooking), 薬膳 (medicinal cuisine), 野菜 (vegetables), 香り (aroma), and 食材 (ingredients).

Articles typically introduce ingredients, recipes, or dietary practices, often framed through seasonal, regional, or personal experiences. Within this topic, traditional medical knowledge is presented as lifestyle-oriented through everyday dietary practices, shifting emphasis from its clinical attributes to its role as a mode of living.

#### Topic 5: International interaction.

Topic 5 accounts for 17.72% of the corpus and includes terms with transnational, historical, and cultural orientations. Articles cover cultural exchange events, academic collaboration, and historical commemoration.

In these articles, concepts related to traditional medicine serve not as primary objects of discussion but as narrative background or symbolic references. They are frequently mentioned as one cultural domain within the broader history of Sino-Japanese exchange, supplementing broader themes of cultural interaction and international relations.

#### Topic 6: Lifestyle and public information.

Topic 6 has an average contribution of 13.17% and consists mainly of standardized lifestyle guidance and announcements for cultural or public activities. Terms concentrate on participation, venues, and registration.

Articles typically appear in local editions or information columns, covering exhibitions, lectures, public health events, cultural festivals, and community gatherings. In this topic, traditional medicine appears as one component of event content. Its primary function is informational, serving to notify readers of activities and provide practical details.

### Diachronic changes in topic proportions

**Fig 5:**
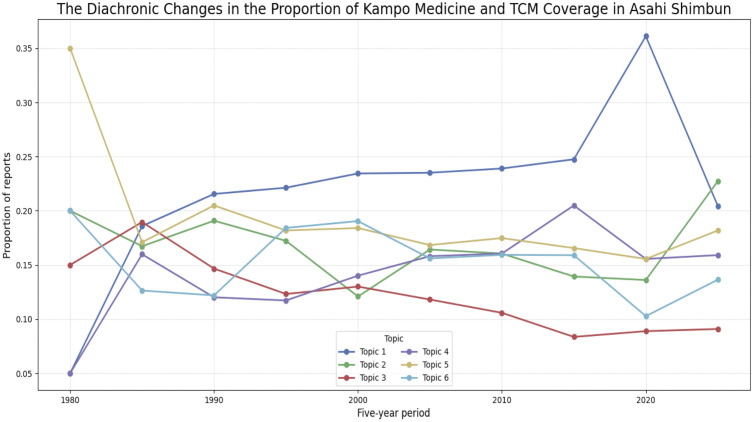
The diachronic changes in the proportion of Kampo Medicine and TCM coverage in *Asahi Shimbun.*

After normalizing the proportions of documents assigned to each topic across periods, the results show that the thematic structure of *Asahi Shimbun*’s coverage of Kampo medicine and TCM is not stable over time. Instead, it undergoes pronounced shifts in emphasis across historical stages, as shown in Fig 5. Proportions fluctuate dynamically, reflecting changes in the focal concerns and discursive functions.

Across the entire period, Topic 1 (medical events and health information) remains consistently prominent and constitutes a core theme. Although its proportion is relatively low in the early 1980s, it shows a sustained increase from the mid-1980s onward, reaching a peak during 2015–2020. Despite a modest decline in the most recent interval, it remains among the most prominent topics, forming a clear contrast between the early and later stages of the time series.

Topic 2 (raw material supply and industrial structure) displays a more segmented trajectory. It accounts for a relatively high proportion in the early stage, declines around 2000 and subsequently recovers with renewed fluctuations. After 2020, it shows a clear upward trend and becomes one of the more prominent themes alongside Topic 1.

Topic 3 (political-economic and metaphorical uses) consistently occupies a relatively small proportion. It shows a noticeable peak in the mid-1980s, followed by a prolonged period at low levels. This pattern suggests that its prominence is driven by specific events, political issues, or socially oriented articles during particular periods.

Topic 4 (dietary therapy and food-related practices) displays a gradual increase followed by stabilization. It begins with a low proportion in the early period, rises steadily from the mid-1980s, and remains within a moderate range thereafter. Although minor fluctuations occur, it maintains a stable secondary presence across periods. Topic 5 (international interaction) peaks prominently in the early 1980s, representing a relatively high share of coverage in the early period. This pattern suggests that Kampo medicine and TCM were more frequently framed within contexts of historical reflection and cross-national exchange at that time. In later periods, the topic maintains a stable but dispersed presence without concentrated peaks. Topic 6 (lifestyle and public information) shows limited variation throughout the time series. Its proportion remains within a narrow range, indicating a structurally stable position within the reporting framework.

Taken together, the diachronic results demonstrate that coverage of Kampo medicine and TCM in *Asahi Shimbun* is anchored by medical events and health information (Topic 1) as a long-term core theme. Over the past two decades, raw material supply and industrial structure (Topic 2) as well as dietary therapy and food-related practices (Topic 4) have become more prominent, contributing to a more diversified thematic structure. In contrast, political-economic and metaphorical uses (Topic 3) follow an episodic, event-driven pattern, while international interaction (Topic 5) and lifestyle and public information (Topic 6) remain relatively stable background themes.

### Semantic analysis with Word2Vec

Beyond the topic structure, semantic analysis provides additional insight into how Kampo medicine and TCM are associated in *Asahi Shimbun*. Using a Word2Vec model, this study extracts two sets of semantic neighborhoods: one centered on Kampo, and the other based on an anchor-based semantic center constructed from multiple TCM-related designations. For each, the top 20 most similar terms are identified to compare neighborhood composition and clustering patterns (Table 2).

**Table 2:** Top 20 terms in the semantic neighborhoods of Kampo and the TCM semantic center.

Rank	Kampo semantic neighborhood (Japanese terms with English glosses)	Cosine Similarity	TCM Semantic Center Neighborhood (Japanese terms with English glosses)	Cosine Similarity
**1**	薬 (medicine)	0.6906	中医学 (traditional Chinese medicine)	0.9241
**2**	せき止め (cough suppressant)	0.5671	中医薬 (Chinese medicinal drugs)	0.9095
**3**	薬理 (pharmacology)	0.5433	中国医学 (Chinese medicine)	0.8977
**4**	茯苓 (poria)	0.5390	中医 (Chinese medicine)	0.8571
**5**	中薬 (Chinese herbal drugs)	0.5281	伝統医学 (traditional medicine)	0.8277
**6**	健胃 (stomachic)	0.5269	西洋薬 (Western pharmaceuticals)	0.7910
**7**	桂皮 (cinnamon bark)	0.5129	富山医科薬科大学 (Toyama Medical and Pharmaceutical University)	0.7699
**8**	効能 (therapeutic efficacy)	0.5037	和漢薬 (Japanese-Chinese herbal medicine)	0.7682
**9**	効用 (medicinal effect)	0.4837	中薬 (Chinese herbal drugs)	0.7678
**10**	古来 (since ancient times)	0.4783	地域医療 (community healthcare)	0.7645
**11**	傷寒論 (Treatise on Cold Damage)	0.4782	現代医学 (modern medicine)	0.7622
**12**	張仲景 (Zhang Zhongjing)	0.4747	慢性肝炎 (chronic hepatitis)	0.7572
**13**	桂枝 (cinnamon twig)	0.4741	東洋医学 (Oriental medicine)	0.7565
**14**	補中 (tonifying the middle)	0.4679	洋薬 (Western pharmaceuticals)	0.7439
**15**	滋養 (nourishment)	0.4677	西洋医学 (Western medicine)	0.7414
**16**	胃腸 (gastrointestinal system)	0.4659	民間薬 (folk medicine)	0.7383
**17**	葛根 (kudzu root)	0.4649	東洋医 (Oriental medicine practitioner)	0.7311
**18**	クコ (goji berry)	0.4644	大学医学 (university medicine)	0.7274
**19**	万病 (all diseases)	0.4643	東京薬科大学 (Tokyo University of Pharmacy)	0.7248
**20**	虚弱 (physical weakness)	0.4642	自覚症 (subjective symptoms)	0.7239

**Fig 6:**
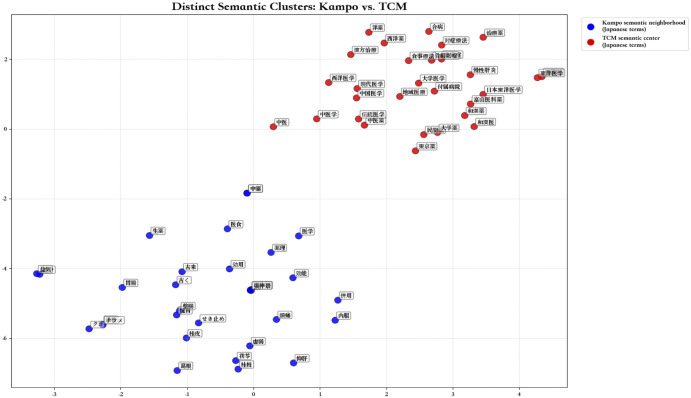
Semantic separation in vector space: t-SNE projection of the Kampo neighborhood and the TCM semantic center. Terms are presented in Japanese as they appear in the original corpus. English glosses of key terms are provided in Table 2.

In the word embedding space, the cosine similarity between Kampo and the TCM semantic center is 0.42, indicating that the two concepts retain an identifiable semantic association consistent with their shared historical origin, while remaining far from semantically overlapping. This observation is further supported by dimensionality-reduced visualizations. As shown in Fig 6, the t-SNE visualization further supports this distinction, revealing a clear separation between the Kampo semantic neighborhood (blue points) and the TCM semantic center (red points), which occupy distinct positions in the embedding space.

A closer examination shows that the Kampo neighborhood is strongly oriented toward therapeutic effects, symptom-related expressions, and concrete materia medica. High-similarity terms include expressions referring to treatment outcomes or bodily regulation, such as “せき止め”(cough suppression), “健胃”(stomachic effects), “鎮痛”(analgesia), “滋養”(nourishment), and “整腸”(intestinal regulation). In addition, the neighborhood includes numerous names of specific crude drugs, such as “茯苓” (poria), “桂枝” (cinnamon twig), “葛根” (kudzu root), “クコ” (goji berry), “ナツメ” (jujube). Notably, classical figures and texts associated with classical Chinese medical traditions—such as Zhang Zhongjing and *Shanghan Lun*—do not appear as central elements within the TCM semantic center but instead occur within the Kampo neighborhood, where they function as references to prescription origins or sources of historical authority.

By contrast, the TCM semantic center is characterized primarily by abstract and institutional terminology. Its closest neighbors consist largely of classificatory labels, including traditional Chinese medicine, Chinese medicine, Oriental medicine, and traditional medicine, as well as terms associated with modern medical systems and institutional contexts, such as modern medicine, Western medicine, university medicine, affiliated hospitals and symptomatic treatment. Taken together, this neighborhood exhibits a classificatory and system-oriented profile.

From a structural perspective, overlap between the neighborhoods is minimal, with only the generic term “Chinese herbal medicine” appearing in both. The two clusters differ substantially in both composition and aggregation patterns: the Kampo neighborhood pivots on concrete therapeutic entities, whereas the TCM semantic center is organized around abstract classificatory and institutional frameworks. These results indicate clear differentiation in patterns of lexical co-occurrence and semantic proximity in *Asahi Shimbun*’s coverage.

## Discussion

### Thematic configuration and evolution of Kampo and TCM coverage in *Asahi Shimbun*

Sustained media attention to an issue increases its perceived importance in public cognition. Beyond salience, the media also reshape the social meaning and discursive functions of reporting objects by embedding them within different public issue frames [6,28]. This process is rooted in specific socio-historical contexts and reflects ongoing interactions between media agendas and broader social structures. In other words, shifts in issue meaning are closely intertwined with historical circumstances, as the media not only respond to social change but also participate in the construction of public meanings [53,54]. The findings of this study show that, over four decades of reporting, *Asahi Shimbun* has dynamically reorganized its agenda structure in response to such contextual shifts.

Based on topic modeling results, *Asahi Shimbun* constructs an agenda environment composed of multiple functionally differentiated topics, including medical events and health information, raw material supply and industrial structure, political-economic and metaphorical uses, dietary therapy and food-related practices, international interaction, and lifestyle and public information. While these topics persist across the corpus, their relative prominence varies substantially. Rather than being represented as static medical objects, Kampo medicine and TCM are repeatedly embedded within different public issues frames, acquiring shifting visibility and distinct functional roles within the news agenda.

From a diachronic perspective, clear differences emerge between early reporting and that of the past two decades. In the 1980s, coverage was more frequently situated within international and historical contexts (Topic 5), where Kampo and TCM functioned primarily as symbolic objects of cross-cultural exchange. From the 1990s onward, however, the agenda gradually shifted. Topics related to medical events and health information as well as raw material supply and industrial structure (Topics 1 and 2), gained prominence and became dominant in later stages. This transition reflects changes in domestic social needs and policy priorities, while also illustrating agenda-setting mechanisms through which, shifts in issue salience shape public perceptions of importance and interpretation. Among the identified topics, the expansion of Topic 1 (medical events and health information) is particularly indicative. Its increasing prominence corresponds to a transformation in the public functions attributed to traditional medicine in media discourse. As Japanese society has entered a phase of advanced population aging, chronic disease management, health maintenance, and preventive medicine have become central public concerns. Correspondingly, reporting has shifted toward more practical orientations targeting individual readers. This is reflected not only in increased reporting frequency but also in reporting practices centered on symptom explanation, risk communication, and treatment options.

During and after the outbreak of the COVID-19 pandemic, public health risks further intensified this shift toward practical, health-focused reporting. Increased attention to immune regulation and disease prevention further reinforced the embedding of traditional medicine within health information frameworks. Through selective emphasis and repetition, media amplified attention to health-related issues [55], while reinforcing journalistic dynamics contributed to the formation of news waves and the perceived urgency of such topics [56]. The prominence of health-related topics reporting during this period exemplifies these mechanisms in a specific historical context.

In contrast, Topic 2 (raw material supply and industrial structure) exhibits a stage-based pattern. Although present in early reporting, its increasing prominence in recent decades reflects a growing alignment between traditional medicine and concerns over resource security, supply chain dependence, and domestic production. This shift reflects the growing salience of supply chain stability in a global context and highlights the discursive flexibility of traditional medicine, whose meaning is redefined and reframed in relation to dominant public concerns.

Topic 3 (political-economic and metaphorical uses) demonstrates a distinctly event-driven pattern. Rather than forming a stable domain, it emerges in response to specific corporate, legal, or political events. In such contexts, Kampo medicine or TCM functions as a referential or rhetorical resource. Notably, Kampo is frequently used metaphorically to describe gradual reform or delayed but cumulative effects, often in contrast to more radical or disruptive interventions. This usage indicates a degree of semantic portability: once established as a familiar and legitimate concept, Kampo can be abstracted and redeployed in broader political and social discourse. Topic 3 thus illustrates that medical knowledge functions as a symbolic resource beyond its original domain.

Topic 4 (dietary therapy and food-related practices) reflects a transition from marginality to stability. With the rise of health-oriented lifestyles and self-care practices, dietary and medicinal cuisine content has become a routine component of reporting. Although not dominant, its sustained presence indicates a stable linkage between traditional medicine and everyday life, positioning it as an intermediate domain between medical and cultural discourse.

Topic 5 (international interaction) is more prominent in early reporting, highlighting the role of Kampo medicine and TCM in cross-cultural exchange. As reporting priorities shifted toward health and industry, its relative salience declined. Nevertheless, it did not disappear entirely but persists as a background narrative resource supporting discussions of international exchange and cultural comparison.

Topic 6 (lifestyle and public information) remains stable throughout the study period, reflecting the newspaper’s fundamental role as a public information provider, through which traditional medicine is routinely incorporated into event announcements or cultural notices.

Overall, through the coexistence and reconfiguration of multiple topics and *Asahi Shimbun* has continuously reshaped the public meanings and discursive functions of Kampo medicine and TCM. Media attention has shifted from an international-cultural frame toward health and industrial concerns, demonstrating the malleability of traditional medical knowledge within media discourse. By embedding such knowledge within dominant public issue frames, the media sustain its salience while continually redefining its thematic positioning. These findings provide a contextual foundation for further analysis of how Kampo medicine and TCM are differentially framed at the semantic level. More broadly, they empirically illustrate that agenda setting is a dynamic process deeply intertwined with socio-historical contexts, in which the media actively shape public understanding of complex issues rather than merely reflecting prevailing concerns.

### Media framing differentiation between Kampo Medicine and traditional Chinese Medicine

Following Entman’s [9] conception of framing as the selection and salience-making of certain aspects of reality, this study focuses on how *Asahi Shimbun* has consolidated two differentiated discursive frames for Kampo medicine and TCM, shaped by the systematic organization of thematic entry points, conceptual structures, and patterns of semantic adjacency. At the level of thematic entry points, topic modeling results reveal a clear differentiation in how Kampo medicine and TCM are positioned within the overall reporting structure. Within the most dominant health-related topic (Topic 1), high-probability articles are overwhelmingly centered on Kampo medicine, anchoring it within a practical frame oriented toward symptom explanation, health management, and treatment choice. This positioning constructs Kampo medicine as a localized, practice-oriented medical resource applicable to everyday health maintenance.

A similar tendency is observed in the topic of raw material supply and industrial structure (Topic 2), where Kampo-related reporting is embedded in industrial and policy-related concerns. In this context, TCM functions primarily as an external reference point through which issues of raw material dependence and supply chain security are articulated, reinforcing a domestically oriented industrial narrative.

In political-economic and social commentary contexts (Topic 3), both traditions may be mobilized as rhetorical resources; however, Kampo medicine retains greater symbolic capital due to its institutionalized domestic status, whereas TCM lacks a stable narrative role in such contexts.

Within topics related to dietary culture and lifestyle services (Topics 4 and 6), TCM attains a relatively stable discursive presence through concepts such as “medicine-food homology”, where it is framed primarily as a cultural or lifestyle-oriented philosophy.

International interaction (Topic 5) initially functioned as an important platform for both traditions, reflecting TCM’s symbolic role as an emblem of cross-cultural exchange. Over time, however, this role has become increasingly backgrounded as media attention shifted toward domestic health and industrial issues.

Taken together, this uneven thematic configuration indicates that *Asahi Shimbun* embeds Kampo medicine within core functional frames that resonate with domestic priorities, while directing TCM toward comparative cultural or explanatory roles. This differentiation is reinforced at the micro-level of lexical co-occurrence. Word embedding analysis shows that Kampo medicine and TCM exhibit limited overall semantic similarity (cosine similarity = 0.42) and form distinct semantic neighborhoods. The semantic network surrounding Kampo medicine is organized around a “efficacy-symptom-materia medica” structure, clustering terms related to treatment outcomes, crude drug names, and pharmaceutical forms. This configuration situates Kampo medicine within a semantic space of concrete bodily experience and therapeutic practice, consolidating its frame as a practice-oriented medical resource involved in everyday health management.

By contrast, the TCM semantic center is organized primarily around a “classification-system-institution” structure. It is framed not as a set of actionable therapies, but as a comprehensive yet external “knowledge system” that requires explanation and positioning in relation to modern medicine. A particularly salient finding concerns the placement of classical figures and texts associated with TCM. Canonical figures such as Zhang Zhongjing and texts such as the *Shanghan lun* are located closer to Kampo medicine than to the semantic center of TCM. This “misplacement” reveals a recontextualization process involving selective extraction and re-embedding, one that departs from existing models of traditional medical knowledge adaptation in modern nation-states, which typically emphasize integration in formal institutions, marginalization as unscientific knowledge, or coexistence alongside biomedical frameworks. However, these models tend to treat traditional medical knowledge as a unified object. The present findings suggest that recontextualization may also operate through the selective differentiation and redistribution of homologous knowledge within a shared cultural-historical field. In this case, classical Chinese medical knowledge is detached from its original context and reconfigured as symbolic capital to bolster the authority and historical legitimacy of Kampo. Shared medical heritage thus ceases to function primarily as an object of genealogical narration and becomes a discursive tool for contemporary Japanese medical narratives.

At a broader level, this framing differentiation reflects the interaction of cultural orientations, institutional settings, and journalistic routines. Building on existing studies of TCM representations in the Chinese context, this study situates its findings within a broader comparative perspective. Chinese media typically frame TCM as a legitimate and institutionalized component of the national healthcare system, often emphasizing its scientific value and cultural significance [33,34]. By contrast, the Japanese case reveals a different pattern. Rather than foregrounding explicit evaluative positions, Japanese media more often situate TCM in comparative, explanatory, or referential contexts, in contrast to the domestically embedded image of Kampo medicine.

Such framing has important implications for understanding media effects in health communication. It shapes how homologous medical knowledge systems are perceived and related to one another. In this respect, the findings align with existing research showing that media framing influences public judgments of credibility, legitimacy, and perceived risk regarding traditional and alternative medicine [57,58]. Importantly, this study suggests that media effects extend beyond shaping general attitudes. In the Japanese context, framing practices contribute to how relationships between related medical traditions are perceived, influencing how audiences understand their relative positions within a shared knowledge domain.

This pattern can be further understood in relation to Japan’s specific socio-cultural orientations and institutional context. Through processes of scientific validation and standardization, Kampo has been progressively incorporated into evidence-based medical practice, distinguishing it from TCM, which is more often associated with experience-based knowledge [59]. Media representations therefore align Kampo medicine with values emphasized in biomedical discourse, including safety, standardization, and clinical evidence. This alignment allows Kampo to function as a legitimate and complementary component of the healthcare system without challenging the dominant role of biomedicine. By contrast, TCM is more often separated from both Kampo medicine and biomedical practice, reinforcing its status as an externalized form of traditional knowledge. Media framing thus tends to maintain a hierarchy of medical knowledge in which biomedicine occupies the center, Kampo medicine functions as a domestically integrated supplement, and TCM remains comparatively marginal.

At the same time, contemporary Chinese state discourse increasingly frames TCM as an instrument of national strategy and cultural soft power [60]. Consequently, *Asahi Shimbun* often situates TCM within macro-contexts such as “Chinese cultural export,” and “international relations.” Within these frames, TCM’s medical properties are frequently overshadowed by political and cultural symbolism, reinforcing its image as “external” and “systemic” in contrast to the “internal” and “practical” image attached to Kampo medicine.

This pattern also resonates with the broader historical reconstruction of modern knowledge in East Asia. As Flowers [61] argues, twentieth-century East Asian nations commonly sought to reposition their medical traditions by decentering China. In Japan, Kampo was delineated as an autonomous East Asian knowledge system, rather than a subsidiary of TCM. This conceptual shift provided the institutional and discursive groundwork for the contemporary media treatment of Kampo medicine and TCM as distinct semantic entities.

In summary, *Asahi Shimbun* has constructed and stabilized two distinct media frames over time. Kampo medicine is framed as an internalized, practical, and de-historized domestic medical resource, centered on functionality and safety. In contrast, TCM is framed as an external, systemic body of knowledge and a cultural symbol oriented toward comparison and categorization. This sustained differentiation emerges from the interaction of journalists’ norms, organizational preferences, political actors, institutional settings, and broader cultural expectations [62]. Ultimately, it reflects a process of selective extraction, functional reorganization, and recontextualization through which shared medical knowledge is restructured into differentiated discursive forms.

## Conclusion

Based on topic modeling and word embedding analysis of over four decades of coverage in *Asahi Shimbun*, this study examines the long-term discursive construction of Kampo medicine and Traditional Chinese Medicine (TCM), two traditions with a shared historical origin. The analysis operates at two levels, focusing on thematic structure and semantic associations. The findings demonstrate that sustained thematic and semantic configurations have directed Kampo medicine and TCM along two separate discursive paths, characterized respectively by localization and othering.

At the thematic level, media coverage has been dynamically reorganized in response to domestic social needs and policy concerns. Kampo medicine is consistently embedded within core public issues such as healthcare provision and industrial security, whereas TCM is more frequently situated within frames of cultural comparison or positioned as an external reference point. This diachronic restructuring assigns the two traditions distinct public roles and functional orientations.

At the semantic level, stable patterns of lexical co-occurrence further consolidate this distinction. The semantic field surrounding Kampo medicine is concentrated on therapeutic effects, symptoms, and concrete materia medica, framing it as a safe, usable, and internally integrated medical resource. By contrast, the semantic organization of TCM emphasizes conceptual classification and systemic attributes, constructing it as an external knowledge system requiring definition and contextualization. Together, thematic differentiation and semantic organization reinforce clear epistemic boundaries between the two.

Taken together, *Asahi Shimbun*’s coverage constitutes a systematic process of meaning production embedded in the context of the modern nation-state. Media representations not only reflect broader social transformations but also actively participate in the construction of cultural ownership, industrial interests, and collective identity, thereby shaping public cognition and knowledge boundaries.

These findings make contributions in several respects. At the theoretical level, this study operationalizes recontextualization within the analysis of long-term media texts, demonstrating how agenda-setting and framing enable the systematic localization and othering of homologous knowledge. In doing so, it extends the applicability of recontextualization theory within communication research, particularly in the analysis of knowledge circulation and cross-cultural media discourse.

At the methodological level, by integrating topic modeling with word embedding analysis, this study establishes a correspondently analytical pathway between thematic structure and semantic organization, allowing agenda configuration and conceptual framing to be examined in a coordinated manner. This approach provides a replicable framework for investigating the long-term media evolution of complex knowledge objects.

At the practical level, this research shifts attention from the frequently examined othering of traditional medicine in Western contexts to processes of knowledge competition and identity reconstruction within East Asian settings. Specifically, for the medical and health communication fields, the findings highlight how media discourse shapes public understanding, credibility, and perceived legitimacy of traditional medical knowledge. Greater awareness of how shared medical heritage is selectively framed may support more context-sensitive communication and help reduce unnecessary polarization, thereby facilitating more constructive engagement among TCM, Kampo, and biomedical systems.

Several limitations should be acknowledged. The present analysis is based on a single newspaper corpus and cannot reflect the full spectrum of media discourse in Japan. For instance, regional newspapers may emphasize local medical practices and policies, while digital and social media tend to carry more contentious, user-driven frames. Professional medical publications may foreground evidence-based and regulatory viewpoints that differ from general news coverage. Future research could extend this inquiry by comparing multiple communicative arenas and incorporating qualitative discourse approaches to examine specific discursive strategies in greater detail, thereby providing a more comprehensive account of the recontextualization of traditional medical knowledge in cross-cultural contexts.

## Supporting information

S1 File Sample News Excerpts. Sample of five news articles used as illustrative excerpts. The original Japanese texts are provided with brief English summaries for reference.
(XLSX)

S2 File Preprocessed Tokens. Minimal dataset containing document IDs, publication dates, and tokenized text used for all analyses.
(XLSX)

S3 File Document-Topic Distributions (k = 6). Matrix of topic proportions for each document generated by the final LDA model.
(XLSX)

S4 File Top Terms per Topic (k = 6). Top 20 terms and their weights for each topic identified by the LDA model.
(XLSX)

S5 File Kampo Semantic Neighbors. Top 20 terms most similar to “Kampo” based on Word2Vec cosine similarity.
(XLSX)

S6 File TCM Semantic Neighbors. Top 20 terms most similar to the TCM semantic center based on Word2Vec cosine similarity.
(XLSX)

S7 File East Asian Traditional Medicine Terminology List. Terminology list derived from Keio University East Asian Traditional Medicine resources, used for term standardization and protection during preprocessing.
(PDF)

S8 File README. Description of the content, structure, and usage of all supporting information files.
(TXT)

S9 File Data Cleaning Code. Python script for text cleaning and preprocessing of raw news data.
(TXT)

S10 File LDA Modeling Code. Python script for topic modeling, including TF-IDF filtering, coherence evaluation, and final model training.
(TXT)

S11 File Word2Vec Semantic Analysis Code. Python script for training word embeddings and conducting semantic similarity analysis.
(TXT)
