# Supplementary material for: From a shared origin to divergent frames: A computational analysis of media representations of Kampo Medicine and Traditional Chinese Medicine in Asahi Shimbun
Source: PLoS One. 2026 Jun 30;21(6):e0351860. doi: 10.1371/journal.pone.0351860 (PMC13318050; doi:10.1371/journal.pone.0351860)
Supplement: S7 File — Terminology list derived from Keio University East Asian Traditional Medicine resources, used for term standardization and protection during preprocessing. (PDF) [file pone.0351860.s007.pdf]

検索表示（原表示の複数語を分離）	IST原表示	Code	Term	Definition/Description	定義/記述
傳統醫學		0.0.1	traditional medicine	the sum total of knowledge, skills and practice of holistic care for maintenance of health and treatment of disease based on indigenous theories,beliefs and experiences handed down from generation to generation	世代を超えて受け継がれてきた土着の理論、信念および経験に基づく、健康維持および疾患治療のためのホリスティックケア（全人的医療）の包括的な知識、技術および実践。
中醫學	中醫學;中醫	0.0.2	traditional Chinese medicine	the traditional medicine that originated in China, and is characterized by holism and treatment based on pattern identification/syndrome differentiation	中国の伝統医学。全体論と証の診断に基づく治療を特徴とする。
中醫	中醫學;中醫	0.0.2	traditional Chinese medicine	the traditional medicine that originated in China, and is characterized by holism and treatment based on pattern identification/syndrome differentiation	中国の伝統医学。全体論と証の診断に基づく治療を特徴とする。
東洋醫學		0.0.3	Oriental medicine	a general term for traditional medicine practiced in East Asian countries, e.g., Japan and Korea	東アジア諸国（日本、韓国など）で実践されている伝統医学。
漢方	漢方;漢方醫學	0.0.4	Kampo medicine	the medicine traditionally practiced in Japan, based on ancient Chinese medicine	日本で伝統的に実践されている医学。古代中医学に基づく。
漢方醫學	漢方;漢方醫學	0.0.4	Kampo medicine	the medicine traditionally practiced in Japan, based on ancient Chinese medicine	日本で伝統的に実践されている医学。古代中医学に基づく。
韓醫學		0.0.5	traditional Korean medicine	the medicine traditionally practiced in Korea, based on ancient Chinese medicine, which focuses principally on constitutional approaches	韓国で伝統的に実践されている医学。古代中医学に基づく。体質に基づくアプローチを中心とする。
越醫學		0.0.6	traditional Vietnamese medicine	the medicine traditionally practiced in Vietnam, based on ancient Chinese medicine	ベトナムで伝統的に実践されている医学。古代中医学に基づく。
藏醫學		0.0.7	Tibetan medicine	the medicine traditionally practiced in Tibet	チベットで伝統的に実践されている医学。
蒙醫學		0.0.8	Mongolian traditional medicine	the medicine traditionally practiced in Mongolia	モンゴルで伝統的に実践されている医学。
維醫學		0.0.9	Uyghur medicine	the medicine traditionally practiced by the Uyghur	ウイグル民族によって伝統的に実践されている医学。
中西醫結合		0.0.10	integration of traditional Chinese and Western medicine	a process of ongoing development of traditional Chinese medicine involving the incorpdration of modern scientific knowledge and measures as well as blending of key aspects of traditional Chinese medicine and modern Western medicine	最新の科学的知見と手段を導入し、中医学と現代西洋医学双方の特徴を活かして医療を実践する中医学の現状。
中醫基礎理論		0.0.11	basic theory of traditional Chinese medicine	the branch of traditional Chinese medicine dealing with the basic concepts, theories, rules and principles	中医学の一部門で、基本的概念、理論、基準、および原則を扱う。
中醫診斷學		0.0.12	traditional Chinese diagnostics	the branch of traditional Chinese medicine dealing with the procedure and practice of examining patients, determining diseases and differentiating syndromes/identifying patterns of signs and symptoms of diseases, also called traditional Chinese medical diagnostics	中医学の一部門で、患者の診察、疾患の診断、疾患の徴候と症状による証の診断を扱う。traditional Chinese medical diagnosticsとも呼ばれる。
中藥學		0.0.13	traditional Chinese pharmacy	the branch of traditional Chinese medicine dealing with the source, nature, collection, processing, dispensing, actions, effects and uses of Chinese medicines	中医学の一部門で、漢方薬の原料、性質、収集、加工、調剤、作用、効果、および使用を扱う。
方劑學		0.0.14	formula study	the branch of traditional Chinese medicine concerned with therapeutic principles, combination of medicinal ingredients, composition of prescriptions and the clinical uses of herbal medicines, also called Chinese herbal formula study	中医学の一部門で、治療の原理、薬効成分の組み合わせ、処方薬の組成、および生薬療法の臨床使用を扱う。中医方剤学とも呼ばれる。
中藥炮製學		0.0.15	processing of herbal medicinals	the branch of traditional Chinese medicine dealing with the theory, technology, specifications and standards of processing herbal medicine, also called preparation and processing of Chinese herbal medicine	中医学の一部門で、漢方薬加工の理論、技術、規格および基準を扱う。中医製剤学とも呼ばれる。
經絡學		0.0.16	meridian and collateral (study)	the branch of acupuncture concerned with the study of structural connection, physiology, pathology, diagnostics and therapeutic principles, on the basis of meridian phenomena, also known as channel and networks study	鍼治療の一部門で、経絡現象に基づく構造的な接続、生理学、病理学、診断法および治療原理を扱う。channel and networks studyとしても知られる。
輸穴學	輸穴學;經穴學	0.0.17	acupuncture points (study)	the branch of acupuncture dealing with the study of the location, action and indication of acupuncture points and their related theories	鍼治療の一部門で、経穴の位置、作用と適応症、および関連する理論を扱う。
經穴學	輸穴學;經穴學	0.0.17	acupuncture points (study)	the branch of acupuncture dealing with the study of the location, action and indication of acupuncture points and their related theories	鍼治療の一部門で、経穴の位置、作用と適応症、および関連する理論を扱う。
中醫推拿學		0.0.18	traditional chinese tuina	the branch of traditional Chinese medicine concerned with the principles and clinical use of tuina (massage) therapy	中医学の一部門で、推拿（マッサージ）療法の原理と臨床使用を扱う。
中醫養生學		0.0.19	traditional Chinese life nurturing	the branch of traditional Chinese medicine concerned with promotion of health, prevention of disease and longevity, also called traditional Chinese health cultivation	中医学の一部門で、健康増進、疾患予防、長寿を扱う。traditional Chinese health cultivationとも呼ばれる。
中醫康復學		0.0.20	traditional Chinese rehabilitation	the branch of traditional Chinese medicine concerned with the restoration of function after injury or illness, also called traditional Chinese medicine rehabilitation	中医学の一部門で、外傷または病後の機能回復を扱う。中医リハビリテーション学とも呼ばれる。
中醫護理學		0.0.21	traditional Chinese nursing	the branch of traditional Chinese medicine dealing with the study, methods and clinical application of nursing care	中医学の一部門で、看護の研究、方法、および臨床応用を扱う。
溫病學		0.0.22	warm disease (study)	the branch of traditional Chinese medicine dealing with the study of pathogenesis, diagnosis, treatment and prevention of warm pathogen diseases, also known as warm pathogen disease (study)	中医学の一部門で、温病の病因、診断、治療および予防を扱う。warm pathogen disease （study）としても知られる。
養生		0.0.23	life nurturing	traditional health-care to promote health, prevent disease and enhance longevity, also called health preservation/cultivation	健康増進、疾患予防、および長寿のための伝統的な健康管理法。health preservation/cultivationとも呼ばれる。
康復		0.0.24	rehabilitation	the treatment of an ill, injured, or disabled patient with the aim of restoring normal health and function or to prevent a disability from deteriorating	正常な健康と機能の回復および障害の悪化予防を目的とした、疾患患者、外傷患者および障害患者の治療法。
導引		0.0.25	conduction exercise	promotion of health and prevention of disease by specially designed physical movements and breathing exercises, also called guiding and conducting exercise	特別に考案された動作と呼吸法による健康増進および疾患予防法。guiding and conducting exerciseとも呼ばれる。

検索表示（原表示の複数語を分離）	IST原表示	Code	Term	Definition/Description	定義/記述
考證學派		0.0.26	classicist school	a Kampo school which bases its study on ancient philological theories in classic textbooks	漢方の一流派で、古典教本を文献学的に研究する。
後世派		0.0.27	Gosei school	a Kampo school in Japan, mainly relies on the five-phase and meridian theory, the same as the latter-day school	日本の漢方の一流派で、主に五行学説と経絡学説を基礎とする。latter-day schoolと同義。
古方派		0.0.28	Koho school	a Kampo school advocating the practical type of medicine of the Shanghanlun, emphasizes abdominal signs and formula-pattern coupling, the same as the antiquity school	漢方の一流派で、傷寒論の実践的な医学を支持し、腹部の徴候とそれに合わせた薬の処方を重視する。antiquity schoolと同義。
折衷派		0.0.29	Sechu school	a Kampo school which blends traditions from the Koho and Gosei schools, the same as the eclectic school	漢方の一流派で、古方派と後世派それぞれの長所を取り入れている。eclectic schoolと同義。
李朱醫學		0.0.30	Li-Zhu medicine	the medicine of Li Dongyuan and Zhu Danxi	李東垣と朱丹渓の医学。
體質; 稟賦		0.0.31	constitution	the characteristics of an individual, including structural and functional characteristics, temperament, adaptability to environmental changes and susceptibility to disease. It is relatively stable, being in part, genetically determined and in part, acquired	構造的・機能的特徴、気質、環境の変化に対する適応能力、疾患感受性を含む個人の特性。相対的に安定しており、一部は先天的、また一部は後天的。
體質醫學		0.0.32	constitutional medicine	the branch of medicine based on assessment of an individual's constitution involving physiology, pathology, diagnosis, treatment and maintenance of health	医学の一部門で、生理的機能、病状、診断、治療および健康維持に関連する個人の体質の評価に基づく。
四象醫學		0.0.33	Four-constitution Medicine	the branch of traditional Korean medicine initiated by Lee Je-ma, which stresses the theory of the four constitutions: physiology, pathology, diagnosis, and maintenance of health, also called Sasang Constitutional Medicine	李済馬によって創設された韓医学の一部門で、四象（生理機能、病状、診断、健康維持）に重きを置く。Sasang Constitutional Medicineとも呼ばれる。
四象人		0.0.34	four constitution types	four types of constitution: a greater yang person: a lesser yang person: a greater yin person: and a lesser yin person	四象体質：太陽人、少陽人、太陰人、少陰人。
太陽人		0.0.35	greater yang person	one who has strong lung and weak liver in the context of Four-Constitution Medicine, also called a Tai-yang person	四象医学では、肺が強く、肝臓が弱いとされる。Tai-yang personとも呼ばれる。
少陽人		0.0.36	lesser yang person	one who has strong spleen and weak kidney in the context of Four-Constitution Medicine, also called a So-yang person	四象医学では、脾臓が強く、腎機能が低いとされる。So-yang personとも呼ばれる。
太陰人		0.0.37	greater yin person	one who has strong liver and weak lung in the context of Four-Constitution Medicine, also called a Tai-eum person	四象医学では、肝臓が強く、肺が弱いとされる。Tai-eum personとも呼ばれる。
少陰人		0.0.38	lesser yin person	one who has strong kidney and weak spleen in the context of Four-Constitution Medicine, also called a So-eum person	四象医学では、腎臓が強く、脾臓が弱いとされる。So-eum personとも呼ばれる。
基礎理論		**1.0.0**	**BASIC THEORIES**		
精氣學說, 陰陽學說, 五行學說		**1.1.0**	**Essential Qi Theory, Yin-yang Theory, and Five Phase Theory**		
天人相應		1.1.1	correspondence between nature and human	one of the basic concepts in traditional Chinese medicine which emphasizes that humans are in adaptive conformity with the natural environment	中医学の基本概念の1つで、ヒトは自然環境と適応協調することを強調している。
整體觀念		1.1.2	holism	one of the philosophical ideas regarding the human body as an organic whole, which is integrated with the external environment	哲学的概念の1つで、人体は有機的統一体であり、外部環境と一体化しているとする。
辨證論治		1.1.3	pattern identification/syndrome differentiation and treatment	diagnosis of the pattern/syndrome, through comprehensive analysis of symptoms and signs, which has implications for determining the cause, nature and location of the illness and the patient's physical condition, and their treatment	症状および徴候の総合的分析による証の診断。疾患の原因、性質、部位や患者の身体状態の判定、さらには治療法の決定に影響を及ぼす。
精氣學說		1.1.4	essential qi theory	one of the basic theories in traditional Chinese medicine about qi, the essential part of which constitutes the body and maintain the activities of life, visceral function and metabolism	気に関する中医学の基礎理論の1つ。気の主要部分が体を構成し、生命活動や内臓機能、代謝を維持する。
陰陽學說		1.1.5	yin-yang theory	an ancient Chinese philosophical concept, dealing with two opposite aspects of matters in nature which are interrelated with each other. Its principle is widely applied to traditional Chinese medicine	古代中国の哲学的概念の1つで、相互に関連する自然界の事物に存在する2つの相反する側面（陰陽）を取り上げたもの。この原理は中医学に幅広く応用されている。
陰陽		1.1.6	yin and yang	the general descriptive terms for the two opposite, complementary and inter-related cosmic forces found in all matter in nature. The ceaseless motion of both yin and yang gives rise to all changes seen in the world	自然界のすべての事物に認められる、2つの対立的、相補的、相関的な宇宙の力を表す一般的な記述用語。陰陽両方の絶え間ない動きが、世界中のあらゆる変化を引き起こしている。
陰		1.1.7	yin	In Chinese philosophy, the feminine, latent and passive principle (characterized by dark, cold, wetness, passivity, disintegration, etc.) of the two opposing cosmic forces into which creative energy divides and whose fusion in physical matter brings the phenomenal world into being	中国哲学においては、創造的エネルギーが分かれて2つの相反する宇宙の力が生じ、これらが物質において融合すると現象界が生み出されるとされる。この2つの相反する力のうち、陰とは女性的、潜在的、受動的な要素（暗、寒、湿潤、受動性、分解などを特徴とする）をいう。
陽		1.1.8	yang	In Chinese philosophy, the masculine, active and positive principle (characterized by light, warmth, dryness, activity, etc.) of the two opposing cosmic forces into which creative energy divides and whose fusion in physical matter brings the phenomenal world into being	中国哲学においては、創造的エネルギーが分かれて2つの相反する宇宙の力が生じ、これらが物質において融合すると現象界が生み出されるとされる。この2つの相反する力のうち、陽とは男性的、活動的、肯定的な要素（明、暖、乾燥、活動性などを特徴とする）をいう。
陰中之陽		1.1.9	yang within yin	the yang aspect of the yin category, for example, the night is regarded as yin in relation to daytime, the period between midnight and dawn is the yang part within yin	陰の範疇における陽の側面。たとえば、夜は昼との関係からは陰とされるが、真夜中から夜明けまでの時間は陰中の陽である。
陰中之陰		1.1.10	yin within yin	the yin aspect of the yin category, for example, the night is regarded as yin in relation to daytime, the period from nightfall to midnight is the yin part within yin	陰の範疇における陰の側面。たとえば、夜は昼との関係から陰とされ、日没から真夜中までの時間は陰中の陰である。
陽中之陽		1.1.11	yang within yang	the yang aspect of the yang category, for example, the daytime is regarded as yang in relation to night, and the period between dawn and noon is the yang part within yang	陽の範疇における陽の側面。たとえば、昼は夜との関係から陽とされ、夜明けから正午までの時間は陽中の陽である。
陽中之陰		1.1.12	yin within yang	the yin aspect of the yang category, for example, the daytime is regarded as yang in relation to night, and the period between midday and nightfall is the yin part of yang	陽の範疇における陰の側面。たとえば、昼は夜との関係からは陽とされるが、正午から日没までの時間は陽中の陰である。

検索表示（原表示の複数語を分離）	IST原表示	Code	Term	Definition/Description	定義/記述
陰陽對立		1.1.13	opposition of yin and yang	the mutually opposing, repelling and contending relationship between yin and yang	陰と陽が相互に対立、反発、競争する関係。
陰陽互根		1.1.14	mutual rooting of yin and yang	the mutually dependent relationship between yin and yang, the same as interdependence between yin and yang	陰と陽が相互に依存する関係。陰陽間の相互依存と同義。
陰陽消長		1.1.15	waxing and waning of yin and yang	alternation of strength and prevalence between the paired yin and yang, the same as natural flux of yin and yang or inter-consuming-supporting relationship of yin and yang	対をなす陰陽間の力と勢いの変化。陰陽の自然な流れまたは陰陽の相克 - 相生関係と同義。
陰陽平衡		1.1.16	yin-yang balance	the state in which yin and yang are balanced	陰と陽が平衡を保っている状態。
陰陽調和		1.1.17	yin-yang harmony	the state in which yin and yang are in harmonious coordination	陰と陽がスムーズに協調している状態。
陰陽轉化		1.1.18	yin-yang conversion	the property of the same thing can be transformed between yin and yang, also called inter-transformation of yin and yang	同一事物の性質が陰と陽の間で転換しうること。陰陽の相互転換とも呼ばれる。
陰極似陽		1.1.19	extreme yin resembling yang	a pathological change in which yang qi is extremely weakened while yin is exuberant in the interior, forcing the yang qi to float on the surface, forming a true cold and false heat pattern/syndrome	陽の気が極端に弱まり、内部に陰が満ちるような病的な変化のこと。陽の気は表面に追いやられ、真寒証と偽熱証が出現する。
陽極似陰		1.1.20	extreme yang resembling yin	a pathological change in which extremely exuberant pathogenic heat makes yang qi depressed and deeply hidden in the interior, with yin restricted to the outside, giving rise to a true heat and false cold pattern/syndrome	病原となる熱が極度に満ちあふれ、陽の気を衰えさせて内部深くに隠してしまうような病的な変化のこと。陰は外側に限定され、真熱証と偽寒証が出現する。
五行學説		1.1.21	five phase theory	one of the philosophical theories of medical practice in ancient China, concerning the composition and evolution of the physical universe, epitomized by the nature and the inhibition-generation relationships of the five phases, wood, fire, earth, metal and water, serving as the guiding ideology and methodology of physiology, pathology, clinical diagnosis and treatment, also known as five elements theory	古代中国における医療の哲学理論の1つ。自然界に代表される物理的宇宙の構成と進化、さらには五行（木、火、土、金、水）の相克 - 相生関係に関する理論で、生理学、病理学、臨床診断、治療を導くイデオロギーや方法となる。five elements theoryとしても知られる。
五行		1.1.22	five phases	the five phases: wood, fire, earth, metal and water, and their movements and changes, also known as five elements	五行（木、火、土、金、水）と、これらの動きや変化。五要素ともいう。
木		1.1.23	wood	one of the five phases, with which the season spring, the color blue or green, the taste sourness, and the liver and gallbladder in the body are associated	五行の1つで、春季、青色または緑色、酸味、肝臓および胆嚢が属する。
火		1.1.24	fire	one of the five phases, with which the season summer, the color red, the taste bitterness, and the heart and small intestine in the body are associated	五行の1つで、夏季、赤色、苦味、心臓および小腸が属する。
土		1.1.25	earth	one of the five phases, with which the season of late summer, the color yellow, the taste sweetness, and the spleen and stomach in the body are associated	五行の1つで、晩夏、黄色、甘味、脾臓および胃が属する。
金		1.1.26	metal	one of the five phases, with which the season autumn, the color white, the taste acridity-pungent, and the lung and large intestine in the body are associated	五行の1つで、秋季、白色、辛味・刺激味、肺および大腸が属する。
水		1.1.27	water	(1) one of the five phases, with which the season winter, the color black, the taste saltiness, and the kidney and bladder in the body are associated; (2) pathologic aspect of body fluid	（1）五行の1つで、冬季、黒色、塩味、腎臓および膀胱が属する、（2）体液の病的な状態。
五行歸類		1.1.28	categorization according to the five phases	classification of material things and phenomena into five categories by comparing their structures, properties and actions with the five phases	物質や現象の構造、性質、作用を五行と比較することによって、それらを5つのカテゴリーに分類すること。
相生	(相)生	1.1.29	engendering	the relationship in which each phase and its associated phenomena give rise to or promote another sequential phase, also the same as generating	各行とそれに関連する現象が次の行を発生させる、あるいは促進する関係。generatingと同義。
生	(相)生	1.1.29	engendering	the relationship in which each phase and its associated phenomena give rise to or promote another sequential phase, also the same as generating	各行とそれに関連する現象が次の行を発生させる、あるいは促進する関係。generatingと同義。
木生火		1.1.30	wood engenders fire	the category of wood generates or promotes the category of fire, also the same as wood generating fire	木は火を生じさせる。wood generating fireと同義。
火生土		1.1.31	fire engenders earth	the category of fire generates or promotes the category of earth, also called fire generating earth	火は土を生じさせる。fire generating earthと同義。
土生金		1.1.32	earth engenders metal	the category of earth generates or promotes the category of metal, also called earth generating metal	土は金を生じさせる。earth generating metalと同義。
金生水		1.1.33	metal engenders water	the category of metal generates or promotes the category of water, also called metal generating water	金は水を生じさせる。metal generating waterと同義。
水生木		1.1.34	water engenders wood	the category of water generates or promotes the category of wood, also called water generating wood	水は木を生じさせる。water generating woodと同意
相克	(相)克	1.1.35	restraining	the relationship in which each phase and its associated phenomena restrict/check/control another phase	各行とそれに関連する現象が別の行を制限／阻止／抑制する関係。
克	(相)克	1.1.35	restraining	the relationship in which each phase and its associated phenomena restrict/check/control another phase	各行とそれに関連する現象が別の行を制限／阻止／抑制する関係。
木克土		1.1.36	wood restrains earth	the category of wood restricts or checks the category of earth, also called wood controlling earth	木は土を制限または阻止する。wood controlling earthと同義。
火克金		1.1.37	fire restrains metal	the category of fire restricts or checks the category of metal, also called fire controlling metal	火は金を制限または阻止する。fire controlling metalと同義。
土克水		1.1.38	earth restrains water	the category of earth restricts or checks the category of water, also called earth controlling water	土は水を制限または阻止する。earth controlling waterと同義。
水克火		1.1.39	water restrains fire	the category of water restricts or checks the category of fire, also called water controlling fire	水は火を制限または阻止する。water controlling fireと同義。
金克木		1.1.40	metal restrains wood	the category of metal restricts or checks the category of wood, also called metal controlling wood	金は火を制限または阻止する。metal controlling woodと同義。
相乘	(相)乘	1.1.41	overwhelming	abnormally severe restraining of the five phases in the same sequence as normal restraining, also known as over-acting	正常な相克と同じ順序での、五行の過剰な相克。over-actingとしても知られる。
乘	(相)乘	1.1.41	overwhelming	abnormally severe restraining of the five phases in the same sequence as normal restraining, also known as over-acting	正常な相克と同じ順序での、五行の過剰な相克。over-actingとしても知られる。
相侮	(相)侮	1.1.42	rebellion	restraining opposite to that of the normal restraining sequence of the five phases, also known as insulting	正常な相克と逆の順序での相克。insultingとしても知られる。

検索表示（原表示の複数語を分離）	IST原表示	Code	Term	Definition/Description	定義/記述
侮	(相)侮	1.1.42	rebellion	restraining opposite to that of the normal restraining sequence of the five phases, also known as insulting	正常な相克と逆の順序での相克。insultingとしても知られる。
五常		1.1.43	five constants	a collective term referring to wood, fire, earth, metal and water in normal movement	正常に動く木、陽、土、金、水の総称。
制化		1.1.44	inhibition and generation	the engendering and restraining relationships for maintaining a relative balance and normal coordination in the five phase theory	五行学説において、相対的バランスと正常な協調を維持するための相生相克関係。
亢害承制		1.1.45	harmful hyperactivity and responding inhibition	one of the principles of the five phase theory indicating that hyperactivity of any phase is harmful, and its restriction will restore the normal balance	五行学説の原則の1つで、いずれの行の過剰も有害であり、その抑制により正常なバランスの回復を図るとする。
母氣		1.1.46	mother qi	qi of the viscus that engenders in the engendering sequential relationship of the five phases	五行の相生関係において、行を生じさせる内臓の気。
子氣		1.1.47	child qi	qi of the viscus that is engendered in the engendering sequential relationship of the five phases	五行の相生関係において、行が生じる内臓の気。
母子相及		1.1.48	mother and child affecting each other	influence of one phase exerted on the phase that it engenders or that engenders it sequentially	1つの行と、その行から生じた別の行またはさらにそれから派生する行との元の行が及ぼしあう影響
精, 神, 氣, 血, 津液		**1.2.0**	**Essence, Spirit, Qi, Blood, Fluid and Humor**		
精		1.2.1	essence	(1) the fundamental substance that builds up the physical structure and maintains body function; (2) reproductive essence stored in the kidney	(1) 身体構造を構築し、身体機能を維持するための基本的物質、(2) 生殖の精は腎臓に貯蔵される。
先天之精		1.2.2	innate essence	the original substance responsible for construction of the body and generation of offspring, often referring to the reproductive essence, also called prenatal essence	身体の形成と子孫の産出をつかさどる、生まれつき備わった物質。生殖の精と称されることもある。prenatal essenceとも呼ばれる。
後天之精		1.2.3	acquired essence	the essential substance acquired from the food after digestion and absorption, and used to maintain the vital activities and metabolism of the body, the same as postnatal essence	食物に由来し、消化・吸収後に得られる必須物質で、生命活動の維持および身体の代謝に使われる。postnatal essenceと同義。
腎精		1.2.4	kidney essence	the original essence stored in the kidney	生まれつき備わった、腎臓に貯蔵される精。
神		1.2.5	mind	mental activities, referring to mentality, consciousness, thinking and feeling	知性、意識、思考および感情に関する精神活動。
神		1.2.6	spirit	spiritual activities	精神的活動。
神		1.2.7	vitality	manifestations of vital functioning	生命機能の現れ。
精神		1.2.8	essence-spirit	state of mind or mood, reflection of the strength of essence, also called spirit or mind	心または気持ちの状態で、精の強さを反映する。spiritまたはmindとも呼ばれる。
魂		1.2.9	ethereal soul	the moral and spiritual part of the human being	人の感情的・精神的部分。
魄		1.2.10	corporeal soul	the animating part of one's mind	身体を活気づける心の部分。
意		1.2.11	ideation	act or power of thinking and forming ideas	思考およびアイデア形成の行為または能力。
志		1.2.12	will	mental power by which a person can direct his thoughts and actions	考えおよび行動を方向付けるための精神力。
命門		1.2.13	life gate	(1) the place where qi transformation of the human body originates, serving as the root of life; (2) right kidney; (3) acupuncture point (GV4)	(1) 生命の根源であり、人体の気の転換が起こる場所、(2) 右腎、(3) 経穴（GV4）。
命門之火	命門之火;先天之火	1.2.14	life gate fire	innate fire from the life gate, a synonym of kidney yang	命門に由来する生まれつき備わった火。腎陽と同義。
先天之火	命門之火;先天之火	1.2.14	life gate fire	innate fire from the life gate, a synonym of kidney yang	命門に由来する生まれつき備わった火。腎陽と同義。
君火		1.2.15	sovereign fire	another name for heart fire, in contrast to the ministerial fire	心火の別名。相火と対象をなす。
相火		1.2.16	ministerial fire	a kind of physiological fire originating in the kidney and attached to the liver, gallbladder and triple energizer, which, in cooperation with the sovereign fire from the heart, warms the viscera and promotes their activities. If this fire is hyperactive, it is also harmful to the body	腎臓に由来する生理的な火の一種で、肝臓、胆嚢、および三焦に結びつき、心臓に由来する君火と共に内臓を温め、活動を促す。相火が過剰になると、身体に有害となる。
氣		1.2.17	qi	the basic element that constitutes the cosmos and, through its movements, changes and transformations, produces everything in the world, including the human body and life activities. In the field of medicine, qi refers both to the refined nutritive substance that flows within the human body as well as to its functional activities	宇宙を構成する基本的要素。その動き、変化および転換を通して、人体および生命活動を含む世界の万物を作り出す。医学において、気は人体内を流れる純粋な栄養物質と、その機能活性双方を指す。
先天之氣		1.2.18	innate qi	the qi that exists from birth and is stored in the kidney, also the same as prenatal qi	生まれつき備わった、腎臓に貯蔵される気。prenatal qiと同義。
後天之氣		1.2.19	acquired qi	the qi that is acquired after birth and is formed from the food in combination with the fresh air inhaled in the lung, also the same as post-natal qi	誕生後に得られる気で、食物と肺に吸い込まれる新鮮な空気によって形成される。post-natal qiと同義。
正氣		1.2.20	healthy qi	a collective designation for all normal functions of the human body and the abilities to maintain health, including the abilities of self-regulation, adaptation to the environments, resistance against pathogens and self-recovery from illness, the same as normal/genuine qi	人体のすべての正常機能と、自己調整力、環境への適応力、病原体への抵抗力、病気からの自己回復力を含む健康維持能力の総称。正常気／真気と同義。
眞氣		1.2.21	genuine qi	the combination of the innate qi and the acquired qi, serving as the physical substrata and dynamic force of all vital functions, also known as true qi	先天の気と後天の気からなるもので、すべての生命機能の物理的基盤および原動力。正気としても知られる。
原氣	原氣;元氣	1.2.22	source qi	the combination of the innate qi and the acquired qi, serving as the most fundamental qi of the human body; the same as original/primordial qi	先天の気と後天の気からなるもので、人体の最も基本的な気。元気／primordial qiと同義。
元氣	原氣;元氣	1.2.22	source qi	the combination of the innate qi and the acquired qi, serving as the most fundamental qi of the human body; the same as original/primordial qi	先天の気と後天の気からなるもので、人体の最も基本的な気。原気／primordial qiと同義。

検索表示（原表示の複数語を分離）	IST原表示	Code	Term	Definition/Description	定義/記述
宗氣		1.2.23	ancestral qi	the combination of the essential qi derived from food with the air inhaled, stored in the chest, and serving as the dynamic force of blood circulation, respiration, voice, and bodily movements, the same as pectoral qi	食物に由来する精気と吸い込まれた空気からなるもので、胸中に貯蔵される。血流、呼吸、発声および身体の動きの原動力。胸の気と同義。
衛氣		1.2.24	defense qi	the qi that moves outside the vessels, protecting the body surface and warding off external pathogens, the same as defensive qi	脈外を循る気で、体表を保護し、病原体の侵入を防ぐ。defensive qiと同義。
營氣		1.2.25	nutrient qi	the qi that moves within the vessels and nourishes all the organs and tissues, the same as nutritive qi	脈内を脈外を循る気で、すべての器官および組織に栄養分を与える。栄気と同義。
津氣		1.2.26	fluid qi	(1) the same as fluid; (2) the qi carried by fluid	(1) 津と同義、(2) 津により運ばれる氣。
臟氣		1.2.27	visceral qi	(1) the qi that enables the viscera to perform their activities; (2) the functional activities of viscera	(1) 臟の活動を支える気、(2) 臟の機能的活動。
腑氣		1.2.28	bowel qi	(1) the qi that enables the bowels to perform their activities; (2) the functional activities of bowels	(1) 腑の活動を支える気、(2) 腑の機能的活動。
心氣		1.2.29	heart qi	essential qi of the heart, the physical substrata and dynamic force of the functional activities of the heart	心臓の精気で、心臓の機能的活動の物理的基盤および原動力。
肝氣		1.2.30	liver qi	essential qi of the liver, the physical substrata and dynamic force of the functional activities of the liver	肝臓の精気で、肝臓の機能的活動の物理的基盤および原動力。
脾氣		1.2.31	spleen qi	essential qi of the spleen, the physical substrata and dynamic force of the functional activities of the spleen	脾臓の精気で、脾臓の機能的活動の物理的基盤および原動力。
肺氣		1.2.32	lung qi	essential qi of the lung, the physical substrata and dynamic force of the functional activities of the lung	肺の精気で、肺の機能的活動の物理的基盤および原動力。
腎氣		1.2.33	kidney qi	essential qi of the kidney, the physical substrata and dynamic force of the functional activities of the kidney	腎臓の精気で、腎臓の機能的活動の物理的基盤および原動力。
腎間動氣		1.2.34	stirring qi of the kidney region	that part of genuine qi stored between the kidneys, as the motive force necessary for all the activities of the body, also known as motive force of the kidney region	真気の一部で、左右の腎臓の間に、身体のあらゆる活動に必要な原動力として左右の腎臓の間に貯蔵される。motive force of the kidney regionとしても知られる。
膽氣		1.2.35	gallbladder qi	essential qi of the gallbladder, the physical substrata and dynamic force of the functional activities of the gallbladder	胆嚢の精気で、胆嚢の機能的活動の物理的基盤および原動力。
胃氣		1.2.36	stomach qi	essential qi of the stomach, the physical substrata and dynamic force of the functional activities of the stomach. Also used to denote a state of basic vitality detected by examination of the radial pulse	胃の精気で、胃の機能的活動の物理的基盤および原動力。また、橈骨動脈拍動の脈診で確認される基礎活力の状態を指す。
中氣		1.2.37	middle qi	qi of the middle energizer, the physical substrata and dynamic force of the functional activities of the spleen, stomach and small intestine, including digestion, absorption, transportation, upbearing of the clear and downbearing of the turbid	中焦の気で、消化、吸収、運搬、昇清および降濁を含む脾臓、胃、および小腸の機能的活動の物理的基盤および原動力。
經氣	經氣; 經絡之氣	1.2.38	meridian qi	the qi that flows through the meridians, the same as collateral qi	経絡を通して流れる気。経脈気と同義。
經絡之氣	經氣; 經絡之氣	1.2.38	meridian qi	the qi that flows through the meridians, the same as collateral qi	経絡を通して流れる気。経脈気と同義。
陰氣		1.2.39	yin qi	the yin aspect of qi, particularly referring to that aspect of qi as physical substrata	気の陰の側面。特に物理的基盤としての側面を指す。
陽氣		1.2.40	yang qi	the yang aspect of qi, particularly referring to that aspect of qi as functional activities	気の陽の側面。特に機能的活動の側面を指す。
氣化		1.2.41	qi transformation	a general term referring to various changes through the activity of qi, namely the metabolism and mutual transformation between essence, qi, blood and fluids	気の活動を通したさまざまな変化、すなわち代謝および精気、気、血、津間での相互変換の一般的な用語。
氣機		1.2.42	qi movement	movement of qi, including ascending, descending, exiting and entering as its basic forms, also known as qi dynamic/qi mechanism	基本的な気の動き（昇、降、 出、入）。qi dynamic／qi mechanismとしても知られる。
升降出入		1.2.43	upward, downward, inward and outward movement	the basic forms of qi movement, that is, ascending, descending, exiting and entering	基本的な気の動き（昇、降、 出、入）。
血		1.2.44	blood	the red fluid circulating through the blood vessels, and nourishing and moistening the whole body	血管を通して循環する赤い液体。全身に栄養と水分を供給する。
營血		1.2.45	nutrient and blood	a collective term for nutrient and blood	栄養と血の総称
津		1.2.46	fluid	the liquid substance that circulates with qi and blood, also known as thin fluid	気と血と共に循環する液体。thin fluidとしても知られる。
液		1.2.47	humor	the thick fluid stored in body cavities such as bowels, viscera, articular and cranial cavities, also known as thick fluid	腸、内臓、関節腔、頭蓋腔などの身体の空洞部に貯蔵される濃い液体。thick fluidとしても知られる。
津液		1.2.48	fluid and humor	a general term for all kinds of normal fluid in the body, except the blood, also known as body fluids	血を除く体内のすべての正常な液体の総称。体液としても知られる。
津血同源		1.2.49	homogeny of fluid and blood	the physiological phenomenon that body fluid and blood share a common source, the essential qi of food	体液と血が共通の源（食物に由来する精気）に由来する生理的現象。
精血同源		1.2.50	homogeny of essence and blood	the physiological phenomenon that essence and blood share a common source, the essential qi of food	精と血が共通の源（食物に由来する精気）に由来する生理的現象。
汗		1.2.51	sweat	the fluid that exudes from sweat glands; the humor of the heart	汗腺からにじみ出る液体（心臓の液）。
淚		1.2.52	tears	the fluid secreted by the lacriminal glands; the humor of the liver	涙腺から分泌される液体（肝臓の液）。
涎		1.2.53	drool	thinner saliva; the humor of the spleen	より薄い唾液（脾臓の液）。
涕		1.2.54	snivel	the fluid that exudes from the nose; the humor of the lung	鼻からにじみ出る液体（肺の液）。
唾		1.2.55	spittle	thicker saliva; the humor of the kidney	より濃い唾液（腎臓の液）。
營衛		1.2.56	nutrient and defense	a collective term for nutrient qi and defense qi	営気と衛気の総称。

検索表示（原表示の複数語を分離）	IST原表示	Code	Term	Definition/Description	定義/記述
氣血水		1.2.57	qi, blood and water	the three vital substances of the human body, any of which, when overwhelmed by toxin, causes disease	生命維持に必要な3要素。いずれかが毒気に当てられると、疾患を引き起こす。
水穀		1.2.58	water and food	a term referring to food and drink; diet	食物、飲料、食事を指す。
臟腑		**1.3.0**	**Viscera and Bowels**		
臟象		1.3.1	visceral manifestation	the outward manifestation of internal organs through which physiological functions as well as pathological changes can be detected and the state of health appraised	内臓の外観上の症状発現。生理的機能および病的変化を検知し、健康状態を評価できる。
臟象學說		1.3.2	visceral manifestation theory	the theory dealing with the study of physiological functions and pathological changes of the internal organs as well as their interrelationship and external manifestation	内臓の生理的機能および病的変化と、それらの相互関係および外観上の症状発現を解き明かす理論。
臟		1.3.3	viscus	an internal organ where essence and qi are formed and stored, viscera in plural	精および気が形成、貯蔵される内臓。複数形はviscera。
腑		1.3.4	bowel	an internal organ where food is received, transported and digested	食物を受け取り、運び、消化する内臓。
臟腑		1.3.5	viscera and bowels	a collective term for internal organs, also called zang-organs and fu-organs	内臓の総称。zang organs、fu-organsとも呼ばれる。
五臟		1.3.6	five viscera	a collective term for the heart, liver, spleen, lung and kidney	心臓、肝臓、脾臓、肺、および腎臓の総称。
六腑		1.3.7	six bowels	a collective term for the gallbladder, stomach, large intestine, small intestine, urinary bladder and triple energizers	胆嚢、胃、大腸、小腸、膀胱、および三焦の総称。
陽臟		1.3.8	yang viscus	viscera of yang nature, referring to the heart and the liver	陽の内臓。心臓と肝臓を指す。
陰臟		1.3.9	yin viscus	viscera of yin nature, referring to the spleen, the lung and the kidney	陰の内臓。脾臓、肺、および腎臓を指す。
心		1.3.10	heart	the organ located in the thoracic cavity above the diaphragm, which controls blood circulation and mental activities	横隔膜上部の胸腔に位置する臓器。血流と精神活動を制御する。
肝		1.3.11	liver	the organ located in the right hypochondrium below the diaphragm, which stores blood, facilitates the coursing of qi, and is closely related to the function of the sinews and eyes	横隔膜下部の右季肋部に位置する臓器。血を貯蔵し、気の流れを促すとともに、腱および眼の機能と密接に関わる。
脾		1.3.12	spleen	the organ located in the middle energizer below the diaphragm, whose main function is to transport and transform food, upbear the clear substances, keep the blood flowing within the vessels, and is closely related to the limbs and flesh	横隔膜下部の中焦に位置する臓器。主に食物の運化、精微な物質の昇精、血管内での血流維持をつかさどり、四肢および肌肉と密接に関わる。
肺		1.3.13	lung	a pair of organs located in the thoracic cavity above the diaphragm, which control respiration, dominate qi, govern diffusion and depurative downbearing, regulate the waterways, and are closely related to the function of the nose and skin surface	横隔膜上部の胸腔に位置する対になった臓器。呼吸の制御、気の支配、宣発・粛降、水道の調整をつかさどり、鼻および皮膚表面の機能と密接に関わる。
腎		1.3.14	kidney	a pair of organs located in the lumbar region, which store vital essence, promote growth, development, reproduction, and urinary function, and also have a direct effect on the condition of the bone and marrow, activities of the brain, hearing and inspiratory function of the respiratory system	腰部に位置する対になった臓器。生命維持に必要な精の貯蔵、発育、成長、生殖および泌尿器機能の促進をつかさどり、骨と髄、脳の活動、聴力、および呼吸器系の吸気機能に直接的な影響を及ぼす。
心包	心包;<br>心包絡	1.3.15	pericardium	the outer covering of the heart, including the pericardium collateral	心臓を包み込む嚢。心膜を含む。
心包絡	心包;<br>心包絡	1.3.15	pericardium	the outer covering of the heart, including the pericardium collateral	心臓を包み込む嚢。心膜を含む。
膽		1.3.16	gallbladder	one of the six bowels, which, connecting with the liver, stores and discharges bile	六腑の1つで、肝臓につながっており、胆汁を貯蔵および排出する。
胃		1.3.17	stomach	one of the six bowels, whose main function is to receive and initiate food digestion	六腑の1つで、主な機能は食物の受け入れと消化の開始。
小腸		1.3.18	small intestine	one of the six bowels, whose main function is to receive food content of the stomach, further digest it and absorb nutrients and water	六腑の1つで、主な機能は胃から送られてくる食物の受け入れ、さらなる消化、および栄養素と水分の吸収。
大腸		1.3.19	large intestine	one of the six bowels, which receives waste passed down from the small intestine and then forms it into stool before discharging it from the body	六腑の1つで、小腸から送られてくる残渣を受け取り、体外へ排出するための糞便を形成する。
膀胱	膀胱;<br>胞	1.3.20	bladder	one of the six bowels, which stores and discharges urine	六腑の1つで、尿を貯蔵、排出する。
胞	膀胱;<br>胞	1.3.20	bladder	one of the six bowels, which stores and discharges urine	六腑の1つで、尿を貯蔵、排出する。
三焦		1.3.21	triple energizers	a collective term for the three portions of the body cavity, through which the visceral qi is transformed, also widely known as triple burners	臓気が転換される、体腔の3部分の総称。triple burnersとしても広く知られる。
上焦		1.3.22	upper energizer	the chest cavity, i.e., the portion above the diaphragm housing the heart and lung, also known as upper burner	胸腔、すなわち横隔膜上部の心臓と肺がある部分。upper burnerとしても知られる。
中焦		1.3.23	middle energizer	the upper abdominal cavity, i.e., the portion between the diaphragm and the umbilicus housing the spleen, stomach, liver and gallbladder, also known as middle burner	上腹腔、すなわち横隔膜とへその間の脾臓、胃、肝臓および胆嚢がある部分。middle burnerとしても知られる。
下焦		1.3.24	lower energizer	the lower abdominal cavity, i.e., the portion below the umbilicus housing the kidneys, bladder, small and large intestines, also known as lower burner	下腹腔、すなわちへそ下部の腎臓、膀胱、小腸、大腸がある部分。lower burnerとしても知られる。
奇恒之腑		1.3.25	extraordinary organs	a collective term for the brain, marrow, bones, blood vessels, gallbladder and uterus. They are called extraordinary because their morphological and physiological properties are different from the ordinary bowels and viscera	脳、髄、骨、脈（血管）、胆嚢、および子宮の総称。生理的特性が五臓六腑とは異なる。
腦		1.3.26	brain	one of the extraordinary organs enclosed within the skull where the marrow converges, and the spirit, mental activities as well as thinking take place	奇恒の腑の1つで、頭蓋内に位置し、髄が集合し、心が宿り、精神活動および思考が起こる臓器。
元神之府		1.3.27	house of the original spirit	another name for the brain, being the source of mental activities	脳の別名。精神活動の源。
髓海		1.3.28	sea of marrow	another name for the brain, being where the marrow assembles	脳の別名。髄が集合する。

検索表示（原表示の複数語を分離）	IST原表示	Code	Term	Definition/Description	定義/記述
髓		1.3.29	marrow	an extraordinary organ including bone marrow and spinal marrow, both of which are nourished by the kidney essence	奇恒の腑の1つで、骨髄、脊髄（いずれも腎精より栄養を得る）を含む。
骨		1.3.30	bone	one of the extraordinary organs that forms the framework of the body, protects the internal organs and facilitates movement	奇恒の腑の1つで、身体の枠組みを形成し、内臓を保護し、動きを容易にする。
脈		1.3.31	vessel	the conduit through which qi and blood pass	気および血液の通り道。
胞		1.3.32	placenta	one of the extraordinary organs lining the uterus during pregnancy by which the fetus is nourished, and from which it is expelled at birth	奇恒の腑の1つ（胎盤）で、妊娠中に胎児を育む子宮内に形成される。出産時に排出される。
胞	胞; 胞宮; 女子胞	1.3.33	uterus	(in women) organ in which offspring is carried and nourished while it develops before birth	出産前の発達中の子を宿し、育む（女性の）臓器。
胞宮	胞; 胞宮; 女子胞	1.3.33	uterus	(in women) organ in which offspring is carried and nourished while it develops before birth	出産前の発達中の子を宿し、育む（女性の）臓器。
女子胞	胞; 胞宮; 女子胞	1.3.33	uterus	(in women) organ in which offspring is carried and nourished while it develops before birth	出産前の発達中の子を宿し、育む（女性の）臓器。
血室		1.3.34	blood chamber	another name for the uterus	子宮の別名。
陰道		1.3.35	vagina	genital canal in the female, leading from the uterus to the vulva	子宮から外陰部へ続く管状の女性器。
心血		1.3.36	heart blood	the blood governed by the heart, which flows all over the body, particularly that part of blood serving as the basis for the physiological activities of the heart, including mental activities	心臓によって支配される血液で、全身を流れる。特に心臓を流れる血液は、精神活動を含む心臓の生理的活動の基礎となる。
心陰		1.3.37	heart yin	yin essence of the heart, the opposite of heart yang, the quiescent and moistening aspect of heart function	心臓の陰精で、心陽に相対する。心臓の静的および滋潤作用に関わる。
心陽		1.3.38	heart yang	yang qi of the heart, which stimulates the activities of the heart and mind, and has a warming action	心臓の陽精で、心臓と心の活動を刺激する。温煦作用がある。
肝血		1.3.39	liver blood	the blood that is stored in the liver and nourishes the liver system, including the liver itself, liver meridian, eyes, tendons and nails, as well as the whole body	肝臓に貯蔵される血で、肝臓、肝経、眼、腱、爪を含む肝臓系に栄養を与える。
肝陰		1.3.40	liver yin	the opposite of liver yang, the liver essence-blood and the quiescent and nutritive aspect of liver function, which also inhibits overactivity of liver yang	肝陽に相対する。肝精－血および肝機能の静的および滋養に関する側面。肝陽の過活動を抑制する。
肝陽		1.3.41	liver yang	yang qi of the liver, opposite to liver yin, referring chiefly to the warming, upbearing and coursing-freeing function of the liver	肝臓の陽気で、肝陰に相対する。主に肝臓の温煦、昇発および疏泄機能を指す。
脾陰		1.3.42	spleen yin	yin fluid of the spleen, in opposition to spleen yang, referring to the moistening, nourishing and astringing aspect of the spleen	脾臓の陰津で、脾陽に相対する。脾臓の滋潤、滋養、および収斂機能を指す。
脾陽		1.3.43	spleen yang	the yang aspect of the spleen, which refers to the promotion of spleen functions including transporting, transforming, upbearing and warming actions	脾臓の陽の側面。運化、昇発および温煦作用を含む脾臓機能の促進を指す。
肺陰		1.3.44	lung yin	the opposite of lung yang, the yin fluid that moistens the lung, in coordination with lung qi	肺陽に相対する。陰津は肺気と協調し肺を滋潤する。
肺陽		1.3.45	lung yang	the yang aspect of the lung, referring to the warming, moving, ascending and diffusing functions of the lung	肺の陽の側面。肺の温煦、推動、宣発機能を指す。
腎陰		1.3.46	kidney yin	the yin aspect of the kidney, which has a moistening, nourishing and cooling effect on all organs	腎臓の陰の側面。すべての臓器に対する滋潤、滋養、および冷却効果を有する。
腎陽		1.3.47	kidney yang	the yang aspect of the kidney, which warms and activates all organs	腎臓の陽の側面。すべての臓器を温め、活性化する。
胃陰		1.3.48	stomach yin	the opposite of stomach yang, the fluids of the stomach necessary for maintaining normal food intake and preliminary digestion in coordination with stomach yang	胃陽に相対する。胃津は、胃陽との協調下での正常な食物摂取の維持と予備的消化に必要。
胃陽		1.3.49	stomach yang	the opposite of stomach yin, the yang qi of the stomach, referring to the activity or function of the stomach in food intake and preliminary digestion	胃陰に相対する。胃の陽気で、食物の摂取および予備的消化における胃の活動と機能を指す。
胃津		1.3.50	stomach fluid	fluid of the stomach, the same as stomach yin	胃液。胃陰と同意。
神明		1.3.51	bright spirit	all the human life activities including mind, will, mood and thinking, governed by the heart	心臓がつかさどる、精神、意志、気分および思考を含む人のすべての生命活動。
血脈		1.3.52	blood vessel	the vessels in which blood circulates	血液が流れる脈管。
泌別清濁		1.3.53	separation of the clear and turbid	the small intestine’s function, by which the clear (the food essence and water) is absorbed while the turbid (the waste matter) is passed to the large intestine	小腸の機能。清（水穀の精微）を吸収し、濁（廃棄物）を大腸に送る。
升發		1.3.54	upbearing and effusion	the upward and outward movement of qi, a function governed by the liver, the same as sending the qi upwards, outwards and throughout the body	上方および外側へ向かう気の動き。肝臓がつかさどる機能で、sending the qi upwards, outwards and throughout the body と同義。
剛臟		1.3.55	unyielding viscus	an internal organ that has a tendency to hyperactivity and counterflow of qi, referring to the liver, also known as resolute viscus	活動過剰と気の逆流の傾向がある内臓で、肝臓を指す。resolute viscus としても知られる。
疏泄		1.3.56	free coursing	referring to the function of the liver that ensures the free movement of qi and prevents qi stagnation, also known as soothing	気の自由な動きを確実にし、気滞を防ぐ肝臓の機能。soothing としても知られる。
血海		1.3.57	sea of blood	a term referring to: (1) the thoroughfare vessel; (2) the liver	(1) 主要な血管、(2) 肝臓を指す。
升淸		1.3.58	upbearing the clear	referring to the function of spleen that sends the food essence to the heart and lung, the same as sending the clear upward	水穀の精微を心臓と肺へ送る脾臓の機能を指す。sending the clear upward と同義。
降濁		1.3.59	downbearing the turbid	referring to the function of the stomach that sends the partially digested food down to the intestines, the same as sending the turbid downward	部分消化された食物を腸へ送る胃の機能を指す。sending the turbid downward と同義。

検索表示（原表示の複数語を分離）	IST原表示	Code	Term	Definition/Description	定義/記述
運化		1.3.60	transportation and transformation	the function of the spleen by which the essence is transformed from food and drink, absorbed, and distributed to all parts of the body	脾臓の機能。飲食物から転換された精が吸収され、身体のあらゆる部位に送られる。
生化		1.3.61	engendering transformation	referring to formation of qi and blood from food essence that is closely related to the spleen function, also known as generation and transformation	水穀の精微からの気および血の形成を指し、脾臓の機能と密接に関わる。generation and transformation としても知られる。
嬌臟		1.3.62	delicate viscus	an expression referring to the lung which is the viscus most susceptible to invasion by external pathogens	外部からの病原体侵入に最も弱い臓である肺を指す。
貯痰之器		1.3.63	receptacle that holds phlegm	the organ where phlegm collects, referring to the lung	痰が溜まる臓器。肺を指す。
水之上源		1.3.64	upper source of water	an expression referring to the lung, which is situated in the upper energizer regulating water metabolism	水分代謝を制御する上焦に位置する肺を指す。
呼吸		1.3.65	breathing	drawing air into the lung and expelling it to perform gas exchange, the same as respiration	空気を肺に引き込み、排出することで換気を行う。respiration と同義。
治節		1.3.66	management and regulation	the function of the lung to keep the physiological activity of the whole body in balance	全身の生理作用のバランスを保つ肺の機能。
肅降		1.3.67	purification and down-sending	the downward movement and purifying action of lung qi in contrast to its diffusing action	肺気の降下および浄化作用。宣発作用と相対する。
宣發		1.3.68	diffusion	the upward and outward movement of lung qi, also known as dispersion	肺気の上方および外部に向けた動き。dispersion としても知られる。
水道		1.3.69	waterways	the pathway of water metabolism in the body	体内の水分代謝経路。
通調水道		1.3.70	regulate the waterways	dredge and regulate the pathway of water metabolism	水分代謝経路の浚渫および調節。
天癸		1.3.71	heavenly tenth	that upon which development of the reproductive organs and maintenance of reproductive function depends, derived from the kidney essence when it is abundant, also called (female) sex-stimulating essence/menstruation	生殖器の発達および生殖機能の維持の基盤。腎精の充実とともに成熟する。（女性では）sex-stimulating essence／menstruation とも呼ばれる。
生殖		1.3.72	reproduction	the process by which humans give rise to offspring	人が子孫を産み出すプロセス。
納氣		1.3.73	qi absorption	the action of the kidney in connection with normal respiration. The lung sends the qi down to the kidney, which holds the qi down, and then absorbs it	正常呼吸に関連する腎臓の活動。腎臓は肺から送られてくる気を保持、吸収する。
先天		1.3.74	innate	possessed from birth, relating to the natural endowment, in contrast to acquired after birth, the same as inborn	生まれつき（天賦の才）。後天と相対する。inbornと同義。
先天之本		1.3.75	root of innate endowment	that which is inherited at conception	受胎時に受け継いだもの。
膀胱氣化		1.3.76	bladder qi transformation	the function of the bladder in storing and discharging urine, based on qi transformation of the kidney	尿の貯蔵と排出における膀胱の機能で、腎臓の気の転換に基づく。
心腎相交		1.3.77	heart-kidney interaction	superior-inferior, ascending-descending, water-fire and yin-yang coordination between the heart and kidney	心臓と腎臓間での、優劣、昇降、水火、陰陽調整。
心開竅于舌		1.3.78	heart opens at the tongue	the heart opens into the tongue, reflecting its physiological and pathological conditions	心臓は舌に通じており、舌には心臓の生理的状態および病的状態が現れる。
肺開竅于鼻		1.3.79	lung opens at the nose	the lung opens into the nose, reflecting its physiological and pathological conditions	肺は鼻に通じており、鼻には肺の生理的状態および病的状態が現れる。
脾開竅于口		1.3.80	spleen opens at the mouth	the spleen opens into the mouth, reflecting its physiological and pathological conditions	脾臓は口に通じており、口には脾臓の生理的状態および病的状態が現れる。
肝開竅于目		1.3.81	liver opens at the eyes	the liver opens into the eyes, reflecting its physiological and pathological conditions	肝臓は眼に通じており、眼には肝臓の生理的状態および病的状態が現れる。
腎開竅于耳		1.3.82	kidney opens at the ears	the kidney opens into the ears, reflecting its physiological and pathological conditions	腎臓は耳に通じており、耳には腎臓の生理的状態および病的状態が現れる。
肝腎同源		1.3.83	homogeny of liver and kidney	the liver and the kidney are intimately related, as they nourish and support each other, the blood stored in the liver and the essence stored in the kidney are of the same origin, and the ministerial fire present in both the liver and the kidney is from a common source	肝臓と腎臓は密接な関係にあり、相互に滋養および支援しあう。肝臓に貯蔵された血と腎臓に貯蔵された精の起源は同じであり、また双方の相火は同じ源に由来する。
經絡		**1.4.0**	**Meridian and Collateral**		
經絡		1.4.1	meridian and collateral	a system of conduits through which qi and blood circulate, connecting the bowels, viscera, extremities, superficial organs and tissues, making the body an organic whole, the same as channels and networks; meridians or channels, in short	気と血が流れる道筋。臓腑、四肢、外部器官および組織を結び、身体を有機的統一体とならしめる。channels and networksと同義。略してmeridiansまたはchannelsと称する。
經絡學說		1.4.2	meridian and collateral theory	a theory concerned the meridians and collaterals, the same as channels and networks theory	経絡に関する理論。channels and networks theoryと同義。
經脈		1.4.3	meridian vessel	the main pathways of qi and blood coursing vertically, composed of the twelve regular meridians and the eight extra meridians, the same as channel vessel	気と血の主要な経路で、垂直に走る。十二正経と奇経八脈で構成される。channel vessel と同義。
手三陰經		1.4.4	three yin meridians of the hand	the meridians/channels running through the anterior aspect of the upper limbs from the chest to the hands, namely, the lung meridian, the heart meridian and the pericardium meridian	上肢前面の胸から手へと走る経絡（肺経、心経、心包経）。
手三陽經		1.4.5	three yang meridians of the hand	the meridians/channels running through the posterior aspect of the upper limbs from the hands to the head, namely, the large intestine meridian, the small intestine meridian and the triple energizer meridian	上肢後面の手から頭へと走る経絡（大腸経、小腸経、三焦経）。
足三陽經		1.4.6	three yang meridians of the foot	the meridians/channels running from the head through the back downward to the feet, namely, the stomach meridian, the bladder meridian and the gallbladder meridian	頭から背部を通り足へと走る経絡（胃経、膀胱経、胆経）。
足三陰經		1.4.7	three yin meridians of the foot	the meridians/channels running through the medial side of the lower limbs from the feet to the abdomen and chest, namely, the spleen meridian, the kidney meridian and the liver meridian	下肢内側の足から腹部および胸部へ走る経絡（脾経、腎経、肝経）。
十四經	十四經;十四經脈	1.4.8	fourteen meridians	a collective term for the twelve regular meridians plus the governor and conception vessels	十二正経と督脈および任脈の総称。

検索表示（原表示の複数語を分離）	IST原表示	Code	Term	Definition/Description	定義/記述
十四經脈	十四經; 十四經脈	1.4.8	fourteen meridians	a collective term for the twelve regular meridians plus the governor and conception vessels	十二正経と督脈および任脈の総称。
十二經	十二經; 十二正經; 十二經脈	1.4.9	twelve meridians	a collective term for the three yin meridians and three yang meridians of each hand and foot, also the same as twelve regular meridians	手足それぞれの三陰経および三陽経の総称。twelve regular meridiansと同義。
十二正經	十二經; 十二正經; 十二經脈	1.4.9	twelve meridians	a collective term for the three yin meridians and three yang meridians of each hand and foot, also the same as twelve regular meridians	手足それぞれの三陰経および三陽経の総称。twelve regular meridiansと同義。
十二經脈	十二經; 十二正經; 十二經脈	1.4.9	twelve meridians	a collective term for the three yin meridians and three yang meridians of each hand and foot, also the same as twelve regular meridians	手足それぞれの三陰経および三陽経の総称。twelve regular meridiansと同義。
手太陰肺經		1.4.10	lung meridian (LU)	one of the regular twelve meridians which begins internally in the middle energizer, descends to connect with the large intestine, then ascends to the lung and throat, courses laterally and exits superficially at zhongfu (LUl), and then descends along the lateral side of the arm and forearm, terminates at shaoshang (LUl 1),with 11 acupuncture points on either side	十二正経の1つで、体内の中焦に起こり、下降して大腸につながる。肺および咽頭へと上昇し、横方向へ向き、中府（LU1）で体表に出る。その後、腕および前腕側面を下降し、少商（LU11）で終わる。（経穴数：11／片側）
手陽明大腸經		1.4.11	large intestine meridian (LI)	one of the regular twelve meridians which originates at shangyang (LI1) and ascends the dorsal surface of the hand and forearm, the lateral side of the arm, the dorsal side of the shoulder to jugu (LI16) where the meridian/channel enters internally and travels posteriorly to dazhui (GV14), and then courses anteriorly to the supraclavicular fossa where it descends past the diaphragm to connect with the large intestine. The superficial supraclavicular branch ascends the anterior lateral neck and the mandible, connects internally with the lower teeth, encircles the lips and terminates at the opposite yingxiang (LI20). There are 20 acupuncture points on either side of the body	十二正経の1つで、商陽（LI1）に起こり、手および前腕の背面を上昇し、腕の側面、肩の背面を循って巨骨（LI16）で体内に入り、後方の大椎（GV14）に向かう。その後、前方に向かい、鎖骨上窩より下降し、横隔膜を通過して大腸へつながる。鎖骨上窩で分かれた支脈は、頸部前側および下顎を上昇し、体内で下顎歯につながり、唇を循って反対側の迎香（LI20）で終わる。（経穴数：20／片側）
足陽明胃經		1.4.12	stomach meridian (ST)	one of the regular twelve meridians which originates internally at the lateral edge of the nose, then continues to ST1 at the inferior border of the orbit, descends to the upper gum, courses around the mouth, and travels up to ST8 at the hairline of the temple. From here it continues internally to terminate at GV24. The facial branch descends from ST5 where it turns internally and descends past the diaphragm to connect with the stomach and spleen. The supraclavicular fossa branch descends along the midclavicular line to ST30 in the inguinal region, then anteriorly along the lateral margin of the femur to the patella, terminating at ST45 on the lateral side of the tip of the second toe. The gastric branch descends internally past the umbilicus and terminates at ST30. The tibial branch leaves ST36 and descends along the fibula, terminating at the lateral side of the tip of the middle toe. The dorsal foot branch leaves ST42 and descends to the medial side of the great toe at SP1. There are 45 acupuncture points on either side of the body	十二正経の1つで、鼻の外側縁内部に起こり、内眼角へと上昇し、体表の最初の経穴である、眼窩下縁に位置する承泣（ST1）を通過後、上歯肉へと下降し、口腔を循り、こめかみの生え際に位置する頭維（ST8）に到達する。頭維より体内に入り、神庭（GV24）で終わる。顔の支脈は大迎（ST5）で体内へと入って下降し、横隔膜を通過して胃および脾臓へつながる。鎖骨上窩支脈は鎖骨中線に沿って鼠径部の気衝（ST30）へと下降し、大腿の外側縁に沿って膝蓋骨へと前方に進み、第2趾先端部側面の厲兌（ST45）で終わる。胃の支脈は体内を下降し、へそを通過して気衝（ST30）で終わる。脛骨の支脈は足三里（ST36）を発し、腓骨に沿って下降し、第3趾側面で終わる。背面の足の支脈は衝陽（ST42）を発し、第1趾内側の隠白（SP1）へと下降する。（経穴数：45／片側）
足太陰脾經		1.4.13	spleen meridian (SP)	one of the twelve regular meridians which runs from yinbai (SP1) at the medial side of the great toe, ascends along the medial side of the foot and tibia and anteromedial side of the thigh to the lower abdomen. It then enters the abdomen and connects with the spleen and stomach. The meridian ascends at a distance of 4.0 cun lateral to the conception vessel, and terminates superficially at dabao (SP21) in the sixth intercostal space on the midaxillary line. The meridian continues internally past the supraclavicular fossa and terminates at the base of the tongue. The gastric branch leaves the stomach and ascends internally past the diaphragm and connects with the heart. There are 21 acupuncture points on either side of the body	十二正経の1つで、第1趾内側の隠白（SP1）を発し、足および脛骨の内側と大腿部の前内側に沿って下腹部へと上昇する。腹部内に入り、脾臓および胃につながる。任脈の側方4寸の位置を上昇し、腋窩中線上の第6肋間間隙に位置する体表の大包（SP21）で終わる。体内では鎖骨上窩を通過し、舌根で終わる。胃の支脈は胃を発して体内を上昇し、横隔膜を通過して心臓につながる。
手少陰心經		1.4.14	heart meridian (HT)	one of the twelve regular meridians which originates in the heart, descends internally past the diaphragm and connects with the small intestine. The cardiac branch ascends internally paralateral to the esophagus and terminates at the eye. The main branch leaves the heart, traverses the lung and emerges superficially in the midaxilla at jiquan (HT1) and descends along the ulnar side of the forearm medially, terminating at shaochong (HT9) on the radial side of the tip of the small finger, with 9 acupuncture points on either side	十二正経の1つで、心臓に起こり、体内を下降し、横隔膜を通過して小腸につながる。心臓の支脈は体内で食道側面の近くを上昇し、眼で終わる。主脈は心臓を発し、肺を横断し、腋窩中央の极泉（HT1）で体表に現れ、上腕内側の尺側に沿って下降し、小指先端部の橈骨側にある少衝（HT9）で終わる。（経穴数：9／片側）

検索表示（原表示の複数語を分離）	IST原表示	Code	Term	Definition/Description	定義/記述
手太陽小腸經		1.4.15	small intestine meridian (SI)	one of the twelve regular meridians which originates at shaoze (SI1) at the ulnar side of the little finger, ascends the ulnar side of the forearm, the arm, over the scapula to dazhui (GV14) between the spinous processes of the 7th cervical and 1st thoracic vertebrae. The meridian then descends internally to the heart, and follows the esophagus past the diaphragm, to connect with the small intestine. The supraclavicular fossa branch ascends superficially along the lateral side of the neck, past the cheek to the lateral corner of the eye, and terminates at tinggong (SI19). the buccal branch leaves the main meridian at the cheek, and ascends to the medial canthus of the eye. There are 19 acupuncture points on either side of the body	十二正経の1つで、小指の尺側にある少沢（SI1）に起こり、上腕尺側、腕、肩甲骨を通過して第7頸椎と第1胸椎の棘突起間に位置する大椎（GV14）へと上昇する。その後、体内を心臓へと下降し、食道に沿って横隔膜を通過し、小腸につながる。鎖骨上窩の支脈は頸部側面に沿って体表を上昇し、頬および眼の側縁を通過して、聴宮（SI19）で終わる。頬の支脈は頬で主脈から分かれ、内眼角へと上昇する。（経穴数：19／片側）
足太陽膀胱經		1.4.16	bladder meridian (BL)	one of the twelve regular meridians which runs from jingming (BL1) at the medial canthus of the eye, ascends the forehead to the vertex and then enters the brain and exits at the nape of the neck where it divides into two parallel branches: the first branch descends the back at a distance of 1.5 cun from the spine, and during its course it connects with the kidney and bladder, and continues along the posterior thigh to the popliteal crease; the second branch descends the back at a distance of 3 cun from the spine, continues along the latero-posterior side of the thigh to popliteal fold where it meets the first branch. The meridian continues to descend along the posterior calf to the lateral malleolus, terminating at zhiyin (BL67) on the lateral side of the tip of the small toe. There are 67 acupuncture points on either side of the body	十二正経の1つで、内眼角の睛明（BL1）を発し、額から頭頂へと上昇後、脳に入り、うなじで体表に出て2本の平行に走る支脈へと分かれる。第1支脈は背部で背骨から1.5寸の位置を下降し、その途中で腎臓と膀胱につながり、大腿部後面に沿って膝窩へと続く。第2支脈は、背部で背骨から3寸の位置を下降し、大腿後側に沿って膝窩へと続き、ここで第1支脈と合流する。その後、ふくらはぎ後面に沿って外果へと降下し、第5趾先端部外側の至陰（BL67）で終わる。（経穴数：67／片側）
足少陰腎經		1.4.17	kidney meridian (KI)	one of the twelve regular meridians which begins on the plantar tip of the small toe and travels to yongquan (KI1) in the center of the sole, continues along the medial side of the lower limb to the symphysis pubis, turns internally to the kidney and bladder, and back to the symphysis pubis, ascending along the abdomen and chest up to shufu (KI27) in the depression between the first rib and the lower border of the clavicle, with 27 acupuncture points on either side	十二正経の1つで、第5趾裏先端部を発し、足底中心にある湧泉（KI1）を通り、下肢の内側に沿って恥骨結合に達する。ここで体内に入り、腎臓および膀胱に向かい、再び恥骨結合に戻り、腹部、胸部に沿って、第1肋骨と鎖骨下縁間の陥凹中にある兪府（KI27）まで上昇する。（経穴数：27／片側）
手厥陰心包經		1.4.18	pericardium meridian (PC)	one of the twelve regular meridians which originates in the center of the thorax, connects with the pericardium, and descends to the lower abdomen, linking all the three energizers. The thoracic branch exits superficially at tianchi (PC1) near the nipple, and descends along the midline of anterior side of the arm to zhongchong (PC9) at the midpoint of the tip of the middle finger, with 9 acupuncture points on either side	十二正経の1つで、胸部の中心で起こり、心膜につながり、3焦を結びながら下腹部へと降下する。胸部の支脈は、乳頭近くの天地（PC1）で体表に現れ、腕前側の正中線に沿って下降し、中指先端部の中点にある中衝（PC9）に到達する。（経穴数：9／片側）
手少陽三焦經		1.4.19	triple energizer meridian (TE)	one of the twelve regular meridians which runs from guanchong (TE1) at the ulnar side of the ring finger, travels along the midline of the posterior side of the arm and through the regions of the shoulder, neck, ear and eye, and terminates at sizhukong (TE23) at the lateral aspect of canthus. A branch is sent from the supraclavicular fossa to the pericardium and down through the thorax and abdomen, linking the upper, middle and lower energizers. There are 23 acupuncture points on either side of the body	十二正経の1つで、薬指の尺側にある関衝（TE1）を発し、腕後側の正中線に沿って走り、肩、頸部、耳、眼を通り、外眼角の絲竹空（TE23）で終わる。支脈は鎖骨上窩から心膜に向かい、胸部、腹部を通過して上焦、中焦、下焦を結ぶ。（経穴数：23／片側）
足少陽膽經		1.4.20	gallbladder meridian (GB)	one of the twelve regular meridians which runs from tongziliao (GB1) at the lateral canthus of the eye, through the regions of the temple, ear, neck, shoulder, flank, and the outer side of the lower limb, terminating at zuqiaoyin (GB44) on the lateral side of the tip of the 4th toe. The post-auricular branch travels into the ear and down to the supraclavicular fossa where it joins the original branch. It continues down into the chest, past the diaphragm, connecting with the gallbladder and liver, and travels down to the lower abdomen into the inguinal canal. The dorsal foot branch leaves the main meridian at zulinqi (GB41) and descends between the first and second metatarsals, terminating at the base of the great toe nail. There are 44 acupuncture points on either side of the body	十二正経の1つで、外眼角の瞳子髎（GB1）を発し、こめかみ、耳、頸部、肩、横腹、下肢の外側を走り、第4趾先端部外側の足竅陰（GB44）で終わる。耳介後部から始まる支脈は、耳内部を通って降下し、鎖骨上窩で主脈に合流する。主脈は胸部を降下し、横隔膜を通過して胆嚢、肝臓につながり、さらに下腹部から鼠径管へと続く。足背面の支流は足臨泣（BG41）で主脈から分かれ、第1および第2中足骨の間を下降し、第1趾の爪基部で終わる。（経穴数：44／片側）
足厥陰肝經		1.4.21	liver meridian (LR)	one of the twelve regular meridians which runs from dadun (LR1) on the medial aspect of the big toe just behind the nail, through the inner side of the lower limb, external genitalia and abdomen, to qimen (LR14), a point about 2 cun below the nipple. From qimen (LR14) the meridian/channel enters the abdomen, traverses through the stomach paralaterally to connect with the liver and gallbladder. From the liver, the meridian/channel ascends past the diaphragm along the trachea, larynx, sinus cavity, connecting with the eye, and then ascends to the vertex where it meets the governor vessel at baihui (GV20). There are 14 acupuncture points on either side of the body	十二正経の1つで、第1趾の爪基部の直後にある大敦（LR1）を発し、下肢の内側、外性器および腹部を通り、乳頭下約2寸に位置する期門（LR14）に至る。期門（LR14）より腹部に入り、胃を横断し、肝臓および胆嚢につながる。肝臓より上昇し、横隔膜を通過して気管、喉頭、副鼻腔に沿って走り、眼につながる。その後、頭頂へと上昇し、百会（GV20）で督脈に合流する。（経穴数：14／片側）
正經		1.4.22	main meridian	another name of twelve meridians, also called regular meridian	十二正経の別名。regular meridianとも呼ばれる。
奇經		1.4.23	extra meridian	an abbreviation for eight extra meridians	奇経八脈の略称。
奇經八脈		1.4.24	eight extra meridians	a collective term for governor vessel, conception vessel, thoroughfare vessel, belt vessel, yin heel vessel, yang heel vessel, yin link vessel, and yang link vessel	督脈、任脈、衝脈、帯脈、陰蹻脈、陽蹻脈、陰維脈、陽維脈の総称。

検索表示（原表示の複数語を分離）	IST原表示	Code	Term	Definition/Description	定義/記述
督脈		1.4.25	governing vessel (GV)	one of the eight extra meridians which originates in the lower abdomen and exits at changqiang (GV1), a point at the back of the anus, sending one branch forward to huiyin (CV1). The main portion of the meridian/channel ascends along the midline of the back to the top of the head and then descends along the midline of the face down to yinjiao (GV28), a point between the upper lip and the upper gum in the labia frenum, also called governing vessel	奇経八脈の1つで、下腹部に起こり、肛門後部に位置する長強（GV1）で体外に出る。支脈が会陰（CV1）へと走る。主脈は背部の正中線に沿って上昇し、頭頂に到達し、その後、顔の正中線を通り、上唇と上唇小帯の上歯肉の間にある齦交（GV28）へと下降する。
任脈		1.4.26	controlling vessel	one of the eight extra meridians which originates in the lower abdomen, exists at huiyin (CV1), a point in the center of perineum, and ascends the midline of the abdominal wall and chest to chengjiang (CV24), midpoint of the mentolabial sulcus. The internal portion of this meridian/channel ascends from chengjiang (CV24), encircling the mouth and traveling to the eyes. Another branch travels internally from the pelvic cavity and ascends the spine to the throat, also called controlling vessel	奇経八脈の1つで、下腹部に起こり、会陰の中心にある会陰（CV1）で体外に出る。腹壁および胸部の正中線を通って上昇し、オトガイ唇溝の中点である承漿（CV24）に至る。体内では承漿（CV24）より上昇し、口の周りを回り、眼に到達する。もう1本の支脈は、体内で骨盤腔を発し、脊柱を上昇して咽頭に至る。cotrolling vesselとも呼ばれる。
衝脈		1.4.27	thoroughfare vessel	one of the eight extra meridians which originates in the lower abdomen, exits at the perineum, and comes into confluence with kidney meridian and then runs upward along the two sides of the abdomen to the chest	奇経八脈の1つで、下腹部に起こり、会陰で体表に出て腎経と合流し、腹部から胸部の両側に沿って上昇する。
帯脈		1.4.28	belt vessel	one of the eight extra meridians which originates from the lower part of hypochondrium and passes around the waist	奇経八脈の1つで、下肋部に起こり、胴の周りを回る。
陰蹻脈		1.4.29	yin heel vessel	one of the eight extra meridians which originates from the medial side of the heel, running upward along the medial side of the lower limb, through the front external genitalia, the abdomen, chest, neck, either side of the nose and terminating in the eye	奇経八脈の1つで、かかとの内側に起こり、下肢内側に沿って上昇し、外性器より体内に入り、腹部、胸部、頸部、鼻の片側を通り、眼で終わる。
陽蹻脈		1.4.30	yang heel vessel	one of the eight extra meridians which originates from the lateral side of the heel, running upward along the outer ankle, the lateral side of the lower limb, through the abdomen, chest, shoulder and cheek, terminating at the back of the neck	奇経八脈の1つで、かかとの外側に起こり、足首および下肢の外側に沿って上昇し、腹部、胸部、肩、頬を通り、後頸部で終わる。
陰維脈		1.4.31	yin link vessel	one of the eight extra meridians which originates from the upper part of the inner ankle, running upward along the medial side of the lower limb, through the abdomen, chest and throat, terminating at the back of the neck	奇経八脈の1つで、足首内側上部に起こり、下肢の内側に沿って上昇し、腹部、胸部、喉頭を通し、後頸部で終わる。
陽維脈		1.4.32	yang link vessel	one of the eight extra meridians which originates from the lower part of the outer ankle running upward along the lateral side of the lower limb, through the side of the trunk, the shoulder, neck and terminating at the top of the head	奇経八脈の1つで、足首外側下部に起こり、下肢の外側に沿って上昇し、胴体、肩および頸部の片側を通り、頭頂で終わる。
十二經別		1.4.33	twelve meridian divergences	the divergent passages of the twelve main meridians going deep in the body	十二正経から分岐する支脈で、身体の深部を走る。
經別		1.4.34	meridian divergence	the divergent passage of a regular meridian going deep in the body	正経から分岐する支脈で、身体の深部を走る。
十二經筋		1.4.35	twelve meridian sinews	the sinew systems attributed to the twelve meridians	十二正経を起源とする筋系。
經筋		1.4.36	meridian sinew	the sinew system attributed to a certain meridian/channel	特定の経絡を起源とする筋系。
十二皮部		1.4.37	twelve cutaneous regions	the regions of the skin reflecting the functional condition of the twelve meridians respectively	十二正経それぞれの機能状況を反映する皮膚の部位。
皮部		1.4.38	cutaneous region	the region of the skin reflecting the functional condition of a certain meridian	特定の経絡の機能状況を反映する皮膚の部位。
絡脈		1.4.39	collateral vessel	the small branches of the meridians, serving as a network linking the various aspects of the body	経絡の支脈で、全身に分布し、経絡を結ぶ。
十五絡脈		1.4.40	fifteen collateral vessels	a collective term referring to the main collaterals derived from the fourteen meridians and together with the great collateral of the spleen, fifteen in all	十四経脈に由来する主脈の総称。脾の大絡を加えて絡脈数は15本。
脾之大絡		1.4.41	great collateral vessel of the spleen	the collateral that emerges from dabao (SP21) and spread over the thoracic and hypochondriac regions	大包（SP21）より胸部および下肋部へ広がる。
孫絡		1.4.42	tertiary collateral vessel	small branches of the collateral/network, also known as grandchild collateral vessel	絡脈の支脈で、grandchild collateral vesselとしても知られる。
浮絡		1.4.43	superficial collateral vessel	collateral/network vessels in the superficial layers of the body	身体の表層を走る絡脈。
形體和官竅;身體構造		**1.5.0**	**Body Constituents and Orifices of Sense Organ**		
形體		1.5.1	body constituent	a collective term for skin, vessels, flesh, sinews and bones	皮膚、脈管、肌肉、腱および骨の総称。
皮毛		1.5.2	skin and (body) hair	a collective term for the skin and its fine hair	皮膚および体毛の総称。
腠理		1.5.3	interstices	a term referring to the striae of the skin, muscles and viscera, and also to the tissue between the skin and muscles	皮膚、筋肉、および内臓の紋理、および皮膚と筋肉の間隙を埋める組織を指す。
玄府		1.5.4	mysterious mansion	another name for sweat pore. It is so named because it is too minute to be visible	汗孔の別名。あまりにも小さく、目に見えないことに由来する。
氣門		1.5.5	qi gate	another name for sweat pore	汗孔の別名。
赤白肉際		1.5.6	border between the red and white flesh	the skin boundary between the palm or sole (red in color) and the back of the hand or foot (white in color), respectively	掌部（赤色）と手の甲(白色）または足底部（赤色）と足裏(白色）間の皮膚の境界部。
筋		1.5.7	sinew	tough band or cord of tissue that joins muscle to bone	筋肉を骨に結合する、弾力性のある腱または索状組織。
宗筋		1.5.8	ancestral sinew	a collective term for sinews/male external genitalia	腱／男性の外性器の総称。
溪谷		1.5.9	muscle interspace	the gap junction or depression between two muscles	2つの筋肉間のギャップ結合部または陥凹。
膻中		1.5.10	chest center	the center of the chest between the nipples	乳頭間にある胸部の中心。

検索表示（原表示の複数語を分離）	IST原表示	Code	Term	Definition/Description	定義/記述
胃脘		1.5.11	stomach duct	(1) stomach cavity and adjoining section of the esophagus; (2) epigastrium	(1) 胃腔および食道との接続部、(2) 心窩部。
胸脇		1.5.12	chest and hypochondrium	the portion of the body between the neck and the abdomen and the superolateral regions of the abdomen, overlying the costal cartilages	頸部と腹部の間の、腹部外側の肋軟骨より上の部位。
募原	募原; 膜原	1.5.13	membrane source	(1) pleurodiaphragmatic interspace; (2) interior-exterior interspace where the pathogens of epidemic febrile disease tends to settle	(1) 胸膜と横隔膜の間の空間、(2) 伝染性熱性疾患の病原体が定着する、体内と体外の間の空間。
膜原	募原; 膜原	1.5.13	membrane source	(1) pleurodiaphragmatic interspace; (2) interior-exterior interspace where the pathogens of epidemic febrile disease tends to settle	(1) 胸膜と横隔膜の間の空間、(2) 伝染性熱性疾患の病原体が定着する、体内と体外の間の空間。
膏肓		1.5.14	cardiodiaphragmatic interspace	the space inferior to the heart and superior to the diaphragm	心臓の下、横隔膜の上の空間。
小腹		1.5.15	lower abdomen	the part of abdomen between the umbilicus and the upper margin of pubic bone	腹部の、へそと恥骨上縁の間の部位。
少腹		1.5.15	lower abdomen	the part of abdomen between the umbilicus and the upper margin of pubic bone	腹部の、へそと恥骨上縁の間の部位。
臍傍		1.5.16	para-umbilical region	that part of abdomen lateral to the umbilicus	腹部の、へその横の部位。
臍下		1.5.17	infra-umbilical region	that part of abdomen inferior to the umbilicus	腹部の、へその下の部位。
丹田		1.5.18	cinnabar field	three regions of the body to which one's mind is focused while practicing qigong: the lower cinnabar field - the region located in the upper 2/3 of the line joining the umbilicus and symphysis pubis; the middle cinnabar field - the xiphoid area; and the upper cinnabar field - the region between the eyebrows	気功において、精神が集中する身体の3つの部位を指す：下丹田（へそと恥骨結合を結ぶ線の上部3分の2に位置する部位）、中丹田（剣状突起の部位）、上丹田（両眉の間）。
骸	骸; 百骸	1.5.19	skeleton	the supportive structure or framework of the body	身体の支持構造または枠組み。
百骸	骸; 百骸	1.5.19	skeleton	the supportive structure or framework of the body	身体の支持構造または枠組み。
眉稜骨		1.5.20	eyebrow bone	the upper ridge of the orbital bone	眼窩上隆起。
頸骨		1.5.21	cervical vertebrae	a collective term for the cervical vertebrae	頸椎の総称。
脊		1.5.22	vertebrae	a collective term of the thoracic, lumbar and sacral vertebrae, the same as spine	胸椎、腰椎、仙椎の総称。spineと同義。
腰骨		1.5.23	lumbar vertebrae	lumbar bone	腰骨。
輔骨		1.5.24	assisting bone	the bony prominences on the sides of the knee, namely, the condyles of femur and the condyles of tibia	膝側面にある骨の突起、すなわち大腿骨および脛骨の顆骨頭を指す。
高骨		1.5.25	high bone	any bony process of the body surface, particularly referring to the styloid process of the radius	体表面の骨突起、特に橈骨の茎状突起を指す。
胂		1.5.26	prominent muscle	(1) paravertebral muscle (2) the muscle below the iliac crest	(1) 傍脊椎筋、(2) 腸骨稜下の筋肉。
精明之府		1.5.27	house of bright essence	an expression referring to the head	頭を指す。
腦戶		1.5.28	back of the head	the occipital region	後頭部。
太陽	太陽; 太陽穴	1.5.29	temple	(1) the area on each side of the forehead above the cheek bones, lateral to and slightly superior to the outer canthus of the eye; (2) an non-meridian acupuncture point on the temporal part of the head	(1) 額の左右それぞれの側で、頬骨上部、外側眼角の側部、やや上側に位置する部位、(2) 側頭部に位置する奇穴。
太陽穴	太陽; 太陽穴	1.5.29	temple	(1) the area on each side of the forehead above the cheek bones, lateral to and slightly superior to the outer canthus of the eye; (2) an non-meridian acupuncture point on the temporal part of the head	(1) 額の左右それぞれの側で、頬骨上部、外側眼角の側部、やや上側に位置する部位、(2) 側頭部に位置する奇穴。
官竅		1.5.30	orifice of sense organ	a general term for the external opening of sence organs	感覚器官の開口部の総称。
五官		1.5.31	five sense organs	a collective term of the nose, eyes, mouth, tongue and ears, associated with five phase theory	五行学説における、鼻、眼、口、下、舌の総称。
七竅		1.5.32	seven orifices	a collective term of the two ears, two eyes, two nostrils and the mouth	左右の耳、眼および鼻孔、ならびに口の総称。
上竅		1.5.33	upper orifices	the eyes, ears, mouth and nose	眼、耳、口および鼻。
下竅		1.5.34	lower orifices	the anus and genito-urinary openings	肛門および泌尿生殖器口。
苗竅		1.5.35	sprout orifices	the sense organs that reflect the change of qi, blood, yin and yang, also known as signaling orifices/sense organs	気、血、陰および陽の変化を示す感覚器官。signaling orifices/sense organsとしても知られる。
五輪		1.5.36	five wheels	five regions of the eye from the outer to the inner: the flesh wheel, blood wheel, qi wheel, wind wheel and water wheel, also the same as five orbiculi	眼の5つの部位（外側から内側に向かって肉輪、血輪、気輪、風輪、水輪）。
八廓		1.5.37	eight belts	a collective term of the eight external ocular regions	眼の8つの外部部位の総称。
氣輪		1.5.38	qi wheel	the bulbar conjunctiva and sclera, also the same as qi orbiculus	眼球結膜および強膜。qi orbiculusと同義。
水輪		1.5.39	water wheel	the pupil, also the same as water orbiculus	瞳孔。water orbiculusと同義。
血輪		1.5.40	blood wheel	the canthus, also the same as blood orbiculus	眼角。blood orbiculusと同義。
風輪		1.5.41	wind wheel	the cornea, also the same as wind orbiculus	角膜。wind orbiculusと同義。
肉輪		1.5.42	flesh wheel	the eyelids, also the same as flesh orbiculus	眼瞼。flesh orbiculusと同義。
大眥		1.5.43	inner canthus	the canthus closer to the nose, the same as greater canthus	鼻側の眼角。greater canthusと同義。
鋭眥	鋭眥; 小眥	1.5.44	outer canthus	the canthus closer to the temple, the same as lesser canthus	こめかみ側の眼角。lesser canthusと同義。
小眥	鋭眥; 小眥	1.5.44	outer canthus	the canthus closer to the temple, the same as lesser canthus	こめかみ側の眼角。lesser canthusと同義。
涙堂		1.5.45	lacrimal orifice	the opening from which tears flow	涙が流れ出る開口部。
白睛		1.5.46	white of the eye	the white opaque part of the outer surface of the eyeball	眼球外面の、白く不透明な部分。

検索表示（原表示の複数語を分離）	IST原表示	Code	Term	Definition/Description	定義/記述
黑睛		1.5.47	dark of the eye	the transparent membranous structure forming the central anterior part of the eye, i.e. the cornea	前眼部の中央を形成する透明な膜構造（角膜）。
瞳神		1.5.48	pupil	the opening at the center of the iris, posterior to the cornea, through which light enters the eye	虹彩の中央、角膜の後方に位置する開口部（瞳孔）。光は瞳孔を通して入射する。
神水		1.5.49	aqueous humor	the fluid produced in the eye, occupying the space between the crystalline lens and cornea	眼内で生成される液体。水晶体と角膜の間の空間を満たす。
神膏		1.5.50	vitreous humor	the clear colorless transparent jelly that fills the eyeball	眼球を満たす無色透明のゼリー状の物質。
目系		1.5.51	eye connector	the cord connecting the eye with the brain	眼と脳を結ぶ索状組織。
目眶	目眶; 目眶骨	1.5.52	eye socket	the bony cavity that contains the eye	眼球を収容する頭蓋骨のくぼみ。
目眶骨	目眶; 目眶骨	1.5.52	eye socket	the bony cavity that contains the eye	眼球を収容する頭蓋骨のくぼみ。
目上網		1.5.53	sinew mesh above the eyes	upper palpebral musculature	上眼瞼の筋組織。
目下網		1.5.54	sinew mesh below the eyes	lower palpebral musculature	下眼瞼の筋組織。
明堂		1.5.55	bright hall	an ancient term for nose, especially the apex of the nose	鼻、特に先端部を指す古来の用語。
鼻準		1.5.56	tip of the nose	the most distal portion of the nose, the same as apex nasi	鼻の先端部（鼻先）を指す。apex nasiと同義。
山根		1.5.57	root of the nose	the upper portion of the nose, which is situated between the eyes, the same as radix nasi	眼の間に位置する鼻の上部（鼻根）。radix nasiと同義。
頞	頞; 鼻莖	1.5.58	bridge of the nose	that part of the nose formed by the junction of its lateral surfaces, the same as dorsum nasi	鼻外側面の接合部で形成される部分（鼻筋）。dorsum nasiと同義。
鼻莖	頞; 鼻莖	1.5.58	bridge of the nose	that part of the nose formed by the junction of its lateral surfaces, the same as dorsum nasi	鼻外側面の接合部で形成される部分（鼻筋）。dorsum nasiと同義。
眞牙		1.5.59	wisdom tooth	the third molar tooth	第3大臼歯。
蒂丁	蒂丁; 小舌	1.5.60	uvula	the pendular fleshy lobe in the middle of the posterior border of the soft palate, usually referring to uvula palatina	軟口蓋の後縁中央に垂れ下がっている部分（口蓋垂）。通常uvula palatinaを指す。
小舌	蒂丁; 小舌	1.5.60	uvula	the pendular fleshy lobe in the middle of the posterior border of the soft palate, usually referring to uvula palatina	軟口蓋の後縁中央に垂れ下がっている部分（口蓋垂）。通常uvula palatinaを指す。
喉核		1.5.61	throat node	faucial or palatine tonsil, a pair of prominent masses that lie one on each side of the throat	口峡または口蓋扁桃。喉の左右に1つづつある隆起。
喉關		1.5.62	throat pass	that part of the throat formed by the tonsils, uvula and back of the tongue	扁桃腺、口蓋垂および舌後部で構成される喉の部位。
喉嗌		1.5.63	pharynx	the part of the throat through which food or drink is swallowed	嚥下された飲食物が通る喉の部位。
喉底		1.5.64	retropharynx	the posterior part of the pharynx	咽頭後部。
頏顙		1.5.65	nasopharynx	the upper part of the pharynx continuous with the nasal passages	咽頭の上部。鼻腔につながっている。
精室		1.5.66	essence chamber	the part of the body where the semen is stored in a male	精液が貯蔵される、男性の臓器。
精竅		1.5.67	essence orifice	the external orifice of the male urethra, from which the semen is discharged	精液が放出される、男性の尿道外口。
睾		1.5.68	testicle	the male reproductive organ where the sperms are produced	精子が形成される、男性の生殖器。
前陰		1.5.69	anterior yin	the external genitalia including the external orifice of the urethra	尿道外口を含む外性器。
後陰		1.5.70	posterior yin	the anus, the posterior opening of the large intestine	肛門（大腸の後部開口）。
衛分		1.5.71	defense aspect	the most superficial stratum of the body apt to be invaded at the initial stage of an acute febrile disease, often referring to the lung	身体の最も浅い位置にある層で、急性熱性疾患の初期段階で侵される。多くの場合、肺を指す。
氣分		1.5.72	qi aspect	the second stratum of the body deeper than the defense aspect, often referring to the lung, gallbladder, spleen, stomach and large intestine	衛分に続く身体の2番目の層で、多くの場合、肺、胆嚢、脾臓、胃、大腸を指す。
營分		1.5.73	nutrient aspect	that stratum of the body between the qi and blood aspects	気分と血分の間の層。
血分		1.5.74	blood aspect	the deepest stratum of the body involved in the severest stage of an acute febrile disease	身体の最も深い位置にある層で、急性熱性疾患の最重度の段階で影響を受ける。
病因		**1.6.0**	**Cause of Disease**		
病因		1.6.1	cause of disease	pathogenic factor	病原因子。
病因學說		1.6.2	theory of causes of disease	the theory dealing with the classification of pathogenic factors and their properties, and pathogenic characteristics and processes, the same as etiology	病原因子の分類、病原因子の特性、病原性の特徴および発病過程を扱う。病因学と同義。
病邪	病邪; 邪氣; 邪	1.6.3	pathogen	an agent causing disease, also called pathogenic factor or pathogenic qi	疾患を引き起こす病原。pathogenic factorまたはpathogenic qiとも呼ばれる。
邪氣	病邪; 邪氣; 邪	1.6.3	pathogen	an agent causing disease, also called pathogenic factor or pathogenic qi	疾患を引き起こす病原。pathogenic factorまたはpathogenic qiとも呼ばれる。
邪	病邪; 邪氣; 邪	1.6.3	pathogen	an agent causing disease, also called pathogenic factor or pathogenic qi	疾患を引き起こす病原。pathogenic factorまたはpathogenic qiとも呼ばれる。
外邪	外邪; 客邪	1.6.4	external pathogen	any pathogen originating outside the body	体外に由来する病邪。
客邪	外邪; 客邪	1.6.4	external pathogen	any pathogen originating outside the body	体外に由来する病邪。
時邪		1.6.5	seasonal pathogen	a general designation for the pathogenic factors causing seasonal diseases	季節病を引き起こす病原因子の一般名。
陽邪		1.6.6	yang pathogen	pathogen of yang nature	陽の病邪。
陰邪		1.6.7	yin pathogen	pathogen of yin nature	陰の病邪。
合邪		1.6.8	combined pathogen	any combination of more than one pathogen	複数の病邪の組み合わせ。

検索表示（原表示の複数語を分離）	IST原表示	Code	Term	Definition/Description	定義/記述
外感		1.6.9	external contraction	disease or morbid condition produced by any of the six excesses (external etiological factors) or other noxious factors, the same as exopathic disease	六淫(外的な病因要素)あるいは他の有害要素によって起こる疾患または病的状態。exopathic diseaseと同義。
六淫		1.6.10	six excesses	a collective term for the six excessive or untimely climatic influences as external pathogenic factors: wind, cold, summerheat, dampness, dryness and fire, also the same as six climatic pathogenic factors	6つの過度あるいは不順な気候の影響の総称(風邪、寒邪、暑邪、湿邪、燥邪、火邪)。six climatic pathogenic factorsと同義。
風	風; 風邪	1.6.11	wind	wind as a pathogenic factor characterized by its rapid movement, swift changes, and ascending and opening actions, also called pathogenic wind	病原因子としての風。機敏な動き、素早い変化、上昇および解放作用を特徴とする。pathogenic windとも呼ばれる。
風邪	風; 風邪	1.6.11	wind	wind as a pathogenic factor characterized by its rapid movement, swift changes, and ascending and opening actions, also called pathogenic wind	病原因子としての風。機敏な動き、素早い変化、上昇および解放作用を特徴とする。pathogenic windとも呼ばれる。
寒	寒; 寒邪	1.6.12	cold	cold as a pathogenic factor characterized by the damage to yang qi, deceleration of activity, congealing and contracting actions, also called pathogenic cold	病原因子としての寒さ。陽気への悪影響、活動の減速、凝固および収縮作用を特徴とする。pathogenic coldとも呼ばれる。
寒邪	寒; 寒邪	1.6.12	cold	cold as a pathogenic factor characterized by the damage to yang qi, deceleration of activity, congealing and contracting actions, also called pathogenic cold	病原因子としての寒さ。陽気への悪影響、活動の減速、凝固および収縮作用を特徴とする。pathogenic coldとも呼ばれる。
暑	暑; 暑邪	1.6.13	summerheat	summerheat as a pathogenic factor, only occurring in summer, characterized by hotness and evaporation, also called pathogenic summerheat	病原因子としての暑さ。夏にのみ発生する。暑さおよび蒸発を特徴とする。pathogenic summerheatとも呼ばれる。
暑邪	暑; 暑邪	1.6.13	summerheat	summerheat as a pathogenic factor, only occurring in summer, characterized by hotness and evaporation, also called pathogenic summerheat	病原因子としての暑さ。夏にのみ発生する。暑さおよび蒸発を特徴とする。pathogenic summerheatとも呼ばれる。
濕	濕; 濕邪	1.6.14	dampness	dampness as a pathogenic factor characterized by its impediment to qi movement and its turbidity, heaviness, stickiness and downward flowing properties, also called pathogenic dampness	病原因子としての湿気。気機の阻害と混乱、重さ、粘性、および下方への流れを特徴とする。pathogenic dampnessとも呼ばれる。
濕邪	濕; 濕邪	1.6.14	dampness	dampness as a pathogenic factor characterized by its impediment to qi movement and its turbidity, heaviness, stickiness and downward flowing properties, also called pathogenic dampness	病原因子としての湿気。気機の阻害と混乱、重さ、粘性、および下方への流れを特徴とする。pathogenic dampnessとも呼ばれる。
燥	燥; 燥邪	1.6.15	dryness	dryness as a pathogenic factor characterized by dryness and is apt to injure the lung and consume fluid, also called pathogenic dryness	病原因子としての乾燥。乾燥を特徴とし、肺を傷つけ、体液を消耗させる傾向にある。 pathogenic drynessとも呼ばれる。
燥邪	燥; 燥邪	1.6.15	dryness	dryness as a pathogenic factor characterized by dryness and is apt to injure the lung and consume fluid, also called pathogenic dryness	病原因子としての乾燥。乾燥を特徴とし、肺を傷つけ、体液を消耗させる傾向にある。 pathogenic drynessとも呼ばれる。
火	火; 火邪	1.6.16	fire	fire as a pathogenic factor characterized by intense heat that is apt to injure fluid, consume qi, engender wind, inducing bleeding, and disturb the mental activities, also called pathogenic fire	病原因子としての火。激しい熱を特徴とし、体液に害を及ぼし、気を消耗させ、出血を含む風邪を引き起こし、精神活動を害する傾向にある。pathogenic fireとも呼ばれる。
火邪	火; 火邪	1.6.16	fire	fire as a pathogenic factor characterized by intense heat that is apt to injure fluid, consume qi, engender wind, inducing bleeding, and disturb the mental activities, also called pathogenic fire	病原因子としての火。激しい熱を特徴とし、体液に害を及ぼし、気を消耗させ、出血を含む風邪を引き起こし、精神活動を害する傾向にある。pathogenic fireとも呼ばれる。
熱	熱; 熱邪	1.6.17	heat	heat as a pathogenic factor that causes heat pattern/syndrome, also called pathogenic heat	病原因子としての熱。熱証を引き起こす。pathogenic heatとも呼ばれる。
熱邪	熱; 熱邪	1.6.17	heat	heat as a pathogenic factor that causes heat pattern/syndrome, also called pathogenic heat	病原因子としての熱。熱証を引き起こす。pathogenic heatとも呼ばれる。
外風		1.6.18	external wind	wind as one of the six excesses that causes external wind pattern/syndrome	六淫の1つの風邪で、外風証を引き起こす。
外寒		1.6.19	external cold	cold as one of the six excesses that causes external cold pattern/syndrome	六淫の1つの寒邪で、外寒証を引き起こす。
外濕		1.6.20	external dampness	dampness as one of the six excesses that causes externally contracted dampness pattern/syndrome	六淫の1つの湿邪で、外湿証を引き起こす。
外燥		1.6.21	external dryness	dryness as one of the six excesses that causes external dryness pattern/syndrome	六淫の1つの燥邪で、外燥証を引き起こす。
內風		1.6.22	internal wind	the same as liver wind, wind in the interior due to abnormal movement of body’s yang qi	肾風と同義。身体の陽気の異常な動きに起因する体内の風。
內寒		1.6.23	internal cold	cold in the interior due to deficiency of yang qi or preponderance of yin cold	陽気不足または陰寒の優勢に起因する体内の寒。
內濕		1.6.24	internal dampness	dampness produced in the body due to yang deficiency of the spleen and kidney with decreased fluid transportation and transformation and resultant water stagnation	脾臓および腎臓の陽虚に起因する体内の湿。津の運化低下とそれによる水の停滞を伴う。
內燥		1.6.25	internal dryness	dryness in the interior due to consumption of body fluid	体液の消耗に起因する体内の燥。
風寒		1.6.26	wind-cold	a combined pathogen of external wind and cold	外風と外寒が組み合わさった病邪。
風熱		1.6.27	wind-heat	a combined pathogen of external wind and heat	外風と外熱が組み合わさった病邪。
風濕		1.6.28	wind-dampness	(1) a combined pathogen of external wind and dampness; (2) arthritis with soft tissue inflammation	(1) 外風と外湿が組み合わさった病邪、(2) 軟組織の炎症を伴う関節炎。
風燥		1.6.29	wind-dryness	a combined pathogen of external wind and dryness	外風と外燥が組み合わさった病邪。
風痰		1.6.30	wind-phlegm	a combined pathogen of wind and phlegm	風邪と痰が組み合わさった病邪。
寒濕		1.6.31	cold-dampness	a combined pathogen of cold and dampness	風邪と湿邪が組み合わさった病邪。
風寒濕		1.6.32	wind-cold-dampness	a combined pathogen of wind, cold and dampness	風邪、寒邪および湿邪が組み合わさった病邪。
濕熱		1.6.33	dampness-heat	a combined pathogen of dampness and heat	湿邪と熱邪が組み合わさった病邪。
濕火		1.6.34	dampness-fire	depressed dampness transforming into fire that damages spleen-stomach yin	低下した湿邪が火邪に変化し、脾胃陰を損傷する。
濕濁		1.6.35	dampness turbidity	the same as dampness. It describes the heavy, viscid and turbid nature of dampness	湿邪と同義。湿邪の重く、粘着性の、濁った性質を表す。
濁邪		1.6.36	turbid pathogen	pathogenic dampness-turbidity or phlegm-turbidity, usually impeding the movement of yang qi	病邪となる湿濁または痰濁で、通常、陽気の動きを低下させる。

検索表示（原表示の複数語を分離）	IST原表示	Code	Term	Definition/Description	定義/記述
穢濁		1.6.37	foul turbidity	putrid and filthy qi that causes disease, including miasma	病因となる穢濁の氣。瘴気を含む。
暑氣		1.6.38	summerheat qi	summerheat as a pathogenic factor	病原因子としての暑さ。
暑熱		1.6.39	summerheat-heat	summerheat as a pathogen that gives rise to disease characterized by heat symptoms	病邪としての暑さで、熱証を特徴とする疾患を引き起こす。
暑濕		1.6.40	summerheat-dampness	a combined pathogen of summerheat and dampness	暑邪と湿邪が組み合わさった病邪。
燥氣		1.6.41	dryness qi	dryness as a pathogenic factor	病原因子としての乾燥。
燥熱		1.6.42	dryness-heat	a combined pathogen of dryness and heat	燥邪と暑邪が組み合わさった病邪。
凉燥		1.6.43	cool dryness	the pathogenic factor that causes cool dryness pattern/syndrome	涼燥証を引き起こす病原因子。
溫燥		1.6.44	warm dryness	the pathogenic factor that causes warm dryness pattern/syndrome	温燥証を引き起こす病原因子。
溫邪		1.6.45	warm pathogen	a collective term for various pathogens causing acute febrile diseases	急性熱性疾患を引き起こすさまざまな病邪の総称。
疫癘		1.6.46	pestilence	a collective term for various highly infectious epidemic diseases	感染性の強い流行性疾患の総称。
伏氣	伏氣; 伏邪	1.6.47	latent qi	the pathogen that induces the onset of disease after a latent period, also called incubative pathogen	潜伏期を経た後に発症を引き起こす病邪。incubative pathogenとも呼ばれる。
伏邪	伏氣; 伏邪	1.6.47	latent qi	the pathogen that induces the onset of disease after a latent period, also called incubative pathogen	潜伏期を経た後に発症を引き起こす病邪。incubative pathogenとも呼ばれる。
時行戾氣	時行戾氣; 癘氣; 疫毒	1.6.48	epidemic pathogen	pathogen that cause an epidemic infectious diseases, also called pestilential qi	伝染性感染症を引き起こす病邪。pestilential qiとも呼ばれる。
癘氣	時行戾氣; 癘氣; 疫毒	1.6.48	epidemic pathogen	pathogen that cause an epidemic infectious diseases, also called pestilential qi	伝染性感染症を引き起こす病邪。pestilential qiとも呼ばれる。
疫毒	時行戾氣; 癘氣; 疫毒	1.6.48	epidemic pathogen	pathogen that cause an epidemic infectious diseases, also called pestilential qi	伝染性感染症を引き起こす病邪。pestilential qiとも呼ばれる。
毒		1.6.49	toxin	any virulent pathogen that causes a fulminating disease	劇症疾患を引き起こす毒性の病邪。
熱毒		1.6.50	heat toxin	a virulent pathogen arising from accumulated pathogenic heat	熱邪の蓄積によって生じる毒性の病邪。
火毒		1.6.51	fire toxin	(1) pathogenic agent formed in the course of smoldering of pathogenic fire; (2) burns or scalds complicated with infection	(1) 火邪のくすぶりの過程で形成される病原因子、(2) 感染を伴う熱傷（やけど）。
濕毒		1.6.52	dampness toxin	noxious pathogenic factor formed by stagnation of dampness, which may cause hematochezia when it occurs in the intestine, or ulcer of the shank when in the muscles and skin of the lower limbs	湿邪の停滞によって形成される有毒の病原因子。腸で発生した場合には血便を、筋肉および下肢で発生した場合には脚の潰瘍を引き起こす場合がある。
寒毒		1.6.53	cold toxin	a cold pathogen with virulent coldness	有毒な寒さを備えた寒邪。
時毒		1.6.54	seasonal toxin	(1) virulent pathogens prevalent in a particular season or seasons; (2) painful swelling in the neck, cheek and jaw, caused by epidemic seasonal pathogens which invades the three yang meridians	(1) 有毒な病邪で、特定の季節に流行する、(2) 三陽経が伝染性の季節性病邪に侵されることで生じる、痛みを伴う頸部、頬、顎の腫脹。
痲毒		1.6.55	measles toxin	the pathogen that causes measles	麻疹を引き起こす病邪。
內毒		1.6.56	internal toxin	heat toxins dormant in the body	体内に潜伏する熱毒。
胎毒		1.6.57	fetal toxin	(1) toxic heat affecting the fetus; (2) any congenital pathogenic factor; also known as fetal toxicosis	(1) 胎児に悪影響を及ぼす有毒な熱、(2) 先天性の病原因子、fetal toxicosisとしても知られる。
蠱毒		1.6.58	parasitic toxin	a pathogenic factor that may cause diseases marked by abdominal lumps, tympanites and ascites, also known as worm toxin	腹部の腫瘤、膨張、および腹水を特徴とする疾患を引き起こす可能性のある病原因子。worm toxinとしても知られる。
瘴毒	瘴毒; 瘴氣; 山嵐瘴氣	1.6.59	miasmic toxin	noxious mountainous vapor alleged to be the cause of certain kinds of malaria, also known as miasma	特定のタイプのマラリアを引き起こすと思われる、有毒な山の気。miasmaとしても知られる。
瘴氣	瘴毒; 瘴氣; 山嵐瘴氣	1.6.59	miasmic toxin	noxious mountainous vapor alleged to be the cause of certain kinds of malaria, also known as miasma	特定のタイプのマラリアを引き起こすと思われる、有毒な山の気。miasmaとしても知られる。
山嵐瘴氣	瘴毒; 瘴氣; 山嵐瘴氣	1.6.59	miasmic toxin	noxious mountainous vapor alleged to be the cause of certain kinds of malaria, also known as miasma	特定のタイプのマラリアを引き起こすと思われる、有毒な山の気。miasmaとしても知られる。
惡氣		1.6.60	malign qi	(1) a general term for the pathogenic qi, including six excesses and pestilential factors; (2) a pathological product derived from stagnation of qi and blood; also called evil qi	(1) 六淫および悪疫性の因子を含む邪気の一般名、(2) 気および血の停滞に起因する病的産物。evil qiとも呼ばれる。
內傷		1.6.61	internal damage	any detrimental effect on visceral qi produced by intemperance of the seven emotions, overexertion and fatigue, improper diet and sexual overindulgence	臓気に対する有害な影響。過度の七情、老倦、不適切な食事および房労によって形成される。
五志		1.6.62	five minds	a collective term for joy, anger, thought, anxiety, and fear, the same as five emotions	喜、怒、思、憂、恐の総称。five emotionsと同義。
五志過極		1.6.63	excess among the five minds	excessive anger, joy, grief, thought and fear, which may disturb the normal flow of qi and blood of the internal organs, the same as five excessive emotions	過度の怒、喜、悲、思、恐。内臓の気および血の正常な流れを妨げる場合がある。five excessive emotionsと同義。
五志化火		1.6.64	transformation of the five minds into fire	transformation of the five emotions (anger, joy, grief, thought and fear) into heat with fire symptoms, the same as transformation of the five emotions into fire	五志（怒、喜、悲、思、恐）の熱邪への転換で、火証を伴う。transformation of the five emotions into fireと同義。

検索表示（原表示の複数語を分離）	IST原表示	Code	Term	Definition/Description	定義/記述
七情		1.6.65	seven emotions	a collective term for joy, anger, thought, anxiety, sorrow, fear and fright, taken as endogenous factors causing diseases if in excess	喜、怒、思、憂、悲、恐、驚の総称で、過剰な場合に疾患を引き起こす内因性因子とされる。
喜		1.6.66	joy	one of the seven emotions that in excess may make the heart qi sluggish, resulting in absentmindedness, palpitations, insomnia and even mental disturbance	七情の1つで、過剰になると心気の停滞が起こることがあり、放心、動悸、不眠症、さらには精神障害が生じる。
怒		1.6.67	anger	one of the seven emotions that in excess may cause the liver qi to ascend together with blood, resulting in headache, flushed face, blood-shot eyes, or hematemesis, even sudden fainting	七情の1つで、過剰になると腎気が血と共に上昇することがあり、頭痛、顔面紅潮、眼の充血あるいは吐血、さらには突然の気絶が生じる。
憂		1.6.68	anxiety	one of the seven emotions that in excess may cause damage to the lung, and in combination with thought may injure the spleen	七情の1つで、過剰になると肺を害することがある。思と組み合わさると脾臓を損なうことがある。
思		1.6.69	thought	one of the seven emotions that in excess may cause stagnation of the spleen qi, and even damage to the transporting and transforming function of the spleen	七情の1つで、過剰になると脾気の停滞を起こし、さらには脾臓の運化機能を害することがある。
悲		1.6.70	sorrow	one of the seven emotions that in excess may consume the lung qi, resulting in shortness of breath, listlessness and fatigue	七情の1つで、過剰になると肺気を消耗することがあり、息切れ、倦怠、および疲労が生じる。
恐		1.6.71	fear	one of the seven emotions that in excess may cause the kidney qi to sink, resulting in incontinence of urine and stools, or even syncope	七情の1つで、過剰になると腎気が低下することがあり、尿および便失禁、さらには失神を生じる。
驚		1.6.72	fright	one of the seven emotions that occurs suddenly, may disturb the heart qi, resulting in palpitations or mental confusion	七情の1つで、突然に起こる。心気が乱れることがあり、動悸あるいは精神錯乱が生じる。
勞倦		1.6.73	overexertion and fatigue	abnormal degree of fatigue brought about by excessive activity, the same as overstrain	過度な活動による異常な疲労。overstrainと同義。
房勞		1.6.74	sexual overindulgence	exhaustion due to sexual overindulgence	性的耽溺に起因する極度の疲労。
五味偏嗜		1.6.75	flavor predilection	habitual preference for a particular flavor or taste that may give rise to disease, the same as flavor craving/preference	特定の風味または味に対する習慣性の嗜好で、疾患を引き起こすことがある。flavor craving/ preferenceと同義。
飲食不節		1.6.76	dietary irregularities	diet harmful to health, including ingestion of raw, cold or contaminated food, voracious eating or excessive hunger, predilection for a special food, alcohol addiction, etc.	健康に有害な食生活で、生、冷たい、あるいは汚染された食物の摂取、暴食または極度の空腹、偏食、およびアルコール依存症を含む。
酒癖		1.6.77	liquor addiction	the state of heavy dependence on alcohol	アルコールへの過度の依存状態。
水土不服		1.6.78	failure to acclimatize to a new environment	temporary inadaptability of a person to a new natural and living environment, the same as non-acclimatization	新しい自然環境および生活環境への一時的な不適応。non-acclimatizationと同義。
痰飲		1.6.79	phlegm-retained fluid	a combination of phlegm and retained fluid as an entity to cause disease, also called phlegm-fluid retention	痰と水飲が組み合わさったもので、疾患を引き起こす。phlegm-fluid retentionとも呼ばれる。
痰		1.6.80	phlegm	(1) pathologic secretions of the diseased respiratory tract, which is known as sputum; (2) the viscous turbid pathological product that can accumulate in the body, causing a variety of diseases	(1) 病変した気道からの病的な分泌物で、sputumとして知られる、(2) 身体に蓄積し、さまざまな疾患を引き起こす粘性の不透明な病的産物。
飲	飲; 水飲	1.6.81	retained fluid	the clear and watery pathological product due to disordered fluid metabolism, also called fluid retention	異常な水代謝に起因する透明な水様の病的産物。fluid retentionとも呼ばれる。
水飲	飲; 水飲	1.6.81	retained fluid	the clear and watery pathological product due to disordered fluid metabolism, also called fluid retention	異常な水代謝に起因する透明な水様の病的産物。fluid retentionとも呼ばれる。
水濕		1.6.82	water-dampness	any water or dampness as an entity to cause disease	病因としての水あるいは湿。
痰濕		1.6.83	phlegm-dampness	a combination of phlegm and internal dampness as an entity to cause disease, the same as dampness-phlegm	痰と内湿が組み合わさったもので、疾患を引き起こす。dampness-phlegmと同義。
瘀血		1.6.84	static blood	a pathological product of blood stagnation, including extravasated blood and the blood circulating sluggishly or blood congested in a viscus, all of which may turn into pathogenic factor, the same as blood stasis or stagnant blood	血の停滞による病的産物で、病原因子となり得る溢血および血流の低下または内臓の鬱血を含む。blood stasisまたはstagnant bloodと同義。
稟賦不足		1.6.85	constitutional insufficiency	congenital constitutional weakness as the main etiological factor of deficiency conditions	先天的な虚弱体質で、虚状態の主な病因要素。
三因		1.6.86	three causes	external cause, internal cause, and cause neither internal nor external - an ancient classification of causes of disease, also called three categories of causes of disease	古来からの疾患原因の分類（外因、内因、不内外因）。three categories of causes of disease とも呼ばれる。
內因		1.6.87	internal cause	one of the three causes of disease, one that arises within the body, referring chiefly to the excessive emotional changes	三因の1つで、体内で生じる原因。主に過剰な情動的変化を指す。
外因		1.6.88	external cause	one of the three causes of disease, one that originates outside the body, referring chiefly to the six excesses and pestilential pathogens	三因の1つで、体外で生じる原因。主に六淫と悪疫性の病原因子を指す。
不內外因		1.6.89	cause neither internal nor external	one of the three causes of disease, including dietary irregularities, overexertion and fatigue, traumatic injuries, and insect and animal bites	三因の1つで、不規則な食生活、過度の活動と疲労、外傷および虫さされや動物咬傷を含む。
病機		**1.7.0**	**Mechanism of Disease**		
病機		1.7.1	mechanism of disease	the mechanism by which disease arises and develops, the same as pathogenesis	疾患の発生および進行機序。pathogenesisと同義。
病機學說		1.7.2	theory of mechanism of disease	the theory that deals with the mechanism by which disease arises and develops	疾患の発生および進行機序に関する理論。
病位		1.7.3	location of disease	the part of the body affected by a disease	病気に冒された身体の部位。
病性		1.7.4	nature of disease	the heat, cold, excess or deficiency quality of a disease	疾患の熱、寒、実、虚の特性。
病證		1.7.5	disease pattern	summarization of the cause, nature and location of the pathological change at a certain stages of disease	特定の病期における原因、病性、および病位の要約。
正邪相爭		1.7.6	struggle betweenthe healthy qi and pathogenic qi	the basic mechanism of disease in which any disease is considered as the process of struggle between the healthy qi and pathogenic qi	疾患の基本的機序。あらゆる疾患は、正気と病邪（邪気）による争いの過程であると考えられている。

検索表示（原表示の複数語を分離）	IST原表示	Code	Term	Definition/Description	定義/記述
邪正盛衰	邪正盛衰; 邪正消長	1.7.7	exuberance and debilitation of the healthy qi or pathogenic qi	the key factor that determines the process of disease and prognosis, namely, rise of the healthy qi with decline of the pathogenic qi leading to improvement and cure, while exuberance of the pathogenic qi with debilitation of the healthy qi resulting in deterioration and even death	疾患の過程と予後を決定する主要な要因。正気の力が病邪（邪気）を上回れば回復および治癒に向かい、逆に病邪の力が正気を上回ると悪化、さらには死に至る。
邪正消長	邪正盛衰; 邪正消長	1.7.7	exuberance and debilitation of the healthy qi or pathogenic qi	the key factor that determines the process of disease and prognosis, namely, rise of the healthy qi with decline of the pathogenic qi leading to improvement and cure, while exuberance of the pathogenic qi with debilitation of the healthy qi resulting in deterioration and even death	疾患の過程と予後を決定する主要な要因。正気の力が病邪（邪気）を上回れば回復および治癒に向かい、逆に病邪の力が正気を上回ると悪化、さらには死に至る。
卒發		1.7.8	sudden onset	the rapid appearance of symptoms after contraction	罹病後の急速な症状発現。
徐發		1.7.9	gradual onset	the gradual appearance of symptoms after contraction	罹病後の漸次的な症状発現。
勞復		1.7.10	taxation relapse	relapse of disease due to over-fatigue	過労による疾患の再発。
食復		1.7.11	relapse due to dietary irregularity	relapse of disease due to improper diet	不適切な食生活による疾患の再発。
女勞復		1.7.12	sexual taxation relapse	relapse of disease due to intemperance in sexual activity	過度の性的行為による疾患の再発。
陰陽離決		1.7.13	dissociation of yin and yang	divorce of yin and yang, indicating the end of life	陰と陽の分離。命の終わりを指す。
陰陽失調	陰陽失調; 陰陽不和	1.7.14	yin-yang disharmony	a general term for all kinds of pathological changes due to imbalance and incoordination of yin and yang	陰と陽の不均衡および不調整に起因するあらゆる病的変化を指す一般用語。
陰陽不和	陰陽失調; 陰陽不和	1.7.14	yin-yang disharmony	a general term for all kinds of pathological changes due to imbalance and incoordination of yin and yang	陰と陽の不均衡および不調整に起因するあらゆる病的変化を指す一般用語。
陰陽偏盛		1.7.15	abnormal exuberance of yin or yang	any pathological change marked by yin or yang higher than the normal level due to yin pathogens or yang pathogens	陰邪または陽邪によって正常レベルよりも高くなった陰または陽を特徴とする病的変化。
陰陽偏衰		1.7.16	abnormal debilitation of yin or yang	any pathological change marked by yin or yang lower than the normal level due to deficiency of yin or yang of the human body	陰邪または陽邪によって正常レベルよりも低くなった陰虚または陽虚を特徴とする病的変化。
陰陽自和		1.7.17	spontaneous harmonization of yin and yang	spontaneous recovery from imbalance of yin and yang by its own natural regulatory function, also known as spontaneous restoration of yin-yang equilibrium	生得の制御機能による陰陽の不均衡の自然な回復。spontaneous restoration of yin-yang equilibriumとしても知られる。
傷陽		1.7.18	damage to yang	a general term for various kinds of pathological changes marked by impairment of yang qi	陽気の障害を特徴とするあらゆる病的変化を指す一般用語。
傷陰		1.7.19	damage to yin	a general term for various kinds of pathological changes marked by consumption of yin qi	陰気の障害を特徴とするあらゆる病的変化を指す一般用語。
陽損及陰		1.7.20	detriment to yang affects yin	a pathological change in which weakness of yang qi impedes the generation of yin, resulting in deficiency of both yin and yang with a preponderance of yang deficiency	陽気の衰弱によって陰の生成が阻害される病的変化。陰虚および陽虚に至るが、陽虚が優勢。
陰損及陽		1.7.21	detriment to yin affects yang	a pathological change in which consumption of yin weakens yang qi, resulting in deficiency of both yin and yang with a preponderance of yin deficiency	陰の消耗によって陽気が衰弱する病的変化。陰虚および陽虚に至るが、陰虚が優勢。
陰虛		1.7.22	yin deficiency	a pathological change marked by deficiency of yin with diminished moistening, calming, downbearing and yang-inhibiting function, leading to relative hyperactivity of yang qi; in Kampo medicine, "yin" and "deficiency" are independently understood	陰の不足を特徴とする病的変化で、滋潤、沈静化、降濁および陽抑制機能の低下を伴う。陽気の相対的な活動亢進に至る。漢方医学では、「陰」と「虚」が個別に解釈される。
陰虛內熱		1.7.23	yin deficiency with internal heat	insufficient yin failing to counterbalance yang, causing deficiency-heat to arise internally	陰の不足によって陽との釣り合いが取れなくなり、体内で虚熱が生じる。
陰虛陽亢		1.7.24	yin deficiency with yang hyperactivity	insufficient essence, blood and fluid failing to restrain yang, causing increased activity of yang	精、血および津の不足により陽を抑制できなくなり、陽の活動亢進が生じる。
陰虛火旺		1.7.25	yin deficiency with effulgent fire	insufficient yin failing to restrain yang, causing exuberant fire of the deficiency type	陰の不足によって陽を抑制できなくなり、陰の火旺が生じる。
虛火		1.7.26	deficiency fire	consumption of yin fluid failing to restrain yang fire, giving rise to fire of the deficiency type	陰津の消耗によって陽火を抑制できなくなり、虚火が生じる。
虛火上炎		1.7.27	deficiency fire flaming upward	a pathological change occurring in yin deficiency which is unable to inhibit yang, causing upflaming of the deficiency fire	陰虚で生じる病的変化で、陽を抑制できず、虚火の炎上を生じる。
相火妄動		1.7.28	frenetic stirring of the ministerial fire	deficiency of liver-kidney yin leading to hyperactivity and upsurge of ministerial fire	肝腎陰虚により、相火の活動亢進と増大が生じる。
陰盛		1.7.29	yin exuberance	a pathological state in which yin pathogen is exuberant while the healthy qi has not been damaged, giving rise to an excess-cold syndrome, the same as yin excess	正気は害されていないながら陰邪が過盛な病的状態。実寒証を生じる。yin excessと同義。
陰盛陽衰		1.7.30	yin exuberance with yang debilitation	exuberant yin cold with resultant debilitation of yang qi, the same as yin excess with yang deficiency	過盛な陰寒とその結果生じる陽気の衰弱。yin excess with yang deficiencyと同義。
陽虛		1.7.31	yang deficiency	a pathological state characterized by deficiency of body's yang qi that leads to diminished functions, decreased metabolic activities, reduced body reactions as well as deficiency-cold manifestations; in Kampo medicine, "yang" and "excess" are independently understood	身体の陽気不足を特徴とする病的状態で、機能、代謝活性および身体反応の低下、ならびに虚寒証の発現に至る。漢方医学では、「陽」と「実」が個別に解釈される。
陽虛陰盛		1.7.32	yang deficiency with yin exuberance	insufficient yang failing to counterbalance yin, resulting in relative exuberance of yin, the same as yang deficiency with yin excess	陽の不足によって陰との釣り合いが取れなくなり、陰の相対的な過盛に至る。yang deficiency with yin excessと同義。
陽盛		1.7.33	yang exuberance	a pathological state in which yang is exuberant while yin has not been debilitated, giving rise to an excess-heat syndrome, the same as yang excess	陰は衰弱していないながら陽が過盛な病的状態。実熱証を生じる。yang excessと同義。
陽盛陰衰		1.7.34	yang exuberance with yin debilitation	a pathological state characterized by simultaneous presence of exuberance of yang and debilitation of yin, also the same as yang excess with yin deficiency	陽盛と陰の衰弱が同時に存在する病的状態。yang excess with yin deficiencyと同義。

検索表示（原表示の複数語を分離）	IST原表示	Code	Term	Definition/Description	定義/記述
陰陽格拒		1.7.35	yin-yang repulsion	a serious pathological state in which extremely excessive yin in the interior forces the asthenic yang to spread outward or extremely exuberant yang in the interior keeps insufficient yin on the outside, forming pseudo-heat or pseudo-cold phenomena	体内の極度に過剰な陰が衰弱した陽を体外へと拡散させる、あるいは体内の極度に過盛な陽が不足している陰を体外へ追い出す重篤な病的状態。仮熱または仮寒の症状を生じる。
陰盛格陽	陰盛格陽; 格陽	1.7.36	exuberant yin repelling yang	a pathological state in which extremely excessive yin entrenched in the exterior forces the asthenic yang to float on the body surface, leading to pseudo-heat symptoms, the same as excessive yin repelling yang, and also known as repelled yang	体外の極度に過剰な陰が衰弱した陽を体表で浮遊させる病的状態。仮熱の症状を生じる。excessive yin repelling yangと同義。repelled yangとしても知られる。
格陽	陰盛格陽; 格陽	1.7.36	exuberant yin repelling yang	a pathological state in which extremely excessive yin entrenched in the exterior forces the asthenic yang to float on the body surface, leading to pseudo-heat symptoms, the same as excessive yin repelling yang, and also known as repelled yang	体外の極度に過剰な陰が衰弱した陽を体表で浮遊させる病的状態。仮熱の症状を生じる。excessive yin repelling yangと同義。repelled yangとしても知られる。
陽盛格陰	陽盛格陰; 格陰	1.7.37	exuberant yang repelling yin	a pathological state in which extremely exuberant yang trapped in the interior keeps insufficient yin in the exterior, leading to pseudo-cold symptoms, the same as excessive yang repelling yin, and also known as repelled yin	体外の極度に過剰な陰が衰弱した陽を体表で浮遊させる病的状態。仮熱の症状を生じる。excessive yang repelling yinと同義。repelled yinとしても知られる。
格陰	陽盛格陰; 格陰	1.7.37	exuberant yang repelling yin	a pathological state in which extremely exuberant yang trapped in the interior keeps insufficient yin in the exterior, leading to pseudo-cold symptoms, the same as excessive yang repelling yin, and also known as repelled yin	体内の極度に過盛な陽が不足している陰を体外に閉め出す病的状態。仮寒の症候を生じる。excessive yang repelling yinと同義。repelled yinとしても知られる。
戴陽		1.7.38	upcast yang	a pathological state characterized by exuberant yin cold in the lower part of the body which forces the asthenic yang to stay at the upper surface of the body	下半身の過盛な陰が衰弱した陽を上半身表面に追いやる病的状態。
陰陽兩虛		1.7.39	dual deficiency of yin and yang	a pathological state characterized by deficiency of both yin and yang	陰陽双方の不足を特徴とする病的状態。
虛陽上浮	虛陽上浮; 孤陽上越	1.7.40	deficiency yang floating upward	a pathological change in which consumption of essence and blood deprives yang of its base, causing yang to float at the upper surface	精と血の消耗により陽がその基盤を失い、陽が上半身表面を浮遊する病的状態。
孤陽上越	虛陽上浮; 孤陽上越	1.7.40	deficiency yang floating upward	a pathological change in which consumption of essence and blood deprives yang of its base, causing yang to float at the upper surface	精と血の消耗により陽がその基盤を失い、陽が上半身表面を浮遊する病的状態。
亡陰	亡陰; 脫陰; 陰脫	1.7.41	yin collapse	a pathological change caused by sudden massive loss of fluid leading to collapse	突然の津の著しい喪失によって生じる病的変化で、虚脱状態に至る。
脫陰	亡陰; 脫陰; 陰脫	1.7.41	yin collapse	a pathological change caused by sudden massive loss of fluid leading to collapse	突然の津の著しい喪失によって生じる病的変化で、虚脱状態に至る。
陰脫	亡陰; 脫陰; 陰脫	1.7.41	yin collapse	a pathological change caused by sudden massive loss of fluid leading to collapse	突然の津の著しい喪失によって生じる病的変化で、虚脱状態に至る。
亡陽	亡陽; 脫陽; 陽脫	1.7.42	yang collapse	a pathological change where yang qi is suddenly exhausted, resulting in abrupt failure of bodily functions	陽気が突然失われる病的変化で、急激な身体機能不全に至る。
脫陽	亡陽; 脫陽; 陽脫	1.7.42	yang collapse	a pathological change where yang qi is suddenly exhausted, resulting in abrupt failure of bodily functions	陽気が突然失われる病的変化で、急激な身体機能不全に至る。
陽脫	亡陽; 脫陽; 陽脫	1.7.42	yang collapse	a pathological change where yang qi is suddenly exhausted, resulting in abrupt failure of bodily functions	陽気が突然失われる病的変化で、急激な身体機能不全に至る。
陽亡陰竭		1.7.43	collapse of yang and exhaustion of yin	collapse of both yin and yang, indicating the moribund state of a critically ill patient	陰陽双方の衰弱。重篤患者の瀕死状態を指す。
陰竭陽脫		1.7.44	exhaustion of yin and collapse of yang	exhaustion of yin fluid and collapse of yang qi with resultant functional failure, indicating that the patient is critically ill	陰津の完全な消耗および陽気の衰弱と、その結果生じる機能不全。患者が重篤な状態にあることを示す。
內閉外脫		1.7.45	internal block and external collapse	a pathological change characterized by depression/stagnation of excessive pathogenic qi in the interior with expulsion of the debilitated original qi	体内の過剰な邪気の減退／停滞を特徴とする病的変化で、衰弱した原気の排除を伴う。
結陰		1.7.46	binding in yin	pathogenic qi binding in the yin meridians/channels.	陰経絡に結合する邪気。
結陽		1.7.47	binding in yang	sluggish flow of yang qi in the limbs, leading to retention of water and edema	四肢における陽気の流れの停滞で、水分貯留および浮腫に至る。
表裏		1.7.48	exterior and interior	(1) the outer part of the body (the skin, body hair, flesh and superficial meridians/channels) and the inner part of the body (the bowels and viscera, qi, blood, and bone marrow); (2) two of the eight principles of pattern identification/syndrome differentiation, indicating the depth of penetration of external pathogens	(1) 身体の外部（皮膚、体毛、肌肉および体表の経絡）および内部（臟腑、気、血、および骨髄）、(2) 証診断の八綱の2つで、外部病邪の侵入深さを指す。
半表半裏		1.7.49	half-exterior half-interior	location between the exterior and the interior	表裏の間の位置。
表寒		1.7.50	exterior cold	attack on the exterior part of the body by wind-cold, chiefly manifested by severe aversion to wind and cold, headache and stiff neck, soreness of the limbs and joints, thin white coating of the tongue and floating tense pulse	風寒による身体外部の攻撃で、主に風および寒に対する高度な抵抗反応、頭痛、斜頸、四肢および関節の痛み、舌の苔白および変動性の緊張脈などの症状が発現する。
表熱		1.7.51	exterior heat	attack on the exterior part of the body by wind-heat, chiefly manifested by slight aversion to wind and cold, moderate fever, headache, slight thirst, thin white or thin yellowish coating of the tongue, or red tip of the tongue and floating rapid pulse	風熱による身体外部の攻撃で、主に風および寒に対する軽微な抵抗反応、中等度の発熱、頭痛、軽微な口渇、舌の白苔または黄苔、舌先端部の赤らみ、および変動性の頻脈などの症状が発現する。
表虛		1.7.52	exterior deficiency	deficiency of defense qi in the superficial part of the body, marked by spontaneous sweating or sweating accompanied by aversion to wind and floating feeble pulse	体表部における衛気虚。自然発汗または風に対する抵抗反応に付随して起こる発汗、および変動性の微弱な頻脈を特徴とする。

検索表示（原表示の複数語を分離）	IST原表示	Code	Term	Definition/Description	定義/記述
表實		1.7.53	exterior excess	invasion of external pathogens that causes gathering of defense qi in the skin and flesh and blockage of the interstices and pores marked by absence of sweating, chills and a floating forceful pulse	外部からの病邪の侵入。肌肉での衛気の集合および無発汗によって特徴づけられる毛穴の閉鎖、悪寒および変動性の実脈を引き起こす。
裏寒		1.7.54	interior cold	a pathological state characterized by preponderance of yin cold or decline of yang qi in the interior	体内での陰寒の優勢または陽気の減退を特徴とする病的状態。
裏熱		1.7.55	interior heat	a pathological state characterized by heat in the interior, either due to exuberance of pathogenic heat or due to yin deficiency with endogenous heat	体内の熱（熱邪の過盛または内熱を伴う陰虚による）を特徴とする病的状態。
裏虛		1.7.56	interior deficiency	a general term for deficiency of qi, blood, yin and yang of the internal organs	内臓の気、血、陰および陽の不足を指す一般用語。
裏實		1.7.57	interior excess	(1) a pathological change resulting from an external pathogen transforming into heat and entering the interior to bind in the stomach and intestines; (2) a general term denoting accumulation of pathological products in the body, such as phlegm, retained fluid, stagnant qi and blood, intestinal parasites, and undigested food	(1) 外部病邪の熱への転換と、体内への侵入および胃腸への結合によって生じる病的変化、(2) 痰、水飲、気および血の停滞、腸内寄生虫および未消化の食物など、病的産物の蓄積を指す一般用語。
表寒裏熱		1.7.58	exterior cold and interior heat	a pathological state characterized by the simultaneous presence of exterior cold and interior heat	表寒と裏熱の同時存在を特徴とする病的状態。
表熱裏寒		1.7.59	exterior heat and interior cold	a pathological state characterized by the simultaneous presence of exterior heat and interior cold	表熱と裏寒の同時存在を特徴とする病的状態。
表虛裏實		1.7.60	exterior deficiency and interior excess	a pathological state characterized by the simultaneous presence of exterior deficiency and interior excess	表虚と裏実の同時存在を特徴とする病的状態。
表實裏虛		1.7.61	exterior excess and interior deficiency	a pathological state characterized by the simultaneous presence of exterior excess and interior deficiency.	表実と裏虚の同時存在を特徴とする病的状態。
表裏俱寒		1.7.62	cold in both exterior and interior	a pathological state characterized by the simultaneous presence of exterior cold and interior cold	表寒と裏寒の同時存在を特徴とする病的状態。
表裏俱熱		1.7.63	heat in both exterior and interior	a pathological state characterized by the simultaneous presence of exterior heat and interior heat	表熱と裏熱の同時存在を特徴とする病的状態。
表裏俱實		1.7.64	dual excess of the exterior and interior	a pathological state characterized by the simultaneous presence of exterior excess and interior excess	表実と裏実の同時存在を特徴とする病的状態。
表裏俱虛		1.7.65	dual deficiency of the exterior and interior	a pathological state characterized by the simultaneous presence of exterior deficiency and interior deficiency	表虚と裏虚の同時存在を特徴とする病的状態。
表裏同病		1.7.66	dual disease of the exterior and interior	coexistence of diseases both in the exterior and interior	表裏双方における疾患の存在。
表氣不固	表氣不固; 衛氣不固	1.7.67	insecurity of exterior qi	failure of insufficient defense qi to protect the superficies of the body against external pathogens, the same as insecurity of defense qi	衛気の不足による、外部病邪からの体表保護不能。insecurity of defense qi と同義。
衛氣不固	表氣不固; 衛氣不固	1.7.67	insecurity of exterior qi	failure of insufficient defense qi to protect the superficies of the body against external pathogens, the same as insecurity of defense qi	衛気の不足による、外部病邪からの体表保護不能。insecurity of defense qi と同義。
表邪內陷		1.7.68	inward invasion of exterior pathogen	penetration of an externally contracted pathogen from the exterior into the interior	外部病邪の体内への侵入。
熱邪傳裏		1.7.69	pathogenic heat passing into the interior	process of a disease where external heat pathogens enter the interior to cause an interior heat pattern/syndrome	外部の熱邪が体内へ侵入し、内熱証を引き起こす疾患経過。
表邪入裏		1.7.70	exterior pathogen entering the interior	the process of a disease whereby the external pathogen attacks the exterior first, and then enters the interior, impairing visceral functions	外部病邪がまず外部を攻撃し、その後体内に侵入し、内臓機能を害する疾患経過。
裏病出表		1.7.71	interior disease moving out to the exterior	the process of a disease turning from the interior to the exterior through the struggle of the healthy qi against pathogens	正気と病邪の争いを経て、疾患が体内から体外へと現れる経過。
寒熱		1.7.72	cold and heat	(1) a pair of principles for differentiating the nature of disease: when yin is exuberant, there is cold; when yang prevails, there is heat; (2) chills and fever	(1) 疾患の性質を識別するための1対の原則（陰が過盛になると寒になり、陽が優性になると熱になる）、(2) 悪寒および発熱。
實寒		1.7.73	excess cold	a pathological change arising from contraction of yin cold pathogen	陰寒の病邪に侵されたことによって生じる病的変化。
虛寒		1.7.74	deficiency cold	a pathological change arising when yang qi becomes insufficient and fails to provide adequate warmth	陽気が不足し、十分な暖が得られない場合に生じる病的変化。
熱結下焦		1.7.75	heat binding in the lower energizer	accumulation of heat in the intestines and bladder that causes lower abdominal distention and pain, constipation, short voidings of dark urine or even hematuria, and sometimes rigidity and tenderness of the lower abdomen accompanied by restlessness	腸および膀胱における熱の蓄積で、下腹部の膨満および痛み、便秘、少量の暗色尿さらには血尿、および時に絶え間ない動きを伴う下腹部の硬直と圧痛を引き起こす。
下焦濕熱<br>濕熱下注		1.7.76	lower energizer dampness-heat	a pathological change characterized by dampness-heat pouring down into the lower energizer, including large intestinal dampness-heat, bladder dampness-heat, dampness-heat obstructing the essence chamber, and morbid leukorrhea, pudental itch, and painful swelling of the lower limb joints caused by dampness-heat, the same as downward flow of dampness-heat	下焦への湿熱注入を特徴とする病的変化。大腸湿熱、膀胱湿熱、湿熱による精室のつまり、病的な帯下、陰部掻痒、湿熱に起因する疼痛を伴う下肢関節の腫脹を含む。downward flow of dampness-heatと同義。
熱盛傷津		1.7.77	exuberant heat damaging fluid	the process by which excess heat dissipates body fluids, also the same as excessive heat damaging fluid	実熱が体液を浪費する過程。excessive heat damaging fluidと同義。
寒包火		1.7.78	cold enveloping fire	a pathological change characterized by contraction of wind-cold with accumulated internal heat	風寒と蓄積した内熱の結合を特徴とする病的変化。
寒熱錯雜		1.7.79	cold-heat complex	a pathological change that causes complicated heat and cold conditions such as heat in the upper body with cold in the lower body, and cold in the exterior and heat in the interior	複雑な熱寒の状態（上半身に熱、下半身に寒、および外部は寒、内部は熱など）によって生じる病的変化。

検索表示（原表示の複数語を分離）	IST原表示	Code	Term	Definition/Description	定義/記述
上熱下寒		1.7.80	upper body heat and lower body cold	a complex condition characterized by the simultaneous presence of heat in the upper body and cold in the lower body, the same as heat above and cold below	上半身に熱、下半身に寒が同時存在する複雑な状態。heat above and cold belowと同義。
上寒下熱		1.7.81	upper body cold and lower body heat	a complex condition characterized by the simultaneous presence of cold in the upper body and heat in the lower body, the same as cold above and heat below	上半身に寒、下半身に熱が同時存在する複雑な状態。cold above and heat belowと同義。
寒熱格拒		1.7.82	cold and heat repulsion	a form of pathological change in which extremely excessive cold forces heat to the extremities, and vice versa	極めて過剰な寒／熱が熱／寒を四肢へと追いやる病的変化の一種。
眞寒假熱		1.7.83	true cold with false heat	a pathological change marked by abundant yin-cold in the interior with pseudo-heat manifestations	体内での陰寒の増大を特徴とする病的変化で、仮熱の症状を伴う。
眞熱假寒		1.7.84	true heat with false cold	a pathological change marked by excessive heat in the interior with pseudo-cold manifestations	体内での過剰な熱を特徴とする病的変化で、仮寒の症状を伴う。
虛實		1.7.85	deficiency and excess	one of the guiding principles for analyzing the condition of the body's resistance to pathogenic factors, in which deficiency refers to deficiency of the healthy qi and excess refers to excessiveness of the pathogenic qi	病原因子に対する身体の抵抗力の状態を調べる際の指針の1つ。虚は正気の不足を、実は邪気の過剰を指す。
虛		1.7.86	deficiency	(1) deficiency of the healthy qi; (2) weak constitution; (3) weak reaction against pathogens	(1) 正気の不足、(2) 虚弱体質、(3) 病邪に対する弱い反応。
實		1.7.87	excess	(1) excessiveness of the pathogenic qi; (2) strong constitution; (3) strong reaction against pathogens	(1) 邪気の過剰、(2) 丈夫な体質 (3) 病邪に対する強い反応
虛實夾雜		1.7.88	deficiency-excess complex	a pathological state in which both excess of pathogenic qi and debilitation of healthy qi occur in the disease process	疾患経過において邪気の過剰と正気の衰弱双方が起こる病的状態。
實中夾虛		1.7.89	excess with deficiency complication	excess condition complicated by deficiency symptoms with the former dominant	実が優勢な状況下での、虚の症状を合併した実状態。
虛中夾實		1.7.90	deficiency with excess complication	deficiency condition complicated by excess symptoms with the former dominant	虚が優勢な状況下での、実の症状を合併した虚状態。
上虛下實		1.7.91	upper deficiency and lower excess	deficiency of healthy qi in the upper body and excess of pathogenic qi in the lower body, the same as deficiency above and excess below	上半身における正気の不足および下半身における邪気の過剰。deficiency above and excess belowと同義。
上盛下虛	上盛下虛; 上實下虛	1.7.92	upper excess and lower deficiency	exuberance of pathogenic qi in the upper body and deficiency of healthy qi in the lower body, the same as excess above and deficiency below	上半身における邪気の過盛および下半身における正気の不足。excess above and deficiency belowと同義。
上實下虛	上盛下虛; 上實下虛	1.7.92	upper excess and lower deficiency	exuberance of pathogenic qi in the upper body and deficiency of healthy qi in the lower body, the same as excess above and deficiency below	上半身における邪気の過盛および下半身における正気の不足。excess above and deficiency belowと同義。
虛實眞假		1.7.93	true or false deficiency-excess	false appearance of deficiency-excess manifestations opposite to the true nature of the disease	疾患本来の性質とは逆となる、虚実の仮象の発現。
眞實假虛		1.7.94	true excess with false deficiency	excess pattern/syndrome with pseudo-deficiency symptoms	仮虚象を伴う実証。
眞虛假實		1.7.95	true deficiency with false excess	deficiency pattern/syndrome with pseudo-excess symptoms	仮実象を伴う虚証。
由實轉虛		1.7.96	conversion of excess into deficiency	the process of a disease turning from excess of pathogenic qi into deficiency of healthy qi	邪気の過剰から正気の不足へと変化する疾患過程。
由虛轉實		1.7.97	conversion of deficiency into excess	the process of a disease turning from deficiency of healthy qi into excess of pathogenic qi	正気の不足から邪気の過剰へと変化する疾患過程。
氣虛		1.7.98	qi deficiency	a general term for deficiency of qi that leads to decreased visceral functions and lowered body resistance	気の不足を指す一般用語で、内臓機能の低下および身体の抵抗力低下に至る。
氣虛不攝	氣虛不攝; 氣不攝血	1.7.99	qi deficiency failing to control blood	a pathological change of qi deficiency in which qi is unable to retain the fluids (including blood)	気虚の病的変化で、気による津（血を含む）の保持が不能となる。
氣不攝血	氣虛不攝; 氣不攝血	1.7.99	qi deficiency failing to control blood	a pathological change of qi deficiency in which qi is unable to retain the fluids (including blood)	気虚の病的変化で、気による津（血を含む）の保持が不能となる。
氣虛中滿		1.7.100	qi deficiency with fullness in the middle	a pathological change of qi deficiency in which qi is insufficient for normal transportation and transformation in the middle energizer, thus causing epigastric and abdominal distension	気虚の病的変化で、気の不足により中焦での正常な運化が不可能となり、上腹部および腹部の膨満を生じる。
氣虛血瘀		1.7.101	qi deficiency with blood stasis	a pathological change of qi deficiency in which qi is insufficient to maintain blood flow, thus resulting in blood stasis	気虚の病的変化で、気の不足により血流の維持が不可能となり、鬱血を生じる。
氣機失調		1.7.102	qi movement disorder	a general term for disordered activity of qi in ascending, descending, exiting and entering	気の活動（昇、降、出、入）の乱れを指す一般用語。
氣機鬱滯	氣機鬱滯; 氣鬱	1.7.103	qi movement stagnation	depressed and stagnant flow of qi that causes dysfunction of internal organs and meridians/channels, the same as qi stagnation	気の流れの低下と停滞。内臓および経絡の機能障害を生じる。qi stagnationと同義。
氣鬱	氣機鬱滯; 氣鬱	1.7.103	qi movement stagnation	depressed and stagnant flow of qi that causes dysfunction of internal organs and meridians/channels, the same as qi stagnation	気の流れの低下と停滞。内臓および経絡の機能障害を生じる。qi stagnationと同義。
氣鬱化火		1.7.104	stagnant qi transforming into fire	a pathological change of long-standing stagnation of qi that transforms into fire	長期にわたる気の停滞が火に変化する病的変化。
氣機不利		1.7.105	inhibited qi movement	impeded, obstructed or stagnant qi movement that impairs the functions of viscera and meridians/channels, the same as qi movement depression	気機の阻害、妨害、停滞による内臓および経絡の機能障害。qi movement depressionと同義。
氣化不利		1.7.106	inhibited qi transformation	a pathological change of yang qi deficiency that decreases digestion and absorption, or impairs fluid metabolism leading to water-dampness retention or phlegm retention	陽虚の病的変化で、消化吸収の低下、または水代謝の低下が生じ、水湿または痰の停滞に至る。
氣逆		1.7.107	qi counterflow	reversal of the normal downward flow of qi, the same as qi reflux	気の正常な下降流とは逆の流れ。qi refluxと同義。

検索表示（原表示の複数語を分離）	IST原表示	Code	Term	Definition/Description	定義/記述
氣陷		1.7.108	qi fall	a pathological change of deficient qi marked by failure in its lifting or holding function, also known as qi sinking	気虚の病的変化で、持ち上げる、あるいは固定機能の不全を特徴とする。qi sinkingとしても知られる。
中氣下陷		1.7.109	sunken middle qi	a pathological change of deficient spleen qi marked by its failure in upbearing the clear and other lifting or holding functions	脾気虚の病的変化で、昇清および他の持ち上げまたは固定機能の不全を特徴とする。
氣滯		1.7.110	qi stagnation	a pathological change characterized by impeded circulation of qi that leads to stagnation of qi movement and functional disorder of organs, manifested as distention or pain in the affected part	気の循環障害を特徴とする病的変化で、気機の停滞および内臓の機能不全に至り、罹患部位で拡張あるいは疼痛が発現する。
寒凝氣滯		1.7.111	qi stagnation due to cold congealing	congealing effect of pathogenic cold that leads to stagnant movement of qi	寒邪の硬直作用で、気機の停滞に至る。
氣閉		1.7.112	qi block	a pathological change marked by obstruction of the normal movement of qi	正常な気機の阻害を特徴とする病的変化。
氣脫		1.7.113	qi collapse	desertion of qi due to sudden damage to the healthy qi, massive loss of blood, profuse sweating, excessive vomiting and diarrhea, or due to prolonged consumption in a chronic disease	気の外脱で、突発的な正気の障害、大量失血、大量発汗、過度の嘔吐および下痢、あるいは慢性疾患における持続的な衰弱によって生じる。
血虛		1.7.114	blood deficiency	any pathological change characterized by deficiency of blood which fails to nourish organs, tissues and meridians/channels	血の不足を特徴とするあらゆる病的変化。内臓、組織および経絡が滋養されない。
血瘀		1.7.115	blood stasis	a morbid state of blood stagnancy in a certain area of the body caused by sluggish flow of qi, deficiency of qi or blood, trauma, or yin-cold	身体の特定部位における血流停滞の病的状態。気の流れの停滞、気虚または血虚、外傷、あるいは陰寒に起因する。
血逆		1.7.116	blood counterflow	a pathological change characterized by reverse flow of blood in the meridian/channel causing separation of qi and blood	経絡における血の逆流を特徴とする病的変化。気と血の分離を生じる。
血熱		1.7.117	blood heat	a pathological change in which exuberant heat or fire enters the blood, usually causing hemorrhage	過盛な熱または火が血に侵入する病的変化。通常、出血を生じる。
血寒		1.7.118	blood cold	a pathological change in which cold either externally contracted or originated from yang deficiency, entering the blood aspect, causes qi stagnation or blood stasis by its congealing effect	体外あるいは陽虚に由来する寒が血分に侵入する病的変化。硬直作用により気滞あるいは血瘀を生じる。
血隨氣逆		1.7.119	blood flowing counterflow with qi	a pathological change in which the upward adverse flow of qi leads blood rushing upward	上方向への気の逆流が上方向への血の急激な流れを生じる病的変化。
亡血	亡血; 血脫	1.7.120	blood collapse	acute critical deficiency of blood, mostly due to massive hemorrhage, also called blood desertion	急性の危険な血虚で、多くの場合大量出血に起因する。blood desertionとも呼ばれる。
血脫	亡血; 血脫	1.7.120	blood collapse	acute critical deficiency of blood, mostly due to massive hemorrhage, also called blood desertion	急性の危険な血虚で、多くの場合大量出血に起因する。blood desertionとも呼ばれる。
氣血失調		1.7.121	disharmony of qi and blood	any failure in the mutually coordinating relationship of qi and blood	気と血の相互協調関係におけるあらゆる障害。
氣滯血瘀		1.7.122	blood stasis due to qi stagnation	a pathological change in which a long-standing or severe stagnation of qi impedes the flow of blood, a condition characterized by coexistence of qi stagnation and blood stasis	気の長期的あるいは高度な停滞が血流を阻害する病的変化。気滞と血瘀の同時存在を特徴とする。
氣病及血		1.7.123	qi disease affecting the blood	a pathological change in which diseased conditions of qi tend to induce blood disorders	気の病的状態が、血液障害を引き起こす傾向にある病的変化。
血不歸經	血不歸經; 血不循經	1.7.124	blood failing to stay in the meridians	a pathological change that causes extravasation of blood	溢血を生じる病的変化。
血不循經	血不歸經; 血不循經	1.7.124	blood failing to stay in the meridians	a pathological change that causes extravasation of blood	溢血を生じる病的変化。
血熱妄行		1.7.125	frenetic movement of blood due to heat	a pathological change in which heat forces extravasation of blood	熱が溢血を引き起こす病的変化。
氣隨血脫	氣隨血脫; 血脫氣脫	1.7.126	qi collapse following bleeding	a pathological change in which massive loss of blood leads to collapse of qi	大量失血が気脱に至る病的状態。
血脫氣脫	氣隨血脫; 血脫氣脫	1.7.126	qi collapse following bleeding	a pathological change in which massive loss of blood leads to collapse of qi	大量失血が気脱に至る病的状態。
氣血兩虛		1.7.127	dual deficiency of qi and blood	simultaneous presence of qi deficiency and blood deficiency	気虚と血虚の同時存在。
氣陰兩虛		1.7.128	dual deficiency of qi and yin	simultaneous presence if qi deficiency and yin deficiency	気虚と陰虚の同時存在。
傷津		1.7.129	damage to fluid	a general term for various impairments and loss of body fluid	体液のさまざまな障害および喪失を指す一般用語。
津脫	津脫; 亡津液	1.7.130	fluid collapse	a severe form of damage to fluid, usually due to profuse sweating, excessive vomiting or diarrhea	高度な津の障害。通常、大量発汗、過度の嘔吐または下痢に起因する。
亡津液	津脫; 亡津液	1.7.130	fluid collapse	a severe form of damage to fluid, usually due to profuse sweating, excessive vomiting or diarrhea	高度な津の障害。通常、大量発汗、過度の嘔吐または下痢に起因する。
津液虧損		1.7.131	fluid-humor depletion	a pathological change marked by insufficiency of body fluid-humor that fails to moisten the body tissues	津液の不足による身体組織の滋潤不能を特徴とする病的変化。
津枯血燥		1.7.132	fluid consumption and blood dryness	a pathological change in which insufficiency of fluid gives rise to blood dryness with endogenous heat	津の不足が内熱を伴う血燥を引き起こす病的変化。
津虧血瘀		1.7.133	fluid depletion and blood stasis	a pathological change in which fluid deficiency causes stagnation of blood flow	津の不足が血流の停滞を引き起こす病的状態。
氣隨液脫		1.7.134	qi collapse due to humor depletion	a pathological change in which qi collapse occurs following massive loss of fluid	津の大量喪失に続き気脱が生じる病的変化。
氣不化津		1.7.135	qi failing to form fluid	insufficient yang qi with reduced vaporizing action failing to induce the formation of body fluid	気化作用の低下を伴う陽気の不足による、体液の生成不能。
水停氣阻		1.7.136	water retention due to obstruction of qi	a pathological change in which retention of water in the body causes obstruction of qi movement	体内での水分貯留が気機の阻害を引き起こす病的状態。

検索表示（原表示の複数語を分離）	IST原表示	Code	Term	Definition/Description	定義/記述
上厥下竭		1.7.137	upper body reversal and lower body exhaustion	a pathological change marked by exhaustion of genuine yin and yang in the lower part of the body with syncope	下半身での真陰および真陽の完全な消耗を特徴とする病的変化で、失神を伴う。
下厥上冒		1.7.138	lower body reversal with upper body veiling	a pathological change in which the spleen fails to send the clear up while the stomach qi rises to the head adversely, resulting in dizziness, blurred vision accompanied by nausea, vomiting, and epigastric distension and pain	脾臓による昇清が不能となり、胃気が頭へと逆方向に上昇する病的変化。めまい、悪心を伴う霧視、嘔吐および上腹部の膨満および疼痛が生じる。
心氣盛		1.7.139	exuberant heart qi	a pathological change in which pathogenic factors make the heart qi exuberant, resulting in anxiety or other mental abnormalities	病原因子によって心気が過盛になる病的変化。不安または他の精神異常が生じる。
心氣虛	心氣虛; 心氣不足	1.7.140	heart qi deficiency	a pathological state showing hypofunction of the heart qi, usually manifested by palpitations, shortness of breath, oppressed feelings in the precordial region, spontaneous sweating and weak irregular pulse	心気の機能低下を示す病的状態。通常、動悸、息切れ、前胸部の圧迫感、自然発汗、および弱い不整脈が発現する。
心氣不足	心氣虛; 心氣不足	1.7.140	heart qi deficiency	a pathological state showing hypofunction of the heart qi, usually manifested by palpitations, shortness of breath, oppressed feelings in the precordial region, spontaneous sweating and weak irregular pulse	心気の機能低下を示す病的状態。通常、動悸、息切れ、前胸部の圧迫感、自然発汗、および弱い不整脈が発現する。
心氣不寧		1.7.141	disquieted heart qi	a pathological change characterized by a feeling of uneasiness together with severe palpitations, susceptibility to fright, vexation and insomnia, the same as restless heart qi	重度の動悸を伴う不安感、驚きやすさ、心煩、および不眠を特徴とする病的変化。restless heart qiと同義。
心氣不收	心氣不收; 心氣不固	1.7.142	non-contraction of heart qi	a pathological condition in which the heart qi floats astray, characterized by dissipated spirit, heart palpitations, and susceptibility to fright; the same as insecurity/dispersion of heart qi	心気が浮遊する病的状態。精神の消耗、動悸、驚きやすさを特徴とする。insecurity/dispersion of heart qiと同義。
心氣不固	心氣不收; 心氣不固	1.7.142	non-contraction of heart qi	a pathological condition in which the heart qi floats astray, characterized by dissipated spirit, heart palpitations, and susceptibility to fright; the same as insecurity/dispersion of heart qi	心気が浮遊する病的状態。精神の消耗、動悸、驚きやすさを特徴とする。insecurity/dispersion of heart qiと同義。
心血虛	心血虛; 心血不足	1.7.143	heart blood deficiency	a pathological change of the heart that causes dizziness, insomnia, dream-disturbed sleep palpitation and thready weak pulse	心臓の病的変化で、めまい、不眠、多夢、動悸、および弱脈を生じる。
心血不足	心血虛; 心血不足	1.7.143	heart blood deficiency	a pathological change of the heart that causes dizziness, insomnia, dream-disturbed sleep palpitation and thready weak pulse	心臓の病的変化で、めまい、不眠、多夢、動悸、および弱脈を生じる。
心血瘀阻		1.7.144	heart blood stasis (obstruction)	a pathological change of the heart in which the blood flow in the heart vessels is impeded, causing a feeling of suffocation and precordial pain	心血管の血流が阻害される心臓の病的変化。窒息感および前胸部の疼痛を生じる。
心陰虛	心陰虛; 心陰不足	1.7.145	heart yin deficiency	a pathological change of the heart in which deficiency of yin fails to check yang and results in relative preponderance of heart yang with such manifestations as mental unsteadiness, insomnia, night sweats, and feverish sensation in the palms of the hands and soles of the feet	陰虚により陽を抑制できない心臓の病的変化。心陽が相対的に優性となり、精神不安定、不眠、寝汗、掌および足の裏の熱感などの症候が発現する。
心陰不足	心陰虛; 心陰不足	1.7.145	heart yin deficiency	a pathological change of the heart in which deficiency of yin fails to check yang and results in relative preponderance of heart yang with such manifestations as mental unsteadiness, insomnia, night sweats, and feverish sensation in the palms of the hands and soles of the feet	陰虚により陽を抑制できない心臓の病的変化。心陽が相対的に優性となり、精神不安定、不眠、寝汗、掌および足の裏の熱感などの症候が発現する。
心陽虛	心陽虛; 心陽不足	1.7.146	heart yang deficiency	a pathological change referring to diminution of the heart function in controlling blood and vessels and in governing the mental activities associated with deficiency of yang qi that causes cold manifestations	血および血管の制御、ならびに精神活動の抑制における心機能の低下を指す病的変化。陽虚に関連し、寒証を引き起こす。
心陽不足	心陽虛; 心陽不足	1.7.146	heart yang deficiency	a pathological change referring to diminution of the heart function in controlling blood and vessels and in governing the mental activities associated with deficiency of yang qi that causes cold manifestations	血および血管の制御、ならびに精神活動の抑制における心機能の低下を指す病的変化。陽虚に関連し、寒証を引き起こす。
心火上炎		1.7.147	heart fire flaming upward	a pathological change in which fire flares upward along the heart meridian, causing mental restlessness and oral or lingual erosion	心経に沿って火が上方向へと広がる病的変化。精神不安定および言語機能の低下を生じる。
心火亢盛		1.7.148	hyperactive heart fire	a pathological change marked by exuberant fire of the heart that causes mental disturbances, damage to fluid, and bleeding	心火の過盛を特徴とする病的変化。精神障害、津の障害、および出血を生じる。
心火内熾	心火内熾; 心火内焚	1.7.149	internal blazing of heart fire	a pathological change marked by exuberant heart fire that disturbs mental activities, causing vexation, insomnia, throbbing palpitation, restlessness, or even mania	心火の過盛を特徴とする病的変化。精神活動を阻害し、心煩、不眠、動悸、情動不安、さらには躁病を引き起こす。
心火内焚	心火内熾; 心火内焚	1.7.149	internal blazing of heart fire	a pathological change marked by exuberant heart fire that disturbs mental activities, causing vexation, insomnia, throbbing palpitation, restlessness, or even mania	心火の過盛を特徴とする病的変化。精神活動を阻害し、心煩、不眠、動悸、情動不安、さらには躁病を引き起こす。
心營過耗		1.7.150	overconsumption of heart nutrient	a pathological change in which the nutrient of the heart is excessively consumed by heat or a chronic deficiency conditions, causing emaciation, night fever, and vexation	心臓の営気が熱によって過剰に消費される、あるいは慢性的な不足状態にある病的変化。衰弱、夜間の発熱および心煩を引き起こす。
神不守舍		1.7.151	spirit failing to keep to its abode	mental derangement when the spirit is made to leave the heart	神が心臓を離れた際の精神異常。
熱傷神明		1.7.152	heat damaging bright spirit	mental disturbance such as coma, unconsciousness, delirium caused by high fever in febrile disease	熱性疾患で壮熱によって引き起こされる昏睡、意識喪失、譫妄などの精神障害。
熱入心包		1.7.153	heat entering the pericardium	a morbid condition occurring in the nutrient and blood aspect of an epidemic febrile disease, manifested as high fever, coma, delirium, cold limbs or convulsion	流行性熱性疾患において営分および血分で発生する病的な状態。壮熱、昏睡、譫妄、手足の冷え、痙攣などが発現する。
逆傳心包		1.7.154	reverse transmission to the pericardium	the impairment of consciousness or coma occurring soon after the onset of an acute febrile disease, whereby the warm pathogen directly enters the pericardium without passing through the qi aspect	急性熱性疾患の発症直後に起こる意識障害または昏睡。温邪が気分を介することなく、直接心膜に侵入する。
痰蒙心包	痰蒙心包; 痰迷心竅	1.7.155	phlegm clouding the pericardium	a pathological change in which phlegm causes mental confusion; the same as phlegm confounding the orifices of the heart	痰が精神錯乱を引き起こす病的変化。phlegm confounding the orifices of the heartと同義。

検索表示（原表示の複数語を分離）	IST原表示	Code	Term	Definition/Description	定義/記述
痰迷心竅	痰蒙心包; 痰迷心竅	1.7.155	phlegm clouding the pericardium	a pathological change in which phlegm causes mental confusion; the same as phlegm confounding the orifices of the heart	痰が精神錯乱を引き起こす病的変化。phlegm confounding the orifices of the heartと同義。
痰火擾心		1.7.156	phlegm-fire harassing the heart	a pathological change in which fire-heat and phlegm-turbidity harassing the heart-spirit, lead to mental disturbance	火熱および痰濁が心神を攻撃する病的変化。精神障害に至る。
水氣凌心		1.7.157	water qi intimidating the heart	a pathological change in which upsurge of retained water causes disturbances of the heart	貯留水の急増が心臓の障害を引き起こす病的変化。
心肝火旺		1.7.158	effulgent heart-liver fire	a pathological change in which exuberant liver fire and heart fire disturb mental activity, or cause vascular damage and frenetic blood flow, the same as hyperactive heart-liver fire	過盛な肝火および心火が精神活動を阻害する、あるいは血管損傷および血流の乱れを生じる病的変化。
心肝血虛		1.7.159	heart-liver blood deficiency	a pathological change in which deficiency of heart blood and liver blood deprives the mental activities, head, eyes, sinews and nails of nourishment	心血虚および肝血虚が精神活動、頭部、眼、筋肉、爪から栄養を奪う病的変化。
心胃火燔		1.7.160	heart-stomach fire ablaze	a pathological change arising when intense heart heat gives rise to mental disquiet and stomach fire causes damage to fluids	極度の心熱が精神不安を、胃火が津の障害を引き起こす際に起こる病的変化。
心脾兩虛		1.7.161	dual deficiency of the heart-spleen	a pathological change in which deficiency of both heart blood and spleen qi leads to disordered heart function and disquietness together with failure of the spleen in transportation and transformation	心血および脾気双方の不足が心機能の障害と動揺および脾臓の運化不全に至る病的変化。
心腎不交		1.7.162	non-interaction between the heart and kidney	a disorder of the normal relationship between the heart and kidney ascribed to deficiency of kidney yin and stirring of heart fire	心臓と腎臓の正常な関係の乱れ。腎陰虚および活発な心火に起因する。
心肺氣虛		1.7.163	heart-lung qi deficiency	a pathological change characterized by deficiency of both heart qi and lung qi with impairment of blood circulation and qi diffusion	心気虚および肺気虚を特徴とする病的変化。血流障害および気の拡散を伴う。
心虛膽怯		1.7.164	heart deficiency with timidity	a pathological change characterized by simultaneous deficiency of heart qi and gallbladder qi, marked by disquietness and susceptibility to fright and fear	心気および胆気双方の不足を特徴とする病的変化。動揺および驚きや不安に対する脆弱性を呈する。
小腸實熱		1.7.165	small intestinal excess heat	a pathological change of excess heat ascribed to shifting of heart fire to the small intestine	心火の小腸への移動に起因する実熱の病的変化。
小腸虛寒		1.7.166	small intestinal deficiency cold	impairment of the small intestine by insufficient yang qi with endogenous cold, affecting the separation of the clear and turbid	内寒を伴う陽気の不足による小腸の機能障害。泌別清濁に悪影響を及ぼす。
肝氣實	肝氣實; 肝氣盛	1.7.167	excess of liver qi	presence of excessive pathogenic qi in the liver or liver meridian; the same as exuberance of liver qi	肝臓または肝経における過剰な邪気の存在。exuberance of liver qiと同義。
肝氣盛	肝氣實; 肝氣盛	1.7.167	excess of liver qi	presence of excessive pathogenic qi in the liver or liver meridian; the same as exuberance of liver qi	肝臓または肝経における過剰な邪気の存在。exuberance of liver qiと同義。
肝氣上逆		1.7.168	liver qi ascending counterflow	the hyperactive liver qi attacking the upper part of the body, manifested as dizziness, headache, flushed face, tinnitus, deafness, fullness and pain of the chest and hypochondrium, belching, acid regurgitation, and even hematemesis, and a taut and forceful pulse	活動が亢進した肝気による上半身の攻撃。めまい、頭痛、顔面紅潮、耳鳴り、難聴、胸部および下肋部の充満感および疼痛、おくび、胃酸逆流、さらには吐血および緊張した実脈が発現する。
肝氣不舒		1.7.169	constrained liver qi	a disorder leading to functional disturbances of the liver, manifested as irritability, distension and pain of the chest, hypochondrium, and lower abdomen, distending pain of the breast and abnormal menstruation in the female	肝機能障害に至る障害。興奮性、胸部、下肋部および下腹部の膨満および疼痛、女性では乳房の膨張痛および月経異常が発現する。
肝氣不和		1.7.170	disharmony of liver qi	a pathological change of the liver in its smoothing and discharging function, causing irritability, hypochondriac, mammary or lower abdominal distension and pain, and irregular menstruation	肝臓の円滑化・排出機能の病的変化。興奮性、下肋部、乳房あるいは下腹部の膨満および疼痛、月経異常を引き起こす。
肝氣橫逆		1.7.171	transverse invasion of liver qi	a pathological change in which the depressed liver qi runs transversely, impairing the spleen and stomach	低下した肝気が横方向に走り、脾臓および胃を害する病的変化。
肝氣犯胃		1.7.172	liver qi invading the stomach	a pathological change in which the depressed liver qi running transversely impairs the stomach function in food intake and digestion	低下した肝気が横方向に走り、胃の食物受け入れおよび消化機能を害する病的変化。
肝氣犯脾		1.7.173	liver qi invading the spleen	a pathological change in which the depressed liver qi running transversely impairs the spleen function in transportation and transformation	低下した肝気が横方向に走り、脾臓の運化機能を害する病的変化。
肝虛		1.7.174	liver deficiency	a general term for deficiency conditions of the liver, including deficiency of liver qi, liver blood, liver yin, and liver yang	肝臓の虚状態（肝気虚、肝血虚、肝陰虚、肝陽虚など）を指す一般用語。
肝氣虛		1.7.175	liver qi deficiency	a morbid condition characterized by deficiency of qi resulting in impaired function of the liver in smoothing the flow of qi	気虚を特徴とする病的状態で、気の流れの円滑化における肝機能障害をもたらす。
肝血虛		1.7.176	liver blood deficiency	a morbid condition characterized by depletion of blood in the liver	肝臓における血の減少を特徴とする病的状態。
肝陰虛		1.7.177	liver yin deficiency	a pathological change characterized by insufficient yin fluid of the liver that fails to nourish the head, eyes, sinews and nails, and produces deficiency-heat	肝臓の陰津不足を特徴とする病的変化。頭部、眼、腱および爪の滋養が不能となり、虚熱が生成される。
肝陽虛	肝陽虛; 肝虛寒	1.7.178	liver yang deficiency	a pathological change characterized by insufficient yang qi of the liver that reduces the function of free coursing and storing blood, and produces deficiency cold, also called liver deficiency cold	肝臓の陽気不足を特徴とする病的変化。血の疏泄および貯蔵機能が低下し、虚寒が生成される。liver deficiency coldとも呼ばれる。
肝虛寒	肝陽虛; 肝虛寒	1.7.178	liver yang deficiency	a pathological change characterized by insufficient yang qi of the liver that reduces the function of free coursing and storing blood, and produces deficiency cold, also called liver deficiency cold	肝臓の陽気不足を特徴とする病的変化。血の疏泄および貯蔵機能が低下し、虚寒が生成される。liver deficiency coldとも呼ばれる。
肝陽上亢	肝陽上亢; 肝陽偏旺	1.7.179	ascendant hyperactivity of liver yang	a pathological change in which deficient liver-kidney yin lets liver yang get out of control and stir upward	肝腎陰の不足により、肝陽が制御不能となり、上昇する病的変化。

検索表示（原表示の複数語を分離）	IST原表示	Code	Term	Definition/Description	定義/記述
肝陽偏旺	肝陽上亢; 肝陽偏旺	1.7.179	ascendant hyperactivity of liver yang	a pathological change in which deficient liver-kidney yin lets liver yang get out of control and stir upward	肝腎陰の不足により、肝陽が制御不能となり、上昇する病的変化。
肝陽化火		1.7.180	liver yang transforming into fire	ascendant hyperactivity of liver yang giving rise to fire	肝陽上亢が進展し火が生じる。
肝陽化風		1.7.181	liver yang transforming into wind	hyperactivity of liver yang stirring the internal wind	肝陽の活動過剰が内風の混乱を招く。
肝火		1.7.182	liver fire	a pathological change of exuberant liver qi with heat manifestations	過盛な肝気の病的変化で熱証を伴う。
肝火上炎		1.7.183	liver fire flaming upward	a pathological change characterized by exuberant liver fire flaming upward to the head and eyes	過盛な肝火を特徴とする病的変化。肝火は頭部および眼へと上昇する。
肝火犯肺		1.7.184	liver fire invading the lung	a pathological change characterized by exuberant liver fire ascending counterflow to invade the lung	過盛な肝火を特徴とする病的変化。肝火は逆方向の上方へと広がり、肺に侵入する。
肝風	肝風; 肝風内動; 風氣內動	1.7.185	liver wind	abnormal movement of the body's yang qi turning to internal wind, a general term for liver yang transforming into wind, extreme heat engendering wind, yin deficiency with stirring wind, and blood deficiency engendering wind, also called internal stirring of liver wind	身体の陽気が内風に変化する異常な動き。肝陽の風への変化、極度の熱による風の発生、動風を伴う陰虚、および血虚による風の発生を指す一般用語。internal stirring of liver windとも呼ばれる。
肝風內動	肝風; 肝風內動; 風氣內動	1.7.185	liver wind	abnormal movement of the body's yang qi turning to internal wind, a general term for liver yang transforming into wind, extreme heat engendering wind, yin deficiency with stirring wind, and blood deficiency engendering wind, also called internal stirring of liver wind	身体の陽気が内風に変化する異常な動き。肝陽の風への変化、極度の熱による風の発生、動風を伴う陰虚、および血虚による風の発生を指す一般用語。internal stirring of liver windとも呼ばれる。
風氣內動	肝風; 肝風內動; 風氣內動	1.7.185	liver wind	abnormal movement of the body's yang qi turning to internal wind, a general term for liver yang transforming into wind, extreme heat engendering wind, yin deficiency with stirring wind, and blood deficiency engendering wind, also called internal stirring of liver wind	身体の陽気が内風に変化する異常な動き。肝陽の風への変化、極度の熱による風の発生、動風を伴う陰虚、および血虚による風の発生を指す一般用語。internal stirring of liver windとも呼ばれる。
熱極生風		1.7.186	extreme heat engendering wind	a pathological change characterized by convulsions that arise when exuberant pathogenic heat scorches the liver meridian and deprives the sinews of nourishment	過盛な熱邪が肝経を焼き、腱から栄養を奪う際に生じる痙攣を特徴とする病的変化。
肝寒		1.7.187	liver cold	(1) a morbid condition characterized by cold due to insufficient liver yang that causes congealing of liver qi with depression, timidity, lassitude, cold limbs, sunken thready pulse; (2) the same as cold stagnating in the liver vessel	(1) 肝陽虚に起因する寒を特徴とする病的状態で、鬱状態、臆病、倦怠感、手足の冷え、沈細脈を伴う肝気の停滞を生じる、(2) cold stagnating in the liver vesselと同義。
寒滯肝脈		1.7.188	cold stagnating in the liver vessel	a pathological change marked by cold pathogen congealing in the liver meridian	肝経における寒邪の停滞を特徴とする病的変化。
肝熱		1.7.189	liver heat	a general term for various heat syndromes of the liver such as liver fire and ascendant hyperactivity of liver yang	肝火や肝陽上亢など、肝臓のさまざまな証を指す一般用語。
肝實熱		1.7.190	liver excess heat	a pathological state characterized by presence of exuberant fire-heat in the liver	肝臓における過盛な火熱の存在を特徴とする病的状態。
肝經濕熱		1.7.191	dampness-heat in the liver meridian	a pathological change attributable to dampness-heat accumulated in the liver and pouring downward along the liver meridian	肝臓における湿熱の蓄積および肝経に沿った下注に起因する病的変化。
肝膽濕熱		1.7.192	liver-gallbladder dampness-heat	accumulation of dampness-heat in the liver and gallbladder resulting in impaired bile flow and downward pouring of dampness-heat	肝臓および胆嚢における湿熱の蓄積。胆汁流および湿熱下注の障害を生じる。
肝經實熱		1.7.193	excess heat in the liver meridian	accumulation of exuberant fire-heat in the liver meridian	肝経における過盛な火熱の蓄積。
肝膽俱實		1.7.194	dual excess of the liver-gallbladder	accumulation of excessive pathogen (such as stagnant qi, impeded dampness, accumulated heat, static blood or calculi) in the liver and gallbladder	肝臓および胆嚢における過剰な病邪（気の停滞、湿障害、熱の蓄積、血または結石の停滞など）の蓄積。
肝膽氣鬱		1.7.195	liver-gallbladder qi depression	stagnation of qi in the liver and gallbladder with impaired function of free coursing and bile discharge, the same as liver-gallbladder qi stagnation	肝臓および胆嚢における気の停滞で、疏泄および胆汁排出機能の障害を伴う。liver-gallbladder qi stagnationと同義。
肝氣鬱結	肝氣鬱結; 肝鬱	1.7.196	liver qi depression	stagnation of qi in the liver resulting from impairment of free coursing, also called liver depression	疏泄障害に起因する、肝臓における気の停滞。liver depressionとも呼ばれる。
肝鬱	肝氣鬱結; 肝鬱	1.7.196	liver qi depression	stagnation of qi in the liver resulting from impairment of free coursing, also called liver depression	疏泄障害に起因する、肝臓における気の停滞。liver depressionとも呼ばれる。
肝鬱脾虛		1.7.197	liver depression and spleen deficiency	a pathological change in which depressed liver qi impairs the transporting and transforming function of the spleen, the same as liver stagnation and spleen deficiency	肝気鬱結により脾臓の運化機能が害される病的変化。liver stagnation and spleen deficiencyと同義。
肝腎虧損		1.7.198	liver-kidney depletion	a pathological change in which insufficient essence and blood of the liver and kidney fail to nourish the related body constituents and organs, but do not produce deficiency-fire	肝臓と腎臓における精および血の不足により、関連する体成分や内臓を滋養できない病的変化。ただし虚火は生じない。
肝腎陰虛		1.7.199	liver-kidney yin deficiency	a pathological change in which insufficient yin fluid of the liver and kidney fails to nourish the related body constituents and organs, and gives rise to deficiency-fire symptoms	肝臓と腎臓における陰津の不足により、関連する体成分や内臓を滋養できない病的変化。虚火証を生じる。
膽熱		1.7.200	gallbladder heat	a morbid condition ascribed to attack on the gallbladder and gallbladder meridian by pathogenic heat	熱邪による胆嚢および胆経の攻撃に起因する病的状態。
膽實熱		1.7.201	gallbladder excess heat	a pathological change characterized by excess heat in the gallbladder and gallbladder meridian	胆嚢および胆経における実熱を特徴とする病的変化。
膽氣不足	膽氣不足; 膽虛氣怯	1.7.202	insufficiency of gallbladder qi	a pathological change ascribed to insufficiency of the gallbladder qi that makes the mind disquieted and susceptible to fright	胆気の不足に起因する病的変化。不安および驚きやすさを生じる。
膽虛氣怯	膽氣不足; 膽虛氣怯	1.7.202	insufficiency of gallbladder qi	a pathological change ascribed to insufficiency of the gallbladder qi that makes the mind disquieted and susceptible to fright	胆気の不足に起因する病的変化。不安および驚きやすさを生じる。
脾寒		1.7.203	spleen cold	a general term for various cold conditions of the spleen	脾臓のさまざまな寒状態を指す一般用語。
脾熱		1.7.204	spleen heat	a general term for various heat conditions of the spleen	脾臓のさまざまな熱状態を指す一般用語。

検索表示（原表示の複数語を分離）	IST原表示	Code	Term	Definition/Description	定義/記述
脾虛		1.7.205	spleen deficiency	a general term for deficiency condition of the spleen, such as spleen qi deficiency, spleen yang deficiency, and spleen yin deficiency	脾臓の虚状態（脾気虚、脾陽虚、脾陰虚など）を指す一般用語。
脾氣虛		1.7.206	spleen qi deficiency	a pathological change characterized by qi deficiency with impaired transporting and transforming function of the spleen	脾臓の運化機能障害を伴う気虚を特徴とする病的変化。
脾陰虛		1.7.207	spleen yin deficiency	a pathological change ascribed to deficiency of yin in the spleen with inadequate distribution of essence to the body	脾臓における陰虚に起因する病的変化で、身体への精配分不良を伴う。
脾陽虛	脾陽虛; 脾虛寒	1.7.208	spleen yang deficiency	a pathological change characterized by deficiency of yang in the spleen resulting in deficiency-cold	脾臓における陽虚を特徴とする病的変化で、虚寒を生じる。
脾虛寒	脾陽虛; 脾虛寒	1.7.208	spleen yang deficiency	a pathological change characterized by deficiency of yang in the spleen resulting in deficiency-cold	脾臓における陽虚を特徴とする病的変化で、虚寒を生じる。
脾實	脾實; 脾氣實	1.7.209	spleen excess	a pathological state ascribed to accumulation of excessive pathogenic qi in the spleen, also the same as spleen qi excess	脾臓における過剰な邪気の蓄積に起因する病的状態。spleen qi excessと同義。
脾氣實	脾實; 脾氣實	1.7.209	spleen excess	a pathological state ascribed to accumulation of excessive pathogenic qi in the spleen, also the same as spleen qi excess	脾臓における過剰な邪気の蓄積に起因する病的状態。spleen qi excessと同義。
脾實熱		1.7.210	spleen excess heat	a pathological condition characterized by exuberant pathogenic heat in the spleen	脾臓における過盛な熱邪を特徴とする病的状態。
脾不統血		1.7.211	spleen failing to control the blood	a pathological change characterized by failure of the spleen to keep the blood flowing in the vessels, resulting in hemorrhage, the same as spleen failing to keep the blood within the vessel	脾臓による血管内での血流維持不能を特徴とする病的変化。出血を生じる。spleen failing to keep the blood within the vesseと同義。
濕傷脾陽		1.7.212	dampness damaging spleen yang	a pathological change characterized by stagnant cold-dampness causing damage to spleen yang	寒湿の停滞を特徴とする病的変化で、脾陽を害する。
濕傷脾陰		1.7.213	dampness damaging spleen yin	a pathological change in which the depressed dampness transforms into heat causing damage to spleen yin	低下した湿が火に変化する病的変化で、脾陰を害する。
寒濕困脾		1.7.214	cold-dampness encumbering the spleen	a pathological change in which excessive cold-dampness impairs spleen yang, the same as cold-dampness accumulating in the spleen	過剰な寒湿が脾陽を害する病的変化。cold-dampness accumulating in the spleenと同義。
脾虛濕困		1.7.215	spleen deficiency with dampness encumbrance	a complex condition of deficiency and excess wherein spleen deficiency leads to accumulation of dampness-turbidity, the same as spleen deficiency with dampness accumulation	虚と実の複雑な状態で、脾虚が湿濁の蓄積に至る。spleen deficiency with dampness accumulationと同義。
脾虛生風		1.7.216	spleen deficiency engendering wind	a pathological change characterized by stirring of wind internally due to spleen deficiency	脾虚に起因する風の内動を特徴とする病的変化。
脾胃濕熱		1.7.217	spleen-stomach dampness-heat	a pathological condition ascribed to accumulation of dampness-heat which impairs the functions of the spleen and stomach, the same as dampness-heat in the middle energizer	湿熱の蓄積に起因する病的状態で、脾臓および胃の機能を害する。dampness-heat in the middle energizerと同義。
脾胃虛寒		1.7.218	spleen-stomach deficiency cold	a pathological change characterized by decline of yang qi of the spleen and stomach with diminished functions and endogenous deficiency-cold	脾臓および胃における陽気の低下を特徴とする病的変化で、機能低下と内虚寒を伴う。
脾胃俱虛		1.7.219	dual deficiency of the spleen-stomach	a deficiency condition of both the spleen and stomach, marked by a feeling of emptiness in the stomach, shortness of breath, reversal cold of the limbs and incessant watery diarrhea	脾臓および胃双方の虚状態。胃の空虚感、息切れ、手足厥冷、および絶え間ない水様便を特徴とする。
脾胃虛弱		1.7.220	spleen-stomach weakness	a pathological change characterized by diminished spleen and stomach function of food intake and digestion	脾臓および胃の食物摂取･消化機能低下を特徴とする病的変化。
脾胃俱實		1.7.221	dual excess of the spleen-stomach	a pathological change marked by presence of excessive pathogenic qi in both the spleen and stomach	脾臓および胃双方における過剰な邪気の存在を特徴とする病的変化。
脾胃陰虛		1.7.222	spleen-stomach yin deficiency	a pathological change in which deficiency of yin fluid of the spleen-stomach results in impaired food intake and digestion	脾臓および胃の陰津不足により食物摂取･消化が害される病的変化。
脾腎陽虛		1.7.223	spleen-kidney yang deficiency	a pathological change characterized by insufficient yang qi of the spleen and kidney with endogenous cold and flooding of water	脾臓および腎臓の陽気不足を特徴とする病的変化で、内寒および水の横溢を伴う。
脾失健運		1.7.224	spleen failing in transportation	dysfunction of the spleen in transporting the essence of food and water	脾臓の、水穀の精微の運化機能障害。
脾氣不舒		1.7.225	constrained spleen qi	a pathological change marked by dysfunction of the spleen with impaired digestion and absorption	脾臓の機能障害を特徴とする病的変化で、消化･吸収障害を伴う。
脾氣不升		1.7.226	spleen qi failing to bear upward	dysfunction of the spleen in upbearing nutrients, the same as spleen qi failing to ascend	脾臓の栄養昇発機能障害。spleen qi failing to ascendと同義。
脾氣下陷		1.7.227	sunken spleen qi	a pathological change characterized as sinking of spleen qi induced by weakness of the spleen with diminished upbearing function	脾臓の衰弱による脾気の低下を特徴とする病的変化で、昇発機能の低下を伴う。
中氣不足		1.7.228	insufficiency of middle qi	deficiency of qi in the middle energizer, marked by diminished function of the spleen and stomach	中焦における気虚で、脾臓と胃の機能低下を特徴とする。
中陽不振		1.7.229	devitalized middle yang	weakness of yang qi in the middle energizer, marked by diminished function of the spleen and stomach in digestion and absorption	中焦における陽気の衰弱で、脾臓および胃の消化･吸収機能低下を特徴とする。
胃家實		1.7.230	excessiveness in the stomach-intestines	a pathological change characterized by presence of pathogens deep in the yang brightness meridian that causes exuberant dryness-heat in the gastrointestinal tract	陽明経の深部における病邪の存在を特徴とする病的変化で、胃腸管で過盛な燥熱を生じる。
胃實		1.7.231	stomach excess	a morbid condition due to consumption of body fluid and stagnation of stomach qi, caused by excessive heat accumulated in the stomach	体液の消耗および胃気の停滞に起因する病的状態。胃における過剰な熱の蓄積によって生じる。
胃虛		1.7.232	stomach deficiency	a general term for deficiency conditions of the stomach, including qi deficiency, yin deficiency and yang deficiency of the stomach	胃の虚状態（胃気虚、胃陰虚、胃陽虚など）を指す一般用語。
胃氣虛		1.7.233	stomach qi deficiency	a pathological change characterized by weakness of stomach qi with impaired appetite and digestion	胃気の衰弱を特徴とする病的変化で、食欲および消化機能の低下を伴う。
胃陰虛		1.7.234	stomach yin deficiency	a pathological change characterized by deficiency of fluid in the stomach with impaired function attributable to intense stomach fire or exuberant heat in a warm disease	胃における津虚を特徴とする病的変化で、過度の胃火または温病での過盛な熱に起因する機能障害を伴う。

検索表示（原表示の複数語を分離）	IST原表示	Code	Term	Definition/Description	定義/記述
胃陽虛		1.7.235	stomach yang deficiency	a pathological change characterized by decline of yang qi which leads to deficiency-cold of the stomach with markedly impaired appetite and digestion	陽気の低下を特徴とする病的変化で、著しい食欲および消化機能の低下を伴う胃の虚寒に至る。
胃寒		1.7.236	stomach cold	a pathological change either due to deficiency of stomach yang or caused by direct attack of pathogenic cold, the former being deficiency-cold of the stomach, and the latter, excess-cold in the stomach	胃陽虚（胃の虚寒）または寒邪による直接的な攻撃（胃の実寒）に起因する病的変化。
胃熱		1.7.237	stomach heat	a pathological change due to impairment of the stomach by pathogenic heat or caused by overeating of hot pungent food	熱邪または辛味食品の過剰摂取による胃の機能障害に起因する病的変化。
胃熱消穀		1.7.238	stomach heat with swift digestion	a pathological change in which stomach heat or fire leads to abnormally rapid digestion	胃熱または胃火が異常に急速な消化を引き起こす病的変化。
胃火上升		1.7.239	stomach fire bearing upward	a pathological change in which exuberant fire in the stomach flares up along the meridian/channel, causing toothache, gum swelling or bleeding, or hematemesis	過盛な胃火が経絡に沿って炎上する病的変化。歯痛、歯肉の腫脹または出血、あるいは吐血を生じる。
胃火熾盛		1.7.240	intense stomach fire	presence of excessive pathogenic heat in the stomach with upward flaming of stomach fire	胃における過剰な邪熱の存在で、胃火の上方への炎上を伴う。
胃氣不降		1.7.241	stomach qi failing to bear downward	impaired downbearing of stomach qi, causing anorexia, nausea, vomiting, belching, and a feeling of fullness in the epigastric region, the same as stomach qi failing to descend	胃気の降下不良。食欲不振、悪心、嘔吐、おくび、上腹部膨満感を生じる。stomach qi failing to descendと同義。
胃氣上逆		1.7.242	stomach qi ascending counterflow	a pathological change characterized by counterflow ascent of stomach qi that causes belching, hiccups, acid regurgitation and vomiting	胃気の上方向への逆流を特徴とする病的変化。おくび、しゃっくり、胃酸逆流および嘔吐を生じる。
胃不和		1.7.243	stomach disharmony	a general term referring to various functional disorders of the stomach, i.e., dysfunction in receiving and digesting food as well as in conducting the contents to the intestines	胃のさまざまな機能障害（食物の受け入れ・消化機能、および腸内容物の誘導機能の障害）を指す一般用語。
肺脾兩虛	肺脾兩虛; 脾肺兩虛; 肺脾氣虛	1.7.244	dual deficiency of the lung-spleen	qi deficiency of both the lung and spleen marked by failure of the spleen in transportation and transformation and the lung in diffusion and depurative downbearing; the same as lung-spleen qi deficiency	肺および脾臓双方の気虚。脾臓の運化不能および肺の宣発・粛降不能を特徴とする。lung-spleen qi deficiencyと同義。
脾肺兩虛	肺脾兩虛; 脾肺兩虛; 肺脾氣虛	1.7.244	dual deficiency of the lung-spleen	qi deficiency of both the lung and spleen marked by failure of the spleen in transportation and transformation and the lung in diffusion and depurative downbearing; the same as lung-spleen qi deficiency	肺および脾臓双方の気虚。脾臓の運化不能および肺の宣発・粛降不能を特徴とする。lung-spleen qi deficiencyと同義。
肺脾氣虛	肺脾兩虛; 脾肺兩虛; 肺脾氣虛	1.7.244	dual deficiency of the lung-spleen	qi deficiency of both the lung and spleen marked by failure of the spleen in transportation and transformation and the lung in diffusion and depurative downbearing; the same as lung-spleen qi deficiency	肺および脾臓双方の気虚。脾臓の運化不能および肺の宣発・粛降不能を特徴とする。lung-spleen qi deficiencyと同義。
肺絡損傷		1.7.245	damage to the lung vessels	a pathological change marked by expectoration of blood, caused by severe or enduring cough or by heat pathogen	重度または持続的な咳、あるいは熱邪によって引き起こされる吐血を特徴とする病的変化。
肺腎陰虛		1.7.246	lung-kidney yin deficiency	a pathological change characterized by deficiency of yin fluid of the lung and kidney with engendering of internal heat	肺および腎臓の陰津虚を特徴とする病的変化で、内熱を引き起こす。
肺腎氣虛		1.7.247	lung-kidney qi deficiency	a pathological change characterized by dual deficiency of lung qi and kidney qi with impaired function of depurative downbearing and qi absorption	肺および腎臓双方の気虚を特徴とする病的変化で、粛降および気吸収機能の障害を伴う。
肺津不布		1.7.248	lung failing to distribute fluid	failure of the lung to distribute fluid, leading to production of phlegm and causing cough and dyspnea	肺による津の配分不能。痰が生成され、咳および呼吸困難を生じる。
肺失清肅		1.7.249	impaired lung depuration	a pathological change of lung that gives rise to cough, dyspnea, expectoration of sputum and fullness in the chest	肺の病的変化で、咳、呼吸困難、喀痰、胸部充満感を生じる。
痰濁阻肺		1.7.250	phlegm turbidity obstructing the lung	a pathological change characterized by impairment of diffusion and downbearing of lung qi due to phlegm-dampness obstruction	痰湿による障害に起因する肺気の宣発・粛降障害を特徴とする病的変化。
風寒束肺		1.7.251	wind-cold fettering the lung	a pathological change characterized by attack of wind-cold which impairs the normal flow of lung qi	風寒の攻撃を特徴とする病的変化で、肺気の正常な流れを害する。
肺氣不宣		1.7.252	lung qi failing to diffuse	disordered diffusion of lung qi generally attributable to external pathogens invading the lung or fettering the exterior, and manifesting in cough, hoarse voice, and nasal congestion, the same as lung qi failing to disperse	肺気の宣発障害で、通常、外的病邪による肺への侵入あるいは外側の傷害に起因し、咳、嗄声、および鼻閉を生じる。lung qi failing to disperseと同義。
肺氣不利		1.7.253	inhibited lung qi	any disturbance of depurative downbearing of lung qi and the lung's governing of the waterways	肺気の粛降および肺の水道通調機能のあらゆる障害。
肺氣上逆		1.7.254	lung qi ascending counterflow	a pathological change characterized by counterflow ascending of lung qi instead of depurative downbearing	粛降に代わる、肺気の上方向への逆流を特徴とする病的変化。
肺虛		1.7.255	lung deficiency	a general term for deficiency conditions of the lung, including lung qi deficiency and lung yin deficiency	肺の虚状態（肺気虚、肺陰虚など）を指す一般用語。
肺陰虛		1.7.256	lung yin deficiency	a pathological change characterized by insufficient lung yin with endogenous heat	肺陰の不足を特徴とする病的変化で、内熱を伴う。
肺氣虛		1.7.257	lung qi deficiency	a pathological change characterized by deficiency of qi with diminished function of the lung	気虚を特徴とする病的変化で、肺機能の低下を伴う。
肺寒		1.7.258	lung cold	a term either referring to external cold invading the lung or to deficiency-cold of lung qi	肺への外寒侵入あるいは肺気の虚寒のいずれかを指す用語。
肺實	肺實; 肺氣實	1.7.259	lung excess	any excess condition of the lung, attributable to attack of the lung by external wind, cold, dryness, and heat pathogens or obstruction of the lung by phlegm-heat, phlegm-dampness, etc.	肺のあらゆる実状態で、外部からの風邪、寒邪、燥邪および熱邪による肺の攻撃、あるいは痰熱、痰湿などによる肺の閉塞に起因する。
肺氣實	肺實; 肺氣實	1.7.259	lung excess	any excess condition of the lung, attributable to attack of the lung by external wind, cold, dryness, and heat pathogens or obstruction of the lung by phlegm-heat, phlegm-dampness, etc.	肺のあらゆる実状態で、外部からの風邪、寒邪、燥邪および熱邪による肺の攻撃、あるいは痰熱、痰湿などによる肺の閉塞に起因する。
肺火		1.7.260	lung fire	a pathological change characterized by presence of intense heat in the lung, either of excess type or of deficiency type	肺における激しい熱の存在を特徴とする病的変化。実または虚いずれかのタイプ。
肺熱		1.7.261	lung heat	a general term for heat conditions of the lung	肺の熱状態を指す一般用語。

検索表示（原表示の複数語を分離）	IST原表示	Code	Term	Definition/Description	定義/記述
肺實熱		1.7.262	excess heat in the lung	a pathological change characterized by presence of excessive pathogenic heat in the lung	肺における過剰な熱邪の存在を特徴とする病的変化。
燥氣傷肺		1.7.263	dryness qi damaging the lung	a pathological change characterized by damage of the lung fluid by pathogenic dryness that impairs the diffusion and depurative downbearing function	燥邪による肺津の損傷を特徴とする病的変化で、宣発・粛降機能を害する。
火熱迫肺		1.7.264	fire heat distressing the lung	a pathological change characterized by pathogenic fire-heat accumulating in the lung or intense fire-heat scorching lung yin	肺における火熱邪の蓄積あるいは激しい火熱による肺陰の焼け焦げを特徴とする病的変化。
金破不鳴		1.7.265	broken metal failing to sound	an expression figuratively referring to hoarseness due to deficiency of lung qi	肺気虚による嗄声を比喩的に指す表現。
金實不鳴		1.7.266	excess metal failing to sound	an expression figuratively referring to hoarseness or loss of voice due to excessiveness of lung qi	過度の肺気による嗄声または失声を比喩的に指す表現。
大腸寒結		1.7.267	large intestinal cold bind	a pathological change characterized by retention of cold in the large intestine with constipation, the same as large intestinal cold accumulation	大腸における寒の保持を特徴とする病的変化で、便秘を伴う。large intestinal cold accumulationと同義。
大腸濕熱		1.7.268	large intestinal dampness-heat	a pathological change characterized by accumulation of dampness-heat in the large intestine which impairs the qi movement, damages the intestinal blood vessels, and causes abnormal bowel evacuations	大腸における湿熱の蓄積を特徴とする病的変化で、気機を阻害し、腸血管を損傷し、異常な排便を生じる。
大腸液虧		1.7.269	insufficiency of the large intestinal humor	a pathological change ascribed to insufficient fluid in the large intestine	大腸における津の不足に起因する病的変化。
大腸虛寒		1.7.270	large intestinal deficiency cold	a pathological change in which debilitation of yang qi in the large intestine leads to endogenous cold and causes abnormal bowel evacuations	大腸における陽気の衰弱が内寒に至る病的変化で、異常な排便を生じる。
大腸實熱		1.7.271	large intestinal excess heat	a pathological change characterized by exuberant pathogenic heat obstructing the bowel qi	過盛な熱邪による腑気の阻害を特徴とする病的変化。
熱迫大腸		1.7.272	heat distressing the large intestine	a pathological change in which pathogenic heat impairs the function of the large intestine, leading to acute diarrhea with abdominal pain, burning sensation in the anus, scanty dark urine, and dry yellow tongue coating	熱邪が大腸の機能を害する病的変化。異常な疼痛を伴う急性下痢、肛門の灼熱感、少量の暗色尿、および乾いた舌の黄苔を生じる。
大腸熱結		1.7.273	large intestinal heat bind	a pathological change characterized by exuberant heat in the large intestine that causes dryness and constipation, the same as large intestinal heat accumulation	大腸における過盛な熱を特徴とする病的変化で、燥と便秘を生じる。large intestinal heat accumulationと同義。
大腸虛		1.7.274	large intestinal deficiency	deficiency of the large intestinal qi with abnormal bowel evacuations	大腸の気虚で、異常な排便を伴う。
大腸實		1.7.275	large intestinal excess	excess of pathogens in the large intestine	大腸における病邪の過剰。
大腸熱		1.7.276	large intestinal heat	excess of pathogenic heat in the large intestine	大腸における熱邪の過剰。
腎虛		1.7.277	kidney deficiency	a general term for deficiency conditions of the kidney, including kidney yin deficiency, kidney yang deficiency, insufficiency of kidney essence, and insecurity of kidney qi	腎臓の虚状態（腎陰虚、腎陽虚、腎精不足、不安定な腎気など）を指す一般用語。
腎精不足		1.7.278	insufficiency of kidney essence	a pathological change of the kidney with insufficiency of its essence and impaired function	腎精の不足および腎機能障害を伴う腎臓の病的変化。
腎虛水泛		1.7.279	kidney deficiency with water flood	a pathological change characterized by deficiency of kidney yang with impaired water metabolism and resultant retention of fluid	腎陽虚を特徴とする病的変化で、水分代謝障害およびその結果生じる津の保持を伴う。
腎火偏亢		1.7.280	hyperactive kidney fire	relative excess of kidney fire due to deficiency of kidney yin	腎陰の不足に起因する、腎火の相対的過剰。
腎氣不固		1.7.281	insecurity of kidney qi	a pathological change characterized by diminished essence-storing and astringing function of the kidney	腎臓の精貯蔵および収斂機能低下を特徴とする病的変化。
腎不納氣		1.7.282	kidney failing to receive qi	deficiency of the kidney qi preventing the reception of lung qi	腎気虚による肺気受け入れの阻害。
腎陰虛		1.7.283	kidney yin deficiency	a pathological change characterized by deficiency of yin to nourish the kidney, leading to deficiency-fire or deficiency-heat	陰虚による腎臓の滋養不能を特徴とする病的変化で、虚火または虚熱に至る。
腎陽虛	腎陽虛;腎陽虛衰	1.7.284	kidney yang deficiency	a pathological change characterized by deficiency of kidney yang with diminished warming function and failure in qi transformation	腎陽虚を特徴とする病的変化で、加温機能の低下および気化不能を伴う。
腎陽虛衰	腎陽虛;腎陽虛衰	1.7.284	kidney yang deficiency	a pathological change characterized by deficiency of kidney yang with diminished warming function and failure in qi transformation	腎陽虚を特徴とする病的変化で、加温機能の低下および気化不能を伴う。
命門火衰		1.7.285	debilitation of the life gate fire	a pathological change characterized by impaired reproductive function due to insufficiency of kidney yang	腎陽虚による生殖機能障害を特徴とする病的変化。
腎氣虛		1.7.286	kidney qi deficiency	a pathological change characterized by depletion of the kidney qi with diminished essence-storing and astringing function	腎気の低下を特徴とする病的変化で、精貯蔵および収斂機能の低下を伴う。
精脫		1.7.287	collapse of essence	a pathological change characterized by depletion and loss of kidney essence that leads to impaired hearing	腎精の低下および喪失を特徴とする病的変化で、難聴に至る。
腎熱		1.7.288	kidney heat	a pathological change characterized by kidney yin depletion with endogenous heat	腎陰の低下を特徴とする病的変化で、内熱を伴う。
腎氣實	腎氣實;腎氣盛	1.7.289	kidney qi excess	repletion of the essential qi of the kidney	腎臓の精気の充実。
腎氣盛	腎氣實;腎氣盛	1.7.289	kidney qi excess	repletion of the essential qi of the kidney	腎臓の精気の充実。
腎實		1.7.290	kidney excess	excessive pathogenic qi in the kidney	腎臓における過剰な邪気。
熱灼腎陰		1.7.291	heat scorching kidney yin	damage to kidney yin by pathogenic heat, usually occurring in the advanced stage of warm heat disease, the same as heat damaging kidney yin	熱邪による腎陰の損傷。通常、温熱病の進行した段階で生じる。heat damaging kidney yinと同義。
膀胱不利		1.7.292	inhibited bladder	a pathological change of the bladder’s function in urination	膀胱の排尿機能における病的変化。
膀胱氣閉		1.7.293	bladder qi block	impairment of bladder qi transformation resulting in inhibited and difficult urination or anuria	膀胱の気化障害で、排尿の阻害および排尿困難あるいは無尿症を生じる。

検索表示（原表示の複数語を分離）	IST原表示	Code	Term	Definition/Description	定義/記述
膀胱濕熱		1.7.294	bladder dampness-heat	accumulation of dampness-heat in the bladder	膀胱における湿熱の蓄積。
膀胱虛寒		1.7.295	bladder deficiency cold	impaired activity of the urinary bladder with cold manifestations due to consumption of kidney yang	膀胱の活動障害で、腎陽の消耗による寒証を伴う。
膀胱失約		1.7.296	bladder retention failure	impaired bladder function resulting in urinary incontinence	膀胱機能障害で、尿失禁を生じる。
寒入血室		1.7.297	cold entering the blood chamber	the pathogenic cold entering the uterus, congeals the liver meridian and impedes blood flow	子宮への寒邪の侵入。肝経を硬直させ、血流を阻害する。
熱入血室		1.7.298	heat entering the blood chamber	the pathogenic heat exploiting deficiency during menstruation or after childbirth to enter the blood chamber and contend with the blood	月経中あるいは産後の虚に乗じて熱邪が子宮に侵入し、血と争う。
熱伏衝任		1.7.299	hidden heat in the thoroughfare and conception vessels	a morbid condition in which pathogenic heat stays in the thoroughfare and conception vessels	熱邪が衝脈および任脈に留まる病的状態。
衝任不調		1.7.300	disharmony of the thoroughfare and conception vessels	impeded flow of qi and blood in the thoroughfare and conception vessels causing dysfunction of these vessels	衝脈および任脈における気および血の流れの阻害で、衝脈および任脈の機能障害を生じる。
衝任不固		1.7.301	insecurity of the thoroughfare and conception vessels	loss of function to control menstruation and prevent uterine bleeding and abortion by the thoroughfare and conception vessels	衝脈および任脈による月経制御ならびに子宮出血と流産の防止機能の喪失。
衝任損傷		1.7.302	damage to the thoroughfare and conception vessels	a pathological change arising when sexual overindulgence, frequent pregnancy or external pathogens cause damage to the thoroughfare and conception vessels	房労、頻繁な妊娠、あるいは外的な病邪による衝脈および任脈の損傷が生じた場合に発生する病的変化。
經隧失職		1.7.303	dysfunction of meridians	a pathological change characterized by impaired flow of qi and blood in the meridians/channels	経絡における気および血の流れの低下を特徴とする病的変化。
經氣逆亂		1.7.304	derangement of meridian qi	a pathological change of meridians/channels in which the adverse flow of meridian/channel qi leads to disordered blood circulation	経絡の病的変化で、経絡の気の逆流により血流の乱れが生じる。
風中血脈		1.7.305	wind striking the blood vessels	deviated eyes and mouth, hemiplegia, or numbness of limbs attributed to wind that strikes the weakened blood vessel, the same as wind attacking the blood vessels	風邪が衰弱した血管を襲うことで生じる口眼喎斜、片麻痺、あるいは四肢の無感覚。wind attacking the blood vesselsと同義。
風火內旋		1.7.306	wind-fire whirling internally	a pathological change of extreme heat engendering wind	極度の熱が風邪を生じさせる病的変化。
風寒束表		1.7.307	wind-cold fettering the exterior	a pathological change attributed to external wind-cold attacking the superficial aspect of the body	外的な風寒による体表面の攻撃に起因する病的変化。
風濕相搏		1.7.308	mutual contention of wind and dampness	a pathological change resulting in muscle aches and joint pains, occurring in wind-dampness contraction, the same as wind and dampness attack	筋肉痛、関節痛を生じる病的変化で、風湿邪に罹ることで起こる。wind and dampness attackと同義。
中風	中風; 卒中(風); 中風病	1.7.309	wind stroke	sudden appearance of hemiplegia, deviated eyes and mouth, and impeded speech attributed to contraction of wind	風邪に罹ることで突然発現する片麻痺、口眼喎斜および発語障害。
卒中	中風; 卒中(風); 中風病	1.7.309	wind stroke	sudden appearance of hemiplegia, deviated eyes and mouth, and impeded speech attributed to contraction of wind	風邪に罹ることで突然発現する片麻痺、口眼喎斜および発語障害。
中風病	中風; 卒中(風); 中風病	1.7.309	wind stroke	sudden appearance of hemiplegia, deviated eyes and mouth, and impeded speech attributed to contraction of wind	風邪に罹ることで突然発現する片麻痺、口眼喎斜および発語障害。
中寒		1.7.310	cold in the middle	(1) middle energizer deficiency-cold arising from insufficiency of yang qi; (2) internal cold remaining in the middle energizer causing damage to yang qi and stagnation of qi and blood	(1) 陽気不足による中焦の寒虚、(2) 中焦にとどまる内寒によって引き起こされる陽気の損傷と気および血の停滞。
中寒		1.7.311	cold stroke	attack of cold directly to the stomach and intestines	胃腸に対する寒邪の直接的な攻撃。
中濕		1.7.312	dampness stroke	(1) a pathological change attributed to dampness attack, either from without or from within; (2) a type of apoplexy related to contraction of dampness, the same as dampness attack	(1) 外部、あるいは内部からの湿邪による攻撃に起因する病的変化、(2) 湿邪に罹ったことに関連する溢血の一種。dampness attackと同義。
燥乾清竅		1.7.313	dryness affecting the clear orifices	a pathological change characterized by dryness-heat affection of the upper part of the body impairing the function of sense organs	上半身の燥熱疾患を特徴とする病的変化で、感覚器の機能を害する。
燥結		1.7.314	dryness accumulation	a pathological change characterized by lack of fluid in the gastrointestinal tract resulting in constipation	胃腸管における津不足を特徴とする病的変化で、便秘を生じる。
熱傷筋脈		1.7.315	heat damaging the sinews	a pathological change characterized by intense heat scorching nutrient yin, depriving the sinews of nourishment and thereby causing contracture, flaccidity or paralysis of limbs	激しい熱邪で養陰が焼かれることを特徴とする病的変化。腱から養分が奪われるため、四肢の拘縮、弛緩、あるいは麻痺が生じる。
熱閉		1.7.316	heat block	pathogenic heat congests in the visceral organs and meridians/channels	内臓および経絡における熱邪の停滞。
熱遏		1.7.317	trapped heat	pathogenic heat is kept in the interior and cannot be discharged	体内に保持された熱邪の放出不能。
熱結		1.7.318	heat accumulation	pathological change characterized by gathering and binding of pathogenic heat in the interior such as in the bladder, in the blood aspect, or in the gastrointestinal tract	体内（膀胱、血、胃腸管など）における熱邪の集合と結合を特徴とする病的変化。
熱鬱		1.7.319	heat depression	a pathological change that arises when persistent depression transforms into heat, the same as heat stagnation	持続的な鬱状態が熱に変化する際に生じる病的変化。heat stagnationと同義。
六鬱		1.7.320	six depressions	a collective term for stagnation of qi, blood, damp, fire, phlegm and food, the same as six stagnations	気、血、湿、火、痰、および食物の停滞の総称。six stagnationsと同義。

検索表示（原表示の複数語を分離）	IST原表示	Code	Term	Definition/Description	定義/記述
衛陽被遏		1.7.321	obstruction of defense yang	a pathological change characterized by the depressed defense qi which fails to warm and protect the body surface	衛気の低下を特徴とする病的変化で、体表の加温、保護機能が不能となる。
營衛不和		1.7.322	nutrient-defense disharmony	the pathological state of spontaneous sweating in an exterior pattern/syndrome, as the defense aspect regulates the excretion of sweat while the nutrient aspect provides fluid for the formation of sweat	表証の自然発汗の病的状態。衛分が発汗を制御するのに対し、営分は汗を生成するための津を供給する。
衛弱營强		1.7.323	weak defense with strong nutrient	the pathological state of spontaneous sweating without fever in an exterior pattern/syndrome	自然発汗を特徴とする病的状態で、表証の発熱を伴わない。
衛强營弱		1.7.324	strong defense with weak nutrient	the pathological state of sweating that occurs only during fever in an exterior pattern/syndrome	発汗の病的状態で、表証の発熱が見られる間のみに生じる。
氣分寒		1.7.325	qi aspect cold	a pathological change characterized by cold in the qi aspect, either due to spleen-stomach yang deficiency with preponderance of yin cold or due to cold pathogen invading the qi aspect	気分における寒を特徴とする病的変化で、陰寒が優勢な脾胃陽虚あるいは気分への寒邪の侵入に起因する。
氣分熱		1.7.326	qi aspect heat	a pathological change characterized by heat in the qi aspect, mostly excess in nature	気分における熱を特徴とする病的変化で、大抵は過剰な状態にある。
衛氣同病		1.7.327	disease of both defense and qi aspects	coexistence of diseases in both the defense and qi aspects	衛分および気分双方における疾患の共在。
衛營同病		1.7.328	disease of both defense and nutrient aspects	coexistence of diseases in both the defense and nutrient aspects	衛分および営分双方における疾患の共在。
熱入血分		1.7.329	heat entering the blood aspect	a pathological change characterized by the pathogenic heat entering the blood aspect, causing hemorrhages, mental disturbances, and even convulsions	血分への熱邪の侵入を特徴とする病的変化。出血、精神障害、さらには痙攣を生じる。
血分熱毒		1.7.330	blood aspect heat toxin	a pathological change characterized by the heat toxin penetrating deeply in the blood aspect, causing high fever with macular eruption, hemorrhages and even mental clouding	血分深部への熱毒の侵入を特徴とする病的変化。斑状皮疹を伴う壮熱、出血、さらには意識混濁を生じる。
氣血兩燔		1.7.331	blazing of both qi and blood	a pathological change characterized by the presence of exuberant pathogenic heat in both the qi and blood aspects causing damage to body fluids and hemorrhages	気分および血分双方における過盛な熱邪の存在を特徴とする病的変化。体液の障害および出血を生じる。
氣營兩燔		1.7.332	blazing of both qi and nutrient	a pathological change characterized by simultaneous existence of exuberant pathogenic heat in the qi and nutrient aspects	気分および営分における過盛な熱邪の同時存在を特徴とする病的変化。
營陰鬱滯		1.7.333	depressed nutrient-yin	a pathological change marked by inhibited movement of nutrient-yin qi, the same as stagnated nutrient-yin	営陰気の動きの阻害を特徴とする病的変化。stagnated nutrient-yinと同義。
傳變		1.7.334	(transmission and) transmutation	development of a disease: transmission from one meridian/channel to another with change in the manifestations	疾患の発現（1つの経絡から別の経絡への伝播で、症状の変化を伴う）。
傳化		1.7.335	(transmission and) transformation	development of disease: transmission from one meridian/channel to another with shift of pathological changes	疾患の発現（1つの経絡から別の経絡への伝播で、病的変化の推移を伴う）。
從化		1.7.336	constitutionally influenced transformation	the process of developing a pattern/syndrome in conformity with the patient’s constitution, e.g., heat pattern/syndrome in a patient of yang constitution, and cold pattern/syndrome in a patient of yin constitution	患者の体質に従った証の発現プロセス(陽体質患者における熱証、陰体質患者における寒証など)。
化熱		1.7.337	transform into heat	transformation into a heat pattern/syndrome in the course of disease	疾患経過における熱証への変化。
熱化		1.7.338	heat formation	the process of forming a heat pattern/syndrome in the course of disease	疾患経過における熱証の形成過程。
化火		1.7.339	transform into fire	transformation into a fire pattern/syndrome in the course of disease	疾患経過における火証への変化。
火化		1.7.340	fire formation	the process of forming a fire pattern/syndrome in the course of disease	疾患経過における火証の形成過程。
化風		1.7.341	transform into wind	transformation into a wind pattern/syndrome in the course of disease	疾患経過における風証への変化。
風化		1.7.342	wind formation	the process of forming a wind pattern/syndrome in the course of disease	疾患経過における風証の形成過程。
化燥		1.7.343	transform into dryness	transformation into a dryness pattern/syndrome due to consumption of body fluids	疾患経過における燥証への変化。
燥化		1.7.344	dryness formation	the process of forming a dryness pattern/syndrome due to fluid consumption	疾患経過における燥証の形成過程。
化寒		1.7.345	transform into cold	transformation into a cold pattern/syndrome in the course of disease	疾患経過における寒証への変化。
寒化		1.7.346	cold formation	the process of forming a cold pattern/syndrome in the course of disease	疾患経過における寒証の形成過程。
化濕		1.7.347	transform into dampness	transformation into a dampness pattern/syndrome in the course of disease	疾患経過における湿証への変化。
濕化		1.7.348	dampness formation	the process of forming a dampness pattern/syndrome in the course of disease	疾患経過における湿証の形成過程。
少陰寒化		1.7.349	lesser yin cold transformation	a pathological change characterized by debilitation of heart-kidney yang with exuberance of yin cold, and usually manifested by aversion to cold with no fever, listlessness and sleepiness, reversal cold of the limbs, lienteric diarrhea and faint fine pulse	過盛な陰寒を伴う心腎陽の衰弱を特徴とする病的変化。通常、発熱を伴わない悪寒、倦怠感および眠気、手足の冷え、消化不良性下痢、および弱い細脈が発現する。
少陰熱化		1.7.350	lesser yin heat transformation	a pathological change characterized by depletion of heart-kidney yin with exuberance of yang heat, and usually manifested by fever, vexation, insomnia, dry mouth and throat, reddened tongue and rapid fine pulse	過盛な陽熱を伴う心腎陰の低下を特徴とする病的変化。通常、発熱、心煩、不眠、口およびのどの渇き、紅舌、および速い細脈が発現する。
暑入陽明		1.7.351	summerheat entering yang brightness	penetration of summerheat into yang brightness, giving rise to intense heat involving the body surface, the heart and the head, and forcing the discharge of sweat, manifested by high fever, thirst, vexation, headache, dizziness, and sweating	陽明への暑邪の侵入。体表、心臓、頭部の激しい暑さを生じ、発汗させる。壮熱、口渇、心煩、頭痛、めまい、および発汗が発現する。

検索表示（原表示の複数語を分離）	IST原表示	Code	Term	Definition/Description	定義/記述
順傳		1.7.352	normal transmission	transmission of a febrile disease following normal sequence, e.g., from the defense aspect to the qi aspect, the same as sequential transmission	通常の順序（衛分から気分へなど）での熱性疾患の伝播。sequential transmissionと同義。
逆傳		1.7.353	reverse transmission	transmission of a febrile disease contrary to the normal sequence, e.g., from the defense aspect directly to the nutrient aspect and blood aspect, instead of by way of the qi aspect, the same as non-sequential transmission	通常の順序に反した（衛分から気分を介さず直接営分および血分へなど）熱性疾患の伝播。non-sequential transmissionと同義。
不傳		1.7.354	non-transmission	no further development of febrile disease	熱性疾患のさらなる発現がない。
經盡		1.7.355	ceasing to transmit	exogenous febrile disease stops progressing and begins recovering when it reaches a certain meridian/channel or certain stage of development	特定の経絡あるいは特定の段階に達した際の、外因性熱性疾患の進行停止と回復開始。
再經		1.7.356	transmit to another meridian	the process of a cold-damage disease in which one meridian/channel syndrome passes into another meridian/channel syndrome while the former persists	傷寒の経過。1つの経絡証が別の経絡へと移行し、元の経絡証が持続する。
越經傳		1.7.357	skipping meridians transmission	transmission of a cold-damage disease from one meridian/channel to another with skipping of one or more meridians/channels, e.g., transmission from greater yang to lesser yang with yang brightness skipped over	1つの経絡から別の経絡への傷寒の伝播。1つ以上の経絡を飛び越えて伝播する（太陽から陽明を飛び越えて少陽へなど）。
循經傳		1.7.358	sequential meridians transmission	transmission of a cold-damage disease from one meridian/channel to another, by the order of greater yang, yang brightness, lesser yang, greater yin, lesser yin and reverting yin	1つの経絡から別の経絡への傷寒の伝播。太陽、陽明、少陽、太陰、少陰、厥陰の順に伝播する。
直中		1.7.359	direct strike/stroke	attack of an exogenous pathogen directly on the three yin meridians instead of transmission from the yang meridians, the same as direct attack	陽経からの伝播ではなく、外因性病邪による三陽経への直接的な攻撃。direct attackと同義。
並病		1.7.360	overlap of diseases	overlapping of two meridian/channel diseases, in which they appear in succession and then coexist	2つの経絡疾患の重複。連続して発現し、共在する。
合病		1.7.361	combination of diseases	combination of two or more meridian/channel diseases appearing at the same time	複数の経絡疾患の同時発現。
順證		1.7.362	favorable pattern/syndrome	a pattern/syndrome which indicates a positive prognosis	良好な予後を示す証。
逆證		1.7.363	unfavorable pattern/syndrome	a pattern/syndrome which indicates a negative positive prognosis	不良な予後を示す証。
其他		**1.8.0**	**Others**		
運氣學		1.8.1	theory of five circuits and six qi	the doctrine that aims at studying the rule of climatic changes and its relation to the occurrence of disease in terms of the five circuits and six qi	気候変化の規則および疾患の発生との関連を、五運と六気の観点から研究する学問。
五運		1.8.2	five circuits	a collective name for wood, fire, earth, metal and water circuits	木運、火運、土運、金運、水運の総称。
六氣		1.8.3	six qi	six normal climatic phenomena: wind, cold, summerheat, dampness, dryness and fire (heat)	6つの正常な気象現象：風、寒、暑、湿、燥、火（熱）。
歳運		1.8.4	circuit of year	the circuit qi that controls the meteorological features and changes of a given year as well as the corresponding prevalence of disease	特定の年の気象学的特徴と変化、および対応する疾患の流行をつかさどる運気。
司天		1.8.5	(qi) controlling heaven	the guest qi that controls the climatic changes in the first half of the year	1年の上半期の気候変化をつかさどる客気。
在泉		1.8.6	terrestrial effect	the guest qi that controls the climatic changes in the latter half of the year	1年の下半期の気候変化をつかさどる客気。
合陰		1.8.7	midnight	the time when nutrient qi and defense qi meet in the visceral organs	内臓で営気と衛気が交わる時。
甲子		1.8.8	sixty-year cycle	combination of the hevenly stems and earthly branches used to designate years, months, days and hours	十干十二支の組み合わせで、年、月、日、時間の指定に使用する。
干支		1.8.9	stems and branches	a collective name for the ten heavenly stems and the twelve earthly branches, both used as serial numbers	十干十二支の総称。いずれも連番として使用される。
主氣		1.8.10	dominant qi	the qi that controls the regular seasonal changes of the climate	気候の正常な季節的変化をつかさどる気。
客氣		1.8.11	guest qi	the qi that controls the seasonal changes of the climate in a particular year	特定の年における気候の季節的変化をつかさどる気。
間氣		1.8.12	intermediate qi	that part of guest qi, other than the qi controlling the heaven and the qi with terrestrial effect, called intermediate qi because of its existence between the heaven and earth	客気の一部。司天および在泉以外の気。天と地の間に存在することから間気と呼ばれる。
歳會		1.8.13	annual congruence	coincidence of the circuit qi of the year with the earthly branch in terms of the five phases, happening in eight years of a sixty-year cycle	年の運気と支が五行に関して一致すること。60年周期の8年で起こる。
天符		1.8.14	coincidence of heavenly qi	coincidence of the circuit qi of the year with the qi controlling the heaven in terms of the five phases, occurring in a cycle of sixty years	年の運気と司天が五行に関して一致すること。60年周期で起こる。
平氣		1.8.15	normal circuit qi	the circuit qi that is neither excessive nor insufficient, rarely causing disease	過剰でも不足でもない運気。疾患を引き起こすことはほとんどない。
天年		1.8.16	natural life span	human life expectancy	人の寿命。
三陰三陽		1.8.17	three yin and three yang	a collective term for reverting yin, lesser yin, greater yin, lesser yang, yang brightness and greater yang	厥陰、少陰、太陰、少陽、陽明、太陽の総称。
開闔樞		1.8.18	opening, closing and pivot	the functional characteristics of three yin and three yang: opening referring to outward actions, closing referring to inward actions, while pivot referring to the central role	三陰三陽の機能的特徴。開は外に向かう作用、閉は内に向かう作用、樞は中心的役割を指す。
太陽		1.8.19	greater yang	(1) referring to cold qi in the theory of five circuits and six qi; (2) referring to the bladder meridian and small intestine meridian in the meridian theory	(1) 運気学における寒気、(2) 経絡学説における膀胱経および小腸経。
陽明		1.8.20	yang brightness	(1) referring to dryness qi in the theory of five circuits and six qi; (2) referring to the large intestine meridian and stomach meridian in the meridian theory	(1) 運気学における燥気、(2) 経絡学説における大腸経および胃経。
少陽		1.8.21	lesser yang	(1) referring to fire qi in the theory of five circuits and six qi; (2) referring to the triple energizer meridian and gallbladder meridian in the meridian theory	(1) 運気学における火気、(2) 経絡学説における三焦経および胆経。

検索表示（原表示の複数語を分離）	IST原表示	Code	Term	Definition/Description	定義/記述
太陰		1.8.22	greater yin	(1) referring to dampness qi in the theory of five circuits and six qi; (2) referring to the lung meridian and spleen meridian in the meridian theory	(1) 運気学における湿気、(2) 経絡学説における肺経および脾経。
少陰		1.8.23	lesser yin	(1) referring to heat qi in the theory of five circuits and six qi; (2) referring to the heart meridian and kidney meridian in the meridian theory	(1) 運気学における熱気、(2) 経絡学説における心経および腎経。
厥陰		1.8.24	reverting yin	(1) referring to wind qi in the theory of five circuits and six qi; (2) referring to the pericardium meridian and liver meridian in the meridian theory	(1) 運気学における風気、(2) 経絡学説における心包経および肝経。
保命之主		1.8.25	principals of life preservation	the main principals for the maintenance of health and enhancement of life expectancy for individual constitutions	各体質に関する、健康維持および寿命延長のための主要原則。
吸聚之氣		1.8.26	inhale-gathering qi	the qi that promotes health for the greater yang person	太陽人の健康を増進する気。
陰淸之氣		1.8.27	yin-cool qi	the qi that promotes health for the lesser yang person	少陽人の健康を増進する気。
呼散之氣		1.8.28	exhale-dispersing qi	the qi that promotes health for the greater yin person	太陰人の健康を増進する気。
陽煖之氣		1.8.29	yang-warm qi	the qi that promotes health for the lesser yin person	少陰人の健康を増進する気。
水穀代謝		1.8.30	water-food metabolism	the processes by which food and water are taken in by the spleen and passed out by the kidney	水穀が脾臓によって取り込まれ、腎臓によって排出される過程。
氣液代謝		1.8.31	qi-humor metabolism	processes by which qi and humor are inhaled by the liver and exhaled by the lung	気および液が肝臓によって吸い込まれ、肺によって吐き出される過程。
升降緩束		1.8.32	ascending-descending-congested-uncongested	movement of qi to control water-grains metabolism and qi-humor metabolism	水穀の代謝および気液の代謝をつかさどる気機。
完實無病		1.8.33	healthy state	healthy state without diseases or symptoms, a singular expression in Four Constitution Medicine	疾患および症状のない健康な状態。四象医学特有の表現。
素證		1.8.34	ordinary symptom	symptoms occurring in association with the individual constitution	個々の体質に関連して発現する症状。
性情		1.8.35	temperament	the distinct nature and character of an individual	個人の明白な性質および特徴。
性質材幹		1.8.36	skill and talent	special aptitude or faculty, expertness or ability to enhance social relationships	特別な素質または能力、社会的関係を強化する熟達性または能力。
容貌詞氣		1.8.37	appearance and style of talking	one of the criteria for identifying the type of constitution, e.g., neat appearance, natural speech and shrewd behavior of a lesser yin person	体質を識別するための基準の1つ（例：少陰人のきちんとした容貌、自然な話し方、賢明な振る舞い）。
體形氣像		1.8.38	physical appearance	one of the criteria for identifying the type of constitution, e.g., developed hips for the sitting posture and a weak chest for the self-embracing posture in the lesser yin person	体質を識別するための基準の1つ（例：少陰人の座位での発達した臀部、自己抱擁時のひ弱い胸部）。
事心身物		1.8.39	activity-mind-body-matter	basic factors of Four Constitution Medicine to understand all nature	四象医学で万物を理解するための基本的要素。
診斷學		**2.0.0**	**DIAGNOSTICS**		
診斷		2.0.1	diagnosis	determination of the nature of a diseased condition; identification of a disease by careful investigation of its symptoms and history; also, the opinion (formally stated) resulting from such investigation	疾病状態の性質を判断し、その症状や病歴を注意深く検討することにより疾病を識別すること。また、このような検討から得られた（正式な）見解。
診法		2.0.2	diagnostic method	the basic method of collecting data to determine the disease condition	疾病状態を判断するための基本的なデータ収集法。
症狀		2.0.3	symptom	a (bodily or mental) phenomenon, circumstance, or change of condition arising from and accompanying a disease or other pathlogical cindition, and constituting an indication or evidence of it; a characteristic sign of a particular disease. Especially, in modern use, a subjective indication, perceptible to the patient	疾病やその他の病的状態に起因し、これに付随して起こり、適応症またはその根拠を構成する（身体または精神の）現象、環境、または状態の変化。特定の疾病の特徴的な徴候。とくに、現代では、患者が認識できる主観的な適応症に用いる。
體徵	體徵;徵候	2.0.4	sign	an objective evidence or indication of disease, especially observed by the physician	とくに医師の観察による、疾病の客観的な根拠または適応症。
徵候	體徵;徵候	2.0.4	sign	an objective evidence or indication of disease, especially observed by the physician	とくに医師の観察による、疾病の客観的な根拠または適応症。
疾病		2.0.5	disease	a condition of being (more or less seriously) out of health; illness, sickness	健康から逸脱した状態（重篤度は問わない）。疾患、病気。
辨病論治		2.0.6	disease identification and treatment	identification of the category of a disease and its treatment	疾病およびその治療のカテゴリーを識別すること。
證		2.0.7	pattern/syndrome	(1) diagnostic conclusion of the pathologocal changes at a certain stage of a disease, including the location, cause, and nature of the disease as well as the trend of development; (2) conditions suggesting appropriate treatment; (3) condition specific to the individual	（1）疾病のある段階における病的変化の診断の結論のことで、疾病の部位、原因、性質および発症傾向を含む、（2）適切な治療を示唆する状態、（3）個人に特異的な状態。
證型		2.0.8	pattern/syndrome type	a common pattern/syndrome mode with a standard name	標準名で表される共通の証型。
診籍		2.0.9	case record	traditionally used record of a patient’s medical history, diagnosis and treatment	従来用いられる患者の病歴、診断、治療の記録。
揆度奇恒		2.0.10	assessment of the normal and abnormal	determining the extent to which an individual is exhibiting abnormal characteristics which may be indicative of a disease or pathological condition	人に疾病または病的状態を示すような異常な特徴がどの程度まで現れているかを判断すること。
司外揣內		2.0.11	judging the inside from observation of the outside	making judgment on the condition inside the body based on the signs observed from the outside	体外から観察される徴候に基づいて、体内の状態を判断すること。
四診合參		2.0.12	correlation of all four examinations	comprehensive consideration of the data obtained from the four examinations (inspection, listening and smelling, inquiry, and palpation) for making the diagnosis	四診（望診、聞診［音声を聞く、臭いを嗅ぐ］、問診、切診）で得たデータを総合的に検討して診断すること。
辨證		2.0.13	pattern identification/syndrome differentiation	the process of overall analysis of clinical data to determine the location, cause and nature of a patient’s disease and achieving a diagnosis of a pattern/syndrome, also called pattern differentiation	患者の疾病の部位、原因、性質を判断するために、臨床データを全体的に分析する過程で、これにより証の診断が達成される。pattern differentiationとも呼ばれる。

検索表示（原表示の複数語を分離）	IST原表示	Code	Term	Definition/Description	定義/記述
四診		2.0.14	four examinations	a collective term for inspection, listening and smelling, inquiry, and palpation	望診、聞診（音声を聞く、臭いを嗅ぐ）、問診、切診の総称。
八綱		2.0.15	eight principles	guiding principles of pattern identification/syndrome differentiation, that is, yin and yang, exterior and interior, cold and heat, deficiency and excess	弁証論治を導く原理。すなわち、 陰・陽、表・裏、寒・熱、虚・実の8種類。
望診		**2.1.0**	**Inspection**		
望診		2.1.1	inspection	one of the four diagnostic examinations, including observing the patient's mental state, facial expression, complexion, physical condition, condition of the tongue, secretions and the distribution of superficial venules of the infant's fingers	四診の1つで、患者の精神状態、顔の表情や色、身体の状態、舌の状態、分泌物、小児の指紋（表層の静脈）分布の観察を含む。
望神		2.1.2	inspection of the vitality	inspection of the state and vitality, especially mental and physical activities such as consciousness, thinking, facial expression, speech, and response to external stimuli	状態および生命活動、とくに、意識、思考、顔の表情、発語、外的刺激に対する反応などの精神・身体活動の望診。
得神		2.1.3	presence of vitality	the general state marked by high spirit and mental and physical exuberance with prompt and appropriate responses, indicating that the healthy qi is not damaged and improvement from a pathological condition can be expected	神が高く、精神・身体活動が盛んで、迅速で適切な反応を特徴とする全身状態であり、正気が損なわれることはなく、病的状態からの改善が期待できることを示す。
失神		2.1.4	loss of vitality	the general state marked by indifference, low spirit and impaired mental and physical functions with sluggish and inappropriate responses, indicating the existence of a serious pathological condition	無関心で神が低く、精神・身体機能が損なわれ、緩慢で不適切な反応を特徴とする全身状態であり、重篤な病的状態が存在することを示す。
少神		2.1.5	lack of vitality	the general state marked by indifference, listlessness and low spirit	無関心、無気力、低い神を特徴とする全身状態。
假神		2.1.6	false vitality	transient improvement in spirit in a critical case, often indicating approaching death	危篤状態の患者に一過性の神の改善がみられることで、しばしば臨終の前兆を示す。
昏蒙		2.1.7	mental confusion	clouding of consciousness with sleepiness, but responsive to calling	眠気を伴う意識混濁であるが、呼びかけには反応する。
昏厥		2.1.8	fainting	sudden loss of consciousness, usually of short duration, sometimes accompanied by cold limbs, also called syncope	突然の意識消失。通常は短時間で回復し、四肢の冷えを伴うことがある。失神（syncope）とも呼ばれる。
譫妄		2.1.9	delirium	a mental disturbance characterized by confusion, disordered speech and hallucinations	錯乱、発語障害、幻覚を特徴とする精神障害。
煩躁		2.1.10	agitation	vexation followed by irritability, irascibility, and restlessness	心煩に続き、いらいらが生じ、怒りっぽく、落ち着きがなくなる。
望色		2.1.11	inspection of the complexion	observation of the patient's skin color, particularly the color of the face	患者の皮膚の色、とくに顔色の観察。
面色		2.1.12	(facial) complexion	color and luster of the face	顔の色およびつや。
面色淡白		2.1.13	pale white complexion	a colorless complexion, often indicating blood deficiency or profuse loss of blood	血色のない顔のことで、しばしば血虚または大量出血を示す。
面色蒼白		2.1.14	pale complexion	a white complexion with a hint of blue or gray, a complexion often caused by yang collapse or exuberance of cold	青色または灰色を帯びた白色の顔。しばしば亡陽 または盛んな寒により生じる。
面色㿠白		2.1.15	bright pale complexion	a white complexion with puffiness, often seen in cases of yang deficiency	白色のむくんだ顔で、しばしば陽虚の場合に生じる。
面黑		2.1.16	darkish complexion	dark discoloration of the face, often occurring in kidney deficiency, cold pattern/syndrome, water retention or blood stasis	暗黒色の顔で、しばしば腎虚、寒証、水停または血瘀の場合に生じる。
面紅		2.1.17	reddened complexion	a complexion redder than normal, indicating the presence of heat	正常より赤みを帯びている顔で、熱の存在を示す。
面靑		2.1.18	bluish complexion	bluish discoloration of the face, often occurring in cold pattern/syndrome, pains, qi stagnation, blood stasis or convulsions	青白い顔で、しばしば寒証、疼痛、気滞、血瘀または抽搐の場合に生じる。
面黃		2.1.19	yellow complexion	yellow discoloration of the face, generally suggesting spleen deficiency or accumulation of dampness that causes dysfunction of the spleen	黄色い顔で、一般に、脾虚または脾機能障害を引き起こす湿の蓄積を示唆する。
面色萎黃		2.1.20	sallow complexion	yellowish withered complexion, which usually occurs in cases of spleen qi deficiency	黄色みを帯び、干からびた顔で、通常は脾気虚の場合に生じる。
面浮		2.1.21	puffy face	a soft swollen face, usually indicating a deficiency condition	柔らかく腫れぼったい顔で、通常は虚の状態を示す。
主色		2.1.22	governing complexion	normal natural color of the skin	正常で自然な皮膚の色。
客色		2.1.23	visiting complexion	a normal complexion influenced by a change of climate	気候の変動の影響を受ける正常な顔色。
病色		2.1.24	morbid complexion	abnormal color of the face caused by disease	疾病による異常な顔色。
善色		2.1.25	benign complexion	bright and lustrous complexion indicating a favorable prognosis, also the same as healthy complexion	明るくつやのある顔で、予後良好を示す。健康な顔色（healthy complexion）と同義。
惡色		2.1.26	malign complexion	dark and gloomy complexion indicating an unfavorable prognosis, also the same as unhealthy complexion	暗く陰うつな顔で、予後不良を示す。不健康な顔色（unhealthy complexion）と同義。
眞臟色		2.1.27	true visceral color	color reflected in the face indicating exhaustion of the genuine qi of internal organs	内臓の真気の消耗を示す、顔に表れた色。
胕腫		2.1.28	pitting edema	edema associated with pitting of the skin, which resolves only slowly, usually occurring on the instep	皮膚の圧痕浮腫のことで、消失は非常に緩やかである。通常、足の甲に生じる。
肌膚甲錯		2.1.29	encrusted skin	dried, roughened and scaling skin, indicating chronic blood stasis	乾燥して魚鱗のようにカサカサした皮膚のことで、慢性の血瘀を示す。
抽搐	抽搐;<br>瘛瘲	2.1.30	convulsions	sudden, violent uncontrollable movements of the limbs caused by contraction of muscles	筋収縮による四肢の突然の激しい制御不能な動き。
瘛瘲	抽搐;<br>瘛瘲	2.1.30	convulsions	sudden, violent uncontrollable movements of the limbs caused by contraction of muscles	筋収縮による四肢の突然の激しい制御不能な動き。
便血	便血;<br>圊血	2.1.31	bloody stool	loss of blood through anus, with stool	排便に伴う肛門からの出血。
圊血	便血;<br>圊血	2.1.31	bloody stool	loss of blood through anus, with stool	排便に伴う肛門からの出血。
遠血		2.1.32	distal bleeding	bleeding far from the anus, generally referring to bleeding from upper digestive tract	肛門から遠い部位での出血で、一般に上部消化管出血（bleeding from upper digestive tract）を指す。

検索表示（原表示の複数語を分離）	IST原表示	Code	Term	Definition/Description	定義/記述
近血		2.1.33	proximal bleeding	passing fresh blood before or during defecation, indicating anal or rectal hemorrhage	排便前または排便時に鮮血が流れることで、肛門または直腸からの出血を示す。
望指紋		2.1.34	inspection of finger venules	a diagnostic method for infants, based on observation of the superficial venules on the palmar side of the index finger	小児に用いる診法で、示指の掌側にある指紋の観察に基づく。
三關		2.1.35	three bars	a collective term for the three segments of the index finger used for measuring the extension of the visible venules, i.e., “wind bar”, “qi bar” and “life bar”, also known as three gates	皮膚表面に浮き出た静脈の伸展を測定するために用いる示指の3つの節、すなわち「風関」、「気関」、「命関」の総称。three gatesとしても知られる。
風關		2.1.36	wind bar	the proximal segment of the index finger in the inspection of venules, also known as wind gate	望指紋における示指の近位節。wind gateとしても知られる。
氣關		2.1.37	qi bar	the middle segment of the index finger in the inspection of venules, also known as qi gate	望指紋における示指の中間節。qi gateとしても知られる。
命關		2.1.38	life bar	the distal segment of the index finger in the inspection of venules, also known as life gate	望指紋における示指の遠位節。life gateとしても知られる。
舌診		2.1.39	tongue diagnosis	inspection of the size, shape, color and moisture of the tongue proper and its coating	舌と舌苔の大きさ、形状、色、湿潤の望診。
望舌		2.1.40	inspection of the tongue	examination of the tongue body and its coating	舌体および舌苔の検査。
舌象		2.1.41	tongue manifestation	changes in the appearance of the tongue, relating to changes in the color and form of the tongue body and its coating	舌体および舌苔の色や形状の変化に関連する舌の外観の変化のこと。
舌尖		2.1.42	tip of the tongue	the anterior end of the tongue	舌の先端部。
舌邊		2.1.43	margins of the tongue	the lateral borders of the tongue	舌の両側部。
舌中	舌中; 舌心	2.1.44	center of the tongue	the central part of the tongue	舌の中央部。
舌心	舌中; 舌心	2.1.44	center of the tongue	the central part of the tongue	舌の中央部。
舌根	舌根; 舌本	2.1.45	root of the tongue	the part of the tongue that is attached basally to the bone	骨に付着した舌の根元の部分。
舌本	舌根; 舌本	2.1.45	root of the tongue	the part of the tongue that is attached basally to the bone	骨に付着した舌の根元の部分。
舌體	舌體; 舌質	2.1.46	tongue body	the musculature and vascular tissue of the tongue, also referring to tongue substance	舌の筋肉・脈絡組織。tongue substanceとも言う。
舌質	舌體; 舌質	2.1.46	tongue body	the musculature and vascular tissue of the tongue, also referring to tongue substance	舌の筋肉・脈絡組織。tongue substanceとも言う。
舌色		2.1.47	tongue color	color of the tongue body, which reflects the condition of qi, blood and the visceral organs	舌体の色のことで、気、血、臓器の状態を反映する。
舌神		2.1.48	tongue spirit	the general vitality of the tongue manifest in its luxuriance or witheredness	舌象の全般的な生命活動のことで、潤沢（栄）か干からびているか（枯）で反映される。
榮枯老嫩		2.1.49	luxuriant, withered, tough and tender-soft	a luxuriant tongue is moistened and fresh red in color; a withered tongue is dull, dark, dry and shriveled; a tough tongue is firm with rough texture; a tender-soft tongue is delicate with fine texture	栄の舌は湿潤で鮮紅色、枯の舌はつやがなく色が暗で、乾燥してしわがある。老の舌は硬くてきめが粗く、嫩の舌は柔らかくてきめが細かい。
淡白舌		2.1.50	pale tongue	a tongue less red than normal, indicating qi and blood deficiency or presence of deficiency-cold	正常より赤みの少ない舌で、気虚・血虚または虚寒の存在を示す。
淡紅舌		2.1.51	pale red tongue	a tongue of normal color	正常な色の舌。
紅舌		2.1.52	red tongue	a tongue redder than normal, indicating presence of heat	正常よりも赤みを帯びた舌で、熱の存在を示す。
青舌		2.1.53	blue tongue	a tongue of bluish color, indicating congealing cold and static blood	青みを帯びた舌で、寒凝血瘀を示す。
紫舌		2.1.54	purple tongue	a tongue purple in color, indicating stagnant circulation of qi and blood	紫色の舌で、気および血の循環の停滞を示す。
青紫舌		2.1.55	bluish purple tongue	a cyanotic tongue, indicating blood stasis or heat toxin in the nutrient-blood	チアノーゼを起こしている舌で、血瘀または営血中の熱毒を示す。
絳舌		2.1.56	crimson tongue	a tongue deep red in color, indicating intense heat	深紅色の舌で、激しい熱を示す。
胖大舌		2.1.57	enlarged tongue	a tongue that is larger than normal, pale in color and delicate, usually bearing dental indentations on the margin	正常より大きく、淡白な色のきめ細かな舌で、通常は舌辺に歯痕がみられる。
腫脹舌		2.1.58	swollen tongue	a large and bulging tongue that makes the mouth full or difficult to open	大きく腫れぼったい舌。これが口腔を満たし、口があかなくなる。
瘦薄舌		2.1.59	thin tongue	a tongue thinner than normal	正常より薄い舌。
點刺舌		2.1.60	spotted tongue	a tongue with red, white or black spots as well as thorn-like protrusions on its surface	舌面に赤、白、黒の斑点および棘状の隆起が生じる舌。
芒刺舌		2.1.61	prickly tongue	a tongue with thorn-like protrusions on its surface	舌面に棘状の隆起が生じる舌。
齒痕舌		2.1.62	teeth-marked tongue	a tongue with dental indentations on its margin	舌辺に歯痕が残る舌。
裂紋舌		2.1.63	fissured tongue	a tongue with fissures on its surface	舌面上に亀裂が生じる舌。
光剝舌		2.1.64	peeled tongue	a tongue that has lost its normal coating, giving the appearance of having been peeled	正常な舌苔が失われ、剥れ落ちたような外観の舌。
鏡面舌		2.1.65	mirror tongue	a completely smooth tongue free of coating, like a mirror	舌苔がまったくなく、鏡のように完全にてかてかしている舌。
地圖舌		2.1.66	geographical tongue	a tongue with irregular shedding of the coating and distinct demarcation between the shed and unshed areas, looking like a map	舌苔に不規則な剥落がみられ、剥落している部分と剥落していない部分の境界が明瞭で、地図のように見える舌。
舌衄		2.1.67	spontaneous bleeding of the tongue	bleeding from the tongue not due to traumatic injury	外傷によらない舌からの出血。
强硬舌		2.1.68	stiff tongue	a tongue that is stiff, moves sluggishly, and inhibits speech	舌が強直して動きがのろく、ろれつが回らない。
痿軟舌		2.1.69	limp wilting tongue	a tongue that is flabby and cannot move easily	舌が弛緩して動かしにくい。
顫動舌		2.1.70	trembling tongue	a tongue that involuntarily trembles as it moves	舌を動かすと不随意にふるえる。
歪斜舌		2.1.71	deviated tongue	a tongue that inclines to one side when extended	舌を伸ばしたときに左右いずれかに傾く。
短縮舌		2.1.72	contracted tongue	a tongue that cannot be fully extended from the mouth and appears to be contracted	舌が縮まっているようで口外に伸ばせない。

検索表示（原表示の複数語を分離）	IST原表示	Code	Term	Definition/Description	定義/記述
吐弄舌		2.1.73	protruded agitated tongue	a pathological condition in which the tongue hangs out of the mouth and moves in a circular motion whereby the tip is extended from and retracted back into the mouth, or licks the lips	舌を口から垂らし、舌先を伸ばしたり引っ込めたりしながら、円運動や唇をなめる動作を行う病的状態。
舌縱		2.1.74	protracted tongue	a tongue that is habitually extended out of the mouth and cannot be retracted	舌を伸ばしたまま、口内に戻せない。
舌乾		2.1.75	dry tongue	a tongue lacking moisture and rough to the touch	舌の湿潤性がなく触るとざらざらしている。
痲痹舌		2.1.76	paralyzed tongue	a numb tongue unable to move	舌がしびれて動かない。
舌態		2.1.77	motility of the tongue	the ability of the tongue to move spontaneously	舌を自発的に動かす能力。
舌形		2.1.78	form of the tongue	referring to the shape, luxuriance and witheredness, toughness and softness, thinness and swelling, spots, fissures, and dental indentations	形状、栄枯、老嫩、厚いか薄いか、腫脹の有無、斑点、亀裂、歯痕を指す。
舌苔		2.1.79	tongue fur	a layer of moss-like material covering the tongue, also called tongue coating	舌面上の一層の苔状物のこと。舌垢（coating）とも呼ばれる。
苔色		2.1.80	fur color	color of the tongue coating, white, yellow, gray or black, and in rare cases, green	舌苔の色。白、黄、灰または黒、まれに緑がある。
苔質		2.1.81	texture of fur	characters of tongue coating, including thickness, moisture, sliminess, roughness etc.	厚さ、湿潤性、粘性（ねばねば度）、ざらつきなどの舌苔の性質。
白苔		2.1.82	white fur	tongue coating white in color	白色の舌苔。
白砂苔		2.1.83	white sandy fur	white dry and thick tongue coating like a layer of sand	一層の砂で覆われたような白色で乾燥した厚い舌苔。
黃苔		2.1.84	yellow fur	tongue coating yellow in color	黄色の舌苔。
黑苔		2.1.85	black fur	tongue coating black in color, indicating either excessive cold or extreme heat in the interior	黒色の舌苔で、体内の過剰な寒または極度の熱を示す。
灰苔		2.1.86	gray fur	tongue coating gray in color with similar clinical significance as black fur	灰色の舌苔で、臨床的意義は黒苔とほぼ同じ。
綠苔		2.1.87	greenish fur	tongue coating green in color	緑色の舌苔。
薄苔		2.1.88	thin fur	a tongue coating through which the underlying tongue surface is faintly visible	舌苔の下に舌面がかすかに見える。
厚苔		2.1.89	thick fur	a tongue coating through which the underlying tongue surface is not visible	舌苔の下に舌面がまったく見えない。
霉醬苔		2.1.90	rotten-curdy fur	a reddish thick tongue coating with black and yellow tinge	黒色および黄色みがかった赤色の厚い舌苔。
潤苔		2.1.91	moist fur	a moderately moistened tongue coating	中等度に湿った舌苔。
燥苔		2.1.92	dry fur	a tongue coating that looks dry and feels dry to the touch	乾燥したように見え、触るとざらざらする舌苔。
燥裂苔		2.1.93	dry and cracked fur	a tongue coating that lacks moisture and develops cracks	湿り気がまったくなく、ひび割れがある舌苔。
滑苔		2.1.94	slippery fur	a moist tongue coating with excessive fluid, feels slippery	過剰な水分で湿り、つるつるした感触の舌苔。
膩苔		2.1.95	slimy fur	a dense, turbid, slimy tongue coating, sticking on the tongue, hard to wipe off, also known as greasy fur	顆粒が密で濁りがあるねばねばした舌苔で、舌にこびりつき、剥離しにくい。greasy furとしても知られる。
粘膩苔		2.1.96	sticky slimy fur	a dense, turbid, slimy tongue coating, covered with a layer of thick turbid mucus	顆粒が密で濁りがあるねばねばした舌苔で、一層の厚い濁った粘液で覆われる。
腐苔		2.1.97	curdy fur	a tongue coating consisting of coarse granules like bean dregs, easy to wipe off	豆腐かすのようなまばらな顆粒の舌苔で、剥離しやすい。
剝苔	剝苔;<br>舌苔脫落	2.1.98	peeling fur	complete or partial peeling of the tongue coating	完全または部分的に剥離できる舌苔。
舌苔脫落	剝苔;<br>舌苔脫落	2.1.98	peeling fur	complete or partial peeling of the tongue coating	完全または部分的に剥離できる舌苔。
類剝苔		2.1.99	exfoliated fur	peeling of the tongue coating leaving an unsmooth tongue surface as if covered with regeneration of coating granules	舌苔が剥離でき、舌面が滑らかでなく新しい顆粒ができているように見えるもの。
染苔		2.1.100	stained fur	a tongue coating that is stained, often by food or medicine	しばしば飲食物や薬物で染色される舌苔。
舌卷		2.1.101	curled tongue	a tongue formed into a curved shape and drawn back, inhibiting speech	舌が丸まって口腔内に引っ込み、発語できない。
舌卷囊縮		2.1.102	curled tongue and retracted testicles	the formation of the tongue into a curl and the withdrawing of the testicles into the body, seen in critical cases	舌が丸まり、睾が萎縮して体内に引っこむことで、危篤状態の患者でみられる。
舌下絡脈		2.1.103	sublingual collateral vessels	the vein under the tongue on either side of the frenulum	舌下の舌小帯の両側に位置する静脈。
聞診		**2.2.0**	**Listening and Smelling Examination**		
聞診		2.2.1	listening and smelling examination	one of the four diagnostic examinations, specifically listening and smelling	四診の一つで、とくに音を聞き、臭いを嗅いで診断する。
聞聲音		2.2.2	listening to sounds	listening to the patient's voice, sounds of breathing, cough, vomiting, etc. for determining the cold, heat, deficiency or excess nature of disease as a diagnostic method	寒熱、虚実といった病性を判断するために、患者の声、呼吸、咳、嘔吐などの音を聞く診法。
語聲重濁		2.2.3	deep turbid voice	a voice that is deep and muffled or otherwise unclear	太くこもっているまたはそれ以外のはっきり聞き取れない声。
語聲低微		2.2.4	faint low voice	a voice that is faint and low, scarcely audible	ほとんど聞き取れないほどのかすかな低い声。
語言謇澁		2.2.5	sluggish speech	speech distorted by sluggish movement of the tongue	舌の緩慢な動きにより言語が乱れること。
舌謇		2.2.6	sluggish tongue	a tongue lacking normal agility	正常な敏捷さがない舌。
失音		2.2.7	loss of voice	hoarseness of the voice or total failure of the voice	声がかすれる、またはまったく声が出ない。
嘶嗄		2.2.8	hoarseness	a low harsh or husky quality of the voice	低く耳障りなまたはしゃがれた声。
鄭聲		2.2.9	muttering	unconscious murmuring haltingly with frequent repetitions	無意識に、とぎれとぎれに何度も繰り返しつぶやくこと。
譫語		2.2.10	delirious speech	disordered speech in delirium	譫妄における発語障害。
獨語		2.2.11	soliloquy	talking to oneself regardless of the presence of others	他人がいてもいなくても独り言を言うこと。

検索表示（原表示の複数語を分離）	IST原表示	Code	Term	Definition/Description	定義/記述
錯語		2.2.12	disordered speech	a type of dysphasia in which the patient frequently employs wrong words or uses words in incorrect and senseless combinations	失語症の一種で、患者は頻繁に間違った単語を使ったり、不正確で意味のない単語の組み合わせを用いたりする。
囈語		2.2.13	sleep talking	talking in one’s sleep, the same as somniloquy	睡眠中に話すこと。寝言（somniloquy）と同義。
狂言		2.2.14	manic raving	mentally-deranged wild talk	精神が錯乱し、でたらめを言うこと。
太息		2.2.15	sighing	taking deep breaths with long, audible expiration	周りに聞こえるような長い呼気を伴う深呼吸。
噴嚏		2.2.16	sneezing	sudden violent audible expulsion of breath when the nose is stimulated	鼻が刺激され、突然周りに聞こえるほど激しく、呼気が放出されること。
呵欠		2.2.17	yawning	involuntary intake of breath through the open mouth, usually as a reaction to fatigue	口をあけて行う不随意な吸息で、通常は疲労に対する反応。
喘促		2.2.18	panting	rapid and difficult breathing	速く困難な呼吸。
喘鳴		2.2.19	wheezing dyspnea	difficult breathing with a whistling sound	ヒューヒューという音を伴う呼吸困難。
痰鳴		2.2.20	phlegm rale	an abnormal breathing sound produced by the presence of phlegm in the airways	気道内の痰の存在により生じる異常な呼吸音。
咳逆	咳逆;咳逆上氣	2.2.21	cough with dyspnea	cough with counterflow of qi in the airways, also known as cough with qi reflux	気道内の気の逆流に伴う咳。cough with qi refluxとしても知られる。
咳逆上氣	咳逆;咳逆上氣	2.2.21	cough with dyspnea	cough with counterflow of qi in the airways, also known as cough with qi reflux	気道内の気の逆流に伴う咳。cough with qi refluxとしても知られる。
腸鳴		2.2.22	borborigmus	a rumbling sound made by the movement of gas in the intestines	腸内のガスの移動によるゴロゴロと鳴る音。
振水音		2.2.23	splashing sound	sounds heard on succussion over a distended stomach	膨張した胃の上で連続して聞こえる音。
矢氣		2.2.24	fecal qi	gas expelled from the anus, the same as flatus	肛門から噴出するガスのこと。放屁（flatus）と同義。
鼻鼾		2.2.25	snoring	rough and noisy breathing while sleeping	睡眠中に生じる荒い吸気性雑音。
口氣		2.2.26	mouth odor	smell emitted from the mouth	口腔から発生する臭い。
口臭		2.2.27	fetid mouth odor	bad smell from the mouth, the same as halitosis	口腔から発生する悪臭。halitosisと同義。
問診		**2.3.0**	**Inquiry**		
問診		2.3.1	inquiry	one of the four diagnostic examinations, a way of gaining information for diagnosis by asking the patient about the complaint and the history of the illness	四診の一つで、愁訴および病歴について患者に質問することにより診断のための情報を得る方法。
問寒熱		2.3.2	inquiry about cold and heat	asking the patient about sensations of heat and cold	患者に熱寒の感覚を質問すること。
發熱	發熱;身熱	2.3.3	fever	elevation of the body temperature above the normal or subjective feeling of feverishness	正常を上回る体温の上昇または主観的に熱っぽいと感じること。
身熱	發熱;身熱	2.3.3	fever	elevation of the body temperature above the normal or subjective feeling of feverishness	正常体温を上回る体温の上昇または主観的に熱っぽいと感じること。
惡寒		2.3.4	aversion to cold	sensation of cold which cannot be relieved by warmth, also known as chills	寒いと感じ、暖かくしても治まらないことで、寒気（chills）としても知られる。
畏寒		2.3.5	fear of cold	sensation of cold which can be relieved by warmth, the same as intolerance of cold	寒いと感じるが、暖かくすれば治まることで、寒冷不耐（intolerance of cold）と同義。
惡風		2.3.6	aversion to wind	strong dislike of wind, also known as wind intolerance	風に対する強い嫌悪で、wind intoleranceとしても知られる。
惡熱		2.3.7	aversion to heat	strong dislike of heat, also known as heat intolerance	熱に対する強い嫌悪で、heat intoleranceとしても知られる。
寒戰		2.3.8	shivering	violent chills with trembling	ふるえを伴う激しい寒気。
惡寒發熱	惡寒發熱; 發熱惡寒	2.3.9	aversion to cold with fever	simultaneous appearance of aversion to cold together with fever	悪寒と同時に発熱が出現する。
發熱惡寒	惡寒發熱; 發熱惡寒	2.3.9	aversion to cold with fever	simultaneous appearance of aversion to cold together with fever	悪寒と同時に発熱が出現する。
但熱不寒		2.3.10	fever without chills	elevation of the body temperature with no feeling of cold	体温が上昇するが寒気は感じない。
但寒不熱		2.3.11	chills without fever	feeling of cold with no fever	寒気を感じるが発熱はない。
壯熱		2.3.12	high fever	persistent high fever with aversion to heat but no chills	壮熱が続き悪熱を伴うが悪寒はない。
潮熱		2.3.13	tidal fever	fever with periodic rise and fall of body temperature at fixed hours of the day like the morning and evening tides	潮の干満のように、定刻になると周期的に体温が上下する発熱。
微熱		2.3.14	mild fever	low-grade fever, mostly due to interior heat	軽度の発熱で、主に裏熱による。
日晡潮熱		2.3.15	late afternoon tidal fever	fever more marked at 3-5 p.m. daily, as if the tide comes regularly	定期的に潮が満ちるように午後3〜5時になると顕著に発熱する。
午後潮熱		2.3.16	afternoon tidal fever	fever more marked in the afternoon	午後になると顕著に発熱する。
身熱不揚		2.3.17	unsurfaced fever	a persistent fever in which heat is not easily felt on the body surface and can be felt only by prolonged palpation, a sign of dampness-heat	持続する発熱で、最初は体表面の熱を感じにくいが、長く触っていると熱を感じるようになる。湿熱の徴候。
身熱夜甚		2.3.18	fever worsening at night	fever more marked at night than at daytime	日中よりも夜間に顕著に発熱する。
骨蒸		2.3.19	steaming bone	a subjective feeling of fever deep in the body, which appears to emanate from the bone or marrow	骨または髄から発しているように思われる体内深部の主観的な感覚。
骨蒸熱		2.3.20	bone-steaming fever	fever which is felt to emanate from the bone or marrow	骨または髄から発していると感じる発熱。
骨蒸潮熱		2.3.21	bone-steaming tidal fever	tidal fever in which the heat is felt to emanate from the bones	熱が骨から発していると感じるような潮熱。
五心煩熱		2.3.22	vexing heat in the chest, palms and soles	feeling of heat in the palms of hands, soles of feet and in the chest, accompanied by uneasiness or restlessness, also called five center heat	不安または落ち着きのなさを伴う両側の手掌、足底および胸部の熱感。five center heatとも呼ばれる。
煩熱		2.3.23	heat vexation	fever accompanied by uneasiness or restlessness, also known as irritable heat sensation	不安または落ち着きのなさを伴う発熱で、いらいらするような熱感（irritable heat sensation）としても知られる。

検索表示（原表示の複数語を分離）	IST原表示	Code	Term	Definition/Description	定義/記述
手足煩熱		2.3.24	vexing heat in the extremities	uncomfortable heat sensation in the extremities	四肢の不快な熱感。
手足心熱		2.3.25	heat in the palms and soles	subjective feverish feeling in the palms and soles	手掌および足底の主観的な熱っぽい感じ。
手足厥冷	手足厥冷; 厥冷; 厥逆	2.3.26	reversal cold of the extremities	pronounced cold in the extremities up to the knees and elbows or beyond, also the same as cold extremities	四肢の膝および肘まで、またはそれらを越えて広がる顕著な冷えのこと。四肢の冷え（cold extremities）と同義。
厥冷	手足厥冷; 厥冷; 厥逆	2.3.26	reversal cold of the extremities	pronounced cold in the extremities up to the knees and elbows or beyond, also the same as cold extremities	四肢の膝および肘まで、またはそれらを越えて広がる顕著な冷えのこと。四肢の冷え（cold extremities）と同義。
厥逆	手足厥冷; 厥冷; 厥逆	2.3.26	reversal cold of the extremities	pronounced cold in the extremities up to the knees and elbows or beyond, also the same as cold extremities	四肢の膝および肘まで、またはそれらを越えて広がる顕著な冷えのこと。四肢の冷え（cold extremities）と同義。
厥		2.3.27	reversal of qi	reverse flow of qi characterized by (1) sudden loss of consciousness, usually of short duration or (2) cold extremities up to the elbows and knees or beyond; also known as syncope or severe coldness	（1）突然の意識消失（通常は短時間）、または（2）四肢の膝および肘まで、またはそれらを越えて広がる冷えを特徴とする気の逆流。失神（syncope）または重度の冷え（severe coldness）とも言う。
寒熱往來	寒熱往來; 往來寒熱; 寒熱交作	2.3.28	alternating chills and fever	chills without fever and fever without chills occurring in alternating succession	但寒不熱と但熱不寒が交互に連続して出現する。
往來寒熱	寒熱往來; 往來寒熱; 寒熱交作	2.3.28	alternating chills and fever	chills without fever and fever without chills occurring in alternating succession	但寒不熱と但熱不寒が交互に連続して出現する。
寒熱交作	寒熱往來; 往來寒熱; 寒熱交作	2.3.28	alternating chills and fever	chills without fever and fever without chills occurring in alternating succession	但寒不熱と但熱不寒が交互に連続して出現する。
外寒內熱		2.3.29	external cold and interior heat	a complex condition characterized by the simultaneous presence of cold in the exterior portion of the body and heat in the interior	体外の寒と体内の熱が同時に存在する複合的な状態。
內寒外熱		2.3.30	internal cold and external heat	a complex condition characterized by the simultaneous presence of heat in the exterior portion of the body and cold in the interior	体外の熱と体内の寒が同時に存在する複合的な状態。
問汗		2.3.31	inquiry about sweating	asking the patient about his/her condition of sweating	発汗の状態について患者に質問すること。
多汗	多汗; 大汗	2.3.32	profuse sweating	excessive sweating not related to a hot environment, physical exertion or taking diaphoretics	暑い環境、激しい身体活動または発汗薬の服用に関連しない過剰な発汗。
大汗	多汗; 大汗	2.3.32	profuse sweating	excessive sweating not related to a hot environment, physical exertion or taking diaphoretics	暑い環境、激しい身体活動または発汗薬の服用に関連しない過剰な発汗。
自汗		2.3.33	spontaneous sweating	excessive sweating during the daytime with no apparent cause such as physical exertion, hot weather, thick clothing or medication	日中の過剰な発汗。激しい身体活動、暑い気候、厚着や服薬など、明らかな原因がないのに生じる。
盜汗		2.3.34	night sweating	sweating during sleep that ceases on awakening	覚醒中には生じず睡眠中にのみ生じる発汗。
冷汗		2.3.35	cold sweating	profuse sweating accompanied by cold body and limbs	大量の発汗に伴い身体および四肢に冷えが生じる。
絕汗		2.3.36	expiry sweating	incessant profuse sweating of a patient in moribund state	瀕死状態の患者でみられる絶え間ない大量の発汗。
戰汗		2.3.37	shiver sweating	sweating following shivering	寒戦後の発汗。
頭汗		2.3.38	sweating from the head	excessive sweating on the head, face or neck	頭部、顔面、頚部の過剰な発汗。
手足心汗		2.3.39	sweating from the palms and soles	excessive local sweating from the palms and soles	手掌および足底からの過剰な局所の発汗。
手足汗		2.3.40	sweating from the hands and feet	excessive local sweating from the hands and feet	手足からの過剰な局所の発汗。
心汗		2.3.41	sweating from the heart region	excessive sweating in the precordial region	前胸（心窩）部の過剰な発汗。
腋汗		2.3.42	sweating from the armpits	excessive local sweating from the armpit	腋窩部からの過剰な局所の発汗。
陰汗		2.3.43	genital sweating	localized sweating in the genital region	陰部の局所的な発汗。
無汗		2.3.44	absence of sweating	abnormal deficiency or absence of sweating, the same as anhidrosis	発汗の異常な欠乏または発汗が生じないこと。無汗症（anhidrosis）と同義。
漏汗		2.3.45	leaking sweat	incessant sweating	絶え間ない発汗。
半身汗出		2.3.46	half-body sweating	sweating only on the upper or lower, right or left half of the body	上半身または下半身、右半身または左半身のみに発汗がみられること。
半身無汗		2.3.47	half-body absence of sweating	no sweating on the upper or lower, right or left half of the body	上半身または下半身、右半身または左半身のみに発汗がみられないこと。
油汗		2.3.48	oily sweat	sticky sweat like oil	油のように粘り気のある汗。
大汗淋漓		2.3.49	great dripping sweat	abnormal profuse sweat with continuous dripping	絶え間なくしたたる異常に大量の汗。
神疲		2.3.50	lassitude of spirit	lack of mental vigor	精神の活力がないこと。
乏力		2.3.51	lack of strength	lack of physical strength	体力がないこと。
嗜睡; 嗜臥		2.3.52	somnolence	excessive sleepiness night and day	夜間、日中を問わない過剰な眠気。

検索表示（原表示の複数語を分離）	IST原表示	Code	Term	Definition/Description	定義/記述
失眠	失眠; 不寐; 不得眠	2.3.53	inability to sleep	inability to sleep or abnormal wakefulness, the same as insomnia	睡眠不能または異常な覚醒状態。不得臥と同義。
不寐	失眠; 不寐; 不得眠	2.3.53	inability to sleep	inability to sleep or abnormal wakefulness, the same as insomnia	睡眠不能または異常な覚醒状態。不得臥と同義。
不得眠	失眠; 不寐; 不得眠	2.3.53	inability to sleep	inability to sleep or abnormal wakefulness, the same as insomnia	睡眠不能または異常な覚醒状態。不得臥と同義。
多夢		2.3.54	profuse dreaming	a condition in which sleep is frequently disturbed by dreams	夢のために睡眠が頻繁に妨げられる状態。
夢遊		2.3.55	sleep walking	rising out of bed and walking about or performing other behavior during an apparent state of sleep	明らかな睡眠状態にある間に、寝床を離れ、歩き回ったり他の行動をとったりすること。
身痛		2.3.56	generalized pain	pain involving the whole body, the same as general body ache	全身の疼痛。全身痛（general body ache）と同義。
頭痛		2.3.57	headache	pain in the head	頭部の疼痛。
項强		2.3.58	stiffness of the neck	muscular contraction and rigidity of the neck and the upper back with impaired movement	頚部および上背部に筋収縮と硬直が生じ、動かしづらい状態。
頭項强痛		2.3.59	headache and painful stiff nape	headache accompanied by rigidity and pain of the nape	項部の硬直と疼痛を伴う頭痛。
頭重		2.3.60	heavy-headedness	feeling of heaviness in the head	頭が重苦しい感じ。
眉稜骨痛		2.3.61	supraorbital pain	pain felt in the eyebrow bone	眉稜骨（眼窩上）に感じる疼痛。
耳痛		2.3.62	ear pain	pain in the ear	耳の疼痛。
胸痛		2.3.63	chest pain	pain in the middle or lateral part of the chest	胸部中央部または外側部の疼痛。
乳房疼痛		2.3.64	breast pain	pain in the breast, usually accompanied by feeling of distension	乳房の疼痛で、通常は膨張感を伴う。
脇痛		2.3.65	hypochondriac pain	pain in the area between the armpit and the lowest rib, unilateral or bilateral	片側または両側の腋窩部から最下肋骨部にかけての疼痛。
脘痛	脘痛; 胃痛	2.3.66	stomach pain	the same as epigastric pain	心窩部痛（epigastric pain）と同義。
胃痛	脘痛; 胃痛	2.3.66	stomach pain	the same as epigastric pain	心窩部痛（epigastric pain）と同義。
腹痛		2.3.67	abdominal pain	pain in the region between the hypochondrium and pubic hairline	下肋部から陰毛の生え際にかけての疼痛。
疝		2.3.68	lower abdominal colic	severe colicky pain in the lower abdomen usually accompanied by constipation and ischuria	下腹部の重度の疝痛で、通常は便秘および尿閉に付随する。
背痛		2.3.69	back pain	pain in the upper part of the back	上背部の疼痛。
腰痛		2.3.70	lumbago	pain in the lumbar region	腰部の疼痛。
陰器痛		2.3.71	genital pain	pain in the genitals	生殖器の疼痛。
足跟痛		2.3.72	heel pain	pain in either or both heels, worsened by standing and walking	片側または両側の踵の疼痛で、起立や歩行により悪化する。
脹痛		2.3.73	distending pain	pain accompanied by a distending sensation	膨満感を伴う疼痛。
刺痛		2.3.74	stabbing pain	a sharp pain as if caused by a stab	刺されるような鋭い疼痛。
冷痛		2.3.75	cold pain	pain accompanied by a cold sensation and relieved by warmth	冷感を伴い、温めると消失する疼痛。
灼痛		2.3.76	scorching pain	pain accompanied by a burning sensation, also called burning pain	灼熱感を伴う疼痛で、灼熱痛（burning pain）とも言う。
絞痛		2.3.77	gripping pain	an acute pain in the chest or abdomen, often caused by spasm, obstruction or twisting, also called spasm and pain	胸部または腹部の急性の疼痛で、しばしば拘攣、閉塞またはねじれにより生じる。spasm and painとも呼ばれる。
重痛		2.3.78	heavy pain	a pain accompanied by heaviness sensation	重い感じを伴う疼痛。
掣痛		2.3.79	pulling pain	pain in one part involving other parts	ある部分から他の部分に波及する疼痛。
隱痛		2.3.80	dull pain	a continuous pain not stabbing in nature	持続する疼痛で、刺すような痛みではない。
空痛		2.3.81	empty pain	a pain accompanied by feeling of emptiness	空虚感を伴う疼痛。
竄痛		2.3.82	scurrying pain	pain that repeatedly changes location	部位を変えて繰り返される疼痛。
悶痛		2.3.83	oppressive pain	a pain accompanied by feeling of oppression	重圧感を伴う疼痛。
酸痛		2.3.84	aching pain	a continuous dull pain as the sensation produced by prolonged physical exertion	長時間激しい身体活動を行った後に感じるような持続的な鈍い疼痛。
遊走痛		2.3.85	wandering pain	pain in the joints of the extremities with repeated change of location	場所を変えて繰り返される四肢の関節痛。
痛無定處		2.3.86	pain of unfixed location	a pain whose location is not fixed	部位が固定されない疼痛。
固定痛		2.3.87	fixed pain	a pain that is fixed in location	部位が固定された疼痛。
劇痛		2.3.88	severe pain	intense intolerable pain	耐えがたい激しい疼痛。
陣發痛		2.3.89	paroxysmal pain	pain recurring in paroxysms	発作において繰り返し訪れる疼痛。
持續痛		2.3.90	persistent pain	pain continuing without interruption	途切れることなく持続する疼痛。
拘攣	拘攣; 攣急	2.3.91	spasm	stiffness and tension in the limbs inhibiting normal bending and stretching	正常な屈伸ができない四肢の強直と緊張。
攣急	拘攣; 攣急	2.3.91	spasm	stiffness and tension in the limbs inhibiting normal bending and stretching	正常な屈伸ができない四肢の強直と緊張。
拘急		2.3.92	contracture	abnormal tension of sinews that manifests in inhibited bending and stretching of the limb	四肢の屈伸ができない異常な筋肉の緊張。
項背拘急		2.3.93	contracture of the nape and neck	muscular spasm and rigidity of the nape and back	項部および背部の筋肉の拘攣および硬直。
四肢拘急		2.3.94	contracture of the limbs	muscular contracture of the limbs with difficulty in bending and stretching, the same as acrocontracture	四肢の筋拘縮のために屈伸が困難な状態。四肢拘縮（acrocontracture）と同義。
頭重脚輕		2.3.95	heavy head and light feet	the subjective sensation whereby the head is heavier than feet, accompanied by unsteady gait	主観的に頭部が足部よりも重く感じ、よろよろ歩く。

検索表示（原表示の複数語を分離）	IST原表示	Code	Term	Definition/Description	定義/記述
身重		2.3.96	heavy body	the subjective heaviness sensation of the body with difficult movement	主観的に身体が重い感じがし、動かしづらい状態。
身癢		2.3.97	generalized itching	itching all over the body	全身のかゆみ。
肌膚麻木		2.3.98	numbness of the skin	local absence of normal sensation in the skin	皮膚の正常な局所感覚の欠如。
不仁		2.3.99	insensitivity	no perception to touch, also known as numbness	触られても感じないこと。麻木とも呼ばれる。
筋惕肉瞤		2.3.100	muscular twitching and cramp	short spastic muscular contractions	短い痙攣性筋収縮。
失血	失血; 奪血	2.3.101	loss of blood	a general term for various kinds of profuse bleeding, the same as hemorrhage	さまざまな大量出血に対する一般用語で、出血（hemorrhage）と同義。
奪血	失血; 奪血	2.3.101	loss of blood	a general term for various kinds of profuse bleeding, the same as hemorrhage	さまざまな大量出血に対する一般用語で、出血（hemorrhage）と同義。
喀血	喀血; 咳血	2.3.102	hemoptysis	the expectoration of blood or blood-stained sputum	血または血の混じった痰の喀出。
咳血	喀血; 咳血	2.3.102	hemoptysis	the expectoration of blood or blood-stained sputum	血または血の混じった痰の喀出。
衄血		2.3.103	epistaxis	non-traumatic external bleeding, including bleeding from the ear, nose, gum, and tongue, and subcutaneous hemorrhage	耳、鼻、歯肉、舌、皮下の出血など、非外傷性の体外の出血。
唾血		2.3.104	spitting of blood	expulsion of blood with saliva	唾液とともに血を吐出すること。
蓄血		2.3.105	blood amassment	static blood accumulating in the interior	体内に蓄積した瘀血。
目癢		2.3.106	itchy eyes	itching of the eye	目の痒み。
目痛		2.3.107	eye pain	pain of the eye	目の疼痛。
目眩		2.3.108	dizzy vision	blurred vision accompanied by vertigo	めまいを伴う目昏（かすみ眼）。
目昏	目昏; 視物模糊; 視瞻昏渺	2.3.109	blurred vision	clouded and unclear vision	ぼんやりした不鮮明な視力。
視物模糊	目昏; 視物模糊; 視瞻昏渺	2.3.109	blurred vision	clouded and unclear vision	ぼんやりした不鮮明な視力。
視瞻昏渺	目昏; 視物模糊; 視瞻昏渺	2.3.109	blurred vision	clouded and unclear vision	ぼんやりした不鮮明な視力。
視岐		2.3.110	double vision	seeing one object as two	1つの物が二重に見えること。
耳鳴		2.3.111	tinnitus	ringing in the ear	耳内で音が鳴り響くこと。
耳聾		2.3.112	deafness	loss, complete or partial, of the sense of hearing	完全または部分的な聴覚の喪失。
鼻塞	鼻塞; 鼻窒	2.3.113	nasal congestion	blockage of the nasal passage	鼻腔の遮断。
鼻窒	鼻塞; 鼻窒	2.3.113	nasal congestion	blockage of the nasal passage	鼻腔の遮断。
鼻不聞香臭		2.3.114	loss of smell	partial or total inability to smell	部分的または完全な嗅覚消失。
口乾		2.3.115	dry mouth	lack of fluid in the mouth	口腔内の津の欠如。
口渇		2.3.116	thirst	feeling of dryness of the mouth with a desire to drink	口が乾いた感じを伴う飲水渇望。
納呆		2.3.117	torpid intake	loss of appetite with no desire for food with decreased intake, the same as poor appetite	食欲が喪失し、食物に対する渇望がなく、摂取量が減少すること。食欲不振（poor appetite）と同義。
消穀善饑		2.3.118	swift digestion with rapid hungering	excessive appetite with increased food intake and recurrence of hunger sensation shortly after eating	過剰な食欲のために摂取量が増加し、食べた直後に再び空腹感に襲われること。
口味		2.3.119	taste in the mouth	the taste experienced when there is nothing in the mouth	口腔内に何も入っていないのに味覚を感じること。
口淡		2.3.120	bland taste in the mouth	diminished sensitivity of taste, with no pleasant flavor left by eating	味覚が低下し、食べても良い風味が残らないこと。
口苦		2.3.121	bitter taste in the mouth	a subjective bitter sensation in the mouth	主観的に口の中が苦く感じること。
口甜		2.3.122	sweet taste in the mouth	a subjective sweet sensation in the mouth	主観的に口の中が甘く感じること。
口酸		2.3.123	sour taste in the mouth	a subjective sour sensation in the mouth	主観的に口の中が酸っぱく感じること。
口澁		2.3.124	astringent taste in the mouth	a subjective feeling of astringency in the mouth	主観的に口の中が渋く感じること。
口鹹		2.3.125	salty taste in the mouth	a subjective salty sensation in the mouth	主観的に口の中が塩辛く感じること。
口麻		2.3.126	numbness in the mouth	numbness of the tongue with diminished taste sensitivity	舌がしびれ、味覚が消失すること。
口粘膩		2.3.127	sticky slimy sensation in the mouth	an unpleasant subjective sensation of stickiness in the mouth	口の中がねばねばして不快な主観的な感覚。
咽喉不利		2.3.128	discomfort in the throat	a mild sensation of blockage in the throat that hampers swallowing	喉が軽度に遮断されている感覚で、嚥下が妨げられる。
噎		2.3.129	choke	difficulty in swallowing	嚥下困難。
惡心		2.3.130	nausea	an unpleasant sensation with an urge to vomit	嘔吐を促す不快感。

検索表示（原表示の複数語を分離）	IST原表示	Code	Term	Definition/Description	定義/記述
呑食梗塞		2.3.131	blockage when swallowing	difficulty or even inability to swallow	嚥下困難または嚥下不能。
嘔吐		2.3.132	vomiting	forcible expulsion of the stomach contents through the mouth	胃内容物を強制的に口から吐出すること。
朝食暮吐		2.3.133	vomiting in the evening of food eaten in the morning	a characteristic symptom of stomach reflux, usually occurring together with vomiting in the morning of food eaten in the evening	特徴的な胃逆流症状で、通常は朝食べた物を夕方に吐くこと。
暮食朝吐		2.3.134	vomiting in the morning of food eaten in the evening	a characteristic symptom of stomach reflux, usually occurring together with vomiting in the evening of food eaten in the morning	特徴的な胃逆流症状で、通常は夕方食べた物を朝に吐くこと。
乾嘔		2.3.135	dry retching	a noisy involuntary effort to vomit, but without bringing anything up from the stomach	音を立てて不随意に嘔吐しようとするが、胃からの吐出物はない。
宿食		2.3.136	retained food	food and drink accumulating in the stomach and intestines, undigested overnight	飲食物が胃腸に停滞し、一晩消化されないままの状態。
嗜偏食		2.3.137	dietary predilection	a liking for particular flavors or specific foods	特定の風味や食物への嗜好。
噦		2.3.138	retching	making the sounds and movements of vomiting bringing up little or nothing from the stomach	音を立てて嘔吐行動をとるが、胃からの吐出物はほとんど、またはまったくない。
噯氣		2.3.139	belching	the casting up of gas from the stomach, accompanied by a prolonged sound	長く延びた音を立ててガスが胃から排出される。
噫氣		2.3.139	belching	the casting up of gas from the stomach, accompanied by a prolonged sound	長く延びた音を立ててガスが胃から排出される。
呑酸		2.3.140	acid regurgitation	swallowing of acid contents regurgitated from the stomach to the throat	胃から喉に逆流した酸味の内容物を飲み込むこと。
心悸		2.3.141	palpitations	a subjective sensation of rapid and forceful beating of the heart	主観的に心臓の拍動が速く力強くなったと感じること。
心下悸		2.3.142	palpitations below the heart	pulsations felt in the epigastric region below the xiphoid process	剣状突起より下の心窩部で感じる拍動。
心慌		2.3.143	flusteredness	a nervous agitated state often with palpitations	神経が興奮した状態で、しばしば心悸を伴う。
心煩		2.3.144	vexation	a feeling of restlessness, heat, and oppression in the region of the heart	心部の落ち着きがない感じ、熱感、重圧感。
虚煩		2.3.145	vexation of deficiency type	vexation due to deficiency fire harassing the inner body	体内を苦しめる虚火による心煩
心中懊憹		2.3.146	anguish in the heart	heat vexation and oppression in the region of the heart and chest	心臓および胸部の煩熱および重圧感。
心下急		2.3.147	distress below the heart	a feeling of distress associated with slight pain and fullness in the epigastric region, the same as epigastric distress	軽度の疼痛と充満感を伴う、心窩部が苦しい感じ。心窩部不快感（epigastric distress）と同義。
心下堅	心下堅;心下鞕	2.3.148	rigidity below the heart	abnormal stiffness in the epigastric region, the same as epigastric rigidity	心窩部の異常な強直。心窩部硬直（epigastric rigidity）と同義。
心下鞕	心下堅;心下鞕	2.3.148	rigidity below the heart	abnormal stiffness in the epigastric region, the same as epigastric rigidity	心窩部の異常な強直。心窩部硬直（epigastric rigidity）と同義。
心下支結		2.3.149	tightness below the heart	a feeling of obstruction associated with vexation and distension in the epigastric region, the same as epigastric tightness	心煩および膨満感を伴う心窩部の閉塞感。心窩部圧迫感（epigastric tightness）と同義。
心下痞堅	心下痞堅;心下痞鞕	2.3.150	stuffiness and rigidity below the heart	a feeling of stuffiness associated with local rigidity in the epigastric region, the same as epigastric stuffiness and rigidity	局所硬直を伴う心窩部が詰まった感じ。epigastric stuffiness and rigidityと同義。
心下痞鞕		2.3.150	stuffiness and rigidity below the heart	a feeling of stuffiness associated with local rigidity in the epigastric region, the same as epigastric stuffiness and rigidity	局所硬直を伴う心窩部が詰まった感じ。epigastric stuffiness and rigidityと同義。
心下滿		2.3.151	fullness below the heart	a feeling of fullness in the epigastric region, the same as epigastric fullness	心窩部の充満感。epigastric fullnessと同義。
痞		2.3.152	stuffiness	a localized subjective feeling of fullness and blockage	主観的に局所が充満して遮断された感じ。
痞滿		2.3.153	stuffiness and fullness	unwell feeling of stuffiness and fullness in the chest and upper abdomen	胸部および上腹部の不快な詰まった感じおよび充満感。
鞕滿	鞕滿;硬滿	2.3.154	hardness and fullness	feeling hard to touch with a subjective feeling of fullness	主観的な充満感を伴い触ると硬い感じ。
硬滿	鞕滿;硬滿	2.3.154	hardness and fullness	feeling hard to touch with a subjective feeling of fullness	主観的な充満感を伴い触ると硬い感じ。
胸中痞硬	胸中痞硬;胸下痞硬	2.3.155	stuffiness and rigidity in the chest	feeling of stuffiness and choking in the chest	胸部が詰まり息苦しい感じ。
胸下痞硬		2.3.155	stuffiness and rigidity in the chest	feeling of stuffiness and choking in the chest	胸部が詰まり息苦しい感じ。
胸悶		2.3.156	oppression in the chest	feeling of oppression in the chest	胸部の重圧感。
胸脇滿	胸脇滿;胸脇苦滿	2.3.157	fullness in the chest and hypochondrium	a bloated feeling in the chest and hypochondriac region	胸部および下肋部が張って膨らんだ感じ。
胸脇苦滿	胸脇滿;胸脇苦滿	2.3.157	fullness in the chest and hypochondrium	a bloated feeling in the chest and hypochondriac region	胸部および下肋部が張って膨らんだ感じ。
痃癖		2.3.158	paraumbilical and hypochondriac aggregation	elongated masses located at the side of the umbilicus or in the hypochondriac region that occur intermittently with sharp pain	臍側または下肋部に局在する楕円形の腫瘤で、間欠的に鋭い痛みが生じる。
腹滿		2.3.159	abdominal fullness	a subjective feeling of fullness in the abdomen without pronounced visible or palpable expansion	見たり触ったりしても拡張は感じないが腹部が膨満しているという主観的な感じ。
小腹急結		2.3.160	lower abdominal cramp	subjective sensation of distention and fullness of the lower abdomen, usually with difficult urination	下腹部の主観的な脹満感で、通常、排尿困難となる。

検索表示（原表示の複数語を分離）	IST原表示	Code	Term	Definition/Description	定義/記述
小腹拘急	小腹拘急;小腹弦急	2.3.161	lower abdominal contracture	cramping sensation of the lower abdomen	下腹部がひきつる感じ。
小腹弦急	小腹拘急;小腹弦急	2.3.161	lower abdominal contracture	cramping sensation of the lower abdomen	下腹部がひきつる感じ。
小腹滿	小腹滿;小腹硬滿	2.3.162	lower abdominal fullness	a subjective feeling of fullness in the lower abdomen	主観的な下腹部の充満感。
小腹硬滿	小腹滿;小腹硬滿	2.3.162	lower abdominal fullness	a subjective feeling of fullness in the lower abdomen	主観的な下腹部の充満感。
小腹不仁		2.3.163	lower abdominal numbness	loss of sensation in the lower abdominal region	下腹部の感覚消失。
臍上悸		2.3.164	palpitations above the umbilicus	rapid pulsations felt in the region above the umbilicus, the same as supra-umbilical aortic pulsation	臍上部で感じる速い拍動。supra-umbilical aortic pulsationと同義。
臍傍悸		2.3.165	palpitations beside the umbilicus	rapid pulsations felt in the para-umbilical region, the same as para-umbilical aortic pulsation	臍傍部で感じる速い拍動。para-umbilical aortic pulsationと同義。
臍下悸	臍下悸;臍下悸動	2.3.166	palpitations below the umbilicus	rapid pulsations felt in the region below the umbilicus, the same as sub-umbilical aortic pulsation	臍下部で感じる速い拍動。sub-umbilical aortic pulsationと同義。
臍下悸動	臍下悸;臍下悸動	2.3.166	palpitations below the umbilicus	rapid pulsations felt in the region below the umbilicus, the same as sub-umbilical aortic pulsation	臍下部で感じる速い拍動。sub-umbilical aortic pulsationと同義。
臍下拘急		2.3.167	contracture below the umbilicus	muscular contracture in the region below the umbilicus	臍下部の筋拘縮。
臍下不仁		2.3.168	numbness below the umbilicus	loss of sensation in the region below the umbilicus	臍下部の感覚消失。
泄瀉		2.3.169	diarrhea	abnormally frequent intestinal evacuations with soft or fluid stool	軟便または液状便が異常なほど頻繁に排出されること。
大便自利		2.3.170	spontaneous diarrhea	diarrhea not attributable to purgation	瀉下によらない泄瀉（下痢）。
水瀉	水瀉;注泄	2.3.171	watery diarrhea	serious diarrhea with a forceful discharge of watery stool, also the same as outpour diarrhea	水様便の強制的な排出を伴う重篤な泄瀉。outpour diarrheaと同義。
注泄	水瀉;注泄	2.3.171	watery diarrhea	serious diarrhea with a forceful discharge of watery stool, also the same as outpour diarrhea	水様便の強制的な排出を伴う重篤な泄瀉。outpour diarrheaと同義。
瀉下不爽		2.3.172	ungratifying diarrhea	diarrhea that leaves the patient with a feeling that the bowels have not been satisfactorily emptied	残便感の残る泄瀉。
完穀不化		2.3.173	undigested food in stool	a condition in which the stool contains undigested food	便に未消化の食物が含まれている状態。
下利清穀	下利清穀;完穀下利	2.3.174	clear-food diarrhea	frequent discharge of fluid stools containing undigested food, the same as undigested food diarrhea	未消化の食物を含む液状便が頻繁に排出されること。undigested food diarrheaと同義。
完穀下利	下利清穀;完穀下利	2.3.174	clear-food diarrhea	frequent discharge of fluid stools containing undigested food, the same as undigested food diarrhea	未消化の食物を含む液状便が頻繁に腸から排出されること。undigested food diarrheaと同義。
裏急		2.3.175	abdominal urgency	(1) urge to defecate; (2) contraction of male genital organ	（1）便意切迫感、（2）男性生殖器の収縮。
裏急後重		2.3.176	tenesmus	ineffectual and painful straining at stool	無駄で疼痛のあるいきみ。
五更泄		2.3.177	fifth-watch diarrhea	diarrhea occurring daily at dawn, usually due to kidney yang deficiency, the same as early morning diarrhea	毎日早朝（夜明け）に生じる泄瀉で、通常、腎陽虚による。晨泄（early morning diarrhea）と同義。
滑泄		2.3.178	efflux diarrhea	diarrhea with fecal incontinence	大便滑脱を伴う泄瀉。
洞泄		2.3.179	throughflux diarrhea	diarrhea after eating, with undigested food in the stool	食後の泄瀉で、便中に未消化の食物を含む。
暴瀉		2.3.180	fulminant diarrhea	a pathological change characterized by sudden onset of diarrhea with profuse discharge	大量の分泌液を伴う泄瀉が突然発生する病的な変化。
溏泄		2.3.181	sloppy diarrhea	diarrhea with soft, unformed stool	形状のない軟便の泄瀉。
寒泄		2.3.182	cold diarrhea	diarrhea caused by attack of cold on the gastrointestinal tract	胃腸管への寒邪の攻撃により生じる泄瀉。
濡泄		2.3.183	soggy diarrhea	diarrhea due to damage to the spleen by exuberant dampness	旺盛な湿によって脾が損傷することにより生じる泄瀉。
飧泄		2.3.184	swill diarrhea	diarrhea marked by watery stool containing undigested food	未消化の食物を含む水様便を特徴とする泄瀉。
便溏		2.3.185	sloppy stool	discharge of soft, unformed stools, the same as loose stool	形状のない軟便の排出。軟便（loose stool）と同義。
溏結不調		2.3.186	stool sometimes sloppy and sometimes bound	stool that varies greatly in consistency	さまざまな硬さの便。
便膿血		2.3.187	stool containing pus and blood	passage of blood, pus and mucus together with stool, a symptom usually indicating dysentery	便とともに血、膿、粘液が排出されることで、通常、赤痢を示す。
大便硬結		2.3.188	hard bound stool	hardened feces difficult to evacuate	排出困難な硬い便。
大便乾燥		2.3.189	dry stool	feces lacking moisture content, difficult to evacuate	水分をほとんど含まない排出困難な便。
燥屎		2.3.189	dry stool	feces lacking moisture content, difficult to evacuate	水分をほとんど含まない排出困難な便。
大便滑脫		2.3.190	fecal incontinence	inability to control defecation, with involuntary passage of feces	排便を制御できず、便が不随意に排出される状態。
小便自利		2.3.191	spontaneous urination	spontaneous discharge of urine	自発的な排尿。
小便難	小便難;癃閉	2.3.192	difficult urination	difficulty in urination or anuria	排尿困難または無尿。
癃閉	小便難;癃閉	2.3.192	difficult urination	difficulty in urination or anuria	排尿困難または無尿。

検索表示（原表示の複数語を分離）	IST原表示	Code	Term	Definition/Description	定義/記述
小便不利		2.3.193	inhibited urination	difficult discharge of urine, usually scanty in amount, the same as dysuria	排尿が困難で、通常はごく少量しか排尿できない。排尿困難（dysuria）と同義。
小便失禁		2.3.194	urinary incontinence	failure of voluntary control of urination	排尿を随意に制御できない状態。
小便頻數		2.3.195	frequent urination	increased frequency of urination	排尿回数の増加。
小便澁痛		2.3.196	difficult painful urination	uneasy and painful voiding of urine	排尿が容易でなく痛みを伴う状態。
小便渾濁		2.3.197	turbid urine	murky urine like rice water or grease	米のとぎ汁または油脂のように濁った尿。
尿濁		2.3.197	turbid urine	murky urine like rice water or grease	米のとぎ汁または油脂のように濁った尿。
小便淋漓		2.3.198	dribbling urination	dribbling discharge of urine with inability to achieve a full stream	尿線を描けず、ぽたぽたと落ちる尿。
小便黃赤		2.3.199	reddish yellow urine	dark yellow or even reddish urine	暗黄色または赤色の尿。
遺尿		2.3.200	enuresis	involuntary discharge of urine during sleep	睡眠中の不随意な排尿。
陽痿		2.3.201	impotence	lack of copulative power in the male	男性の性交能の欠如。
性欲淡漠		2.3.202	poor libido	lack of desire for sexual gratification, the same as low libido	性的満足を得たいという欲求の減退。性欲減退（low libido）と同義。
夢交		2.3.203	dreaming of intercourse	dreaming of performing coitus	性交する夢を見ること。
切診		**2.4.0**	**Palpation**		
切診		2.4.1	palpation	one of the four diagnostic examinations, including touching and pressing the body surface with the hand or fingers	四診の1つで、手や指で体表を触ったり押したりすること。
脈診		2.4.2	pulse diagnosis	examination of the pulse for making diagnosis	診断のための脈の診察。
切脈		2.4.3	take the pulse	examine the pulsation of blood vessels by feeling with the fingertips	指先の感覚で脈拍を調べること。
脈象		2.4.4	pulse condition	the condition of the pulse felt on examination	診察時に触れた脈の状態。
脈象主病		2.4.5	disease correspondences of the pulse	a certain type of pulse indicates a particular disease	特定の種類の脈が特定の疾病を示すこと。
三部九候		2.4.6	three positions and nine indicators	(1) the upper, middle and lower arteries of the head, and the upper and lower limbs for pulse examination; (2) three sections of the radial artery at the wrist designated as inch, bar and cubit, each felt with light, moderate and heavy force to study the superficial, medium and deep pulses, respectively	（1）脈診に用いる頭部、上肢、下肢それぞれにある上、中、下の動脈、（2）手首の橈骨動脈を寸、関、尺の3部に分け、それぞれ軽く押さえて浮脈（表層部）、少し力を入れて押さえて中脈（中間部）、強く押さえて沈脈（深部）を把握すること。
寸口	寸口;<br>氣口	2.4.7	wrist pulse	the pulsation of the radial artery felt at the wrist	手首で触れる橈骨動脈の拍動。
氣口	寸口;<br>氣口	2.4.7	wrist pulse	the pulsation of the radial artery felt at the wrist	手首で触れる橈骨動脈の拍動。
寸口診法		2.4.8	wrist pulse-taking method	method of taking pulse at the radial artery of the wrist	手首で橈骨動脈の脈をとる方法。
寸關尺		2.4.9	cun/inch, guan/bar and chi/cubit	the three sections over the radial artery for feeling the pulse: The bar/guan is just central to the radical styloid of the wrist , where the tip of the physician's middle finger is placed, the inch/cun is next to it on the distal side where the tip of the physician's index finger rests, and the cubit/chi is on the proximal side where the tip of the physician's ring finger is placed	脈を感じとるために橈骨動脈を3部に分けた名称。関は手首の橈骨茎状突起のちょうど中心部で、医師が中指の先を当てる場所、寸はその遠位側で、医師が示指を当てる場所、尺は近位側で医師が薬指を当てる場所である。
反關脈		2.4.10	pulse on the back of the wrist	an anatomical anomaly of the radial artery which makes the pulse beat felt on the back of the wrist	橈骨動脈の解剖学的異常で、このため手首の背側で脈に触れる。
斜飛脈		2.4.11	oblique-running pulse	an anatomical anomaly of the radial artery which makes the pulse beat felt running outwards from the cubit/chi to the back of the hand	橈骨動脈の解剖学的異常で、このため尺部から手背部外側に向かう脈に触れる。
人迎		2.4.12	carotid pulsation	pulsation of the carotid artery, easily felt beside the laryngeal prominence	頸動脈の拍動で、喉頭隆起のそばで容易に触れる。
趺陽脈		2.4.13	anterior tibial pulse	the artery whose pulsation can be easily felt at the instep	足甲部で容易に触れる動脈の拍動。
平脈		2.4.14	normal pulse	the pulse of a normal person	正常な人の脈。
病脈		2.4.15	morbid pulse	a pulse indicating pathological changes	病的な変化を示す脈。
胃, 神, 根		2.4.16	stomach, vitality and root	the three qualities for a normal pulse: A normal pulse is regular, smooth and harmonious, indicating the presence of stomach qi. It is supple and powerful, indicating the presence of vitality. It can be felt on deep palpation, indicating the presence of root	正常な脈象の3つの特徴：正常な脈は規則正しく滑らかで調和がとれており、胃気の存在を示す。柔和で力強いものは神が存在していることを示す。脈を沈取できる（深部で脈に触れられる）ものは根の存在を示す。
指法		2.4.17	finger technique	the finger manipulation used in pulse examination	指で脈診を行う方法。
指目		2.4.18	pulse-taking with finger-tips	a form of pulse examination using only the tips of the fingers in contact with the pulse	指の先端のみで脈に触れる脈診法の一形態。
布指		2.4.19	finger positioning	positioning of fingers in pulse examination	脈診における指の位置。
擧, 按, 尋		2.4.20	lifting, pressing and searching	the three manipulations in pulse taking	脈をとるときの3種類の手の動かし方。
推尋		2.4.21	pushing and searching	pushing and moving the finger to examine the pulse	脈診のために指を押して動かすこと。
單按		2.4.22	pressing with one finger	taking the pulse at each of the three sections individually	1本の指で3部の脈を1部ずつとること。
總按		2.4.23	simultaneous palpation	taking the pulse of the three sections with three fingers simultaneously	3本の指で3部の脈を同時にとること。
脈氣		2.4.24	vessel qi	qi flowing in the vessel	脈を流れる氣。
脈靜		2.4.25	tranquil pulse	pulse that becomes gentle, in the course of an illness, usually indicating improvement of the condition	疾患の過程で温和になる脈。通常は状態の改善を示す。

検索表示（原表示の複数語を分離）	IST原表示	Code	Term	Definition/Description	定義/記述
脈躁		2.4.26	agitated pulse	pulse that becomes rapid and rushing, usually indicating deterioration of the condition	速くて慌ただしくなる脈。通常は状態の悪化を示す。
脈暴出		2.4.27	fulminating pulse	sudden throbbing of a hardly perceptible pulse, usually indicating a critical condition	感じにくかった脈が突然脈打つこと。通常は危篤状態を示す。
脈無胃氣		2.4.28	pulse bereft of stomach qi	a pulse that has lost its usual rhythm, frequency and evenness, indicating lack of stomach qi	脈に通常のリズム、頻度、一定性がなくなった状態で、胃気の欠如を示す。
浮脈		2.4.29	floating pulse	a superficially located pulse which can be felt by light touch and grows faint on hard pressure	浅表部にある脈で、軽く押さえると拍動を感じるが、強く押さえると拍動が弱まる。
沈脈		2.4.30	sunken pulse	a deeply located pulse which can only be felt when pressing hard, also called deep pulse	深部にある脈で、強く押さないと拍動が感じられない。deep pulseとも呼ばれる。
遲脈		2.4.31	slow pulse	a pulse with less than four beats to one cycle of the physician's respiration, the same as bradycardia	医師の1呼吸周期当たり4回未満拍動する脈。徐脈（bradycardia）と同義。
數脈		2.4.32	rapid pulse	a pulse with more than five or six beats to one cycle of the physician's respiration, the same as tachycardia	医師の1呼吸周期当たり5〜6回以上拍動する脈。頻脈（tachycardia）と同義。
洪脈		2.4.33	surging pulse	a pulse beating like dashing waves with forceful rising and gradual decline, also called flooding pulse	勢いのある波のように、来るときは力強く、去るときは力が弱まる脈。flooding pulseとも呼ばれる。
細脈		2.4.34	fine pulse	a pulse as thin as a silk thread, straight and soft, feeble yet always perceptible upon hard pressure, also called thin/thready pulse	絹糸のように細く、まっすぐで柔らかく弱いが、強く押さえると常に拍動がはっきりと感じとれる脈。thin/thready pulseとも呼ばれる。
虛脈		2.4.35	vacuous pulse	a general term for a feeble and void pulse	弱く空虚な脈の一般用語。
實脈		2.4.36	replete pulse	a general term for a pulse felt forceful at all the three sections, cun/inch, guan/bar and chi/cubit, also called forceful pulse	3部（寸関尺）すべてで力強い拍動を感じられる脈の一般用語。力強い脈（forceful pulse）とも呼ばれる。
長脈		2.4.37	long pulse	a pulse with beats of long duration, exceeding cun/inch, guan/bar and chi/cubit sections	脈来が長く、寸、関、尺の部位を超える脈。
短脈		2.4.38	short pulse	a pulse with beats of short duration, only felt at guan/bar section	脈来が短く、関でのみ感じられる脈。
滑脈		2.4.39	slippery pulse	a pulse coming and going smoothly like beads rolling on a plate	ガラス玉が板の上を転がるように脈が滑らかに去来する。
澁脈		2.4.40	rough pulse	a pulse coming and going unsmoothly with small, fine, slow joggling tempo like scraping bamboo with a knife	ナイフで竹を削るときのように、脈の去来が滑らかでなく、細小で、遅く揺れながら拍動する。
弦脈		2.4.41	string-like pulse	a straight, long and taut pulse, like a musical string to the touch	楽器の弦に触れたような、まっすぐで長く、張りつめた脈。
緊脈		2.4.42	tight pulse	a pulse feeling like a tightly stretched cord	ひもをピンと張った感じの脈。
濡脈		2.4.43	soggy pulse	a thin and floating pulse which can be felt on light pressure, but growing faint upon hard pressure	細い浮脈で、軽く押さえると拍動が感じられ、強く押さえると弱くなる。
緩脈		2.4.44	moderate pulse	a pulse with four beats to one cycle of the physician's respiration, even and harmonious in its form	医師の1呼吸周期当たり4回拍動する脈で、均一で調和的。
緩脈		2.4.45	relaxed pulse	a pulse with decreased tension	弛緩した脈。
微脈		2.4.46	faint pulse	a thready and soft pulse, scarcely perceptible	糸のように細く柔らかい脈で、はっきり感じとれない。
弱脈		2.4.47	weak pulse	a pulse that is deep, soft, thin and forceless	柔らかく細い沈脈で、力強さがない。
散脈		2.4.48	dissipated pulse	a pulse that feels diffusing and feeble upon a light touch and faint upon hard pressure	軽く押さえると散漫で細く感じられ、強く押さえるとはっきりしなくなる脈。
芤脈		2.4.49	hollow pulse	a floating, large, soft, and hollow pulse	浮遊性で大きく柔らかく、空洞を感じる脈。
革脈		2.4.50	drumskin pulse	a pulse felt hard and hollow as if touching the surface of a drum	太鼓の表面に触れたように、硬く空洞を感じる脈。
牢脈		2.4.51	firm pulse	a broad, forceful and taut pulse, deeply seated and felt only by hard pressure	幅広く力強い張りつめた沈脈で、強く押さえないと感じられない。
伏脈		2.4.52	hidden pulse	a pulse which can only be felt upon pressing to the bone, located deeper than sunken pulse or even totally hidden	骨まで押さえないと拍動を感じられない脈で、沈脈よりも深い部位にあるか、もしくは完全に隠れている。
動脈		2.4.53	stirred pulse	a quick, jerky pulse, like a bouncing pea, slippery, rapid and forceful	豆が弾むような素早い発作的な脈で、滑りやすく、速く、力強い。
代脈		2.4.54	intermittent pulse	a moderate weak pulse, pausing at regular intervals	中等度に弱い脈で、規則的に停止する。
結脈		2.4.55	bound pulse	a moderate weak pulse, pausing at irregular intervals	中等度に弱い脈で、不規則に停止する。
促脈		2.4.56	skipping pulse	a rapid pulse with irregular intermittence	不規則に中断する数脈。
大脈		2.4.57	large pulse	a broad pulse with a bigger amplitude than normal	正常よりも振幅の大きい、幅広い脈。
軟脈		2.4.58	soft pulse	a pulse felt softer than normal	正常よりも柔らかく感じられる脈。
疾脈		2.4.59	racing pulse	a pulse having more than seven beats per respiration	1呼吸当たり7回以上拍動する脈。
怪脈		2.4.60	strange pulse	special pulses signifying critical conditions	危篤状態の前兆となる特殊な脈。
眞臟脈		2.4.61	true visceral pulse	a pulse condition indicating exhaustion of visceral qi	臓気の消耗を示す脈象。
雀啄脈		2.4.62	pecking sparrow pulse	an urgent rapid pulse of irregular rhythm that stops and starts, like a sparrow pecking for food	雀が餌をついばんでいるように、停止と開始のリズムが不規則な切迫した数脈。
釜沸脈		2.4.63	seething cauldron pulse	an extremely rapid floating pulse that is all outward movement and no inward movement, also known as bubble-rising pulse	すべて外側に向かい、内側に向かう動きのない極端に速い浮脈。bubble-rising pulseとしても知られる。
魚翔脈		2.4.64	waving fish pulse	a pulse that seems to be yet seems not to be present, like a fish waving in the water	存在しているようにも、していないようにも思える、魚が水中を動き回っているような脈。
蝦遊脈		2.4.65	darting shrimp pulse	a pulse that arrives almost imperceptibly and vanishes with a flick, like a darting shrimp	ほとんどわからない間にやって来て、パッと飛び跳ねて去ってゆく、機敏に動く海老のような脈。
屋漏脈		2.4.66	leaking roof pulse	a pulse that comes at long and irregular intervals, like water dripping from a leaky roof	雨漏りのように、脈来が長く不規則な脈。
解索脈		2.4.67	untwining rope pulse	a pulse, not loose, not tight, with an irregular rhythm like an untwining rope	結合していない縄のように、緩くもなくきつくもない不規則なリズムの脈。
彈石脈		2.4.68	flicking stone pulse	a sunken replete pulse that feels like flicking a stone with a finger	指で石を弾くような拍動が感じられる沈・実脈。
偃刀脈		2.4.69	upturned knife pulse	a pulse like a knife with the blade pointing upward, i.e., fine, string-like, and extremely tight	刃が上向きのナイフのような脈、すなわち、細・弦・極端な緊脈。
轉豆脈		2.4.70	spinning bean pulse	a pulse that comes and goes away, elusive like a spinning bean	豆を回転させたときのように、行ったり来たりしてつかまえにくい脈。

検索表示（原表示の複数語を分離）	IST原表示	Code	Term	Definition/Description	定義/記述
麻促脈		2.4.71	confused skipping pulse	a pulse extremely fine and faint, and urgent, skipping and chaotic	極端に細くかすかな脈で、切迫し、飛び飛びで進み、混沌としている。
離經脈		2.4.72	anomalous pulse	a sudden change of pulse condition in a pregnant woman when she is about to give birth	出産直前の妊婦における脈象の突然の変化。
按診		2.4.73	body palpation	examination of the body surface by touch with the hand or fingers	手または指で体表面を触れて診察すること。
腹診		2.4.74	abdominal examination	examination of the chest and abdomen by touching and pressing in combination with other examinations for the purpose of determining a morbid condition in diagnosis	触ったり押したりすることによる胸部および腹部の診察。診断の際には、罹患状態を判断するため、他の診察法と組み合わせる。
診尺膚		2.4.75	cubit skin examination	examination of the forearm with the hand for determining the texture of the skin, development of the muscles, and temperature of the extremities	前腕部と手の診察。肌触り、筋肉の発達、四肢温を判断する。
診虛裏		2.4.76	apical impulse examination	examination of the apical pulsation for determining the condition of the stomach qi and ancestral qi	心尖拍動の診察。胃気および宗気の状態を判断する。
叩擊法		2.4.77	tapping examination	examination of bone fracture or bone diseases with the longitudinal force induced by knocking gently on the distal end of the limb	骨折または骨疾患の診察。肢の遠位部を優しく叩きながら縦方向に力をかける。
按腧穴		2.4.78	acupuncture point palpation	palpation of the body surface at the sites of acupuncture points which reflect the condition of the corresponding visceral organs	経穴部位での体表の切診。対応する臓器の状態を反映する。
七惡		2.4.79	seven malign signs	seven signs indicating a poor prognosis of external lesions, i.e., poor heart, poor liver, poor spleen, poor lung, poor kidney, deteriorated viscera, and exhausted qi and blood	外部病変の予後不良を示す７つの徴候。すなわち、心不良、肝不良、脾不良、肺不良、腎不良、臓の悪化、気と血の消耗。
五善		2.4.80	five benign signs	five signs indicating a good prognosis of external lesions, i.e., good heart, good liver, good spleen, good lung and good kidney	外部病変の予後良好を示す5つの徴候。すなわち、心良好、肝良好、脾良好、肺良好、腎良好。
八綱辨證		**2.5.0**	**Eight Principle Pattern Identification/ Syndrome Differentiation**		
陰陽辨證		2.5.1	Yin-Yang Pattern Identification/ Syndrome Differentiation	categorization of patterns/syndromes according to yin-yang theory, e.g., interior, cold, and deficiency patterns/syndromes pertaining to yin, and exterior, heat, and excess patterns/syndromes pertaining to yang	陰陽学説に基づく、陰に関する証（裏証、寒証、虚証）と陽に関する証（表証、熱証、実証）の分類。
陰證		2.5.2	yin pattern/syndrome	a collective term for interior, cold and deficiency patterns/syndromes with inhibitory, hypofunctional, quiescent or dimmed manifestations, or inward and downward symptoms, as well as morbid conditions caused by pathogenic factors of yin nature	裏証、寒証、虚証の総称で、抑制、機能低下、静止・暗い象、内側・下方の症状、および陰の病邪による罹患状態を有する。
陽證		2.5.3	yang pattern/ syndrome	a collective term for exterior, heat and excess patterns/ syndromes with excitatory, hyperfunctional, restless or bright manifestations, or outward and upward symptoms, as well as morbid conditions caused by pathogenic factors of yang nature	表証、熱証、実証の総称で、興奮、機能亢進、運動・明るい象、外側・上方の症状、および陽の病邪による罹患状態を有する。
陰陽失調證		2.5.4	pattern/syndrome of yin-yang disharmony	any pattern/syndrome in which yin and yang are out of balance	陰陽の平衡が崩れている証。
傷陽證		2.5.5	yang damage pattern/syndrome	a pattern/syndrome characterized by damage to yang, resulting from various causes such as exuberance of yin-cold in the interior, excessive use of cold-natured medicinals, excessive sweating or diarrhea, usually manifested as fear of cold, pale complexion, spontaneous sweating, cold extremities and faint or even hardly perceptible pulse	傷陽を特徴とする証で、裏における陰寒が旺盛であること、寒性薬の過度の使用、過剰な発汗または泄瀉など、さまざまな原因で生じる。通常、畏寒、面色蒼白、自汗、四肢の冷えおよび昏厥が発現し、脈がほとんど感じられないことさえある。
傷陰證		2.5.6	yin damage pattern/syndrome	a pattern/syndrome characterized by damage to yin, often resulting from invasion of heat pathogen or in advanced cases of febrile disease with impairment of the liver and kidney yin, usually manifested as low fever, heat sensation in the palms and soles, emaciation, thirst, mala flush, dry and scarlet red tongue, and fine, feeble and rapid pulse	傷陰を特徴とする証で、しばしば熱邪の侵襲が原因で、または肝・腎気の障害を伴う進行した熱病症例で生じる。通常、低熱、手掌および足底の熱感、るいそう、口渇、頬部潮紅、乾燥した鮮紅色の舌、および細く弱々しい数脈が発現する。
陰虛證		2.5.7	yin deficiency pattern/syndrome	a pattern/syndrome resulting from deficiency of yin fluid and essence, incapable of restraining yang and usually manifested by emaciation, dizziness, tinnitus, dryness of the mouth and throat, constipation, dark-colored urine, afternoon fever, malar flush, night sweats, reddened tongue with scanty coating and rapid fine pulse; in Kampo medicine, it is understood as "yin pattern in combination with deficiency pattern"	陰津および陰精の虚により生じ、陽を抑制できなくなる証で、通常、るいそう、めまい、耳鳴、口および喉の乾燥、便秘、暗黒色の尿、午後の発熱、頬部潮紅、盗汗、少量の舌苔を伴う紅舌および数・細脈が発現する。漢方では、陰証と虚証の組み合わせとして理解される。
陽虛證		2.5.8	yang deficiency pattern/syndrome	a pattern/syndrome resulting from deficiency of yang qi with reduction in its warming and activating power, manifested by intolerance of cold, cold extremities, spontaneous sweating loose bowel, long voidings of clear urine, pale tongue, and deep and weak pulse; in Kampo medicine, it is understood as "yang pattern in combination with excess pattern"	陽気の虚により生じ、温め活性化する力の低下を伴う証で、寒冷不耐、四肢の冷え、自汗、泄瀉、長時間の清澄尿の排出、淡白舌、および弱・沈脈が発現する。漢方では、陽証と実証の組み合わせとして理解される。
陰陽兩虛證		2.5.9	pattern/syndrome of dual deficiency of yin and yang	a pattern/syndrome resulting from deficiency of both yin fluid and yang qi of the bowels and viscera, usually manifested as dizziness, tinnitus, lassitude, aversion to cold with cold limbs or vexing heat in the chest, palms or soles, palpitations, lumbar soreness, pale and dry tongue, and rapid weak pulse	臓腑の陰津および陽気の両方の虚により生じる証で、通常、めまい、耳鳴、倦怠感、四肢の冷えを伴う悪寒または胸部、手掌、足底の煩熱、心悸、腰痛、乾燥した淡白舌、および数・弱脈が発現する。

検索表示（原表示の複数語を分離）	IST原表示	Code	Term	Definition/Description	定義/記述
陰虚内熱證		2.5.10	pattern/syndrome of yin deficiency with internal heat	a pattern/syndrome resulting from deficiency of yin fluid with endogenous heat, usually manifested by persistent low fever or afternoon tidal fever, heat sensation in the chest, palms and soles, malar flush, night sweats, dry mouth with desire for drink, short voidings of dark-colored urine, constipation, reddened tongue lacking moisture and rapid fine pulse	陰津の虚および体内の熱により生じる証で、通常、持続性の低熱または午後潮熱、胸部、手掌および足底の熱感、頬部潮紅、盗汗、飲水欲求を伴う口乾、短時間の暗黒色の尿の排出、便秘、潤いのない紅舌および数・細脈が発現する。
陰虚火旺證		2.5.11	pattern/syndrome of yin deficiency with effulgent fire	a pattern/syndrome of effulgent fire arising from yin fluid deficiency, manifested by vexation, insomnia, dry throat and mouth, night sweating, nocturnal emission, flushed cheeks, bone-steaming tidal fever, dry and bound stool, short voidings of yellow urine, or hemoptysis, epistaxis, reddened tongue lacking moisture, or ulceration on the tongue and rapid fine pulse	陰津の虚により火が燃えさかる証で、心煩、失眠、喉および口の乾燥、盗汗、夢精、頬部潮紅、骨蒸潮熱、乾燥して固まった便、短時間の黄色尿の排出、または喀血、衄血、潤いのない紅舌または舌の潰瘍および数・細脈が発現する。
陰虚陽亢證		2.5.12	pattern/syndrome of yin deficiency with yang hyperactivity	a pattern/syndrome of yin fluid deficiency with yang hyperactivity, marked by tidal fever, night sweating, flushed cheeks, dizziness, blurred vision, agitation, insomnia, reddened tongue with scanty coating and rapid fine pulse	陰津の虚および陽の活動亢進の証で、潮熱、盗汗、頬部潮紅、めまい、目昏、煩躁、失眠、少量の舌苔を伴う紅舌および数・細脈が発現する。
陰虚津虧證		2.5.13	pattern/syndrome of yin deficiency with fluid depletion	a pattern/syndrome of yin fluid deficiency, manifested by dire thirst, dry skin, sunken eyes, short voidings of yellow urine, dry and bound stool, vexing heat in the chest, palms and soles, emaciation, night sweats, reddened tongue with scanty coating and rapid fine pulse	陰津の虚の証で、激しい口渇、皮膚の乾燥、落ちくぼんだ目、短時間の黄色尿の排出、乾燥して固まった便、胸部、手掌および足底の煩熱、るいそう、盗汗、少量の舌苔を伴う紅舌および数・細脈が発現する。
陰虚水停證		2.5.14	pattern/syndrome of yin deficiency with water retention	a pattern/syndrome of yin fluid deficiency complicated with retention of water, manifested by localized edema of limbs, reduced passage of urine, dry throat and mouth, vexing heat in the chest, palms and soles, constipation, reddened tongue with scanty coating and rapid fine pulse	陰津の虚と水停（水の停滞）が合併した証で、四肢の局所水腫、乏尿、喉および口の乾燥、胸部、手掌および足底の煩熱、便秘、少量の舌苔を伴う紅舌および数・細脈が発現する。
陰虚濕熱證		2.5.15	pattern/syndrome of yin deficiency and dampness-heat	a pattern/syndrome of yin-fluid deficiency complicated with dampness-heat obstruction, manifested by low fever, night sweating, flushed cheeks in the afternoon, vexing heat in the chest, palms and soles, bitter and greasy taste in the mouth, cumbersome limbs, reddened tongue with yellow slimy coating, and rapid fine pulse	陰津の虚と湿熱の閉塞が合併した証で、低熱、盗汗、午後の頬部潮紅、胸部、手掌および足底の煩熱、口の中が苦く油っぽい、四肢疼重、黄・膩苔のある紅舌および数・細脈が発現する。
陰虚血瘀證		2.5.16	pattern/syndrome of yin deficiency and blood stasis	a pattern/syndrome of yin fluid deficiency complicated with blood stasis, manifested by vexing heat in the chest, palms and soles, dry throat and mouth, low fever in the afternoon, local stabbing pain, expectoration of dark blood with clots, purple spots on the tongue, and fine rough (choppy) pulse	陰津の虚と血瘀が合併した証で、胸部、手掌および足底の煩熱、喉および口の乾燥、午後の低熱、局所刺痛、血餅の混じった暗黒色の血の喀出、舌の紫斑、粗い（むらのある）細脈が発現する。
陽虚氣滯證		2.5.17	pattern/syndrome of yang deficiency with qi stagnation	a pattern/syndrome of qi stagnation arising from yang deficiency, manifested by aversion to cold, cold limbs, pale complexion, painful distention in the chest, epigastrium and abdomen, borborygmi, sloppy stools, long voidings of clear urine, pale and enlarged tongue, and slow, sunken and weak pulse	陽虚による気滞の証で、悪寒、四肢の冷え、面色蒼白、胸部、心窩部、腹部の疼痛を伴う膨満、腹鳴、便溏、長時間の清澄尿の排出、淡白・胖大舌および遅・沈・弱脈が発現する。
陽虚濕阻證		2.5.18	pattern/syndrome of yang deficiency with dampness obstruction	a pattern/syndrome of dampness obstruction attributed to insufficient yang qi with disordered qi transformation, manifested by aversion to cold, cumbersome limbs, edema, inhibited urine, sloppy stool, reduced food intake and abdominal distension,	陽気不足による湿の閉塞および気化の障害の証で、悪寒、動かしにくい四肢、水腫、小便不利、便溏、摂食量減少および腹部膨満、白・滑苔を伴う膨らんだ淡白舌および遅・沈・弱脈が発現する。
陽虚水泛證		2.5.19	pattern/syndrome of yang deficiency with water flood	a pattern/syndrome arising when yang deficiency of the spleen and kidney leads to retention of water in the body, usually manifested by generalized edema, inhibited urine, palpitation, dyspnea, aversion to cold with cold limbs, abdominal distension, soggy diarrhea, pale and plump tongue with white slippery coating, and slow, sunken and week pulse	脾腎の陽虚により体内の水分が停滞するときに生じる証で、通常、全身の水腫、小便不利、心悸、喘、四肢の冷えを伴う悪寒、腹部膨満、濡泄、白・滑苔を伴う膨らんだ淡白舌および遅・沈・弱脈が発現する。
陽虚痰凝證		2.5.20	pattern/syndrome/ of yang deficiency with congealing phlegm	a pattern/syndrome of insufficient yang qi complicated with congealing phlegm, manifested by aversion to cold, cold limbs, dizziness, somnolence, oppression in the chest with profuse phlegm, obesity, or goiter, swollen, inflamed lymph glands, breast nodules, swollen and stiff joints, slimy tongue coating and slippery pulse	陽気不足と凝結した痰が合併した証で、悪寒、四肢の冷え、めまい、嗜睡、大量の痰を伴う胸悶、肥満、または癭、瘰癧、乳房結節、関節の腫大と強直、膩苔および滑脈が発現する。
陽虚寒凝證		2.5.21	pattern/syndrome of yang deficiency with congealing cold	a pattern/syndrome marked by aversion to cold, cold limbs, pain in the chest, epigastrium, abdomen, waist and knees relieved by warmth, and in women, late periods, painful periods, menstrual discharge of dark blood and clots, pale and enlarged tongue with white slippery coating, and slow sunken pulse	悪寒、四肢の冷え、温めると軽減する胸部、心窩部、腹部、手首および膝の疼痛を特徴とする証で、女性では月経の遅れ、月経痛、暗黒色の月経および血餅の排出が認められ、白・滑苔を伴う淡白・胖大舌および遅・沈脈が発現する。
陰血虧虛證		2.5.22	yin-blood depletion pattern/syndrome	a pattern/syndrome marked by emaciation, sallow complexion, low fever, flushed cheeks, numb limbs, dizziness, blurred vision, palpitations, insomnia, reddened tongue with scanty coating and rapid fine pulse	るいそう、面色委黄、低熱、頬部潮紅、四肢のしびれ、めまい、目昏、心悸、失眠、少量の舌苔を伴う紅舌および数・細脈を特徴とする証。
陰盛陽衰證		2.5.23	pattern/syndrome of yin exuberance with yang debilitation	a pattern/syndrome arising when exuberant yin cold leads to debilitation of yang qi, manifested by aversion to cold, cold limbs, long voidings of clear urine, diarrhea, or cold pain with preference for warmth, pale tongue with white coating, and slow sunken pulse	盛んな陰寒により陽気が衰退するときに生じる証で、悪寒、四肢の冷え、長時間の清澄尿の排出、泄瀉、または冷痛のため暖かさを好む、白苔を伴う淡白舌および遅・沈脈が発現する。
陰盛格陽證		2.5.24	pattern/syndrome of exuberant yin repelling yang	a pattern/syndrome in which exuberant internal cold forces yang qi to the outer body, giving rise to signs of true internal cold and false external heat	盛んな内寒のために陽気が体外に押しやられる証で、体内に真寒、体外に仮熱があるという徴候を引き起こす。
陰損及陽證		2.5.25	pattern/syndrome of detriment to yin affecting yang	a pattern/syndrome arising when long-term depletion of yin fluid causes damage to yang, resulting in deficiency of both yin and yang, in which yin deficiency is primary while yang deficiency secondary	長期の陰津の枯渇により傷陽が引き起こされるときに生じる証で、結果的に陰陽の両方が虚となる。この場合、陰虚が先に起こり、陽虚がそれに続く。

検索表示（原表示の複数語を分離）	IST原表示	Code	Term	Definition/Description	定義/記述
陽損及陰證		2.5.26	pattern/syndrome of detriment to yang affecting yin	a pattern/syndrome arising when long-term debilitation of yang qi causes damage to yin, resulting in deficiency of both yin and yang, in which yang deficiency is primary while yin deficiency secondary	長期の陽気の衰退により傷陰が引き起こされるときに生じる証で、結果的に陰陽の両方が虚となる。この場合、陽虚が先に起こり、陰虚がそれに続く。
陰竭陽脱證		2.5.27	pattern/syndrome of yin exhaustion and yang collapse	a critical pattern/syndrome of yin-essence exhaustion followed by yang collapse	陰精の消耗に続き亡陽が出現する危篤の証。
清陽不升證		2.5.28	pattern/syndrome of clear yang failing to ascend	a pattern/syndrome marked by dizziness, blurred vision, tinnitus and impaired hearing, aversion to cold with lack of warmth in the extremities, lassitude and lack of strength, anorexia, sloppy stool, pale tongue with white coating, and relaxed weak pulse	めまい、目昏、耳鳴および聴覚障害、四肢が温まらない悪寒、倦怠感および乏力、厭食、便溏、白苔を伴う淡白舌、および緩・弱脈を特徴とする証。
戴陽證		2.5.29	upcast yang pattern/ syndrome	a critical pattern/syndrome of true lower body cold and false upper body heat characterized by pale complexion with tidal reddening of the cheeks	下半身の真寒と上半身の仮熱を示す危篤の証で、面色蒼白と周期的な頬部潮紅を特徴とする。
亡陰證		2.5.30	yin collapse pattern/ syndrome	a serious pattern/syndrome resulting from exhaustion of yin fluid, manifested by thirst and craving for cold drink, flushed face, restlessness, dry tongue and rapid fine pulse	陰津の消耗により生じる重篤な証で、口渇および冷たい飲料水の渇望、顔面潮紅、落ち着きのなさ、舌乾および数・細脈が発現する。
亡陽證	亡陽證;陽脱證	2.5.31	yang collapse pattern/syndrome	a serious pattern/syndrome resulting from exhaustion of yang qi, manifested by pallor, dripping of cold sweats, cold limbs, pale and moistened tongue, and hardly perceptible pulse	陽気の消耗により生じる重篤な証で、蒼白、したたり落ちる冷汗、四肢の冷え、湿った蒼白舌が発現し、脈はほとんど触れない。
陽脱證	亡陽證;陽脱證	2.5.31	yang collapse pattern/syndrome	a serious pattern/syndrome resulting from exhaustion of yang qi, manifested by pallor, dripping of cold sweats, cold limbs, pale and moistened tongue, and hardly perceptible pulse	陽気の消耗により生じる重篤な証で、蒼白、したたり落ちる冷汗、四肢の冷え、湿った蒼白舌が発現し、脈はほとんど触れない。
表裏辨證		2.5.32	Exterior-Interior Pattern Identification/ Syndrome Differentiation	categorization of patterns/syndromes according to the location of the disease, in the exterior or in the interior, superficially seated or deeply seated	体外（表）か体内（裏）か、表在するか深在するかといった疾病部位による証の分類。
表證		2.5.33	exterior pattern/ syndrome	a general term for patterns/syndromes that occur chiefly at the early stage of external contractions affecting the exterior part of the body, characterized by a sudden onset, aversion to cold or to wind, fever, headache, generalized pain, thin tongue coating, and floating pulse	主に体外を冒す外感の早期に現れる証の一般用語で、卒発、悪寒または悪風、発熱、頭痛、身痛、薄苔および浮脈を特徴とする。
裏證		2.5.34	interior pattern/ syndrome	a general term for patterns/syndromes that indicate the existence of disease in the interior of the body such as bowels and viscera, qi and blood, or bone marrow	臓腑、気血または髄など、体内に疾病が存在することを示す証の一般用語。
半表半裏證		2.5.35	half-exterior half-interior pattern/ syndrome	a variety of patterns/syndromes due to affliction located between the exterior and interior of the body, marked by alternate fever and chills, fullness and choking feeling in the chest and costal region, bitter taste in the mouth, dry throat, nausea and loss of appetite, and string-like pulse	身体の表と裏の間にある苦痛により生じるさまざまな証で、寒熱往来、胸肋部の充満と窒息感、口苦、喉の乾燥、悪心と食欲不振、および弦脈が発現する。
表寒證	表寒證;風寒束表證	2.5.36	exterior cold pattern/ syndrome	a pattern/syndrome arising when wind-cold invades the exterior and characterized by pronounced aversion to cold with mild fever, headache, generalized pain, absence of sweating, thin white tongue coating, and tight floating pulse, the same as the pattern/syndrome of wind-cold fettering the exterior	風寒が表に侵入するときに生じる証で、微熱を伴う著しい悪寒、頭痛、身痛、無汗、薄・白苔および緊・浮脈を特徴とする。pattern/ syndrome of wind-cold fettering the exteriorと同義。
風寒束表證	表寒證;風寒束表證	2.5.36	exterior cold pattern/ syndrome	a pattern/syndrome arising when wind-cold invades the exterior and characterized by pronounced aversion to cold with mild fever, headache, generalized pain, absence of sweating, thin white tongue coating, and tight floating pulse, the same as the pattern/syndrome of wind-cold fettering the exterior	風寒が表に侵入するときに生じる証で、微熱を伴う著しい悪寒、頭痛、身痛、無汗、薄・白苔および緊・浮脈を特徴とする。pattern/ syndrome of wind-cold fettering the exteriorと同義。
表熱證	表熱證;風熱犯表證;風熱襲表證	2.5.37	exterior heat pattern/ syndrome	a pattern/syndrome arising when wind-heat invades the exterior and characterized by marked fever with mild aversion to wind and cold, headache, sore throat, sweating and thirst, thin yellow tongue coating and rapid floating pulse, the same as the pattern/ syndrome of wind-heat invading the exterior	風熱が表に侵入するときに生じる証で、軽度の悪風・悪寒を伴う著しい発熱、頭痛、喉の痛み、発汗および口渇、薄・黄苔および数・浮脈を特徴とする。pattern/ syndrome of wind-heat invading the exteriorと同義。
風熱犯表證	表熱證;風熱犯表證;風熱襲表證	2.5.37	exterior heat pattern/ syndrome	a pattern/syndrome arising when wind-heat invades the exterior and characterized by marked fever with mild aversion to wind and cold, headache, sore throat, sweating and thirst, thin yellow tongue coating and rapid floating pulse, the same as the pattern/ syndrome of wind-heat invading the exterior	風熱が表に侵入するときに生じる証で、軽度の悪風・悪寒を伴う著しい発熱、頭痛、喉の痛み、発汗および口渇、薄・黄苔および数・浮脈を特徴とする。pattern/ syndrome of wind-heat invading the exteriorと同義。
風熱襲表證	表熱證;風熱犯表證;風熱襲表證	2.5.37	exterior heat pattern/ syndrome	a pattern/syndrome arising when wind-heat invades the exterior and characterized by marked fever with mild aversion to wind and cold, headache, sore throat, sweating and thirst, thin yellow tongue coating and rapid floating pulse, the same as the pattern/ syndrome of wind-heat invading the exterior	風熱が表に侵入するときに生じる証で、軽度の悪風・悪寒を伴う著しい発熱、頭痛、喉の痛み、発汗および口渇、薄・黄苔および数・浮脈を特徴とする。pattern/ syndrome of wind-heat invading the exteriorと同義。
表虛證		2.5.38	exterior deficiency pattern/syndrome	a type of exterior pattern/syndrome resulting from attack by wind while the body’s resistance is lowered, manifested by intolerance of wind, persistent sweating, fever, headache, thin white tongue coating, and floating relaxed pulse	身体の抵抗が弱まっているときに風の攻撃を受けて生じる表証の一種で、風の不耐（intolerance of wind）、持続性の発汗、発熱、頭痛、薄・白苔および浮・緩脈が発現する。
表實證		2.5.39	exterior excess pattern/syndrome	an exterior pattern/syndrome with fever, aversion to cold, headache and generalized aching, forceful or tight floating pulse and absence of sweating, showing the external part of the body being attacked by cold, yet the patient’s defense qi is not damaged	発熱、悪寒、頭痛および身痛、力強いまたは緊・浮の脈および無汗がみられる表証で、体表部では寒の攻撃を受けているが、患者の衛気は障害されていないことを示す。

検索表示（原表示の複数語を分離）	IST原表示	Code	Term	Definition/Description	定義/記述
風濕襲表證		2.5.40	pattern/syndrome of wind-dampness assailing the exterior	a pattern/syndrome resulting from wind-dampness invading the exterior part of the body, manifested by heavy cumbersome limbs, dizziness with heavy head, aversion to cold, fever not relieved after sweating, oppression in the chest, no desire to drink, white slimy tongue coating and soggy relaxed pulse	風湿が体表部に侵入するために生じる証で、重く動かしにくい四肢、頭重を伴うめまい、悪寒、発汗後にも消失しない発熱、胸悶、飲水渇望がない、白・膩苔および濡・緩脈を特徴とする。
暑濕襲表證		2.5.41	pattern/syndrome of summerheat-dampness assailing the exterior	a pattern/syndrome resulting from summer-dampness attacking the exterior part of the body, manifested by fever, slight aversion to wind and cold, fatigued and cumbersome body, heavy head as if swathed, grimy face, thirst and vexation, reddened tongue with yellow slimy coating and rapid soggy pulse	暑湿（summer-dampness）が体表部を攻撃するために生じる証で、発熱、軽度の悪風・悪寒、疲労および動かしにくい体、何かを巻き付けられたような頭重、垢に覆われた顔（grimy face）、口渇および心煩、黄・膩苔を伴う紅舌および数・濡脈が発現する。
衛表不固證		2.5.42	defense-exterior insecurity pattern/syndrome	a pattern/syndrome marked by fear of cold spontaneous sweating, shortness of breath, lack of strength, pale tongue and weak pulse	畏寒、自汗、短気、乏力、淡白舌および弱脈が発現する証。
裏寒證		2.5.43	interior cold pattern/syndrome	a type of interior syndrome attributable to deficiency of yang qi of bowels and viscera or external cold directly attacking on or passing to the interior, mainly manifested by intolerance of cold with cold limbs, somber white complexion, absence of thirst or liking for hot drinks, long voidings of clear urine, pale tongue with white moistened coating and slow sunken pulse	臟腑の陽気虚または外寒が直接、体内で、または体内を通って攻撃するために生じる裏証の一種で、主に四肢の冷えを伴う寒冷不耐、くすんだ白い面色、口渇がないまたは熱い飲料水の渇望、長時間の清澄尿の排出、白・潤苔を伴う淡白舌および遅・沈脈が発現する。
裏熱證		2.5.44	interior heat pattern/syndrom e	a type of interior pattern/syndrome with exuberant heat arising when external pathogens enter the internal organs and transforming into heat, mainly manifested by fever with sweating, thirst with intake of fluid, vexation, bitter taste in the mouth, short voiding of reddish urine, a reddened tongue with yellow coating, and rapid surging or rapid string-like pulse	外邪が内臓に進入することにより盛んな熱および化熱がみられる裏証で、主に発汗を伴う発熱、水分摂取を伴う口渇、心煩、口苦、短時間の赤色尿の排出、黄苔を伴う紅舌、および数・洪脈または数・弦脈が発現する。
裏虛證		2.5.45	interior deficiency pattern/syndrome	a type of interior syndrome attributable to deficiency of qi, blood, yin, and yang of the internal organs with diminished function, usually manifested by shortage of qi, reluctance to speak, listlessness, lassitude, palpitations, dizziness, tinnitus, decreased food intake, aching lumbus and limp knees, pale tender-soft tongue and weak pulse	内臓の気、血、陰、陽の虚および機能低下により生じる裏証の一種で、通常、少気、話すのが億劫、無気力、倦怠感、心悸、めまい、耳鳴、摂食量減少、腰部の酸痛および跛行、淡白・嫩舌および弱脈が発現する。
裏實證		2.5.46	interior excess pattern/syndrom e	(1) any pattern/syndrome resulting from external pathogen transforming into heat and entering the interior to bind in the stomach and intestines, manifested by high fever, vexing thirst, abdominal pain and constipation; (2)a general term for patterns/syndromes caused by retention of phlegm, blood stasis, food stagnation or accumulation of parasitic worms	（1）外邪が化熱し、体内に進入して胃腸で結合することにより生じる証で、壮熱、わずらわしい口渇、腹痛および便秘が発現する、（2）痰の貯留、血瘀、食物停滞または寄生虫寄生により生じる証の一般用語。
表裏俱寒證		2.5.47	pattern/syndrom e of dual exterior and interior cold	a pattern/syndrome arising when cold pathogen attacks the exterior and interior of the body simultaneously, usually manifested by aversion to cold with cold limbs,headache and generalized pain, cold pain in the abdomen, vomiting and diarrhea, cough and dyspnea with whitish expectoration, pale tongue with white coating and tight pulse, also called the exterior-interior cold pattern/syndrome	寒邪が身体の表裏を同時に攻撃するときに生じる証で、通常、四肢の冷えを伴う悪寒、頭痛および身痛、腹部の冷痛、嘔吐および泄瀉、白食の喀出物を伴う咳嗽および喘、白苔を伴う淡白舌および緊脈が発現する。exterior-interior cold pattern/syndomeとも呼ばれる。
表裏俱熱證		2.5.48	pattern/syndrom e of dual exterior and interior heat	a heat pattern/syndrome involving both the exterior and the interior, caused either by heat pathogen entering the interior before the exterior pattern/syndrome is resolved or by superimposition of an exterior pattern/syndrome of newly contracted warm disease on a pre-existing interior pattern/syndrome, usually manifested by fever, aversion to wind, headache, flushed face, thirst, vexation or even delirium, constipation, dark-colored urine, reddened tongue with dry yellow coating and rapid forcefeul floating pulse, also called the exterior-interior heat pattern/syndrome	表証が消失する前に裏に進入した熱邪、またはすでに存在している裏証と新規発症した湿病の表証の重複のいずれかにより生じる、表裏両方に関与する熱証で、通常、発熱、悪風、頭痛、顔面潮紅、口渇、いらだたしさまたは譫妄、便秘、暗黒色の尿、燥・黄苔を伴う紅舌および力強い数・浮脈が発現する。exterior-interior heat pattern/syndomeとも呼ばれる。
表裏俱實證		2.5.49	pattern/syndrome of dual exterior and interior excess	an excess pattern/syndrome involving both the exterior and interior, usually manifested by fever without sweating, headache with stiff painful nape, irritability, abdominal distension and constipation	表裏両方に関与する実証で、通常、発汗を伴わない発熱、頭項強痛、いらいらする、腹部膨満および便秘が発現する。
表裏俱虛證		2.5.50	pattern/syndrome of dual exterior and interior deficiency	a deficiency pattern/syndrome involving both the exterior and the interior, usually manifested by sweating with aversion to wind, shortage of qi and reluctance to speak, decreased food intake, loose bowels, pale tongue and weak pulse	表裏両方に関与する虚証で、通常、悪風を伴う発汗、少気および話すのが億劫、摂食量減少、下痢、淡白舌および弱脈が発現する。
表寒裏熱證	表寒裏熱證<br>外寒裏熱證	2.5.51	pattern/syndrome of exterior cold and interior heat	(1) a pattern/syndrome in which cold pathogen fetters the exterior and depressed heat stays in the interior, mainly manifested by fever without sweat and with aversion to cold, headache and generalized pain, vexation, shortness of breath, fullness of the chest, thirst, reddened tongue with mixed yellow and white coating, and floating tight pulse; (2) a pattern/syndrome marked by true heat in the interior with external pseudo-cold manifestations	（1）寒邪が表を捕らえ、弱まった熱が裏にとどまるといった雑然とした症例で、主に発汗がなく悪寒を伴う発熱、頭痛および身痛、心煩、短気、胸部充満、口渇、黄色と白色の舌苔が混在する紅舌、および浮・緊脈が発現する、（2）裏の真熱と表の仮寒を特徴とする証。
外寒裏熱證	表寒裏熱證<br>外寒裏熱證	2.5.51	exterior cold and interior heat pattern/syndrome	(1) a pattern/syndrome in which cold pathogen fetters the exterior and depressed heat stays in the interior, mainly manifested by fever without sweat and with aversion to cold, headache and generalized pain, vexation, shortness of breath, fullness of the chest, thirst, reddened tongue with mixed yellow and white coating, and floating tight pulse; (2) a pattern/syndrome marked by true heat in the interior with external pseudo-cold manifestations	（1）寒邪が表を捕らえ、弱まった熱が裏にとどまるといった雑然とした症例で、主に発汗がなく悪寒を伴う発熱、頭痛および身痛、心煩、短気、胸部充満、口渇、黄色と白色の舌苔が混在する紅舌、および浮・緊脈が発現する、（2）裏の真熱と表の仮寒を特徴とする証。

検索表示（原表示の複数語を分離）	IST原表示	Code	Term	Definition/Description	定義/記述
表熱裏寒證		2.5.52	pattern/syndrome of exterior heat and interior cold	a pattern/syndrome with pre-existing insufficient yang qi complicated by contraction of wind-heat, manifested by fever, slight aversion to cold, headache and generalized pain, cough, loose bowels, pale and flabby tongue with thin yellow coating and rapid floating pulse	すでに存在している陽気不足に、風熱の感受が合併した証で、発熱、軽度の悪寒、頭痛および身痛、咳嗽、下痢、黄・薄苔を伴う弛緩した淡白舌および数・浮脈が発現する。
表虚裏實證		2.5.53	pattern/syndrome of exterior deficiency and interior excess	a pattern/syndrome in which insufficiency of defense is complicated by pathogens bound in the interior, manifested by symptoms of exterior deficiency such as aversion to wind and spontaneous sweating, together with symptoms of interior excess such as abdominal pain, constipation and yellow thick tongue coating	衛気不足が、裏で結合した邪気により悪化する証で、悪風および自汗などの表虚の症状と同時に、腹痛、便秘および黄・厚苔などの裏実の症状が発現する。
表實裏虛證		2.5.54	pattern/syndrome of exterior excess and interior deficiency	a pattern/syndrome in which exterior excess and interior deficiency occurring simultaneously, manifested by aversion to cold, fever with absence of sweating, listlessness and lassitude, shortness of breath, anorexia, palpitations, white tongue coating and floating pulse	表実と裏虚が同時に生じる証で、悪寒、無汗を伴う発熱、無気力および倦怠感、短気、厭食、心悸、白苔および浮脈が発現する。
內閉外脫證		2.5.55	pattern/syndrome of internal block and external collapse	a pattern/syndrome in which excess pathogens are trapped in the interior (as manifested by fever, cough and dyspnea, or by abdominal pain with tenesmus, or by constipation and urinary block, or by colicky pain in the chest, epigastrium and abdomen) while the healthy qi collapses (as manifested by pallor, reversal cold of limbs, cold dripping sweats, feeble breathing and scarcely perceptible pulse)	過剰な邪気が裏で捕捉され（発熱、咳嗽および喘、または裏急後重を伴う腹痛、または便秘および尿閉、または胸部、心窩部、腹部の疝痛が発現）、一方で正気が虚脱する証（蒼白、手足厥冷、したたり落ちる冷汗、弱々しい呼吸およびほとんど触れない脈が発現）。
寒熱辨證		2.5.56	Cold-Heat Pattern Identification/ Syndrome Differentiation	a method of pattern identification/syndrome differentiation in which symptoms are categorized as heat or cold, thereby determining the nature of the disease	症状を熱または寒によって分類し、これにより病性を判断する弁証論治の方法。
寒證		2.5.57	cold pattern/ syndrome	a general term for patterns/syndromes caused either by external cold pathogen or by insufficient yang within the body, commonly manifested by aversion to cold or fear of cold, cold pain with preference for heat, absence of thirst, thin clear sputum and nasal mucus, long voidings of clear urine, loose bowels, white facial complexion, pale tongue with white coating, and tight or slow pulse	体外の寒邪または体内の陽不足のいずれかにより生じる証の一般用語で、悪寒または畏寒、熱を好む冷痛、口渇の欠如、希薄な透明の痰および鼻粘液、長時間の清澄尿の排出、下痢、白い顔色、白苔を伴う淡白舌および緊脈または遅脈がよく発現する。
熱證		2.5.58	heat pattern/ syndrome	a general term for patterns/syndromes resulting either from attack of external heat or from prevalence of yang qi, usually manifested by fever, aversion to heat and liking for cold, thirst, flushed face, irritability and vexation, thick yellow sputum and nasal mucus, short voidings of dark-colored urine, constipation, reddened tongue with yellow coating, and rapid pulse	外熱の攻撃または陽気の勢いのいずれかにより生じる証の一般用語で、通常、発熱、悪熱および冷えたものを欲する、口渇、顔面潮紅、いらいらおよび心煩、粘稠な黄色の痰および鼻粘液、短時間の暗黒色の尿の排出、便秘、黄苔を伴う紅舌、および数脈が発現する。
寒熱錯雜證		2.5.59	cold-heat complex pattern/syndrome	any pattern/syndrome characterized by complicated heat and cold conditions such as heat in the upper part with cold in the lower part of the body, cold in the upper part with heat in lower part of the body, heat in the exterior and cold in the interior of the body, or cold in the exterior and heat in the interior of the body	上熱下寒、上寒下熱、表熱裏寒、表寒裏熱など、熱と寒の合併を特徴とする証。
上寒下熱證		2.5.60	upper cold and lower heat pattern/syndrome	any pattern/syndrome with cold symptoms in the upper part of the body and heat symptoms in the lower	上半身は寒の症状、下半身は熱の症状を示す証。
上熱下寒證		2.5.61	upper heat and lower cold pattern/syndrome	any pattern/syndrome with heat symptoms in the upper part of the body and cold symptoms in the lower	上半身は熱の症状、下半身は寒の症状を示す証。
眞寒假熱證		2.5.62	true cold with false heat pattern/syndrome	a pattern/syndrome arising when exuberant internal cold forces yang qi to outer body, manifested by subjective feverishness, flushing face, agitation, thirst and sore throat occurring simultaneously with coolness of the chest and abdomen to touch, reversal cold of the limbs, long voidings of clear urine, diarrhea with undigested food, and pale tongue with white coating and sunken string-like pulse	盛んな 内寒により陽気が体外に押しやられるときに生じる証で、主観的な熱っぽさ、顔面潮紅、煩躁、口渇、喉の痛みおよび大・浮脈と同時に、触ると冷たい胸部および腹部、手足厥冷、長時間の清澄尿の排出、未消化の食物を含む泄瀉、白苔を伴う淡白舌および弦・沈脈が発現する。
眞熱假寒證		2.5.63	true heat with false cold pattern/syndrome	a pattern/syndrome arising when exuberant pathogenic heat entraps yang qi in the interior, manifested by cold limbs, aversion to cold or even shivering, impaired consciousness, dark purple complexion, but on the other hand, high fever with the chest and abdomen hot to touch and hot breath exhaled from the mouth and nose, fetid mouth odor, coarse breathing, dire thirst, short voidings of yellow urine, reddened tongue with yellow dry coating and rapid string-like pulse	盛んな熱邪により陽気が体内に閉じこめられるときに生じる証で、四肢の冷え、悪寒または寒戦、意識障害、黒紫色の顔色が発現する一方で、触ると熱い胸部および腹部を伴う壮熱および口・鼻からの熱い呼気、口臭、荒い呼吸、わずらわしい口渇、短時間の黄色尿の排出、黄・燥苔を伴う紅舌および弦・速脈も発現する。
熱閉證		2.5.64	heat block pattern/ syndrome	a pattern/syndrome that arises when a heat pathogen congests in the bowels, viscera and meridians/ channels, blocking normal movement out of the body	熱邪が臓腑・経絡内に充満するときに生じる証で、体外への正常な動きができなくなる。
熱極生風證	熱極生風證;熱極動風證	2.5.65	pattern/syndrome of extreme heat engendering wind	a liver wind pattern/syndrome attributed to exuberant pathogenic heat, and marked by high fever with restlessness, convulsions, opisthotonos, and impaired consciousness	盛んな熱邪により生じる肝風証で、落ち着きのなさを伴う壮熱、抽搐、弓なり緊張および意識障害を特徴とする。
熱極動風證	熱極生風證;熱極動風證	2.5.65	pattern/syndrome of extreme heat engendering wind	a liver wind pattern/syndrome attributed to exuberant pathogenic heat, and marked by high fever with restlessness, convulsions, opisthotonos, and impaired consciousness	盛んな熱邪により生じる肝風証で、落ち着きのなさを伴う壮熱、抽搐、弓なり緊張および意識障害を特徴とする。
虛實辨證		2.5.66	deficiency-excess Pattern Identification/ Syndrome differentiation	categorization of pattern identification/syndrome differentiation in which the strength of the healthy qi and pathogenic qi are compared, thereby determining the nature of the disease	正気と邪気の力を比較し、それにより病性を判断する弁証論治の分類。

検索表示（原表示の複数語を分離）	IST原表示	Code	Term	Definition/Description	定義/記述
虛證		2.5.67	deficiency pattern/ syndrome	a general term for patterns/syndromes caused by deficiency of the healthy qi (including deficiency of yin, yang, qi and blood)	正気虚（陰、陽、気、血の虚を含む）により生じる証の一般用語。
實證		2.5.68	excess pattern/syndrome	a general term for patterns/syndromes caused by external pathogenic factors such as six excesses, pestilential pathogens, worms and toxins, or by accumulated pathological products due to dysfunction of internal organs, such as phlegm, retained fluid, water, dampness, pus, static blood and retained food	六淫、有害な病原体・寄生虫・毒素などの外邪、または痰、飲、水、湿、膿、瘀血、宿食など、内臓の機能障害による病的な産物の蓄積に起因する証の一般用語。
虛寒證		2.5.69	deficiency cold pattern/syndrome	a cold pattern/syndrome arising from deficiency of yang qi, the same as the yang deficiency pattern/ syndrome	陽気虚に起因する寒証。陽虚証と同義。
虛熱證		2.5.70	deficiency heat pattern/syndrome	a heat pattern/syndrome caused by deficiency of yin, qi, blood or fluids	陰、気、血、津の虚に起因する熱証。
虛實挾雜證		2.5.71	deficiency-excess complex pattern/ syndrome	a pattern/syndrome arising from struggle between the healthy and pathogenic qi while excessive pathogens and insufficient healthy qi exist simultaneously	過剰な邪気と正気不足が同時に存在するとき、正気と邪気の間で競争が起こり生じる証。
上盛下虛證		2.5.72	upper exuberance and lower deficiency pattern/syndrome	a pattern/syndrome marked by yin deficiency of the liver and kidney in the lower part of the body together with accumulation of qi, static blood or phlegm in the upper part of the body, the same as the excess above and deficiency below pattern/syndrome	下半身における肝腎の陰虚とともに上半身における気、瘀血または痰の蓄積を特徴とする証。excess above and deficiency below pattern/syndromeと同義。
眞虛假實證		2.5.73	true deficiency with false excess pattern/ syndrome	any pattern/syndrome deficient in nature showing some deceptive symptoms of excess	外面的にはいくつかの実の症状を示す虚証。
眞實假虛證		2.5.74	true excess with false deficiency pattern/ syndrome	any pattern/syndrome excess in nature showing some deceptive symptoms of deficiency	外面的にはいくつかの虚の症状を示す実証。
精氣虧虛證		2.5.75	essential qi deficiency pattern/syndrome	a pattern/syndrome arising from deficiency of essential qi, marked by emaciation, dizziness, tinnitus, short and slight of stature, slow in movements, low intelligence, or by infertility, sterility, impotence and premature ejaculation	精気の虚に起因する証で、るいそう、めまい、耳鳴、低身長およびきゃしゃな体形、動作緩慢、知的障害、または不孕、生殖不能、陽痿および早泄を特徴とする。
病因辨證		**2.6.0**	**Disease Cause Pattern Identification/ Syndrome Differentiation**		
病因辨證		2.6.1	disease cause pattern identification/ syndrome differentiation	categorization of patterns/syndromes according to pathological conditions attributable to different kinds of causal factors for making diagnosis	診断のために、種々の病因による病的状態に基づき証を分類すること。
外風證		2.6.2	external wind pattern/ syndrome	a general term for patterns/syndromes caused by external wind pathogen alone or together with other pathogenic factors such as dampness, heat, or pestilent toxin	外風の邪が単独で、または湿、熱、有害な毒など、その他の病邪と組み合わさって生じる証の一般用語。
內風證		2.6.3	internal wind pattern/ syndrome	any pattern/syndrome of internal wind stirring in the interior, manifested by dizziness and blurred vision, numbness of the limbs, tremors, convulsions or pruritus	裏で動き回る内風の証で、めまいおよび目昏、四肢の麻木、顫震、抽搐または掻痒が発現する。
中風閉證		2.6.4	wind-stroke block pattern/syndrome	a pattern/syndrome of wind-stroke characterized by blockage of the orifices manifested by sudden loss of consciousness, hemiplegia, clenched jaw and clenched hands	開口部の遮断を特徴とする中風の証で、突然の神昏、半身不随、固く閉じた顎および固く握りしめた手が発現する。
中風脫證		2.6.5	wind-stroke collapse pattern/syndrome	a pattern/syndrome of wind-stroke characterized by desertion of yang qi, manifested by sudden loss of consciousness, closed eyes with opened mouth, faint breathing, flaccid paralysis of the limbs, profuse cold sweats, and incontinence of urine and feces	陽気の脱出を特徴とする中風の証で、突然の神昏、開口を伴う閉眼、弱々しい呼吸、弛緩性四肢麻痺、大量の冷汗、小便失禁・大便滑脱が発現する。
實熱證		2.6.6	excess heat pattern/ syndrome	a pattern/syndrome caused by excessive pathogenic heat while the body resistance is still sufficient with intense reaction, marked by high fever with restlessness, constipation, large or slippery rapid pulse	熱邪は過剰（実）であるものの、身体の抵抗性がまだ十分にあり強く反応することにより生じる証で、落ち着きのなさを伴う壮熱、便秘、大または滑・数脈を特徴とする。
實寒證		2.6.7	excess cold pattern/ syndrome	any pattern/syndrome due to contraction of yin cold	陰寒の感受により生じる証。
中寒證		2.6.8	pattern/syndrome of cold in the middle	a deficiency-cold pattern/syndrome of the middle energizer arising from insufficiency of yang qi and impaired splenic transportation and transformation, manifested by abdominal pain that can be relieved by warmth and pressure, cold limbs, reduced food intake, loose stool, etc.	陽気不足および脾の運化障害により生じる中焦の虚寒証で、温め圧迫すると消失する腹痛、四肢の冷え、摂食量減少、軟便などを特徴とする。
暑證		2.6.9	summerheat pattern/ syndrome	any pattern/syndrome resulting from contraction of summerheat and marked by high fever with sweating, thirst, shortness of breath, lassitude, cumbersome limbs, short voidings of dark-colored urine, reddened tongue and rapid vacuous pulse	暑邪の感受により生じる証で、発汗を伴う壮熱、口渇、短気、倦怠感、動かしにくい四肢、短時間の暗黒色の尿の排出、紅舌および数・虚脈を特徴とする。
濕證		2.6.10	dampness pattern/ syndrome	any pattern/syndrome resulting from contraction of external dampness or from dampness arising within, marked by physical fatigue, heavy cumbersome limbs, heavy-headedness, poor appetite, abdominal distention, sloppy stool, slippery and slimy tongue coating, and soggy relaxed pulse	外湿または内部で生じた湿邪の感受に起因する証で、身体的疲労、重く動かしにくい四肢、頭重感、食欲不振、腹部膨満、便溏、滑・膩苔および濡・緩脈を特徴とする。
外燥證		2.6.11	external dryness pattern/syndrome	a general term for patterns/syndromes caused by climatic dryness, commonly manifested by dry skin, dry nose, mouth and throat or dry cough	乾燥した気候により生じる証の一般用語で、一般に皮膚、鼻、口、喉の乾燥または乾咳を特徴とする。
內燥證		2.6.12	internal dryness pattern/syndrome	any pattern/syndrome of dryness due to consumption of body fluids, manifested by emaciation, withered skin, dry throat, parched lips, thirst, oliguria, constipation, furless reddened tongue and rapid fine pulse	体液の消耗による燥証で、るいそう、皮膚のひび割れ、喉の乾燥、乾いた唇、口渇、乏尿、便秘、舌苔のない紅舌および数・細脈が発現する。

検索表示（原表示の複数語を分離）	IST原表示	Code	Term	Definition/Description	定義/記述
凉燥證		2.6.13	cool dryness pattern/ syndrome	pattern/syndrome due to coolness and dryness in autumn, marked by headache, more chills than fever, absence of sweating, dryness of the nasal cavity and lips, cough with scanty expectoration, thin and dry whitish tongue fur, and floating and tense pulse	秋の冷却および乾燥により生じる証で、頭痛、発熱よりも寒気、無汗、鼻腔および唇の乾燥、少量の喀出物を伴う咳嗽、薄・燥・白苔、浮・緊脈を特徴とする。
溫燥證		2.6.14	warm dryness pattern/syndrome	pattern/syndrome caused by exopathic warmth and dryness in autumn, marked by fever with slight aversion to wind and cold, dryness of the nasal cavity and lips, thirst, dry cough with scanty expectoration, fidgetiness, thin yellowish tongue fur, and rapid and floating pulse	秋の外因性の暖かさおよび乾燥により生じる証で、軽度の悪風・悪寒を伴う発熱、鼻腔および唇の乾燥、口渇、少量の喀出物を伴う乾咳、そわそわする、薄・黄苔および数・浮脈を特徴とする。
燥乾清竅證		2.6.15	pattern/syndrome of dryness affecting the clear orifices	a pattern/syndrome marked by dry nose, mouth and eyes with lack of nasal mucus, saliva and tears	鼻粘液、唾液、涙を伴わない鼻、口、目の乾燥を特徴とする証。
燥結證		2.6.16	dryness bind pattern/ syndrome	a pattern/syndrome marked by constipation due to deficiency of fluids in the gastrointestinal tract	胃腸の津の虚による便秘を特徴とする証。
實火證		2.6.17	excess fire pattern/ syndrome	a pattern/syndrome caused by intense pathogenic fire, most commonly affecting the stomach and intestines or the liver and gallbladder, and characterized by high fever, headache, red eyes, bitter taste in the mouth, dry mouth and thirst for cold drink	激しい火邪により生じる証で、胃腸または肝・胆の罹患が最も多く、壮熱、頭痛、目の充血、口苦、口乾、口渇のため冷たい飲料をほしがることを特徴とする。
火熱證		2.6.18	fire-heat pattern/ syndrome	any pattern/syndrome of heat and fire, either contracted externally or engendered internally	体外で感受するか、または体内で発生する熱と火の証。
虛火上炎證		2.6.19	pattern/syndrome of deficiency fire flaming upward	a pattern/syndrome arising when insufficient yin fails to inhibit fire, marked by dry and sore throat, vexation, insomnia, dizziness, tinnitus, forgetfulness, heat in the palms and soles, night sweating, or red eyes, ulcers of the tongue or mouth, reddened tender-soft tongue and rapid fine pulse	陰の不足により火が阻害されるときに生じる証で、喉の乾燥および痛み、心煩、失眠、めまい、耳鳴、健忘症、手掌および足底の熱、寝汗または目の充血、舌または口の潰瘍、紅・嫩舌および数・細脈を特徴とする。
膿證		2.6.20	pus pattern/syndrome	a pattern/syndrome marked by purulent discharge from an ulcerative lesion with stinking fetid smell, accompanied by fever, thirst, curdy and slimy tongue coating, and rapid slippery pulse	潰瘍性病変からの著しい悪臭を放つ膿性分泌物とともに、発熱、口渇、腐・膩苔および数・滑脈を特徴とする証。
食積證		2.6.21	food accumulation pattern/syndrome	a pattern/syndrome marked by epigastric and abdominal distention, vomiting of sour matter, anorexia, offensive odor of stools, curdy and slimy tongue coating, the same as the food stagnation pattern/syndrome	心窩部および腹部の膨満、酸っぱい物質の嘔吐、厭食、大便の悪臭、腐・膩苔を特徴とする証。food stagnation pattern/syndromeと同義。
蟲積證		2.6.22	worm accumulation pattern/syndrome	a general term for patterns/syndromes caused by intestinal accumulation of parasitic worms, often marked by abdominal distention or pain, emaciation, lack of strength and sallow complexion	寄生虫の腸への寄生により生じる証の一般用語で、しばしば、腹部の膨満または疼痛、るいそう、乏力および面色萎黄を特徴とする。
風痰證		2.6.23	wind-phlegm pattern/syndrome	a pattern/syndrome attributable either to external wind with phlegm or to liver wind with phlegm, marked by expectoration of foamy sputum, fullness and oppression in the chest, dizziness, and distending pain of the head and eyes, or marked by phlegmatic sounds in the throat, numbness of limbs, and even loss of consciousness with aphasia, and deviated eye and mouth	痰を伴う外風または痰を伴う肝風に起因する証で、泡状の痰の喀出、胸部の充満および胸悶、めまい、頭部および目の脹痛、または喉で痰がからむ音、四肢の麻木、および失語症を伴う神昏、口眼喎斜を特徴とする。
寒痰證		2.6.24	cold-phlegm pattern/syndrome	a pattern/syndrome marked by cough with whitish expectoration, dyspnea or wheezing, aversion to cold with cold limbs, white slimy tongue coating, and wiry slippery or tense pulse	白色の喀出物を伴う咳嗽、喘または哮、四肢の冷えを伴う悪寒、白・膩苔および針金様の滑脈または緊脈を特徴とする証。
熱痰證		2.6.25	heat-phlegm pattern/syndrome	a pattern/syndrome arising when turbid phlegm combined with pathogenic heat accumulates in the lung and harasses the heart, marked by cough with yellowish expectoration, vexing stuffiness in the chest, fever, thirst, palpitations, insomnia, short voidings of deep-colored urine, reddened tongue with yellow greasy slimy coating and rapid slippery pulse	痰濁とともに邪熱が肺に蓄積し、心を苦しめるときに生じる証で、黄色の喀出物を伴う咳嗽、わずらわしい胸部の閉塞感、発熱、口渇、心悸、失眠、短時間の暗黒色の尿の排出、ベトベトした黄・膩苔を伴う紅舌および数・滑脈を特徴とする。
燥痰證		2.6.26	dryness-phlegm pattern/syndrome	a pattern/syndrome due to accumulation of dryness-heat and phlegm-turbidity in the lung, marked by cough with scanty sticky sputum difficult to spit out, or blood streaked sputum, chest pain with oppression feeling, dry nose and mouth, tongue with scanty moisture but slimy coating, and thin choppy pulse	肺における燥熱および痰濁の蓄積により生じる証で、喀出困難な少量の粘着性の痰または血の付着した痰を伴う咳嗽、重圧感を伴う胸痛、鼻および口の乾燥、潤いは少ないが膩苔を伴う舌、および不規則に変わる細脈を特徴とする。
瘀痰證		2.6.27	blood stasis-phlegm pattern/syndrome	a pattern/syndrome arising when turbid phlegm combined with static blood obstructs the qi movement, marked by formation of masses with local stabbing pain, or numbness and wilting of the limb, oppression in the chest with expectoration of profuse sputum or dark bloody sputum, purple tongue or purple spots on the tongue, slimy coating and string-like choppy pulse	痰濁とともに瘀血が気機を閉塞するときに生じる証で、局所刺痛を伴う腫瘤形成、または肢の麻木および衰弱、大量の痰または暗黒色の血痰の喀出を伴う胸悶、紫舌または紫斑のある舌、膩苔および不規則に変わる弦脈を特徴とする。
膿痰證		2.6.28	purulent phlegm pattern/syndrome	a pattern/syndrome marked by expectoration of pus or purulent sputum	膿または膿痰の喀出を特徴とする証。
濕痰證		2.6.29	dampness-phlegm pattern/syndrome	a pattern/syndrome due to accumulation of dampness phlegm in the lung, marked by cough with profuse expectoration, heaviness sensation of the limbs, feeling of stuffiness in the chest, reduced food intake, stickiness of the mouth, white slimy tongue coating and slippery pulse	肺における湿痰の蓄積により生じる証で、大量の喀出物を伴う咳嗽、四肢が重い感じ、胸部が詰まった感じ、摂食量減少、口のねばつき、白・膩苔および滑脈を特徴とする。
痰氣互結證		2.6.30	pattern/syndrome of binding of phlegm and qi	a pattern/syndrome marked by depressed mood, insomnia, dream-disturbed sleep, a sensation of a foreign body in the throat which can be neither swallowed nor ejected, fullness and oppression in the chest, and profuse sputum; white and slimy tongue coating, and slippery string-like pulse	抑うつ気分、失眠、夢に邪魔される睡眠、飲み込んだり排出したりできない喉の異物感、胸部の充満および胸悶、および大量の痰、白・膩苔および滑・弦脈を特徴とする証。

検索表示（原表示の複数語を分離）	IST原表示	Code	Term	Definition/Description	定義/記述
痰熱内擾證		2.6.31	pattern/syndrome of internal harassment of phlegm-heat	a pattern/syndrome arising when phlegm-heat disturbs the spirit and impedes the qi movement, marked by cough with yellow thick expectoration, dyspnea, fever, thirst, vexation, insomnia, dream-disturbed sleeping, reddened tongue with yellow slimy coating and rapid slippery pulse	痰熱が神を邪魔し、気機を妨げるときに生じる証で、黄色の粘稠な喀出物を伴う咳嗽、喘、発熱、口渇、心煩、失眠、夢に邪魔される睡眠、黄・膩苔を伴う紅舌および数・滑脈を特徴とする。
痰熱内閉證		2.6.32	pattern/syndrome of internal block of phlegm-heat	a pattern/syndrome arising when phlegm-heat in the interior obstructs and blocks the heart-spirit, manifested by impaired consciousness, delirium or mania associated with fullness, oppression and scorching pain in the chest, cough, dyspnea and expectoration of yellow thick sputum, fever, and thirst, or in some cases, sudden onset of unconsciousness with whizzing in the throat, reddened tongue with yellow slimy coating and slippery pulse	裏の痰熱が心神を閉塞・遮断するときに生じる証で、意識障害、胸部の充満、重圧感および灼痛に関連する譫妄または躁病、咳嗽、喘および黄色の粘稠な痰の喀出、発熱および口渇が発現する。喉がぜいぜい鳴る音を伴う意識消失の卒発、黄・膩苔を伴う紅舌および滑脈がみられる症例もある。
痰熱動風證		2.6.33	pattern/syndrome of phlegm- heat stirring wind	a pattern/syndrome marked by convulsions or vertigo associated with fullness and oppression in the chest, cough, dyspnea and expectoration of yellow thick sputum, fever, thirst, or nausea and vomiting, reddened tongue with yellow slimy coating and rapid slippery pulse	胸部の充満および胸悶に関連する抽搐またはめまい、咳嗽、喘および黄色の粘稠な痰の喀出、発熱、口渇、または悪心および嘔吐、黄・膩苔を伴う紅舌および数・滑脈を特徴とする証。
痰核留結證		2.6.34	pattern/syndrome of lingering phlegm nodule	a pattern/syndrome marked by lumps below the skin particularly of the neck, which are movable, round, firm and slippery under the finger, without redness, hotness or pain	とくに頚部の皮膚の下にある、可動性があり、丸くて固く、指で触ると逃げるが、発赤、熱っぽさ、疼痛はみられない瘤を特徴とする証。
血瘀風燥證		2.6.35	pattern/syndrome of blood stasis with wind-dryness	a pattern/syndrome arising when internally retained static blood gives rise to dryness and wind, and manifested by encrusted skin, desquamation and itching, associated with vertigo, numbness of the limb, purplish tongue or appearance of purple spots on the tongue, and fine choppy pulse	体内の瘀血が燥および風を引き起こすときに生じる証で、肌膚甲錯、落屑および掻痒に伴い、めまい、肢の麻木、紫舌または紫斑のある舌、不規則に変わる細脈が発現する。
血瘀水停證		2.6.36	pattern/syndrome of blood stasis with water retention	a pattern/syndrome marked by formation of mass in the abdomen with stabbing pain, enlarged and distended abdomen, inhibited urination, purplish tongue or appearance of purple spots on the tongue, and fine choppy pulse	刺痛を伴う腹部の腫瘤形成、腹部の拡張・膨満、小便不利、紫舌または紫斑のある舌、および不規則に変わる細脈を特徴とする証。
寒濕内阻證		2.6.37	pattern/syndrome of internal obstruction of cold-dampness	a pattern/syndrome marked by heaviness feeling of the head and body, joint pain with inhibited bending and stretching, absence of sweat, or edema of the face and limbs, loose stools, and dysuria; white and moistened tongue coating and slippery pulse	頭部および身体が重い感じ、関節痛のため屈伸できない、無汗または顔面および四肢の水腫、軟便、および排尿困難。潤・白苔および滑脈を特徴とする証。
寒凝血瘀證		2.6.38	pattern/syndrome of congealing cold with blood stasis	a pattern/syndrome arising when pathogenic cold obstructs qi movement and blood flow, manifested by pain aggravated by cold and alleviated by warmth, cold and cyanotic limbs, and delayed menstruation, painful periods, menstrual discharge of dark purple blood with clots, dark purple tongue with white coating, and sunken, slow and choppy pulse	寒邪により気機および血の流れが閉塞されるときに生じる証で、冷やすと悪化し温めると軽減する疼痛、四肢の冷えおよびチアノーゼ、および月経後期、有痛性の月経期、血餅の混じった黒紫色の月経、白苔を伴う黒紫舌、および不規則に変わる遅・沈脈が発現する。
血寒證		2.6.39	blood cold pattern/syndrome	a pattern/syndrome that arises when congealing cold and qi stagnation inhibit the movement of blood and give rise to blood stasis, commonly manifested by cold pain of the extremities with dark purple skin or cramps in the lesser abdomen that are relieved by warmth and exacerbated by cold, delayed menstruation and dark purple menstrual discharge with blood clots, white tongue coating and sunken, slow and choppy pulse	寒凝および気滞により血の動きが阻害され、血瘀がもたらされるときに生じる証で、黒紫色の皮膚を伴う四肢の冷痛または温めると緩和し冷やすと悪化する小腹急結、月経後期および血餅の混じった黒紫色の月経、白苔および不規則に変わる遅・沈脈がよく発現する。
濕熱毒蘊證		2.6.40	pattern/syndrome of retained dampness-heat toxin	a pattern/syndrome marked by redness, swelling, ulceration and exudation of the hand, foot, ear, nose, head, face or genital region, or by fever, jaundice, impaired consciousness, eruptions, reddened tongue and rapid soggy pulse	手、足、耳、鼻、頭、顔または陰部の発赤、腫脹、潰瘍および浸出液、または発熱、黄疸、意識障害、疹、紅舌および数・濡脈を特徴とする証。
濕熱下注證		2.6.41	pattern/syndrome of dampness-heat pouring downward	a pattern/syndrome marked by frequent and painful urination, or yellow fetid discharge from the vagina, or ulceration of the leg with purulent discharge	小便頻数および有痛性排尿、または悪臭のある黄帯、または膿性分泌液を伴う脚の潰瘍を特徴とする証。
瘟毒下注證		2.6.42	pattern/syndrome of pestilential toxin pouring downward	a pattern/syndrome marked by downward pouring of the pestilential toxin, e.g., painful swelling of the testis in mumps	痄腮時の精巣の疼痛を伴う腫脹など、有害な毒が下方に注がれることを特徴とする証。
風毒證		2.6.43	wind-toxin pattern/ syndrome	a pattern/syndrome due to wind-toxin attack on the skin and flesh, marked by sudden onset of edema, with numbness, itching and pain, or appearance of wheals, redness and swelling of face, eyes, nose and mouth	皮膚および肌肉に対する風毒の攻撃により生じる証で、麻木を伴う水腫の卒発、掻痒および疼痛、または膨疹、顔、目、鼻および口の発赤および腫脹を特徴とする。
風火熱毒證		2.6.44	wind-fire-heat toxin pattern/syndrome	a pattern/syndrome due to accumulation of wind-fire-heat toxin in the skin and flesh, marked by formation of boils, abscess or carbuncle accompanied by itching, numbness and scorching pain or suppuration and ulceration accompanied by high fever, crimson tongue with brownish yellow coating and rapid surging pulse	皮膚および肌肉における風・火・熱毒の蓄積により生じる証で、掻痒を伴うせつ、癰または有頭疽の形成、麻木および灼痛または壮熱を伴う化膿および潰瘍、黄褐色の舌苔を伴う絳舌および数・洪脈を特徴とする。
火毒證		2.6.45	fire toxin pattern/ syndrome	a pattern/syndrome caused by toxin derived from exuberant heat-fire retaining in the skin and subcutaneous tissue, marked by local redness, swelling and burning pain, followed by abscess formation and accompanied by fever, thirst, reddened tongue with yellow coating, and rapid pulse	皮膚および皮下組織に停滞した盛んな熱火に由来する毒により生じる証で、局所の発赤、腫脹および灼熱痛と、それに続く膿瘍形成および併発する発熱、口渇、黄苔を伴う紅舌および数脈を特徴とする。
火毒内陷證		2.6.46	pattern/syndrome of inward invasion of fire toxin	a syndrome arising when exuberant fire-heat toxin penetrates into the internal organs, usually manifested by high fever with thirst, delirium, constipation, dark-colored urine, crimson tongue with yellow coating and rapid sunken pulse	盛んな火熱毒が内臓に浸透するときに生じる症候群で、通常、口渇を伴う壮熱、譫妄、便秘、暗黒色の尿、黄苔を伴う絳舌および数・沈脈が発現する。
陰毒證		2.6.47	yin toxin pattern/syndrome	a pattern/syndrome arising when toxin derived from yin-cold accumulates, usually manifested by local diffuse swelling with cold pain that is relieved by warmth and failure to suppurate and rupture, or contains thin foul-smelling pus, accompanied by aversion to cold and cold limbs, white tongue coating and sunken pulse	陰寒に由来する毒が蓄積するときに生じる証で、通常、温めると軽減し化膿・破裂しにくい、または希薄な悪臭のある膿を含む局所のびまん性の腫脹とともに冷痛が発現し、悪寒および四肢の冷え、白苔および沈脈が付随する。

検索表示（原表示の複数語を分離）	IST原表示	Code	Term	Definition/Description	定義/記述
蛇毒內攻證		2.6.48	pattern/syndrome of inward attack of snake venom	a pattern/syndrome arising after snake-bite when the snake venom invades the internal organs, marked by headache and dizziness, oppression in the chest, dyspnea, cold sweats and cold limbs, or loss of consciousness	蛇に咬まれた後、蛇毒が内臓に侵入するときに生じる証で、頭痛およびめまい、胸悶、喘、冷汗および四肢の冷え、または神昏を特徴とする。
石阻證		2.6.49	calculus obstruction pattern/syndrome	a pattern/syndrome caused by calculus obstruction, marked by distending pain or colicky pain in the right hypochondrium or in the lumbar region referring to the lesser abdomen	結石の閉塞により生じる証で、下腹に放散する右下肋部または腰部の脹痛や疝痛を特徴とする。
風寒證		2.6.50	wind-cold pattern/ syndrome	a pattern/syndrome caused by externally contracted wind and cold, and manifested by pronounced aversion to cold with mild fever, headache, generalized pain, absence of sweating, stuffy and runny nose with watery discharge, thin white and moistened tongue coating, and floating pulse	体外で風寒を感受することにより生じる証で、微熱を伴う著しい悪寒、頭痛、身痛、無汗、鼻づまりおよび水のような鼻汁の分泌、薄・白・潤苔、および浮脈が発現する。
風熱證		2.6.51	wind-heat pattern/ syndrome	a pattern/syndrome caused by externally contracted wind and heat, and manifested by pronounced fever and mild aversion to cold, cough, thirst, reddened tongue margins and tip, slightly yellow coating and rapid floating pulse	体外で風熱を感受することにより生じる証で、著しい発熱および軽度の悪寒、咳嗽、口渇、紅色の舌辺・舌尖、淡黄苔および数・浮脈が発現する。
風火證		2.6.52	wind-fire pattern/ syndrome	a pattern/syndrome caused by externally contracted wind and fire in combination	体外で風・火の両方を感受することにより生じる証。
風濕證		2.6.53	wind-dampness pattern/syndrome	a pattern/syndrome caused by externally contracted wind and dampness in combination, manifested by generalized pain, heaviness feeling of the body, joint pain with inhibited articular movement	体外で風・湿の両方を感受することにより生じる証で、身痛、身体が重い感じ、関節を動かしにくくなる関節痛が発現する。
風燥證		2.6.54	wind-dryness pattern/ syndrome	a pattern/syndrome caused by externally contracted wind and dryness in combination, and manifested by headache, fever, aversion to wind, absence of sweating, dry nose, lips, throat and skin, dry cough, thin and dry tongue coating	体外で風・燥の両方を感受することにより生じる証で、頭痛、発熱、悪風、無汗、鼻、唇、喉および皮膚の乾燥、乾咳、薄・燥苔が発現する。
熱毒證		2.6.55	heat toxin pattern/ syndrome	a pattern/syndrome arising when accumulated pathogenic fire-heat is transformed into toxin and manifesting in boils, sores, and eruptive diseases	蓄積した火熱邪が毒に転換するときに生じ、せつ、瘡および発疹を伴う疾病において発現する証。
濕毒證		2.6.56	dampness toxin pattern/syndrome	a pattern/syndrome arising when accumulated dampness turns into toxin and marked by a lingering course difficult to cure, and presence of turbid secretions	蓄積した湿が毒に転換するときに生じる証で、経過が長引き治癒しにくく、濁った分泌物の存在を特徴とする。
寒濕證		2.6.57	cold-dampness pattern/syndrome	(1) a pattern/syndrome that arises when the movement of qi and blood is impeded by cold and dampness in combination, and is marked by joint, muscle and bone pains; (2) a pattern/syndrome that arises when dampness harasses the spleen and stomach and cold causes damage to spleen yang, or water-fluid retained in a case of spleen-kidney yang deficiency, and is marked by aversion to cold, cold limbs, abdominal distension, diarrhea or edema	（1）気および血の動きが寒・湿の両方によって妨げられるときに生じる証で、関節、筋肉および骨の疼痛を特徴とする、（2）湿が脾および胃を苦しめ、寒が脾陽に損傷（脾腎陽虚例では水津の停滞）をもたらすときに生じる証で、悪寒、四肢の冷え、腹部膨満、泄瀉または水腫を特徴とする。
濕熱證		2.6.58	dampness-heat pattern/syndrome	a pattern/syndrome caused by a combination of dampness and heat, either of external or of internal origin, with different manifestations according to location, e.g., jaundice when dampness-heat accumulates in the liver and gallbladder, leukorrhea when dampness-heat pours down, and diarrhea for dampness-heat in the intestines	体外または体内のいずれかに由来する湿・熱の両方により生じる証で、部位により異なる症候が発現する。たとえば、湿熱が肝および胆に蓄積すると黄疸、湿熱が吹き出ると帯下、腸に存在すると泄瀉を引き起こす。
陰暑證		2.6.59	yin summerheat pattern/syndrome	a pattern/syndrome that results from exposure to wind or drafts or to excessive consumption of cold drinks in the hot summer, and is manifested by fever, headache, aversion to cold, absence of sweating, and generalized pain; and in some cases, vomiting, diarrhea and abdominal pain	風やすきま風に曝露したこと、または暑い夏に冷たい飲料水を摂取しすぎたことにより生じる証で、発熱、頭痛、悪寒、無汗、身痛が発現する。嘔吐、泄瀉および腹痛が生じる場合もある。
痰濕證		2.6.60	phlegm-dampness pattern/syndrome	a pattern/syndrome that arises when dampness gathers to form phlegm, with different manifestations according to location: cough with profuse whitish expectoration and oppression in chest if phlegm obstructs the lung, and epigastric stuffiness, reduced food intake, and greasy taste in the mouth if phlegm accumulates in the spleen. The general signs of phlegm-dampness are white slimy tongue coating and slippery pulse.	湿が集まって痰を形成するときに生じる証で、部位により異なる症侯が発現する。たとえば、痰が肺を閉塞すると大量の白色の喀出物を伴う咳嗽および胸部重圧感、痰が脾に蓄積すると心窩部が詰まった感じ、摂食量減少、口の中が油っぽい感じを引き起こす。痰湿の一般的な徴候は白・膩苔および滑脈である。
氣血辨證		**2.7.0**	**Qi-Blood Pattern Identification/ Syndrome Differentiation**		
氣血辨證		2.7.1	qi-blood pattern identification/ syndrome differentiation	categorization of patterns/syndromes according to the state of qi and blood	気および血の状態による証の分類。
氣虛證		2.7.2	qi deficiency pattern/ syndrome	a pattern/syndrome of deficiency of genuine qi with diminished function of internal organs, marked by shortness of breath, lassitude, listlessness, spontaneous sweating, pale tongue and weak pulse	内臓機能の低下を伴う真気の虚の証で、短気、倦怠感、無気力、自汗、淡白舌および弱脈を特徴とする。
氣陷證		2.7.3	qi sinking pattern/ syndrome	a pattern/syndrome resulting from failure in its lifting or holding function, marked by dizziness, blurred vision, shortage of qi, lassitude, prolapse of the anus, prolapse of the uterus or visceroptosis, pale tongue with white coating and weak pulse	機能の亢進や維持ができなくなることにより生じる証で、めまい、目昏、少気、倦怠感、肛門脱、子宮脱または内臓下垂、白苔を伴う淡白舌および弱脈を特徴とする。
氣滯證		2.7.4	qi stagnation pattern/ syndrome	a pattern/syndrome resulting from stagnation of qi, marked by intermittent thoracic, hypochondriac, epigastric and abdominal distention or pain, often ameliorated by sighing or belching	気の停滞により生じる証で、しばしば太息または噯気により軽減する、間欠的な胸部、下肋部、心窩部および腹部の膨満感または疼痛を特徴とする。
氣逆證		2.7.5	qi counterflow pattern/syndrome	a pattern/syndrome arising when qi moves abnormally upward, manifested by cough and dyspnea, or nausea, vomiting, hiccup, belching or even hematemesis, or feeling of gas ascending from the lesser abdomen to the chest or throat with distension and oppression, headache and vertigo	気が異常に上方に動くときに生じる証で、咳嗽および喘、または悪心、嘔吐、呃逆、噯気または吐血、または膨満感および重圧感を伴い、ガスが下腹から胸または喉に上がってくる感じ、頭痛およびめまいが発現する。

検索表示（原表示の複数語を分離）	IST原表示	Code	Term	Definition/Description	定義/記述
氣閉證		2.7.6	qi block pattern/ syndrome	a pattern/syndrome marked by sudden loss of consciousness with restlessness, trismus and contracture of limbs, or by colicky pain in the chest and abdomen, or by sudden onset of panting with cyanosis, or by fecal retention and urinary block	落ち着きのなさを伴う突然の神昏、開口障害および四肢拘急、または胸部および腹部の疝痛、またはチアノーゼを伴う喘促の卒発、または便閉および尿閉を特徴とする証。
氣脫證		2.7.7	qi collapse pattern/ syndrome	a pattern/syndrome marked by sudden appearance of profuse sweating, somber pale complexion, cyanotic lips, cold extremities, feeble breathing, even fainting or loss of consciousness with incontinence of urine, pale tongue and hardly perceptible pulse	突然の多汗、くすんで蒼白な顔色、唇のチアノーゼ、四肢の冷え、弱々しい呼吸、小便失禁を伴う昏厥または神昏、淡白舌およびほとんど触れない脈を特徴とする証。
氣機失調證		2.7.8	disordered qi movement pattern/ syndrome	a general term for a group of patterns/syndromes attributed to disorders of qi movement including qi stagnation, qi counterflow, qi fall, qi block and qi collapse	気滞、気逆、気焔、気閉および気脱など、気機の障害に起因する一群の証の一般用語。
氣機不利證		2.7.9	inhibited qi movement pattern/ syndrome	a pattern/syndrome that arises when impeded, obstructed or stagnant qi flow impairs the functions of viscera and meridians/channels and is marked by sensation of oppression, distension, and pain associated with frequent sighing, depressed mood and string-like pulse	妨げられ閉塞した、またはうっ滞した気の流れが臓および経絡の機能を障害することにより生じる証で、重圧感、膨満感および疼痛とともに頻繁な太息、抑うつ気分および弦脈を特徴とする。
氣機鬱滯證		2.7.10	stagnant qi movement pattern/syndrome	a pattern/syndrome caused by stagnation of qi movement, marked by feeling of oppression, distension and pain with a tendency to sighing, emotional depression and string-like pulse	気機のうっ滞により生じる証で、太息傾向を伴う重圧感、膨満感および疼痛、感情の落ち込みおよび弦脈を特徴とする。
氣鬱證		2.7.11	qi depression pattern/ syndrome	a pattern/syndrome marked by feeling of distension in the chest, pain in the hypochondriac region, irritability, irascibility, anorexia and menstrual disorders in women, the same as the qi stagnation pattern/syndrome	胸部膨満感、下肋部の疼痛、いらいら、怒りっぽい、厭食および月経障害（女性）を特徴とする証。気滞証と同義。
氣鬱化火證		2.7.12	pattern/syndrome of depressed qi transforming into fire	a pattern/syndrome marked by emotional depression, irritability, irascibility, distention and burning pain in the chest, and reddened tongue with yellow coating, the same as the pattern/syndrome of stagnated qi transforming into fire	感情の落ち込み、いらいら、怒りっぽい、胸部の膨満感および灼熱痛、および黄苔を伴う紅舌を特徴とする証。pattern/syndrome of stagnated qi transforming into fireと同義。
寒凝氣滯證		2.7.13	pattern/syndrome of congealing cold with qi stagnation	a pattern/syndrome that arises when pathogenic cold hampers the qi movement and qi transformation and is characterized by various pains, such as general pain, headache, painful and rigid neck, back pain and lumbago, epigastric and abdominal pain with cold feeling, pain of extremities and joints	寒邪が気機および気化を妨げるときに生じる証で、身痛、頭痛、頚部の疼痛・硬直、背痛および腰痛、冷感を伴う心窩部痛および腹痛、四肢および関節の疼痛など、さまざまな疼痛を特徴とする。
中氣下陷證		2.7.14	sunken middle qi pattern/syndrome	a pattern/syndrome marked by bearing-down sensation in the epigastrium and abdomen, protracted diarrhea, even prolapse of rectum or visceroptosis	心窩部および腹部が下垂する感じ、長引く泄瀉、直腸脱または内臓下垂を特徴とする証。
氣虛不攝證		2.7.15	pattern/syndrome of qi deficiency with failure to constrain	a pattern/syndrome arising when failure of insufficient qi in constraint leads to loss of liquid substances, marked by seminal emission, incontinence of urine, spontaneous sweating, hemorrhages, lusterless complexion, lassitude, lack of strength, pale and plump tongue, and weak pulse	気の不足により水津を体内に閉じこめることができず、結果として水津が減少するときに生じる証で、遺精、小便失禁、自汗、出血、つやのない顔色、倦怠感、乏力、膨らんだ淡白舌、および弱脈を特徴とする。
氣虛發熱證		2.7.16	qi deficiency fever pattern/syndrome	a pattern/syndrome marked by a low persistent fever exaggerated by physical exertion, associated with fatigue, lack of strength, shortness of breath, pale tongue and weak pulse	持続性の低熱が激しい身体活動により悪化することを特徴とする証で、疲労、乏力、短気、淡白舌および弱脈を伴う。
氣虛濕阻證		2.7.17	pattern/syndrome of qi deficiency with dampness obstruction	a pattern/syndrome marked by listlessness, lassitude, reduced food intake, shortness of breath, heaviness feeling of the head and body, abdominal distension, diarrhea and soggy weak pulse	無気力、倦怠感、摂食量減少、短気、頭部および身体が重い感じ、腹部膨満、泄瀉および濡・弱脈を特徴とする証。
氣虛水停證		2.7.18	pattern/syndrome of qi deficiency with water retention	a pattern/syndrome marked by edema of limbs, inhibited urine, heaviness feeling of the head and body, distension, pain and a feeling of pressure in the chest, epigastrium and abdomen, and pale tongue with white slippery coating	四肢の水腫、小便不利、頭部および身体が重い感じ、胸部、心窩部および腹部の膨満感、疼痛、圧迫感、および白・滑苔を伴う淡白舌を特徴とする証。
氣虛外感證		2.7.19	pattern/syndrome of qi deficiency with external contraction	a pattern/syndrome marked by aversion to cold, fever, spontaneous sweating, headache, stuffy nose, feeble voice, lassitude, lack of strength and shortness of breath	悪寒、発熱、自汗、頭痛、鼻づまり、弱々しい声、倦怠感、乏力および短気を特徴とする証。
氣陰兩虛證;<br>氣陰虧虛證		2.7.20	pattern/syndrome of dual deficiency of qi and yin	a pattern/syndrome marked by listlessness, lack of strength, shortness of breath, reluctance to speak, dry throat and mouth, vexing thirst, flushed cheeks in the afternoon, short voidings of small amount of urine, constipation, emaciation, scanty dry tongue coating and vacuous pulse	無気力、乏力、短気、話すのが億劫、喉および口の乾燥、わずらわしい口渇、午後の頬部潮紅、短時間の少量の尿の排出、便秘、るいそう、少量の燥苔および虚脈を特徴とする証。
血虛證		2.7.21	blood deficiency pattern/syndrome	a pattern/syndrome marked by pale or sallow complexion, pale lips and nails, dizziness, dimmed vision, palpitations, numbness of extremities and fine pulse	面色蒼白または面色委黄、青白い唇および爪、めまい、目暗、心悸、四肢の麻木および細脈を特徴とする証。
血脫證		2.7.22	blood collapse pattern/syndrome	a critical pattern/syndrome occurring in cases of acute massive bleeding, marked by pallor, dizziness, palpitations, faint and short breathing, cold extremities and even mental confusion, pale tongue, hollow pulse or hardly perceptible pulse	急性の大量出血例に生じる危篤の証で、蒼白、めまい、心悸、弱々しい短い呼吸、四肢の冷えおよび昏蒙、淡白舌、芤脈またはほとんど触れない脈を特徴とする。
血瘀證		2.7.23	blood stasis pattern/ syndrome	a pattern/syndrome marked by formation of visible painful and tender purple mass, or abdominal mass with stabbing pain and tenderness, or bleeding of dark purple blood with clots, dark purple tongue, and fine choppy or irregular pulse	肉眼で認められる有痛性の柔らかい紫色の腫瘤形成、または刺痛および圧痛を伴う腹部腫瘤、または血餅の混じった黒紫色の出血、黒紫舌、および不規則に変わる細脈または不整脈を特徴とする証。
蓄血證		2.7.24	blood amassment pattern/syndrome	a pattern/syndrome caused by stagnated blood accumulated in a meridian/channel or an organ, e.g., in the uterus, manifested by distention and pain in the lower abdomen, chills and fever, delirium or other mental disorders at night, or in the middle energizer, manifested by pain, tenderness to touch over the epigastrium	うっ滞した血が経絡または臓器に蓄積することにより生じる証で、たとえば胞（子宮）では、下腹の膨満感および疼痛、寒気および発熱、夜間の譫妄またはその他の精神障害、中焦では、心窩部の疼痛、圧痛が発現し、触れられるのを嫌がる。

検索表示（原表示の複数語を分離）	IST原表示	Code	Term	Definition/Description	定義/記述
血熱證		2.7.25	blood heat pattern/syndrome	a pattern/syndrome that occurs when exuberant pathogenic heat enters the blood aspect and is manifested by fever, nose-bleed, vomiting of blood, expectoration of blood, bloody stool, skin eruptions, or advanced periods with profuse bright-red menstrual discharge, vexation, or even delirium and convulsions, deep crimson tongue and rapid string-like pulse	盛んな 邪熱が血分に進入するときに生じる証で、発熱、鼻血、血の吐出や喀出 、便血、皮疹、または大量の鮮紅色の月経を伴う月経先期、心煩、または譫妄および抽搐、深い絳舌および数･弦脈が発現する。
氣血兩虛證		2.7.26	pattern/syndrome of dual deficiency of qi and blood	a pattern/syndrome marked by listlessness, lack of strength, shortness of breath, pale or sallow complexion, dizziness, dimmed vision, pale lips and nails, palpitation, insomnia, pale tongue and weak pulse	無気力、乏力、短気、面色蒼白または面色委黄、めまい、目暗、蒼白な唇および爪、心悸、失眠、淡白舌および弱脈を特徴とする証。
氣血失調證		2.7.27	qi-blood disharmony pattern/syndrome	a pattern/syndrome resulting from disharmony of qi and blood with failure in mutual nourishing and complementing, and usually associated with persistent pain, reverse flow of qi, menstrual irregularities and chronic bleeding	気血の調和が乱れ、互いに滋養し合い、補い合うことができなくなることにより生じる証で、通常、持続痛、気の逆流、月経不調および慢性出血を伴う。
氣虛血瘀證		2.7.28	pattern/syndrome of qi deficiency with blood stasis	a pattern/syndrome of blood stasis resulting from qi deficiency, marked by somber pale complexion, lack of strength, shortness of breath, local stabbing pain, purplish tongue or purple spots on the tongue and sunken choppy pulse	気虚により生じる血瘀の証で、くすんだ蒼白な顔色、乏力、短気、局所刺痛、紫舌または紫斑のある舌および不規則に変わる沈脈を特徴とする。
氣滯血瘀證		2.7.29	pattern/syndrome of qi stagnation and blood stasis	a pattern/syndrome marked by moving or stabbing pain in the thoracic, hypochondriac, epigastric or abdominal region with or without mass formation, purple tongue or purple-spotted tongue, and string-like choppy pulse	腫瘤形成の有無を問わない胸部、下肋部、心窩部または腹部の遊走痛または刺痛、紫舌または紫斑のある舌および不規則に変わる弦脈を特徴とする証。
氣隨血脫證		2.7.30	pattern/syndrome of qi collapse following bleeding	a pattern/syndrome marked by pale complexion, reverse cold of limbs, profuse sweating, feeble breathing, or even respiratory arrest, and hardly perceptible pulse or vacuous rootless large pulse	面色蒼白、手足厥冷、多汗、弱々しい呼吸、または呼吸停止、およびほとんど触れない脈または根がない虚・大脈を特徴とする証。
氣不攝血證		2.7.31	pattern/syndrome of qi failing to control the blood	a pattern/syndrome marked by hematochezia, bleeding through the pores, gum bleeding, uterine bleeding or excessive menstrual discharge, listlessness, lack of strength, shortness of breath, laziness to speak, lusterless complexion, pale tongue and weak pulse	便血、開口部からの出血、歯衄、子宮出血または過剰な月経、無気力、乏力、短気、話すのが億劫、つやのない顔色、淡白舌および弱脈を特徴とする証。
血虛挾瘀證		2.7.32	pattern/syndrome of blood deficiency complicated by stasis	a pattern/syndrome marked by sallow or pale complexion, dizziness, blurred vision, palpitations, dream-disturbed sleep, stabbing pain fixed in location, scanty menstrual discharge of dark purple blood with clots, dysmenorrhea or amenorrhea, purple tongue or purple spots on the tongue, and fine choppy pulse	面色委黄または面色蒼白、めまい、目昏、心悸、夢に邪魔される睡眠、部位が固定された刺痛、血餅の混じった黒紫色の少量の月経、月経困難症または無月経、紫舌または紫斑のある舌、および不規則に変わる細脈を特徴とする証。
血虛寒凝證		2.7.33	pattern/syndrome of blood deficiency and congealing cold	a pattern/syndrome marked by purplish complexion, dizziness, blurred vision, dark purple lips and tongue, cold hands and feet, and localized cold, pain and numbness; in women, late periods with scanty menstrual discharge of dark blood or clots, painful periods or amenorrhea	紫がかった顔色、めまい、目昏、黒紫色の唇および舌、手足の冷え、および局所の冷え、疼痛および麻木を特徴とする証。女性では、月経後期、少量の暗黒色の血または血餅の分泌、有痛性の月経期または閉経が認められる。
血虛風燥證		2.7.34	pattern/syndrome of blood deficiency and wind-dryness	a pattern/syndrome marked by dry, rough, itchy, shriveled skin with rhagades, withering and loss of hair, numbness of body surface, contraction of hands and feet, lusterless complexion, pale nails, dizziness and blurred vision, pale tongue and fine pulse	あかぎれ、ひび割れおよび脱毛を伴う皮膚の乾燥、荒れ、かゆみ、しわ、体表の麻木、手足の筋収縮、つやのない顔、蒼白の爪、めまいおよび目昏、淡白舌および細脈を特徴とする証。
血虛生風證		2.7.35	pattern/syndrome of blood deficiency engendering wind	a liver wind pattern/syndrome attributed to blood deficiency that deprives the sinews of nourishment, and marked by numbness, tremor, contraction of limbs, itching, vertigo, lusterless nails, pale tongue and fine weak pulse	血の虚により筋の滋養が枯渇することにより生じる肝風証で、麻木、顫震、四肢の筋収縮、掻痒、めまい、つやのない爪、淡白舌および細・弱脈を特徴とする。
津液辨證		**2.8.0**	**Fluid-Humor Pattern Identification/ Syndrome Differentiation**		
津液辨證		2.8.1	fluid-humor pattern identification/ syndrome differentiation	categorization of patterns/syndromes according to the condition of body fluids	津液の状態による証の分類。
痰證		2.8.2	phlegm pattern/ syndrome	a general term for a group of patterns/syndromes marked by cough, dyspnea with profuse expectoration, or by nausea, vomiting and dizziness, or by formation of lumps or nodes	咳嗽、大量の喀出物を伴う喘、または悪心、嘔吐およびめまい、またはしこりや結節の形成を特徴とする一群の証の一般用語。
飮證		2.8.3	fluid retention pattern/syndrome	a pattern/syndrome marked by dizziness, thoracic and epigastric stuffiness sensation, vomiting of clear fluid, slippery tongue coating and string-like pulse	めまい、胸部および心窩部が詰まった感じ、透明な津の嘔吐、滑苔および弦脈を特徴とする証。
水停證		2.8.4	water retention pattern/syndrome	a pattern/syndrome marked by edema, oliguria, or accompanied by ascites, pale plump tongue with white slippery coating and soggy relaxed pulse	水腫、乏尿、またはこれに付随する腹水、白色の滑苔を伴う膨らんだ淡白舌および濡・緩脈を特徴とする証。
液脫證		2.8.5	humor collapse pattern/syndrome	a severe case of fluid deficiency pattern/syndrome, marked by parched or cracked lips, withered skin, sunken eyes, tinnitus, oliguria and dry fecal binding, reddened tongue with no moisture and fine weak pulse	津の虚を示す重症例の証で、乾いたまたはひび割れた唇、皮膚のひび割れ、落ちくぼんだ目、耳鳴、乏尿および乾燥して固まった便、潤いのない紅舌および細・弱脈を特徴とする。
津液虧虛證	津液虧虛證; 津液虧損證	2.8.6	fluid-humor deficiency pattern/syndrome	a pattern/syndrome marked by dry mouth and throat, parched or cracked lips, thirst with desire for drink, oliguria, constipation, reddened tongue lacking moisture, and rapid fine weak pulse	口および喉の乾燥、乾いたまたはひび割れた唇、口渇および冷たい飲料水の渇望、乏尿、便秘、潤いのない紅舌、および数・細・弱脈を特徴とする証。
津液虧損證	津液虧虛證; 津液虧損證	2.8.6	fluid-humor deficiency pattern/syndrome	a pattern/syndrome marked by dry mouth and throat, parched or cracked lips, thirst with desire for drink, oliguria, constipation, reddened tongue lacking moisture, and rapid fine weak pulse	口および喉の乾燥、乾いたまたはひび割れた唇、口渇および冷たい飲料水の渇望、乏尿、便秘、潤いのない紅舌、および速・細・弱脈を特徴とする証。

検索表示（原表示の複数語を分離）	IST原表示	Code	Term	Definition/Description	定義/記述
津氣虧虛證		2.8.7	fluid-qi deficiency pattern/syndrome	a pattern/syndrome due to deficiency of both fluid and qi, manifested by listlessness, shortness of breath, vexing thirst, dry skin, reddened tongue with dry coating and fine weak pulse	津および気の両方の虚により生じる証で、無気力、短気、わずらわしい口渇、皮膚の乾燥、燥苔を伴う紅舌および細・弱脈が発現する。
氣滯水停證		2.8.8	pattern/syndrome of qi stagnation with water retention	a pattern/syndrome marked by edema of limbs, oliguria, heaviness feeling of the head and body, distension, oppression and scurry pain in the chest, epigastrium and abdomen, pale tongue with white slippery coating and string-like relaxed pulse	四肢の水腫、乏尿、頭部および身体が重い感じ、胸部、心窩部、腹部の膨満感、重圧感および竄痛、白・滑苔を伴う淡白舌および弦・緩脈を特徴とする証。
飲停胸脅證		2.8.9	pattern/syndrome of fluid retention in the chest and hypochondrium	a pattern/syndrome characterized by pain in the chest and hypochondrium, which is aggravated by twisting movements or breathing, and particularly by coughing, and associated with thoracic distension, a feeling of pressure in the chest and shortness of breath, white and slippery tongue coating, and string-like pulse	体をよじる動作や呼吸、またとくに咳嗽により悪化する胸部および下肋部の疼痛を特徴とし、胸部膨満感、胸部圧迫感および短気、白・滑苔および弦脈を伴う証。
風水相搏證		2.8.10	pattern/syndrome of mutual contention of wind and water	a pattern/syndrome that arises when pathogenic wind attacking the lung causes dysfunction in diffusion and depurative downbearing and leads to abnormal accumulation of water under the skin, and is manifested by acute onset of edema of the head and face, and then generalized, associated with aversion to cold, fever, absence of sweating, reduced amount of urine, thin white tongue coating and floating pulse	風邪が肺を攻撃し、それにより宣発・粛降の機能障害をきたし、皮下に水が異常に蓄積するときに生じる証で、頭部および顔面に水腫が急性に発現した後、全身に広がり、悪寒を伴い、発熱、無汗、尿量減少、薄・白苔および浮脈が発現する。
臟腑辨證		**2.9.0**	**Visceral Pattern Identification/ Syndrome Differentiation**		
臟腑辨證		2.9.1	visceral pattern identification/ syndrome differentiation	categorization of patterns/syndromes according to the pathological changes of viscera and bowels	臓腑の病的変化による証の分類。
心病辨證		2.9.2	Heart Diseases Pattern Identification/ Syndrome Differentiation	visceral pattern identification/syndrome differentiation dealing with diseases of the heart	心の疾病を取り扱う臓の弁証論治。
心氣虛證	心氣虛證; 心氣不足證; 心氣虧虛證	2.9.3	heart qi deficiency pattern/syndrome	a pattern/syndrome marked by palpitations, shortness of breath, listlessness, spontaneous sweating, pallor, pale tongue, and weak or irregular pulse	心悸、短気、無気力、自汗、蒼白、淡白舌および弱脈または不整脈を特徴とする証。
心氣不足證	心氣虛證; 心氣不足證; 心氣虧虛證	2.9.3	heart qi deficiency pattern/syndrome	a pattern/syndrome marked by palpitations, shortness of breath, listlessness, spontaneous sweating, pallor, pale tongue, and weak or irregular pulse	心悸、短気、無気力、自汗、蒼白、淡白舌および弱脈または不整脈を特徴とする証。
心氣虧虛證	心氣虛證; 心氣不足證; 心氣虧虛證	2.9.3	heart qi deficiency pattern/syndrome	a pattern/syndrome marked by palpitations, shortness of breath, listlessness, spontaneous sweating, pallor, pale tongue, and weak or irregular pulse	心悸、短気、無気力、自汗、蒼白、淡白舌および弱脈または不整脈を特徴とする証。
心血虛證	心血虛證; 心血不足證; 心血虧虛證	2.9.4	heart blood deficiency pattern/syndrome	a pattern/syndrome resulting from deficiency of blood to nourish the heart spirit, manifested by palpitations, dizziness, dream-disturbed sleep, forgetfulness, pale or sallow complexion, pale lips and tongue, and fine pulse	心神を滋養するための血の虚により生じる証で、心悸、めまい、夢に邪魔される睡眠、健忘症、面色蒼白または面色萎黄、淡白な色の唇および舌、および細脈が発現する。
心血不足證	心血虛證; 心血不足證; 心血虧虛證	2.9.4	heart blood deficiency pattern/syndrome	a pattern/syndrome resulting from deficiency of blood to nourish the heart spirit, manifested by palpitations, dizziness, dream-disturbed sleep, forgetfulness, pale or sallow complexion, pale lips and tongue, and fine pulse	心神を滋養するための血の虚により生じる証で、心悸、めまい、夢に邪魔される睡眠、健忘症、面色蒼白または面色萎黄、淡白な色の唇および舌、および細脈が発現する。
心血虧虛證	心血虛證; 心血不足證; 心血虧虛證	2.9.4	heart blood deficiency pattern/syndrome	a pattern/syndrome resulting from deficiency of blood to nourish the heart spirit, manifested by palpitations, dizziness, dream-disturbed sleep, forgetfulness, pale or sallow complexion, pale lips and tongue, and fine pulse	心神を滋養するための血の虚により生じる証で、心悸、めまい、夢に邪魔される睡眠、健忘症、面色蒼白または面色萎黄、淡白な色の唇および舌、および細脈が発現する。
心氣血兩虛證		2.9.5	pattern/syndrome of dual deficiency of heart qi and blood	a pattern/syndrome arising when deficiency of both qi and blood deprives the heart and spirit of nourishment, usually manifested by palpitations, shortness of breath, listlessness, fatigue, dizziness, forgetfulness, dream-disturbed sleep, pale complexion and tongue, and fine weak pulse	気および血の両方の虚のために心神の滋養が枯渇することにより生じる証で、通常、心悸、短気、無気力、疲労、めまい、健忘症、夢に邪魔される睡眠、面色蒼白・淡白舌および細・弱脈が発現する。
心虛膽怯證		2.9.6	pattern/syndrome of heart deficiency with timidity	a pattern/syndrome marked by palpitations, insomnia, timidity and susceptibility to fright, dizziness, a feeling of pressure in the chest, pale tongue, weak pulse or rapid stirred pulse	心悸、失眠、臆病で驚きやすい、めまい、胸部圧迫感、淡白舌、弱脈または数・動脈を特徴とする証。

検索表示（原表示の複数語を分離）	IST原表示	Code	Term	Definition/Description	定義/記述
心陰虛證	心陰虛證; 心陰不足證; 心陰虧虛證	2.9.7	heart yin deficiency pattern/syndrome	a pattern/syndrome resulting from deficiency of yin fluid to nourish the heart spirit, manifested by mental irritability, palpitation, insomnia, low fever, night sweating, malar flush, thirst and rapid fine pulse	心神を滋養するための陰津の虚により生じる証で、精神的ないらいら、心悸、失眠、低熱、盗汗、頬部潮紅、口渇および数・細脈が発現する。
心陰不足證	心陰虛證; 心陰不足證; 心陰虧虛證	2.9.7	heart yin deficiency pattern/syndrome	a pattern/syndrome resulting from deficiency of yin fluid to nourish the heart spirit, manifested by mental irritability, palpitation, insomnia, low fever, night sweating, malar flush, thirst and rapid fine pulse	心神を滋養するための陰津の虚により生じる証で、精神的ないらいら、心悸、失眠、低熱、盗汗、頬部潮紅、口渇および数・細脈が発現する。
心陰虧虛證	心陰虛證; 心陰不足證; 心陰虧虛證	2.9.7	heart yin deficiency pattern/syndrome	a pattern/syndrome resulting from deficiency of yin fluid to nourish the heart spirit, manifested by mental irritability, palpitation, insomnia, low fever, night sweating, malar flush, thirst and rapid fine pulse	心神を滋養するための陰津の虚により生じる証で、精神的ないらいら、心悸、失眠、低熱、盗汗、頬部潮紅、口渇および数・細脈が発現する。
心陽虛證	心陽虛證; 心陽不足證; 心陽虧虛證	2.9.8	heart yang deficiency pattern/syndrome	a pattern/syndrome resulting from deficiency of yang qi to warm and activate the heart, usually manifested by palpitation, dyspnea, a feeling of pressure in the chest, aversion to cold with cold limbs, bright pale complexion, dark lips and tongue with white coating, weak or irregular pulse	心を温め、活性化するための陽気の虚により生じる証で、通常、心悸、喘、胸部圧迫感、四肢の冷えを伴う悪寒、面色㿠白、暗黒色の唇および白苔を伴う舌、弱脈または不整脈が発現する。
心陽不足證	心陽虛證; 心陽不足證; 心陽虧虛證	2.9.8	heart yang deficiency pattern/syndrome	a pattern/syndrome resulting from deficiency of yang qi to warm and activate the heart, usually manifested by palpitation, dyspnea, a feeling of pressure in the chest, aversion to cold with cold limbs, bright pale complexion, dark lips and tongue with white coating, weak or irregular pulse	心を温め、活性化するための陽気の虚により生じる証で、通常、心悸、喘、胸部圧迫感、四肢の冷えを伴う悪寒、面色㿠白、暗黒色の唇および白苔を伴う舌、弱脈または不整脈が発現する。
心陽虧虛證	心陽虛證; 心陽不足證; 心陽虧虛證	2.9.8	heart yang deficiency pattern/syndrome	a pattern/syndrome resulting from deficiency of yang qi to warm and activate the heart, usually manifested by palpitation, dyspnea, a feeling of pressure in the chest, aversion to cold with cold limbs, bright pale complexion, dark lips and tongue with white coating, weak or irregular pulse	心を温め、活性化するための陽気の虚により生じる証で、通常、心悸、喘、胸部圧迫感、四肢の冷えを伴う悪寒、面色㿠白、暗黒色の唇および白苔を伴う舌、弱脈または不整脈が発現する。
心陽虛脫證		2.9.9	heart yang collapse pattern/syndrome	a pattern/syndrome marked by sudden profuse sweating and cold skin, reversal cold of limbs, feeble breathing, palpitations, clouding or loss of consciousness, pale complexion and hardly perceptible pulse	突然の多汗と冷たい皮膚、手足厥冷、弱々しい呼吸、心悸、意識混濁または神昏、面色蒼白およびほとんど触れない脈を特徴とする証。
心火上炎證		2.9.10	pattern/syndrome of heart fire flaming upward	a pattern/syndrome of up-flaring fire from the heart, marked by oral ulceration, mental irritability, insomnia, and a red tip of the tongue	心から上方に火が燃えさかる証で、 口腔潰瘍、精神的ないらいら、失眠、および紅舌尖を特徴とする。
心火熾盛證		2.9.11	intense heart fire pattern/syndrome	a pattern/syndrome of exuberant fire disturbing the heart spirit, marked by fever, thirst, vexation, insomnia, and in severe cases, manic agitation, and delirious speech, reddened tongue tip and yellow coating, and rapid slippery pulse	盛んな火が心神を妨げる証で、発熱、口渇、心煩、失眠を特徴とし、重症例では、躁病性の煩躁、譫語、紅舌尖および黄苔、および数・滑脈が認められる。
熱擾心神證		2.9.12	pattern/syndrome of heat harassing the heart spirit	a pattern/syndrome caused by exuberant heat which disturbs the heart spirit, and marked by fever, thirst, vexation, insomnia, or even manic or delirious speech, flushed face, constipation, deep-colored urine, reddened tongue tip, yellow tongue coating and rapid slippery pulse	盛んな火が心神を妨げることにより生じる証で、発熱、口渇、心煩、失眠、または躁状態での発語（manic speech）または譫語、顔面潮紅、便秘、暗黒色の尿、紅舌尖、黄苔および数・滑脈を特徴とする。
心移熱小腸證		2.9.13	pattern/syndrome of transmission of heart heat to the small intestine	a pattern/syndrome that arises when heart fire is exuberant and spreads to the small intestine, and that is characterized by fever, thirst, vexation, oral sores with painful ulceration, rough painful voidings of reddish urine or even hematuria, reddened tongue tip and yellow coating, and rapid pulse	心火が盛んで、小腸に広がるときに生じる証で、発熱、口渇、心煩、有痛性の潰瘍を伴う口の瘡、不快な痛みを伴う赤色尿の排出または尿血、紅舌尖および黄苔、および数脈を特徴とする。
心血瘀阻證		2.9.14	heart blood stasis (obstruction) pattern/ syndrome	a pattern/syndrome arising when the blood flow in the heart vessels is impeded, marked by palpitations and stabbing pain in the precordial region	心脈の血流が妨げられるときに生じる証で、心悸および前胸部の刺痛を特徴とする。
心脈痹阻證		2.9.15	heart vessel obstruction pattern/ syndrome	a pattern/syndrome arising when the heart vessels are impeded, marked by paroxysms of palpitations with fearful throbbing, pain and a feeling of pressure in the heart and chest referring to the shoulder or upper arm	心脈が妨げられるときに生じる証で、**怔忡**を伴う心悸発作、肩または上腕に放散する心・胸部の疼痛および圧迫感を特徴とする。
痰蒙心神證		2.9.16	pattern/syndrome of phlegm clouding the heart spirit	a pattern/syndrome marked by impairment of consciousness, psychotic depression, or even coma, accompanied with phlegmatic sound in the throat	意識障害、精神的落ち込み、または昏睡とともに、喉に痰がからまる音を特徴とする証。
痰火擾心證	痰火擾心證; 痰火擾神證	2.9.17	pattern/syndrome of phlegm-fire harassing the heart	a pattern/syndrome caused by phlegm-fire which harasses the heart spirit, marked by restlessness, insomnia or even raving madness, reddened tongue tip, yellow dense and slimy tongue coating, and rapid slippery pulse	心神を苦しめる痰火により生じる証で、落ち着きのなさ、失眠または狂乱状態、紅舌尖、顆粒の密な黄・膩苔および数・滑脈を特徴とする。
痰火擾神證	痰火擾心證; 痰火擾神證	2.9.17	pattern/syndrome of phlegm-fire harassing the heart	a pattern/syndrome caused by phlegm-fire which harasses the heart spirit, marked by restlessness, insomnia or even raving madness, reddened tongue tip, yellow dense and slimy tongue coating, and rapid slippery pulse	心神を苦しめる痰火により生じる証で、落ち着きのなさ、失眠または狂乱状態、紅舌尖、顆粒の密な黄・膩苔および数・滑脈を特徴とする。
水氣凌心證		2.9.18	pattern/syndrome of water qi intimidating the heart	a pattern/syndrome arising when deficiency of yang qi of the heart and the kidney results in water flooding, marked by palpitations and shortness of breath associated with general edema, especially in the legs, short voidings of scanty clear urine, associated with listlessness, lassitude, cold extremities, pale or dark gloomy complexion, pale plump tongue with white slippery coating and sunken weak pulse	心・腎の陽気の虚により水があふれ出るときに生じる証で、全身、とくに四肢の水腫に伴う心悸および短気、短時間の少量の清澄尿の排出とともに、無気力、倦怠感、四肢の冷え、蒼白または暗く陰うつな顔、白・滑苔を伴う膨らんだ淡白舌および弱い沈脈を特徴とする。

検索表示（原表示の複数語を分離）	IST原表示	Code	Term	Definition/Description	定義/記述
瘀阻腦絡證		2.9.19	pattern/syndrome of (blood) stasis obstructing the brain collateral	a pattern/syndrome arising when a brain collateral is obstructed by static blood, marked by dizziness, headache with fixed location, or forgetfulness, insomnia, or loss of consciousness, dull lusterless complexion, purple tongue or tongue with purple spots, and fine choppy pulse	瘀血により脳の脈絡が閉塞するときに生じる証で、めまい、固定された部位の頭痛、または健忘症、失眠または神昏、くすんでつやのない顔色、紫舌または紫斑のある舌および不規則に変わる細脈を特徴とする。
氣閉神厥證		2.9.20	pattern/syndrome of qi block with syncope	a pattern/syndrome arising when emotional stimuli cause obstruction of qi movement involving the heart spirit, marked by fainting, aphasia or loss of consciousness, trismus, convulsions and string-like or hidden pulse	感情的な刺激により心神に関与する気機が閉塞することで引き起こされる証で、昏厥、失語症または神昏、開口障害、抽搐および弦または伏脈を特徴とする。
飲停心包證		2.9.21	pattern/syndrome of fluid retention in the pericardium	a pattern/syndrome caused by fluid retention in the pericardium impeding the flow of qi and blood, marked by a forceful heart beat, fullness and a feeling of pressure in the chest, dyspnea with inability to lie flat, purplish tongue with white slippery coating, and sunken or hidden pulse	心膜の飲（津の停滞）により気および血の流れが妨げられることで引き起こされる証で、力強い心拍、胸部の充満および圧迫感、喘のために横になれない、白・滑苔を伴う紫舌、および沈脈または伏脈を特徴とする。
小腸氣滯證		2.9.22	small intestinal qi stagnation pattern/ syndrome	a pattern/syndrome caused by qi stagnation in the small intestine, marked by abdominal pain with borborygmi	小腸の気滞により生じる証で、腹鳴を伴う腹痛を特徴とする。
肺病辨證		2.9.23	Lung diseases Pattern Identification/ Syndrome Differentiation	visceral pattern identification/syndrome differentiation dealing with lung diseases	肺の疾病を取り扱う臟の弁証論治。
肺氣虛證	肺氣虛證; 肺氣虧虛證	2.9.24	lung qi deficiency pattern/syndrome	a pattern/syndrome marked by pale complexion, shortness of breath, feeble voice, intolerance of wind and spontaneous sweating	面色蒼白、短気、弱々しい声、風の不耐および自汗を特徴とする証。
肺氣虧虛證	肺氣虛證; 肺氣虧虛證	2.9.24	lung qi deficiency pattern/syndrome	a pattern/syndrome marked by pale complexion, shortness of breath, feeble voice, intolerance of wind and spontaneous sweating	面色蒼白、短気、弱々しい声、風の不耐および自汗を特徴とする証。
肺陰虛證	肺陰虛證; 肺陰虧虛證	2.9.25	lung yin deficiency pattern/syndrome	a pattern/syndrome attributed to yin deficiency of the lung with endogenous heat, manifested by unproductive cough, afternoon fever, night sweating, flushed cheeks, dry throat, red and dry tongue, and rapid fine pulse	肺陰の虚および体内の熱により生じる証で、乾咳、午後の発熱、盗汗、頬部潮紅、喉の乾燥、紅舌・舌乾および数・細脈が発現する。
肺陰虧虛證	肺陰虛證; 肺陰虧虛證	2.9.25	lung yin deficiency pattern/syndrome	a pattern/syndrome attributed to yin deficiency of the lung with endogenous heat, manifested by unproductive cough, afternoon fever, night sweating, flushed cheeks, dry throat, red and dry tongue, and rapid fine pulse	肺陰の虚および体内の熱により生じる証で、乾咳、午後の発熱、盗汗、頬部潮紅、喉の乾燥、紅舌・舌乾および数・細脈が発現する。
肺陽虛證		2.9.26	lung yang deficiency pattern/syndrome	a pattern/syndrome resulting from deficiency of yang qi to warm the lung, usually manifested by cough, dyspnea and thin expectoration, fear of cold and cold extremities, spontaneous sweating, pale complexion, enlarged tongue with white slippery coating and weak pulse	肺を温める陽気の虚により生じる証で、通常、咳嗽、喘および希薄な喀出物、畏寒および四肢の冷え、自汗、面色蒼白、白・滑苔を伴う胖大舌および弱脈が発現する。
風寒襲肺證		2.9.27	pattern/syndrome of wind-cold assailing the lung	a pattern/syndrome marked by chilliness, stuffy nose, sneezing, profuse watery nasal discharge, thin sputum, thin white tongue coating and floating tight pulse	寒気、鼻づまり、噴嚔、大量の水っぽい鼻汁、希薄な痰、薄・白苔および浮・緊脈を特徴とする証。
風寒束肺證		2.9.28	pattern/syndrome of wind-cold fettering the lung	a pattern/syndrome arising when wind-cold invades the lung to cause nondiffusion of lung qi, manifested by cough with thin white phlegm, aversion to cold with possible mild fever, stuffy nose with clear nasal discharge, itchy throat, oppression in the chest, white tongue coating and floating tight pulse	風寒が肺に侵入し、肺気が宣発できなくなるときに生じる証で、希薄な白色痰を伴う咳嗽、おそらく微熱を伴う悪寒、透明な鼻汁を伴う鼻づまり、喉のかゆみ、胸悶、白苔および浮・緊脈が発現する。
風熱犯肺證		2.9.29	pattern/syndrome of wind-heat invading the lung	a pattern/syndrome marked by fever with mild chilliness, headache, sore throat, cough, reddened tip of the tongue with thin yellowish coating, and rapid floating pulse	軽度の寒気を伴う発熱、頭痛、喉の痛み、咳嗽、薄・黄苔を伴う紅舌尖、および数・浮脈を特徴とする証。
燥邪犯肺證	燥邪犯肺證; 燥邪傷肺證	2.9.30	pattern/syndrome of dryness invading the lung	a pattern/syndrome marked by dry cough without sputum or with scanty sticky sputum difficult to expectorate, chest pain, mild chills and fever, thirst, dry lips, mouth, throat and nose, and floating pulse	痰を伴わないまたは喀出困難な少量のねばつく痰を伴う乾嗽、胸痛、軽度の寒気および発熱、口渇、唇、口、喉、鼻の乾燥および浮脈を特徴とする証。
燥邪傷肺證	燥邪犯肺證; 燥邪傷肺證	2.9.30	pattern/syndrome of dryness invading the lung	a pattern/syndrome marked by dry cough without sputum or with scanty sticky sputum difficult to expectorate, chest pain, mild chills and fever, thirst, dry lips, mouth, throat and nose, and floating pulse	痰を伴わないまたは喀出困難な少量のねばつく痰を伴う乾嗽、胸痛、軽度の寒気および発熱、口渇、唇、口、喉、鼻の乾燥および浮脈を特徴とする証。
肺熱證		2.9.31	lung heat pattern/ syndrome	a general term for heat patterns/syndromes of the lung	肺熱の証の一般用語。
肺熱熾盛證		2.9.32	intense lung heat pattern/syndrome	a pattern/syndrome marked by fever, thirst, cough, dyspnea or chest pain, constipation, dark urine, reddened tongue with yellow coating	発熱、口渇、咳嗽、喘または胸痛、便秘、暗黒色の尿、黄苔を伴う紅舌を特徴とする証。
痰熱閉肺證		2.9.33	pattern/syndrome of phlegm- heat obstructing the lung	a pattern/syndrome marked by cough, dyspnea, expectoration of thick, yellow or blood-stained sputum, chest pain, reddened tongue with yellowish slimy coating and rapid slippery pulse	咳嗽、喘、粘稠な黄色または血の付着した痰の喀出、胸痛、黄・膩苔を伴う紅舌および数・滑脈を特徴とする証。
痰濁阻肺證		2.9.34	pattern/syndrome of phlegm turbidity obstructing the lung	a pattern/syndrome marked by cough with expectoration of copious whitish sputum, feeling of stuffiness in the chest, whitish slimy coating of the tongue and soggy pulse	大量の白色痰の喀出を伴う咳嗽、胸部が詰まった感じ、白・膩苔および濡脈を特徴とする証。
寒痰阻肺證		2.9.35	pattern/syndrome of cold- phlegm obstructing the lung	a pattern/syndrome marked by cough with profuse phlegm easy to expectorate and a feeling of pressure in the chest, or phlegmatic wheezing, aversion to cold and cold limbs, pale tongue with white slimy or slippery coating, and string-like slippery pulse	喀出しやすい大量の痰を伴う咳嗽および胸部圧迫感、または痰を伴う喘鳴、悪寒および四肢の冷え、白・膩苔または滑苔を伴う淡白舌、および弦・滑脈を特徴とする証。

検索表示（原表示の複数語を分離）	IST原表示	Code	Term	Definition/Description	定義/記述
暑傷肺絡證		2.9.36	pattern/syndrome of summerheat damaging the lung vessel	a pattern/syndrome marked by fever, thirst, cough, expectoration of fresh blood, reddened tongue with yellow fur and rapid weak pulse	発熱、口渇、咳嗽、鮮血の喀出、黄苔を伴う紅舌および数・弱脈を特徴とする証。
熱毒閉肺證		2.9.37	pattern/syndrome of heat toxin blocking the lung	a pattern/syndrome marked by fever, reversal cold of limbs, cough, dyspnea, a feeling of pressure in the chest with coarse breath, reddened tongue with yellow coating and rapid sunken pulse	発熱、手足厥冷、咳嗽、喘、荒い呼吸を伴う胸部圧迫感、黄苔を伴う紅舌および数・沈脈を特徴とする証。
肺燥腸閉證		2.9.38	pattern/syndrome of lung dryness with intestinal obstruction	a pattern/syndrome marked by cough, dyspnea, thirst, constipation, abdominal distension, yellow dry coating of the tongue and sunken replete pulse	咳嗽、喘、口渇、便秘、腹部膨満、黄・燥苔および沈・実脈を特徴とする証。
脾病辨證		2.9.39	Spleen Diseases Pattern Identification/ Syndrome Differentiation	visceral pattern identification/syndrome differentiation dealing with spleen diseases	脾の疾病を取り扱う臓の 弁証論治。
脾虛證		2.9.40	spleen deficiency pattern/syndrome	any deficiency pattern/syndrome of the spleen, including deficiency of spleen qi, yin and yang	脾の気、陰、陽の虚を含む脾虚の証。
脾氣虧虛證	脾氣虛證; 脾氣虧虛證	2.9.41	spleen qi deficiency pattern/syndrome	a pattern/syndrome marked by dizziness, fatigue, sallow face, indigestion, abdominal distension, lassitude, anorexia and loose bowels	めまい、疲労、面色萎黄、消化不良、腹部膨満、倦怠感、厭食および下痢を特徴とする証。
脾氣虛證	脾氣虛證; 脾氣虧虛證	2.9.41	spleen qi deficiency pattern/syndrome	a pattern/syndrome marked by dizziness, fatigue, sallow face, indigestion, abdominal distension, lassitude, anorexia and loose bowels	めまい、疲労、面色萎黄、消化不良、腹部膨満、倦怠感、厭食および下痢を特徴とする証。
脾失健運證		2.9.42	pattern/syndrome of spleen failing in transportation	a pattern/syndrome that occurs in any deficiency condition of the spleen and that is often characterized by anorexia, abdominal distention, diarrhea, borborygmi, and in chronic cases, emaciation, lack of strength, and edema of limbs	脾が虚の状態で生じる証で、しばしば、厭食、腹部膨満、泄瀉、腹鳴を特徴とし、慢性例ではるいそう、乏力、四肢の水腫が認められる。
脾陰虛證	脾陰虛證; 脾陰虧虛證	2.9.43	spleen yin deficiency pattern/syndrome	a pattern/syndrome attributed to deficiency of yin fluid with impaired splenic transportation, and marked by hunger with inability to eat, emaciation and lassitude	陰津の虚および脾の運化障害により生じる証で、摂食不能による空腹感、るいそうおよび倦怠感を特徴とする。
脾陰虧虛證	脾陰虛證; 脾陰虧虛證	2.9.43	spleen yin deficiency pattern/syndrome	a pattern/syndrome attributed to deficiency of yin fluid with impaired splenic transportation, and marked by hunger with inability to eat, emaciation and lassitude	陰津の虚および脾の運化障害により生じる証で、摂食不能による空腹感、るいそうおよび倦怠感を特徴とする。
脾陽虛證	脾陽虛證; 脾陽虧虛證; 脾虛寒證	2.9.44	spleen yang deficiency pattern/ syndrome	a pattern/syndrome attributed to insufficient yang qi failing to warm and activate the spleen, usually manifested by cold limbs, coldness and pains in the abdomen, anorexia, abdominal fullness, chronic diarrhea, lassitude, emaciation and edema, the same as the spleen deficiency cold pattern/syndrome	陽気不足により、脾を温め活性化できないことに起因する証で、通常、四肢の冷え、腹部の冷えおよび疼痛、厭食、腹満、久泄、倦怠感、るいそうおよび水腫が発現する。spleen deficiency cold pattern/syndromeと同義。
脾陽虧虛證	脾陽虛證; 脾陽虧虛證; 脾虛寒證	2.9.44	spleen yang deficiency pattern/ syndrome	a pattern/syndrome attributed to insufficient yang qi failing to warm and activate the spleen, usually manifested by cold limbs, coldness and pains in the abdomen, anorexia, abdominal fullness, chronic diarrhea, lassitude, emaciation and edema, the same as the spleen deficiency cold pattern/syndrome	陽気不足により、脾を温め活性化できないことに起因する証で、通常、四肢の冷え、腹部の冷えおよび疼痛、厭食、腹満、久泄、倦怠感、るいそうおよび水腫が発現する。spleen deficiency cold pattern/syndromeと同義。
脾虛寒證	脾陽虛證; 脾陽虧虛證; 脾虛寒證	2.9.44	spleen yang deficiency pattern/ syndrome	a pattern/syndrome attributed to insufficient yang qi failing to warm and activate the spleen, usually manifested by cold limbs, coldness and pains in the abdomen, anorexia, abdominal fullness, chronic diarrhea, lassitude, emaciation and edema, the same as the spleen deficiency cold pattern/syndrome	陽気不足により、脾を温め活性化できないことに起因する証で、通常、四肢の冷え、腹部の冷えおよび疼痛、厭食、腹満、久泄、倦怠感、るいそうおよび水腫が発現する。spleen deficiency cold pattern/syndromeと同義。
脾不統血證		2.9.45	pattern/syndrome of spleen failing to control the blood	a pattern/syndrome that arises when weak spleen qi fails to control blood, resulting in various kinds of chronic bleeding such as purpura, flooding and spotting in women, accompanied by sallow complexion, anorexia, loose bowels, lassitude, lack of strength, shortness of breath, laziness to speak, pale tongue and weak pulse	脾気が弱いために血を制御できないときに生じる証で、紫斑、女性の崩漏など、種々の慢性出血とともに、面色萎黄、厭食、下痢、倦怠感、乏力、短気、話すのが億劫、淡白舌および弱脈が認められる。
脾虛氣陷證	脾虛氣陷證; 脾氣下陷證	2.9.46	sunken spleen qi pattern/syndrome	a pattern/syndrome marked by epigastric bearing-down sensation, more prominent after meals, or urgency of defecation at short intervals with bearing-down feeling of the anus, or chronic persistent diarrhea, or prolapse of the rectum or uterus, associated with shortness of breath, lassitude, reluctance to speak, dizziness, pale tongue with white coating, and relaxed weak pulse, the same as the pattern/syndrome of spleen deficiency with sunken qi	食後に顕著な、心窩部が下垂する感じ、または肛門が下垂する感じを伴う頻繁な便意切迫または持続性の久泄、または直腸脱および子宮脱とともに、短気、倦怠感、話すのが億劫、めまい、白苔を伴う淡白舌、および緩・弱脈を特徴とする証。pattern/syndrome of spleen deficiency with sunken qiと同義。
脾氣下陷證	脾虛氣陷證; 脾氣下陷證	2.9.46	sunken spleen qi pattern/syndrome	a pattern/syndrome marked by epigastric bearing-down sensation, more prominent after meals, or urgency of defecation at short intervals with bearing-down feeling of the anus, or chronic persistent diarrhea, or prolapse of the rectum or uterus, associated with shortness of breath, lassitude, reluctance to speak, dizziness, pale tongue with white coating, and relaxed weak pulse, the same as the pattern/syndrome of spleen deficiency with sunken qi	食後に顕著な心窩部が下垂する感じ、または肛門が下垂する感じを伴う頻繁な便意切迫または持続性の久泄、または直腸脱および子宮脱とともに、短気、倦怠感、話すのが億劫、めまい、白苔を伴う淡白舌、および緩・弱脈を特徴とする証。pattern/syndrome of spleen deficiency with sunken qiと同義。

検索表示（原表示の複数語を分離）	IST原表示	Code	Term	Definition/Description	定義/記述
脾虛濕困證		2.9.47	pattern/syndrome of spleen deficiency with dampness encumbrance	a pattern/syndrome marked by epigastric distension, poor appetite, borborygmi, diarrhea, nausea, thirst but no desire to drink, lassitude, and dense and slippery tongue coating, the same as the pattern/syndrome of spleen deficiency with dampness accumulation	心窩部膨満、食欲不振、腹鳴、泄瀉、悪心、口渇はあるが飲水欲はない、倦怠感、および顆粒が密な滑苔を特徴とする証。pattern/syndrome of spleen deficiency with dampness accumulationと同義。
脾虛動風證		2.9.48	pattern/syndrome of spleen deficiency with stirring of wind	a pattern/syndrome marked by tremor of the limbs or convulsions associated with reduced food intake, abdominal distension, loose bowels, lassitude, lack of strength, pale complexion, pale tongue and weak pulse	四肢の顫震または抽搐とともに、摂食量減少、腹部膨満、下痢、倦怠感、乏力、面色蒼白、淡白舌および弱脈を特徴とする証。
脾虛水泛證		2.9.49	pattern/syndrome of spleen deficiency with water flood	a pattern/syndrome marked by edema of the face and limbs or together with ascites, associated with reduced food intake, abdominal distension, sloppy stools, lassitude, lack of strength, pale complexion, pale plump tongue with white slippery coating, and soggy or weak pulse	顔および四肢の水腫または腹水とともに、摂食量減少、腹部膨満、便溏、倦怠感、乏力、面色蒼白、白・滑苔を伴う膨らんだ淡白舌および濡・弱脈を特徴とする証。
寒濕困脾證	寒濕困脾證;濕困脾陽證	2.9.50	pattern/syndrome of cold-dampness encumbering the spleen	a pattern/syndrome marked by epigastric and abdominal distention, stickiness and tastelessness in the mouth, nausea, loose bowels, heaviness sensation of the head and body, or jaundice with dull yellow discoloration, pale plump tongue with white slimy coating and soggy relaxed pulse	心窩部・腹部膨満、口のねばつきおよび食べても味がない、悪心、下痢、頭部および身体が重い感じ、くすんだ黄色の変色がみられる黄疸、白膩苔を伴う膨らんだ淡白舌および弛緩した濡脈を特徴とする証。
濕困脾陽證	寒濕困脾證;濕困脾陽證	2.9.50	pattern/syndrome of cold-dampness encumbering the spleen	a pattern/syndrome marked by epigastric and abdominal distention, stickiness and tastelessness in the mouth, nausea, loose bowels, heaviness sensation of the head and body, or jaundice with dull yellow discoloration, pale plump tongue with white slimy coating and soggy relaxed pulse	心窩部・腹部膨満、口のねばつきおよび食べても味がない、悪心、下痢、頭部および身体が重い感じ、くすんだ黄色の変色がみられる黄疸、白・膩苔を伴う膨らんだ淡白舌および濡・緩脈を特徴とする証。
濕熱蘊脾證		2.9.51	pattern/syndrome of dampness-heat in the spleen	a pattern/syndrome marked by abdominal distention, nausea, vomiting, anorexia, heaviness sensation in the limbs, or jaundice, reddened tongue with yellow slimy coating and rapid soggy pulse	腹部膨満、悪心、嘔吐、厭食、四肢が重い感じ、または黄疸、黄・膩苔を伴う紅舌および数・濡脈を特徴とする証。
脾胃濕熱證	脾胃濕熱證;中焦濕熱證	2.9.52	pattern/syndrome of dampness-heat in the spleen and stomach	a pattern/syndrome marked by epigastric or abdominal distention, anorexia, nausea, vomiting, lassitude, heaviness sensation of the body, or jaundice with bright yellow discoloration of the skin and the white of the eyes, yellow dense and slimy tongue coating, the same as the pattern/syndrome of dampness-heat in the middle energizer	心窩部・腹部膨満、厭食、悪心、嘔吐、倦怠感、身体が重い感じ、または皮膚の明るい黄色の変色を伴う黄疸および白睛、顆粒が密な黄・膩苔を特徴とする証。patern/syndrome of dampness-heat in the middle energizerと同義。
中焦濕熱證	脾胃濕熱證;中焦濕熱證	2.9.52	pattern/syndrome of dampness-heat in the spleen and stomach	a pattern/syndrome marked by epigastric or abdominal distention, anorexia, nausea, vomiting, lassitude, heaviness sensation of the body, or jaundice with bright yellow discoloration of the skin and the white of the eyes, yellow dense and slimy tongue coating, the same as the pattern/syndrome of dampness-heat in the middle energizer	心窩部・腹部膨満、厭食、悪心、嘔吐、倦怠感、身体が重い感じ、または皮膚の明るい黄色の変色を伴う黄疸および白睛、顆粒が密な黄・膩苔を特徴とする証。patern/syndrome of dampness-heat in the middle energizerと同義。
脾胃虛寒證	脾胃虛寒證;脾胃陽虛證	2.9.53	spleen-stomach deficiency cold pattern/syndrome	a pattern/syndrome attributed to insufficient yang qi to warm the spleen and stomach with endogenous cold, and manifested by cold and pains over the stomach, accompanied by anorexia, abdominal fullness, belching, vomiting thin fluid, chronic diarrhea, lassitude and cold limbs, the same as the spleen-stomach yang deficiency pattern/syndrome	脾・胃を温める陽気の不足とともに内寒により生じる証で、胃全体の冷えおよび疼痛とともに、厭食、腹満、噯気、希薄な液体の嘔吐、久泄、倦怠感および四肢の冷えが発現する。spleen-stomach yo deficiency pattern/syndromeと同義。
脾胃陽虛證	脾胃虛寒證;脾胃陽虛證	2.9.53	spleen-stomach deficiency cold pattern/syndrome	a pattern/syndrome attributed to insufficient yang qi to warm the spleen and stomach with endogenous cold, and manifested by cold and pains over the stomach, accompanied by anorexia, abdominal fullness, belching, vomiting thin fluid, chronic diarrhea, lassitude and cold limbs, the same as the spleen-stomach yang deficiency pattern/syndrome	脾・胃を温める陽気の不足とともに内寒により生じる証で、胃全体の冷えおよび疼痛とともに、厭食、腹満、噯気、希薄な液体の嘔吐、久泄、倦怠感および四肢の冷えが発現する。spleen-stomach yang deficiency pattern/syndromeと同義。
脾胃虛弱證		2.9.54	spleen-stomach weakness pattern/syndrome	a combined pattern/syndrome of spleen qi deficiency and stomach qi deficiency	脾気虚と胃気虚が組み合わさった証。
脾胃陰虛證		2.9.55	spleen-stomach yin deficiency pattern/syndrome	a pattern/syndrome attributed to insufficient yin fluid to moisten the spleen and stomach, and manifested by dry mouth and throat, hunger but no desire for food, or gastric upset, abdominal distension and dull pain, retching and hiccough, leanness, constipation, reddened tongue lacking moisture and fine rapid pulse	脾・胃を潤す陰津の不足により生じる証で、口乾および喉の乾燥、空腹であるが食物に対する欲求がない、または嘈雜、または腹部膨満および隠痛、または噦およびしゃっくり、るいそう、便秘、潤いのない紅舌および細・数脈が発現する。
脾胃不和證		2.9.56	spleen-stomach disharmony pattern/ syndrome	a pattern/syndrome attributed to stagnation of qi movement that causes dysfunction of the spleen and stomach, and manifested by epigastric stuffiness and distension, anorexia, sloppy stool, belching, borborygmi and string-like pulse	気機のうっ滞により脾および胃の機能障害が生じることに起因する証で、心窩部の閉塞感および膨満、厭食、便溏、噯気、腹鳴および弦脈が発現する。
胃腸病辨證		2.9.57	Gastrointestinal Pattern Identification/ Syndrome Differentiation	visceral pattern identification/syndrome differentiation dealing with diseases of the stomach and intestines	胃・腸の疾病を取り扱う臟の 弁証論治。
胃虛證		2.9.58	stomach deficiency pattern/syndrome	a collective term for various deficiency patterns/ syndromes of the stomach, including deficiency of stomach qi, yang and yin	胃の気、陽、陰の虚を含む種々の胃虚の証の総称。
胃氣虛證		2.9.59	stomach qi deficiency pattern/syndrome	a pattern/syndrome marked by dull epigastric pain relieved by pressure, anorexia, pale tongue and weak pulse	圧迫すると消失する心窩部の陰痛、厭食、淡白舌および弱脈を特徴とする証。

検索表示（原表示の複数語を分離）	IST原表示	Code	Term	Definition/Description	定義/記述
胃陽虛證	胃陽虛證; 胃虛寒證	2.9.60	stomach yang deficiency pattern/syndrome	a pattern/syndrome attributed to insufficiency of yang qi to warm the stomach and marked by continuous epigastric pain, ameliorated by warmth and pressure, reduced food intake, stuffiness sensation in the stomach, aversion to cold with cold limbs, pale tongue with whitish coating and sunken slow weak pulse	胃を温める陽気の不足により生じる証で、温め圧迫すると軽減する持続性の心窩部痛、摂食量減少、胃の閉塞感、四肢の冷えを伴う悪寒、白苔を伴う淡白舌および沈・遅・弱脈を特徴とする。
胃虛寒證	胃陽虛證; 胃虛寒證	2.9.60	stomach yang deficiency pattern/syndrome	a pattern/syndrome attributed to insufficiency of yang qi to warm the stomach and marked by continuous epigastric pain, ameliorated by warmth and pressure, reduced food intake, stuffiness sensation in the stomach, aversion to cold with cold limbs, pale tongue with whitish coating and sunken slow weak pulse	胃を温める陽気の不足により生じる証で、温め圧迫すると軽減する持続性の心窩部痛、摂食量減少、胃の閉塞感、四肢の冷えを伴う悪寒、白苔を伴う淡白舌および沈・遅・弱脈を特徴とする。
胃陰虛證	胃陰虛證; 胃陰虧虛證	2.9.61	stomach yin deficiency pattern/ syndrome	a pattern/syndrome attributed to insufficiency of yin fluid to moisten the stomach and marked by dryness in the mouth, thirst, anorexia, constipation, retching and reddened peeled tongue	胃を潤す陰津の不足により生じる証で、口乾、口渇、厭食、便秘、噦および紅・光剥舌を特徴とする。
胃陰虧虛證	胃陰虛證; 胃陰虧虛證	2.9.61	stomach yin deficiency pattern/ syndrome	a pattern/syndrome attributed to insufficiency of yin fluid to moisten the stomach and marked by dryness in the mouth, thirst, anorexia, constipation, retching and reddened peeled tongue	胃を潤す陰津の不足により生じる証で、口乾、口渇、厭食、便秘、噦および紅・光剥舌を特徴とする。
胃寒證		2.9.62	stomach cold pattern/ syndrome	a pattern/syndrome of stomach cold, either of excess type or of deficiency type	胃寒が実型または虚型のいずれかである証。
胃實寒證		2.9.63	stomach excess cold pattern/syndrome	a pattern/syndrome arising when the stomach is attacked by pathogenic cold, usually manifested by acute severe epigastric pain with cold sensation, vomiting of watery fluid, aversion to cold with cold limbs and white tongue coating	胃が寒邪に攻撃されるときに生じる証で、通常、冷感を伴う急性の重度の心窩部痛、水状の津の嘔吐、四肢の冷えを伴う悪寒および白苔が発現する。
胃熱證	胃熱證; 胃火證; 胃熱壅盛證; 胃火熾盛證	2.9.64	stomach heat pattern/syndrome	a pattern/syndrome due to impairment of the stomach by pathogenic heat or caused by overeating of hot pungent food, mainly manifested by thirst, foul breath, hyperorexia, oliguria with dark urine, constipation, and ulceration of the mouth or gingivitis, reddened tongue with yellow coating and rapid pulse, the same as the (intense)stomach fire pattern/syndrome	熱邪による胃障害または熱い刺激物の過食により生じる証で、主に口渇、口臭、過食症、暗黒色の尿を伴う乏尿、便秘および口の潰瘍または歯肉炎、黄苔を伴う紅舌および数脈が発現する。(intense) stomach fire pattern/syndromeと同義。
胃火證	胃熱證; 胃火證; 胃熱壅盛證; 胃火熾盛證	2.9.64	stomach heat pattern/syndrome	a pattern/syndrome due to impairment of the stomach by pathogenic heat or caused by overeating of hot pungent food, mainly manifested by thirst, foul breath, hyperorexia, oliguria with dark urine, constipation, and ulceration of the mouth or gingivitis, reddened tongue with yellow coating and rapid pulse, the same as the (intense)stomach fire pattern/syndrome	熱邪による胃障害または熱い刺激物の過食により生じる証で、主に口渇、口臭、過食症、暗黒色の尿を伴う乏尿、便秘および口の潰瘍または歯肉炎、黄苔を伴う紅舌および数脈が発現する。(intense) stomach fire pattern/syndromeと同義。
胃熱壅盛證	胃熱證; 胃火證; 胃熱壅盛證; 胃火熾盛證	2.9.64	stomach heat pattern/syndrome	a pattern/syndrome due to impairment of the stomach by pathogenic heat or caused by overeating of hot pungent food, mainly manifested by thirst, foul breath, hyperorexia, oliguria with dark urine, constipation, and ulceration of the mouth or gingivitis, reddened tongue with yellow coating and rapid pulse, the same as the (intense)stomach fire pattern/syndrome	熱邪による胃障害または熱い刺激物の過食により生じる証で、主に口渇、口臭、過食症、暗黒色の尿を伴う乏尿、便秘および口の潰瘍または歯肉炎、黄苔を伴う紅舌および数脈が発現する。(intense) stomach fire pattern/syndromeと同義。
胃火熾盛證	胃熱證; 胃火證; 胃熱壅盛證; 胃火熾盛證	2.9.64	stomach heat pattern/syndrome	a pattern/syndrome due to impairment of the stomach by pathogenic heat or caused by overeating of hot pungent food, mainly manifested by thirst, foul breath, hyperorexia, oliguria with dark urine, constipation, and ulceration of the mouth or gingivitis, reddened tongue with yellow coating and rapid pulse, the same as the (intense)stomach fire pattern/syndrome	熱邪による胃障害または熱い刺激物の過食により生じる証で、主に口渇、口臭、過食症、暗黒色の尿を伴う乏尿、便秘および口の潰瘍または歯肉炎、黄苔を伴う紅舌および数脈が発現する。(intense) stomach fire pattern/syndromeと同義。
瘀阻胃絡證		2.9.65	pattern/syndrome of (blood) stasis in the stomach collateral	a pattern/syndrome arising when static blood obstructs the stomach collateral, marked by epigastric stabbing pain that is aggravated by pressure or palpable mass in the epigastric region, or vomiting of dark-colored blood with clots, purple spots on the tongue and string-like choppy pulse	瘀血が胃の絡脈を閉塞するときに生じる証で、圧迫すると悪化する心窩部の刺痛または心窩部の触知可能な腫瘤、または血餅の混じった暗黒色の血の嘔吐、紫斑のある舌および不規則に変わる弦脈を特徴とする。
腸燥津虧證	腸燥津虧證; 腸燥津傷證	2.9.66	pattern/syndrome of intestinal dryness and fluid depletion	a pattern/syndrome marked by dry feces difficult to evacuate, infrequent bowel movement, abdominal distension and pain, or palpable mass in the lower abdomen, thirst, reddened tongue with scanty moisture and yellow dry coating, and string-like choppy pulse	排出困難な乾燥した便、少ない排便回数、腹部膨満および腹痛、下腹部の触知可能な腫瘤、口渇、不十分な潤苔および黄・燥苔を伴う紅舌、および 不規則に変わる弦脈を特徴とする証。
腸燥津傷證	腸燥津虧證; 腸燥津傷證	2.9.66	pattern/syndrome of intestinal dryness and fluid depletion	a pattern/syndrome marked by dry feces difficult to evacuate, infrequent bowel movement, abdominal distension and pain, or palpable mass in the lower abdomen, thirst, reddened tongue with scanty moisture and yellow dry coating, and string-like choppy pulse	排出困難な乾燥した便、少ない排便回数、腹部膨満および腹痛、下腹部の触知可能な腫瘤、口渇、不十分な潤苔および黄・燥苔を伴う紅舌、および不規則に変わる弦脈を特徴とする証。
血虛腸燥證		2.9.67	pattern/syndrome of blood deficiency and intestinal dryness	a pattern/syndrome caused by deficiency of blood depriving the intestines of moisture, and marked by dry stool difficult to defecate or accompanied by hematochezia, pale complexion and tongue, and fine choppy pulse	血の虚により腸の湿潤性が枯渇することによって生じる証で、大便乾燥による排便困難またはこれに伴う便血、面色蒼白および淡白舌、および不規則に変わる細脈を特徴とする。
寒滯胃腸證		2.9.68	pattern/syndrome of cold stagnating in stomach and intestines	a pattern/syndrome arising when pathogenic cold invades the gastrointestinal tract and gives rise to disordered transmission and transformation, usually manifested by sudden pain in the epigastric region and abdomen aggravated by cold and alleviated by warmth, nausea, vomiting, bland taste in the mouth with no thirst, watery diarrhea, aversion to cold with cold limbs, white tongue coating and string-like tight pulse	寒邪が胃腸に侵入し、伝化障害をきたしたときに生じる証で、通常、冷えると悪化し、温めると軽減する心窩部および腹部の突然の疼痛、悪心、嘔吐、口渇を伴わない口淡、水瀉、四肢の冷えを伴う悪寒、白苔および弦・緊脈が発現する。
腸道濕熱證		2.9.69	intestinal dampness-heat pattern/syndrome	a syndrome marked by discharge of purulent and bloody stools, accompanied by abdominal pain, tenesmus, scanty dark urine, yellow and slimy tongue coating, and rapid slippery pulse	膿性の便血の排出とともに、腹痛、裏急後重、少量の暗黒色の尿、黄・膩苔および数・滑脈を特徴とする症候群。

検索表示（原表示の複数語を分離）	IST原表示	Code	Term	Definition/Description	定義/記述
腸熱腑實證		2.9.70	pattern/syndrome of intestinal heat and bowel excess	a pattern/syndrome marked by high fever or late afternoon fever, abdominal fullness, pain, tenderness and refusal of pressure, constipation or heat bind with circumfluence, thirst, or impaired consciousness and delirium, short voidings of reddish urine, reddened tongue with thick yellow and dry coating, and sunken rapid forceful pulse	壮熱または夕方の発熱、腹満、疼痛、圧痛および押されるのを嫌がる、便秘または熱結、口渇または意識障害および譫妄、短時間の赤色尿の排出、厚・黄・燥苔を伴う紅舌および力強い沈・数脈を特徴とする証。
胃腸氣滯證		2.9.71	gastrointestinal qi stagnation pattern/ syndrome	a pattern/syndrome marked epigastric and abdominal distending pain or moving pain, belching, borborygmi alleviated by the passing of flatus, nausea, ungratifying diarrhea, thick tongue coating and string-like pulse	心窩部および腹部の脹痛または遊走痛、噯気、放屁により軽減する腹鳴、悪心、瀉下不爽、厚苔および弦脈を特徴とする証。
陰虛動風證		2.9.72	pattern/syndrome of yin deficiency with stirring wind	a pattern/syndrome arising when deficiency of yin fluid deprives the meridians/channels of nourishment and stirs up the internal wind, marked by twitching of extremities, accompanied by dizziness, tinnitus, flushed cheeks and dry reddened tongue	陰津の虚により滋養の経絡が枯渇し、内風が刺激されるときに生じる証で、四肢のれん縮とともに、めまい、耳鳴、頬部潮紅および乾いた紅舌を特徴とする。
飲留胃腸證		2.9.73	pattern/syndrome of fluid retention in the stomach and intestines	a pattern/syndrome caused by retention of fluid in the stomach and intestines, and marked by epigastric distension and fullness with splashing sounds in the stomach, rumbling noises in the intestines, bland taste in mouth with no thirst, white and slimy tongue coating, and sunken slippery pulse	胃腸における津の停滞により生じる証で、胃の振水音を伴う心窩部の脹満、腸の腹鳴、口渇を伴わない口淡、白・膩苔および沈・滑脈を特徴とする。
蟲積腸道證		2.9.74	pattern/syndrome of worms accumulating in the intestines	a pattern/syndrome attributed to intestinal parasitosis, especially ascariasis, and marked by paroxysms of abdominal pain, sometimes accompanied by mass formation or discharge of ascaris with stool, grinding of teeth during sleep, or appearance of white miliary spots on the inside of the lips	小腸の寄生虫病、とくに回虫症により生じる証で、時として腫瘤形成または排便時の回虫の排出を伴う腹痛発作、睡眠中の歯ぎしり、または唇内部に粟粒状の白斑がみられることを特徴とする。
大腸津虧證		2.9.75	large intestinal fluid deficiency pattern/ syndrome	a pattern/syndrome marked by constipation or difficulty in defecation accompanied by dry throat and reddened tongue with scanty coating	便秘または排便困難とともに、喉の乾燥およびまばらな舌苔を伴う紅舌を特徴とする証。
大腸熱結證		2.9.76	large intestinal heat bind pattern/ syndrome	a pattern/syndrome marked by constipation with abdominal pain and tenderness, yellow and dry coating of the tongue, and sunken forceful pulse	腹痛および圧痛を伴う便秘、黄色の燥苔および力強い沈脈を特徴とする証。
大腸濕熱證		2.9.77	large intestinal dampness-heat pattern/syndrome	a pattern/syndrome marked by discharge of purulent and bloody stools, abdominal pain, tenesmus, scanty dark urine, yellow and slimy tongue coating, and rapid slippery pulse	膿性の便血の排出、腹痛、裏急後重、少量の暗黒色の尿、黄・膩苔および数・滑脈を特徴とする証。
肝膽病辨證		2.9.78	Liver-gallbladder Diseases Pattern Identification/ Syndrome Differentiation	visceral pattern identification/syndrome differentiation dealing with liver and gallbladder diseases	肝・胆の疾病を取り扱う臓の弁証論治。
肝氣鬱結證	肝氣鬱結證; 肝鬱證	2.9.79	liver qi depression pattern/syndrome	a pattern/syndrome marked by depression, frequent sighing, hypochondriac or lower abdominal distention or moving pain, and string-like pulse; and in women, distending pain of the breast and irregular menstruation, the same as the liver qi stagnation/ constraint pattern/syndrome	鬱病、頻繁な太息、下肋部または下腹部の膨満または遊走痛および弦脈を特徴とする証。女性では乳房脹痛および月経不調が認められる。liver qi stagnation/ constraint pattern/syndromeと同義。
肝鬱證	肝氣鬱結證; 肝鬱證	2.9.79	liver qi depression pattern/syndrome	a pattern/syndrome marked by depression, frequent sighing, hypochondriac or lower abdominal distention or moving pain, and string-like pulse; and in women, distending pain of the breast and irregular menstruation, the same as the liver qi stagnation/ constraint pattern/syndrome	鬱病、頻繁な太息、下肋部または下腹部の膨満または遊走痛および弦脈を特徴とする証。女性では乳房脹痛および月経不調が認められる。liver qi stagnation/ constraint pattern/syndromeと同義。
肝血虛證	肝血虛證; 肝血虧虛證	2.9.80	liver blood deficiency pattern/syndrome	a pattern/syndrome marked by sallow complexion, impaired vision, insomnia, deficient amount or absence of menstruation, pale tongue and lips	面色委黄、視覚障害、失眠、過少月経または無月経、淡白な舌および唇を特徴とする証。
肝血虧虛證	肝血虛證; 肝血虧虛證	2.9.80	liver blood deficiency pattern/syndrome	a pattern/syndrome marked by sallow complexion, impaired vision, insomnia, deficient amount or absence of menstruation, pale tongue and lips	面色委黄、視覚障害、失眠、過少月経または無月経、淡白な舌および唇を特徴とする証。
肝陰虛證	肝陰虛證; 肝陰虧虛證	2.9.81	liver yin deficiency pattern/syndrome	a pattern/syndrome attributed to insufficient yin fluid that fails to moisten and nourish the liver, marked by dizziness, headache, blurred vision, dryness of eyes, insomnia, thirst, dry throat, scanty tongue coating and fine pulse	陰津の不足により肝を潤し滋養することができなくなることから生じる証で、めまい、頭痛、目昏、眼の乾燥、失眠、口渇、喉の乾燥、少量の舌苔および細脈を特徴とする。
肝陰虧虛證	肝陰虛證; 肝陰虧虛證	2.9.81	liver yin deficiency pattern/syndrome	a pattern/syndrome attributed to insufficient yin fluid that fails to moisten and nourish the liver, marked by dizziness, headache, blurred vision, dryness of eyes, insomnia, thirst, dry throat, scanty tongue coating and fine pulse	陰津の不足により肝を潤し滋養することができなくなることから生じる証で、めまい、頭痛、目昏、眼の乾燥、失眠、口渇、喉の乾燥、少量の舌苔および細脈を特徴とする。
肝陽虛證		2.9.82	liver yang deficiency pattern/syndrome	a pattern/syndrome due to weakened yang qi with diminished function of the liver, marked by depression and susceptibility to fright, distension and oppression in the hypochondriac regions, fear of cold with cold limbs, dizziness, blurred vision, pale tongue with white coating and sunken slow weak pulse	陽気が弱まり肝機能が低下したことにより生じる証で、鬱病、驚きやすい、下肋部の膨満および重圧感、四肢の冷えを伴う畏寒、めまい、目昏、白苔を伴う淡白舌および沈・遅・弱脈を特徴とする。
肝風內動證	肝風內動證; 肝風證	2.9.83	pattern/syndrome of internal stirring of liver wind	a pattern/syndrome marked by convulsion, tremor or spasm, also known as the liver wind pattern/ syndrome	抽搐、顫震または拘攣を特徴とする証。liver wind pattern/ syndromeとしても知られる。
肝風證	肝風內動證; 肝風證	2.9.83	pattern/syndrome of internal stirring of liver wind	a pattern/syndrome marked by convulsion, tremor or spasm, also known as the liver wind pattern/ syndrome	抽搐、顫震または拘攣を特徴とする証。liver wind pattern/ syndromeとしても知られる。
肝陽化風證		2.9.84	pattern/syndrome of liver yang transforming into wind	a pattern/syndrome marked by dizziness with tendency to fall or even sudden attack of syncope, shaking of head, tremor of limbs, irritability, irascibility, flushing of face, reddened tongue and string-like pulse	転倒傾向または突然の厥発作を伴うめまい、頭を振る、四肢の顫震、いらいらする、怒りっぽい、顔面潮紅、紅舌および弦脈を特徴とする証。

検索表示（原表示の複数語を分離）	IST原表示	Code	Term	Definition/Description	定義/記述
肝鬱氣滯證		2.9.85	pattern/syndrome of liver depression and qi stagnation	a pattern/syndrome marked by depressed mood, frequent sighing, feeling of a foreign body in the throat, distension, oppression and scurrying pain in the chest or lesser abdomen, distending pain of the breast and menstrual irregularities in women, white tongue coating and string-like pulse	抑うつ気分、頻繁な太息、異物が喉に詰まった感じ、胸部または下腹部の膨満感、重圧感および竄痛、乳房の脹痛および月経不順（女性）、白苔および弦脈を特徴とする証。
肝鬱血瘀證	肝鬱血瘀證; 肝血瘀滯證	2.9.86	pattern/syndrome of liver depression and blood stasis	a pattern/syndrome arising when stagnant liver qi causes blood stasis in the liver, marked by depressed mood, hypochondriac distending or stabbing pain, or formation of mass in the hypochondriac region or lesser abdomen, purple tongue or purple spots on the tongue, and string-like choppy pulse	うっ滞した肝気により肝で血瘀が引き起こされるときに生じる証で、抑うつ気分、下肋部の膨満または刺痛、下肋部または下腹部の腫瘤形成、紫舌または紫斑のある舌、および不規則に変わる弦脈を特徴とする。
肝血瘀滯證	肝鬱血瘀證; 肝血瘀滯證	2.9.86	pattern/syndrome of liver depression and blood stasis	a pattern/syndrome arising when stagnant liver qi causes blood stasis in the liver, marked by depressed mood, hypochondriac distending or stabbing pain, or formation of mass in the hypochondriac region or lesser abdomen, purple tongue or purple spots on the tongue, and string-like choppy pulse	うっ滞した肝気により肝で血瘀が引き起こされるときに生じる証で、抑うつ気分、下肋部の膨満または刺痛、下肋部または下腹部の腫瘤形成、紫舌または紫斑のある舌、および不規則に変わる弦脈を特徴とする。
肝鬱化火證		2.9.87	pattern/syndrome of depressed liver qi transforming into fire	a pattern/syndrome marked by distension, pain and burning sensation in the hypochondriac region, irritability, irascibility, bitterness and dryness in the mouth, reddened tongue with yellow coating and rapid string-like pulse, the same as the pattern/syndrome of stagnated liver qi transforming into fire	下肋部の膨満感、疼痛および灼熱感、いらいらする、怒りっぽい、口苦および口乾、黄苔を伴う紅舌および数・弦脈を特徴とする証。pattern/syndrome of stagnated liver qi transforming into fireと同義。
肝火上炎證		2.9.88	pattern/syndrome of liver fire flaming upward	a pattern/syndrome marked by headache, dizziness, tinnitus with buzzing in the ears, impairment of hearing, blood-shot eyes, mental irritability, bitter taste in the mouth, yellow coating of the tongue, rapid string-like pulse, and hematuria, hemoptysis or epistaxis in severe cases	頭痛、めまい、ブンブンいう音が聞こえる耳鳴、聴覚障害、目の充血、精神的ないらいら、口苦、黄苔、数・弦脈、および重症例では尿血、喀血または鼻血を特徴とする証。
肝火熾盛證		2.9.89	intense liver fire pattern/syndrome	a pattern/syndrome marked by hypochondriac pain, dryness and bitterness in the mouth, vomiting of bitter fluid, irritability, irascibility, insomnia or dream-disturbed sleep, flushed face, blood-shot eyes, constipation, dark urine, reddened tongue with yellow coating and rapid string-like pulse	脇痛、口乾および口苦、苦い津の嘔吐、いらいらする、怒りっぽい、失眠または夢に邪魔される睡眠、顔面潮紅、目の充血、便秘、暗黒色の尿、黄苔を伴う紅舌および数・弦脈を特徴とする証。
肝陽上亢證		2.9.90	pattern/syndrome of ascendant hyperactivity of liver yang	a pattern/syndrome marked by dizziness, headache, flushed face, blurred vision, tinnitus, bitter taste in the mouth and string-like pulse	めまい、頭痛、顔面潮紅、目昏、耳鳴、口苦および弦脈を特徴とする証。
肝膽濕熱證		2.9.91	liver-gallbladder dampness-heat pattern/syndrome	a pattern/syndrome marked by fever and chills, jaundice, hypochondriac and abdominal pain, bitter taste in the mouth, nausea and rapid slippery pulse	発熱および寒気、黄疸、下肋部痛および腹痛、口苦、悪心および数・滑脈を特徴とする証。
寒滯肝脈證		2.9.92	pattern/syndrome of cold stagnating in the liver meridian	a pattern/syndrome marked by spasmodic symptoms in the area related to the liver meridian, such as stretching pain with cold sensation in the lower abdomen and testicles	下腹および睾の冷感を伴う牽引痛のような、肝経に関連する領域での拘攣性症状を特徴とする証。
肝經濕熱證		2.9.93	pattern/syndrome of dampness-heat in the liver meridian	a pattern/syndrome marked by distending pain in the hypochondriac region, or itching and painful swollen genitalia, or ear pain with purulent discharge, reddened tongue with yellow slimy coating and rapid slippery pulse	臆病、驚きやすさ、失眠および夢の多い睡眠、心煩、胸部および下肋部の重圧感および膨満感、頻繁な太息、めまい、口苦、悪心、嘔吐、白・膩苔および数・滑脈を特徴とする証。
膽熱證		2.9.94	gallbladder heat pattern/syndrome	a pattern/syndrome marked by irritability, irascibility, hypochondriac distension, bitterness in the mouth, or ear pain, tinnitus, insomnia, reddened tongue with yellow coating	いらいらする、怒りっぽい、下肋部膨満、口苦または耳痛、耳鳴、失眠、黄苔を伴う紅舌を特徴とする証。
膽鬱痰擾證		2.9.95	pattern/syndrome of depressed gallbladder with harassing phlegm	a pattern/syndrome marked by timidity, susceptibility to fright, insomnia and dreamful sleep, vexation, oppression and distension in the chest and hypochondria! region, frequent sighing, dizziness, bitter taste in the mouth, nausea, vomiting, white slimy tongue coating and string-like pulse, the same as the pattern/syndrome of stagnated gallbladder with harassing phlegm	臆病、驚きやすさ、失眠および夢の多い睡眠、心煩、胸部および下肋部の重圧感および膨満感、頻繁な太息、めまい、口苦、悪心、嘔吐、白膩苔および弦脈を特徴とする証。pattern/syndrome of stagneted gallbladder with harassing phlegmと同義。
膽氣虛證	膽氣虛證; 膽氣虧虛證	2.9.96	gallbladder qi deficiency pattern/ syndrome	a pattern/syndrome marked by panic, suspicion, sighing, nervousness, irritability, lassitude, dizziness and insomnia	パニック、疑い深い、太息、神経質、いらいらする、倦怠感、めまいおよび失眠を特徴とする証。
膽氣虧虛證	膽氣虛證; 膽氣虧虛證	2.9.96	gallbladder qi deficiency pattern/ syndrome	a pattern/syndrome marked by panic, suspicion, sighing, nervousness, irritability, lassitude, dizziness and insomnia	パニック、疑い深い、太息、神経質、いらいらする、倦怠感、めまいおよび失眠を特徴とする証。
蟲擾膽腑證		2.9.97	pattern/syndrome of worms harassing the gallbladder	a pattern/syndrome marked by paroxysms of unbearable abdominal pain associated with pale complexion, reversal cold of limbs, vomiting of bitter fluid or ascarides	面色蒼白、手足厥冷、苦みのある液体または回虫の嘔吐を伴う耐えがたい腹痛発作を特徴とする証。
腎膀胱病辨證		2.9.98	Kidney-bladder Diseases Pattern Identification/ Syndrome Differentiation	visceral pattern identification/syndrome differentiation dealing with diseases of the kidney and bladder	腎・膀胱の疾病を取り扱う臓の弁証論治。
腎虛證		2.9.99	kidney deficiency pattern/syndrome	any deficiency pattern/syndrome of the kidney, including deficiency of kidney qi, yin and yang	腎の気、陰、陽の虚を含む腎虚の証。
腎精不足證		2.9.100	kidney essence insufficiency pattern/ syndrome	a pattern/syndrome marked by retarded development in children, premature senility, decreased reproductive function, tinnitus, loosening of teeth, loss of hair and forgetfulness in adults	発達遅滞（小児）、早老、生殖機能低下、耳鳴、歯のゆるみ、脱毛症および健忘症（成人）を特徴とする証。

検索表示（原表示の複数語を分離）	IST原表示	Code	Term	Definition/Description	定義/記述
腎氣虛證	腎氣虛證; 腎氣虧虛證	2.9.101	kidney qi deficiency pattern/syndrome	a pattern/syndrome marked by dizziness, forgetfulness, tinnitus, backache, lack of libido and weak pulse	めまい、健忘症、耳鳴、背痛、性欲減退および弱脈を特徴とする証。
腎氣虧虛證	腎氣虛證; 腎氣虧虛證	2.9.101	kidney qi deficiency pattern/syndrome	a pattern/syndrome marked by dizziness, forgetfulness, tinnitus, backache, lack of libido and weak pulse	めまい、健忘症、耳鳴、背痛、性欲減退および弱脈を特徴とする証。
腎氣不固證		2.9.102	kidney qi insecurity pattern/syndrome	a pattern/syndrome marked by frequent urination, dribbling of urine after voiding, incontinence of urine or feces, nocturnal emission or premature ejaculation in men, continuous dribbling of menstrual discharge or liability to abortion in women, aching back and knees, and weak pulse	小便頻数、排尿後尿滴下、小便失禁または大便滑脱、夢精または早泄（男性）、月経血の持続性滴下または流産傾向（女性）、背部および膝の酸痛、弱脈を特徴とする証。
腎陰虛證	腎陰虛證; 腎陰虧虛證	2.9.103	kidney yin deficiency pattern/syndrome	a pattern/syndrome marked by lumbago, lassitude, dizziness, tinnitus, nocturnal emission in men and oligomenorrhea in women, emaciation, dry throat, thirst, flushed cheeks, hot sensation in the palms and soles, afternoon fever, night sweating, reddened tongue with little or no coating, and rapid fine pulse	腰痛、倦怠感、めまい、耳鳴、夢精（男性）、過少月経（女性）、るいそう、喉の乾燥、口渇、頬部潮紅、手掌および足底の熱感、午後の発熱、盗汗、舌苔がほとんどまたはまったくない紅舌および数・細脈を特徴とする証。
腎陰虧虛證	腎陰虛證; 腎陰虧虛證	2.9.103	kidney yin deficiency pattern/syndrome	a pattern/syndrome marked by lumbago, lassitude, dizziness, tinnitus, nocturnal emission in men and oligomenorrhea in women, emaciation, dry throat, thirst, flushed cheeks, hot sensation in the palms and soles, afternoon fever, night sweating, reddened tongue with little or no coating, and rapid fine pulse	腰痛、倦怠感、めまい、耳鳴、夢精（男性）、過少月経（女性）、るいそう、喉の乾燥、口渇、頬部潮紅、手掌および足底の熱感、午後の発熱、盗汗、舌苔がほとんどまたはまったくない紅舌および数・細脈を特徴とする証。
腎陰虛火旺證		2.9.104	pattern/syndrome of kidney yin deficiency with fire effulgence	a pattern/syndrome marked by tidal fever, night sweating, flushed cheeks, vexing heat in the chest, palms and soles, nocturnal emission, premature ejaculation, hypersexuality, lumbar pain, tinnitus, reddened tongue with yellow coating lacking moisture and rapid fine pulse	潮熱、盗汗、頬部潮紅、胸部、手掌および足底の煩熱、夢精、早泄、性欲亢進症、腰痛、耳鳴、潤いのない黄苔を伴う紅舌および数・細脈を特徴とする証。
腎陽虛證	腎陽虛證; 腎陽虧虛證	2.9.105	kidney yang deficiency pattern/syndrome	a pattern/syndrome arising when declined kidney yang fails to warm the body, marked by aversion to cold, cold limbs, listlessness, weakness and soreness of the loins and knees, premature ejaculation or impotence in men and frigidity or infertility in women, nocturia, whitish tongue coating and weak pulse at cubit (chi) section	腎陽の低下により身体を温められないときに生じる証で、悪寒、四肢の冷え、無気力、腰部・膝の脱力および痛み、遺精または陽痿（男性）および不感症または不孕（女性）、夜間頻尿、白苔および尺部の弱脈を特徴とする。
腎陽虧虛證	腎陽虛證; 腎陽虧虛證	2.9.105	kidney yang deficiency pattern/syndrome	a pattern/syndrome arising when declined kidney yang fails to warm the body, marked by aversion to cold, cold limbs, listlessness, weakness and soreness of the loins and knees, premature ejaculation or impotence in men and frigidity or infertility in women, nocturia, whitish tongue coating and weak pulse at cubit (chi) section	腎陽の低下により身体を温められないときに生じる証で、悪寒、四肢の冷え、無気力、腰部・膝の脱力および痛み、遺精または陽痿（男性）および不感症または不孕（女性）、夜間頻尿、白苔および尺部の弱脈を特徴とする。
腎不納氣證		2.9.106	pattern/syndrome of kidney failing to receive qi	a pattern/syndrome marked by dyspnea with prolonged exhalation, asthenic cough and feeble voice	呼気の延長を伴う喘、無力性咳嗽および弱々しい声を特徴とする証。
腎虛水泛證		2.9.107	pattern/syndrome of kidney deficiency with water flood	a pattern/syndrome arising when insufficient kidney qi fails in qi transformation and leads to water flooding, marked by edema, particularly of the lower extremities, accompanied by oliguria, tinnitus, aching of the back and knees, pale tongue with whitish slippery coating and weak pulse	腎気不足により気化できず、水があふれだすときに生じる証で、水腫（とくに下肢）とともに乏尿、耳鳴、背部および膝の酸痛、白・滑苔を伴う淡白舌および弱脈を特徴とする。
腎經寒濕證		2.9.108	kidney meridian cold-dampness pattern/ syndrome	a pattern/syndrome attributed to deficiency of kidney yang with infusion of cold-dampness, and marked by feeling of heaviness, cold and pain in the lumbus and knees with limitation of movement, aversion to cold and cold limbs, white slimy tongue coating and soggy relaxed pulse	腎陽の虚と寒湿の注入により生じる証で、腰部・膝が重い感じ、冷え、疼痛および動きの制限、悪寒および四肢の冷え、白・膩苔および濡・緩脈を特徴とする。
膀胱虛寒證		2.9.109	bladder deficiency cold pattern/ syndrome	a pattern/syndrome arising when kidney yang deficiency causes impaired bladder qi transformation, marked by frequent urination, incontinence of urine or dribbling of urine, cold feeling in the lower abdomen, whitish moist tongue coating and weak pulse	腎陽の虚により膀胱の気化が障害されるときに生じる証で、小便頻数、小便失禁または尿滴下、下腹部の冷感、白・潤苔および弱脈を特徴とする。
膀胱濕熱證		2.9.110	bladder dampness-heat pattern/ syndrome	a pattern/syndrome attributed to dampness-heat attacking on and accumulating in the bladder, marked by frequency and urgency of urination, strangury, turbid urine or hematuria, reddened tongue with yellow slimy coating and rapid pulse	膀胱を攻撃し、そこに蓄積する湿熱により生じる証で、頻尿および尿意切迫、有痛排尿困難、尿濁または尿血、黄・膩苔を伴う紅舌および数脈を特徴とする。
熱積膀胱證		2.9.111	pattern/syndrome of heat accumulating in the bladder	a pattern/syndrome marked by distension and fullness of the lower abdomen, strangury, frequent urination and fever without chills	下腹の脹満、有痛排尿困難、小便頻数および寒気のない発熱を特徴とする証。
臟腑兼病辨證		2.9.112	Combined Visceral Pattern Identification/ Syndrome Differentiation	pattern identification/syndrome differentiation dealing with diseases in which two or more visceral organs are simultaneously involved	2つ以上の臓器の疾病を同時に取り扱う弁証論治。
心腎不交證		2.9.113	heart-kidney non-interaction pattern/syndrome	a pattern/syndrome attributed to deficiency of yin fluid of the heart and the kidney with relative preponderance of yang, marked by restlessness, insomnia, palpitation, dizziness, tinnitus, aching of the lower back and knees, seminal emission, vexing heat in the chest, palms and soles, night sweating, reddened tongue with scanty coating and rapid fine pulse	心・腎の陰津の虚と、相対的に優勢な陽により生じる証で、落ち着きのなさ、失眠、心悸、めまい、耳鳴、背部および膝の酸痛、遺精、胸部、手掌および足底の煩熱、盗汗、少量の舌苔を伴う紅舌および数・細脈を特徴とする。
心腎陽虛證		2.9.114	heart-kidney yang deficiency pattern/ syndrome	a pattern/syndrome arising when insufficient yang qi fails to warm and activate both the heart and the kidney, marked by palpitations, aversion to cold, lack of warmth in the extremities, inhibited urination, edema of the legs, aching and cold lumbus and knees, white slimy tongue coating and weak sunken pulse	陽気不足により、心・腎の両者を温め活性化できないときに生じる証で、心悸、悪寒、四肢が暖かくない、小便不利、脚の水腫、腰部および膝の酸痛および冷え、白・膩苔および弱・沈脈を特徴とする。

検索表示（原表示の複数語を分離）	IST原表示	Code	Term	Definition/Description	定義/記述
心肺氣虛證		2.9.115	heart-lung qi deficiency pattern/syndrome	a pattern/syndrome marked by palpitations, a feeling of pressure in the chest, cough, dyspnea and shortness of breath aggravated on exertion, thin expectoration, dizziness, listlessness and lack of strength, feeble voice, spontaneous sweating, pale tongue and weak pulse	心悸、胸部圧迫感、咳嗽、激しい身体活動により悪化する喘および短気、希薄な喀出物、めまい、無気力および乏力、弱々しい声、自汗、淡白舌および弱脈を特徴とする証。
心脾兩虛證		2.9.116	pattern/syndrome of dual deficiency of the heart and spleen	a pattern/syndrome marked by palpitation, amnesia, insomnia or dream-disturbed sleep, loss of appetite, abdominal distention, loose bowels, lassitude, sallow face, pale tender-soft tongue and fine weak pulse	心悸、健忘症、失眠または夢に邪魔される睡眠、食欲不振、腹部膨満、下痢、倦怠感、面色委黄、淡白・嫩舌および細・弱脈を特徴とする証。
心肝血虛證		2.9.117	heart-liver blood deficiency pattern/syndrome	a pattern/syndrome marked by palpitations, forgetfulness, insomnia, dream-disturbed sleep, dizziness, blurred vision, pale complexion, numbness of the limbs, lusterless nails, scanty volume of pale menstrual flow or even amenorrhea in women, pale tongue and fine pulse	心悸、健忘症、失眠、夢に邪魔される睡眠、めまい、目昏、面色蒼白、四肢の麻木、つやのない爪、少量の薄い色の月経または無月経（女性）、淡白舌および細脈を特徴とする証。
肺腎氣虛證		2.9.118	lung-kidney qi deficiency pattern/syndrome	a pattern/syndrome marked by dyspnea with prolonged exhalation, spontaneous sweating, lack of strength, aching lumbus and limp legs, and cough with thin sputum	喘、喘息、短気、自汗、乏力、腰部の酸痛および跛行、および希薄な痰を伴う咳嗽を特徴とする証。
肺腎陰虛證		2.9.119	lung-kidney yin deficiency pattern/syndrome	a pattern/syndrome attributed to deficiency of yin fluid of the lung and the kidney with harassment of endogenous heat, marked by cough with scanty expectoration, dryness of the mouth and throat or hoarseness of voice, aching lumbus and limp legs, bone-steaming tidal fever, flushed cheeks, night sweats, nocturnal emission in men and menstrual irregularities in women, reddened tongue with scanty coating and rapid fine pulse	肺・腎の陰津が不足し、内熱が苦しまされることにより生じる証で、少量の喀出物を伴う咳嗽、口および喉の乾燥または嘶嗄、腰部の酸痛および跛行、骨蒸、潮熱、頬部潮紅、盗汗、夢精（男性）および月経不調（女性）、少量の舌苔を伴う紅舌 および速・細脈を特徴とする。
肺腎陽虛證	肺腎陽虛證; 水寒射肺證	2.9.120	lung-kidney yang deficiency pattern/syndrome	a pattern/syndrome arising when debilitated kidney yang causes water flood harassing the lung, marked by aversion to cold with cold limbs, cough and dyspnea with expectoration of profuse white thin sputum, edema of legs, oliguria, pale enlarged tongue with whitish slippery coating and weak pulse	腎陽の衰退により水があふれ出し、肺が苦しめられることにより生じる証で、四肢の冷えを伴う悪寒、大量の白色の希薄な痰の喀出を伴う咳嗽および喘、四肢の水腫、乏尿、白・滑苔を伴う膨らんだ淡白舌および弱脈を特徴とする。
水寒射肺證	肺腎陽虛證; 水寒射肺證	2.9.120	lung-kidney yang deficiency pattern/syndrome	a pattern/syndrome arising when debilitated kidney yang causes water flood harassing the lung, marked by aversion to cold with cold limbs, cough and dyspnea with expectoration of profuse white thin sputum, edema of legs, oliguria, pale plump tongue with whitish slippery coating and weak pulse	腎陽の衰退により水があふれ出し、肺が苦しめられることにより生じる証で、四肢の冷えを伴う悪寒、大量の白色の希薄な痰の喀出を伴う咳嗽および喘、四肢の水腫、乏尿、白・滑苔を伴う膨らんだ淡白舌および弱脈を特徴とする。
脾肺氣虛證	脾肺氣虛證; 脾肺兩虛證	2.9.121	spleen-lung qi deficiency pattern/syndrome	a pattern/syndrome marked by anorexia, loose stools, abdominal distension, protracted cough with dyspnea and shortness of breath, expectoration of large amount of thin sputum, feeble voice, laziness to speak, pale lusterless complexion, pale tongue with white slippery coating and weak fine pulse	厭食、軟便、腹部膨満、喘を伴う長引く咳嗽および短気、大量の希薄な痰の喀出、弱々しい声、話すのが億劫、蒼白のつやのない顔色、白・滑苔を伴う淡白舌および弱・細脈を特徴とする証。
脾肺兩虛證	脾肺氣虛證; 脾肺兩虛證	2.9.121	spleen-lung qi deficiency pattern/syndrome	a pattern/syndrome marked by anorexia, loose stools, abdominal distension, protracted cough with dyspnea and shortness of breath, expectoration of large amount of thin sputum, feeble voice, laziness to speak, pale lusterless complexion, pale tongue with white slippery coating and weak fine pulse	厭食、軟便、腹部膨満、喘を伴う長引く 咳嗽および短気、大量の希薄な痰の喀出、弱々しい声、話すのが億劫、蒼白のつやのない顔色、白・滑苔を伴う淡白舌および弱・細脈を特徴とする証。
肝火犯肺證		2.9.122	pattern/syndrome of liver fire invading the lung	a pattern/syndrome marked by bitter taste in the mouth, dizziness and red eyes, irritability, irascibility, moving pain in the chest and hypochondriac region, cough with thick expectoration or even hemoptysis, reddened tongue and rapid tight string-like pulse	口苦、めまいおよび目の充血、いらいらする、怒りっぽい、胸部および下肋部の遊走痛、粘稠な喀出物または喀血を伴う咳嗽、紅舌および速・緊・弦脈を特徴とする証。
肝氣犯胃證	肝氣犯胃證; 肝胃不和證	2.9.123	pattern/syndrome of liver qi invading the stomach	a pattern/syndrome marked by dizziness, hypochondriac pain, irritability, epigastric distension and pain, anorexia, belching, nausea, vomiting and string-like pulse, also known as the liver-stomach disharmony pattern/syndrome	めまい、脇痛、いらいらする、心窩部の膨満感および疼痛、厭食、噯気、悪心、嘔吐および弦脈を特徴とする証。liver-stomach disharmony pattern/syndromeとしても知られる。
肝胃不和證	肝氣犯胃證; 肝胃不和證	2.9.123	pattern/syndrome of liver qi invading the stomach	a pattern/syndrome marked by dizziness, hypochondriac pain, irritability, epigastric distension and pain, anorexia, belching, nausea, vomiting and string-like pulse, also known as the liver-stomach disharmony pattern/syndrome	めまい、脇痛、いらいらする、心窩部の膨満感および疼痛、厭食、噯気、悪心、嘔吐および弦脈を特徴とする証。liver-stomach disharmony pattern/syndromeとしても知られる。
肝鬱脾虛證	肝鬱脾虛證; 肝脾不調證	2.9.124	pattern/syndrome of liver depression and spleen deficiency	a pattern/syndrome marked by hypochondriac and abdominal painful distension, depressed mood, frequent sighing, anorexia, ungratifying loose bowels or alleviation of abdominal pain after defecation,borborygmi with flatus and white slimy tongue coating,the same as the pattern/syndrome of liver stagnation and spleen dificiency,also known as the liver-spleen dishaharmony pattern/syndrome	下肋部および腹部の疼痛を伴う膨満感、抑うつ気分、頻繁な太息、厭食、不快な下痢または排便後に軽減する腹痛、放屁を伴う腹鳴および白・膩苔を特徴とする証。pattern/syndrome of liver stagnation and spleen dificiencyと同義。liver-spleen dishaharmony pattern/syndromeとしても知られる。
肝脾不調證	肝鬱脾虛證; 肝脾不調證	2.9.124	pattern/syndrome of liver depression and spleen deficiency	a pattern/syndrome marked by hypochondriac and abdominal painful distension, depressed mood, frequent sighing, anorexia, uncomfortable loose bowels or alleviation of abdominal pain after defecation, borborygmi with flatus and white slimy tongue coating, the same as the pattern/syndrome of liver stagnation and spleen deficiency, also known as the liver-spleen dishaharmony pattern/syndrome	下肋部および腹部の疼痛を伴う膨満感、抑うつ気分、頻繁な太息、厭食、不快な下痢または排便後に軽減する腹痛、放屁を伴う腹鳴および白・膩苔を特徴とする証。pattern/syndrome of liver stagnation and spleen dificiencyと同義。liver-spleen dishaharmony pattern/syndromeとしても知られる。

検索表示（原表示の複数語を分離）	IST原表示	Code	Term	Definition/Description	定義/記述
肝腎陰虛證		2.9.125	liver-kidney yin deficiency pattern/syndrome	a pattern/syndrome attributed to insufficiency of yin fluid of the liver and the kidney with harassment of endogenous heat, marked by dizziness, blurred vision, tinnitus, forgetfulness, insomnia and dream-disturbed sleep, hypochondriac pain, aching lumbus and poor muscle tone in legs, flushed cheeks, heat sensation in the chest, palms and soles, night sweating, nocturnal emission in men and scant menstruation in women, reddened tongue with scanty coating, and rapid fine pulse	肝・腎の陰津が不足し、内熱が苦しめられることにより生じる証で、めまい、目昏、耳鳴、健忘症、失眠および夢に邪魔される夢、脇痛、腰部の酸痛および跛行、頬部潮紅、胸部、手掌および足底の熱感、盗汗、夢精（男性）および月経過少（女性）、少量の舌苔を伴う紅舌 および速・細脈を特徴とする。
脾腎陽虛證		2.9.126	spleen-kidney yang deficiency pattern/syndrome	a pattern/syndrome attributed to insufficient yang qi of the spleen and the kidney with endogenous cold, marked by bright pale complexion, aversion to cold with cold limbs, coldness and pain in the loins and lower abdomen, chronic diarrhea, or edema with inhibited urination, pale enlarged tongue with white slippery coating, and slow, sunken and forceless pulse	脾・腎の陽気の不足とともに内寒により生じる証で、面色晄白、四肢の冷えを伴う悪寒、腰部および下腹の冷えおよび疼痛、久泄、または小便不利を伴う水腫、白・滑苔を伴う膨らんだ淡白舌、および力強い遅・沈脈を特徴とする。
脾腎虛寒證		2.9.126	spleen-kidney yang deficiency pattern/syndrome	a pattern/syndrome attributed to insufficient yang qi of the spleen and the kidney with endogenous cold, marked by bright pale complexion, aversion to cold with cold limbs, coldness and pain in the loins and lower abdomen, chronic diarrhea, or edema with inhibited urination, pale enlarged tongue with white slippery coating, and slow, sunken and forceless pulse	脾・腎の陽気の不足とともに内寒により生じる証で、面色晄白、四肢の冷えを伴う悪寒、腰部および下腹の冷えおよび疼痛、久泄、または小便不利を伴う水腫、白・滑苔を伴う膨らんだ淡白舌、および力強い遅・沈脈を特徴とする。
各科辨證		**2.10.0**	**Various Pattern Identification/ Syndorome Differentiation**		
衝任不固證		2.10.1	pattern/syndrome of insecurity of thoroughfare and conception vessels	a pattern/syndrome marked by continuous dribbling of menstrual discharge, profuse uterine bleeding or liability to abortion	月経の持続性滴下、大量の子宮出血または胎動不安を特徴とする証。
衝任失調證		2.10.2	pattern/syndrome of disharmony of thoroughfare and conception vessels	a pattern/syndrome marked by irregular menstruation and lower abdominal distention and pain	月経不調および下腹部の膨満感および疼痛を特徴とする証。
寒凝胞宮證		2.10.3	pattern/syndrome of cold congealing in the uterus	a pattern/syndrome marked by cold and pain in the lower abdomen, dysmenorrhea or delayed periods with dark menstrual discharge, white tongue coating and sunken tight pulse	下腹部の冷えおよび疼痛、暗黒色の月経を伴う月経困難症または月経後期、白苔および沈・緊脈を特徴とする証。
瘀阻胞宮證		2.10.4	pattern/syndrome of (blood) stasis obstructing the uterus	a pattern/syndrome marked by stabbing lower abdominal pain, fixed in location and tender to touch, or accompanied by mass formation, or late periods with scanty discharge of dark purple blood and clots, or cessation of menstruation, or abnormal uterine bleeding, dark purple tongue or purple spots on the tongue, and string-like choppy pulse	部位が固定された下腹部の刺痛および圧痛、またはこれに伴う腫瘤形成、または月経後期に少量の黒紫色の血および血餅の排出を伴う、または月経停止、または異常子宮出血、黒紫舌または紫斑のある舌、および不規則に変わる弦脈を特徴とする証。
胞宮虛寒證		2.10.5	uterine deficiency cold pattern/syndrome	a pattern/syndrome attributed to deficiency of kidney yang that fails to warm the uterus, and manifested by aversion to cold with cold limbs, aching and coldness of the lumbus and knees, dull pain in the lower abdomen which is alleviated by warmth and pressure, pale-colored and thin menstrual flow, or clear thin leukorrhea, or infertility, or abortion, accompanied by pale complexion, pale tongue with white coating and sunken weak pulse	腎陽の虚により胞を温められなくなることに起因する証で、四肢の冷えを伴う悪寒、腰部および膝の酸痛および冷え、温め圧迫すると緩和する下腹部の隠痛、色が薄く希薄な月経、または透明で希薄な帯下、または不孕、または流産、ならびに面色蒼白、白苔を伴う淡白舌および沈・弱脈が発現する。
胞宮濕熱證		2.10.6	uterine dampness-heat pattern/ syndrome	a pattern/syndrome marked by large amount of yellowish leukorrhea, thick, fetid, associated with pudental itching and erosion, reddened tongue with yellow slimy coating and rapid slippery pulse	外陰部の掻痒およびびらんに伴う大量の粘稠な悪臭のある黄帯、黄・膩苔を伴う紅舌および数・滑脈を特徴とする証。
胞宮積熱證		2.10.7	pattern/syndrome of accumulated heat in the uterus	a pattern/syndrome marked by scorching pain in the lower abdomen, early periods and large amount of bright red menstrual discharge, or yellowish thick and fetid leukorrhea, reddened tongue with yellow coating and rapid pulse	下腹部の灼痛、月経周期の短縮および大量の鮮紅色の月経、または粘稠な悪臭のある黄帯、黄苔を伴う紅舌および数脈を特徴とする証。
濕熱阻滯精室證		2.10.8	pattern/syndrome of dampness-heat obstructing the essence chamber	a pattern/syndrome marked by heat and pain in the perineum, scrotal itching and erosion, seminal emission or pus in the seminal fluid, difficult and painful urination, reddened tongue with yellow slimy coating and rapid slippery pulse	会陰部の熱感および疼痛、陰嚢の掻痒およびびらん、遺精または膿精液、排尿困難または有痛性排尿、黄・膩苔を伴う紅舌および数・滑脈を特徴とする証。
痰阻精室證		2.10.9	pattern/syndrome of phlegm obstructing the essence chamber	a pattern/syndrome marked by impotence, thin seminal fluid, lack of libido, obesity, lack of strength, pale tongue with white slimy coating and slippery pulse	陽痿、希薄な精液、性欲減退、肥満、乏力、白・膩苔を伴う淡白舌および滑脈を特徴とする証。
瘀阻精室證		2.10.10	pattern/syndrome of (blood) stasis obstructing the essence chamber	a pattern/syndrome marked by stabbing pain that refuses pressure in the perineal region with fixed location or mass formation or oligospermia and impotence or painful ejaculation or hematospermia	部位が固定された会陰部の刺痛（圧迫されるのを嫌がる）または腫瘤形成、または精子過少症および陽痿または有痛性射精または血精液症を特徴とする証。
風中經絡證		2.10.11	pattern/syndrome of wind striking the meridians and collaterals	a pattern/syndrome marked by numbness, itching or sudden onset of deviation of eye and mouth	麻木、掻痒または口眼喎斜の卒発を特徴とする証。
風寒襲絡證		2.10.12	pattern/syndrome of wind-cold assailing the collaterals	a pattern/syndrome marked by aversion to cold, pain and cold feeling of the limbs with contracture or numbness and purple or pale discoloration of the skin, white tongue coating and string-like tight pulse	悪寒、拘急または麻木を伴う四肢の疼痛および冷感、および紫色または蒼白色の皮膚、白苔および弦・緊脈を特徴とする証。

検索表示（原表示の複数語を分離）	IST原表示	Code	Term	Definition/Description	定義/記述
風寒阻絡證		2.10.13	pattern/syndrome of wind-cold obstructing the collaterals	a pattern/syndrome attributed to impeded blood flow in superficial meridians/channels and collaterals/ networks by pathogenic cold, and manifested by aversion to cold, coldness, pain, contracture or numbness of limbs with dark purple or pale skin, pale tongue coating and string-like tight pulse	寒邪により表層の経絡（浮絡）の血の流れが妨げられることにより生じる証で、悪寒、冷え、疼痛、四肢の拘急または麻木、および黒紫色または蒼白色の皮膚、淡白苔および弦・緊脈が発現する。
風勝行痹證		2.10.14	pattern/syndrome of wind-prevailing migratory arthralgia	a pattern/syndrome attributed to stagnation of pathogenic wind-cold-dampness (with predominance of wind) in sinews, bones and joints, and manifested by wandering arthralgia with inhibited movement of the joint, thin white tongue coating and floating pulse	筋、骨、関節における風寒湿邪（風が優勢）のうっ滞により生じる証で、関節の遊走痛のために関節運動が阻害され、薄・白苔および浮脈が発現する。
寒勝痛痹證		2.10.15	pattern/syndrome of cold-prevailing agonizing arthralgia	a pattern/syndrome attributed to stagnation of pathogenic wind-cold-dampness (with predominance of cold) in sinews, bones and joints, and manifested by joint cold pain with fixed location, white tongue coating and string-like tight pulse, the same as the pattern/syndrome of cold-prevailing painful arthralgia	筋、骨、関節における風寒湿邪（寒が優勢）のうっ滞により生じる証で、部位が固定された関節の冷痛、白苔および弦・緊脈が発現する。pattern/syndrome of cold-prevailing painful arthralgiaと同義。
濕勝著痹證		2.10.16	pattern/syndrome of dampness-prevailing fixed arthralgia	a pattern/syndrome attributed to stagnation of pathogenic wind-cold-dampness (with predominance of dampness) in sinews, bones and joints, and manifested by pain, feeling of heaviness and swelling in the joint with fixed location, local skin numbness, white slimy tongue coating, and slippery or soggy pulse	筋、骨、関節における風寒湿邪（湿が優勢）のうっ滞により生じる証で、部位が固定された関節の疼痛、重い感じおよび腫脹、局所の肌膚麻木、白・膩苔、および滑脈または濡脈が発現する。
熱邪阻痹證		2.10.17	pattern/syndrome of heat-obstructing arthralgia	a pattern/syndrome attributed to stagnation of pathogenic wind-dampness-heat (with predominance of heat) in sinews, bones and joints, and manifested by joint pain with local scorching hotness, redness and swelling, yellow tongue coating and rapid slippery pulse	筋、骨、関節における風寒湿邪（熱が優勢）のうっ滞により生じる証で、局所の灼熱感、発赤および腫脹を伴う関節痛、黄苔および数・滑脈が発現する。
外傷瘀滯證		2.10.18	pattern/syndrome of stasis and stagnation due to traumatic injury	a pattern/syndrome attributed to traumatic injury causing local stagnation of qi and blood, manifested by local ecchymosis, pain and tenderness	気および血の局所うっ滞を招く外傷に起因する証で、局所の斑状出血、疼痛および圧痛が発現する。
損傷筋骨證		2.10.19	pattern/syndrome of damage to sinew and bone	a pattern/syndrome attributed to traumatic injury causing damage to the tendons and bones, marked by local swelling, pain and impaired movement	腱および骨の損傷を招く外傷に起因する証で、局所の腫脹、疼痛および運動障害を特徴とする。
風寒犯頭證		2.10.20	pattern/syndrome of wind-cold invading the head	a pattern/syndrome marked by headache with pain of the nape and back, aggravated by wind and cold, thin white tongue coating and floating tight pulse	風・寒により悪化する項部および背部の疼痛を伴う頭痛、薄・白苔および浮・緊脈を特徴とする証。
風熱犯頭證		2.10.21	pattern/syndrome wind-heat invading the head	a pattern/syndrome marked by headache with a distention sensation, fever or aversion to wind, thirst, flushing of face, reddened tongue tip and edges, thin and yellowish tongue coating, and rapid floating pulse	膨張感を伴う頭痛、発熱または悪風、口渇、顔面潮紅、紅色の舌尖および舌辺、薄・黄苔、および数・浮脈を特徴とする証。
風濕犯頭證		2.10.22	pattern/syndrome of wind-dampness invading the head	a pattern/syndrome marked by headache as if the head were tightly swathed, slight aversion to wind and cold, heaviness feeling of the body and limbs, stuffiness sensation in the chest, anorexia, white slippery tongue coating and soggy pulse	頭に何かをきつく巻き付けられたような頭痛、軽度の悪風および悪寒、身体および四肢が重い感じ、胸部が詰まった感じ、厭食、白・滑苔および濡脈を特徴とする証。
瘀血犯頭證		2.10.23	pattern/syndrome of static blood invading the head	a pattern/syndrome arising when static blood obstructs the vessels of the head due to traumatic injury, manifested by persistent sharp pain with fixed location, dizziness, forgetfulness, gloomy complexion, purple tongue or purple spots on the tongue, and string-like rough pulse	外傷により瘀血が頭部の脈を閉塞することにより生じる証で、部位が固定された持続性の鋭痛、めまい、健忘症、陰うつな顔、紫舌または紫斑のある舌、および弦・渋脈が発現する。
痰濁犯頭證		2.10.24	pattern/syndrome of phlegm turbidity invading the head	a pattern/syndrome marked by headache associated with mental clouding, tinnitus, impaired hearing, blurred vision, stuffiness and a feeling of pressure in the chest and epigastrium, vomiting of phlegm/ mucus, white slimy tongue coating and string-like slippery pulse	意識混濁に関連する頭痛、耳鳴、聴覚障害、目昏、胸部および心窩部の詰まった感じおよび圧迫感、痰／粘液の喀（吐）出、白膩苔および弦・滑脈を特徴とする証。
肝火犯頭證		2.10.25	pattern/syndrome of liver fire invading the head	a pattern/syndrome marked by severe headache associated with flushing face, red eyes, irritability, irascibility, bitter taste in the mouth, reddened tongue with yellow coating and rapid string-like pulse	顔面潮紅に関連する重度の頭痛、目の充血、いらいらする、怒りっぽい、口苦、黄苔を伴う紅舌および数・弦脈を特徴とする証。
風火攻目證		2.10.26	pattern/syndrome of wind-fire attacking the eyes	a pattern/syndrome marked by reddened, swollen and painful eye and lacrimination associated with fever, aversion to wind and floating rapid pulse	発熱に伴う目の充血、腫脹、疼痛および流涙、悪風および浮・数脈を特徴とする証。
風濕凌目證		2.10.27	pattern/syndrome of wind-dampness insulting the eyes	a pattern/syndrome marked by swelling of the eyelid, reddened and itchy eye with lacrimination, and photophobia	眼瞼腫脹、流涙を伴う目の充血および掻痒、および羞明を特徴とする証。
外傷目絡證		2.10.28	pattern/syndrome of traumatic injury of ocular vessel	a pattern/syndrome marked by swollen and painful eyelid dark purple in color, or the white of the eye turning into red or dark purple, or pain of the eye with fundus bleeding	黒紫色の変色を伴う眼瞼の腫脹または疼痛、または白睛の赤色（充血）または黒紫色の変色、または眼底出血を伴う目痛を特徴とする証。
蟲積化疳證		2.10.29	pattern/syndrome of worm accumulation transforming into malnutrition	a pattern/syndrome that arises when intestinal parasites deprive the eyes of nourishment, and that manifests in impaired vision, night blindness, dryness of the eyes and photophobia	腸の寄生虫により目の滋養が枯渇するときに生じる証で、視覚障害、雀目、目の乾燥および羞明が発現する。
肝火燔耳證		2.10.30	pattern/syndrome of liver fire blazing the ear	a pattern/syndrome marked by painful distension in the ear, congestion or perforation of tympanic membrane, or discharge of pus and blood from the meatus, associated with tinnitus, dizziness, bitter taste in the mouth, reddened face and eyes, vexation, irascibility, reddened tongue with yellow coating and rapid string-like pulse	疼痛を伴う耳張、鼓膜の閉塞または穿孔、または耳道からの膿および血の排出、これに伴う耳鳴、めまい、口苦、顔面潮紅および目の充血、心煩、怒りっぽい、黄苔を伴う紅舌および数・弦脈を特徴とする証。
風熱犯耳證		2.10.31	pattern/syndrome of wind-heat invading the ear	a pattern/syndrome marked by distension and obstruction in the ear, congestion of the tympanic membrane or a feeling of pressure in the ear, associated with tinnitus, headache, fever with slight aversion to wind and cold, thin yellow tongue coating and rapid floating pulse	耳脹および耳の閉塞、鼓膜閉塞または耳の圧迫感、これに関連する耳鳴、頭痛、軽度の悪風および悪感を伴う発熱、薄・黄苔および数・浮脈を特徴とする証。

検索表示（原表示の複数語を分離）	IST原表示	Code	Term	Definition/Description	定義/記述
濕熱犯耳證		2.10.32	pattern/syndrome of dampness-heat invading the ear	a pattern/syndrome marked by redness, swelling, pain, erosion, exudation, incrustation of the meatus or auricle, or discharge of thick yellow pus from the ear, or distension of the ear with tinnitus, yellow slimy tongue coating and rapid slippery pulse	耳道または耳介の発赤、腫脹、疼痛、びらん、浸出液、痂皮形成、または耳からの粘稠な黄色膿の排出、または耳鳴を伴う耳脹、黄・膩苔および数・滑脈を特徴とする証。
痰濕犯耳證		2.10.33	pattern/syndrome of phlegm-dampness attacking the ear	a pattern/syndrome marked by distension and pressure in the ear associated with dizziness, tinnitus, heaviness feeling of the head and nausea; or thin purulent discharge from the ear, impaired hearing and visible fluid horizon through the drum membrane; slippery slimy tongue coating and string-like slippery pulse	めまいに関連する耳脹および耳の圧迫、耳鳴、頭重感および悪心、または耳からの希薄な膿性分泌液の排出、聴覚障害および鼓膜を通して肉眼で認められる液体、滑・膩苔および弦・滑脈を特徴とする証。
氣虛耳竅失充證		2.10.34	pattern/syndrome of qi deficiency with hearing loss	a pattern/syndrome attributed to weakened healthy qi with the clear yang qi failing in ascending, and marked by tinnitus like the sound of cicadas, impairment of hearing, associated with dizziness, lack of strength, shortness of breath, reluctance to speak, pale tongue and weak pulse, the same as the pattern/syndrome of hearing loss due to qi deficiency	正気が弱まり、清明な陽気が上行できないことにより生じる証で、蝉が鳴いているような耳鳴、聴覚障害、これに伴うめまい、乏力、短気、話すのが億劫、淡白舌および弱脈を特徴とする。pattern/syndrome of hearing loss due to qi deficiencyと同義。
風寒犯鼻證		2.10.35	pattern/syndrome of wind-cold invading the nose	a pattern/syndrome marked by stuffy runny nose or nasal itching with sneezing, pale mucous membrane of the nose with thin clear secretion, associated with aversion to wind and cold, thin white tongue coating and floating tight pulse	水のような鼻汁による鼻づまりまたは鼻の掻痒と噴嚏、蒼白な鼻粘膜および希薄な透明の分泌液、これに伴う悪風および悪寒、薄・舌苔および浮・緊脈を特徴とする証。
風熱犯鼻證		2.10.36	pattern/syndrome of wind-heat invading the nose	a pattern/syndrome marked by nasal obstruction and discharge, swollen conchae and congested mucous membrane with thick secretion, impairment of the sense of smell, associated with fever and slight aversion to wind and cold, thin yellow tongue coating and floating rapid pulse	鼻の閉塞および鼻汁、粘稠な分泌液を伴う鼻甲介の腫脹および粘膜充血、嗅覚障害、ならびに発熱および軽度の悪風および悪寒、薄・黄苔および浮・数脈を特徴とする証。
氣虛鼻竅失充證		2.10.37	pattern/syndrome of qi deficiency with loss of smell	a pattern/syndrome marked by nasal obstruction with clear discharge and frequent sneezing, and pale swollen conchae, associated with dizziness, lack of strength, shortness of breath, reluctance to speak, pale tongue and weak pulse, the same as the pattern/syndrome of loss of smell due to qi deficiency	透明な鼻汁を伴う鼻閉塞および頻繁な噴嚏、および蒼白な腫脹した鼻甲介、およびめまい、乏力、短気、話すのが億劫、淡白舌および弱脈を特徴とする証。pattern/syndrome of loss of smell due to qi deficiensyと同義。
陰虛鼻竅失濡證		2.10.38	pattern/syndrome of yin deficiency with dryness of the nose	a pattern/syndrome marked by enlarged nasal cavity with feeling of scorching hotness, dried nasal membrane, atrophied conchae and discharge of a little mucus with crusts or blood, or even epistaxis, dry throat and mouth, tida! fever with malar flush, dry tongue with dry coating and rapid fine pulse	灼熱感を伴う鼻腔拡大、鼻粘膜乾燥、鼻甲介萎縮および痂皮または血の混じった少量の粘液排出、または鼻血、喉および口の乾燥、頬部潮紅を伴う潮熱、燥苔を伴う舌乾および数・細脈を特徴とする証。
風寒襲喉證		2.10.39	pattern/syndrome of wind-cold assailing the throat	a pattern/syndrome marked by painful, itchy and slightly swollen throat dark red in color associated with hoarseness, fever, aversion to cold, absence of sweat, stuffy nose and floating tight pulse	疼痛、掻痒および軽度の腫脹が認められ、暗赤色に変色した咽喉およびこれに伴う嗄声、発熱、悪寒、無汗、鼻づまりおよび浮・緊脈を特徴とする証。
風熱侵喉證		2.10.40	pattern/syndrome of wind-heat invading the throat	a pattern/syndrome marked by painful swollen throat or enlarged congested tonsils, with discomfort in swallowing and hoarseness of voice, fever, slight aversion to wind and cold, mild thirst, thin yellow tongue coating and rapid floating pulse	喉の疼痛および腫脹または扁桃腺の肥大およびうっ血、ならびに嚥下時の不快感および嘶嗄、発熱、軽度の悪風および悪寒、軽度の口渇、薄・黄苔および数・浮脈を特徴とする証。
毒熱攻喉證		2.10.41	pattern/syndrome of toxic heat attacking the throat	a pattern/syndrome marked by redness, swelling and pain of the throat with difficulty in swallowing or even ulceration and suppuration with fetid mouth odor, intense fever, thirst, reddened tongue with yellow coating and rapid forceful pulse	喉の発赤、腫脹および疼痛に伴う嚥下困難、または口臭を伴う潰瘍および化膿、激しい発熱、口渇、黄苔を伴う紅舌および力強い数脈を特徴とする証。
氣滯痰凝咽喉證		2.10.42	pattern/syndrome of qi stagnating and phlegm congealing in the throat	a pattern/syndrome marked by depressed mood, discomfort sensation of a foreign body present in the throat, swelling of the pharyngeal mucous membrane, slimy tongue coating and string-like slippery pulse	抑うつ気分、喉の異物による不快感、咽頭粘膜の腫脹、膩苔および弦・滑脈を特徴とする証。
胃火燔齦證		2.10.43	pattern/syndrome of stomach fire blazing the gums	a pattern/syndrome marked by red, swollen and painful gums with exudation of blood and pus between the teeth, thirst, fetid mouth odor, constipation, reddened tongue with yellow coating and rapid pulse	歯間からの血および膿性浸出液を伴う歯肉の発赤、腫脹および疼痛、口渇、口臭、便秘、黄苔を伴う紅舌および数脈を特徴とする証。
濕熱蒸齒證		2.10.44	pattern/syndrome of dampness-heat steaming the teeth	a pattern/syndrome marked by toothache or dental caries with pain aggravated by heat, sweetness and sourness, or red swollen gums, fetid mouth odor, reddened tongue with yellow slimy coating and rapid slippery pulse	熱、甘味、酸味により疼痛が悪化する歯の酸痛または齲歯、または歯肉の発赤または腫脹、口臭、黄・膩苔を伴う紅舌および数・滑脈を特徴とする証。
虛火灼齦證		2.10.45	pattern/syndrome of deficiency fire scorching the gums	a pattern/syndrome marked by dried and atrophied gums with exposure of the dental roots, looseness of the teeth with dull pain, associated with vexing heat in the chest, palms and soles, flushed cheeks, reddened tongue with little moisture and rapid fine pulse	歯根の露出を伴う歯肉の乾燥および歯挺、隠痛を伴う歯のゆるみ、これに関連する胸部、手掌および足底の煩熱、頬部潮紅、ほとんど潤いがない紅舌および数・細脈を特徴とする証。
陰虛咽喉失濡證		2.10.46	pattern/syndrome of yin deficiency with dryness of the throat	a pattern/syndrome marked by a scorching feeling in the throat with itching, mild pain and hoarseness, or sensation of presence of a foreign body in the throat, dry and slightly reddened pharynx or local ulceration, reddened tongue with little moisture and rapid fine pulse	掻痒、軽度の疼痛および嘶嗄を伴う喉の灼熱感、または喉の異物感、咽頭の乾燥および軽度の発赤または局所潰瘍、潤いのほとんどない紅舌および数・細脈を特徴とする証。
毒火攻唇證		2.10.47	pattern/syndrome of toxic fire attacking the lips	a pattern/syndrome marked by a red swollen lip with severe pain or local ulceration with discharge of fetid pus, intense fever, thirst, reddened tongue with yellow coating and rapid pulse	重度の疼痛を伴う唇の発赤および腫脹または悪臭のある膿性分泌物を伴う局所潰瘍、激しい発熱、口渇、黄苔を伴う紅舌および数脈を特徴とする証。
濕熱蒸口證		2.10.48	pattern/syndrome of dampness-heat steaming the mouth	a pattern/syndrome marked by redness, swelling and pain of the mucous membrane of the mouth, or ulceration in the oral cavity with discharge of fetid pus, or reddened, swollen and ulcerated lip with inflamed angular rhagades, reddened tongue with yellow slimy coating and rapid soggy pulse	口腔粘膜の発赤、腫脹および疼痛、または悪臭のある膿性分泌物を伴う口腔潰瘍、または唇の発赤、腫脹および潰瘍ならびに炎症を伴う口角の亀裂、黄・膩苔を伴う紅舌および数・濡脈を特徴とする証。

検索表示（原表示の複数語を分離）	IST原表示	Code	Term	Definition/Description	定義/記述
濕熱蒸舌證		2.10.49	pattern/syndrome of dampness-heat steaming the tongue	a pattern/syndrome marked by a reddened, swollen and painful tongue, even with festering ulceration and yellow slimy tongue coating	化膿した潰瘍を伴う舌の発赤、腫脹および疼痛、および黄・膩苔を特徴とする証。
熱毒攻舌證		2.10.50	pattern/syndrome of heat toxin attacking the tongue	a pattern/syndrome marked by redness, swelling and pain of the tongue or a part of the tongue with impaired lingual movement, fever, thirst and rapid forceful pulse	舌または舌の一部の発赤、腫脹および疼痛とこれに伴う舌の運動障害、発熱、口渇および力強い数脈を特徴とする証。
血瘀舌下證		2.10.51	sublingual blood stasis pattern/ syndrome	a pattern/syndrome marked by the development of a sublingual dark purple mass, purple tongue or purple spots on the tongue, and impaired motility of the tongue	黒紫色の舌下腫瘤の発現、紫舌または紫斑のある舌、および舌の運動障害を特徴とする証。
六經辨證		**2.11.0**	**Six-Meridian Pattern Identification/ Syndrome Differentiation**		
六經辨證		2.11.1	six-meridian pattern identification/ syndrome differentiation	categorization of patterns/syndromes according to the theory of the six meridians/channels, applied to the diagnosis of acute febrile diseases at different stages, but also useful for the pattern identification/syndrome differentiation of other diseases	6つの経絡の理論により証を分類することで、異なる段階の急性熱病の診断に用いられるが、その他の疾病の弁証論治にも役立つ。
太陽病證	太陽病證; 太陽病	2.11.2	greater yang disease pattern/syndrome	a general term for greater yang meridian pattern/ syndrome and greater yang bowel pattern/syndrome, occurring at the onset of the febrile disease, chiefly manifested by headache, painful stiff nape, aversion to cold and floating pulse, also called the greater yang disease	熱病の発症時に生じる太陽の経絡の証および太陽の腑の証の一般用語で、主に頭項強痛、悪寒および浮脈が発現する。greater yang diseaseとも呼ばれる。
太陽病	太陽病證; 太陽病	2.11.2	greater yang disease pattern/syndrome	a general term for greater yang meridian pattern/ syndrome and greater yang bowel pattern/syndrome, occurring at the onset of the febrile disease, chiefly manifested by headache, painful stiff nape, aversion to cold and floating pulse, also called the greater yang disease	熱病の発症時に生じる太陽の経絡の証および太陽の腑の証の一般用語で、主に頭項強痛、悪寒および浮脈が発現する。greater yang diseaseとも呼ばれる。
太陽經證		2.11.3	greater yang meridian pattern/syndrome	one of the patterns/syndromes of the six meridians due to attack of pathogenic wind-cold on the greater yang meridian of the body surface, usually seen in the initial stage of the contraction, marked by chills and fever, headache and painful stiff nape, and floating pulse	風寒邪が体表の太陽の経絡を攻撃することにより生じる六経の証の1つで、通常、感受の初期段階で認められる。寒気および発熱、頭項強痛および浮脈を特徴とする。
太陽腑證		2.11.4	greater yang bowel pattern/syndrome	a pattern/syndrome in which the urinary bladder (greater yang bowel) is attacked by the pathogen in an unrelieved greater yang meridian	長引く太陽経の病邪が膀胱（太陽の腑）を攻撃することにより生じる証。
太陽傷寒證	太陽傷寒證; 太陽傷寒	2.11.5	greater yang cold damage pattern/ syndrome	a pattern/syndrome caused by pathogenic cold attacking the greater yang meridian, chiefly manifested by fever, chills, absence of sweat and floating pulse, also called the greater yang cold damage	寒邪が太陽の経絡を攻撃することにより生じる証で、主に発熱、寒気、無汗および浮脈が発現する。greater yang cold damageとも呼ばれる。
太陽傷寒	太陽傷寒證; 太陽傷寒	2.11.5	greater yang cold damage pattern/ syndrome	a pattern/syndrome caused by pathogenic cold attacking the greater yang meridian, chiefly manifested by fever, chills, absence of sweat and floating pulse, also called the greater yang cold damage	寒邪が太陽の経絡を攻撃することにより生じる証で、主に発熱、寒気、無汗および浮脈が発現する。greater yang cold damageとも呼ばれる。
太陽蓄水證	太陽蓄水證; 太陽蓄水	2.11.6	greater yang water-retention pattern/ syndrome	a type of greater yang bowel pattern/syndrome marked by disordered water metabolism, chiefly manifested by impaired urination and thirst but vomiting immediately after the ingestion of water, also called the greater yang water-retention	水分代謝障害を特徴とする太陽の腑の証の一種で、主に排尿障害および口渇が発現するが、水分摂取直後に嘔吐は発現しない。greater yang water-retentionとも呼ばれる。
太陽蓄水	太陽蓄水證; 太陽蓄水	2.11.6	greater yang water-retention pattern/ syndrome	a type of greater yang bowel pattern/syndrome marked by disordered water metabolism, chiefly manifested by impaired urination and thirst but vomiting immediately after the ingestion of water, also called the greater yang water-retention	水分代謝障害を特徴とする太陽の腑の証の一種で、主に排尿障害および口渇が発現するが、水分摂取直後に嘔吐は発現しない。greater yang water-retentionとも呼ばれる。
陽明病證	陽明病證; 陽明病	2.11.7	yang brightness disease pattern/ syndrome	a pattern/syndrome marked by exuberant yang and dryness-heat in the stomach and intestines occurring in the course of an externally contracted disease, manifested by abdominal distention with constipation, tidal fever and deep and replete pulse, also called the yang brightness disease	盛んな陽および胃腸の燥熱を特徴とし、体外で感受した疾病の過程で生じる証で、便秘を伴う腹部膨満、潮熱および沈・実脈が発現する。yang brightness diseaseとも呼ばれる。
陽明病	陽明病證; 陽明病	2.11.7	yang brightness disease pattern/ syndrome	a pattern/syndrome marked by exuberant yang and dryness-heat in the stomach and intestines occurring in the course of an externally contracted disease, manifested by abdominal distention with constipation, tidal fever and deep and replete pulse, also called the yang brightness disease	盛んな陽および胃腸の燥熱を特徴とし、体外で感受した疾病の過程で生じる証で、便秘を伴う腹部膨満、潮熱および沈・実脈が発現する。yang brightness diseaseとも呼ばれる。
陽明經證		2.11.8	yang brightness meridian pattern/ syndrome	a pattern/syndrome caused by exuberant pathogenic heat flooding in the yang brightness meridian and spreading over the body but not yet inducing constipation, characteristically manifested by high fever without chills, profuse sweating, strong thirst and large surging pulse	盛んな邪熱が陽明の経絡にあふれ出て、体全体に広がることにより生じるが、まだ便秘をもたらしていない証で、寒気のない壮熱、多汗、強い口渇および大・洪脈が特徴的に発現する。
陽明腑證		2.11.9	yang brightness bowel pattern/ syndrome	a pattern/syndrome caused by accumulation of pathogenic heat in the stomach and large intestine, manifested by tidal fever, abdominal distension, fullness and pain, constipation, agitation or even delirium	熱邪が胃および大腸に蓄積することにより生じる証で、潮熱、腹部の脹満および疼痛、便秘、煩躁または譫妄が発現する。
少陽病證	少陽病證; 少陽病	2.11.10	lesser yang disease pattern/syndrome	a pattern/syndrome in which the pathogen exists between the exterior and interior of the body, marked by alternate fever and chills, fullness and choking feeling in the chest and hypochondriac region, dry throat and string-like pulse, also called the lesser yang disease	病邪が身体の表裏の間に存在する証で、寒熱往来、胸部および下肋部の充満感および息苦しい感じ、喉の乾燥および弦脈を特徴とする。lesser yang diseaseとも呼ばれる。
少陽病	少陽病證; 少陽病	2.11.10	lesser yang disease pattern/syndrome	a pattern/syndrome in which the pathogen exists between the exterior and interior of the body, marked by alternate fever and chills, fullness and choking feeling in the chest and hypochondriac region, dry throat and string-like pulse, also called the lesser yang disease	病邪が身体の表裏の間に存在する証で、寒熱往来、胸部および下肋部の充満感および息苦しい感じ、喉の乾燥および弦脈を特徴とする。lesser yang diseaseとも呼ばれる。

検索表示（原表示の複数語を分離）	IST原表示	Code	Term	Definition/Description	定義/記述
少陽經證		2.11.11	lesser yang meridian pattern/syndrome	a lesser yang pattern/syndrome involving the gallbladder meridian, marked by alternating chills and fever, chest and hypochondriac pain and fullness, no desire for food and drink, irritable disposition, desire to vomit, bitter taste in the mouth, dry throat, dizzy vision, white tongue coating and string-like pulse	胆の経絡を障害する少陽の証で、寒熱往来、胸部および下肋部の疼痛および充満感、飲食したがらない、いらいら傾向、嘔吐したがる、口苦、喉の乾燥、目眩、白舌苔および弦脈を特徴とする。
少陽腑證		2.11.12	lesser yang bowel pattern/syndrome	aa lesser yang pattern/syndrome associated with heat binding in the gastrointestinal tract, marked by alternating chills and fever, chest and hypochondriac pain and fullness, incessant vomiting, epigastric cramp, irritable disposition, constipation or heat bind with circumfluence, reddened tongue with yellow dry coating and forceful string-like pulse	熱が胃腸管と結合する少陽の証で、寒熱往来、胸部および下肋部の疼痛および充満感、絶え間ない嘔吐、心窩部のけいれん、いらいら傾向、便秘または環流性の熱結、黄・燥苔を伴う紅舌および力強い弦脈を特徴とする。
太陰病證		2.11.13	greater yin disease pattern/syndrome	a pattern/syndrome characterized by decline of spleen yang with production of cold-dampness, and manifested by anorexia, vomiting, abdominal fullness and dull pain, diarrhea and weak pulse, also called the greater yin disease	脾陽の低下と寒湿の生成を特徴とする証で、厭食、嘔吐、腹部の充満感および隠痛、泄瀉および弱脈が発現する。greater yin diseaseとも呼ばれる。
太陰病		2.11.13	greater yin disease pattern/syndrome	a pattern/syndrome characterized by decline of spleen yang with production of cold-dampness, and manifested by anorexia, vomiting, abdominal fullness and dull pain, diarrhea and weak pulse, also called the greater yin disease	脾陽の低下と寒湿の生成を特徴とする証で、厭食、嘔吐、腹部の充満感および隠痛、泄瀉および弱脈が発現する。greater yin diseaseとも呼ばれる。
太陰中風證		2.11.14	greater yin wind stroke pattern/syndrome	a pattern/syndrome of greater yin deficiency cold combined with exterior wind-cold, marked by aversion to wind, fever, sweating, abdominal fullness and pain alleviated by warmth and pressure, and watery diarrhea	太陰の虚寒と表の風寒が組み合わさった証で、悪風、発熱、発汗、腹部充満感および温め圧迫すると緩和する疼痛、および水瀉を特徴とする。
少陰病證	少陰病證; 少陰病	2.11.15	lesser yin disease pattern/ syndrome	a pattern/syndrome occurring at the late stage of an externally contracted disease, marked by deficiency cold of the heart and kidney, and manifested by aversion to cold, listlessness, irritable disposition, insomnia, cold limbs, diarrhea with undigested food and fine pulse, also called the lesser yin disease	体外で感受した疾病の後期で生じ、心・腎の寒虚を特徴とする証で、悪寒、無気力、いらいら傾向、失眠、四肢の冷え、未消化食物の泄瀉および細脈が発現する。lesser yin diseaseとも呼ばれる。
少陰病	少陰病證; 少陰病	2.11.15	lesser yin disease pattern/ syndrome	a pattern/syndrome occurring at the late stage of an externally contracted disease, marked by deficiency cold of the heart and kidney, and manifested by aversion to cold, listlessness, irritable disposition, insomnia, cold limbs, diarrhea with undigested food and fine pulse, also called the lesser yin disease	体外で感受した疾病の後期で生じ、心・腎の寒虚を特徴とする証で、悪寒、無気力、いらいら傾向、失眠、四肢の冷え、未消化食物の泄瀉および細脈が発現する。lesser yin diseaseとも呼ばれる。
少陰表寒證		2.11.16	lesser yin exterior cold pattern/syndrome	a pattern/syndrome arising when pathogenic cold invades the lesser yin and then the greater yang concurrently in a patient with yang deficiency constitution, and manifested by fever, aversion to cold, headache, absence of sweating, cold limbs, listlessness, white tongue coating and sunken pulse	陽虚の体質が認められる患者において、寒邪が少陰に侵入し、同時に太陽にも侵入するときに生じる証で、発熱、悪寒、頭痛、無汗、四肢の冷え、無気力、白苔および沈脈が発現する。
少陰寒化證		2.11.17	lesser yin cold transformation pattern/syndrome	a lesser yin pattern/syndrome with cold transformation of the pathogen which invades the heart and kidney, usually manifested as aversion to cold, cold limbs, sleepiness, lienteric diarrhea, pale tongue and sunken faint pulse	心・腎に侵入した病邪が寒に転換する少陰の証で、通常、悪寒、四肢の冷え、眠気、未消化物を含む泄瀉、淡白舌および沈・微脈が発現する。
少陰熱化證		2.11.18	lesser yin heat transformation pattern/syndrome	a lesser yin pattern/syndrome with heat transformation of the pathogen, manifested by irritable disposition, insomnia, dry mouth and throat, reddened tongue tip and fine rapid pulse	病邪が熱に転換する少陰の証で、いらいら傾向、失眠、口乾および喉の乾燥、紅舌尖および細・数脈が発現する。
厥陰病證	厥陰病證; 厥陰病	2.11.19	reverting yin disease pattern/syndrome	a pattern/syndrome occurring at the latest stage of three yin disease characterized by interweaving of cold and heat or yin and yang in a critically ill case, also called the reverting yin disease	3つの陰病の最終段階で生じ、危篤の場合に寒・熱または陰・陽が絡み合うことを特徴とする証。reverting yin diseaseとも呼ばれる。
厥陰病	厥陰病證; 厥陰病	2.11.19	reverting yin disease pattern/syndrome	a pattern/syndrome occurring at the latest stage of three yin disease characterized by interweaving of cold and heat or yin and yang in a critically ill case, also called the reverting yin disease	3つの陰病の最終段階で生じ、危篤の場合に寒・熱または陰・陽が絡み合うことを特徴とする証。reverting yin diseaseとも呼ばれる。
厥陰熱厥證		2.11.20	reverting yin heat reversal pattern/ syndrome	a pattern/syndrome occurring at the latest stage of an externally contracted disease when the pathogen prevents the extremely exuberant yang heat from reaching the exterior, and manifested by cold extremities, flushed face and congested eyes, scorching hotness of the chest and abdomen, thirst, irritable disporition,restlessness and inability to sleep, short voidings of reddish urine, constipation, reddened tongue with yellow coating and rapid slippery pulse	体外で感受した疾病の最終段階で生じ、病邪により極度に盛んな陽熱が体外に到達できないときに生じる証で、四肢の冷え、顔面潮紅および目の充血、胸部および腹部の灼熱感、口渇、いらいら傾向、落ち着きのなさおよび失眠、短時間の赤色尿の排出、便秘、黄苔を伴う紅舌および数・滑脈が発現する。
厥陰寒厥證		2.11.21	reverting yin cold reversal pattern/syndrome	a pattern/syndrome occurring at the latest stage of an externally contracted disease characterized by reversal cold of the extremities and hardly perceptible or skipping pulse	体外で感受した疾病の最終段階で生じる証で、手足厥冷およびほとんど触れない脈または促脈を特徴とする。
太陽蓄血證	太陽蓄血證; 太陽蓄血	2.11.22	greater yang blood amassment pattern/syndrome	a type of greater yang bowel pattern/syndrome in which the pathogen combines with blood and remains in the lower abdomen, marked by lower abdominal cramps or fullness with rigidity, polyuria, delirium, amnesia, dark stool, and sunken choppy or sunken bound pulse,also called the greater yang blood amassment	病邪が血に結合し、下腹にとどまる太陽の腑の証の一種で、硬直を伴う下腹部のけいれんまたは充満感、多尿、譫妄、健忘症、暗黒色の便、および不規則に変わる沈脈または沈・結脈を特徴とする。greater yang blood amassmentとも呼ばれる。
太陽蓄血	太陽蓄血證; 太陽蓄血	2.11.22	greater yang blood amassment pattern/syndrome	a type of greater yang bowel pattern/syndrome in which the pathogen combines with blood and remains in the lower abdomen, marked by lower abdominal cramps or fullness with rigidity, polyuria, delirium, amnesia, dark stool, and sunken choppy or sunken bound pulse, also called the greater yang blood amassment	病邪が血に結合し、下腹にとどまる太陽の腑の証の一種で、硬直を伴う下腹部のけいれんまたは充満感、多尿、譫妄、健忘症、暗黒色の便、および不規則に変わる沈脈または沈・結脈を特徴とする。greater yang blood amassmentとも呼ばれる。
熱入血室證		2.11.23	pattern/syndrome of heat entering blood chamber	a pattern/syndrome marked by penetration of heat into the uterus, which causes abdominal pain, menstrual disturbances, alternate fever and chills, and delirium at night	熱が胞に浸透し、これにより腹痛、月経障害、寒熱往来および夜間譫妄が引き起こされる証。

検索表示（原表示の複数語を分離）	IST原表示	Code	Term	Definition/Description	定義/記述
衛氣營血辨證		**2.12.0**	**Defense, Qi, Nutrient and Blood Pattern Identification / Syndrome Differentiation**		
衛氣營血辨證		2.12.1	defense, qi, nutrient and blood pattern identification/ syndrome differentiation	categorization of epidemic febrile diseases patterns/ syndromes according to the theory of defense, qi, nutrient and blood which indicate the stages of the clinical course with corresponding pathological changes	臨床経過の段階とこれに対応する病的変化を示す衛、気、営、血の理論による流行性熱病の証の分類。
衛分證		2.12.2	defense aspect pattern/syndrome	the initial stage of an epidemic febrile disease when only the superficial part of the defense qi is involved, marked by fever, slightly aversion to wind and cold, headache, reddened tongue tip and rapid floating pulse	衛気の障害が表層部に限定されている流行性熱病の初期段階で、発熱、軽度の悪風および悪寒、頭痛、紅舌尖および数・浮脈を特徴とする。
氣分證		2.12.3	qi aspect pattern/ syndrome	the second stage of an epidemic febrile disease showing intrusion of pathogenic heat on the yang brightness meridian or the lung, gallbladder, spleen, stomach or large intestine, marked by high fever without chills, strong thirst, flushed face, dark urine, reddened tongue with yellow coating and rapid forceful pulse	陽明の経絡、または肺、胆、脾、胃または大腸への熱邪の侵入を示す流行性熱病の第2段階で、寒気のない壮熱、強い口渇、顔面潮紅、暗黒色の尿、黄苔を伴う紅舌および力強い数脈を特徴とする。
營分證		2.12.4	nutrient aspect pattern/syndrome	serious development of an epidemic febrile disease characterized by pathogenic heat entering the nutrient aspect and disturbing the heart (mind), manifested by fever higher at night, restlessness or delirium, faint skin rashes and crimson tongue	熱邪が営分に進入し、心（精神）を障害するという特徴をもつ流行性熱病の重篤な発症のことで、夜間に高くなる熱、落ち着きのなさまたは譫妄、かすかな皮疹および絳舌が発現する。
血分證		2.12.5	blood aspect pattern/ syndrome	epidemic febrile disease at its severest stage, characterized by severe damage of yin blood, with various forms of bleeding such as hemoptysis, epistaxis, hematuria, hematochezia, in addition to high fever, coma or convulsions	陰血の重度の障害を特徴とする最も重度な段階での流行性熱病で、喀血、鼻血、尿血、便血などの種々の出血に加えて、壮熱、昏睡または抽搐が認められる。
衛氣同病證		2.12.6	pattern/syndrome of both defense-qi aspects disease	a pattern/syndrome characterized by coexistence of pathogenic heat in the defense and qi aspects, manifested by high fever, slight aversion to wind and cold, thirst, irritability, reddened tongue and rapid floating pulse	衛分と気分の両方に熱邪がみられるという特徴をもつ証で、壮熱、軽度の悪風および悪寒、口渇、いらいらする、紅舌および数・浮脈が発現する。
衛營同病證		2.12.7	pattern/syndrome of both defense-nutrient aspects disease	a pattern/syndrome of epidemic febrile disease characterized by coexistence of pathogenic heat in the defense and nutrient aspects, manifested by high fever and delirium together with chills, headache and general aching	衛分と営分の両方に熱邪がみられるという特徴をもつ流行性熱病の証で、壮熱および譫妄とともに寒気、頭痛および全身の酸痛が発現する。
氣營兩燔證		2.12.8	pattern/syndrome of dual blaze of qi-nutrient aspects	a pattern/syndrome characterized by simultaneous existence of syndromes of qi and nutrient aspects, manifested by high fever, thirst, mental irritability, delirium and barely visible skin eruption	気分と営分の症候群が同時にみられるという特徴をもつ証で、壮熱、口渇、精神的ないらいら、譫妄およびかろうじて肉眼で認められる皮疹が発現する。
氣血兩燔證		2.12.9	pattern/syndrome of dual blaze of qi-blood aspects	a pattern/syndrome characterized by coexistence of syndromes of qi and blood aspects, manifested by high fever, thirst, delirium, skin eruptions, and various bleeding symptoms	気分と血分の症候群が同時にみられるという特徴をもつ証で、壮熱、口渇、譫妄、皮疹、および種々の出血症状が発現する。
熱入血分證		2.12.10	pattern/syndrome of heat entering the blood aspect	a pattern/syndrome marked by fever, impaired consciousness, bleeding and deep crimson tongue	発熱、意識障害、出血および絳舌を特徴とする証。
熱入營血證		2.12.11	pattern/syndrome of heat entering nutrient-blood aspects	a pattern/syndrome marked by fever higher at night, vexing insomnia or impaired consciousness, barely visible skin rashes or bleeding, constipation, crimson tongue and rapid fine pulse	夜間に高くなる熱、わずらわしい失眠または意識障害、かろうじて肉眼で認められる皮疹または出血、便秘、絳舌および数・細脈を特徴とする証。
熱盛動風證		2.12.12	pattern/syndrome of exuberant heat stirring wind	a pattern/syndrome marked by high fever, coma, convulsions with rigid neck, opisthotonos, lockjaw, crimson tongue with yellow coating and rapid string-like pulse	壮熱、昏睡、頚部硬直を伴う抽搐、弓なり緊張、開口障害、黄苔を伴う絳舌および数・弦脈を特徴とする証。
熱盛動血證		2.12.13	pattern/syndrome of exuberant heat with bleeding	a pattern/syndrome marked by high fever, thirst, delirium, flushed face, red eyes, hematochezia, hematuria, epistaxis, distinct skin eruption, crimson tongue with yellow coating and rapid surging pulse	壮熱、口渇、譫妄、顔面潮紅、目の充血、便血、尿血、鼻血、明瞭な皮疹、黄苔を伴う絳舌および数・洪脈を特徴とする証。
熱入心包證	熱入心包證; 熱閉心包證	2.12.14	pattern/syndrome of heat entering the pericardium	a pattern/syndrome marked by high fever, thirst, delirium, red face, coarse breath, reddened tongue with yellow coating and slippery rapid pulse	壮熱、口渇、譫妄、顔面潮紅、荒い呼吸、黄苔を伴う紅舌および滑・数脈を特徴とする証。
熱閉心包證	熱入心包證; 熱閉心包證	2.12.14	pattern/syndrome of heat entering the pericardium	a pattern/syndrome marked by high fever, thirst, delirium, red face, coarse breath, reddened tongue with yellow coating and slippery rapid pulse	壮熱、口渇、譫妄、顔面潮紅、荒い呼吸、黄苔を伴う紅舌および滑・数脈を特徴とする証。
餘熱未淸證		2.12.15	residual heat pattern/ syndrome	a pattern/syndrome characterized by lingering of residual heat with damage to body fluids, and manifested by persistent low fever, irritable disposition, thirst, constipation, deep-colored urine, reddened tongue lacking moisture and rapid fine pulse	残留熱がなかなか消えず、体液が障害されることを特徴とする証で、持続性の微熱、いらいら傾向、口渇、便秘、暗黒色の尿、潤いのない紅舌および数・細脈が発現する。
毒壅上焦證		2.12.16	pattern/syndrome of toxin congesting the upper energizer	a pattern/syndrome marked by chills and fever at the onset, with reddened and swollen head and face, itching and sore throat, and then by high fever, thirst, hot and swollen head and face, skin eruption, agitation, inflamed and infected throat, associated with cough and dyspnea	発症時の寒気および発熱、頭部・顔面の発赤および腫脹、喉の掻痒および痛み、およびその後の壮熱、口渇、頭部・顔面の熱感および腫脹、皮疹、煩躁、喉の炎症および感染、これに伴う咳嗽および喘を特徴とする証。
風熱疫毒證		2.12.17	pattern/syndrome of wind-heat with epidemic toxin	a pattern/syndrome marked by chills and fever, redness and swelling of the head and face, painful swollen throat, skin eruption, agitation, thirst, headache, nausea, crimson tongue and rapid surging pulse	寒気および発熱、頭部・顔面の発赤および腫脹、喉の痛みを伴う腫脹、皮疹、煩躁、口渇、頭痛、悪心、絳舌および数・洪脈を特徴とする証。

検索表示（原表示の複数語を分離）	IST原表示	Code	Term	Definition/Description	定義/記述
濕遏衛陽證		2.12.18	pattern/syndrome of dampness obstructing defense yang	a pattern/syndrome marked by aversion to cold, lack of sweat, unsurfaced fever, headache as if swathed, cumbersome body and limbs, oppression in the chest, no feeling of hunger, absence of thirst, white slimy tongue coating and soggy relaxed pulse	悪寒、無汗、身熱不揚、何かを巻き付けられたような頭痛、動かしにくい身体および四肢、胸悶、空腹感の欠如、口渇欠如、白・膩苔および濡・緩脈を特徴とする証。
邪伏膜原證		2.12.19	pattern/syndrome of pathogen hidden in the pleurodiaphragmatic interspace	a pattern/syndrome marked by episodes of chills and fever at fixed hours, severe headache and generalized pain, distension and oppression in the chest and hypochondriac regions, vomiting of phlegmatic substance, and white powdered coating of the tongue	一定時刻に発現する寒熱発作、重度の頭痛および身痛、胸部および下肋部の膨満感および重圧感、痰性物質の喀出、および粉末状の白苔を特徴とする証。
氣分濕熱證		2.12.20	qi aspect dampness-heat pattern/ syndrome	a pattern/syndrome arising when pathogenic dampness-heat invades qi aspect, marked by unsurfaced fever, oppression in the chest and distension in the abdomen, jaundice, cumbersome limbs, nausea and vomiting, reddened tongue with yellow slimy coating and rapid slippery pulse	湿熱邪が気分に侵入するときに生じる証で、身熱不揚、胸悶および腹部膨満、黄疸、動かしにくい四肢、悪心および嘔吐、黄・膩苔を伴う紅舌および数・滑脈を特徴とする。
濕熱鬱阻氣機證		2.12.21	pattern/syndrome of dampness-heat obstructing qi movement	a pattern/syndrome arising when the dampness-heat pathogen obstructs qi movements, marked by fever, lassitude, aching limbs, thoracic oppression and abdominal distension, nausea, vomiting, reddish urine, and white slimy or slimy turbid tongue coating	湿熱邪により気機が妨げられるときに生じる証で、発熱、倦怠感、四肢の酸痛、胸悶および腹部膨満感、悪心、嘔吐、赤色尿、および白・膩苔または濁りがある膩苔を特徴とする。
濕重於熱證		2.12.22	pattern/syndrome of dampness predominating over heat	a pattern/syndrome marked by unsurfaced fever, thirst without appreciable intake of fluid, heavy feeling of the head and cumbersome limbs, impaired urination, diarrhea, reddened tongue with yellowish slimy coating, and slightly rapid and slippery pulse	身熱不揚、飲水欲を伴わない口渇、頭重感および動かしにくい四肢、排尿障害、泄瀉、黄・膩苔を伴う紅舌、および軽度の数・滑脈を特徴とする証。
熱重於濕證		2.12.23	pattern/syndrome of heat predominating over dampness	a pattern/syndrome marked by fever, thirst, reddened face and eyes, cumbersome limbs and head, ungratifying loose bowels, short voidings of little urine, reddened tongue with yellow slimy coating and rapid slippery pulse	発熱、口渇、顔面潮紅および目の充血、動かしにくい四肢および頭部、不快な下痢、短時間の微量尿の排出、黄・膩苔を伴う紅舌および数・滑脈を特徴とする証。
濕熱浸淫證		2.12.24	spreading dampness-heat pattern/ syndrome	a pattern/syndrome marked by redness, swelling, itching, ulceration and exudation of the eyelid, ear, nose, mouth angle, fingers or toes	眼瞼、耳、鼻、口角、指または踵の発赤、腫脹、掻痒、潰瘍および浸出液を特徴とする証。
暑兼寒濕證		2.12.25	pattern/syndrome of summerheat with cold-dampness	a pattern/syndrome arising when a combination of summerheat and cold-dampness attacks the exterior of the body, marked by headache, fever, aversion to cold, absence of sweating, epigastric oppression, irritable disposition and thin slimy tongue coating	暑と寒湿の両方が体表を攻撃するときに生じる証で、頭痛、発熱、悪寒、無汗、心窩部重圧感、いらいら傾向および薄・膩苔を特徴とする。
暑濕困阻中焦證		2.12.26	pattern/syndrome of summerheat-dampness encumbering the middle energizer	a pattern/syndrome arising when a combination of summerheat and dampness harasses the spleen and stomach, marked by intense fever, vexing thirst, profuse sweating, short voidings of urine, epigastric stuffiness,heaviness of the body and large surging pulse	暑と湿の両方が脾・胃を苦しめるときに生じる証で、激しい発熱、、わずらわしい口渇、多汗、短時間の尿の排出、心窩部が詰まった感じ、身体が重い感じおよび大・洪脈を特徴とする。
暑熱證		2.12.27	summerheat-heat pattern/syndrome	a pattern/syndrome of summerheat marked by fever, thirst, listlessness, shortness of breath, irritable disposition, dizziness, sweating, short voidings of yellow urine, reddened tongue with dry yellow coating and large surging pulse	発熱、口渇、無気力、短気、いらいら傾向、めまい、発汗、短時間の黄色尿の排出、黄・燥苔を伴う紅舌および大・洪脈を特徴とする暑熱の証。
暑濕證		2.12.28	summerheat dampness pattern/syndrome	a pattern/syndrome caused by a combination of summerheat and dampness, marked by fever, vexation, sensation of pressure in the chest, nausea and vomiting, reddened tongue with yellow slimy coating, and rapid slippery pulse	暑・湿の両方により生じる証で、発熱、心煩、胸部圧迫感、悪心および嘔吐、黄・膩苔を伴う紅舌および数・滑脈を特徴とする。
暑入陽明證		2.12.29	pattern/syndrome of summerheat entering yang brightness	a pattern/syndrome attributed to exuberant summer-heat that enters yang brightness, i.e., the qi aspect, marked by intense fever, profuse sweating, irritable disposition, headache with dizziness, reddened face, coarse breathing, thirst, dry teeth, yellow dry tongue coating, large surging pulse or large surging hollow pulse	旺盛な暑が陽明、すなわち気分に進入することにより生じる証で、激しい発熱、多汗、いらいら傾向、めまいを伴う頭痛、顔面潮紅、荒い呼吸、口渇、歯の乾燥、黄・燥苔、大・洪脈または大・洪芤脈を特徴とする。
暑傷津氣證		2.12.30	pattern/syndrome of summerheat damaging fluid and qi	a pattern/syndrome caused by summerheat which consumes qi and damages fluids, and manifested by fever with sweating, strong thirst, irritable disposition, flushed face, listlessness, lack of strength, shortness of breath, short voidings of deep-colored urine, redden tongue with dry yellow coating, and large floating weak pulse	暑による気の消耗および津の障害が原因で生じる証で、発汗を伴う発熱、強い口渇、いらいら傾向、顔面潮紅、無気力、乏力、短気、短時間の濃色尿の排出、燥・黄苔を伴う紅舌、および大・浮・弱脈が発現する。
暑熱動風證		2.12.31	pattern/syndrome of summerheat-heat stirring wind	a pattern/syndrome of summerheat marked by high fever, loss of consciousness, convulsion, opisthotonos and trismus	壮熱、神昏、抽搐、弓なり緊張および開口障害を特徴とする暑の証。
暑閉氣機證		2.12.32	pattern/syndrome of summerheat blocking qi movement	a pattern/syndrome of summerheat marked by sudden fainting, fever without much sweating, reversal cold of limbs, dyspnea, lockjaw, or nausea, vomiting and abdominal pain	突然の昏厥、大量の発汗を伴わない発熱、手足厥冷、喘、開口障害、または悪心、嘔吐および腹痛を特徴とする暑の証。
三焦辨證		**2.13.0**	**Triple Energizer Pattern Identification/ Syndrome Differentiation**		
三焦辨證		2.13.1	triple energizer pattern identification/ syndrome differentiation	categorization of patterns/syndromes according to the theory of the triple energizer	三焦の理論による証の分類。
上焦病證		2.13.2	upper energizer disease pattern/ syndrome	a pattern/syndrome arising when the pathogen invades the lung meridian at the early stage of epidemic febrile disease, marked by chills and fever, sweating, headache, cough, thirst, reddened tongue tip and edges, and rapid floating pulse; or marked by high fever without chills, cough, dyspnea, sweating, delirium and even loss of consciousness if the pathogen adversely passes to the pericardium	流行性熱病の初期に病邪が肺の経絡に侵入するときに生じる証で、寒気および発熱、発汗、頭痛、咳嗽、口渇、紅色の舌尖・舌辺、および数・浮脈、または、病邪が逆行して心包に至る場合は、寒気を伴わない壮熱、咳嗽、喘、発汗、譫妄および神昏を特徴とする。

検索表示（原表示の複数語を分離）	IST原表示	Code	Term	Definition/Description	定義/記述
中焦病證		2.13.3	middle energizer disease pattern/ syndrome	a pattern/syndrome attributed to invasion of the pathogen on the stomach meridian at the middle stage of epidemic febrile disease, either marked by dryness-heat in the stomach with yin damage, or marked by dampness-heat in the spleen with yin damage	流行性熱病の中期に病邪が胃の経絡に侵入するときに生じる証で、傷陰を伴う胃の燥熱、または傷陰を伴う脾の湿熱のいずれかを特徴とする。
下焦病證		2.13.4	lower energizer disease pattern/ syndrome	a pattern/syndrome due to impairment of liver-kidney yin at the later stage of epidemic febrile disease	流行性熱病の後期に肝腎陰の障害により生じる証。
三焦濕熱證		2.13.5	triple energizer dampness-heat pattern/syndrome	a pattern/syndrome attributed to invasion of the pathogenic dampness-heat on the whole body involving all three energizers	湿熱邪が全身に侵入し、三焦すべてを冒すことにより生じる証。
上焦濕熱證		2.13.6	upper energizer dampness-heat pattern/syndrome	a pattern/syndrome occurring at the initial stage of dampness-heat disease, generally manifested by chills and fever, heaviness feeling of the head and body, oppression in the chest, cough, absence of sweat and rapid soggy pulse	湿熱病の初期に生じる証で、一般に寒気および発熱、頭部および身体が重い感じ、胸悶、咳嗽、無汗および数・濡脈が発現する。
下焦濕熱證		2.13.7	lower energizer dampness-heat pattern/syndrome	a pattern/syndrome attributed to invasion of the pathogenic dampness-heat on the large intestine or bladder	湿熱邪が大腸または膀胱に侵入することにより生じる証。
臨床各科		**3.0.0**	**DISEASE**		
内科		**3.1.0**	**Internal Medicine**		
陰病		3.1.1	yin disease	(1) disease of yin meridians; (2) a general designation for deficiency patterns/syndromes and/or cold patterns/syndromes of the viscera, also called yin stages of disease transformation	(1)陰経の病。(2)臓の虚証および／または寒証の総称。病の傳化における陰病期とも呼ばれる。
陽病		3.1.2	yang disease	(1)disease of yang meridians;(2)a general designation for excess patterns/syndromes and/or heat patterns/syndromes, also called yang stages of disease transformation	(1)陽経の病。(2)臓の実証および／または熱証の総称。病の傳化における陽病期とも呼ばれる。
傷寒		3.1.3	cold damage	(1) a general term for various externally contracted febrile diseases; (2) a condition caused by cold, manifested as chills and fever, absence of sweating, headache and floating tense pulse	(1)外感する多様な熱病の一般用語。(2)寒による病態で、悪寒および発熱を発現し、無汗、頭痛および浮・緊脈を呈する。
雜病		3.1.4	miscellaneous disease	various internal diseases other than cold-induced diseases and warm diseases	寒病および温病以外の多様な内病。
新感		3.1.5	new contraction	immediate onset of the febrile diseases after invasion by the exopathogen, marked by exterior syndrome of aversion to wind-cold at the beginning	外邪侵入による熱病の速やかな発症。初期に悪風、悪寒の表証を現す。
時疫		3.1.6	seasonal epidemic	epidemic infectious disease in a certain season	特定の季節に流行する感染症。
感冒		3.1.7	common cold	affliction of the lung-superficies by pathogenic wind, mainly manifested as fever, chills, headache, general aching, congested nose, sneezing, itching throat and cough	風邪による肺表の苦痛であり、主に発熱、悪寒、頭痛、全身痛、鼻閉、噴嚏、喉の掻痒および咳嗽を発現する。
時行感冒		3.1.8	influenza	a disease attributable to invasion of the lung-superficies by an epidemic pathogen that causes acute fever, sore throat, headache and general aching	流行性病邪の肺表への侵入による疾患で、急な発熱、喉頭痛、頭痛および全身痛を起こす。
傷濕		3.1.9	dampness damage	a disease due to external contraction of dampness or obstruction of the stomach and intestines by dampness-turbidity	外感湿邪または湿濁による胃腸閉塞による疾患。
痢疾		3.1.10	dysentery	a disease characterized by abdominal pain, tenesmus, diarrhea with stool containing mucus and blood	腹痛、裏急後重、粘液および血液を含む便を伴う泄瀉を特徴とする疾患。
疫毒痢		3.1.11	epidemic toxin dysentery	severe case of dysentery characterized by acute onset of high fever, headache, severe abdominal pain, frequent stools containing blood and mucus, and even loss of consciousness with convulsions or reversal cold of the limbs and cyanosis	急性発症する壮熱、頭痛、重度の腹痛、血液および粘液を含む頻回の排便を特徴とする重度の痢疾で、抽搐あるいは四肢の厥冷を伴う神昏およびチアノーゼを起こすことすらある。
休息痢		3.1.12	intermittent dysentery	chronic dysentery with frequent relapse	頻繁に再発する慢性痢疾。
噤口痢		3.1.13	food-denying dysentery	severe case of dysentery with utter loss of appetite and vomiting upon eating and drinking	完全な食欲喪失および飲食時の嘔吐を伴う重度の痢疾。
霍亂		3.1.14	cholera	a disease characterized by sudden onset of simultaneous vomiting and diarrhea with the vomitus and stool like rice water, referring to acute gastroenteritis, food poisoning and cholera	米のとぎ汁様の嘔吐物と便を伴う嘔吐、痢疾の同時突発を特徴とする疾患。急性胃腸炎、食中毒、コレラを表す。
乾霍亂		3.1.15	dry cholera	an acute illness characterized by sudden onset of abdominal colic and epigastric oppression accompanied by desire but failure to vomit and desire but failure to defecate	腹部疝痛および心窩部圧迫の突発を特徴とする急性疾患。嘔吐および便意を催すが排便はない。
瘧	瘧; 瘧疾(病)	3.1.16	malaria	a disease attributed to contraction of malarial parasites, marked by paroxysms of shivering chills, high fever and sweating, also known as malaria-like disease	マラリア寄生虫感受による疾患で、悪寒戦慄、壮熱、発汗の発作を現す。マラリア様疾患としても知られる。
瘧疾	瘧; 瘧疾(病)	3.1.16	malaria	a disease attributed to contraction of malarial parasites, marked by paroxysms of shivering chills, high fever and sweating, also known as malaria-like disease	マラリア寄生虫感受による疾患で、悪寒戦慄、壮熱、発汗の発作を現す。マラリア様疾患としても知られる。
瘧疾病	瘧; 瘧疾(病)	3.1.16	malaria	a disease attributed to contraction of malarial parasites, marked by paroxysms of shivering chills, high fever and sweating, also known as malaria-like disease	マラリア寄生虫感受による疾患で、悪寒戦慄、壮熱、発汗の発作を現す。マラリア様疾患としても知られる。
濕瘧		3.1.17	dampness malaria	malaria complicated by dampness, manifested paroxysms of chills and unsurfaced fever, accompanied by impaired sweating, nausea, vomiting, anorexia, slimy tongue coating and relaxed pulse	湿により悪化した瘧で、悪寒および非表熱の発作を発現する。発汗不全、悪心、嘔吐、厭食、膩苔、緩脈を併存する。
溫瘧		3.1.18	warm malaria	malaria with higher fever and lower chills than an ordinary attack, accompanied by inhibited sweating and dire thirst	通常の発作より熱が高く、軽度の悪寒を伴う瘧。発汗不利、極度の口渇が付随する。
寒瘧		3.1.19	cold malaria	malaria with higher chills and lower fever or even no fever, no sweating, and absence of thirst	強度の悪寒、軽度の発熱または無発熱の場合すらあり、無汗、無口渇が付随する。
勞瘧		3.1.20	taxation malaria	chronic malaria with mild chills and fever, and with attacks being brought on by fatigue	軽度の悪寒および発熱、疲労による発作を伴う慢性の瘧。
瘴瘧		3.1.21	miasmic malaria	severe malaria with loss of consciousness or jaundice	神昏または黄疸を伴う重度の瘧。

検索表示（原表示の複数語を分離）	IST原表示	Code	Term	Definition/Description	定義/記述
瘴氣		3.1.22	miasma	noxious effluvium that is alleged to cause malaria	瘧の原因といわれる有害な臭気。
瘟疫		3.1.23	pestilence	a general term for virulent infectious epidemic diseases	悪性の感染症の一般用語。
大頭瘟		3.1.24	erysipelas facialis	an acute infection of the face marked by high fever and local redness, swelling, hotness and pain	壮熱、局所発赤を発現する急性感染症で、顔貌、腫張、熱、疼痛を特徴とする。
溫毒		3.1.25	warm toxin	(1) warm pathogen combined with heat toxin; (2) any acute inflammatory diseases attributable to contraction of such pathogen	(1)熱毒を伴う温邪。(2)この種の温邪の感受による急性炎症疾患。
溫病		3.1.26	warm disease	a general terms for acute externally contracted diseases caused by warm pathogens, with fever as the chief manifestation, also known as warm pathogen disease	外感した温邪による急性疾患の一般用語。主な発現として発熱を伴う。温邪病としても知られる。
春溫		3.1.27	spring warmth	a warm disease contracted in spring	春季に感受した温邪。
風溫		3.1.28	wind-warmth	a warm disease attributable to contraction of wind-heat pathogen	風熱邪の感受による温邪。
中暑	中暑;暍	3.1.29	summerheat stroke	an acute febrile disease attributable to contraction of summerheat, the same as heatstroke	暑の感受による急性熱性疾患。熱射病と同義。
暍	中暑;暍	3.1.29	summerheat stroke	an acute febrile disease attributable to contraction of summerheat, the same as heatstroke	暑の感受による急性熱性疾患。熱射病と同義。
冒暑		3.1.30	summerheat affliction	common cold in summer	夏季にみられる感冒。
傷暑		3.1.31	summerheat damage	a general term for various conditions caused by summerheat, especially for mild cases of heatstroke and sunstroke	特に軽度の熱射病および日射病に対する、暑邪による多様な病態の一般用語。
伏暑		3.1.32	latent summerheat	an acute febrile disease caused by latent summerheat or summerheat-dampness pathogen, occurring in autumn or winter	秋季または冬季に発生する、後発性の暑邪または暑湿邪により起こる急性発熱性疾患。
暑癎		3.1.33	summerheat convulsions	a type of severe summerheat stroke in summer with loss of consciousness and convulsions	神昏および抽搐を伴う夏季の重度の中暑の一種。
暑溫		3.1.34	summerheat-warmth	a warm disease attributable to contraction of summerheat pathogen	暑邪の感受による温疾。
暑風		3.1.35	summerheat-wind	a disease marked by sudden onset of opisthotonos and convulsions caused by summerheat	暑により起こる弓なり緊張の突発および抽搐を現す疾患。
濕溫		3.1.36	dampness-warmth	a warm disease attributable to contraction of dampness-heat pathogen	湿暑邪の感受による温病。
陰毒		3.1.37	yin toxin	a disease characterized by erythema and bluish facial complexion, accompanied by generalized pain and sore throat	紅斑および青みがかった面色を特徴とする疾患。全身痛および喉頭痛が付随する。
陽毒		3.1.38	yang toxin	a disease characterized by erythema bright in color, accompanied by sore throat and spittle with bloody pus	色の明るい紅斑を特徴とする疾患。喉頭痛および血性の膿を伴う唾液が付随する。
咳嗽		3.1.39	cough	(1) the expelling of air from the lungs suddenly with an explosive noise or expectoration of sputum; (2) any disease mainly manifested by cough	(1)破裂音または喀痰を伴う、突然の肺からの排気。(2)主に咳嗽を発現する疾患。
乾咳		3.1.40	dry cough	cough with no or little expectoration	喀痰を伴わない、またはほとんど伴わない咳嗽。
五更咳		3.1.41	fifth-watch cough	cough occurring or exaggerated daily just before daybreak	連日夜明け直前に発生あるいは悪化する咳嗽。
肺咳		3.1.42	lung cough	cough due to ascending counterflow of lung qi	肺気の上逆による咳嗽。
哮喘		3.1.43	wheezing and dyspnea	a diseased state characterized by paroxysmal labored breathing accompanied by a whistling sound, such as in asthma	喘息など、笛声音を併存する発作性の努力性呼吸を特徴とする病的状態。
哮		3.1.44	wheezing	difficult and labored breathing with a whistling sound	笛声音を伴う、困難な努力性呼吸。
寒哮	寒哮;冷哮	3.1.45	cold wheezing	a type of asthma due to cold-phlegm obstructing the airway, marked by dyspnea with wheezing, cough with thin mucous expectoration, fullness and oppression in the chest, whitish and slippery tongue coating, and floating tight pulse	寒痰の気道閉塞による喘息の一種。哮を伴う喘、希薄な痰の喀出、胸部の満および圧迫感、白みがかった滑苔、浮・緊脈を現す。
冷哮	寒哮;冷哮	3.1.45	cold wheezing	a type of asthma due to cold-phlegm obstructing the airway, marked by dyspnea with wheezing, cough with thin mucous expectoration, fullness and oppression in the chest, whitish and slippery tongue coating, and floating tight pulse	寒痰の気道閉塞による喘息の一種。哮を伴う喘、希薄な痰の喀出、胸部の満および圧迫感、白みがかった滑苔、浮・緊脈を現す。
熱哮		3.1.46	heat wheezing	a type of asthma due to retention of heat-phlegm in the lung, marked by dyspnea, wheezing, thick and yellowish expectoration, flushed face, thirst, reddened tongue with yellow slimy coating and rapid slippery pulse	熱痰の肺内滞留による喘息の一種。喘、哮、黄色がかった濃厚な痰の喀出、顔面潮紅、口渇、黄・膩苔を伴う発赤した舌および数・滑脈を現す。
喘		3.1.47	dyspnea	difficult and labored breathing	困難な努力性呼吸。
實喘		3.1.48	dyspnea of excess type	dyspnea caused by external contraction, marked by rapid, forceful and coarse breathing with acute onset and short duration	外感により起こる喘。急速な発症および短期間の持続を伴う、急速、強力、粗い呼吸を現す。
虛喘		3.1.49	dyspnea of deficiency type	dyspnea due to insufficient lung and kidney qi, marked by shortness of breath and dyspnea upon exertion, usually gradual on onset and chronic in nature	肺腎の不十分な気による喘。短気、労作による喘を現し、通常、発症は緩徐であり性質は慢性的である。
短氣		3.1.50	shortness of breath	rapid labored breathing	急速な努力性呼吸。
少氣		3.1.51	shortage of qi	weak or faint breathing, also known as shallow breathing	虚弱性または失神性呼吸。浅呼吸 としても知られる。
肺脹		3.1.52	lung distention	a disease of the lung characterized by persistent distension of the lung, manifested by a sensation of pressure in the chest, chronic cough and expectoration, shortness of breath or dyspnea, cyanotic lips and tongue	肺の持続的な脹を特徴とする肺疾患。胸部圧迫感、慢性的な咳嗽および喀痰、短気あるいは喘、口唇および舌のチアノーゼを発現する。
肺癰		3.1.53	lung abscess	abscess occurring in the lung due to heat toxin, marked by sudden onset of fever, cough, chest pain, expectoration of foul-smelling bloody purulent sputum	熱毒により肺内に発生する癰。突発的な発熱、咳嗽、胸部痛、悪臭を有する血性化膿性痰の喀出を現す。
肺癆		3.1.54	lung consumption	a consumptive disease due to overstrain, characterized by cough, hemoptysis, tidal fever, night sweating and emaciation	過労による消費性疾患。咳嗽、喀血、潮熱、盜汗、羸痩を特徴とする。
梅核氣		3.1.55	plum-pit qi	a disease characterized by a sensation of a foreign body present in the throat which can be neither swallowed nor ejected, also known as globus hystericus	嚥下、排出のいずれも不能な、喉頭の異物感を特徴とする疾患。ヒステリー球としても知られる。

検索表示（原表示の複数語を分離）	IST原表示	Code	Term	Definition/Description	定義/記述
驚悸		3.1.56	fright palpitations	palpitation ascribed to being frightened	驚による心悸。
怔忡		3.1.57	fearful throbbing	a severe case of palpitation	重度の心悸。
胸痺		3.1.58	chest impediment	a disease characterized by paroxysms of pectoral pain, sometimes accompanied by a feeling of oppression	胸筋痛の発作を特徴とする疾患で、圧迫感を併存することがある。
結胸		3.1.59	chest bind	a diseased state attributable to accumulation of pathogens (such as heat or cold in combination with retained fluid or phlegm or stagnant food) in the chest and abdomen, often manifested by local rigidity, fullness and tenderness, also the same as chest constriction	胸部および腹部内に邪(津または痰の滞留、または食の滞留と組み合わせた熱または寒など)が蓄積することによる病的状態。しばしば局所の堅、満、圧痛を発現する。胸部絞窄感と同義。
心痛		3.1.60	heart pain	a general term for pain in the precordial and epigastric regions	前胸部および心窩部の疼痛に対する一般用語。
卒心痛		3.1.61	sudden heart pain	sudden attack of heart pain due to contraction of pathogenic heat	熱邪の感受による心痛の突発的発作。
眞心痛		3.1.62	true heart pain	a disease characterized by sudden occurrence of severe heart pain accompanied by sweating, pale complexion, cyanotic lips, cold limbs with the hands and feet turning blue up to the joints, and hardly perceptible pulse	突然発生する重度の心痛を特徴とする疾患。発汗、面色蒼白、口唇チアノーゼ、関節まで蒼白になった手足を伴う四肢の冷えを併存し、脈はほとんど触れない。
眩暈	眩暈;<br>頭暈;<br>瞑眩	3.1.63	dizziness	a diseased state characterized by a whirling sensation in the head with visual distortion	視覚の歪みを伴う、頭部内で旋回するような感覚を特徴とする病的状態。
頭暈	眩暈;<br>頭暈;<br>瞑眩	3.1.63	dizziness	a diseased state characterized by a whirling sensation in the head with visual distortion	視覚の歪みを伴う、頭部内で旋回するような感覚を特徴とする病的状態。
瞑眩	眩暈;<br>頭暈;<br>瞑眩	3.1.63	dizziness	a diseased state characterized by a whirling sensation in the head with visual distortion	視覚の歪みを伴う、頭部内で旋回するような感覚を特徴とする病的状態。
神昏		3.1.64	loss of consciousness	any diseased state characterized by loss of consciousness with no response to stimulation	刺激に対する無反応を伴う、意識喪失を特徴とする病的状態。
類中風		3.1.65	apoplectic wind stroke	cerebral stroke caused by endogenous pathogenic wind	内風邪により起こる脳卒中。
中風前兆症		3.1.66	prodrome of wind stroke	symptoms indicating the onset of wind stroke, such as headache and dizziness, numbness and weakness of the limb	頭痛および眩暈、四肢の麻木および虚弱など、中風発症を示す症状。
中風後遺症		3.1.67	sequela of wind stroke	functional disturbances following an attack of wind stroke, such as hemiplegia, dysphasia and dementia	半身不随、言語障害、認知症など、風邪の発作に後続する機能障害。
中絡		3.1.68	collateral stroke	mildest form of wind stroke with slight distortion of the face and numbness of limbs	わずかな顔貌の歪み、四肢の麻木を伴う、極めて軽度の中風。
中經		3.1.69	meridian stroke	mild form of wind stroke with hemiplegia, paralysis of the face and limbs, dysphasia, but no impairment of consciousness	半身不随、顔面および四肢の麻痺、不全失語症を伴う軽度の中風。しかし、意識障害はない。
中腑		3.1.70	bowel stroke	serious form of wind stroke with onset of fainting, hemiplegia, distortion of the face and dysphasia	昏厥、半身不随、顔貌の歪み、不全失語症の発症を伴う、重度の中風。
中臟		3.1.71	visceral stroke	very serious form of wind stroke with sudden loss of consciousness, aphasia, and paralysis of lips with salivation	突然の神昏、失語症、流涎を伴う口唇の麻痺を伴う極めて重度の中風。
半身不隨	半身不隨; 偏枯	3.1.72	hemiplegia	paralysis of one side of the body	体の片側の麻痺。
偏枯	半身不隨; 偏枯	3.1.72	hemiplegia	paralysis of one side of the body	体の片側の麻痺。
痲木		3.1.73	numbness	reduced sensitivity to touch	触覚低下。
不得臥	不得臥;<br>失眠	3.1.74	insomnia	prolonged inability to obtain normal sleep	長期の正常睡眠不能。
失眠	不得臥;<br>失眠	3.1.74	insomnia	prolonged inability to obtain normal sleep	長期の正常睡眠不能。
健忘		3.1.75	forgetfulness	poor memory; tendency to forget matters, the same as amnesia	記憶力の低下、物事を忘れやすい。健忘と同義。
痴呆		3.1.76	dementia	diseases characterized by marked decline of cognitive and intellectual functions, usually progressive, without loss of perception or consciousness	認知機能、知的機能の顕著な低下を特徴とする疾患。通常進行性であり、感覚喪失または神昏は伴わない。
陽癇		3.1.77	yang epilepsy	epilepsy of yang nature, characterized by paroxysmal transient loss of consciousness, seizures, upward staring of the eyes often preceded by vocalizations	陽性の癲癇。発作性の一時的な神昏、発作、しばしば発声が先行する上方凝視を特徴とする。
陰癇		3.1.78	yin epilepsy	epilepsy of yin nature characterized by transient clouding of consciousness without convulsions	陰性の癲癇。抽搐を伴わない一時的な意識混濁を特徴とする。
癲病		3.1.79	depressive psychosis	a psychotic disorder characterized by severe depression	重度の鬱病を特徴とする精神病性障害。
狂病		3.1.80	manic psychosis	a psychotic disorder characterized by mental and physical hyperactivity, disorganization of behavior, and elevation of mood	精神的および肉体的運動亢進、行動の解体、気分高揚を特徴とする精神病性傷害。
百合病		3.1.81	lily disease	ancient term for neurosis characterized by mental strain, listlessness, sleeplessness, anorexia, pseudo heat and pseudo cold, bitterness in the mouth, yellow urine and rapid pulse	精神的負担、気力低下、不眠、厭食、偽熱および偽寒、口苦、黄色尿、数脈を特徴とする神経症の古語。
胃脘痛		3.1.82	stomach duct pain	a diseased state characterized by pain in the epigastric region, the same as epigastric pain	心窩部の疼痛を特徴とする病的状態。心窩部痛と同義。
脘痞	脘痞;<br>心下痞	3.1.83	gastric stuffiness	a diseased state characterized by discomfort in the epigastric region with feelings of fullness and pressure but without local rigidity and tenderness	充満感および圧迫感を伴うが局所の痞および圧痛は伴わない、心窩部の不快感を特徴とする病的状態。
心下痞	脘痞;<br>心下痞	3.1.83	gastric stuffiness	a diseased state characterized by discomfort in the epigastric region with feelings of fullness and pressure but without local rigidity and tenderness	充満感および圧迫感を伴うが局所の痞および圧痛は伴わない、心窩部の不快感を特徴とする病的状態。
實痞		3.1.84	stuffiness of excess type	sensation of stuffiness caused by accumulation of pathogenic factors	邪の蓄積により起こる痞の感覚。

検索表示（原表示の複数語を分離）	IST原表示	Code	Term	Definition/Description	定義/記述
虚痞		3.1.85	stuffiness of deficiency type	sensation conditions of stuffiness occurring in deficiency condition	虚の状態で発生する痞の感覚。
嘔吐		3.1.86	vomiting	a diseased state characterized by forcible expulsion of the stomach contents through the mouth	胃内容物の口からの激しい圧出を特徴とする病的状態。
吐酸		3.1.87	acid vomiting	expulsion through the mouth of sour fluid that flows up from the stomach	胃から湧出する酸味のある液体の口からの圧出。
嘈雜		3.1.88	gastric upset	a diseased state characterized by a disturbed feeling in the stomach, often accompanied by acid regurgitation	胃をかき乱されたような感覚を特徴とする病的状態。しばしば酸の逆流が付随する。
呃逆	呃逆;噦	3.1.89	hiccup	upward reversion of stomach qi with an involuntary movement of the diaphragm, causing a characteristic sound	横隔膜の不随意運動を伴う胃気の逆行。特徴的な音を発する。
噦	呃逆;噦	3.1.89	hiccup	upward reversion of stomach qi with an involuntary movement of the diaphragm, causing a characteristic sound	横隔膜の不随意運動を伴う胃気の逆行。特徴的な音を発する。
噎膈		3.1.90	dysphagia-occlusion	difficulty in swallowing caused by narrowing or obstruction of the esophagus	食道狭窄により起こる嚥下困難。
反胃	反胃;胃反	3.1.91	stomach reflux	flowing back of the stomach contents into the esophagus and mouth a long time after eating, also known as gastro esophageal reflux	食後長時間経過後の、食道および口への胃内容物の逆流。胃食道逆流としても知られる。
胃反	反胃;胃反	3.1.91	stomach reflux	flowing back of the stomach contents into the esophagus and mouth a long time after eating, also known as gastro esophageal reflux	食後長時間経過後の、食道および口への胃内容物の逆流。胃食道逆流としても知られる。
關格		3.1.92	block and repulsion (disease)	a diseased state characterized by urinary stoppage and vomiting	尿の停止および嘔吐を特徴とする病的状態。
便秘		3.1.93	constipation	infrequent or difficult evacuation of the feces	低頻度排便および排便困難。
泄瀉	泄瀉;下利;泄瀉病	3.1.94	diarrhea	a diseased condition characterized by abnormal frequency and liquidity of fecal discharge	排便頻度および糞便中水分量の異常を特徴とする病態。
下利	泄瀉;下利;泄瀉病	3.1.94	diarrhea	a diseased condition characterized by abnormal frequency and liquidity of fecal discharge	排便頻度および糞便中水分量の異常を特徴とする病態。
泄瀉病	泄瀉;下利;泄瀉病	3.1.94	diarrhea	a diseased condition characterized by abnormal frequency and liquidity of fecal discharge	排便頻度および糞便中水分量の異常を特徴とする病態。
久泄		3.1.95	chronic diarrhea	diarrhea lasting for a long time or continually recurring	長期間持続または継続的に再発する泄瀉。
脾約		3.1.96	splenic constipation	infrequent passage of dry hardened feces as a consequence of dysfunction of the spleen	脾の機能不全の結果、乾固した便が低頻度で排出されること。
藏結	藏結;臟結	3.1.97	visceral bind	a diseased state arising when excess cold bound in the internal organs and manifested by epigastric distension, fullness and pain or tender mass accumulated in the hypochondriac region	実寒が内臓に結合したときに生じる病的状態。心窩部の脹満感、疼痛または脇部に蓄積した圧痛のある腫瘤を発現する。
臟結	藏結;臟結	3.1.97	visceral bind	a diseased state arising when excess cold bound in the internal organs and manifested by epigastric distension, fullness and pain or tender mass accumulated in the hypochondriac region	実寒が内臓に結合したときに生じる病的状態。心窩部の脹満感、疼痛または脇部に蓄積した圧痛のある腫瘤を発現する。
傷食		3.1.98	food damage	any disease of damage to the spleen and stomach by food	食による脾および胃への傷による疾患。
黃胖病		3.1.99	sallow disease	a diseased state characterized by withered-yellowish discoloration of the skin and swelling of the face and ankles	干からびて黄色がかった肌膚の変色、顔および足関節の腫脹を特徴とする病的状態。
黃疸		3.1.100	jaundice	a diseased condition characterized by yellow discoloration of the skin and sclera	肌膚および強膜の黄変を特徴とする病態。
急黃		3.1.101	acute jaundice	a critical case of jaundice with sudden onset, rapid deterioration and poor prognosis, accompanied by high fever, dire thirst, impairment of consciousness and delirium, indicating inward invasion of dampness-heat with toxin into the blood and nutrient aspects	突然の発症、急速な悪化、予後不良を伴う重症例の黄疸。壮熱、極度の口渇、意識障害、譫妄を併存し、血分および栄分への毒を伴う湿熱の内襲を示す。
陽黃		3.1.102	yang jaundice	a type of jaundice characterized by bright yellow discoloration of the skin and sclera, accompanied by damp-heat symptoms such as fever, thirst and yellow slimy tongue coating	肌膚および強膜の明黄色への変化を特徴とする黄疸の一種。熱、口渇、黄色の膩苔など湿熱症状が付随する。
陰黃		3.1.103	yin jaundice	a type of jaundice characterized by dim yellow discoloration of the skin and sclera, usually chronic and accompanied by cold-dampness symptoms such as listlessness, fear of cold, white slimy tongue coating and soggy relaxed pulse	肌膚および強膜の薄暗い黄色への変化を特徴とする黄疸の一種。通常慢性であり、気力低下、畏寒、白色の膩苔、濡・緩脈などの寒湿症状が付随する。
穀疸		3.1.104	dietary jaundice	a type of jaundice characterized by dizziness immediately after meal, and painful fullness in the stomach due to indigestion	食事直後の眩暈、および消化不良による胃での疼痛のある満を特徴とする黄疸の一種。
酒疸		3.1.105	alcoholic jaundice	a type of jaundice due to excessive consumption of liquor	酒の過飲による黄疸の一種。
肝著		3.1.106	liver fixity	a diseased condition arising when qi and blood of the liver meridian become depressed and stagnant	肝経の気血が低下および停滞した時に生じる病態。
膽脹		3.1.107	gallbladder distention	a diseased condition arising when the gallbladder qi is stagnant, and manifested by recurrent right upper abdominal pain with stuffiness and distension	胆嚢の気が停滞している時に生じる病態。痞、脹を伴う再発性の右上腹部痛を発現する。
鼓脹		3.1.108	tympanites	a disease characterized by the severely distended abdomen like a drum accompanied by a somber yellow discoloration of the skin and prominent veins on the abdominal wall	太鼓様の重度の脹腹を特徴とする疾患。肌膚の黒ずんだ黄変、腹壁の大きな静脈が付随する。
膚脹		3.1.109	cutaneous distention	a type of distension disease due to contraction of cold pathogen, arising when the pathogen stays in the skin together with the defense qi	寒邪の感受による脹疾患の一種。邪が衛気とともに肌膚内にあるときに生じる。
脹滿		3.1.110	distention and fullness	distension without and fullness within, a diseased condition in which damage to the spleen and stomach results in qi stagnation	外脹および内満。脾と胃の傷が気の停滞をもたらす時の病態。
水脹		3.1.111	water distention	edema due to insufficiency of yang qi that leads to accumulation of water-dampness, marked initially by abdominal distension, and then swelling of the extremities	水湿の蓄積につながる、陽気不十分による浮腫。初期は腹脹、その後は四肢の腫脹を現す。

検索表示（原表示の複数語を分離）	IST原表示	Code	Term	Definition/Description	定義/記述
水腫	水腫;<br>水氣<br>(病)	3.1.112	edema	any disease characterized by subcutaneous fluid retention	津の皮下滞留を特徴とする疾患。
水氣	水腫;<br>水氣<br>(病)	3.1.112	edema	any disease characterized by subcutaneous fluid retention	津の皮下滞留を特徴とする疾患。
水氣病	水腫;<br>水氣<br>(病)	3.1.112	edema	any disease characterized by subcutaneous fluid retention	津の皮下滞留を特徴とする疾患。
陽水		3.1.113	yang edema	edema due to attack of wind or immersion of water-dampness involving the lung and the spleen respectively, usually with an acute onset and a short course, accompanied by exterior, heat and excess symptoms	それぞれ肺と脾に関与する、風の襲または水湿の浸漬による水腫。通常急性の発症および短期の経過を伴い、外、熱、実の症状を併存する。
陰水		3.1.114	yin edema	edema due to deficiency of the spleen and the kidney with inhibited qi movement, usually with a gradual onset and a long course, accompanied by interior, cold and deficiency symptoms	氣機不利を伴う、脾および腎の虚による水腫。通常、緩徐な発症と長期の経過を伴い、内、寒、虚の症状が付随する。
風水		3.1.115	wind edema	a type of edema especially of the face and head, ascribed to attack on the lung by pathogenic wind, manifested by sudden onset of edema accompanied by fever with aversion to wind, aching joints, and oliguria	風邪による肺への侵襲による、特に顔面および頭部の水腫の一種。悪風、関節痛、乏尿を伴う発熱の付随する、水腫が突然発症する。
皮水	皮水;<br>裏水	3.1.116	skin edema	pitting edema accompanied by abdominal distension and floating pulse	腹脹および浮脈を伴う浮腫。
裏水	皮水;<br>裏水	3.1.116	skin edema	pitting edema accompanied by abdominal distension and floating pulse	腹脹および浮脈を伴う浮腫。
正水		3.1.117	regular edema	a type of edema accompanied by abdominal fullness and dyspnea	腹満および喘を伴う浮腫の一種。
石水		3.1.118	stony edema	a type of edema marked by stony hardness of the lower abdomen	下腹部の石様の堅を現す浮腫の一種。
黃汗		3.1.119	yellow sweat	a disease mainly caused by the interior accumulation of dampness-heat, and characterized by edema, fever and yellow-colored sweat	主に湿熱の裏への蓄積により起こる疾患。浮腫、発熱、黄色汗を特徴とする。
淋病		3.1.120	strangury disease	a variety of diseases characterized by frequent, painful and dripping urination	頻尿、排尿痛、淋瀝を特徴とする多様な疾患。
熱淋		3.1.121	heat strangury	strangury ascribed to dampness-heat, marked by urgency and frequency of painful urination with acute onset, and accompanied by chills and fever, lumbar pain and cramps, and distension in the lower abdomen	湿熱による淋で、急性発症を伴う有痛性の尿意促迫、頻尿を現す。悪寒および発熱、腰痛および腰椎抽搐、下腹部の脹が付随する。
石淋		3.1.122	stone strangury	painful and difficult urination due to the passage of urinary calculi	尿路結石の排出による排尿痛および排尿困難。
氣淋		3.1.123	qi strangury	strangury due to bladder qi disorders, characterized by painful voiding of urine and lower abdominal distension and fullness	膀胱の気の障害による淋。排尿痛および下腹部の脹と満を特徴とする。
血淋		3.1.124	blood strangury	strangury characterized by painful discharge of bloody urine	有痛性の血尿排出を特徴とする淋。
膏淋		3.1.125	unctuous strangury	painful discharge of turbid, milky urine like rice-water	有痛性の、米のとぎ汁様の乳濁尿の排出。
勞淋		3.1.126	fatigue strangury	a type of strangury characterized by recurrent dripping of urine ascribed to taxing fatigue, usually seen in chronic cases	重い労による再発性滴下尿を特徴とする淋の一種。通常慢性症例に見られる。
白淫		3.1.127	white ooze	seminal fluid in the urine or persistent vaginal discharge	尿中の精液または持続する膣分泌物。
白濁		3.1.128	white turbidity	(1) murky urine white in color; (2) discharge of a murky white substance from the urethra	(1)白濁尿。(2)尿道からの白濁した物質の排出。
尿濁		3.1.129	turbid urine (disease)	discharge of turbid urine, white like rice water, with no difficulty or pain in urination, a condition different from unctuous strangury	米のとぎ汁様の白濁尿の排泄。排尿困難および排尿痛はなく、淋とは異なる病態を伴う。
癃閉		3.1.130	dribbling urinary block (disease)	dribbling urination or complete blockage of urine flow	小便淋漓または排尿の完全閉塞。
遺精		3.1.131	seminal emission (disease)	a diseased state characterized by involuntary emission of semen	不随意な精液排出を特徴とする病的状態。
夢遺		3.1.132	dream emission (disease)	involuntary emission of semen during sleep associated with dreaming	夢に伴う睡眠中の不随意な精液排出。
滑精		3.1.133	spermatorrhea	involuntary and frequent discharge of semen without copulation	交合を伴わない、不随意で頻回の精液排出。
早泄		3.1.134	premature ejaculation (disease)	ejaculation of semen immediately after or even prior to penetration	性器挿入直後の、または挿入前の場合すらある射精。
陽痿		3.1.135	impotence	an abnormal state of a male characterized by inability to initiate or maintain an erection in sexual intercourse	性交時の勃起開始または維持の不能を特徴とする、男性機能の異常状態。
陽强		3.1.136	persistent erection	abnormal persistent erection accompanied by tenderness of the penis	陰茎の圧痛が付随する、持続的な勃起異常。
精冷		3.1.137	seminal cold	a diseased state characterized by cold thin seminal fluid which leads to sterility	寒で希薄な精液を特徴とする病的状態。不妊につながる。
精濁		3.1.138	seminal turbidity (disease)	an inflammatory disease of male genital organ marked by frequent discharge of white turbid secretion from the urethra, generally referring to prostatitis	尿道からの頻繁な白濁分泌物の排出を現す男性生殖器の炎症性疾患。一般に前立腺炎を表す。
陰陽易		3.1.139	yin-yang transmission	the disease contracted by a healthy person after sexual intercourse with one who has not yet recovered from an external contraction, also known as yin-yang exchange	外感から未回復の人が健康な人と性交した後に悪化あるいは新病に感受する疾患。陰陽交換としても知られる。
奔豚	奔豚;<br>奔豚氣	3.1.140	running piglet	an ancient name for the morbid condition characterized by a feeling of masses of gas ascending within the abdomen like running piglets, also known as running piglet qi	走っている豚のように、気体の塊が腹中を上っているような感覚を特徴とする罹患状態の古代名。奔豚気としても知られる。
奔豚氣	奔豚;<br>奔豚氣	3.1.140	running piglet	an ancient name for the morbid condition characterized by a feeling of masses of gas ascending within the abdomen like running piglets, also known as running piglet qi	走っている豚のように、気体の塊が腹中を上っているような感覚を特徴とする罹患状態の古代名。奔豚としても知られる。

検索表示（原表示の複数語を分離）	IST原表示	Code	Term	Definition/Description	定義/記述
鬱病		3.1.141	depression	a general term for diseased states characterized by a depressed mood with feelings of despair or uneasiness	絶望感、不安感を伴う抑うつ気分を特徴とする病的状態の一般用語。
血病		3.1.142	blood disease	a general term for diseases involving the blood, including bleeding, blood stasis and blood heat	出血、血瘀、血熱など、血に関与する疾患の一般用語。
鼻衄		3.1.143	nosebleed	hemorrhage from the nose irrelevant to trauma, also called epistaxis	外傷に関係しない鼻からの出血。鼻出血とも呼ばれる。
齒衄		3.1.144	gum bleeding	bleeding from the gums irrelevant to trauma	外傷に関係しない歯茎からの出血。
吐血		3.1.145	hematemesis	vomiting of blood	血の嘔吐。
便血		3.1.146	hematochezia	passage of blood in the stool	大便中への血の排出。
尿血		3.1.147	hematuria	discharge of bloody urine or blood streaks in the urine, but with no pain during urination	血尿排泄すなわち尿中の血痕であるが、排尿時の疼痛は伴わない。
紫斑		3.1.148	purpura	any disease characterized by ecchymosis or small hemorrhages in the skin and mucous membrane	斑状出血すなわち肌膚および粘膜内の小出血を特徴とする疾患。
蓄血		3.1.149	blood amassment (disease)	a disease caused by stagnated blood accumulated in a meridian/channel or an organ, e.g., in the uterus, or in the middle energizer	経穴、子宮などの器官または三焦中に蓄積した滞血による疾患。
痰飮		3.1.150	phlegm-fluid retention	(1) a general term for retention of phlegm and fluid in any part of the body; (2) a particular designation for retention of fluid in the gastrointestinal tract, e.g., gastric retention in pyloric stenosis	(1)体のいずれかの部分での痰および津の停滞の一般用語。(2)幽門狭窄における胃の停滞など消化管中の津の停滞に特定した定義。
懸飮		3.1.151	pleural fluid retention	a disease characterized by excess fluid retained in the side of the thorax with stretching pain during cough	喉頭側部に停滞する実津を特徴とする疾患。咳嗽時に引き延ばされるような痛みを伴う。
溢飮		3.1.152	subcutaneous fluid retention	a disease characterized by excessive fluid of the body spilling outward to the body surface	体表に溢れ出すほど過剰な体の津を特徴とする疾患。
支飮		3.1.153	thoracic fluid retention	retention of excessive fluid in the lung and chest	肺および胸の過剰な津の停滞。
伏飮		3.1.154	recurrent fluid retention	a disease characterized by phlegm-fluid lying latent in the body with episodes brought on by external contractions	体内に伏する痰津を特徴とする疾患。外感によりもたらされる発作を伴う。
微飮		3.1.155	mild fluid retention	a mild case of fluid retention mainly manifested by shortness of breath	主に短気を発現する、軽度の飲。
留飮		3.1.156	persistent fluid retention	a case of fluid retention with lingering course and difficult to cure	残存性経過および治癒困難を伴う飲の一例。
汗證	汗證; 汗病	3.1.157	abnormal sweating	any diseased state characterized by generalized or local abnormal sweating	全身性または局所性の異常発汗を特徴とする病的状態。
汗病	汗證; 汗病	3.1.157	abnormal sweating	any diseased state characterized by generalized or local abnormal sweating	全身性または局所性の異常発汗を特徴とする病的状態。
脫汗		3.1.158	shock sweating	profuse sweating associated with listlessness, cold limbs, and hardly perceivable pulse, usually occurring in a critical case	気力低下、四肢の冷え、ほとんど触れない脈と関連する大量発汗。通常、重症例で発生する。
消渴	消渴; 消渴病	3.1.159	wasting-thirst	any diseased state characterized by polydipsia, polyphagia, and polyuria, similar to diabetes	多飲、多食、多尿を特徴とする病的状態。糖尿病に類似。
消渴病	消渴; 消渴病	3.1.159	wasting-thirst	any diseased state characterized by polydipsia, polyphagia, and polyuria, similar to diabetes	多飲、多食、多尿を特徴とする病的状態。糖尿病に類似。
上消		3.1.160	upper wasting-thirst	a wasting-thirst case characterized by polydipsia with excessive drinking	過剰の飲酒を伴う多飲を特徴とする、消の症例。
中消		3.1.161	middle wasting-thirst	a wasting-thirst case characterized by polyphagia and emaciation	多食、羸痩を特徴とする、消の症例。
下消		3.1.162	lower wasting-thirst	a wasting-thirst case characterized chiefly by polyuria	主に多尿を特徴とする、消の症例。
虛勞		3.1.163	consumptive disease	a general term for chronic deficiency diseases due to consumption of yin, yang, qi and blood	陰、陽、気、血の消費による慢性の虚病の一般用語。
勞倦		3.1.164	fatigue due to overexertion	a diseased state caused by overexertion, manifested by fatigue, lassitude, shortness of breath upon exertion, and spontaneous sweating	労作過剰により起こる病的状態で、疲労、倦怠、労作時の短気、自発発汗を発現する。
積聚	積聚; 癥瘕	3.1.165	aggregation-accumulation	a general term referring to mass formation in the chest and abdomen, also called abdominal mass	胸部および腹部の腫瘤形成を表す一般用語。腹部腫瘤とも呼ばれる。
癥瘕	積聚; 癥瘕	3.1.165	aggregation-accumulation	a general term referring to mass formation in the chest and abdomen, also called abdominal mass	胸部および腹部の腫瘤形成を表す一般用語。腹部腫瘤とも呼ばれる。
厥	厥; 厥證	3.1.166	syncope	a morbid state characterized by temporary loss of consciousness with cold extremities up to the elbows and knees or beyond, the same as collapse	一時的な神昏を特徴とする病的状態で、肘部または膝部まで、またはそれを超えた四肢の冷えを伴う。虚脱と同義。
厥證	厥; 厥證	3.1.166	syncope	a morbid state characterized by temporary loss of consciousness with cold extremities up to the elbows and knees or beyond, the same as collapse	一時的な神昏を特徴とする病的状態で、肘部または膝部まで、またはそれを超えた四肢の冷えを伴う。虚脱と同義。
薄厥		3.1.167	flopping syncope	an acute disease characterized by sudden fainting	突発の昏厥を特徴とする急性疾患。
藏厥	藏厥; 臟厥	3.1.168	visceral syncope	syncope due to yang debilitation of internal organs	内臓の陽気の衰による厥。
臟厥	藏厥; 臟厥	3.1.168	visceral syncope	syncope due to yang debilitation of internal organs	内臓の陽気の衰による厥。
食厥		3.1.169	crapulent syncope	syncope due to eating and drinking too much at one sitting	1回での過剰な飲食による厥。
熱厥	熱厥; 煎厥	3.1.170	heat syncope	syncope due to yin debilitation and excessive heat, also called scorching syncope	陰衰および実熱による厥。灼厥とも呼ばれる。
煎厥	熱厥; 煎厥	3.1.170	heat syncope	syncope due to yin debilitation and excessive heat, also called scorching syncope	陰衰および実熱による厥。灼厥とも呼ばれる。
寒厥		3.1.171	cold syncope	syncope due to yang debilitation and excessive cold	陽衰および実寒による厥。
風厥		3.1.172	wind syncope	syncope due to transformation of liver qi into wind	肝気の風化による厥。
氣厥		3.1.173	qi syncope	syncope induced by emotional upset with upward counterflow of qi	気の上逆を伴う、情の雑により誘導される厥。
血厥		3.1.174	blood syncope	syncope due to a fit of rage that causes an upward reverse flow of qi and blood, marked by flushed face and stringy forceful pulse	気血の上逆流を起こす怒の合による厥。紅顔および強い弦脈を現す。
痰厥		3.1.175	phlegm syncope	syncope due to exuberant phlegm that causes a qi block	気閉を起こす過剰な痰による厥。

検索表示（原表示の複数語を分離）	IST原表示	Code	Term	Definition/Description	定義/記述
眞頭痛		3.1.176	true headache	a critical case of headache marked by sudden attack of severe pain in the head accompanied by nausea, vomiting, and counterflow cold of extremities up to the elbows and knees	重症例の頭痛。悪心、嘔吐、肘部および膝部までの肢の逆寒を併存する、頭部の劇痛の突発を現す。
頭風	頭風; 腦風	3.1.177	head wind	(1) chronic headache with repeated recurrence; (2) a general term for affections of the head caused by pathogenic wind, including headache, dizziness, facial paralysis, and itching of the scalp with much scurf	(1)繰り返し再発する慢性の頭痛。(2)頭痛、眩暈、顔面神経麻痺、大量のふけを伴う頭皮の掻痒など、風邪による頭部の疾患の一般用語。
腦風	頭風; 腦風	3.1.177	head wind	(1) chronic headache with repeated recurrence; (2) a general term for affections of the head caused by pathogenic wind, including headache, dizziness, facial paralysis, and itching of the scalp with much scurf	(1)繰り返し再発する慢性の頭痛。(2)頭痛、眩暈、顔面神経麻痺、大量のふけを伴う頭皮の掻痒など、風邪による頭部の疾患の一般用語。
偏頭風		3.1.178	hemilateral head wind	chronic recurrent headache occurring on one side or one part of the head	頭部の片側または一部で起こる慢性の再発性頭痛。
雷頭風		3.1.179	thunder head wind	a diseased state characterized by pain with loud noise in the head	頭部内の大騒音を伴う疼痛を特徴とする病的状態。
痹病		3.1.180	impediment disease	a group of diseases caused by the invasion of wind, cold, dampness or heat pathogen on the meridian/ channel involving muscles, sinews, bones and joints, manifested by local pain, soreness, heaviness, or hotness, and even articular swelling, stiffness and deformities, also referring to arthralgia	風、寒、湿、熱の病邪が筋肉、筋、骨、関節などの経穴に侵襲することにより起こる疾患の一群。局所痛、痛み、重感、熱感を発現し、関節腫脹、硬直、変形の発現すらある。関節痛を表すこともある。
行痹	行痹; 風痹	3.1.181	moving impediment	an impediment disease characterized by migratory joint pains, also called wind impediment	移動性関節痛を特徴とする痹病。風痺とも呼ばれる。
風痹	行痹; 風痹	3.1.181	moving impediment	an impediment disease characterized by migratory joint pains, also called wind impediment	移動性関節痛を特徴とする痹病。行痺とも呼ばれる。
痛痹	痛痹; 寒痹	3.1.182	painful impediment	an impediment disease with severe joint pain exaggerated by cold, also called cold impediment	寒により悪化する重度の関節痛を伴う痹病。寒痺とも呼ばれる。
寒痹	痛痹; 寒痹	3.1.182	painful impediment	an impediment disease with severe joint pain exaggerated by cold, also called cold impediment	寒により悪化する重度の関節痛を伴う痹病。痛痺とも呼ばれる。
着痹	着痹; 濕痹	3.1.183	fixed impediment	an impediment disease with fixed joint pain, also called dampness impediment	固定性関節痛を伴う痹病。濕痹とも呼ばれる。
濕痹	着痹; 濕痹	3.1.183	fixed impediment	an impediment disease with fixed joint pain, also called dampness impediment	固定性関節痛を伴う痹病。着痹とも呼ばれる。
熱痹		3.1.184	heat impediment	an impediment disease with an acute onset of joint pain accompanied by local redness, swelling and hotness	関節痛の急性発症を伴う痹病。局所発赤、腫脹、熱感を併存する。
歴節風		3.1.185	joint-running wind	a disease characterized by redness and swelling of multiple joints, with acute pain and difficulty in bending and stretching, and finally developing into articular deformity	複数関節の発赤および腫脹を特徴とする疾患で、屈伸時の急性疼痛および困難を伴い、最終的に関節変形を発症する。
骨痹		3.1.186	bone impediment	a type of impediment disease mainly involving the bones and joints	主に骨および関節に関与する痹病の一種。
肌痹		3.1.187	flesh impediment	a type of impediment disease mainly involving the flesh	主に肉に関与する痹病の一種。
血痹		3.1.188	blood impediment	a type of impediment disease marked by local numbness of extremities	四肢の局所麻木を現す、痹病の一種。
脈痹		3.1.189	vessel impediment	a type of impediment disease mainly involving the vessels	主に血管に関与する痹病の一種。
心痹		3.1.190	heart impediment	a type of impediment disease mainly involving the heart	主に心に関与する痹病の一種。
腸痹		3.1.191	intestinal impediment	a type of impediment disease mainly involving the intestines	主に腸に関与する痹病の一種。
痙病		3.1.192	convulsive disease	any diseased state marked by neck rigidity, convulsion of the limbs, and even clenched jaw and opisthotonos	項強、四肢抽搐を現し、口の食いしばりおよび後弓反射を現すことすらある病的状態。
柔痙		3.1.193	febrile convulsion without chills	a type of pyretic convulsion associated with sweating but no chills	熱性痙攣の一種で、発汗を付随するが寒戦はない。
剛痙		3.1.194	febrile convulsion with chills	a type of pyretic convulsion associated with chills but no sweating	熱性痙攣の一種で、寒戦を伴うが発汗はない。
痿病		3.1.195	wilting disease	weakness and limpness of the sinews that in severe cases leads to muscular atrophy and prevents the lifting of the legs and arms, the same as atrophy-flaccidity	筋の虚弱化および跛行で、重症例では筋萎縮につながり、脚および腕の挙上を妨げる。痿軟と同義。
肺痿		3.1.196	lung atrophy	a disease of the lung due to chronic cough, marked by atrophy of the lung with shortness of breath and expectoration	慢性の咳嗽による肺疾患。短気および喀痰を伴う肺萎縮を現す。
骨痿	骨痿; 腎痿	3.1.197	bone wilting	wilting attributed to heat in kidney qi, characterized by limp aching lumbar spine and weak wilting lower limbs preventing the patient from getting up and standing, the same as kidney atrophy	腎気の熱による痿。腰椎が痛む跛行、下肢の虚弱・痿による起床、自立の妨げを特徴とする。腎痿と同義。
腎痿	骨痿; 腎痿	3.1.197	bone wilting	wilting attributed to heat in kidney qi, characterized by limp aching lumbar spine and weak wilting lower limbs preventing the patient from getting up and standing, the same as kidney atrophy	腎気の熱による痿。腰椎が痛む跛行、下肢の虚弱・痿による、起床、自立の妨げを特徴とする。骨痿と同義。
脈痿	脈痿; 心痿	3.1.198	vessel wilting	wilting attributed to heat in heart qi, characterized by articular relaxation of the lower limbs preventing the patient from standing, the same as heart atrophy	心気の熱による痿。下肢の関節弛緩による、患者の自立の妨げを特徴とする。心痿と同義。
心痿	脈痿; 心痿	3.1.198	vessel wilting	wilting attributed to heat in heart qi, characterized by articular relaxation of the lower limbs preventing the patient from standing, the same as heart atrophy	心気の熱による痿。下肢の関節弛緩による、患者の自立の妨げを特徴とする。脈痿と同義。
肉痿	肉痿; 脾痿	3.1.199	fleshy wilting	wilting attributed to heat in spleen qi, characterized by numbness of the flesh, and in severe cases, inability to move the limbs, the same as spleen atrophy	脾気の熱による痿。肉の麻木を特徴とし、重症例では四肢の運動が不能である。脾痿と同義。
脾痿	肉痿; 脾痿	3.1.199	fleshy wilting	wilting attributed to heat in spleen qi, characterized by numbness of the flesh, and in severe cases, inability to move the limbs, the same as spleen atrophy	脾気の熱による痿。肉の麻木を特徴とし、重症例では四肢の運動が不能である。肉痿と同義。
筋痿	筋痿; 肝痿	3.1.200	sinew wilting	wilting attributed to heat in liver qi, characterized by sinew contracture gradually giving way to wilting that prevents normal movements, the same as liver atrophy	肝気の熱による痿。筋の拘急を特徴とし、徐々に痿が進み、正常な運動を妨げる。肝痿と同義。

検索表示（原表示の複数語を分離）	IST原表示	Code	Term	Definition/Description	定義/記述
肝痿	筋痿; 肝痿	3.1.200	sinew wilting	wilting attributed to heat in liver qi, characterized by sinew contracture gradually giving way to wilting that prevents normal movements, the same as liver atrophy	肝気の熱による痿。筋の拘急を特徴とし、徐々に痿が進み、正常な運動を妨げる。筋痿と同義。
痿躄		3.1.201	leg flaccidity	loss of muscle tone in the legs due to atrophy and difficulty in legs movement	脚部運動時の萎縮および困難による筋緊張喪失。
顫震		3.1.202	tremor	a diseased state characterized by involuntary trembling of the head or limbs	頭部および四肢の不随意な振戦を特徴とする病的状態。
腰痛		3.1.203	lumbago	pain in the lumbar region caused by disordered qi and blood flow in the related collateral vessels in exogenous afflictions, traumatic injuries or kidney deficiency	外因性疾患、外傷または腎虚で関連する副行血管中の気および血の流れの失調による、腰部の疼痛。
疝	疝; 疝氣	3.1.204	genital disease	a collective term for various diseases characterized by pain and/or swelling of the lower abdomen or scrotum	下腹部または陰嚢の疼痛および／または腫脹を特徴とする多様な疾患の総称。
疝氣	疝; 疝氣	3.1.204	genital disease	a collective term for various diseases characterized by pain and/or swelling of the lower abdomen or scrotum	下腹部または陰嚢の疼痛および／または腫脹を特徴とする多様な疾患の総称。
疝	疝; 小腸氣	3.1.205	hernia	protrusion of a part of an organ or tissue from a body cavity through the tissues that normally contain it	器官または組織の一部が、正常時にはそれを内包する組織を貫通して体腔から突出すること。
小腸氣	疝; 小腸氣	3.1.205	hernia	protrusion of a part of an organ or tissue from a body cavity through the tissues that normally contain it	器官または組織の一部が、正常時にはそれを内包する組織を貫通して体腔から突出すること。
寒疝		3.1.206	cold abdominal colic	acute abdominal pain due to cold, also called cold induced colic	寒による急性腹痛。寒による疝痛とも呼ばれる。
外科		**3.2.0**	**External Medicine**		
瘡瘍		3.2.1	sore and ulcer	a general term for suppurative diseases of the body surface	体表の化膿性疾患に対する一般用語。
瘡		3.2.2	sore	an abbreviation of sore and ulcer	瘡瘍の略。
潰瘍		3.2.3	ulcerating sore	a break in skin or mucous membrane with loss of surface tissue, disintegration and necrosis	表皮組織の喪失、崩壊、壊死を伴う肌膚または粘膜の損傷。
腫瘍		3.2.4	swollen sore	any swelling in external diseases that has not suppurated and ruptured	化膿および破裂を起こしていない外的疾患における腫脹。
髮際瘡		3.2.5	hairline sore	sore occurring at the back of the neck close to the hairline	生え際近くの後頸部に発生した瘡。
坐板瘡		3.2.6	seat sore	sore on the buttock	臀部の瘡。
臁瘡		3.2.7	shank sore	chronic ulcer on the lower leg	下腿部の慢性潰瘍。
褥瘡		3.2.8	bedsore	an ulceration caused by prolonged pressure in a patient lying in bed for a long period of time	長期間寝台に横臥していた患者にみられる、長期の圧迫により起こる潰瘍。
癤		3.2.9	furuncle	an acute localized inflammation of the skin, having a hard central core, and forming pus, also known as boil	肌膚の局在性の急性炎症で、固い中心核があり、膿を形成する。おできとしても知られる。
螻蛄癤		3.2.10	mole cricket boil disease	multiple abscesses of the scalp, referring to folliculitis abscedens et suffodiens	頭部の多発性膿瘍。膿瘍性穿掘性毛包炎を表す。
癤病		3.2.11	furunculosis	a condition of tending to develop multiple furuncles	多発性癤発症の傾向がある状態。
疔		3.2.12	deep-rooted boil	a boil with its central core deeply rooted	深く根付いた中心核のあるおでき。
蛇頭疔		3.2.13	snake-head whitlow	a swollen fingertip resembling the head of a snake, referring to digital pyogenic inflammation	蛇の頭部に似た、指先の腫脹。指化膿性炎症を表す。
疫疔		3.2.14	cutaneous anthrax	an infectious disease characterized by small pruritic papular lesions of the head, face and limbs, which enlarge, ulcerate, discharge bloody serum, associated with severe systemic symptoms such as septicemia, high fever, vomiting, sweating, headache and prostration	感染症の一つで、頭部、顔、四肢に、肥大化、潰瘍化し、血液の混じった血清を排出する小型で掻痒性の丘疹病変の発現を特徴とする。敗血症、壮熱、嘔吐、発汗、頭痛、衰弱など重度の全身性症状を伴う。
癰		3.2.15	abscess	a pyogenic infection with localized collection of pus buried in tissues or organs	化膿性感染症の一つで組織または器官に埋没した膿の局所集中を伴う。
頸癰		3.2.16	cervical abscess	a pyogenic infection with abscess formation at the lateral aspect of the neck	頸部側面の癰形成を伴う化膿性感染症。
腋癰		3.2.17	axillary abscess	a pyogenic infection with abscess formation in the axillary region	腋窩部の癰形成を伴う化膿性感染症。
委中毒		3.2.18	popliteal infection	an acute pyogenic inflammation in the popliteal region	膝窩部の化膿性急性炎。
臍癰		3.2.19	umbilical abscess	a pyogenic infection with abscess formation in the umbilical region	臍部の癰形成を伴う化膿性感染症。
臀癰		3.2.20	gluteal abscess	a pyogenic infection with abscess formation in the gluteal region	殿溝部の癰形成を伴う化膿性感染症。
腸癰		3.2.21	intestinal abscess	a localized pyogenic inflammation of the intestine, referring to acute appendicitis	腸の局在性の化膿性炎症。急性盲腸炎を表す。
發		3.2.22	effusion	acute, diffuse and suppurative inflammation of the subcutaneous tissue	皮下組織の急性びまん性化膿性感染症。
手發背		3.2.23	effusion of the back of the hand	acute pyogenic infection of the dorsum of the hand with diffuse swelling and inflammation	びまん性腫脹および炎症を伴う手背部の急性化膿性感染症。
足發背		3.2.24	effusion of the dorsum of the foot	acute pyogenic infection of the dorsum of the foot with diffuse swelling and inflammation	びまん性腫脹および炎症を伴う足背部の急性化膿性感染症。
有頭疽		3.2.25	carbuncle	a necrotizing infection of skin and subcutaneous tissue with multiple openings for the discharge of pus and sloughing of dead tissue	膿排出および死亡組織の脱落のため複数の開口部を伴う肌膚および皮下組織の壊死性感染症。
附骨疽		3.2.26	suppurative osteomyelitis	a suppurative inflammatory disease of bone, marked by local death and separation of tissue	組織の局所的な死亡および分離を現す、骨の化膿性炎症性疾患。
環跳疽		3.2.27	suppurative coxitis	suppurative inflammation of the hip joint	股関節の化膿性炎症。
脫疽		3.2.28	digital gangrene	gangrene of the extremities, especially referring to thromboangiitis	四肢の壊疽。特に血栓性血管炎を表す。
流注		3.2.29	deep multiple abscess	a secondary abscess, usually multiple, deeply located, and distant from the primary lesion, also called metastatic abscess	二次性の癰。通常、多発性、深部局在、原発病変から遠位である。転移性癰とも呼ばれる。
發頤		3.2.30	suppurative parotitis	inflammation of the parotid gland associated with suppuration	化膿に関連する耳下腺炎。
丹毒	丹毒; 火丹	3.2.31	erysipelas	an acute infection of the skin marked by intense local redness	強い局所発赤を特徴とする肌膚の急性感染。

検索表示（原表示の複数語を分離）	IST原表示	Code	Term	Definition/Description	定義/記述
火丹	丹毒; 火丹	3.2.31	erysipelas	an acute infection of the skin marked by intense local redness	強い局所発赤を特徴とする肌膚の急性感染。
赤遊丹		3.2.32	wandering erysipelas	a type of erysipelas in the newborn characterized by changing the location of the lesion either from the trunk to the extremities or from the extremities to the trunk	新生児における丹毒の一種で、体幹から四肢へ、または四肢から体幹への病変位置の変化を特徴とする。
流痰	流痰; 骨癆	3.2.33	flowing phlegm	chronic pyogenic infection, especially tuberculosis, of bone and joint, also known as osteoarticular tuberculosis	特に結核による、骨および関節の慢性化膿性感染。骨関節結核としても知られる。
骨癆	流痰; 骨癆	3.2.33	flowing phlegm	chronic pyogenic infection, especially tuberculosis, of bone and joint, also known as osteoarticular tuberculosis	特に結核による、骨および関節の慢性化膿性感染。骨関節結核としても知られる。
瘰癧		3.2.34	scrofula	chronic inflammation of the cervical lymph nodes	頸部リンパ節の慢性炎症。
乳癰		3.2.35	acute mastitis	acute pyogenic inflammation of the breast	乳房の急性化膿性炎症。
內吹乳癰		3.2.36	mastitis during pregnancy	inflammation of the breast occurring during pregnancy	妊娠中に発生する乳房の炎症。
外吹乳癰		3.2.37	postpartum mastitis	inflammation of the breast occurring after delivery	分娩後に発生する乳房の炎症。
乳發		3.2.38	phlegmonous mastitis	a serious suppurative inflammation of the breast leading to necrosis and abscess formation	乳房の重篤な化膿性炎症で、壊死、癰形成をもたらす。
乳癆	乳癆; 乳痰	3.2.39	mammary phthisis	tuberculosis of the breast	乳房の結核。
乳痰	乳癆; 乳痰	3.2.39	mammary phthisis	tuberculosis of the breast	乳房の結核。
乳癧		3.2.40	gynecomastia	excessive development of the breast in the children and male adults	小児および成人男性における乳房過剰発達。
乳漏		3.2.41	mammary fistula	an abnormal passage that leads to the cutaneous surface of the breast or areola of the nipple and exudes pus	乳房の皮膚表面または乳輪に開通し膿を滲出する異常排出。
乳衄		3.2.42	thelorrhagia	bleeding from the mammary nipple	乳首からの出血。
乳癖		3.2.43	mammary hyperplasia	benign hyperplasia of mammary gland	乳腺の良性過形成。
乳頭風		3.2.44	cracked nipple	painful fissuring of the mammary nipple or areola	乳首または乳輪の有痛性の亀裂。
癭		3.2.45	goiter	enlargement of the thyroid gland, causing a swelling in the front part of the neck	前頸部の腫脹を起こす甲状腺肥大。
氣癭		3.2.46	qi goiter	a goiter that is painless and soft to the touch and that swells and shrinks with the patient’s moods	無痛で触わると軟らかく患者の気とともに腫脹および縮小する甲状腺腫。
肉癭		3.2.47	fleshy goiter	a benign goiter, soft or beefy, with a slow progress, mostly referring to adenomatous or cystic goiter	進行が遅く、軟らかいまたは牛肉様の良性甲状腺腫。主に腺腫様甲状腺腫または嚢胞性甲状腺腫を表す。
石癭		3.2.48	stony goiter	enlarged thyroid, nodulated and as hard as stone, mostly referring to carcinoma of the thyroid	結節性で石状に固い甲状腺肥大。主に甲状腺癌を表す。
瘤		3.2.49	tumor	a neoplasm which persists and has no physiological use	持続性で生理学上の用途を持たない新生物。
氣瘤		3.2.50	qi tumor	a term for multiple pedunculated soft tumors arising superficially under the skin, becoming flat on pressing and bulging again when the pressure is removed as if they were filled with air, mostly referring to neurofibroma	表面付近の皮下で発生する、多発性で軟性の有茎性腫瘍に対する用語で、押すと平坦になり、離すと空気が入っているかのように膨隆する。主に神経線維腫を表す。
血瘤		3.2.51	blood tumor	a vascular tumor composed of dilated blood vessels in the skin or subcutaneously	表皮内または皮下の拡張血管からなる血管腫瘍。
筋瘤		3.2.52	sinew tumor	a dilated and tortuous vein, usually in the leg	通常脚部の拡張および蛇行静脈。
肉瘤		3.2.53	fleshy tumor	tumor of subcutaneous fatty tissue	皮下脂肪組織の腫瘍。
骨瘤		3.2.54	bone tumor	tumor of the bone, either benign or malignant	良性または悪性の骨腫瘍。
巖		3.2.55	rocky mass/cancer	any malignant tumor occurring on body surface that looks and feels like rock	外見および感触が岩状の、体表上に発生する悪性腫瘍。
繭脣		3.2.56	cocoon lip	malignant tumor occurring in the labial region	唇部に発生する悪性腫瘍。
失榮		3.2.57	cervical malignancy with cachexia	advanced case of malignant tumor of the cervical lymph node, either primary or metastatic, accompanied by cachexia	頸部リンパ節の悪性腫瘍が進行した症例で、原発性または転移性のいずれかであり、悪液質が付随する。
乳巖		3.2.58	rocky mass in the breast/breast cancer	malignant tumor occurring in the mammary region	乳房部に発生する悪性腫瘍。
疹		3.2.59	eruption	visible efflorescent lesions of the skin, resembling millet seeds, and marked by redness and prominence	粟粒状の、目視で確認できる肌膚の発疹病変。発赤および隆起を特徴とする。
斑疹		3.2.60	macula	a general term for any spot or area distinguishable by color from the surrounding skin	色により周囲の肌膚と識別可能な、斑または部位の一般用語。
丘疹		3.2.61	papule	small conical elevation of the skin	小型で円錐形の皮膚の挙上。
膿皰		3.2.62	pustule	small circumscribed elevation of the skin, containing pus	小型で境界明確な皮膚の挙上で、膿を内包する。
痂		3.2.63	scab	outer layer of wound formed by the drying of a bodily exudate	体の浸出液により形成される創の外層。
結核		3.2.64	subcutaneous node	a general term for any round mass formed under the skin	皮下に形成される円形の腫瘤の一般用語。
息肉		3.2.65	polyp	a protruding growth from the mucous membrane	粘膜由来の突出した増殖。
漏		3.2.66	fistula	an abnormal passage leading from an abscess or hollow organ to the body surface	膿瘍または中空器官から体表につながる異常な開通。
熱瘡		3.2.67	herpes simplex	the development of fluid-filled vesicles on the margin of skin and mucous membrane as a concomitant of fever, usually referring to herpes simplex	肌膚および粘膜辺縁に液体を含む小胞を発症し、同時に発熱を伴う。通常は単純疱疹を表す。
蛇丹	蛇丹;蛇串瘡; 纏腰火丹; 纏腰蛇丹; 火帶瘡	3.2.68	herpes zoster	an acute eruptive disease characterized by severe pain along the girdled distribution of clustered vesicles	集簇性小胞の帯状分布に沿った劇痛を特徴とする、急性発疹性疾患。

検索表示（原表示の複数語を分離）	IST原表示	Code	Term	Definition/Description	定義/記述
蛇串瘡	蛇丹;蛇串瘡; 纏腰火丹; 纏腰蛇丹; 火帯瘡	3.2.68	herpes zoster	an acute eruptive disease characterized by severe pain along the girdled distribution of clustered vesicles	集簇性小胞の帯状分布に沿った劇痛を特徴とする、急性発疹性疾患。
纏腰火丹	蛇丹;蛇串瘡; 纏腰火丹; 纏腰蛇丹; 火帯瘡	3.2.68	herpes zoster	an acute eruptive disease characterized by severe pain along the girdled distribution of clustered vesicles	集簇性小胞の帯状分布に沿った劇痛を特徴とする、急性発疹性疾患。
纏腰蛇丹	蛇丹;蛇串瘡; 纏腰火丹; 纏腰蛇丹; 火帯瘡	3.2.68	herpes zoster	an acute eruptive disease characterized by severe pain along the girdled distribution of clustered vesicles	集簇性小胞の帯状分布に沿った劇痛を特徴とする、急性発疹性疾患。
火帯瘡	蛇丹;蛇串瘡; 纏腰火丹; 纏腰蛇丹; 火帯瘡	3.2.68	herpes zoster	an acute eruptive disease characterized by severe pain along the girdled distribution of clustered vesicles	集簇性小胞の帯状分布に沿った劇痛を特徴とする、急性発疹性疾患。
疣		3.2.69	verruca	a horny projection on the skin, also known as a wart	肌膚上の角化突起。疣贅としても知られる。
疣目		3.2.70	verruca vulgaris	a lobulated hyperplastic epidermal lesion with a horny surface, usually occurring on the back of the hand, fingers or scalp	表面が角化した、小葉状過形成上皮病変。通常手甲部、指あるいは頭部に発生する。
鶏眼	鶏眼; 肉刺	3.2.71	corn	a horny thickening of the skin, forming a conical mass pointing down into the corium (dermis)	肌膚の角化肥厚で、真皮に食い込む円錐状腫瘤を形成する。
肉刺	鶏眼; 肉刺	3.2.71	corn	a horny thickening of the skin, forming a conical mass pointing down into the corium (dermis)	肌膚の角化肥厚で、真皮に食い込む円錐状腫瘤を形成する。
肥瘡		3.2.72	fat sore	a type of ringworm characterized by formation of yellow cup-shaped crusts, referring to tinea favosa	たむしの一種で黄色の茶碗形の痂皮形成を特徴とする。黄癬を表す。
鵝掌風		3.2.73	tinea manuum	ringworm affecting the hands, referring to chronic dermatitis with keratosis	手に罹患したたむし。角化症を伴う慢性皮膚炎を表す。
紫白癜風		3.2.74	tinea versicolor	a non-inflammatory disorder characterized by multiple macular patches of all sizes and shapes, varying from white in pigmented skin to tan or brown in pale skin, usually seen on the neck, trunk and proximal ends of limbs that are apt to sweat	すべての大きさ・形状の多発性黄斑を特徴とする非炎症性疾患。色素沈着した肌膚の白色斑から、肌膚色が薄い皮膚の黒ずんだまたは褐色斑まで、多様である。通常、頸部、体幹、発汗しやすい四肢末端にみられる。
白癜風	白癜風; 白駁風	3.2.75	vitiligo	a skin disease manifested as smooth white spots on various parts of the body	体の多様な部位に平滑な白色斑が発現する肌膚疾患。
白駁風	白癜風; 白駁風	3.2.75	vitiligo	a skin disease manifested as smooth white spots on various parts of the body	体の多様な部位に平滑な白色斑が発現する肌膚疾患。
疥瘡	疥瘡; 疥癩	3.2.76	scabies	a contagious skin disease caused by mites	ダニによる伝染性肌膚疾患。
疥癩	疥瘡; 疥癩	3.2.76	scabies	a contagious skin disease caused by mites	ダニによる伝染性肌膚疾患。
癮疹	癮疹; 風癮疹	3.2.77	urticaria	an allergic disorder of the skin, marked by red or pale wheals, intermittent, associated with intense itching	赤色または蒼白の膨疹を現す、間欠性のアレルギー性肌膚疾患。強い掻痒を伴う。
風癮疹	癮疹; 風癮疹	3.2.77	urticaria	an allergic disorder of the skin, marked by red or pale wheals, intermittent, associated with intense itching	赤色または蒼白の膨疹を現す、間欠性のアレルギー性肌膚疾患。強い掻痒を伴う。
乾癬		3.2.78	dry ringworm	a chronic skin disease characterized by clearly circumscribed thickening of the skin, fissuring and itching that causes scaling, mostly referring to chronic eczema or neurodermatitis	境界明確な肌膚肥厚、亀裂、落屑を起こす掻痒を特徴とする慢性肌膚疾患。主に慢性湿疹または神経皮膚炎を表す。
牛皮癬		3.2.79	oxhide lichen	chronic disorder of the skin characterized by patches of itching lichenoid eruption resembling cattle hide, referring to neurodermatitis	牛皮革に似た、痒みのある苔癬様の発疹の斑点を特徴とする慢性肌膚疾患。神経皮膚炎を表す。
白疕	白疕; 松皮癬	3.2.80	psoriasis	a chronic skin disease marked by rounded, circumscribed, erythematous, dry, scaling patches, covered by silvery white, lamellar scales, resembling the bark of a pine tree	松の木の皮に似た、円形、境界明確、紅斑性、乾燥、落屑斑、銀白色に被覆、葉状落屑を現す慢性肌膚疾患。
松皮癬	白疕; 松皮癬	3.2.80	psoriasis	a chronic skin disease marked by rounded, circumscribed, erythematous, dry, scaling patches, covered by silvery white, lamellar scales, resembling the bark of a pine tree	松の木の皮に似た、円形、境界明確、紅斑性、乾燥、落屑斑、銀白色に被覆、葉状落屑を現す慢性肌膚疾患。
面遊風		3.2.81	seborrheic dermatitis	a chronic inflammation of the skin marked by excessive secretion of sebum	皮脂過剰分泌を現す慢性肌膚疾患。
粉刺	粉刺; 酒刺	3.2.82	acne	a chronic inflammatory disease of the follicles and sebaceous glands, occurring on the face, chest and back	顔面、胸部、背部に発生する毛包および皮脂腺の慢性炎症性疾患。
酒刺	粉刺; 酒刺	3.2.82	acne	a chronic inflammatory disease of the follicles and sebaceous glands, occurring on the face, chest and back	顔面、胸部、背部に発生する毛包および皮脂腺の慢性炎症性疾患。
油風		3.2.83	alopecia	a disease marked by sudden patchy loss of hair, which usually occurs in sharply defined areas, referring to alopecia areata	突発的な毛髪の斑状喪失を現す疾患で、通常は境界明瞭な範囲で発生する。円形脱毛症を表す。
貓眼瘡		3.2.84	erythema multiforme	an acute self-limited inflammatory skin disease characterized by sudden onset of erythematous macular, bullous, papular, or vesicular eruption	紅斑性斑状、水疱性、丘疹または小胞性発疹の突発を特徴とする急性自己限定炎症性肌膚疾患。
瓜藤纏		3.2.85	erythema nodosum	a skin disease which most often affects young women and is characterized by the development of crops of transient, inflammatory nodules that are usually tender, multiple, and bilateral, most commonly located on the shins	若年女性にきわめてよくみられる肌膚疾患で、一時的な炎症性結節の病変発症を特徴とする。通常は圧痛があり多発性で両側性であり、主に脛骨前部に局在する。

検索表示（原表示の複数語を分離）	IST原表示	Code	Term	Definition/Description	定義/記述
皮痹		3.2.86	skin impediment	a disease characterized by hardening and thickening of the skin, followed by atrophy at the late stage, referring to scleroderma	肌膚の硬化および肥厚化を特徴とする疾患で、後期には萎縮が後続する。強皮症を表す。
痔		3.2.87	hemorrhoid	varicose dilatation of a vein of the superior or inferior hemorrhoidal plexus	表または裏の直腸静脈叢の静脈の静脈瘤拡張。
內痔		3.2.88	internal hemorrhoid	varicose dilatation of a vein of the superior hemorrhoidal plexus, situated distal to the pectinate line	表の直腸静脈叢の静脈の静脈瘤拡張。櫛状線近位に位置する。
外痔		3.2.89	external hemorrhoid	varicose dilatation of a vein of the inferior hemorrhoidal plexus, situated distal to the pectinate line	裏の直腸静脈叢の静脈の静脈瘤拡張。櫛状線遠位に位置する。
肛漏	肛漏;<br>肛瘻	3.2.90	anal fistula	a fistula opening on the cutaneous surface near the anus, which may communicate with the rectum	肛門付近の表皮に開口した漏。直腸とつながっている場合がある。
肛瘻	肛漏;<br>肛瘻	3.2.90	anal fistula	a fistula opening on the cutaneous surface near the anus, which may communicate with the rectum	肛門付近の表皮に開口した漏。直腸とつながっている場合がある。
脫肛		3.2.91	prolapse of the rectum	protrusion of the rectal mucous membrane through the anus	肛門を通った直腸粘膜の突出。
息肉痔		3.2.92	polyp of rectum	a protruding growth from the mucous membrane of the rectum	直腸粘膜由来の突出性増殖。
子癰		3.2.93	abscess of the testicle	an inflammatory infection of the testis and epididymis marked by local pain and swelling, referring to epididymitis and orchitis	精巣および精巣上体の炎症性感染症。局所的疼痛および腫脹を特徴とする。精巣上体炎および精巣炎を表す。
囊癰		3.2.94	scrotal abscess	an acute pyogenic inflammation of the scrotum excluding the testis, marked by redness, swelling and pain of the scrotum associated with chills and fever, and followed by tightness and luster of the scrotal skin with excruciating pain	精巣を除く陰嚢の急性発熱性炎症。発赤、腫脹、悪寒および発熱と関連する陰嚢の疼痛を特徴とし、非常に激しい痛みを伴う陰嚢の肌膚の硬化および光沢が後続する。
水疝		3.2.95	hydrocele	an accumulation of fluid in the testicle	精巣の津の蓄積。
凍瘡	凍瘡;<br>凍風	3.2.96	frostbite	a lesion marked by swelling, numbness, cyanosis, blister or ulcerating sore attributed to exposure to cold that causes local stagnation of qi and blood	腫脹、しびれ、チアノーゼ、水疱または潰瘍を現す病変。局所的な気血の滞留を起こす寒への曝露による。
凍風	凍瘡;<br>凍風	3.2.96	frostbite	a lesion marked by swelling, numbness, cyanosis, blister or ulcerating sore attributed to exposure to cold that causes local stagnation of qi and blood	腫脹、しびれ、チアノーゼ、水疱または潰瘍を現す病変。局所的な気血の滞留を起こす寒への曝露による。
破傷風	破傷風;<br>金瘡痓	3.2.97	tetanus	a disease characterized by sustained muscular contraction caused by pathogenic wind toxin which enters the body through a puncture wound	持続性の筋肉硬直を特徴とする疾患。破傷から体内に入った風邪毒による。
金瘡痓	破傷風;<br>金瘡痓	3.2.97	tetanus	a disease characterized by sustained muscular contraction caused by pathogenic wind toxin which enters the body through a puncture wound	持続性の筋肉硬直を特徴とする疾患。破傷から体内に入った風邪毒による。
青蛇毒		3.2.98	green-blue snake toxin sore	inflammation of a superficial vein associated with thrombus formation, referring to superficial thrombophlebitis	血栓形成に付随する表皮静脈の炎症。表在性血栓性静脈炎を表す。
股腫		3.2.99	thigh swelling	thrombus formation and inflammation of a deep-located vein, referring to deep thrombophlebitis	深部に局在する静脈の血栓形成および炎症。深部血栓性静脈炎を表す。
狐惑	狐惑;<br>狐惑病	3.2.100	fox-creeper disease	a disease characterized by erosion of the mouth, throat and genitalia, red eyes and black canthi, resembling Behcet's syndrome	口、咽喉頭、生殖器のびらん、眼充血、黒色の眼角を特徴とする疾患。ベーチェット症候群に類似。
狐惑病	狐惑;<br>狐惑病	3.2.100	fox-creeper disease	a disease characterized by erosion of the mouth, throat and genitalia, red eyes and black canthi, resembling Behcet's syndrome	口、咽喉頭、生殖器のびらん、眼充血、黒色の眼角を特徴とする疾患。ベーチェット症候群に類似。
婦産科; 産婦人科		**3.3.0**	**Gynecology and Obstetrics**		
並月		3.3.1	bimonthly menstruation	menstruation occurring once every two months, but causing no symptoms	2ヵ月に1回の月経。しかし無症状。
居經	居經;<br>季經	3.3.2	trimonthly menstruation	menstruation occurring once every three months, but causing no symptoms	3ヵ月に1回の月経。しかし無症状。
季經	居經;<br>季經	3.3.2	trimonthly menstruation	menstruation occurring once every three months, but causing no symptoms	3ヵ月に1回の月経。しかし無症状。
避年		3.3.3	annual menstruation	menstruation occurring once a year, but causing no symptoms	1年に1回の月経。しかし無症状。
五不女		3.3.4	five unwomanlinesses	five forms of female infertility	女性の不妊症の5形態。
激經	激經;<br>垢胎	3.3.5	menstruation during pregnancy	regular menstruation during early pregnancy, which stops spontaneously when the fetus is fully developed	妊娠初期の定期的な月経。胎児が十分発育すれば自然に停止する。
垢胎	激經;<br>垢胎	3.3.5	menstruation during pregnancy	regular menstruation during early pregnancy, which stops spontaneously when the fetus is fully developed	妊娠初期の定期的な月経。胎児が十分発育すれば自然に停止する。
臨產		3.3.6	labor	the process of giving birth to offspring, including the expulsion of the fetus, placenta and membranes from the uterus through the vagina	子の出産の過程。子宮から膣を経由した、胎児、胎盤、子宮内膜の排出を含める。
試胎	試胎;<br>試月	3.3.7	testing labor	abdominal pain that occurs for a short time and then stops, occurring during the eighth or ninth month of pregnancy	妊娠第8ヵ月または9ヵ月に起こる、短時間の発生の後に止まる腹痛。
試月	試胎;<br>試月	3.3.7	testing labor	abdominal pain that occurs for a short time and then stops, occurring during the eighth or ninth month of pregnancy	妊娠第8ヵ月または9ヵ月に起こる、短時間の発生の後に止まる腹痛。
弄胎		3.3.8	false labor	intermittent abdominal pain that occurs toward the end of the term of pregnancy without backache, also known as agitated fetus	妊娠満期にかけて発生する、背部痛を伴わない間欠性の下腹部痛。煩胎としても知られる。
試水		3.3.9	early leakage of amniotic fluid	the condition characterized by leakage of the amniotic fluid which is not followed by childbirth	羊水は漏出するが出産が後続しないことを特徴とする状態。
傷產		3.3.10	injured labor	premature delivery due to traumatic injury	外傷による、満期以前の分娩。
產褥		3.3.11	puerperium	the period between childbirth and the return of the uterus to its normal condition	出産から子宮が正常な状態に戻るまでの期間。
月經病		3.3.12	menstrual disease	a collective term for diseases characterized by abnormal interval, duration, quantity, color, quality of menses, non-physiological stoppage, as well as manifest symptoms with the periods or around menopause, the same as emmeniopathy	月経の間隔、期間、経血量、経血の色、質の異常、非生理的な月経停止、月経期間中または更年期前後の症状発現を特徴とする疾患の一般用語。月経異常と同義。

検索表示（原表示の複数語を分離）	IST原表示	Code	Term	Definition/Description	定義/記述
月經不調		3.3.13	menstrual irregularities	a general term for irregular menstruation and other menstrual complaints, such as abnormal duration, amount, color and quality of menstrual discharge	不定期な月経および他の月経愁訴(月経の期間、経血量、経血の色、質の異常など)に対する一般用語。
月經先期	月經先期; 經行先期; 經早	3.3.14	advanced menstruation	periods that come one week or more ahead of due time, for more than two successive periods	2周期を超えて連続した、予定より1週間以上早い月經。
經行先期	月經先期; 經行先期; 經早	3.3.14	advanced menstruation	periods that come one week or more ahead of due time, for more than two successive periods	2周期を超えて連続した、予定より1週間以上早い月經。
經早	月經先期; 經行先期; 經早	3.3.14	advanced menstruation	periods that come one week or more ahead of due time, for more than two successive periods	2周期を超えて連続した、予定より1週間以上早い月經。
月經後期	月經後期; 經行後期; 經遲	3.3.15	delayed menstruation	periods that come one week or more after due time, for more than two successive periods	2周期を超えて連続した、予定より1週間以上遅れた月經。
經行後期	月經後期; 經行後期; 經遲	3.3.15	delayed menstruation	periods that come one week or more after due time, for more than two successive periods	2周期を超えて連続した、予定より1週間以上遅れた月經。
經遲	月經後期; 經行後期; 經遲	3.3.15	delayed menstruation	periods that come one week or more after due time, for more than two successive periods	2周期を超えて連続した、予定より1週間以上遅れた月經。
月經先後無定期	月經先後無定期; 經亂	3.3.16	menstruation at irregular intervals	periods that come with an irregular cycle, more than one week early or later	予定より1週間を超えて前後した、不定周期の月經。
經亂	月經先後無定期; 經亂	3.3.16	menstruation at irregular intervals	periods that come with an irregular cycle, more than one week early or later	予定より1週間を超えて前後した、不定周期の月經。
月經過少	月經過少; 月經澁少	3.3.17	scant menstruation	menstrual discharge of less than the normal amount occurring at regular intervals	一定周期であるが経血量が通常より少ない月經。
月經澁少	月經過少; 月經澁少	3.3.17	scant menstruation	menstrual discharge of less than the normal amount occurring at regular intervals	一定周期であるが経血量が通常より少ない月經。
月經過多	月經過多; 經水過多; 月水過多	3.3.18	profuse menstruation	excessive uterine bleeding occurring at regular intervals, the same as menorrhagia	一定周期であるが過多の子宮出血。
經水過多	月經過多; 經水過多; 月水過多	3.3.18	profuse menstruation	excessive uterine bleeding occurring at regular intervals, the same as menorrhagia	一定周期であるが過多の子宮出血。月経過多と同義。
月水過多	月經過多; 經水過多; 月水過多	3.3.18	profuse menstruation	excessive uterine bleeding occurring at regular intervals, the same as menorrhagia	一定周期であるが過多の子宮出血。月経過多と同義。
崩漏		3.3.19	flooding and spotting	sudden onset of profuse uterine bleeding or incessant dripping of blood, occurring not in the regular menstruation period, the same as metrorrhagia	定期の月経期間外に発生した、大量の子宮出血または間断のない漏血の突発。不正子宮出血と同義。
血崩	血崩; 崩中	3.3.20	flooding	sudden massive uterine bleeding, the same as metrorrhagia	突然の子宮からの大量出血。不正子宮出血と同義。
崩中	血崩; 崩中	3.3.20	flooding	sudden massive uterine bleeding, the same as metrorrhagia	突然の子宮からの大量出血。不正子宮出血と同義。
漏下		3.3.21	spotting	slight but persistent leakage of blood from the uterus, the same as metrostaxis	少量であるが持続的な子宮出血。子宮漏血と同義。
經期延長		3.3.22	prolonged menstruation	excessively prolonged menstruation in regular cycles, the same as menostaxis	一定周期であるが過度に長期の月經。子宮漏血と同義。
經間期出血		3.3.23	intermenstrual bleeding	uterine bleeding occurring not in the regular menstruation period	定期の月経期間外に発生した子宮出血。
閉經	閉經; 經閉	3.3.24	amenorrhea	failure of menstruation to occur at puberty or abnormal stoppage of the menses for more than three months after menarche	思春期に発生する月経不全または初潮後3か月を超えた月経異常停止。
經閉	閉經; 經閉	3.3.24	amenorrhea	failure of menstruation to occur at puberty or abnormal stoppage of the menses for more than three months after menarche	思春期に発生する月経不全または初潮後3か月を超えた月経異常停止。
痛經	痛經; 月經痛; 經行腹痛	3.3.25	dysmenorrhea	lower abdominal pain or referring pain to the lower back occurring around or during the menstrual period	月経期間前後または期間中の下腹部痛または下背部の関連痛。

検索表示（原表示の複数語を分離）	IST原表示	Code	Term	Definition/Description	定義/記述
月經痛	痛經; 月經痛; 經行腹痛	3.3.25	dysmenorrhea	lower abdominal pain or referring pain to the lower back occurring around or during the menstrual period	月経期間前後または期間中の下腹部痛または下背部の関連痛。
經行腹痛	痛經; 月經痛; 經行腹痛	3.3.25	dysmenorrhea	lower abdominal pain or referring pain to the lower back occurring around or during the menstrual period	月経期間前後または期間中の下腹部痛または下背部の関連痛。
倒經	倒經; 逆經	3.3.26	inverted menstruation	discharge of blood from some part other than the vagina during the menstrual period, such as bleeding from the nose or vomiting of blood	鼻血または嘔血など、月経期間中の膣以外の部位からの出血。
逆經	倒經; 逆經	3.3.26	inverted menstruation	discharge of blood from some part other than the vagina during the menstrual period, such as bleeding from the nose or vomiting of blood	鼻血または嘔血など、月経期間中の膣以外の部位からの出血。
經行乳房脹痛		3.3.27	distending pain in the breasts during menstruation	distention or sensation of fullness and pain in the breasts before, during, or after menstruation	月経期間前、期間中、期間後の乳房の脹または充満感および疼痛。
經行情志異常		3.3.28	moodiness during menstruation	depression, gloominess, irritability and other changes of mood occurring around each menstrual period and returning to normal after the period	各月経期間前後に発生する、鬱病、陰気、易刺激性およびその他の気分の変化。
帶下		3.3.29	vaginal discharge	(1) normal discharge from the vagina; (2) diseases characterized by discharge from the vagina, the same as leukorrhea	(1)膣からの正常な分泌物。(2)膣からの分泌物を特徴とする疾患。白帯下と同義。
白帶		3.3.30	white vaginal discharge	(1) whitish discharge from the vagina; (2) a disease marked by white discharge from the vagina	(1)膣からの白みがかった分泌物。(2)膣からの白色分泌物を特徴とする疾患。
黃帶		3.3.31	yellow vaginal discharge	yellowish viscous discharge from the vagina, usually indicating the presence of pathogenic damp-heat	膣からの黄色がかった粘性のある分泌物。通常湿熱邪の存在を示す。
惡阻	惡阻; 妊娠惡阻	3.3.32	morning sickness	nausea and vomiting during early pregnancy	妊娠初期の悪心、嘔吐。
妊娠惡阻	惡阻; 妊娠惡阻	3.3.32	morning sickness	nausea and vomiting during early pregnancy	妊娠初期の悪心、嘔吐。
胞阻	胞阻; 妊娠腹痛	3.3.33	uterine obstruction	lower abdominal pain occurring in pregnancy, usually due to the impeded flow of qi and blood in uterine vessels	妊娠中に起こる下腹部痛。通常、子宮血管中の気血の流れの妨害による。
妊娠腹痛	胞阻; 妊娠腹痛	3.3.33	uterine obstruction	lower abdominal pain occurring in pregnancy, usually due to the impeded flow of qi and blood in uterine vessels	妊娠中に起こる下腹部痛。通常、子宮血管中の気血の流れの妨害による。
胎漏		3.3.34	vaginal bleeding during pregnancy	small amount of blood discharge through vagina during pregnancy, intermittent, but with no lumbar pain or abdominal pain	妊娠中の膣からの少量出血。間欠性であるが、腰痛、腹痛は伴わない。
胎動不安		3.3.35	threatened abortion	continuous moving of the fetus with lumbar pain and abdominal pain or small amount of vaginal bleeding	腰痛および腹痛、または少量の膣出血を伴う継続的な胎動。
滑胎		3.3.36	habitual abortion	spontaneous abortion in three or more consecutive pregnancies	3回以上連続した妊娠における自然流産。
墮胎		3.3.37	early abortion	spontaneous abortion occurring within the first twelve weeks of pregnancy	妊娠第12週以内に発生する自然流産。
小産		3.3.38	late abortion	spontaneous abortion occurring after the twelfth week and before the twenty-eighth week of pregnancy	妊娠第12週の後〜第27週に発生する自然流産。
鬼胎		3.3.39	pseudopregnancy	abnormal pregnancy resulting in a mass in the uterus resembling a bunch of grapes, referring to hydatidiform mole	子宮内にブドウの房状の腫瘤を生じさせる異常妊娠。胞状奇胎を表す。
子滿	子滿; 胎水腫滿	3.3.40	hydramnios	that causing abnormally enlarged abdomen, sensation of fullness and dyspnea	異常な腹部肥大、充満感、喘を起こすもの。
胎水腫滿	子滿; 胎水腫滿	3.3.40	hydramnios	that causing abnormally enlarged abdomen, sensation of fullness and dyspnea	異常な腹部肥大、充満感、喘を起こすもの。
子腫	子腫; 妊娠腫脹	3.3.41	pregnancy swelling	edema of the face and limbs occurring in the late stage of pregnancy	妊娠後期に発生する顔面および四肢の水腫。
妊娠腫脹	子腫; 妊娠腫脹	3.3.41	pregnancy swelling	edema of the face and limbs occurring in the late stage of pregnancy	妊娠後期に発生する顔面および四肢の水腫。
子煩	子煩; 妊娠心煩	3.3.42	pregnancy vexation	depression or irritability occurring during pregnancy	妊娠中に発生する鬱病または易刺激性。
妊娠心煩	子煩; 妊娠心煩	3.3.42	pregnancy vexation	depression or irritability occurring during pregnancy	妊娠中に発生する鬱病または易刺激性。
子暈	子暈; 妊娠眩暈	3.3.43	dizziness in pregnancy	dizziness with blurred vision or even fainting occurring in pregnancy, the same as gravid vertigo	妊娠中に発生する目昏を伴う浮動性めまいで、昏厥を伴うことすらある。妊娠性めまいと同義。
妊娠眩暈	子暈; 妊娠眩暈	3.3.43	dizziness in pregnancy	dizziness with blurred vision or even fainting occurring in pregnancy, the same as gravid vertigo	妊娠中に発生する目昏を伴う浮動性めまいで、昏厥を伴うことすらある。妊娠性めまいと同義。
子懸	子懸; 胎氣上逆	3.3.44	pregnancy suspension	feeling of pressure in the abdomen and thorax, even with dyspnea and irritability, during pregnancy, the same as gravid oppression or upward flow of fetus qi	妊娠中の腹部および喉頭の圧迫感で、喘および易刺激性を伴うことすらある。妊娠性苦悶感または胎気の上逆と同義。
胎氣上逆	子懸; 胎氣上逆	3.3.44	pregnancy suspension	feeling of pressure in the abdomen and thorax, even with dyspnea and irritability, during pregnancy, the same as gravid oppression or upward flow of fetus qi	妊娠中の腹部および喉頭の圧迫感で、喘および易刺激性を伴うことすらある。妊娠性苦悶感または胎気の上逆と同義。

検索表示（原表示の複数語を分離）	IST原表示	Code	Term	Definition/Description	定義/記述
子癇	子癇; 妊娠癇證	3.3.45	eclampsia of pregnancy	sudden onset of convulsions and loss of consciousness occurring in a pregnant or puerperal woman with headache and vertigo	妊娠期または産褥期に発生する、抽搐および神昏の突発。頭痛および回転性めまいを伴う。
妊娠癇證	子癇; 妊娠癇證	3.3.45	eclampsia of pregnancy	sudden onset of convulsions and loss of consciousness occurring in a pregnant or puerperal woman with headache and vertigo	妊娠期または産褥期に発生する、抽搐および神昏の突発。頭痛および回転性めまいを伴う。
子嗽	子嗽; 妊娠咳嗽	3.3.46	cough during pregnancy	persistent cough during pregnancy, the same as gravid cough	妊娠中の持続性咳嗽。妊娠性咳嗽と同義。
妊娠咳嗽	子嗽; 妊娠咳嗽	3.3.46	cough during pregnancy	persistent cough during pregnancy, the same as gravid cough	妊娠中の持続性咳嗽。妊娠性咳嗽と同義。
子淋	子淋; 妊娠小便淋痛	3.3.47	strangury of pregnancy	difficult and painful discharge of urine during pregnancy	妊娠中の排尿困難および排尿痛。
妊娠小便淋痛	子淋; 妊娠小便淋痛	3.3.47	strangury of pregnancy	difficult and painful discharge of urine during pregnancy	妊娠中の排尿困難および排尿痛。
過期不産		3.3.48	post-term pregnancy	pregnancy extended beyond the expected date of delivery	分娩予定日を超過した妊娠。
難産	難産; 産難	3.3.49	difficult delivery	slow and difficult delivery	遅延分娩および難産。
産難	難産; 産難	3.3.49	difficult delivery	slow and difficult delivery	遅延分娩および難産。
胞衣不下	胞衣不下; 息胞	3.3.50	retention of placenta	retarded delivery of the afterbirth	出産後の胎盤の滞留。
息胞	胞衣不下; 息胞	3.3.50	retention of placenta	retarded delivery of the afterbirth	出産後の胎盤の滞留。
子死腹中		3.3.51	dead fetus in the uterus	retention of a dead fetus in the uterus beyond the period of normal gestation	正常な妊娠期間を超えた、死胎の子宮内滞留。
惡露		3.3.52	lochia	vaginal discharge in the puerperium	産褥期の膣分泌物。
惡露不下		3.3.53	retention of the lochia	absence of postpartum vaginal discharge	産後の膣分泌物の分泌不全。
惡露不絶	惡露不絶; 惡露不止	3.3.54	persistent flow of the lochia	abnormally prolonged discharge of lochia for more than 3 weeks after childbirth, the same as lochiorrhea	産後3週間を超える、長期の悪露異常分泌。悪露過多と同義。
惡露不止	惡露不絶; 惡露不止	3.3.54	persistent flow of the lochia	abnormally prolonged discharge of lochia for more than 3 weeks after childbirth, the same as lochiorrhea	産後3週間を超える、長期の悪露異常分泌。悪露過多と同義。
缺乳		3.3.55	oligogalactia	deficiency of milk secretion during lactation, the same as hypogalactia	授乳期中の乳汁分泌不足。乳汁分泌不全と同義。
乳汁不行	乳汁不行; 乳汁不通	3.3.56	agalactia	no milk secretion following childbirth	産後の乳汁不分泌。
乳汁不通	乳汁不行; 乳汁不通	3.3.56	agalactia	no milk secretion following childbirth	産後の乳汁不分泌。
乳汁自出	乳汁自出; 乳溢	3.3.57	galactorrhea	spontaneous flow of milk irrespective of nursing	授乳に無関係な乳汁の自然流出。
乳溢	乳汁自出; 乳溢	3.3.57	galactorrhea	spontaneous flow of milk irrespective of nursing	授乳に無関係な乳汁の自然流出。
産後乳汁自出		3.3.58	postpartum galactorrhea	spontaneous flow of milk irrespective of nursing following childbirth	産後の授乳に無関係な乳汁の自然流出。
不孕		3.3.59	infertility	lack of capacity to produce offspring	受胎能の欠如。
腸覃		3.3.60	lower abdominal mass (in woman)	formation of mass in the lower abdomen with regular menstrual cycle	定期的な月経周期を伴う下腹部の腫瘤形成。
石瘕		3.3.61	stony conglomeration	a mass as hard as stone formed in the uterus	子宮内に形成される、石様に硬い腫瘤。
轉胞		3.3.62	shifted bladder	a disease marked by acute pain below the umbilicus associated with anuresis	尿閉に付随する臍下部の急性疼痛を現す疾患。
陰挺	陰挺; 陰脱	3.3.63	prolapse of the uterus	downward displacement of the uterus, even with the entire uterus outside the vaginal orifice	子宮の下方偏位。膣口外に子宮全体が出ることすらある。
陰脱	陰挺; 陰脱	3.3.63	prolapse of the uterus	downward displacement of the uterus, even with the entire uterus outside the vaginal orifice	子宮の下方偏位。膣口外に子宮全体が出ることすらある。
陰癢		3.3.64	pudendal itch	itching of the female external genitalia or vagina	女性外生殖器または膣の掻痒。
陰腫		3.3.65	pudendal swelling	painful swelling of the female external genitalia	女性外生殖器の有痛性腫脹。
陰瘡		3.3.66	pudendal sore	sore or ulcer in the female external genital region	女性外生殖器の瘡または潰瘍。
陰痛		3.3.67	pudendal pain	pain of the female pudendum, including the vestibule of the vagina and vaginal orifice	膣前庭部および膣口を含む、陰唇の疼痛。
陰吹		3.3.68	vaginal flatus	noisy expulsion of gas from the vagina	膣からの有音なガス排出。
臟躁		3.3.69	hysteria	a paroxysmal mental disease marked by depression and melancholy, irritability, lack of control over emotions, sadness with an urge to weep, and sighing for no apparent reason	鬱病を現す発作性精神疾患。明白な理由のない憂うつ、易刺激性、感情制御の欠如、啼泣衝動を伴う悲嘆、嘆息を現す。

検索表示（原表示の複数語を分離）	IST原表示	Code	Term	Definition/Description	定義/記述
兒科; 小兒科		**3.4.0**	**Pediatrics**		
胎怯	胎怯; 胎弱	3.4.1	fetal weakness	congenital weak constitution	先天性虚弱体質。
胎弱	胎怯; 胎弱	3.4.1	fetal weakness	congenital weak constitution	先天性虚弱体質。
胎熱		3.4.2	fetal heat	a group of diseases with heat manifestations occurring in the newborn due to contraction of heat toxin in the fetal stage	胎児期の熱毒感受による、新生児に発生する熱発現を伴う疾患群。
囟陷		3.4.3	depressed fontanel	sunken fontanel in an infant	乳児の泉門沈下。
囟塡		3.4.4	bulging fontanel	outward swelling of the fontanel in an infant	乳児の泉門の外側への腫脹。
變蒸		3.4.5	growth fever	a physiological phenomenon due to the growth and development of the child	小児の成長および発達による生理学的現象。
滯頤	滯頤; 小兒多涎	3.4.6	dribbling	salivation in an infant with wet cheeks	頬が濡れるほどの乳児の流涎。
小兒多涎	滯頤; 小兒多涎	3.4.6	dribbling	salivation in an infant with wet cheeks	頬が濡れるほどの乳児の流涎。
溢乳		3.4.7	milk regurgitation	vomiting of milk due to improper feeding	不適切な授乳による乳汁嘔吐。
百晬内嗽		3.4.8	neonatal cough	cough with sputum and dyspnea occurring in newborn within one hundred days after birth	生後100日以内の乳児に起こる、痰を伴う咳嗽と喘。
小兒哮喘		3.4.9	infantile asthma	an infantile disease characterized by paroxysmal labored breathing accompanied by a whistling sound	発作性努力呼吸を特徴とする小児疾患で、喘鳴が付随する。
馬脾風		3.4.10	horse-spleen wind	critical case of asthma and lung distension in children with a sudden attack	突然の発作を伴う、小児の喘息および肺脹の重症例。
食積		3.4.11	food accumulation	a diseased state characterized by the accumulation of undigested food in the stomach and intestines, causing abdominal distension and pain, vomiting, diarrhea and anorexia	胃および小腸での未消化食物の蓄積を特徴とする病的状態。腹脹および腹痛、嘔吐、泄瀉、厭食の原因となる。
疳	疳; 疳癆	3.4.12	(infantile) malnutrition	a chronic nutritional disorder of infants with emaciation and weakness	羸痩および虚弱を伴う乳児の慢性的栄養疾患。
疳癆	疳; 疳癆	3.4.12	(infantile) malnutrition	a chronic nutritional disorder of infants with emaciation and weakness	羸痩および虚弱を伴う乳児の慢性的栄養疾患。
疳氣		3.4.13	mild (infantile) malnutrition	a mild case of infantile malnutrition at the early stage	初期の疳の軽度な症例。
疳積		3.4.14	mild (infantile) malnutrition with accumulation	infantile malnutrition at the intermediate stage, accompanied by food accumulation and stagnation in the stomach and intestines	中期の疳で、胃および小腸の食物蓄積および停滞を併存する。
丁奚疳		3.4.15	T-shaped malnutrition	a severe case of infantile malnutrition with an emaciated T-shaped figure	衰弱したT字型体型を伴う、疳の重症例。
乾疳		3.4.16	dryness (infantile) malnutrition	a case of infantile malnutrition at the extreme stage with drying up of fluid and blood, marked by a dry and shriveled body worn	津血の乾を伴う、極度の病期の疳の症例。乾燥し萎縮した体の消耗を現す。
血疳		3.4.17	blood (infantile) malnutrition	a case of infantile malnutrition marked by blood deficiency	血虚を現す疳の症例。
脾疳	脾疳; 食疳	3.4.18	spleen (infantile) malnutrition	a case of infantile malnutrition due to debilitation of the spleen and stomach with accumulation of dampness-heat, the same as food malnutrition	湿熱の蓄積を伴う、脾および胃の衰弱による疳の症例。食疳と同義。
食疳	脾疳; 食疳	3.4.18	spleen (infantile) malnutrition	a case of infantile malnutrition due to debilitation of the spleen and stomach with accumulation of dampness-heat, the same as food malnutrition	湿熱の蓄積を伴う、脾および胃の衰弱による疳の症例。脾疳と同義。
心疳	心疳; 驚疳	3.4.19	heart (infantile) malnutrition	a case of infantile malnutrition due to debilitation of the spleen and stomach with depressed heat in the heart meridian, the same as fright malnutrition	心経の鬱熱を伴う、脾および胃の衰弱による疳の症例。驚疳と同義。
驚疳	心疳; 驚疳	3.4.19	heart (infantile) malnutrition	a case of infantile malnutrition due to debilitation of the spleen and stomach with depressed heat in the heart meridian, the same as fright malnutrition	心経の鬱熱を伴う、脾および胃の衰弱による疳の症例。心疳と同義。
肝疳	肝疳; 筋疳	3.4.20	liver (infantile) malnutrition	a case of infantile malnutrition due to debilitation of the spleen and stomach with depressed heat in the liver meridian, the same as sinew malnutrition	肝経の鬱熱を伴う、脾および胃の衰弱による疳の症例。筋疳と同義。
筋疳	肝疳; 筋疳	3.4.20	liver (infantile) malnutrition	a case of infantile malnutrition due to debilitation of the spleen and stomach with depressed heat in the liver meridian, the same as sinew malnutrition	肝経の鬱熱を伴う、脾および胃の衰弱による疳の症例。肝疳と同義。
肺疳	肺疳; 氣疳	3.4.21	lung (infantile) malnutrition	a case of infantile malnutrition due to debilitation of the spleen and stomach with depressed heat damaging the lung, the same as qi malnutrition	肺を傷する鬱熱を伴う、脾および胃の衰弱による疳の症例。氣疳と同義。
氣疳	肺疳; 氣疳	3.4.21	lung (infantile) malnutrition	a case of infantile malnutrition due to debilitation of the spleen and stomach with depressed heat damaging the lung, the same as qi malnutrition	肺を傷する鬱熱を伴う、脾および胃の衰弱による疳の症例。肺疳と同義。
腎疳	腎疳; 骨疳	3.4.22	kidney (infantile) malnutrition	a case of infantile malnutrition ascribed to a congenital defect with weak constitution or chronic spleen disorder that involves the kidney, resulting in dual deficiency of the spleen and kidney, the same as bone malnutrition	全身性虚弱または腎に関わる慢性脾疾患を伴う、先天異常による疳の症例であり、脾および腎の両虚をもたらす。骨疳と同義。
骨疳	腎疳; 骨疳	3.4.22	kidney (infantile) malnutrition	a case of infantile malnutrition ascribed to a congenital defect with weak constitution or chronic spleen disorder that involves the kidney, resulting in dual deficiency of the spleen and kidney, the same as bone malnutrition	全身性虚弱または腎に関わる慢性脾疾患を伴う、先天異常による疳の症例であり、脾および腎の両虚をもたらす。腎疳と同義。
眼疳		3.4.23	eye (infantile) malnutrition	attack of liver fire to the eyes in a malnourished child	疳を持つ小児での、肝火の眼への侵襲。
蛔疳		3.4.24	ascaris (infantile) malnutrition	malnutrition due to ascaris infestation	回虫寄生による疳。
哺乳疳		3.4.25	lactational malnutrition	malnutrition of an infant due to improper breast-feeding	不適切な哺乳による乳児での疳。

検索表示（原表示の複数語を分離）	IST原表示	Code	Term	Definition/Description	定義/記述
疳腫脹		3.4.26	(infantile) nutritional edema	infantile malnutrition marked by edema and abdominal distention	水腫および腹脹を現す疳。
厭食		3.4.27	anorexia	a diseased state in children characterized by loss of appetite for food	食欲喪失を特徴とする、小児の病的状態。
鵝口瘡	鵝口瘡;雪口	3.4.28	thrush	a disease marked by white patches on the buccal mucosa and tongue	頬粘膜および舌の白色斑を特徴とする疾患。
雪口	鵝口瘡;雪口	3.4.28	thrush	a disease marked by white patches on the buccal mucosa and tongue	頬粘膜および舌の白色斑を特徴とする疾患。
口瘡		3.4.29	aphtha	small painful oral ulcer	小さい有痛性の口腔潰瘍。
馬牙		3.4.30	gingival eruption	sporadic eruption of small yellowish spots on the gums in the newborn	新生児の歯茎上の、小さい黄色がかった斑点の散発的発生。
木舌		3.4.31	wooden tongue	swollen, hardened tongue, stiff as a piece of wood, seen in glossitis of the newborn	新生児の舌炎に見られる、木片のように硬く、腫脹し硬化した舌。
齘齒		3.4.32	teeth grinding	noisy grinding of teeth during sleep	睡眠中の、音の大きい歯の摩擦音。
驚風		3.4.33	infantile convulsion	infantile diseases marked by convulsions and loss of consciousness	抽搐および神昏を現す小児疾患。
天釣		3.4.34	convulsion with uplifted eyes	infantile convulsion with high fever, tossed head and upward staring eyes	壮熱、頭部後反、上方凝視を伴う小児抽搐。
内釣		3.4.35	convulsion with abdominal pain	infantile convulsion mainly manifested as visceral contraction and abdominal colic	主に臓惑および腹疝を発現する小児抽搐。
急驚風		3.4.36	acute infantile convulsion	infantile convulsion of acute onset, accompanied by high fever and loss of consciousness	急性発症する小児抽搐。壮熱、神昏を併存する。
慢驚風		3.4.37	chronic infantile convulsion	repeated infantile convulsion of gradual onset, associated with loss of consciousness or paralysis and poor prognosis	緩徐に発症する反復性小児抽搐。神昏または麻痺、予後不良と関連する。
慢脾風		3.4.38	chronic spleen wind	a critical case of chronic infantile convulsion marked by yang debilitation and yin exuberance, usually occurring after protracted vomiting and diarrhea with spleen qi deficiency and stirring of liver wind	陰盛陽衰を特徴とする慢性の小児痙攣の危篤症例。通常、脾の気虚および肝風の動を伴う長期にわたる嘔吐および泄瀉の後に発生する。
癲癇	癲癇;癲疾;癇病	3.4.39	epilepsy	a disease characterized by temporary loss of consciousness with foaming at the mouth and convulsions of the limbs, and return to normal after the episode	口腔内泡沫および四肢抽搐を伴う一時的神昏を特徴とする疾患。発作後は正常に回復する。
癲疾	癲癇;癲疾;癇病	3.4.39	epilepsy	a disease characterized by temporary loss of consciousness with foaming at the mouth and convulsions of the limbs, and return to normal after the episode	口腔内泡沫および四肢抽搐を伴う一時的神昏を特徴とする疾患。発作後は正常に回復する。
癇病	癲癇;癲疾;癇病	3.4.39	epilepsy	a disease characterized by temporary loss of consciousness with foaming at the mouth and convulsions of the limbs, and return to normal after the episode	口腔内泡沫および四肢抽搐を伴う一時的神昏を特徴とする疾患。発作後は正常に回復する。
驚癇		3.4.40	fright epilepsy	epileptic seizure induced by fright	驚により誘発される癲癇発作。
風癇		3.4.41	wind epilepsy	epileptic seizure induced by liver wind stirring internally	内動する肝風により誘発される癲癇発作。
痰癇		3.4.42	phlegm epilepsy	epileptic seizure induced by depressed qi and bound phlegm	鬱気および結痰により誘発される癲癇発作。
龜背		3.4.43	tortoise back	back shaped like a tortoise-shell due to kyphosis	脊柱後弯症による亀の甲羅様の形状の背部。
龜胸	龜胸;鷄胸	3.4.44	pigeon chest	deformity of the chest in which the sternum is prominent, like the chicken breast, also known as pectus carinatum	胸骨が顕著な胸部の、鶏の胸部様の変形。鳩胸としても知られる。
鷄胸	龜胸;鷄胸	3.4.44	pigeon chest	deformity of the chest in which the sternum is prominent, like the chicken breast, also known as pectus carinatum	胸骨が顕著な胸部の、鶏の胸部様の変形。鳩胸としても知られる。
解顱		3.4.45	ununited skull	abnormal accumulation of excess fluid within the skull marked by enlargement of the head and retarded closure of the skull sutures, the same as hydrocephalus	頭蓋骨内の過剰な津の異常蓄積。頭部肥大、頭蓋骨縫合の遅れを現す。水頭症と同義。
五遲		3.4.46	five retardations	retarded development in infants covering standing, walking, hair-growth, tooth eruption and speaking	起立、歩行、毛髪成長、歯牙萌出、発声にわたる小児の発達遅延。
五軟		3.4.47	five limpnesses/ flaccidity	flaccidity of the neck, nape, extremities, muscles and mastication as striking features of delayed growth and mental retardation in infants	頸部、項部、四肢、筋肉、咀嚼の軟。小児の成長遅延、精神遅滞を著明な特徴とする。
五硬		3.4.48	five stiffnesses	stiffness of the hand, foot, waist, flesh and neck in the newborn due to a congenital defect	先天性欠損による新生児の手部、足部、腰部、肉、頸部の硬。
疰夏		3.4.49	summer non-acclimatization	children's disease usually occurring in summer, with symptoms of lassitude, dyspepsia, and abnormal bowel movements	通常夏に発生する小児の疾患で、気力低下、胸焼け、腸の異常運動の症状を伴う。
痘瘡	痘瘡;天花	3.4.50	smallpox	an acute highly contagious febrile disease characterized by skin eruptions with pustules, sloughing, and scar formation, the same as variola	急性で伝染性の高い熱性疾患。膿疱、口腔粘膜脱落、瘢痕形成を伴う皮疹を特徴とする。痘瘡・天然痘と同義。
天花	痘瘡;天花	3.4.50	smallpox	an acute highly contagious febrile disease characterized by skin eruptions with pustules, sloughing, and scar formation, the same as variola	急性で伝染性の高い熱性疾患。膿疱、口腔粘膜脱落、瘢痕形成を伴う皮疹を特徴とする。痘瘡・天然痘と同義。
痲疹		3.4.51	measles	an acute epidemic eruptive disease marked by fever and generalized maculopapular rash preceded by cough, nasal discharge, profuse tearing, and white speckles inside the mouth	発熱および全身性斑状丘疹状皮疹を現す急性の発疹性疫病。咳嗽、鼻水、大量の涙液分泌、口腔内の白色斑点が先行する。
痧		3.4.52	exanthem	a general term for skin eruption or rash, but usually referring to measles	皮疹または発疹の一般用語。しかし通常麻疹を表す。
風疹	風疹;風痧(病)	3.4.53	rubella	a contagious eruptive disease caused by seasonal wind-heat or wind-heat toxin, and marked by mild fever, followed by enlargement of postauricular and suboccipital lymph nodes, and the appearance of pink maculo-papular rash that begins on the head and spreads to become generalized	時風熱または風熱毒により起こる伝染性発疹性疾患。微熱を特徴とし、耳後部リンパ節および後頭下リンパ節の肥大、頭部に始まり全身に広がるピンク色の斑状丘疹状皮疹が続く。
風痧	風疹;風痧(病)	3.4.53	rubella	a contagious eruptive disease caused by seasonal wind-heat or wind-heat toxin, and marked by mild fever, followed by enlargement of postauricular and suboccipital lymph nodes, and the appearance of pink maculo-papular rash that begins on the head and spreads to become generalized	時風熱または風熱毒により起こる伝染性発疹性疾患。微熱を特徴とし、耳後部リンパ節および後頭下リンパ節の肥大、頭部に始まり全身に広がるピンク色の斑状丘疹状皮疹が続く。

検索表示（原表示の複数語を分離）	IST原表示	Code	Term	Definition/Description	定義/記述
風痧病	風疹; 風痧(病)	3.4.53	rubella	a contagious eruptive disease caused by seasonal wind-heat or wind-heat toxin, and marked by mild fever, followed by enlargement of postauricular and suboccipital lymph nodes, and the appearance of pink maculo-papular rash that begins on the head and spreads to become generalized	時風熱または風熱毒により起こる伝染性発疹性疾患。微熱を特徴とし、耳後部リンパ節および後頭下リンパ節の肥大、頭部に始まり全身に広がるピンク色の斑状丘疹状皮疹が続く。
喉痧	喉痧; 爛喉丹痧; 丹痧; 疫痧; 爛喉風	3.4.54	scarlatina	an acute seasonal infectious disease marked by swelling and erosion of the throat, erythematous rash and raspberry tongue	喉頭の腫脹およびびらん、紅斑性皮疹、いちご舌を特徴とする急性の季節性の感染症。
爛喉丹痧	喉痧; 爛喉丹痧; 丹痧; 疫痧; 爛喉風	3.4.54	scarlatina	an acute seasonal infectious disease marked by swelling and erosion of the throat, erythematous rash and raspberry tongue	喉頭の腫脹およびびらん、紅斑性皮疹、いちご舌を特徴とする急性の季節性の感染症。
丹痧	喉痧; 爛喉丹痧; 丹痧; 疫痧; 爛喉風	3.4.54	scarlatina	an acute seasonal infectious disease marked by swelling and erosion of the throat, erythematous rash and raspberry tongue	喉頭の腫脹およびびらん、紅斑性皮疹、いちご舌を特徴とする急性の季節性の感染症。
爛喉風	喉痧; 爛喉丹痧; 丹痧; 疫痧; 爛喉風	3.4.54	scarlatina	an acute seasonal infectious disease marked by swelling and erosion of the throat, erythematous rash and raspberry tongue	喉頭の腫脹およびびらん、紅斑性皮疹、いちご舌を特徴とする急性の季節性の感染症。
水痘	水痘; 水疱; 水花; 水瘡	3.4.55	chickenpox	an acute contagious disease caused by seasonal toxin and manifested by fever and bursts of macular and papular eruptions followed by incrustation but no scar formation, the same as varicella	時毒により起こる急性伝染性疾患。熱と斑状疹および丘疹の破裂を発現し、瘡蓋が後続するが、瘡形成はない。水痘と同義。
水疱	水痘; 水疱; 水花; 水瘡	3.4.55	chickenpox	an acute contagious disease caused by seasonal toxin and manifested by fever and bursts of macular and papular eruptions followed by incrustation but no scar formation, the same as varicella	時毒により起こる急性伝染性疾患。熱と斑状疹および丘疹の破裂を発現し、瘡蓋が後続するが、瘡形成はない。水痘と同義。
水花	水痘; 水疱; 水花; 水瘡	3.4.55	chickenpox	an acute contagious disease caused by seasonal toxin and manifested by fever and bursts of macular and papular eruptions followed by incrustation but no scar formation, the same as varicella	時毒により起こる急性伝染性疾患。熱と斑状疹および丘疹の破裂を発現し、瘡蓋が後続するが、瘡形成はない。水痘と同義。
水瘡	水痘; 水疱; 水花; 水瘡	3.4.55	chickenpox	an acute contagious disease caused by seasonal toxin and manifested by fever and bursts of macular and papular eruptions followed by incrustation but no scar formation, the same as varicella	時毒により起こる急性伝染性疾患。熱と斑状疹および丘疹の破裂を発現し、瘡蓋が後続するが、瘡形成はない。水痘と同義。
痄腮	痄腮; 腮腫	3.4.56	mumps	an epidemic disease caused by wind-heat toxin and characterized by painful swelling of one or both parotid glands	風熱毒により起こる流行性疾患で、耳下腺の片方または両方の有痛性腫脹を特徴とする。
腮腫	痄腮; 腮腫	3.4.56	mumps	an epidemic disease caused by wind-heat toxin and characterized by painful swelling of one or both parotid glands	風熱毒により起こる流行性疾患で、耳下腺の片方または両方の有痛性腫脹を特徴とする。
白喉	白喉; 白纏喉	3.4.57	diphtheria	an acute infectious disease characterized by fever, sore throat and the formation of white false membrane attached firmly to the pharynx, larynx and nose	熱、咽喉痛および咽頭・喉頭・鼻に固着した白色の偽膜形成を特徴とする急性感染症。
白纏喉	白喉; 白纏喉	3.4.57	diphtheria	an acute infectious disease characterized by fever, sore throat and the formation of white false membrane attached firmly to the pharynx, larynx and nose	熱、咽喉痛および咽頭・喉頭・鼻に固着した白色の偽膜形成を特徴とする急性感染症。
頓咳		3.4.58	whooping cough	an acute contagious infection of the respiratory system with characteristic paroxysmal cough, consisting of a deep inspiration, followed by a series of quick, short coughs which end with a long shrill and whooping inspiration, referring to pertussis	呼吸器系の急性伝染性感染症。深吸気があり、一連の短く素早い咳が後続し、長く甲高い、ゼーゼーいう吸気で終わる、特徴的な発作性咳嗽を伴う。百日咳を表す。
夜啼		3.4.59	night crying	morbid night crying in babies, but quiet at daytime	乳児の病的な夜泣きであるが、昼間は静かである。
客忤夜啼		3.4.60	night crying due to fright	night crying in babies caused by fright	驚による乳児の夜泣き。
客忤		3.4.61	fright seizure	a seizure of fright that causes vomiting, abdominal pain, and even convulsions	嘔吐、腹痛、場合によっては抽搐を起こすことすらある、驚の発作。
胎黃	胎黃; 胎疸	3.4.62	neonatal jaundice	yellow discoloration of the skin and sclera seen in the newborn	新生児に見られる肌膚および強膜の黄変。
胎疸	胎黃; 胎疸	3.4.62	neonatal jaundice	yellow discoloration of the skin and sclera seen in the newborn	新生児に見られる肌膚および強膜の黄変。
胎赤		3.4.63	fetal redness	abnormal redness of the skin in a newborn, often due to affection by toxic heat at the fetal stage, the same as erythroderma neonatorum	新生児の肌膚の異常発赤。しばしば胎児期の毒熱罹患による。新生児紅皮症と同義。
臍風		3.4.64	umbilical wind	a disease of the newborn characterized by cyanotic lips, lockjaw, generalized tonic spasm and tetanic convulsions, referring to neonatal tetanus	口唇チアノーゼ、牙関緊急、全身性筋緊張性攣縮、強縮性痙攣を特徴とする新生児疾患。新生児痙攣を表す。
臍濕		3.4.65	umbilical dampness	a condition of wetness of and possible exudation from the umbilicus after the umbilical cord has been shed, referring to omphalorrhea	臍帯が取れた後の臍部の湿りおよび臍部からの滲出液の状態。臍リンパ液漏を表す。
臍瘡		3.4.66	umbilical sores	redness and swelling of the umbilicus or ulceration spreading around the umbilicus with purulent exudation	化膿性滲出液を伴う、臍部の発赤および腫脹または臍部周辺に広がる潰瘍。

検索表示（原表示の複数語を分離）	IST原表示	Code	Term	Definition/Description	定義/記述
臍疝	臍疝; 臍突	3.4.67	umbilical hernia	a type of abdominal hernia in which part of the intestine protrudes at the umbilicus and is covered with skin, sometimes red and swollen	小腸の一部が臍部で突出した腹部ヘルニアの一種で、肌膚に覆われており、発赤、腫脹することがある。
臍突	臍疝; 臍突	3.4.67	umbilical hernia	a type of abdominal hernia in which part of the intestine protrudes at the umbilicus and is covered with skin, sometimes red and swollen	小腸の一部が臍部で突出した腹部ヘルニアの一種で、肌膚に覆われており、発赤、腫脹することがある。
眼科		**3.5.0**	**Ophthalmology**		
羞明		3.5.1	photophobia	abnormal fear of light or painful sensitiveness to light with lacrimination	流涙を伴う、光に対する異常な恐怖または痛感。
目暗		3.5.2	dim vision	hazy and indistinct vision	かすんだ、不明瞭な視界。
翳		3.5.3	corneal opacity	cloudy opacity of the cornea	角膜の曇った混濁。
障		3.5.4	vision obstruction	a general term for any eye disease with visual disturbance	視覚障害を伴う眼疾患の一般用語。
目盲		3.5.5	blindness	lack or loss of ability to see	視力の不足または喪失。
鍼眼		3.5.6	sty	a small furuncle occurring on the eyelid, in the shape and size of wheat grain	小麦粒様の形、大きさをした、眼瞼に発生する小型の癰。
胞生痰核		3.5.7	phlegm node of the eyelid	a small lump formed on the eyelid, without redness or pain, mostly referring to chalazion	眼瞼上に形成される小さな塊で、発赤や疼痛は伴わない。主に霰粒腫を表す。
椒瘡		3.5.8	trachoma	an eye disease marked by innumerable granulations, red and hard, shaped like Chinese prickly ash, accumulating on the conjunctival surfaces	多数の肉芽形成を現す眼疾患で、肉芽は赤く硬質で花椒様の形状であり、結膜表面に蓄積する。
粟瘡		3.5.9	conjunctival folliculitis	an eye disease marked by formation on the conjunctival surface of numerous follicles in the shape and size of millet	結膜表面上における粟様の形状・大きさの無数の胞の形成を特徴とする眼疾患。
瞼弦赤爛		3.5.10	marginal blepharitis	inflammation and ulceration of the margin of the eyelid	眼瞼辺縁の炎症および潰瘍。
風赤瘡痍		3.5.11	wind red sore	disease of the eyelid characterized by redness of the palpebral skin with vesicles which turn into erosion after rupture	眼瞼肌膚の発赤を特徴とする眼瞼疾患。破裂後びらんになる小胞を伴う。
胞腫如桃		3.5.12	inflammatory swelling of the eyelid	severe swelling of the eyelid with redness resembling a ripe peach	熟した桃に類似した発赤を伴う眼瞼の重度の腫脹。
胞虚如球		3.5.13	non-inflammatory edema of the eyelid	severe swelling of the eyelid resembling a soft ball, but with no change in the local skin color, and not painful on touch	軟らかいボールに類似した眼瞼の重度の腫脹であるが、局所的な肌膚の変色はなく、触れても痛みはない。
上胞下垂		3.5.14	blepharoptosis	drooping of the upper eyelid, impairing the vision if it covers the pupil	上眼瞼の下垂。瞳孔を覆うと視界を阻害する。
瞼廢		3.5.15	drooping eyelid	serious blepharoptosis	重篤な眼瞼下垂。
胞輪振跳		3.5.16	twitching eyelid	involuntary twitching of the eyelid	眼瞼の不随意な攣縮。
倒睫拳毛		3.5.17	trichiasis	inversion of the margin of the eyelid with ingrowing eyelashes, causing pain of the eye, lacrimination and photophobia	逆さ睫毛を伴う、眼瞼辺縁の逆転。眼痛、落涙、羞明を起こす。
熱涙		3.5.18	heat tearing	dacryorrhea of heat type, occurring in inflammatory eye diseases	炎症性眼疾患で発生する熱型の流涙過多。
冷涙		3.5.19	cold tearing	dacryorrhea of cold type, with no redness, pain or opacity of the eye	寒型の流涙過多。発赤、疼痛、眼の混濁を伴わない。
漏睛	漏睛; 漏睛膿出; 眥漏	3.5.20	dacryopyorrhea	chronic inflammation of the lacrimal sac with frequent outflow of fluid or pus from the inner canthus	涙嚢の慢性炎。内眼角からの津または膿の頻繁な流出を伴う。
漏睛膿出	漏睛; 漏睛膿出; 眥漏	3.5.20	dacryopyorrhea	chronic inflammation of the lacrimal sac with frequent outflow of fluid or pus from the inner canthus	涙嚢の慢性炎。内眼角からの津または膿の頻繁な流出を伴う。
眥漏	漏睛; 漏睛膿出; 眥漏	3.5.20	dacryopyorrhea	chronic inflammation of the lacrimal sac with frequent outflow of fluid or pus from the inner canthus	涙嚢の慢性炎。内眼角からの津または膿の頻繁な流出を伴う。
胬肉攀睛		3.5.21	pterygium	triangular fleshy mass occurring at the canthus and covering a part of the cornea	眼角で発生し角膜の一部を覆う、三角形の肉塊。
暴風客熱		3.5.22	sudden attack of wind-heat on the eye	acute inflammation of the white of the eye with a sudden onset, due to external contraction of wind-heat	風熱の外感による、突然発症する白睛の急性炎症。
天行赤眼		3.5.23	epidemic conjunctivitis	a highly contagious eye disease characterized by sudden onset of inflammation of the white of the eye, usually bilateral, and quickly spreading among the community	白睛の炎症の突発を特徴とする、伝染性の高い眼疾患。通常両側性で、共同体内に急速に蔓延する。
暴赤生翳		3.5.24	acute conjunctivitis with nebula	an acute epidemic inflammatory disease of the conjunctiva and cornea with redness, swelling, pain, photophobia and lacrimination, followed by clusters of nebula formation	結膜および角膜の急性の流行性炎症性疾患。発赤、腫脹、疼痛、羞明、落涙を伴い、角膜白濁形成クラスターが後続する。
金疳	金疳; 金瘍	3.5.25	phlyctenular conjunctivitis	a variety of conjunctivitis marked by the presence of small vesicles, each surrounded by a reddened zone, also known as follicular conjunctivitis	小胞存在を現す多様な結膜炎。赤色の領域でおのおの囲まれており、濾胞性結膜炎としても知られる。
金瘍	金疳; 金瘍	3.5.25	phlyctenular conjunctivitis	a variety of conjunctivitis marked by the presence of small vesicles, each surrounded by a reddened zone, also known as follicular conjunctivitis	小胞存在を現す多様な結膜炎。赤色の領域でおのおの囲まれており、濾胞性結膜炎としても知られる。
火疳	火疳; 火瘍	3.5.26	acute scleritis	an eye disease caused by excessive fire which invades the inner surface of the white of the eye, resulting in bulging of localized dark violet patches, also referring to episcleritis	白睛の内表に侵襲する過剰な火を原因とする眼疾患。局在性の暗紫色の斑点の膨隆をもたらす。上強膜炎を表すこともある。
火瘍	火疳; 火瘍	3.5.26	acute scleritis	an eye disease caused by excessive fire which invades the inner surface of the white of the eye, resulting in bulging of localized dark violet patches, also referring to episcleritis	白睛の内表に侵襲する過剰な火を原因とする眼疾患。局在性の暗紫色の斑点の膨隆をもたらす。上強膜炎を表すこともある。
白膜侵睛		3.5.27	invasion of white membrane into the cornea	a special type of phlyctenular conjunctivitis marked by presence of small vesicles close to the cornea with resultant white membranous substance entering the cornea	角膜近接の小胞の存在を特徴とする、特殊型のフリクテン性角結膜炎。角膜に侵入する白色膜物質を伴う。

検索表示（原表示の複数語を分離）	IST原表示	Code	Term	Definition/Description	定義/記述
白睛青藍		3.5.28	bluish discoloration of sclera	bluish discoloration of the bulbar conjunctiva surrounding the cornea after recurrent inflammation of the sclera with violet bulging	強膜の再発性炎症後の角膜周辺の眼球結膜の青みがかった変色。青紫色の膨隆を伴う。
白睛溢血		3.5.29	subconjunctival hemorrhage	extravasations beneath the superficial membrane of the white of the eye	白睛の表面膜下の血管外漏出。
聚星障		3.5.30	superficial punctate keratitis	an eye disease characterized by the appearance of multiple fine drops of opacity on the cornea	角膜上の多発性の細かい水滴状の混濁の出現を特徴とする眼疾患。
花翳白陷		3.5.31	corneal ulcer	an eye disease characterized by ulceration of a corneal nebula looking like a flower	花状に見える、角膜片雲の潰瘍化を特徴とする眼疾患。
凝脂翳		3.5.32	purulent keratitis	severe keratitis with purulent disintegration of the cornea	角膜の化膿性崩壊を伴う重度の角膜炎。
赤膜下垂		3.5.33	drooping pannus	a membranous vascular tissue extending downward into the cornea, most frequently occurring in cases of trachoma	角膜内に下垂する膜血管組織。トラコーマ症例において最も頻繁に発生する。
血翳包睛		3.5.34	keratic pannus	superficial vascularization covering the entire cornea	角膜全体を覆う表面血管新生。
瞳神乾缺	瞳神乾缺; 瞳人乾缺	3.5.35	pupillary metamorphosis	loss of the normal round shape of the pupil, usually seen in chronic iridocyclitis	円形の正常な瞳孔の喪失。通常、慢性虹彩毛様体炎で見られる。
瞳人乾缺	瞳神乾缺; 瞳人乾缺	3.5.35	pupillary metamorphosis	loss of the normal round shape of the pupil, usually seen in chronic iridocyclitis	円形の正常な瞳孔の喪失。通常、慢性虹彩毛様体炎で見られる。
綠風內障		3.5.36	greenish glaucoma	an acute eye disease characterized by hardening of the eyeball, drastic impairment of vision, dilation of the pupil with greenish discoloration, and severe headache and eye pain	眼球硬化、強烈な視覚障害、緑がかった変色を伴う瞳孔拡大、重度の頭痛および眼痛を特徴とする急性眼疾患。
圓翳內障		3.5.37	cataract	a chronic eye disease marked by opacity in the lens, impairing vision or causing blindness	水晶体混濁、視覚障害または盲目発生を特徴とする慢性眼疾患。
驚震內障		3.5.38	traumatic cataract	cataract resulting from injury to the eye	眼の損傷に起因する白内障。
胎患內障		3.5.39	congenital cataract	opacity of the lens capsule of the eye at birth	出生時の水晶体嚢の混濁。
雲霧移睛		3.5.40	hyalosis	presence of cloudy or star-shaped opacities in the vitreous humor	神膏内の雲状または星型の混濁の存在。
暴盲		3.5.41	sudden blindness	a severe eye disease characterized by sudden loss of vision while no abnormal appearance of the eye can be found	外見上の眼の異常は見られないが突然の視力喪失を特徴とする重度の眼疾患。
青盲		3.5.42	bluish blindness	a group of eye diseases characterized by increasing impairment of vision to total blindness with no abnormal appearance of the external eye, referring to optic nerve atrophy	眼表面に外見上の異常は伴わないが、全盲にいたる増加性の視覚障害を特徴とする眼疾患の一群。視神経萎縮を表す。
雀目	雀目; 雀盲	3.5.43	night blindness	failure of vision at night or in a dim light	夜間または薄暗い照明下での視力不全。
雀盲	雀目; 雀盲	3.5.43	night blindness	failure of vision at night or in a dim light	夜間または薄暗い照明下での視力不全。
高風內障		3.5.44	retinopathy pigmentosa	a progressive degenerative disease characterized by night blindness, constriction of the visual field, and eventual blindness	雀目、視野狭窄、最終的には目盲を特徴とする進行性変性疾患。
異物入目		3.5.45	foreign body in the eye	small foreign body attached to or embedded in the surface of the eyeball	眼球表面に付着または埋め込まれた小さな異物。
撞擊傷目		3.5.46	collision eye injury	eye injury due to a knock with no ruptured wound	破裂による創傷は伴わない、打撃による眼損傷。
眞睛破損		3.5.47	ruptured wound of the eyeball	penetrating injury to the eyeball or other eye injury with a ruptured wound	破裂による創傷を伴う、眼球への鋭的損傷または他の眼の損傷。
疳眼		3.5.48	(nutritional) keratomalacia	an eye disease resulting from infantile malnutrition, characterized by dryness of the eyeball, turbidness, softening and ulceration of the cornea	乳児期の栄養失調に起因する眼疾患。眼球乾燥、角膜の混濁、軟化、潰瘍化を特徴とする。
風牽偏視		3.5.49	paralytic strabismus	sudden onset of squint with impaired movement of the eye and double vision attributed to an attack of wind	風邪の襲来による眼球運動障害、複視を伴う斜視の突発。
鶻眼凝睛		3.5.50	fixed protruding eye	a sign marked by fixation and protrusion of the eyeball like a falcon’s eye	鷹の目様の眼球の固定および突出を特徴とする徴候。
突起睛高		3.5.51	sudden protrusion of the eyeball	an acute eye disease characterized by painful protrusion and distension of the eyeball, often referring to purulent ophthalmia	有痛性の眼球突出、脹を特徴とする急性眼疾患。しばしば化膿性眼炎を表す。
睛脹		3.5.52	distention of eyeball	a synonym for sudden protrusion of the eyeball	突起睛高の同義語。
眵		3.5.53	eye discharge	secretion of the eye, thin or mucilaginous or even pus-like	眼からの分泌物。希薄または粘液性、または膿様の場合すらある。
口眼喎斜		3.5.54	deviated eye and mouth	deviation of one eye and the mouth to one side with the eye unable to close and salivation from the homolateral corner of the mouth	片方の眼球および口の一方への偏位。閉眼不能であり、同側の口角からは流涎を伴う。
耳鼻喉口齒科		**3.6.0**	**Otorhinolaryngostomatology**		
耳疔		3.6.1	ear boil	boil of the external auditory meatus	外耳道のおでき。
耳瘡		3.6.2	ear sore	diffuse inflammation of the external auditory meatus	外耳道のびまん性炎症。
旋耳瘡	旋耳瘡; 月蝕瘡	3.6.3	eczema of external ear	skin lesion characterized by redness, itching, exudation, oozing, vesiculation and crusting surrounding the ear	耳周辺の発赤、掻痒、滲出、滲出性出血、小水疱形成、痂皮形成を特徴とする肌膚病変。
月蝕瘡	旋耳瘡; 月蝕瘡	3.6.3	eczema of external ear	skin lesion characterized by redness, itching, exudation, oozing, vesiculation and crusting surrounding the ear	耳周辺の発赤、掻痒、滲出、滲出性出血、小水疱形成、痂皮形成を特徴とする肌膚病変。
耵耳		3.6.4	impacted cerumen	accumulated cerumen forming a solid mass that clogs the external auditory meatus	外耳道を塞ぐ固形塊を形成する耳垢蓄積。
耳脹		3.6.5	ear distension	a disease of the ear characterized by feeling of distension with pain in the ear and impaired hearing, often referring to acute non-suppurative otitis media	耳内の疼痛および難聴を伴う脹感を特徴とする耳の疾患。しばしば急性非化膿性中耳炎を表す。
膿耳		3.6.6	purulent ear	a disease of the ear characterized by perforation of the tympanic membrane and discharge of pus, referring to otopyorrhea	鼓膜の穿孔および膿の排出を特徴とする耳の疾患。耳漏を表す。

検索表示（原表示の複数語を分離）	IST原表示	Code	Term	Definition/Description	定義/記述
耳根毒		3.6.7	postauricular infection	a disease marked by pain and tenderness at the mastoid region, local swelling and even rupture with discharge of pus	乳様突起領域の疼痛および圧痛、局所の腫脹を現す疾患。膿の排出を伴う破裂を現すことすらある。
耳根癰		3.6.8	postauricular abscess	postauricular infection with formation of abscess	癰形成を伴う耳後部の感染。
耳痔		3.6.9	ear pile	nodular papilloma of the external auditory meatus	外耳道の結節性乳頭腫。
耳菌	耳菌;<br>耳蕈	3.6.10	ear polyp	polyp of the external auditory meatus	外耳道のポリープ。
耳蕈	耳菌;<br>耳蕈	3.6.10	ear polyp	polyp of the external auditory meatus	外耳道のポリープ。
耳挺		3.6.11	ear protuberance	a long-stemmed papilloma of the external auditory meatus	外耳道の長茎乳頭腫。
耳瘻		3.6.12	ear fistula	fistula anterior or posterior to the auricle of the ear	耳介の前部および後部の瘻孔。
重聽		3.6.13	hardness of hearing	decreased sense of hearing or distorted hearing	聴覚低下または聴力障害。
鼻疔		3.6.14	nasal boil	boil occurring at the nasal vestibule, or at the tip or wing of the nose	鼻前庭部、鼻先端部または鼻翼に発生するおでき。
鼻燥	鼻燥;<br>鼻乾	3.6.15	dry nose	dryness in the nose	鼻内の燥。
鼻乾	鼻燥;<br>鼻乾	3.6.15	dry nose	dryness in the nose	鼻内の燥。
鼻瘡	鼻瘡;<br>鼻疳	3.6.16	nasal sore	a disease marked by recurrent inflammation of the nasal vestibule with ulceration, crusting, itching and pain, the same as nasal vestibulitis	潰瘍、痂皮、掻痒、疼痛を伴う鼻前庭部の再発性炎症を現す疾患。鼻前庭炎と同義。
鼻疳	鼻瘡;<br>鼻疳	3.6.16	nasal sore	a disease marked by recurrent inflammation of the nasal vestibule with ulceration, crusting, itching and pain, the same as nasal vestibulitis	潰瘍、痂皮、掻痒、疼痛を伴う鼻前庭部の再発性炎症を現す疾患。鼻前庭炎と同義。
鼻槁		3.6.17	atrophic rhinitis	a disease of the nose characterized by dry mucous membrane with atrophy, enlarged nasal passages and foul smell	萎縮、鼻道肥大、悪臭を伴う乾性粘膜を特徴とする鼻の疾患。
鼻鼽	鼻鼽;<br>鼽嚏	3.6.18	allergic rhinitis	a disease characterized by sudden and recurrent attacks of nasal itching, sneezing, thin discharge and stuffy nose	鼻の掻痒、噴嚏、希薄な鼻水、鼻閉の突発性および再発性発作を特徴とする疾患。
鼽嚏	鼻鼽;<br>鼽嚏	3.6.18	allergic rhinitis	a disease characterized by sudden and recurrent attacks of nasal itching, sneezing, thin discharge and stuffy nose	鼻の掻痒、噴嚏、希薄な鼻水、鼻閉の突発性および再発性発作を特徴とする疾患。
鼻痔	鼻痔;<br>鼻息肉;<br>鼻菌	3.6.19	nasal polyp	a pendulated growth in the nasal cavity	鼻腔内の下垂状増殖。
鼻息肉	鼻痔;<br>鼻息肉;<br>鼻菌	3.6.19	nasal polyp	a pendulated growth in the nasal cavity	鼻腔内の下垂状増殖。
鼻菌	鼻痔;<br>鼻息肉;<br>鼻菌	3.6.19	nasal polyp	a pendulated growth in the nasal cavity	鼻腔内の下垂状増殖。
鼻淵	鼻淵;<br>腦漏	3.6.20	sinusitis	a nasal disease characterized by persistent excessive flow of turbid nasal discharge	濁った鼻汁の持続的な過剰流出を特徴とする鼻の疾患。
腦漏	鼻淵;<br>腦漏	3.6.20	sinusitis	a nasal disease characterized by persistent excessive flow of turbid nasal discharge	濁った鼻汁の持続的な過剰流出を特徴とする鼻の疾患。
乳蛾	乳蛾;<br>喉蛾	3.6.21	tonsillitis	inflammation of the palatine tonsils, sometimes covered with a yellowish white secretion like milk	口蓋扁桃の炎症。乳汁様の黄白色分泌物に覆われることがある。
喉蛾	乳蛾;<br>喉蛾	3.6.21	tonsillitis	inflammation of the palatine tonsils, sometimes covered with a yellowish white secretion like milk	口蓋扁桃の炎症。乳汁様の黄白色分泌物に覆われることがある。
石蛾		3.6.22	chronic tonsillitis	hard hypertrophied palatine tonsils in children with no inflammation	炎症を伴わない、小児における口蓋扁桃の硬い肥大。
喉痹		3.6.23	throat impediment	redness, swelling and pain or itching of the throat, with impediment to swallowing	咽喉の発赤、腫脹、疼痛または掻痒。嚥下
喉癰		3.6.24	throat abscess	abscess of the throat, including retropharyngeal abscess and peritonsillar abscess	咽喉の癰。咽後の癰および扁桃の癰を含む。
猛疽		3.6.25	ominous abscess of the throat	a severe case of laryngopharyngeal abscess that causes obstruction of breathing and is often fatal if not properly treated in time	呼吸閉塞を引き起こす咽喉頭の癰の重度の症例。適切な処置が間に合わない場合、しばしば致命的になる。
喉癬		3.6.26	lichenoid erosion of the throat	the ulceration of the laryngopharyngeal mucosa resembling tinea, often referring to laryngeal tuberculosis	白癬に類似した、咽喉頭粘膜の潰瘍。咽頭結核を表すことが多い。
喉菌	喉菌;<br>喉巖	3.6.27	throat cancer	malignant tumor of the throat in the shape of mushroom	茸の形状をした咽喉悪性腫瘍。
喉巖	喉菌;<br>喉巖	3.6.27	throat cancer	malignant tumor of the throat in the shape of mushroom	茸の形状をした咽喉悪性腫瘍。
骨鯁		3.6.28	bone stuck in the throat	a condition in which fish or splintered animal bones become lodged in the throat	魚骨または割れた動物骨が咽喉に刺さった状態。
喉瘤		3.6.29	tumor of the throat	new growth of the throat	咽喉の新生物。
喉風		3.6.30	throat wind	a general term for acute conditions of swelling and pain in the throat	急性状態の咽喉の腫脹および疼痛の一般用語。
緊喉風		3.6.31	fulminant throat wind	an acute throat infection manifested as swelling and pain in the throat, difficulty in breathing and swallowing	咽喉の腫脹および疼痛、呼吸時および嚥下時の困難を発現する咽喉の急性感染症。
纏喉風		3.6.32	entwining throat wind	a severe throat infection with redness entwining the fauces in the interior and swelling surrounding the neck at the exterior	裏では口峡に絡みつく発赤、表では頸周辺の腫脹を伴う咽喉の重度の感染症。
鎖喉風		3.6.33	obstructive throat wind	acute infection of throat with local pain and swelling, dyspnea, difficulty in speaking and swallowing, and lockjaw	局所的疼痛および腫脹、喘、発声時および嚥下時の困難、牙関緊急を伴う咽喉の急性感染症。
重舌		3.6.34	double tongue	protruding sublingual swelling and redness, making a shape of doubled tongue	突出した舌下の腫脹および発赤。二重の舌のような形になる。
絆舌		3.6.35	ankyloglossia	restricted movement of the tongue, often resulting from short lingual frenulum	舌の運動の制限。しばしば舌小帯短小に起因する。
舌癰		3.6.36	tongue abscess	suppurative inflammation of the tongue	舌の化膿性炎症。

検索表示（原表示の複数語を分離）	IST原表示	Code	Term	Definition/Description	定義/記述
舌疔		3.6.37	tongue boil	a pustule on the tongue characterized by localized painful and hard swelling, associated with chills and fever	局在性で有痛性の硬質な腫脹を特徴とする、舌の膿疱。悪寒および発熱と関連する。
舌瘡		3.6.38	tongue sore	a sore occurring on the tongue with local cracking, swelling and discharge of blood, accompanied by foul breath and constipation	局所的なひび割れ、腫脹、出血を伴う、舌に発生する瘡。口臭、便秘が付随する。
舌菌		3.6.39	tongue cancer	cancer of the tongue like a mushroom with a big head and thin stalk	大きな頭部と薄い茎を持つ茸状の舌癌。
痰包	痰包; 舌下痰包	3.6.40	phlegm cyst	a swelling beneath the tongue shaped like a pouch, smooth and soft to touch, containing thick yellowish fluid	嚢様の形状をした舌下の腫脹。触れると滑らかで軟らかく、濃厚な黄色がかった津を内包する。
舌下痰包	痰包; 舌下痰包	3.6.40	phlegm cyst	a swelling beneath the tongue shaped like a pouch, smooth and soft to touch, containing thick yellowish fluid	嚢様の形状をした舌下の腫脹。触れると滑らかで軟らかく、濃厚な黄色がかった津を内包する。
牙痛		3.6.41	toothache	pain in a tooth or teeth	一本または複数の歯痛。
齲齒		3.6.42	dental caries	a dental disease marked by decay of the tooth with localized destruction and cavity formation	局在性の破壊および窩形成を伴う齲歯を現す歯科疾患。
牙宣	牙宣; 食床; 齒挺; 齒齦宣露	3.6.43	gum atrophy	exposure of the root surfaces of teeth due to the drawing back of gingivae from the necks of teeth	歯頸部からの歯肉の後退による歯根表面の露出。
食床	牙宣; 食床; 齒挺; 齒齦宣露	3.6.43	gum atrophy	exposure of the root surfaces of teeth due to the drawing back of gingivae from the necks of teeth	歯頸部からの歯肉の後退による歯根表面の露出。
齒挺	牙宣; 食床; 齒挺; 齒齦宣露	3.6.43	gum atrophy	exposure of the root surfaces of teeth due to the drawing back of gingivae from the necks of teeth	歯頸部からの歯肉の後退による歯根表面の露出。
齒齦宣露	牙宣; 食床; 齒挺; 齒齦宣露	3.6.43	gum atrophy	exposure of the root surfaces of teeth due to the drawing back of gingivae from the necks of teeth	歯頸部からの歯肉の後退による歯根表面の露出。
牙疳		3.6.44	ulcerative gingivitis	a disease marked by painful inflammation of the gums with necrosis and fetid discharge	壊死および口臭排出を伴う歯肉の有痛性炎症を特徴とする疾患。
口糜	口糜; 口疳	3.6.45	oral erosion	a condition marked by multiple spots of erosion on the buccal mucosa	頬粘膜上にある多数の斑点状のびらんを特徴とする状態。
口疳	口糜; 口疳	3.6.45	oral erosion	a condition marked by multiple spots of erosion on the buccal mucosa	頬粘膜上にある多数の斑点状のびらんを特徴とする状態。
骨槽風		3.6.46	maxillary osteomyelitis	an inflammatory disease of the jaw bone characterized by local death and separation of tissue with pus discharge	顎の骨の炎症性疾患。膿排出を伴い、組織の局所的死亡および分離を特徴とする。
脣風		3.6.47	exfoliative cheilitis	inflammation of the lip with cracks and exudation	ひび割れ、滲出を伴う口唇の炎症。
脣疔		3.6.48	lip pustule	furuncle on the lip or at the corner of the mouth, small but deep-rooted, with pustule formation	小さいが根強い、口唇または口角の癤。膿疱形成を伴う。
脣菌		3.6.49	lip cancer	a mushroom-like cancer occurring on the lip	口唇に発生する茸状の癌。
口僻		3.6.50	deviated mouth	a diseased state characterized by skewing of the mouth to one side of the face with drooling from the corner of the mouth and inability to close the eye	口部の顔面の一方への歪みを特徴とする病的状態。口角からの流涎、閉眼不能を伴う。
骨傷科		**3.7.0**	**Orthopedics and Traumatology**		
落枕		3.7.1	stiff neck	acute inflammation or sprain of the soft tissue of the neck	頸部軟部組織の急性炎または捻挫。
筋傷	筋傷; 傷筋	3.7.2	sinew injury	injury of the subcutaneous tissue, including muscle, sinew, tendon sheath, ligament, joint capsule, synovial bursa, intervertebral disc, peripheral nerve, and blood vessel	筋肉、筋、腱鞘、靭帯、関節包、滑液包、椎間板、末梢神経、血管など、皮下組織の損傷。
傷筋	筋傷; 傷筋	3.7.2	sinew injury	injury of the subcutaneous tissue, including muscle, sinew, tendon sheath, ligament, joint capsule, synovial bursa, intervertebral disc, peripheral nerve, and blood vessel	筋肉、筋、腱鞘、靭帯、関節包、滑液包、椎間板、末梢神経、血管など、皮下組織の損傷。
筋斷		3.7.3	ruptured sinew	complete or partial disruption of muscle and tendon	筋肉および腱の完全または部分断裂。
筋縮		3.7.4	contracted sinew	permanent shortening of muscle with deformity and dysfunction	変形および機能不全を伴う筋肉の永続的な短縮。
筋粗		3.7.5	thickened sinew	thickening of muscle tendon after injury	損傷後の筋腱の肥厚。
筋攣		3.7.6	hypertonicity of the sinews	muscular spasm and contracture of a limb, with difficulty in relaxation and movement	弛緩および運動の困難を伴う、筋肉抽搐および四肢の拘急。
筋痹		3.7.7	impediment of the sinew	an impediment disease caused by wind-cold-dampness chiefly involving the sinews	主に筋に関与する、風寒湿により起こる障害性疾患。
扭傷		3.7.8	sprain	soft tissue injury attributed to a sudden or violent twist or wrench of a joint	突発の、または激しい関節のねじれまたはひねりによる軟部組織損傷。
其他		**3.8.0**	**Others**		
所生病		3.8.1	disease produced by viscus	disease of a certain meridian/channel induced by the disease or syndrome of the relevant visceral organ, also known as viscus-induced disease	関連臓器の疾患または症候により誘発される、特定の経穴の疾患。臓による疾患としても知られる。
是動病		3.8.2	disease transmitted by meridian	disease of a certain visceral organ induced by the disease or syndrome of the relevant meridian/channel, also known as meridian/channel-induced disease	関連臓器の疾患または症候により誘発される、特定の経穴の疾患。臓による疾患としても知られる。
六經病		3.8.3	six-meridian disease	a general term for diseases of greater yang, yang brightness, lesser yang, greater yin, lesser yin and reverting yin	太陽、陽明、少陽、太陰、少陰、逆陰の疾患の一般用語。

検索表示（原表示の複数語を分離）	IST原表示	Code	Term	Definition/Description	定義/記述
脾受寒表寒病		3.8.4	exterior cold disease induced from the spleen affected by cold	exterior cold disease induced by cold affecting the spleen in a lesser yang person	少陽人の、脾に罹患する寒により誘発される表寒病。
胃受熱裏熱病		3.8.5	interior febrile disease induced from the stomach affected by heat	interior febrile disease induced by heat affecting the stomach in a lesser yang person	少陽人の、胃に罹患する熱により誘発される裏熱病。
腎受熱表熱病		3.8.6	exterior febrile disease induced from the kidney affected by heat	exterior febrile disease induced by heat affecting the kidney in a lesser yin person	少陰人の、腎に罹患する熱により誘発される表熱病。
胃受寒裏寒病		3.8.7	interior cold disease induced from the stomach affected by cold	interior cold disease induced by cold affecting the stomach in a lesser yin person	少陰人の、胃に罹患する寒により誘発される裏寒病。
肝受熱裏熱病		3.8.8	interior febrile disease induced from the liver affected by heat	interior febrile disease induced by heat affecting the liver in a greater yin person	太陰人の、肝に罹患する熱により誘発される裏熱病。
胃脘受寒表寒病		3.8.9	exterior cold disease induced from the stomach duct affected by cold	exterior cold disease induced by cold affecting the stomach duct in a greater yin person	太陰人の、胃に罹患する寒により誘発される表寒病。
外感腰脊病		3.8.10	lumbar vertebrae disease induced by exopathogen	lumbar vertebral disease induced by external pathogen in a greater yang person	太陽人の、外邪により誘発される腰椎疾患。
內觸小腸病		3.8.11	small intestine disease induced by endopathogen	small intestine disease induced by endogenous pathogen in a greater yang person	太陽人の、内邪により誘発される小腸疾患。
治療學		**4.0.0**	**THERAPEUTICS**		
治則		**4.1.0**	**therapeutic principle**	a general rule that should be followed in treating disease	疾患治療の際に従うべき一般規則。
治本	治本;本治法	4.1.1	treat the root	treat the primary aspect of a disease	疾患の根本的な面を治療すること。
本治法	治本;本治法	4.1.1	treat the root	treat the primary aspect of a disease	疾患の根本的な面を治療すること。
治標	治標;標治法	4.1.2	treat the tip	treat the secondary aspect of a disease	疾患の二次的な面を治療すること。
標治法	治標;標治法	4.1.2	treat the tip	treat the secondary aspect of a disease	疾患の二次的な面を治療すること。
正治	正治;正治法	4.1.3	routine treatment	use of medicines opposite in nature to the disease, e.g., treating heat syndrome with medicines cold in nature	疾患に対して逆の性質の薬物を使用すること。例、熱症を、寒の性質を持つ薬物で治療する。
正治法	正治;正治法	4.1.3	routine treatment	use of medicines opposite in nature to the disease, e.g., treating heat syndrome with medicines cold in nature	疾患に対して逆の性質の薬物を使用すること。例、熱症を、寒の性質を持つ薬物で治療する。
寒者熱之		4.1.4	treat cold with heat	treat cold conditions with warming or heating method	寒証を温熱法で治療すること。
熱者寒之		4.1.5	treat heat with cold	treat heat conditions with cold or cooling method	熱証を寒涼法で治療すること。
虛則補之		4.1.6	treat deficiency by tonification	treat deficiency conditions by supplementation or tonification	虚証を補法により治療すること。
實則瀉之		4.1.7	treat excess by purgation	treat excess conditions by purgation or reduction	実証を下法により治療すること。
反治	反治;反治法	4.1.8	paradoxical treatment	use of medicines similar in nature to the disease	疾患と性質が同じ薬物を使用すること。
反治法	反治;反治法	4.1.8	paradoxical treatment	use of medicines similar in nature to the disease	疾患と性質が同じ薬物を使用すること。
寒因寒用		4.1.9	treating cold with cold	treating pseudo-cold symptoms with medicines cool or cold in nature	仮寒を、涼または寒の性質を持つ薬物で治療すること。
熱因熱用		4.1.10	treating heat with heat	treating pseudo-heat symptoms with medicines warm or hot in nature	仮熱を、温または熱の性質を持つ薬物で治療すること。
通因通用		4.1.11	treating the unstopped by unstopping	applying unstopping method to unstopped conditions , e.g., using purgation to treat diarrhea caused by food stagnation	通利の症状に通利法を適用すること。例、食物鬱滞による下痢の治療に瀉下薬を用いる。
塞因塞用		4.1.12	treating the stopped by stopping	applying stopping method to stopped conditions , e.g., using tonification to treat constipation due to qi deficiency	閉塞の症状に補塞法を適用すること。例、気虚による便秘の治療に補法を使用する。
扶正祛邪		4.1.13	reinforce the healthy qi and eliminate the pathogenic factors	two general principles of treatment — to reinforce the patient's healthy qi and to eliminating the invading pathogenic factors, which can be applied separately or in combination according to the particular condition of the case	治療の2つの一般原則。— 患者の正気を強めることと、侵入している病邪を排除すること。これらは、症例の状態によって、別々に、または組み合わせて適用できる。
攻補兼施		4.1.14	treat with both elimination and reinforcement	a principle of treatment with eliminating and reinforcing methods used in combination, suitable for patients with a weak constitution suffering from an excess syndrome	攻邪と扶正を併用する治療の原則。実証を起こしている虚弱体質の患者に適する。
誤治		4.1.15	erroneous treatment	incorrect treatment that may be ineffective and/or that results in harmful effects	無効となるまたは有害な影響を与える可能性のある、間違った治療。
異病同治		4.1.16	same treatment for different diseases	applying the same method of treatment to patients with different kinds of disease but have the same pattern/syndrome	異なる種類の疾患であるが同じ証を有する患者に、同じ治療法を適用すること。
同病異治		4.1.17	different treatments for the same disease	applying different methods of treatment to the same kind of disease but have different patterns/syndromes	同じ種類の疾患であるが異なる証を有する患者に、異なる治療法を適用すること。

検索表示（原表示の複数語を分離）	IST原表示	Code	Term	Definition/Description	定義/記述
先急後緩		4.1.18	treating the acute before the chronic	the acute condition should be treated prior to the chronic condition	急性疾患は慢性疾患よりも先に治療すべきであること。
先表後裏		4.1.19	treating the exterior before the interior	the exterior syndrome should be first treated and then the interior syndrome	表症は最初に治療すべきであり、裏症はその後で治療すべきであること。
加減方		4.1.20	modified formula	a formula derived from the original one with some addition or reduction of ingredients	本来の処方から成分をいくつか追加または削除した処方。
合方		4.1.21	combined formula	a compound formula composed of two or more formulae in combination	2以上の処方を組み合わせた薬物の処方。
治法		**4.2.0**	**Method of Treatment**		
治法		4.2.1	method of treatment	any specific intervention derived from a principle of treatment	治療の原則から派生したすべての特定の介入法。
八法		4.2.2	eight methods	a collective term for diaphoresis, emesis, purgation, mediation, warming, clearing, tonification and resolution	和法、温法、清法、補法、汗法、吐法、下法、消法の総称。
扶正		4.2.3	reinforce the healthy qi	a general term for treating disease by strengthening the healthy qi of the body	体の正気を強めて治療することの一般用語。
祛邪		4.2.4	eliminate the pathogenic factors	a general term for treating disease by dispelling the pathogenic factors from the body	体から病邪を排除して治療することの一般用語。
汗法		4.2.5	promoting sweating	one of the eight principal therapeutic methods used for releasing exterior pattern/syndrome, also called diaphoresis	治療八法の1つ。表症を解除するために用いられる。発汗療法とも呼ばれる。
解表		4.2.6	release the exterior	a general term for dispelling pathogenic factors from the exterior portion of the body	体の表面から病邪を排除することの一般用語。
發汗解表		4.2.7	promote sweating to release the exterior	a therapeutic method to release exterior pattern/ syndrome by using diaphoretics	発汗剤により表証を解除する治療法。
辛溫解表		4.2.8	release the exterior with pungent-warm	a therapeutic method to treat wind-cold exterior pattern/syndrome by using pungent-warm exterior-releasing medicinals	辛温解表薬を用いた風寒表証の治療法。
辛涼解表		4.2.9	release the exterior with pungent-cool	a therapeutic method to release wind-heat exterior pattern/syndrome by using pungent-cool medicinals	辛涼薬を用いた風熱表証の治療法。
解肌		4.2.10	release the flesh	a general term for dispelling pathogens from the superficial flesh	肌から病邪を排除することの一般用語
透表		4.2.11	outthrust through the exterior	a therapeutic method used to expel pathogens from the body in the treatment of externally contracted disease at the early stage with an exterior pattern/ syndrome	表証で、外感病の初期治療において、体から病邪を除去するために使用される治療法。
透疹		4.2.12	outthrust rashes	a therapeutic method used to promote skin eruption in measles for preventing complications, the same as promoting eruption	はしかの発疹を促進し、合併症を予防するために使用される治療法。発疹促進と同義。
祛風		4.2.13	dispel wind	a general term for relieving external wind pattern/ syndrome	外風証を緩和することの一般用語。
疏風		4.2.14	disperse wind	a therapeutic method used to disperse external wind pathogen for releasing exterior pattern/syndrome	外風邪を消散させ表証を解除するために使用される治療法。
散寒		4.2.15	dissipate cold	a therapeutic method of dissipating external cold pathogen	外寒邪を消散させる治療法。
疏風泄熱		4.2.16	disperse wind and discharge heat	a therapeutic method to treat externally contracted wind with interior heat by using exterior-releasing medicinals and heat-clearing medicinals in combination	解表薬と清熱薬を併用し、裏熱を伴う外感風を治療する方法。
疏表潤燥		4.2.17	disperse from the exterior and moisten dryness	a therapeutic method of using exterior-releasing and dryness-moistening medicinals, indicated in the treatment of wind-dryness attacking the exterior	解表および潤燥薬を用いた治療法。表の風燥 の治療に適用される。
疏散風熱		4.2.18	disperse wind-heat	a therapeutic method to treat external wind-heat contraction by using pungent-cool exterior-releasing medicinals	辛涼解表薬を用いて外感風熱を治療する方法。
扶正解表		4.2.19	reinforce the healthy qi and release the exterior	a therapeutic method of combined use of exterior-releasing medicinals and tonifying medicinals to treat exterior pattern/syndrome occurring in a deficiency condition	解表薬と補薬を併用して、虚証で生じる表証を治療する方法。
宣肺		4.2.20	diffuse the lung	a therapeutic method to diffuse the lung and restore the normal qi movement of the lung	肺を広げ、肺の正常な気機を回復させる治療法。
宣肺止咳平喘		4.2.21	diffuse the lung to suppress cough and to calm panting	restoring the normal qi movement of the lung to suppress cough and to calm panting	肺の正常な気機を回復させ、咳嗽を抑え、あえぎ呼吸を鎮めること。
宣肺止咳		4.2.22	diffuse the lung to suppress cough	restoring the normal qi movement of the lung to suppress cough	肺の正常な気機を回復させ、咳嗽を抑えること。
宣肺平喘		4.2.23	diffuse the lung to calm panting	restoring the normal qi movement of the lung to calm panting	肺の正常な気機を回復させ、あえぎ呼吸を鎮めること。
宣肺化痰		4.2.24	diffuse the lung to resolve phlegm	restoring the normal qi movement of the lung to expel the phlegm	肺の正常な気機を回復させ、痰を排出すること。
通鼻	通鼻;通(鼻)竅	4.2.25	relieve the stuffy nose	a therapeutic method that relieves nasal obstruction	鼻閉を緩和する治療法。
通竅	通鼻;通(鼻)竅	4.2.25	relieve the stuffy nose	a therapeutic method that relieves nasal obstruction	鼻閉を緩和する治療法。
通鼻竅	通鼻;通(鼻)竅	4.2.25	relieve the stuffy nose	a therapeutic method that relieves nasal obstruction	鼻閉を緩和する治療法。
利咽		4.2.26	soothe the throat	a therapeutic method that relieves a sore throat	喉の痛みを緩和する治療法 。
清法		4.2.27	clearing method	one of the eight principal therapeutic methods by administering medicinals of cool or cold nature to treat fire or heat pattern/syndrome	治療八法の1つ。涼または寒の性質を持つ薬剤を投与して、火証または熱証を治療すること。
清熱		4.2.28	clear heat	a therapeutic method of clearing pathogenic heat	熱邪を解除する治療法。
瀉火		4.2.29	purge fire	a therapeutic method of removing pathogenic fire by using bitter-cold medicinals	苦寒薬を用いて火邪を除去する治療法。

検索表示（原表示の複数語を分離）	IST原表示	Code	Term	Definition/Description	定義/記述
清氣	清氣; 清氣泄熱	4.2.30	clear the qi aspect	a therapeutic method of clearing pathogenic heat from the qi aspect	気分から熱邪を解除する治療法。
清氣泄熱	清氣; 清氣泄熱	4.2.30	clear the qi aspect	a therapeutic method of clearing pathogenic heat from the qi aspect	気分から熱邪を解除する治療法。
清心		4.2.31	clear the heart	a therapeutic method to treat pathogen entering the heart or pericardium in a warm disease	温疾患において心臓または心包に侵入している病邪を治療する治療法。
清宮		4.2.32	clear the pericardium	a therapeutic method of clearing pathogenic heat from the pericardium	心包から熱邪を解除する治療法。
清營		4.2.33	clear the nutrient aspect	a therapeutic method of clearing pathogenic heat from the nutrient aspect	営分から熱邪を解除する治療法。
瀉心		4.2.34	purge the heart	a therapeutic method to remove intense pathogenic heat or fire from the heart by using purgative medicines	下剤を用いて心臓から激しい熱邪または火邪を除去する治療法。
瀉肺		4.2.35	purge the lung	a therapeutic method of purging the lung of fire by using bitter-cold medicinals	苦寒薬を用いて肺から火邪を清瀉する治療法。
瀉肝		4.2.36	purge the liver	a therapeutic method of purging the liver of fire by using bitter-cold medicinals	苦寒薬を用いて肝臓から火邪を清瀉する治療法。
清氣涼營	清氣涼營; 氣營兩清	4.2.37	clear the qi aspect and cool the nutrient aspect	combined use of qi-clearing and nutrient-clearing methods to treat pathogenic heat in qi and nutrient aspects	清気法および清営法を併用して、気分および営分における熱邪を治療すること。
氣營兩清	清氣涼營; 氣營兩清	4.2.37	clear the qi aspect and cool the nutrient aspect	combined use of qi-clearing and nutrient-clearing methods to treat pathogenic heat in qi and nutrient aspects	清気法および清営法を併用して、気分および営分における熱邪を治療すること。
清營涼血		4.2.38	clear the nutrient aspect and cool the blood aspect	combined use of therapeutic methods of nutrient-clearing and blood-cooling methods to treat pathogenic heat in the nutrient and blood aspects	清営法および涼血法を併用して、営分および血分における熱邪を治療すること。
清營透疹		4.2.39	clear the nutrient aspect and promote eruption	a combined therapeutic method of clearing the nutrient of heat and promoting skin eruption	営分から熱邪を解除し、発疹を促進する併用治療法。
清熱涼血	清熱涼血; 涼血	4.2.40	clear heat to cool the blood	a therapeutic method of using heat-clearing and blood-cooling medicinals to treat blood heat pattern/ syndrome	清熱涼血薬を用いた血熱証の治療法。
涼血	清熱涼血; 涼血	4.2.40	clear heat to cool the blood	a therapeutic method of using heat-clearing and blood-cooling medicinals to treat blood heat pattern/ syndrome	清熱涼血薬を用いた血熱証の治療法。
透營轉氣	透營轉氣; 透熱轉氣	4.2.41	expel from the nutrient aspect through the qi aspect	a therapeutic method of outthrusting pathogenic heat from nutrient aspect to qi aspect and then expelling it out of the body	営分から気分へと熱邪を押し出し、その後体外に排出する治療法。
透熱轉氣	透營轉氣; 透熱轉氣	4.2.41	expel from the nutrient aspect through the qi aspect	a therapeutic method of outthrusting pathogenic heat from nutrient aspect to qi aspect and then expelling it out of the body	営分から気分へと熱邪を押し出し、その後体外に排出する治療法。
清營祛瘀		4.2.42	clear the nutrient aspect and eliminate stasis	a therapeutic method of jointly using heat-clearing detoxicating medicinals and blood-activating stasis-resolving medicinals to treat pattern/syndrome of heat toxin contending with blood	清熱解毒薬と活血化瘀薬を併用した、瘀血と相搏つ熱邪証の治療法
清熱生津		4.2.43	clear heat and engender fluid	a therapeutic method of using heat-clearing medicinals together with fluid-producing medicinals for treating fire or heat pattern/syndrome with fluid damage	清熱薬と生津薬を併用した、津の消耗を伴う火証または熱証の治療法。
清心火		4.2.44	clear heart fire	a therapeutic method of clearing the heart fire to treat exuberant heart fire pattern/syndrome	心火を解除して心火盛証を治療する方法。
清肝火		4.2.45	clear liver fire	a therapeutic method of clearing the liver fire to treat exuberant liver fire pattern/syndrome and pattern/syndrome of liver fire flaming upward	肝火を解除して、肝火盛証および肝火上炎証を治療する方法。
清肝		4.2.46	clear the liver	a therapeutic method of clearing the liver heat or fire	肝熱または肝火を解除する治療法
清熱利濕		4.2.47	clear heat and drain dampness	a therapeutic method of clearing heat and removing dampness through diuresis to treat accumulation of dampness-heat in the lower energizer by combined use of heat-clearing medicinals and dampness-draining medicinals	利尿により熱邪を解除し、湿邪を除去する治療法。清熱薬と利湿薬を併用して、下焦における湿熱の蓄積を治療する。
清胃火		4.2.48	clear stomach fire	a therapeutic method of clearing fire from the stomach to treat exuberant stomach fire	胃から火邪を解除し、胃火盛を治療する方法。
清胃		4.2.49	clear the stomach	a therapeutic method of clearing the stomach of heat or fire	胃から熱邪または火邪を解除する治療法。
清熱和胃		4.2.50	clear heat and harmonize the stomach	a therapeutic method to treat exuberant stomach fire by clearing heat and harmonizing the stomach	熱邪を解除し、胃を調和させることで、胃火盛を治療する方法。
制酸止痛		4.2.51	inhibit acidity to relieve pain	a therapeutic method to relieve epigastric pain by using acid-inhibiting medicinals	制酸薬を用いて胃痛を緩和する治療法。
清肺火	清肺火; 清肺	4.2.52	clear lung fire	a therapeutic method to treat exuberant fire in the lung by using lung-heat-clearing medicinals	肺清熱薬を用いて肺の火盛を治療する方法。
清肺	清肺火; 清肺	4.2.52	clear lung fire	a therapeutic method to treat exuberant fire in the lung by using lung-heat-clearing medicinals	肺清熱薬を用いて肺の火盛を治療する方法。
清相火	清相火; 清腎火	4.2.53	clear ministerial fire	a therapeutic method of clearing the kidney deficiency-fire	腎虚火を解除する治療法
清腎火	清相火; 清腎火	4.2.53	clear ministerial fire	a therapeutic method of clearing the kidney deficiency-fire	腎虚火を解除する治療法
清暑熱		4.2.54	clear summerheatheat	a therapeutic method for clearing pathogenic summerheat-heat	暑熱邪を解除する治療法
解暑		4.2.55	release summerheat	a therapeutic method for releasing summerheat	暑邪を解除する治療法

検索表示（原表示の複数語を分離）	IST原表示	Code	Term	Definition/Description	定義/記述
清暑益氣		4.2.56	clear summerheat and boost qi	a therapeutic method to treat pattern/syndrome of summerheat damaging fluid and qi by using heat-clearing and summerheat-resolving medicinals and qi-supplementing medicinals and fluid-engendering medicinals	津および気を消耗させる暑証を、清熱去暑薬と補気薬および生津薬を用いて治療する方法。
泄熱救津		4.2.57	discharge heat to preserve fluid	a therapeutic method of using heat-clearing medicinals and fluid-engendering medicinals to treat pattern/syndrome of fire-heat damaging fluid	津を消耗させる火熱証を、清熱薬と生津薬を用いて治療する方法。
輕清宣化		4.2.58	clear and disperse with lightness	a therapeutic method of using aromatic medicinals to resolve pathogenic dampness, indicated in the treatment of dampness stagnation pattern/syndrome	芳香薬を用いて、湿邪を解除する治療法。湿滞証の治療に適用される。
苦寒泄火		4.2.59	discharge fire with bitter-cold	a therapeutic method to discharge fire by using bitter-cold medicinals	苦寒薬を用いて火邪を排除する治療法。
苦寒清熱		4.2.60	clear heat with bitter-cold	a therapeutic method for clearing internal heat by using bitter-cold medicinals	苦寒薬を用いて内熱を解除する治療法。
苦寒清氣		4.2.61	clear qi with the bitter-cold	a therapeutic method of clearing pathogenic heat from the qi aspect by using bitter-cold medicinals	苦寒薬を用いて気分から熱邪を解除する治療法。
辛寒清氣		4.2.62	clear qi with the pungent-cold	a therapeutic method of clearing pathogenic heat from qi aspect by using pungent-cold medicinals	辛寒薬を用いて気分から熱邪を解除する治療法。
降火		4.2.63	downbear fire	a therapeutic method of using heat-clearing and fire-purging medicinals to treat upward invasion of heat-fire	清熱瀉火薬を用いて熱火邪の昇侵入を治療する方法
解毒		4.2.64	detoxify	(1) measure to lessen the virulence of pathogens; (2) a method of neutralizing the toxic property of poisons	(1) 病邪の毒性を低減させる方法；(2)毒物の毒性を中和する方法。
除疳熱		4.2.65	relieve (infantile) malnutrition fever	a therapeutic method for treating malnutritional fever in children	小児の栄養不良性発熱を治療する方法。
下法	下法; 瀉下(法)	4.2.66	purgation	one of the eight principal therapeutic methods used to relieve constipation, remove stagnant food, static blood, internal heat or excessive fluid through the bowels	治療八法の1つ。便秘を緩和し、鬱滞した食物、鬱血、内熱または過剰な津を、腸を介して除去すること。
瀉下	下法; 瀉下(法)	4.2.66	purgation	one of the eight principal therapeutic methods used to relieve constipation, remove stagnant food, static blood, internal heat or excessive fluid through the bowels	治療八法の1つ。便秘を緩和し、鬱滞した食物、鬱血、内熱または過剰な津を、腸を介して除去すること。
瀉下法	下法; 瀉下(法)	4.2.66	purgation	one of the eight principal therapeutic methods used to relieve constipation, remove stagnant food, static blood, internal heat or excessive fluid through the bowels	治療八法の1つ。便秘を緩和し、鬱滞した食物、鬱血、内熱または過剰な津を、腸を介して除去すること。
寒下	寒下; 瀉下泄熱	4.2.67	cold purgation	a therapeutic method of inducing purgation with cold-natured medicinals to treat excessive heat in the interior	寒の性質を持つ薬剤を用いて瀉下を起こし、内部の過剰な熱邪を治療する方法。
瀉下泄熱	寒下; 瀉下泄熱	4.2.67	cold purgation	a therapeutic method of inducing purgation with cold-natured medicinals to treat excessive heat in the interior	寒の性質を持つ薬剤を用いて瀉下を起こし、内部の過剰な熱邪を治療する方法。
溫下		4.2.68	warm purgation	a therapeutic method of inducing purgation and dissipating cold to treat interior excess pattern/ syndrome due to internal cold binding	瀉下を起こし、寒を消散させて内寒結による裏実証を治療する方法。
峻下	峻下; 急下	4.2.69	drastic purgation	a therapeutic method of treating interior excess pattern/syndrome with drastic purgatives	峻下剤を用いて裏実証を治療する方法。
急下	峻下; 急下	4.2.69	drastic purgation	a therapeutic method of treating interior excess pattern/syndrome with drastic purgatives	峻下剤を用いて裏実証を治療する方法。
輕下	輕下; 緩攻	4.2.70	mild purgation	a therapeutic method of inducing bowel movement with mild purgatives	穏やかな下剤を用いて腸の動きを起こさせる治療法
緩攻	輕下; 緩攻	4.2.70	mild purgation	a therapeutic method of inducing bowel movement with mild purgatives	穏やかな下剤を用いて腸の動きを起こさせる治療法
緩下		4.2.71	laxation	a therapeutic method of relieving constipation with laxatives	潤下薬を用いて便秘を緩和する治療法。
潤下		4.2.72	lubricant laxation	a therapeutic method of inducing laxation with fluid-nourishing and lubricating medicinals to treat constipation due to intestinal dryness	補津潤滑薬を用いて便通を起こし、腸燥による便秘を治療する方法。
潤腸		4.2.73	moisten the intestines	a therapeutic method for constipation due to intestinal dryness by using moistening medicinals	加湿薬を用いて、腸燥による便秘を治療する方法。
通便		4.2.74	relax the bowels	therapeutic method for relieving constipation	便秘を緩和する治療法。
瀉下軟堅		4.2.75	soften hardness with purgation	a therapeutic method of removing hard fecal masses in the intestines by using purgatives	腸内の硬い糞便の塊を、下剤を用いて取り除く治療法。
瀉下攻積		4.2.76	remove accumulation with purgation	a therapeutic method of removing accumulated undigested food by using purgatives	蓄積された未消化の食物を、下剤を用いて取り除く治療法。
釜底抽薪		4.2.77	take away firewood from under cauldron	metaphorical expression for the method of discharging heat with purgation	瀉下薬で熱邪を解除する方法の比喩的な表現。
溫下寒積		4.2.78	remove cold accumulation with warm purgation	a therapeutic method of administering warming medicinals with purgatives for treating interior excess pattern/syndrome due to internal cold binding	温化薬と下剤を投与して、内寒結による裏実証を治療する方法。
瀉下逐水		4.2.79	expel water by purgation	a therapeutic method of eliminating water retention by using hydragogues	利尿薬を用いて水停を解除する治療法。
瀉下逐飲	瀉下逐飲; 攻逐水飲	4.2.80	expel retained fluid by purgation	a therapeutic method of expelling retained fluid by using hydragogues	利尿薬を用いて鬱滞した津を排出する治療法
攻逐水飲	瀉下逐飲; 攻逐水飲	4.2.80	expel retained fluid by purgation	a therapeutic method of expelling retained fluid by using hydragogues	利尿薬を用いて鬱滞した津を排出する治療法
增液潤下	增液潤下; 增液潤腸	4.2.81	increase humor to relax bowels	a therapeutic method to promote bowel movement by using fluid-nourishing and intestine-moistening medicinals	補津潤腸薬を用いて腸の動きを促進する治療法。
增液潤腸	增液潤下; 增液潤腸	4.2.81	increase humor to relax bowels	a therapeutic method to promote bowel movement by using fluid-nourishing and intestine-moistening medicinals	補津潤腸薬を用いて腸の動きを促進する治療法。

検索表示（原表示の複数語を分離）	IST原表示	Code	Term	Definition/Description	定義/記述
和法		4.2.82	harmonizing method	one of the eight principal therapeutic methods to regulate the functions of visceral organs for restoring their normal correlation or to mediate the part between the exterior and interior of the body for eliminating the pathogens	治療八法の1つ。内臓の機能を調節して正常な関係を回復させる、または体の外部と内部の間の部分を仲介して病邪を解除すること。
和血		4.2.83	harmonize the blood	a therapeutic method to relieve or cure blood disorders	血液疾患を緩和または治癒させる治療法 。
和營		4.2.84	harmonize the nutrient	a therapeutic method to regulate the nutrient aspect	営分を調節する治療法。
調和氣血		4.2.85	harmonize qi and blood	a therapeutic method of using qi-regulating and blood-activating medicinals to treat disharmony of qi and blood	理気活血薬を用いて気血失調を治療する方法。
調和營衛		4.2.86	harmonize the nutrient and defense	a therapeutic method to treat nutrient-defense disharmony	営衛不和を治療する方法。
理氣		4.2.87	regulate qi	a general term for treating disordered flow of qi, including stagnant flow and counterflow	気の流れの鬱滞や逆流など、異常な気の流れの治療の一般用語。
行氣		4.2.88	move qi	a therapeutic method of relieving stagnation of qi	気の鬱滞を緩和する治療法。
理中		4.2.89	regulate the middle	a therapeutic method to tonify and regulate the spleen and stomach in deficiency-cold conditions	虚寒証において脾および胃を強化し調節する治療法。
和胃		4.2.90	harmonize the stomach	a therapeutic method to treat dysfunction of the stomach	胃の機能不全の治療法。
調和脾胃		4.2.91	harmonize the spleen and stomach	a therapeutic method of treating disharmony of the spleen and stomach by regulating their qi movement	気機を調節することで脾胃不和を治療する方法。
調和肝胃		4.2.92	harmonize the liver and stomach	a therapeutic method of soothing the liver and harmonizing the stomach to treat liver qi invading the stomach and liver-stomach disharmony	肝を疏散し、胃を調和させ、肝気犯胃および肝胃不和を治療する方法。
調和肝脾		4.2.93	harmonize the liver and spleen	a therapeutic method of soothing the liver, invigorating the spleen and regulating the qi activity to treat liver qi invading the spleen and liver-spleen disharmony	肝を疏散し、脾を活性化し、気の活動を調節して、肝気犯脾および肝脾不和を治療する方法。
和解表裏		4.2.94	harmonize and release the exterior and interior	a therapeutic method of treating mild cases of dual exterior-interior pattern/syndrome	軽度の表裏倶証の治療法。
和解少陽		4.2.95	harmonize and release the lesser yang	a therapeutic method of treating an externally contracted febrile disease by administering medicinals to combat the pathogens lingering at lesser yang (half-exterior and half-interior of the body)	少陽（体の半表半裏）に留まっている病邪と戦う薬剤を投与することで外感熱性疾患を治療する方法。
交通心腎		4.2.96	coordinate the heart and kidney	a therapeutic method of treating heart-kidney non-interaction by clearing heart fire and nourishing kidney yin	心火を解除し、腎陰を補うことで心腎不交を治療する方法。
開達膜原		4.2.97	open onto the pleurodiaphragmatic interspace	a therapeutic method of eliminating filthy turbid pathogens hidden in the pleuro-diaphragmatic space	穢濁邪伏膜原を解除する治療法。
表裏雙解		4.2.98	release both the exterior and interior	a general term for therapeutic methods that eliminate pathogens from both the exterior and interior parts of the body, indicated in the treatment of dual disease of the exterior and interior	体の外部および内部から病邪を解除する治療の一般用語。表裏同病の治療に適用される。
祛濕		4.2.99	dispel dampness	a general term for various measures to treat dampness pattern/syndrome, including resolving dampness by using aromatics, eliminating dampness by using bitter-cold medicinals, and removing dampness through diuresis	湿証のさまざまな治療法の一般用語。芳香薬を用いた湿邪の解除、苦寒薬を用いた湿邪の解除、利尿による湿邪の除去など。
清熱除濕		4.2.100	clear heat and eliminate dampness	a therapeutic method of treating dampness-heat in the upper and middle energizers by combined use of heat-clearing medicinals and dampness-resolving medicinals	清熱薬と化湿薬を併用して、上焦および中焦の湿熱を治療する方法。
瀉肝除濕		4.2.101	purge the liver and eliminate dampness	a therapeutic method of treating dampness-heat in the liver and its meridian	肝およびその経絡の湿熱を治療する方法。
散寒祛濕		4.2.102	dissipate cold and dispel dampness	a therapeutic method of treating cold-dampness obstruction pattern/syndrome by using pungent-warm medicinals to dispel cold and dry dampness	辛温薬を用いて、寒邪を消散させ、湿邪を乾燥させて、寒湿阻証を治療する方法。
化濕		4.2.103	resolve dampness	one of the measures to dispel dampness by using aromatics with warm nature and drying effect	温の性質を持ち、乾燥効果を有する芳香薬を用いて湿邪を消散させる方法の1つ。
清熱化濕		4.2.104	clear heat and resolve dampness	a therapeutic method of treating dampness-heat pattern/syndrome by using heat-clearing medicinals and dampness-resolving medicinals in combination	清熱薬と化湿薬を併用して湿熱証を治療する方法。
祛暑化濕		4.2.105	dispel summerheat and resolve dampness	a therapeutic method of combined use of summerheat-clearing medicinals and dampness-resolving medicinals for treating summerheat dampness pattern/syndrome	清暑剤と化湿薬を併用して、暑湿証を治療する方法。
醒脾化濕		4.2.106	enliven the spleen and resolve dampness	a therapeutic method to treat stagnancy of dampness due to spleen dysfunction	脾臓機能不全による湿邪鬱滞の治療法。
芳香化濕		4.2.107	resolve dampness with aroma	a therapeutic method of using aromatics to treat dampness pattern/syndrome	芳香薬を用いた湿証の治療法。
芳香化濁		4.2.108	resolve turbidity with aroma	a therapeutic method of using aromatics to treat dampness-turbidity pattern/syndrome	芳香薬を用いた湿濁証の治療法。
芳香辟穢		4.2.109	dispel filth with aroma	a therapeutic method of using aromatics to remove filthy turbidity	芳香薬を用いて穢濁を除去する治療法。
苦寒直折		4.2.110	direct repulsion with bitter-cold	a therapeutic method to directly purge heat and downbear fire by using bitter-cold medicinals	苦寒薬を用いて直接熱邪を清瀉し火邪を降濁する治療法。
化濕行氣		4.2.111	resolve dampness to move qi	a therapeutic method to treat qi stagnation by resolving dampness-turbidity	湿濁を除去することにより気滞を治療する方法。
燥濕		4.2.112	dry dampness	one of the measures to dispel dampness by using desiccating medicinals	乾燥薬を用いて湿邪を消散させる方法の1つ。

検索表示（原表示の複数語を分離）	IST原表示	Code	Term	Definition/Description	定義/記述
苦溫燥濕		4.2.113	dry dampness with bitter-warm	a therapeutic method of treating cold-dampness pattern/syndrome by using bitter-warm medicinals	苦温薬を用いて寒湿証を治療する方法。
清熱燥濕		4.2.114	clear heat and dry dampness	a therapeutic method for relieving accumulation of dampness-heat	湿熱の蓄積を緩和する治療法。
燥濕健脾		4.2.115	dry dampness to fortify the spleen	a therapeutic method of administering pungent-flavored drying medicinals to eliminate dampness for invigorating the spleen, indicated in the treatment of dampness encumbering	辛味の乾燥薬を投与し、湿邪を解除して、脾を活気づける治療法。湿困脾陽の治療に適用される。
利濕		4.2.116	drain dampness	one of the measures to dispel dampness by promoting diuresis	利尿を促進することで湿邪を消散させる 方法の1つ。
利水滲濕		4.2.117	induce diuresis to drain dampness	a therapeutic method to treat internal retention of dampness by using diuretics	利尿薬を用いて湿邪の内部鬱滞を治療する方法。
淡滲利濕	淡滲利濕; 淡滲祛濕	4.2.118	drain dampness with bland	a therapeutic method of removing pathogenic water-dampness by using sweet- or bland-tasting diuretics	甘いまたは刺激性の低い利尿薬を用いて、湿邪を 除去する治療法
淡滲祛濕	淡滲利濕; 淡滲祛濕	4.2.118	drain dampness with bland	a therapeutic method of removing pathogenic water-dampness by using sweet- or bland-tasting diuretics	甘いまたは刺激性の低い利尿薬を用いて、湿邪を 除去する治療法
利水消腫		4.2.119	induce diuresis to alleviate edema	a therapeutic method to treat edema by using diuretics	利尿薬を用いた水腫の治療法。
分消上下	分消上下; 分消走泄	4.2.120	separate elimination from upper and lower	a therapeutic method of using medicinals to open the upper, diffuse the middle and drain the lower for treating the condition in which the triple energizer's qi movement is obstructed by warm-heat and phlegm- dampness	薬剤を用いて上焦を開き、中焦を広げ、下焦を排液して、三焦の気機が温熱および痰湿により妨げられている状態を治療する方法。
分消走泄	分消上下; 分消走泄	4.2.120	separate elimination from upper and lower	a therapeutic method of using medicinals to open the upper, diffuse the middle and drain the lower for treating the condition in which the triple energizer's qi movement is obstructed by warm-heat and phlegm- dampness	薬剤を用いて上焦を開き、中焦を広げ、下焦を排液して、三焦の気機が温熱および痰湿により妨げられている状態を治療する方法。
化飮		4.2.121	resolve retained fluid	any therapeutic method of dispelling retained fluid	鬱滞した津を消散させる全ての治療法。
祛痰		4.2.122	dispel phlegm	a general term for therapeutic measures to treat phlegm pattern/syndrome, such as resolving phlegm or eliminating phlegm	痰証の治療法の一般用語。痰の解消または痰の解除など。
化痰		4.2.123	resolve phlegm	one of the measures to dispel phlegm in which the phlegm is disintegrated and dissolved	痰を消散させる方法の1つ。痰は分解され溶解する。
燥濕化痰		4.2.124	dry dampness to resolve phlegm	a therapeutic method of treating dampness-phlegm pattern/syndrome by using medicinals bitter in taste and drying in action	苦味で乾燥作用のある薬剤を用いて湿痰証を治療する方法。
清化熱痰		4.2.125	clear and resolve heat-phlegm	a therapeutic method of combined use of heat-clearing and phlegm-resolving medicinals for treating heat- phlegm pattern/syndrome	清熱化痰薬を併用して、熱痰証を治療する方法。
溫化寒痰		4.2.126	warm and resolve cold-phlegm	a therapeutic method of using yang-warming, cold-dispelling and phlegm-resolving medicinals to treat cold -phlegm pattern/syndrome	温陽、去寒、化痰薬を用いて、寒痰証を治療する方法。
溫肺化痰		4.2.127	warm the lung and resolve phlegm	a therapeutic method to treat accumulation of cold-phlegm in the lung by using warming and phlegm-resolving medicinals	温化化痰薬を用いて、肺の寒痰蓄積を治療する方法。
溫肺化飮		4.2.128	warm the lung and resolve fluid retention	a therapeutic method to treat retention of cold fluid in the lung by using warming and fluid-resolving medicinals	温化化津薬を用いて、肺の寒津鬱滞を治療する方法。
熄風化痰		4.2.129	extinguish wind and resolve phlegm	a therapeutic method to treat wind-phlegm pattern/ syndrome by combined use of wind-extinguishing and phlegm-resolving medicinals	熄風薬と化痰薬を併用して風痰証を治療する方法。
祛風痰		4.2.130	dispel wind-phlegm	a therapeutic method of treating pattern/syndrome of phlegm combined with pathogenic wind or wind-heat	風邪または風熱邪と組み合わさった痰証を治療する方法。
潤燥化痰		4.2.131	moisten dryness to resolve phlegm	a therapeutic method to treat dryness-phlegm pattern/ syndrome	燥痰証の治療法。
降氣化痰	降氣化痰; 下氣消痰	4.2.132	direct qi downward to resolve phlegm	a therapeutic method of combined use of qi-downbearing medicinals and phlegm-resolving medicinals to treat reverse flow of qi due to phlegm obstruction	降気薬および化痰薬を併用して、気滞による痰の逆流を治療する方法。
下氣消痰	降氣化痰; 下氣消痰	4.2.132	direct qi downward to resolve phlegm	a therapeutic method of combined use of qi-downbearing medicinals and phlegm-resolving medicinals to treat reverse flow of qi due to phlegm obstruction	降気薬および化痰薬を併用して、気滞による痰の逆流を治療する方法。
降逆止咳平喘		4.2.133	downbear counterflow to suppress cough and to calm panting	a therapeutic method for treating counterflow of lung qi with cough and dyspnea	咳嗽および呼吸困難を伴う肺気の逆流を治療する方法。
納氣平喘		4.2.134	promote qi absorption to calm panting	a therapeutic method for treating dyspnea due to kidney failing to receive qi	腎不納気による呼吸困難の治療法。
消痰軟堅		4.2.135	eliminate phlegm and soften hardness	a therapeutic method to treat hard nodules due to phlegm-turbidity accumulation	痰濁蓄積による硬い結節の治療法。
軟堅化痰		4.2.136	soften hardness and resolve phlegm	a therapeutic method to treat hard phlegmatic mass	硬い痰の塊の治療法。
清燥潤肺		4.2.137	clear dryness to moisten the lung	a therapeutic method to treat dryness-heat damaging the lung	燥熱傷肺の治療法。
潤肺止咳		4.2.138	moisten the lung to suppress cough	a therapeutic method of using yin-nourishing and lung-moistening medicinals to treat cough due to lung dryness	養陰および肺加湿薬を用いて、肺燥による咳嗽を治療する方法。
斂肺止咳		4.2.139	constrain the lung to suppress cough	a therapeutic method of using astringents to treat persistent unproductive cough due to lung deficiency	収斂薬を用いて、肺虚による持続性の乾性咳嗽を治療する方法。
瀉肺平喘		4.2.140	purge the lung to calm panting	a therapeutic method to treat dyspnea caused by accumulation of pathogens in the lung	肺の病邪蓄積による呼吸困難を治療する方法。

検索表示（原表示の複数語を分離）	IST原表示	Code	Term	Definition/Description	定義/記述
溫法		4.2.141	warming method	one of the eight principal therapeutic methods in which warming medicinals are used for treating cold pattern/syndrome	治療八法の1つ。温化薬を用いて寒証を治療すること。
溫補		4.2.142	warm tonification	a therapeutic method to treat deficiency-cold conditions by using warm-tonifying medicinals	温補薬を用いて、虚寒状態を治療する方法。
溫裏散寒	溫裏散寒; 溫裏祛寒	4.2.143	warm the interior to dissipate cold	a therapeutic method for treating interior-cold pattern/ syndrome	裏寒証の治療法。
溫裏祛寒	溫裏散寒; 溫裏祛寒	4.2.143	warm the interior to dissipate cold	a therapeutic method for treating interior-cold pattern/ syndrome	裏寒証の治療法。
溫裏		4.2.144	warm the interior	a general term for the methods of treating interior-cold pattern/syndrome	裏寒証の治療法の一般用語。
溫脾		4.2.145	warm the spleen	a therapeutic method to treat cold pattern/syndrome of the spleen by using yang-warming medicinals	温陽薬を用いて脾の寒証を治療する方法。
溫胃		4.2.146	warm the stomach	a therapeutic method to treat cold pattern/syndrome of the stomach by using warm-or hot-natured medicinals	温または熱の性質を持つ薬剤を用いて胃の寒証を治療する方法。
溫中		4.2.147	warm the middle	a therapeutic method to treat yang deficiency of the spleen and stomach with warm-tonifying medicinals	温補薬を用いて脾および胃の陽虚を治療する方法。
溫中散寒	溫中散寒; 溫[illegible]	4.2.148	warm the middle and dissipate cold	a therapeutic method to treat yang deficiency of the spleen and stomach by dispelling cold and harmonizing the middle [illegible]	温補薬を用いて寒を消散、中焦を調和させて、脾および胃の陽虚を治療する方法。
溫中祛寒	溫中散寒; 溫中祛寒	4.2.148	warm the middle and dissipate cold	a therapeutic method to treat yang deficiency of the spleen and stomach by dispelling cold and harmonizing the middle energizer with warm-tonifying medicinals	温補薬を用いて寒を消散、中焦を調和させ、脾および胃の陽虚を治療する方法。
溫中和胃		4.2.149	warm the middle to harmonize the stomach	a therapeutic method of using middle-warming and cold-dissipating medicinals to treat cold stagnation in the stomach	中焦温化、寒消散薬を用いて、胃の寒鬱滞を治療する方法。
溫中止嘔		4.2.150	warm the middle to check vomiting	a therapeutic method of warming the middle energizer to stop vomiting	中焦を温め、嘔吐を止める治療法。
溫肺		4.2.151	warm the lung	a therapeutic method to treat cold pattern/syndrome of the lung by using warming medicinals	温化薬を用いて肺の寒証を治療する方法。
溫肺散寒		4.2.152	warm the lung and dissipate cold	a therapeutic method to treat deficiency-cold of the lung by using warm-tonifying medicinals	温補薬を用いて肺の虚寒を治療する方法。
溫腎	溫腎; 溫腎陽	4.2.153	warm the kidney	a therapeutic method of invigorating kidney yang by using warm-tonifying medicinals	温補薬を用いて、腎陽を活気付ける治療法
溫腎陽	溫腎; 溫腎陽	4.2.153	warm the kidney	a therapeutic method of invigorating kidney yang by using warm-tonifying medicinals	温補薬を用いて、腎陽を活気付ける治療法
溫陽		4.2.154	warm yang	a therapeutic method of warming and unblocking yang qi	陽気を温化し通す治療法。
溫陽行水		4.2.155	warm yang to move water	a therapeutic method of using yang-warming medicinals and diuretics to treat water retention due to spleen-kidney yang deficiency	温陽薬と利尿薬を用いて、脾腎陽虚による水停を治療する方法。
暖宮		4.2.156	warm the uterus	a therapeutic method to treat cold congealing in the uterus	寒凝胞宮の治療法。
溫經		4.2.157	warm the meridian	a therapeutic method of warming and unblocking the meridian/channel	温化して経絡を通す治療法。
溫經止痛		4.2.158	warm the meridian to relieve pain	a therapeutic method for relieving pain caused by stagnation of pathogenic cold in the meridian/ channel	経絡における寒邪の鬱滞による疼痛を緩和する治療法。
溫經散寒		4.2.159	warm the meridian to dissipate cold	a therapeutic method of using yang-warming, cold-dissipating and collateral-opening medicinals to treat pattern/syndrome of cold congealing in the meridian/ channel	温陽、寒消散、経脈開通薬を用いて、経絡の寒凝証を治療する方法。
溫經回陽	溫經回陽; 溫經扶陽	4.2.160	warm the meridian to restore yang	a therapeutic method to treat impending collapse by warming and tonifying yang qi in the meridian/ channel	経絡の陽気を温め強めることで、切迫した虚脱を治療する方法。
溫經扶陽	溫經回陽; 溫經扶陽	4.2.160	warm the meridian to restore yang	a therapeutic method to treat impending collapse by warming and tonifying yang qi in the meridian/ channel	経絡の陽気を温め強めることで、切迫した虚脱を治療する方法。
回陽救逆	回陽救逆; 回陽	4.2.161	restore yang to save from collapse	a therapeutic method of using a large dose of warm- or hot-natured medicinals to prevent the patient from collapsing, the same as to restore yang	温または熱の性質を持つ薬剤を大量に使用して、患者が虚脱するのを防ぐ治療法。扶陽 と同義。
回陽	回陽救逆; 回陽	4.2.161	restore yang to save from collapse	a therapeutic method of using a large dose of warm- or hot-natured medicinals to prevent the patient from collapsing, the same as to restore yang	温または熱の性質を持つ薬剤を大量に使用して、患者が虚脱するのを防ぐ治療法。扶陽と同義。
溫經行滯		4.2.162	warm the meridian to move stagnation	a therapeutic method of using meridian/channel-warming medicinals and blood-activating and stasis-resolving medicinals to treat pattern/syndrome of congealing cold with blood stasis	正経温化薬および活血化瘀薬を用いて、寒凝血瘀証を治療する方法。
溫經養血		4.2.163	warm the meridian to nourish blood	a therapeutic method of using meridian/channel-warming medicinals and blood-nourishing medicinals to treat pattern/syndrome of blood deficiency with congealing cold	正経温化薬および補血薬を用いて、血虚寒凝証を治療する方法。
宣痹通陽		4.2.164	diffuse impediment and free yang	a therapeutic method of removing impediments to normalize the flow of yang-qi, as in the treatment of chest impediment	胸痹の治療で、障害を除去し、陽気の流れを正常化する治療法。
行氣寬中		4.2.165	move qi to soothe the middle	a therapeutic method of promoting the flow of qi to alleviate stuffiness in the spleen and stomach	気の流れを促進し、脾臓および胃の痞えを軽減する治療法。
行氣止痛		4.2.166	move qi to relieve pain	a therapeutic method of promoting the flow of qi to relieve pain caused by stagnant qi movement	気の流れを促進し、気機鬱滞による疼痛を緩和する治療法。

検索表示（原表示の複数語を分離）	IST原表示	Code	Term	Definition/Description	定義/記述
行氣寬胸		4.2.167	move qi to soothe the chest	a therapeutic method of relieving stuffiness of the chest by promoting the flow of qi	気の流れを促進することで胸の痞えを緩和する治療法。
理氣健脾		4.2.168	regulate qi and fortify the spleen	a therapeutic method to treat spleen failing in transportation by combined use of qi-moving and stagnation-relieving medicinals and qi-replenishing and spleen-invigorating medicinals	行気、鬱滞緩和薬と、益気活脾薬を併用して、脾失健運を治療する方法。
疏肝理氣		4.2.169	soothe the liver and regulate qi	a therapeutic method to regulate liver qi and relieve stagnation for treating liver qi stagnation pattern/ syndrome	肝気を調節し、鬱滞を緩和し、肝気滞証を治療する方法。
疏肝養血		4.2.170	soothe the liver and nourish the blood	a therapeutic method with combined use of liver-soothing medicinals and blood-nourishing medicinals for treating liver qi stagnation with blood deficiency	疏肝薬と補血薬を併用し、血虚を伴う肝気滞を治療する方法。
疏肝瀉火		4.2.171	soothe the liver and purge fire	a therapeutic method used to treat pattern/syndrome of stagnated liver qi transforming into fire	肝鬱化火証の治療法。
疏肝健脾		4.2.172	soothe the liver and fortify the spleen	a therapeutic method of regulating liver qi and strengthening spleen qi to harmonize the liver and spleen for treating pattern/syndrome of liver qi invading the spleen and pattern/syndrome of liver-spleen disharmony	肝気を調節し、脾気を強め、肝臓と脾臓を調和させて、肝気犯脾証および肝脾不和証を治療する方法。
疏肝理脾		4.2.173	soothe the liver and regulate the spleen	a therapeutic method of regulating the activities of the liver and spleen to restore their normal coordination for treating liver stagnation with spleen deficiency	肝と脾の活性を調節し、正常な協調性を回復させ、脾虚を伴う肝臓鬱滞を治療する方法。
疏肝和胃		4.2.174	soothe the liver and harmonize the stomach	a therapeutic method of regulating the activities of the liver and stomach for treating liver-stomach qi stagnation and liver-stomach disharmony	肝と胃の活性を調節し、肝胃気滞および肝胃不和を治療する方法。
疏肝		4.2.175	soothe the liver	a therapeutic method of restoring the normal functioning of stagnated liver qi	肝気欝結の正常な機能を回復させる治療法 。
降逆下氣	降逆下氣; 降氣; 下氣	4.2.176	direct qi downward	a therapeutic method of treating upward counterflow of qi in the lung and stomach manifested as cough, asthma, hiccupping or vomiting	咳嗽、喘息、吃逆または嘔吐として現れる、肺と胃の気の上向きの逆流を治療する方法。
降氣	降逆下氣; 降氣; 下氣	4.2.176	direct qi downward	a therapeutic method of treating upward counterflow of qi in the lung and stomach manifested as cough, asthma, hiccupping or vomiting	咳嗽、喘息、吃逆または嘔吐として現れる、肺と胃の気の上向きの逆流を治療する方法。
下氣	降逆下氣; 降氣; 下氣	4.2.176	direct qi downward	a therapeutic method of treating upward counterflow of qi in the lung and stomach manifested as cough, asthma, hiccupping or vomiting	咳嗽、喘息、吃逆または嘔吐として現れる、肺と胃の気の上向きの逆流を治療する方法。
降氣止呃	降氣止呃; 降逆止呃	4.2.177	direct qi downward to relieve hiccup	a therapeutic method of treating hiccups	吃逆の治療法。
降逆止呃	降氣止呃; 降逆止呃	4.2.177	direct qi downward to relieve hiccup	a therapeutic method of treating hiccups	吃逆の治療法。
破氣		4.2.178	break qi	a therapeutic method of using drastic medicinals to relieve stagnation of qi	作用が強烈な薬剤を用いて気鬱滞を緩和する治療法。
凉血止血		4.2.179	cool the blood to stop bleeding	a therapeutic method of using blood-cooling hemostatics to treat bleeding due to heat in the blood	涼血止血薬を用いて血中の熱邪による出血を治療する方法。
凉血散瘀		4.2.180	cool the blood and dissipate stasis	a therapeutic method for treating blood heat with blood stasis	血熱血瘀の治療法。
固衝止血		4.2.181	strengthen thoroughfare vessel to stop bleeding	a therapeutic method of astringing the thoroughfare and conception vessels to treat abnormal uterine bleeding or excessive menstrual flow	衝脈および任脈を収斂させ、異常な子宮出血や過剰な経血を治療する方法。
活血		4.2.182	activate blood	a general term for promoting blood flow in the treatment of blood stasis	血瘀の治療において血流を促進することの一般用語。
活血止痛		4.2.183	activate blood to relieve pain	a therapeutic method for treating painful conditions caused by blood stasis	血瘀による有痛状態の治療法。
活血化瘀		4.2.184	activate blood and resolve stasis	a general term for various therapeutic methods with blood-activating and stasis-resolving effects indicated in the treatment of blood stasis	血瘀の治療に適用される、活血化瘀効果のあるさまざまな治療法の一般用語。
活血行氣		4.2.185	activate blood and move qi	a therapeutic method to treat blood stasis with qi stagnation	血瘀気滞の治療法。
活血調經		4.2.186	activate blood to regulate menstruation	a therapeutic method of activating blood, removing stasis and regulating menstruation to treat menstrual irregularities due to blood stasis	血液を活性化させ、鬱滞を除去し、月経を調節して、血瘀による月経不順を治療する方法。
調經		4.2.187	regulate menstruation	a method used for treating menstrual irregularities	月経不順の治療に用いられる方法。
通經		4.2.188	unblock the meridian	a method to remove obstruction in meridian/channel	経絡の閉塞を除去する方法。
散瘀		4.2.189	dissipate (blood) stasis	a general term for removing static blood in the treatment of blood stasis	血瘀の治療において鬱血を除去することの一般用語。
破血逐瘀	破血逐瘀; 破瘀; 逐瘀	4.2.190	break blood and expel stasis	a therapeutic method to treat severe cases of blood stasis with intact health qi by using drastic blood-activating medicinals	作用が強烈な活血薬を用いて、完全な正気を伴う血瘀の重症例を治療する方法。
破瘀	破血逐瘀; 破瘀; 逐瘀	4.2.190	break blood and expel stasis	a therapeutic method to treat severe cases of blood stasis with intact health qi by using drastic blood-activating medicinals	作用が強烈な活血薬を用いて、完全な正気を伴う血瘀の重症例を治療する方法。

検索表示（原表示の複数語を分離）	IST原表示	Code	Term	Definition/Description	定義/記述
逐瘀	破血逐瘀; 破瘀; 逐瘀	4.2.190	break blood and expel stasis	a therapeutic method to treat severe cases of blood stasis with intact health qi by using drastic blood-activating medicinals	作用が強烈な活血薬を用いて、完全な正気を伴う血瘀の重症例を治療する方法。
舒筋活絡		4.2.191	relax sinews and activate collaterals	a therapeutic method used to treat blockage of meridian/channel qi with muscle contraction	筋収縮を伴う経絡脈の気の遮断に対する治療法。
舒筋和絡		4.2.191	relax sinews and activate collaterals	a therapeutic method used to treat blockage of meridian/channel qi with muscle contraction	筋収縮を伴う経絡脈の気の遮断に対する治療法。。
通絡		4.2.192	free the collateral vessels	a therapeutic method used to relieve obstruction of collateral vessels	絡脈の閉塞を緩和する方法。
祛風通絡		4.2.193	dispel wind to free the collateral vessels	a therapeutic method to treat numbness, paralysis or muscle contracture due to wind-dampness impeding the collateral/network vessels	絡脈の循行を妨害している風湿によるしびれ、麻痺、または筋痙縮の治療法
潛陽		4.2.194	subdue yang	a therapeutic method to treat ascendant hyperactivity of liver yang by administering heavy mineral and shell medicinals	重鉱物および貝殻類を投与することで肝陽上亢を治療する方法。
平肝潛陽		4.2.195	pacify the liver to subdue yang	a therapeutic method to treat ascendant hyperactivity of liver yang by using yin blood nourishing medicinals and heavy mineral and shell medicinals	補陰養血薬および重鉱物と貝殻類 を用いて肝陽上亢を治療する方法。
熄風		4.2.196	extinguish wind	a therapeutic method of using wind-extinguishing medicinals to relieve internal wind pattern/syndrome	熄風薬を用いて内風証を緩和する治療法。
熄風止痙	熄風止痙; 熄風解痙	4.2.197	extinguish wind to arrest convulsions	a therapeutic method of administering wind-extinguishing medicinals to relieve internal wind pattern/syndrome with convulsions	熄風薬を投与して、痙攣を伴う内風証を緩和する治療法。
熄風解痙	熄風止痙; 熄風解痙	4.2.197	extinguish wind to arrest convulsions	a therapeutic method of administering wind-extinguishing medicinals to relieve internal wind pattern/syndrome with convulsions	熄風薬を投与して、痙攣を伴う内風証を緩和する治療法。
凉肝熄風		4.2.198	cool the liver to extinguish wind	a therapeutic method of clearing and purging the liver of fire to calm and extinguish liver wind	肝から火邪を解除して清瀉し、肝風を鎮め消滅させる治療法。
滋陰熄風		4.2.199	nourish yin to extinguish wind	a therapeutic method of nourishing yin fluid to treat yin deficiency with stirring wind	陰津を補充して、陰虚動風を治療する方法。
淸熱熄風		4.2.200	clear heat to extinguish wind	a therapeutic method of treating internal wind due to serious impairment of yin fluid at the late stage of a febrile disease	熱性疾患の後期に、陰津の重度損傷による内風を治療する方法。
鎭肝熄風		4.2.201	calm the liver to extinguish wind	a therapeutic method of treating internal wind caused by hyperactivity of the liver	肝の活動亢進による内風の治療法。
補法		4.2.202	tonifying method	a general term for the methods to restore the healthy qi by using tonifying medicinals, one of the eight principal therapeutic methods, also called restoring method	補薬を用いて正気を回復させる方法の一般用語。治療八法の1つで、補益法とも呼ばれる。
補氣	補氣; 益氣	4.2.203	tonify qi	a therapeutic method to treat qi deficiency by using qi tonifying medicinals, the same as to replenish or restore qi	補気薬を用いて気虚を治療する方法。益気と同義。
益氣	補氣; 益氣	4.2.203	tonify qi	a therapeutic method to treat qi deficiency by using qi tonifying medicinals, the same as to replenish or restore qi	補気薬を用いて気虚を治療する方法。補気と同義。
大補元氣		4.2.204	greatly tonify the original qi	a therapeutic method of using potent qi-tonifying medicinals to treat critical cases of qi deficiency with hardly perceptible pulse	強力な補気薬を用いて、脈がほとんど触れない重度の気虚を治療する方法。
補氣壯陽		4.2.205	tonify qi and invigorate yang	a therapeutic method of treating deficiency of yang qi, especially related to the heart and kidney	特に心臓および腎に関係する陽気虚の治療法。
補氣生血		4.2.206	tonify qi and engender blood	a therapeutic method of treating deficiency of both qi and blood primarily due to qi deficiency	主として気虚による気と血の欠乏を治療する方法。
補益氣血		4.2.207	tonify qi and replenish blood	a therapeutic method to treat deficiency of both qi and blood by using qi-tonifying and blood-nourishing medicinals	補気補血薬を用いて気と血の欠乏を治療する方法。
升提中氣	升提中氣; 升擧中氣; 升陽; 升提	4.2.208	upraise the middle qi	a therapeutic method to treat sunken middle qi by using qi-tonifying medicinals with upraising actions	昇提作用を有する 補気薬を用いて中気下陷を治療する方法。
升擧中氣	升提中氣; 升擧中氣; 升陽; 升提	4.2.208	upraise the middle qi	a therapeutic method to treat sunken middle qi by using qi-tonifying medicinals with upraising actions	昇提作用を有する 補気薬を用いて中気下陷を治療する方法。
升陽	升提中氣; 升擧中氣; 升陽; 升提	4.2.208	upraise the middle qi	a therapeutic method to treat sunken middle qi by using qi-tonifying medicinals with upraising actions	昇提作用を有する 補気薬を用いて中気下陷を治療する方法。
升提	升提中氣; 升擧中氣; 升陽; 升提	4.2.208	upraise the middle qi	a therapeutic method to treat sunken middle qi by using qi-tonifying medicinals with upraising actions	昇提作用を有する 補気薬を用いて中気下陷を治療する方法。
補陽		4.2.209	tonify yang	a general term for the methods of treating yang deficiency conditions with tonifying medicinals	補薬により陽虚を治療する方法の一般用語。
壯陽		4.2.210	invigorate yang	a therapeutic method to strengthen yang qi of the body with warm-tonifying medicinals	温補薬により体の陽気を強める治療法。
溫補命門		4.2.211	warm and tonify the life gate	a therapeutic method to treat kidney yang deficiency by using yang-warming and kidney-tonifying medicinals	温陽補腎薬を用いて腎陽虚を治療する方法。
補益中氣		4.2.212	tonify and replenish the middle qi	a therapeutic method of tonifying qi, fortifying the spleen and replenishing the stomach for treating spleen and stomach qi deficiency with sunken middle qi	気を強め、脾臓を強化し、胃を補充して、中気下陷を伴う脾臓と胃の気虚を治療する方法。

検索表示（原表示の複数語を分離）	IST原表示	Code	Term	Definition/Description	定義/記述
補血; 養血		4.2.213	tonify blood	a therapeutic method to treat blood deficiency by using blood-tonifying medicinals, the same as to nourish or restore blood	補血薬を用いて血虚を治療する方法。養血と同義。
補養心血	補養心血; 養心	4.2.214	tonify and nourish heart blood	a therapeutic method to treat heart blood deficiency by using blood-tonifying and heart-nourishing medicinals, the same as to nourish heart	補血養心薬を用いて、心血虚を治療する方法。養心 と同義。
養心	補養心血; 養心	4.2.214	tonify and nourish heart blood	a therapeutic method to treat heart blood deficiency by using blood-tonifying and heart-nourishing medicinals, the same as to nourish heart	補血養心薬を用いて、心血虚を治療する方法。補養心血 と同義
補脾		4.2.215	tonify the spleen	a therapeutic method to treat diminished functional activities of the spleen by using tonifying medicinals	補脾薬を用いて、脾の機能活性低下を治療する方法。
健脾		4.2.216	fortify the spleen	a therapeutic method of invigorating the transporting and transforming functions of the spleen	脾の輸送および変換機能を活気付ける治療法。
健脾益氣		4.2.217	fortify the spleen and replenish qi	a therapeutic method to treat spleen qi deficiency by using spleen-fortifying and qi-replenishing medicinals	補脾益気薬を用いて、脾気虚を治療する方法。
健脾養血		4.2.218	fortify the spleen and nourish blood	a therapeutic method to treat pattern/syndrome of spleen deficiency with blood depletion	血液消耗を伴う脾虚証の治療法。
健脾扶陽		4.2.219	fortify the spleen and support yang	a therapeutic method to treat spleen yang deficiency by using spleen-fortifying and yang-reinforcing medicinals	健脾補陽薬を用いて脾陽虚を治療する方法。
健脾利濕		4.2.220	fortify the spleen and drain dampness	a therapeutic method of using spleen-tonifying and diuretic medicinals to treat spleen deficiency with dampness retention or dampness encumbering the spleen	補脾利尿薬を用いて、湿邪鬱滞を伴う脾虚または湿困脾を治療する方法。
健脾豁痰		4.2.221	fortify the spleen to sweep phlegm	a therapeutic method to treat phlegm-dampness pattern/syndrome through fortifying the spleen by using qi-tonifying medicinals	補気薬を用いて脾を強め、痰湿証を治療する方法。
健胃		4.2.222	invigorate the stomach	a therapeutic method of invigorating the stomach function to promote digestion	胃の機能を活気付け、消化を促進する治療法。
開胃		4.2.223	increase the appetite	a therapeutic method of stimulating the desire for food	食欲を刺激する治療法。
溫補脾胃		4.2.224	warm and tonify the spleen and stomach	a therapeutic method used for treating spleen and stomach yang deficiency pattern/syndrome	脾胃陽虚証の治療法。
補火助陽	補火助陽; 補腎火	4.2.225	tonify fire and assist yang	a therapeutic method to treat kidney yang deficiency by using yang-warming and kidney-tonifying medicinals; the same as to tonify the kidney fire	温陽補腎薬を用いて腎陽虚を治療する方法。補腎火と同義。
補腎火	補火助陽; 補腎火	4.2.225	tonify fire and assist yang	a therapeutic method to treat kidney yang deficiency by using yang-warming and kidney-tonifying medicinals; the same as to tonify the kidney fire	温陽補腎薬を用いて腎陽虚を治療する方法。補火助陽と同義。
柔肝	柔肝; 養肝	4.2.226	emolliate the liver	a therapeutic method to treat liver yin deficiency or liver blood deficiency by using liver blood-tonifying and liver yin-replenishing medicinals	肝活血補陰薬を用いて、肝陰虚または肝血虚を治療する方法。
養肝	柔肝; 養肝	4.2.226	emolliate the liver	a therapeutic method to treat liver yin deficiency or liver blood deficiency by using liver blood-tonifying and liver yin-replenishing medicinals	肝活血補陰薬を用いて、肝陰虚または肝血虚を治療する方法。
滋陰		4.2.227	enrich yin	a therapeutic method to treat yin deficiency by using medicinals to replenish yin fluid	陰液を補充する薬剤を用いて 陰虚を治療する方法。
滋陰補陽		4.2.228	enrich yin and tonify yang	a therapeutic method to treat dual deficiency of yin and yang	陰陽両虚の治療法。
滋陰潛陽		4.2.229	enrich yin and subdue yang	a therapeutic method of using yin-nourishing medicinals and heavy settling medicinals to treat yin deficiency with yang hyperactivity or deficiency yang floating upward	養陰薬および強い重鎮薬を用いて、陰虚陽亢または虚陽上浮を治療する方法。
滋陰抑陽		4.2.230	enrich yin to repress yang	a therapeutic method of supplementing yin fluid to suppress excessive yang qi for treating yin deficiency with exuberant yang	陰液を補充し、盛んな陽気を抑え、陰虚陽盛を治療する方法。
補心陰	補心陰; 養心陰	4.2.231	tonify the heart yin	a therapeutic method to treat heart yin deficiency by using heart yin tonifying medicinals, the same as to nourish the heart yin	補心陰薬を用いて心陰虚を治療する方法。nourish the heart yinと同義。
養心陰	補心陰; 養心陰	4.2.231	tonify the heart yin	a therapeutic method to treat heart yin deficiency by using heart yin tonifying medicinals, the same as to nourish the heart yin	補心陰薬を用いて心陰虚を治療する方法。nourish the heart yinと同義。
補肝陰	補肝陰; 養肝陰	4.2.232	tonify the liver yin	a therapeutic method of treating liver yin deficiency by using liver yin tonifying medicinals, the same as to nourish the liver yin	補肝陰薬を用いて、肝陰虚を治療する方法。 nourish the liver yinと同義。
養肝陰	補肝陰; 養肝陰	4.2.232	tonify the liver yin	a therapeutic method of treating liver yin deficiency by using liver yin tonifying medicinals, the same as to nourish the liver yin	補肝陰薬を用いて、肝陰虚を治療する方法。 nourish the liver yinと同義。
補肺		4.2.233	tonify the lung	a general term for therapeutic methods of using tonifying medicinals to treat deficiency patterns/ syndromes of the lung	補薬を用いて肺虚証を治療する方法の一般用語。
補肺陰	補肺陰; 養肺陰	4.2.234	tonify the lung yin	a therapeutic method to treat lung yin deficiency, the same as to nourish the lung yin	肺陰虚の治療法。**養肺陰**と同義。
養肺陰	補肺陰; 養肺陰	4.2.234	tonify the lung yin	a therapeutic method to treat lung yin deficiency, the same as to nourish the lung yin	肺陰虚の治療法。補肺陰 と同義。
潤肺		4.2.235	moisten the lung	a therapeutic method to treat lung dryness pattern/ syndrome by using moistening medicinals	化湿薬を用いて肺燥証を治療する方法。
生津		4.2.236	engender fluid	a therapeutic method to treat fluid depletion with fluid-nourishing medicinals	補陰薬を用いて津傷を治療する方法。
補腎		4.2.237	tonify the kidney	a general term for treating deficiency patterns/ syndromes of the kidney with tonifying medicinals	補薬を用いた腎虚証の治療法の一般用語。
補腎陰	補腎陰; 滋腎陰	4.2.238	tonify the kidney yin	a therapeutic method to treat kidney yin deficiency by using kidney yin tonifying medicinals, the same as to replenish the kidney yin	腎補陰薬を用いた腎陰虚の治療法。滋腎陰と同義。
滋腎陰	補腎陰; 滋腎陰	4.2.238	tonify the kidney yin	a therapeutic method to treat kidney yin deficiency by using kidney yin tonifying medicinals, the same as to replenish the kidney yin	腎補陰薬を用いた腎陰虚の治療法。補腎陰と同義。

検索表示（原表示の複数語を分離）	IST原表示	Code	Term	Definition/Description	定義/記述
養胃陰	養胃陰; 補胃陰; 養胃	4.2.239	nourish the stomach yin	a therapeutic method to treat stomach yin deficiency pattern/syndrome, the same as to tonify the stomach yin or to nourish the stomach	胃陰虚証の治療法。補胃陰および養胃と同義。
補胃陰	養胃陰; 補胃陰; 養胃	4.2.239	nourish the stomach yin	a therapeutic method to treat stomach yin deficiency pattern/syndrome, the same as to tonify the stomach yin or to nourish the stomach	胃陰虚証の治療法。養胃陰および養胃と同義。
養胃	養胃陰; 補胃陰; 養胃	4.2.239	nourish the stomach yin	a therapeutic method to treat stomach yin deficiency pattern/syndrome, the same as to tonify the stomach yin or to nourish the stomach	胃陰虚証の治療法。養胃陰および補胃陰と同義。
補腎益氣		4.2.240	tonify the kidney and replenish qi	a therapeutic method to treat kidney qi deficiency pattern/syndrome	腎気虚証の治療法。
滋腎益陰		4.2.241	enrich the kidney and replenish yin	a therapeutic method to treat kidney yin deficiency pattern/syndrome	腎陰虚証の治療法。
滋腎養肝		4.2.242	enrich the kidney and nourish the liver	a therapeutic method to treat kidney-liver yin deficiency pattern/syndrome	腎肝陰虚証の治療法。
溫腎健脾		4.2.243	warm the kidney and fortify the spleen	a therapeutic method to treat spleen-kidney yang deficiency by using warming and tonifying medicinals	温裏補陽薬を用いて脾腎陽虚を治療する方法。
調肝補腎		4.2.244	regulate the liver and supplement the kidney	a therapeutic method to treat liver-kidney yin deficiency with exuberant liver yang	肝陽上亢を伴う肝腎陰虚の治療法。
養血柔肝		4.2.245	nourish the blood and emolliate the liver	a therapeutic method to treat liver blood deficiency with exuberant wind yang by using blood-tonifying and liver-emolliating medicinals	補血および平肝熄風薬を用いて風化風を伴う肝血虚を治療する方法。
滋水涵木		4.2.246	enrich water to moisten wood	a therapeutic method to treat liver yin deficiency (pertaining to wood) by nourishing kidney yin (pertaining to water)	腎陰 (水に関係する)を補充することで 肝陰虚 (木に関係する)を治療する方法。
酸甘化陰		4.2.247	form yin with the sour and sweet	a therapeutic method of replenishing yin blood by combined use of sour-tasted medicinals and sweet-tasted ones	酸っぱい薬剤と甘い薬剤を併用して陰血を補充する治療法 。
辛甘化陽		4.2.248	form yang with the pungent and sweet	a therapeutic method of reinforcing yang qi by combined use of pungent-tasted medicinals and sweet-tasted ones	辛い薬剤と甘い薬剤を併用して、陽気を強化する治療法。
引火歸原	引火歸原; 引火下行	4.2.249	conduct fire back to its origin	a therapeutic principle for the ascending of asthenic fire, by adding drugs for tonifying the kidney yang to those for nourishing the kidney yin to lead the ascending deficiency fire back down to the kidney, the same as to conduct fire	虚火の上浮に対する治療原則。腎補陰薬に腎補陽薬を追加し、上浮した虚火を腎臓に帰させる。引火下行と同義。
引火下行	引火歸原; 引火下行	4.2.249	conduct fire back to its origin	a therapeutic principle for the ascending of asthenic fire, by adding drugs for tonifying the kidney yang to those for nourishing the kidney yin to lead the ascending deficiency fire back down to the kidney, the same as to conduct fire	虚火の上浮に対する治療原則。腎補陰薬に腎補陽薬を追加し、上浮した虚火を腎臓に帰させる。引火歸原と同義。
溫腎納氣		4.2.250	warm the kidney to promote qi absorption	a therapeutic method to treat kidney failing to receive qi from the lung	肺からの腎不納気の治療法。
固澁法		4.2.251	securing and astringing method	a general term for therapeutic methods of using styptic or astringent medicinals to treat spontaneous sweating, seminal emission, chronic diarrhea, or hemorrhage	止血または収斂薬を用いて、自汗、射精、慢性下痢、または出血を治療する方法の一般用語。
澁腸止瀉		4.2.252	astringe the intestines and check diarrhea	a therapeutic method to treat chronic diarrhea with astringents	収斂薬を用いた慢性下痢の治療法。
固精		4.2.253	secure essence	a therapeutic method to treat seminal emission due to kidney consumption by using kidney-tonifying and astringent medicinals	補腎収斂薬を用いて腎消耗による遺精・滑精を治療する方法。
固精縮尿		4.2.254	secure essence and reduce urination	a therapeutic method to treat seminal emission, spermatorrhea, enuresis or incontinence of urine due to kidney qi insecurity and bladder retention failure	腎気不固および膀胱失約による射精、精液漏、遺尿また尿失禁の治療法。
固崩止帶		4.2.255	stem flooding and check vaginal discharge	a therapeutic method to treat abnormal uterine bleeding or excessive leukorrhea by using astringent medicinals	収斂薬を用いて異常な子宮出血または過剰な 帯下を治療する方法。
固衝止帶		4.2.256	secure the thoroughfare vessel and stanch vaginal discharge	a therapeutic method to treat leukorrhagia due to kidney insufficiency with insecurity of the thoroughfare and conception vessels	衝任不固を伴う腎機能不全による大量の帯下を治療する方法。
固表		4.2.257	secure the exterior	a therapeutic method to treat exterior deficiency and insecurity	表虚および不固の治療法。
固表止汗	固表止汗; 斂汗固表	4.2.258	secure the exterior to check sweating	a therapeutic method to treat defensive qi deficiency with spontaneous sweating	自汗を伴う衛気虚の治療法。
斂汗固表	固表止汗; 斂汗固表	4.2.258	secure the exterior to check sweating	a therapeutic method to treat defensive qi deficiency with spontaneous sweating	自汗を伴う衛気虚の治療法。
安神		4.2.259	tranquilize	a general term for tranquilizing measures	静穏にさせる方法の一般用語。
養心安神		4.2.260	nourish the heart to tranquilize	a therapeutic method of using yin blood tonifying medicinals to treat palpitations, insomnia, dream-disturbed sleep and forgetfulness due to disquieted heart spirit	補陰血薬を用いて、不安な心神による心悸亢進、不眠症、夢に妨げられる睡眠および健忘を治療する方法。
鎭驚安神		4.2.261	settle fright and tranquilize	a therapeutic method of using settling tranquilizers to relieve mental uneasiness caused by fright	鎮静薬を用いて、驚きによる精神的不安を緩和する治療法。
重鎭安神	重鎭安神; 鎭靜安神	4.2.262	tranquilize by heavy settling	a therapeutic method to treat insomnia and mental excitement by using heavy mineral and shell medicinals	重鉱物および貝殻類を用いて 不眠および精神的興奮を治療する方法。
鎭靜安神	重鎭安神; 鎭靜安神	4.2.262	tranquilize by heavy settling	a therapeutic method to treat insomnia and mental excitement by using heavy mineral and shell medicinals	重鉱物および貝殻類を用いて 不眠および精神的興奮を治療する方法。

検索表示（原表示の複数語を分離）	IST原表示	Code	Term	Definition/Description	定義/記述
開竅	開竅;醒腦	4.2.263	open the orifices	any therapeutic method of inducing resuscitation	意識を回復させる全ての治療法。
醒腦	開竅;醒腦	4.2.263	open the orifices	any therapeutic method of inducing resuscitation	意識を回復させる全ての治療法。
清心開竅		4.2.264	clear the heart and open the orifices	a therapeutic method of using heat-clearing, fire-purging and orifice-opening medicinals to induce resuscitation in patients with heat blocked in the pericardium	清熱瀉火および開竅薬を用いて、熱陥心包の患者の意識を回復させる治療法。
清熱開竅		4.2.265	clear heat to open the orifices	a therapeutic method to treat impaired consciousness in cases of acute febrile diseases	急性熱性疾患の場合に、意識障害を治療する方法。
化痰開竅		4.2.266	resolve phlegm to open the orifices	a therapeutic method of using phlegm-resolving medicinals to treat phlegm syncope and phlegm confounding the orifices of the heart	化痰薬を用いて、痰厥および痰濁内蒙心包を治療する方法。
芳香開竅		4.2.267	open the orifices with aroma	emergency treatment for loss of consciousness by employing aromatic medicinals	芳香薬を用いた、意識喪失の緊急治療。
消食		4.2.268	promote digestion	a general term for the treatment of food stagnation	食物鬱滞の治療の一般用語。
消食導滯		4.2.269	promote digestion and remove food stagnation	a therapeutic method of using digestants and laxatives to treat dyspepsia caused by improper diet or overeating	消化薬および潤下薬を用いて、不適当な食事および過食による消化不良を治療する方法。
消癰散結		4.2.270	disperse abscesses and nodules	a therapeutic method of dissolving abscesses and nodules before suppuration	化膿する前に膿瘍および結節を解消する治療法。
消癰散癤		4.2.271	disperse abscesses and boils	a therapeutic method of dissolving abscesses and boils before suppuration	化膿する前に膿瘍および癤を解消する治療法。
消骨鯁		4.2.272	dissolve fish bones	a therapeutic method for removing a fish bone stuck in the throat or esophagus	喉や食道に刺さった魚の骨を除去する治療法。
退翳明目	退翳明目; 退目翳	4.2.273	remove nebula and improve vision	a therapeutic method to remove opacity and improve vision used in the treatment of corneal nebula	不明瞭さを取り除き、視覚を改善する治療法。角膜白濁の治療に用いられる。
退目翳	退翳明目; 退目翳	4.2.273	remove nebula and improve vision	a therapeutic method to remove opacity and improve vision used in the treatment of corneal nebula	不明瞭さを取り除き、視覚を改善する治療法。角膜白濁の治療に用いられる。
明目		4.2.274	improve vision	any therapeutic method that can ameliorate impairment of vision	視覚障害を改善する全ての治療法。
通陽		4.2.275	unblock yang	a therapeutic method used to address blockage of yang qi due to cold-damp obstruction or congealing phlegm and stasis obstruction	寒湿阻滞または痰凝および停滞閉塞による陽気不通の解消に用いられる治療法。
通陽散結		4.2.276	unblock yang to dissipate binds	a therapeutic method for dissipating masses caused by yang deficiency and cold congealing	陽虚および寒凝による腫瘤を消散させる治療法 。
塞流	塞流,澄源,復舊	4.2.277	arrest of bleeding, clarification of cause and recovery of health	three therapeutic methods for treating flooding and spotting in gynecology	婦人科学における大量出血および少量出血の3つの治療法。
澄源	塞流,澄源,復舊	4.2.277	arrest of bleeding, clarification of cause and recovery of health	three therapeutic methods for treating flooding and spotting in gynecology	婦人科学における大量出血および少量出血の3つの治療法。
復舊	塞流,澄源,復舊	4.2.277	arrest of bleeding, clarification of cause and recovery of health	three therapeutic methods for treating flooding and spotting in gynecology	婦人科学における大量出血および少量出血の3つの治療法。
化積		4.2.278	resolve accumulation	a therapeutic method to treat food accumulation pattern/syndrome by using digestant and evacuant medicinals	消化薬および瀉下薬を用いて食積証を治療する方法。
安胎		4.2.279	prevent abortion	preventive and therapeutic measure for threatened miscarriage and habitual abortion	切迫流産および習慣流産の予防および治療法
下乳		4.2.280	promote lactation	a therapeutic method to promote the flow of breast milk after delivery	出産後の母乳の流れを促進する治療法。
斷乳	斷乳;回乳	4.2.281	terminate lactation	a therapeutic method to stop the flow of milk	母乳の流れを止める治療法。
回乳	斷乳;回乳	4.2.281	terminate lactation	a therapeutic method to stop the flow of milk	母乳の流れを止める治療法。
驅蟲		4.2.282	expel worms	a therapeutic method of expelling intestinal parasites	腸内寄生虫を排除する治療法 。
殺蟲		4.2.283	kill worms	a therapeutic method of destroying intestinal parasites	腸内寄生虫を駆除する治療法。
安蛔		4.2.284	quiet ascaris	a therapeutic method to treat ascariasis, usually for relieving abdominal pain or biliary colic caused by ascaris	回虫症の治療法。通常、回虫による腹痛や胆道仙痛を緩和する。
安蛔定痛		4.2.285	quiet ascaris to relieve pain	a therapeutic method to treat abdominal pain due to intestinal or biliary ascariasis	腸回虫症や胆道回虫症による腹痛の治療法。
排膿		4.2.286	expel pus	a therapeutic method of promoting pus discharge	排膿を促進する治療法。
祛瘀生新		4.2.287	dispel stasis to promote regeneration	a therapeutic method of activating blood and removing stasis to promote blood regeneration for treating blood stasis complicated with blood deficiency	血液再生を促進することで血を活性化させ、鬱滞を除去する治療法で、血虚を併発した血瘀の治療に用いる。
生肌收口		4.2.288	promote tissue regeneration and close wound	a therapeutic method of treating wounds to promote the growth of new tissue by using detoxicants and astringents	解毒薬および収斂薬を用いて、新しい組織の増殖を促進することで創傷を治療する方法。
引血下行		4.2.289	conduct blood downward	a therapeutic method of treating upsurge of blood flow	血流の急増の治療法。
軟堅散結		4.2.290	soften hardness and dissipate binds	a therapeutic method of using qi-moving, blood-activating and phlegm-eliminating medicinals to treat lumps formed by turbid phlegm or static blood	行気、活血、去痰薬を用いて、痰濁または鬱血により形成された塊を治療する方法。
辟穢		4.2.291	repel foulness	a therapeutic method of using aromatic medicinals to treat diseases caused by pathogenic foul turbidity	芳香薬を用いて、穢濁による疾患を治療する方法。
緩急		4.2.292	relax tension	a therapeutic method of relieving spasm, contraction or hypertonicity	痙攣、萎縮または過緊張を緩和する治療法。

検索表示（原表示の複数語を分離）	IST原表示	Code	Term	Definition/Description	定義/記述
托毒		4.2.293	expel toxin	a therapeutic method of expelling toxins from within in the treatment of boils and sores	癤およびただれの治療において、毒素を排除する治療法。
托瘡		4.2.294	expel pus of sores	a therapeutic method of expelling pus in the treatment of boils, sores and abscess	癤、ただれおよび膿瘍の治療において、膿を排除する治療法。
内托		4.2.295	expel from within	a therapeutic method of using tonifying and pus-discharging medicinals to support healthy qi and promote outflow of pus, in order to prevent inward penetration of the pathogenic toxin	疫毒の内部への侵入を予防するため、補薬および排膿薬を用いて、正気を維持させ、膿の流出を促進する治療法。
攝唾		4.2.296	constrain spittle	a therapeutic method of checking excessive salivation	過剰な唾液の分泌を阻止する治療法
截瘧		4.2.297	interrupt malaria	method of treating malaria applied before an episode to prevent its arrival	マラリアの治療法で、その到達を予防するために、エピソード発現前に適用される。
解毒殺蟲		4.2.298	detoxify and kill worms	a therapeutic method to remove toxicity and kill parasites such as mites in the treatment of scabies	疥癬の治療において、毒性を除去し、ダニなどの寄生虫を駆除する治療法。
止癢		4.2.299	relieve itching	any therapeutic measure that has an antipruritic effect	止痒性の効果を有する全ての治療法。
燥濕止癢		4.2.300	dry dampness to relieve itching	a therapeutic method for removing exudate and alleviating itching, as used in the treatment of eczema	滲出物を除去し、かゆみを軽減する治療法。湿疹の治療に使われる。
消腫		4.2.301	disperse swelling	a therapeutic method that induces detumescence or promotes subsidence of swelling	腫脹消退を起こさせるかまたは腫脹の鎮静化を促進する治療法。
拔毒		4.2.302	draw out toxin	a therapeutic method to remove toxins from inflammatory lesions	炎症性病変から毒素を除去する治療法。
提膿拔毒		4.2.303	draw out pus and toxin	a therapeutic method to promote discharge of pus and remove toxins in the treatment of purulent toxin pattern/syndrome	膿毒証の治療において、膿の排出を促進し、毒素を除去する治療法。
提膿祛腐		4.2.304	draw out pus and dispel putridity	a therapeutic method to promote pus discharge and remove putridity in the treatment of embedded sores	皮膚内部にあるただれの治療において、膿の排出を促進し、腐敗物を除去する治療法。
化腐	化腐; 去腐肉	4.2.305	resolve putridity	a therapeutic method of using corrosive medicinals for treating external diseases, especially wounds and sores	腐食薬を用いて、外部の疾患、特に創傷およびただれを治療する方法。
去腐肉	化腐; 去腐肉	4.2.305	resolve putridity	a therapeutic method of using corrosive medicinals for treating external diseases, especially wounds and sores	腐食薬を用いて、外部の疾患、特に創傷およびただれを治療する方法。
蝕瘡去腐		4.2.306	heal wounds and eliminate putridity	a therapeutic method of eliminating putrid material in the treatment of wounds and sores	創傷およびただれの治療において、腐敗物を除去する治療法。
生肌斂瘡		4.2.307	promote tissue regeneration and wound healing	a therapeutic method to promote healing of wounds or ulcers on the body surface	体表の創傷または潰瘍の治癒を促進する治療法。
煨膿長肉		4.2.308	promote suppuration to regenerate flesh	a therapeutic method of using pus-discharging medicinals or other therapies to promote the growth of granulation tissue and healing of the wound	排膿薬やその他の治療を用いて、肉芽組織の増殖および創傷治癒を促進する治療法。
透邪		4.2.309	outthrust the pathogen	a therapeutic method of driving pathogens out of the body, usually used in the treatment of exterior patterns/syndromes	病邪を体外に出す治療法。通常、表証の治療に用いられる。
潰堅		4.2.310	promote rupture	a therapeutic method used to treat abscess by promoting its rupture	破裂を促進することで膿瘍を治療する方法。
烏鬚髮		4.2.311	blacken the beard and hair	a therapeutic method for treating premature graying of the hair and beard	毛髪および髭の若白髪を治療する方法。
膏藥療法	膏藥療法; 藥膏療法; 薄貼療法	4.2.312	plaster therapy	a therapeutic method of applying plasters to the diseased site or relevant points to treat local or generalized diseases	疾患部位または関連部位に膏薬を適用し、局所または全身疾患を治療する方法。
藥膏療法	膏藥療法; 藥膏療法; 薄貼療法	4.2.312	plaster therapy	a therapeutic method of applying plasters to the diseased site or relevant points to treat local or generalized diseases	疾患部位または関連部位に膏薬を適用し、局所または全身疾患を治療する方法。
薄貼療法	膏藥療法; 藥膏療法; 薄貼療法	4.2.312	plaster therapy	a therapeutic method of applying plasters to the diseased site or relevant points to treat local or generalized diseases	疾患部位または関連部位に膏薬を適用し、局所または全身疾患を治療する方法。
藥酒療法		4.2.313	medicated wine therapy	a therapeutic method of treatment by taking medicated wine or liquor, a preparation made by steeping medicinals in grain wine or liquor	薬剤を穀物発酵酒や蒸留酒に浸して作製した薬用発酵酒や薬用蒸留酒を摂取することで治療する方法。
熏法		4.2.314	fumigation	a therapeutic method by utilizing both the medicinal and heat effects to open the interstices and promote the flow of qi and blood for reducing swelling, alleviating pain, dispelling wind and relieving itching	薬剤と熱の作用の両方を利用する治療法で、腠理を開き 、気と血の流れを促進して腫脹を低減し、疼痛を軽減し、風邪を消散させ、かゆみを緩和する。
熨法		4.2.315	hot medicinal compress	a therapeutic measure involving pressing and rubbing the diseased area with hot medical substances wrapped in cloth	布に包んだ熱い薬剤で、疾患部位を押し、こする治療法。
烙法		4.2.316	cauterization	application of a searing iron to destroy diseased tissue	焼きごてを使用して、罹患組織を破壊すること。
坐藥療法		4.2.317	suppository therapy	a therapeutic method involving the introduction of a specially shaped solid medicinal preparation into the rectal, urethral or vaginal orifice to treat diseases, generally diseases of the anus, rectum, lower colon, urethra, uterus and vagina	直腸、尿道、または膣の開口部に、特殊な形状をした固形薬剤を挿入し、疾患（通常、肛門、直腸、下部結腸、尿道、子宮および膣の疾患）を治療する方法。
藥撚療法		4.2.318	medicated thread therapy	an external medical technique whereby a twisted paper coated with or enveloping medicinal powder is introduced into the diseased site for treating abscess, sores, flowing phlegm and cancers	薬用の粉末を被覆したまたは包んだ、ねじった紙を罹患部位に挿入し、膿瘍、ただれ、流痰および癌を治療する外用療法。
捏脊		4.2.319	spine pinching	a therapeutic method by pinching and kneading the skin and muscles bilateral to the spine	背骨の両側の皮膚と筋肉を、同時につまんでもむ治療法。
結紮療法		4.2.320	ligation therapy	an external medical technique whereby hemorrhoids, polyps or warts are treated by binding them at the base with threads so that the distal portion sloughs away within several days	痔核、ポリープ、またはいぼを、その基部をひもで結び、末端部位を数日間で脱落させる外用療法。

検索表示（原表示の複数語を分離）	IST原表示	Code	Term	Definition/Description	定義/記述
熏洗療法		4.2.321	fuming-washing therapy	a therapeutic method involving fuming the diseased area with the vapor of a boiling decoction and then washing the area with the decoction	罹患部位を、沸騰させた煎剤の蒸気で燻り、その部位を煎剤で洗浄する治療法。
沖洗法	沖洗法;沖洗療法	4.2.322	douche	an external therapeutic method by directing a stream of a medicinal solution against the diseased site	罹患部位に薬剤液の蒸気をあてる外用の治療法 。
沖洗療法	沖洗法;沖洗療法	4.2.322	douche	an external therapeutic method by directing a stream of a medicinal solution against the diseased site	罹患部位に薬剤液の蒸気をあてる外用の治療法 。
吹藥法		4.2.323	insufflations	blowing powdered medicine into the throat or inner part of the mouth for therapeutic purposes	喉や口の内部に、治療目的で散剤を吹き付けること。
牽引療法		4.2.324	traction therapy	treatment of skeletal or joint disorders by applying a pulling force	骨格または関節の疾患を、牽引力で治療すること。
理筋手法		4.2.325	therapeutic manipulation for sinew injury	a collective term for various manipulations for restoring and treating injured soft tissues	負傷した軟部組織を回復させ治療するさまざまな手技の総称。
按摩	按摩;推拿;按蹻	4.2.326	massage	rubbing, kneading, or percussion of the soft tissues and joints of the body with the hands, usually performed by one person on another, esp. to relieve tension or pain	体の軟部組織と関節を、手でこすったり、もんだり、たたいたりすること。通常、1人が別の人に対して、特に緊張や疼痛を緩和するため行う。
推拿	按摩;推拿;按蹻	4.2.326	massage	rubbing, kneading, or percussion of the soft tissues and joints of the body with the hands, usually performed by one person on another, esp. to relieve tension or pain	体の軟部組織と関節を、手でこすったり、もんだり、たたいたりすること。特に緊張や疼痛を緩和するために、通常、1人が別の人に対して行う。
按蹻	按摩;推拿;按蹻	4.2.326	massage	rubbing, kneading, or percussion of the soft tissues and joints of the body with the hands, usually performed by one person on another, esp. to relieve tension or pain	体の軟部組織と関節を、手でこすったり、もんだり、たたいたりすること。特に緊張や疼痛を緩和するために、通常、1人が別の人に対して行う。
旋轉法		4.2.327	rotating manipulation	a manipulation performed by holding the distal end of the injured limb and rotating it gently	負傷した手足の遠位部を持ち、穏やかに回転させる手技。
搓法		4.2.328	twisting manipulation	a manipulation of giving an injured limb twists with two palms in the direction opposite each other	負傷した手足を両手掌でお互い逆の方向にねじる手技。
摩法		4.2.329	rubbing manipulation	rubbing the affected part with the ventral aspect of the finger tips or the palm in a circular motion with moderate force and frequency	罹患部位を、指先の腹または手掌で、適度な力で頻繁に、円形にこすること。
揉法		4.2.330	kneading manipulation	a manipulation involving pressing and moving to and fro or circularly on an affected area with the flat of the thumb, the thenar or the root of the palm	罹患部位を、親指の平坦部、母指球、または手掌の根本で、前後に、または円形に、圧迫して動かす手技。
推法		4.2.331	pushing manipulation	pushing and squeezing the muscles with the fingers or palms forward, apart or spirally, with force	指または手掌で、力を入れて、前方、別々またはらせん状に、筋肉を押して圧迫すること。
一指禪推法		4.2.332	qi-concentrated single-finger pushing manipulation	a pushing manipulation performed by using the thumb alone in a swaying movement	親指のみを用いて、揺れる動きで押す手技。
滾法		4.2.333	rolling manipulation	a manipulation performed by turning the back of the hand over the patient’s body surface back and forth continuously	患者の体表面で、前後に連続的に手背を捻転させる手技。
擦法		4.2.334	scrubbing manipulation	a manipulation performed by rubbing with the flat of the finger, the thenar, or the palm to and fro over the skin continuously with a high frequency	皮膚を、指の平坦部、母指球または手掌で、高頻度に連続的に前後にこする手技。
抖法		4.2.335	shaking manipulation	a manipulation by which the distal end of the affected extremity is held and pulled outwards by the operator and shaken up and down within the limit of movement	施術者が、罹患した手足の遠位末端を持ち、外側に引っ張り、動かせる範囲内で上下に振る手技。
彈法		4.2.336	flicking manipulation	a manipulation performed by hitting the affected area with the back of the index fingertip or middle fingertip by flicking against the thumb	人差し指または中指の先の背で、親指に対してはじいて、罹患部位をたたく手技。
彈筋法		4.2.337	sinew-flicking manipulation	a manipulation performed by repeatedly pulling up the tendon or muscle and immediately releasing it	腱または筋肉を繰り返し引っ張り上げ、すぐに放す手技。
按法		4.2.338	pressing manipulation	a manipulation involving pushing steadily in a direction vertical to the body surface	体表に垂直な方向に、絶え間なく押す手技。
拿法		4.2.339	grasping manipulation	a manipulation performed by lifting and squeezing the affected muscles with the thumb and the index and middle fingers or with the thumb and the other four fingers of one or both of the operator’s hands	施術者の片手または両手の親指と人差し指と中指、または親指と残りの4本の指で、罹患した筋肉を持ち上げて圧迫する手技。
捏法		4.2.340	pinching manipulation	a manipulation performed by holding and lifting the soft tissues by using the thumb with the index and middle fingers or the thumb with the other four fingers, and squeezing and pushing forward	親指と人差し指と中指、または親指と残りの4本の指で軟部組織を持ち上げ、圧迫して前に押す手技。
踩蹺法		4.2.341	treading manipulation	a manipulation performed by treading on the affected area for reduction, e.g., treading on the back for the reduction of a protruded intervertebral disc	罹患部位を踏み、還納させる手技。例、突き出した椎間板を還納するために背中を踏む。
扳法		4.2.342	pulling manipulation	a manipulation of extending or rotating a joint by holding its proximal and distal ends and pulling with force in an opposite or similar direction instantaneously	関節の近位末端および遠位末端を持ち、力を入れて逆方向または同じ方向に瞬間的に引っ張り、関節をのばすまたは回す手技。
搖法		4.2.343	rocking manipulation	a manipulation performed by holding the part of the limb proximal to the joint with one hand and the part distal to the joint with the other hand, and gently rotating the joint from side to side	関節から近位の足の一部を片方の手で持ち、関節から遠位の一部をもう片方の手で持ち、関節を左右に穏やかに回す手技。
背法		4.2.344	back-packing manipulation	a manipulation to reduce a protruded lumbar intervertebral disc by carrying the patient on the operator’s back while the two are standing back to back	背中合わせで立ち、患者を施術者の背中に乗せて、突き出した椎間板を還納する手技。
揉撚法		4.2.345	massage technique before/after acupuncture	a manipulation in which the practitioner using his (her) thenar eminence, palmar root or the ventral aspect of fingers rubs and presses a certain area or point of the patient’s body with gentle and circular movements before/after needling	施術者が母指球の隆起、手掌の根もと、または指の腹側を使って、穿刺の前後に、穏やかな円形の動きで患者の体の特定の部位または点をこするまたは圧迫する手技。
叩打法		4.2.346	tapping technique	a manipulation performed by tapping with the tips of fingers held together	そろえた指の先端で軽くたたくことにより行う手技。

検索表示（原表示の複数語を分離）	IST原表示	Code	Term	Definition/Description	定義/記述
指壓		4.2.347	finger pressure	a manipulation performed by pressing acupuncture points with the finger or thumb instead of needling, also known as shiatsu	鍼を打つのではなく、経穴を指や親指で押すことにより行う手技。指圧としても知られる。
**鍼灸（學）**		**5.0.0**	**ACUPUNCTURE AND MOXIBUSTION**		
**鍼;鍼法**		**5.1.0**	**acupuncture**	the insertion of needles into humans or animals for remedial purposes or its methods	治療を目的として鍼を人または動物に刺入すること、またはその方法。
鍼灸;<br>鍼灸學		5.1.1	acupuncture and moxibustion	(1)a collective term for acupuncture and moxibustion;(2)a branch of traditional Chinese medicine which mainly involves the theory of meridians, location, usage, indications and combinations of acupoints, needling manipulations and application of ignited moxa in the treatment of disease through regulation of qi, blood and visceral functions	(1)鍼療法および灸療法の総称(2)気、血、および内臓機能の調節による疾治療における、経絡の理論、位置、用途、適用、経穴の組み合わせ、鍼療法および点火したもぐさの適用を主とした、伝統的な中医学の一部門。
刺灸法		5.1.2	needling and moxibustion method	a collective term for the techniques of acupuncture and moxibustion	鍼療法および灸療法の総称。
火鍼		5.1.3	fire needling	an acupuncture procedure involving the swift pricking of the diseased part with a red hot needle	赤く焼いた鍼で罹患部位を速やかに刺す鍼療法。
溫鍼	溫鍼;<br>溫鍼灸	5.1.4	warm needling	(1) a practice performed by placing an ignited moxa stick on the handle of the needle after insertion; (2) a method of acupuncture in which the needle is warmed before/during needling, the same as warm needling moxibustion	(1) 鍼刺入後に、点火した艾條を鍼柄に当てる手技。(2) 鍼刺入前または鍼刺入中に鍼を温める鍼療法。溫鍼灸と同義 。
溫鍼灸	溫鍼;<br>溫鍼灸	5.1.4	warm needling	(1) a practice performed by placing an ignited moxa stick on the handle of the needle after insertion; (2) a method of acupuncture in which the needle is warmed before/during needling, the same as warm needling moxibustion	(1) 鍼刺入後に、点火した艾條を鍼柄に当てる手技。(2) 鍼刺入前または鍼刺入中に鍼を温める鍼療法。溫鍼と同義 。
九鍼		5.1.5	nine classical needles	a collective term for the nine types of needles described in Lingshu, namely, filiform needle, shear needle, round-pointed needle, spoon needle, lance needle, round-sharp needle, stiletto needle, long needle and big needle	「靈樞」に記載されている9種類の鍼の総称。毫鍼、鑱鍼、圓鍼、鍉鍼、鋒鍼、員利鍼、鈹鍼、長鍼および大鍼。
毫鍼		5.1.6	filiform needle	a type of fine needle of varying length most commonly used in performing acupuncture at present	さまざまな長さの細い鍼の一種。現在鍼療法を施行する際に最もよく使われている。
鑱鍼		5.1.7	shear needle	a pricking instrument like an arrowhead with a sharp tip, the same as a chisel needle or sagital needle	鍼尖がとがった、矢じりに似た刺すための器具。chisel needleやsagital needleと同義。
圓鍼		5.1.8	round-pointed needle	a cylindrical instrument with the shaft 1.6 cun long and an oval tip, used for massage on the body surface, the same as a rounded needle	1.6寸の長さの鍼体を持ち、鍼尖が卵状になった、筒状の器具。体表のマッサージに利用される。rounded needleと同義。
鍉鍼		5.1.9	spoon needle	an instrument with a thick shaft and a slightly sharp round tip used for pressing the meridians/channels	太い鍼体と、少し鋭い丸い鍼尖を持つ器具。経絡を圧迫するために用いられる。
鋒鍼		5.1.10	lance needle	another name for three-edged needle, the same as a lancing needle	三稜鍼の別名。lancing needle と同義 。
員利鍼		5.1.11	round-sharp needle	a needle with thin shaft and a somewhat enlarged and sharp round tip, the same as a sharp round needle	鍼体が細く、幾分太くて鋭い丸い鍼尖を持つ鍼。sharp round needleと同義。
鈹鍼		5.1.12	stiletto needle	an instrument like a double-edged sword used for drainage of pus, the same as a sword needle	両刃の剣に似た器具。排膿に用いられる。sword needleと同義。
長鍼		5.1.13	long needle	one of the nine classical needles, 7 cun in length, used for deep puncture	九鍼の1つ。長さ7寸で、深い穿刺に用いられる。
大鍼		5.1.14	big needle	a needle with a long and thick shaft and a somewhat round tip, the same as a large needle	長くて太い鍼体と、幾分丸い鍼尖を持つ鍼。large needleと同義。
巨鍼		5.1.15	great needle	that resembling a filiform needle, but thicker and longer, used in the treatment of hemiplegia and paralysis	毫鍼に似ているが、太くて長く、片麻痺および麻痺の治療に用いられる。
砭石		5.1.16	stone needle	needle made of stone used as a primitive instrument for acupuncture and surgical incision in ancient times	古代、鍼療法および手術の切開で原始的な器具として使われていた、石でできた鍼。
砭刺療法		5.1.17	stone needle therapy	treatment of disease with stone needles in ancient times	砭石を用いた古代の疾患治療。
毫鍼療法		5.1.18	filiform needle therapy	acupuncture therapy which uses filiform needles	毫鍼を用いた鍼療法。
鍼尖		5.1.19	tip of the needle	the sharp point of the needle	鍼の鋭い先端のこと。
鍼柄		5.1.20	handle of the needle	the part of the needle, distal to the tip and shaft, by which it is held	鍼の一部で、鍼尖および鍼体より末端部の、手で持つ部分。
鍼體		5.1.21	shaft of the needle	the part of the needle between the handle and tip	鍼の一部で、鍼柄と鍼尖の間の部分。
鍼根		5.1.22	root of the needle	junction between the handle and shaft of the needle	鍼柄と鍼体との境目。
梅花鍼		5.1.23	plum-blossom needle	a dermal needling instrument with a bundle of five short embedded needles resembling a plum blossom	5本の短い植え込まれた鍼の束がついた皮膚用の鍼器具 。梅の花に似ている。
梅花鍼療法		5.1.24	plum-blossom needle therapy	therapy by tapping with a plum-blossom needle	梅花鍼で軽く叩く治療法。
七星鍼		5.1.25	seven-star needle	a dermal needling instrument with seven short needles attached to the end of a handle in a cluster	鍼柄の末端に7本の短い鍼がまとめて取り付けられている皮膚用の鍼器具。
三棱鍼		5.1.26	three-edged needle	a thick needle with a sharp three-edged tip	鋭い3本の刃のある鍼尖を持つ太い鍼。
三棱鍼療法		5.1.27	three-edged needle therapy	a variety of therapeutic method to cure illness by using a three-edged needle	疾患を治癒させるための三稜鍼を用いたさまざまな治療法
揿鍼		5.1.28	thumbtack needle	an intradermal needle resembling a thumbtack	画鋲に似た皮内鍼。
皮內鍼		5.1.29	intradermal needle	a small needling instrument for embedding in the skin	皮膚に固定する小さな鍼器具
皮內鍼療法		5.1.30	intradermal needle therapy	a therapeutic method by embedding a needle at a certain point for extended periods	特定の経穴に長期間鍼を固定する治療法。
皮膚鍼		5.1.31	dermal needle	a needling instrument composed of several short needles used for tapping the points	複数の短い鍼で構成される鍼器具。経穴を軽く叩くために用いられる。
皮膚鍼療法		5.1.32	dermal needle therapy	a therapeutic method whereby points are tapped with a dermal needle	経穴を皮膚鍼で軽く叩く治療法。

検索表示（原表示の複数語を分離）	IST原表示	Code	Term	Definition/Description	定義/記述
滾刺筒		5.1.33	roller needle	a metallic roller designed for dermal needling	皮膚鍼用にデザインされた金属製のローラー。
銀鍼		5.1.34	silver needle	a needle made of silver	銀製の鍼。
不銹鋼鍼		5.1.35	stainless steel needle	a needle made of stainless steel	ステンレス鋼製の鍼。
一回用鍼	一回用鍼; 一次性鍼	5.1.36	disposable needle	a single use needle which is disposed after of use	使用後廃棄する、使い捨ての鍼。
一次性鍼	一回用鍼; 一次性鍼	5.1.36	disposable needle	a single use needle which is disposed after of use	使用後廃棄する、使い捨ての鍼。
小兒鍼		5.1.37	infant needle	acupuncture needle used in infant patients	小児患者の鍼療法用の鍼。
小兒鍼法		5.1.38	infant acupuncture therapy	acupuncture therapy in infant patients	小児患者用の鍼療法
舍巖鍼法		5.1.39	Sa-am acupuncture therapy	a school of acupuncture initiated by Sa-am,characterized by applying the five phases theory and mother-child reinforcement-reduction principle to the selection of points and needling manipulation	舍巖が創始した鍼療法の流派。五行学説および母子相生・相克原則を、経穴の選択と鍼処置に適用することを特徴とする。
八體質鍼		5.1.40	eight constitutions acupuncture	a school of acupuncture developed by Kwon Do-won based on the theory of eight sub-constitutions	Kwon Do-wonが創始した鍼療法の流派。八體質の理論に基づく。
微鍼系統		5.1.41	microsystem acupuncture	a general term for various types of acupuncture in a specific local area, including scalp acupuncture, ear acupuncture, nose acupuncture, hand acupuncture, foot acupuncture, etc.	特定の部位におけるさまざまなタイプの鍼療法の総称。頭鍼療法、耳鍼療法、鼻鍼療法、手鍼療法、足鍼療法など。
頭鍼	頭鍼; 頭皮鍼	5.1.42	scalp acupuncture	acupuncture at the specific lines located on the scalp	頭皮に位置する特定のラインにおける鍼療法。
頭皮鍼	頭鍼; 頭皮鍼	5.1.42	scalp acupuncture	acupuncture at the specific lines located on the scalp	頭皮に位置する特定のラインにおける鍼療法。
頭鍼療法		5.1.43	scalp acupuncture therapy	one of the microsystem acupuncture therapies, in which specific lines located on the head are needled for therapeutic purpose	微鍼系統の1つ。頭に位置する特定のラインに、治療のために鍼刺入する。
面鍼		5.1.44	facial acupuncture	acupuncture at the specific points located on the face	顔に位置する特定穴における鍼療法。
耳鍼		5.1.45	ear acupuncture	acupuncture at the points located on the auricle, also called auriculo-acupuncture	耳介に位置する経穴における鍼療法。auriculo-acupunctureとも呼ばれる。
耳鍼療法		5.1.46	ear acupuncture therapy	one of the microsystem acupuncture therapies, in which points located on the auricle are needled for therapeutic purpose	微鍼系統の1つ。耳介に位置する経穴に、治療のために鍼刺入する。
鼻鍼		5.1.47	nose acupuncture	acupuncture at the specific points located on the nose	鼻に位置する特定穴における鍼療法。
鼻鍼療法		5.1.48	nose acupuncture therapy	one of the microsystem acupuncture therapies, in which specific points located on the nose are needled for therapeutic purpose	微鍼系統の1つ。鼻に位置する特定穴に、治療のために鍼刺入する。
手鍼		5.1.49	hand acupuncture	acupuncture at the specific points located on the hand	手に位置する特定穴における鍼療法。
手鍼療法		5.1.50	hand acupuncture therapy	one of the microsystem acupuncture therapies, in which specific points located on the hand are needled for therapeutic purpose	微鍼系統の1つ。手に位置する特定穴に、治療のために鍼刺入する。
手指鍼術	手指鍼術; 高麗手鍼療法	5.1.51	hand-finger acupuncture technique	a microsystem acupuncture characterized by needling at the specific points located on the hand and fingers, also called Koryo hand acupuncture therapy	微鍼系統の1つ。手と指に位置する特定穴に対する鍼刺入が特徴。Koryo hand acupuncture therapyとも呼ばれる。
高麗手鍼療法	手指鍼術; 高麗手鍼療法	5.1.51	hand-finger acupuncture technique	a microsystem acupuncture characterized by needling at the specific points located on the hand and fingers, also called Koryo hand acupuncture therapy	微鍼系統の1つ。手と指に位置する特定穴に対する鍼刺入が特徴。Koryo hand acupuncture therapyとも呼ばれる。
足鍼療法		5.1.52	foot acupuncture therapy	a microsystem acupuncture characterized by needling at the specific points located on the feet	微鍼系統の1つ。足に位置する特定穴に対する鍼刺入が特徴。
穴	穴; 腧穴; 穴位	5.1.53	acupuncture point	the point where a needle is inserted and manipulated in acupuncture therapy	鍼療法において鍼を刺入および操作する部位。
腧穴	穴; 腧穴; 穴位	5.1.53	acupuncture point	the point where a needle is inserted and manipulated in acupuncture therapy	鍼療法において鍼を刺入および操作する部位。
穴位	穴; 腧穴; 穴位	5.1.53	acupuncture point	the point where a needle is inserted and manipulated in acupuncture therapy	鍼療法において鍼を刺入および操作する部位。
經穴		5.1.54	meridian point	acupuncture points of a main meridian or the governor or conception vessel	正経、督脈または任脈にある、鍼治療を行う部位。
經外奇穴	經外奇穴; 奇穴	5.1.55	extra point	acupuncture points not located on the meridians, also known as non-meridian point	経絡上にない経穴。non-meridian point としても知られる。
奇穴	經外奇穴; 奇穴	5.1.55	extra point	acupuncture points not located on the meridians, also known as non-meridian point	経絡上にない経穴。non-meridian point としても知られる。
特定穴		5.1.56	specific point	points on the fourteen meridians with specific therapeutic effects	十四経絡に属する、特定の治療効果を有する経穴。
五輸穴		5.1.57	five transport points	five specific points of the twelve meridians located distal to the elbows and knees, namely the well point, brook point, stream point, river point and sea point	肘および膝よりも遠位に位置する十二経絡の5つの特定穴。すなわち、井穴、栄穴、兪穴、経穴および合穴
井穴		5.1.58	well point	one of the five transport points located at the distal end of the fingers or toes, where the meridian/channel qi starts to bubble	五輸穴の1つ。手指または足指の遠位末端に位置する。経気が発生ところである。

検索表示（原表示の複数語を分離）	IST原表示	Code	Term	Definition/Description	定義/記述
滎穴		5.1.59	brook point	one of the five transport points, mostly located distal to the metacarpophalangeal or metatarsophalangeal joints, where the meridian/channel qi starts to spout	五輸穴の1つ。ほとんどが、中手指節関節または中足指節関節よりも遠位に位置する。経気が溜まるところである。
輸穴		5.1.60	stream point	one of the five transport points, mostly located proximal to the metacarpophalangeal or metatarsophalangeal joints, where the meridian/channel qi starts to flourish	五輸穴の1つ。ほとんどが、中手指節関節または中足指節関節よりも近位に位置する。経気が注ぐところである。
經穴		5.1.61	river point	one of the five transport points, located on the lower limbs and forearms	五輸穴の1つ。下肢および前腕に位置する。
合穴		5.1.62	sea point	one of the five transport points, mostly located near the elbows or knees, where the meridian/channel qi goes deep into the body just as a river flows into the sea	五輸穴の1つ。ほとんどが肘または膝の近傍に位置する。川が海に注ぐがごとく、経気が体の深部に入るところである。
原穴		5.1.63	source point	points where the original qi of the visceral organs pours, passes or stays	内臓の元気が流出し、通過し、留まる経穴。
八會穴		5.1.64	eight meeting points	eight important points where the qi of viscus, bowel, qi, blood, tendon, vessel, bone and marrow gathers respectively	臓・腑・気・血・筋・脈・骨・髄の気がそれぞれ集まる、8つの重要な経穴。
交會穴		5.1.65	crossing point	point where two or more meridians/channels intersect	2つ以上の経絡が交わる経穴。
八脈交會穴		5.1.66	confluence points of the eight vessels	points where the main meridians communicate with the eight extra meridians	正経が奇経八脈とお互いに通じ合っている経穴。
六腑下合穴	六腑下合穴; 下合穴	5.1.67	lower sea points of the six bowels	specific points on the three yang meridians of the foot corresponding to the six bowels	足三陽経上の特定穴。六腑と合する。
下合穴	六腑下合穴; 下合穴	5.1.67	lower sea points of the six bowels	specific points on the three yang meridians of the foot corresponding to the six bowels	足三陽経上の特定穴。六腑と合する。
阿是穴	阿是穴; 天應穴	5.1.68	ouch point	an acupuncture point with no specific name nor definite location, the site of which is determined by tenderness or other pathological responses, also known as the ashi point	特定の名前や明確な位置が決められていない経穴。その位置は痛みを感じたり、その他の異常な反応が起きることにより決められる。ashi pointとしても知られる。
天應穴	阿是穴; 天應穴	5.1.68	ouch point	an acupuncture point with no specific name nor definite location, the site of which is determined by tenderness or other pathological responses, also known as the ashi point	特定の名前や明確な位置が決められていない経穴。その位置は痛みを感じたり、その他の異常な反応が起きることにより決められる。ashi pointとしても知られる。
郄穴	郄穴; 郤穴	5.1.69	cleft point	point where the meridian/channel qi accumulates deeply	経気が深く集まる経穴。
郤穴	郄穴; 郤穴	5.1.69	cleft point	point where the meridian/channel qi accumulates deeply	経気が深く集まる経穴。
絡穴		5.1.70	connecting point	point at the site where the meridian/channel sends out its collateral vessel	経脈が絡脈と通じているところの経穴。
募穴	募穴; 腹募穴	5.1.71	alarm point	specific points on the chest or abdomen where the qi of the respective viscus is concentrated	それぞれの内臓の気が集まる、胸部または腹部の特定穴。
腹募穴	募穴; 腹募穴	5.1.71	alarm point	specific points on the chest or abdomen where the qi of the respective viscus is concentrated	それぞれの内臓の気が集まる、胸部または腹部の特定穴。
俞穴	俞穴; 背俞穴	5.1.72	transport point	specific points on the back where the qi of the visceral organs is infused	内臓の気が注入される、背中の特定穴。
背俞穴	俞穴; 背俞穴	5.1.72	transport point	specific points on the back where the qi of the visceral organs is infused	内臓の気が注入される、背中の特定穴。
耳穴		5.1.73	ear point	points on the auricle of the ear	耳介に位置する経穴。
五行穴		5.1.74	five phase points	a series of five acupuncture points below the elbow or knee on each of twelve meridians, associated with the five phases theory	肘または膝下の、それぞれの十二経絡上に位置する一連の5つの経穴。五行学説と関連がある。
氣街		5.1.75	qi thoroughfare	a pathway along which qi gathers and flows	これに沿って気が集まり流れる経路。
良導点		5.1.76	ryodo points	points or small areas of the skin where the electric resistance is remarkably lower than the surrounding skin when a low-voltage electric circuit is set up	低電圧の電気回路を設置した時、周囲の皮膚よりも電気抵抗が顕著に低い経穴または皮膚の狭い領域。
輸穴特異性		5.1.77	special characteristics of acupuncture points	specific action and therapeutic effect of acupuncture points	経穴の特異的な作用および治療効果
經穴部位	經穴部位; 輸穴定位(法)	5.1.78	acupuncture point location	(method of determining) the location of acupuncture points	経穴の位置（の決定法）
輸穴定位	經穴部位; 輸穴定位(法)	5.1.78	acupuncture point location	(method of determining) the location of acupuncture points	経穴の位置（の決定法）
輸穴定位法	經穴部位; 輸穴定位(法)	5.1.78	acupuncture point location	(method of determining) the location of acupuncture points	経穴の位置（の決定法）
體表解剖標誌定位法	體表解剖標誌定位法; 自然標誌定位法	5.1.79	location of points by anatomical landmarks	location of points according to various anatomical landmarks of the body surface	体表のさまざまな解剖学的標識点による経穴の位置決定法。
自然標誌定位法	體表解剖標誌定位法; 自然標誌定位法	5.1.79	location of points by anatomical landmarks	location of points according to various anatomical landmarks of the body surface	体表のさまざまな解剖学的標識点による経穴の位置決定法。

検索表示（原表示の複数語を分離）	IST原表示	Code	Term	Definition/Description	定義/記述
骨度折量定位法	骨度折量定位法;<br>骨度分寸定位法;<br>骨度法	5.1.80	location of points by bone standard	the length of equally divided portions of a certain long bone or the distance between two anatomical landmarks, taken as one cun, as a unit of measurement for locating points, the same as location of points by skeletal standard	特定の長骨を等分した部分の長さ、または2つの解剖学的標識点間の距離を1寸として、経穴の位置の測定単位とした定位法。location of points by skeletal standardと同義。
骨度分寸定位法	骨度折量定位法;<br>骨度分寸定位法;<br>骨度法	5.1.80	location of points by bone standard	the length of equally divided portions of a certain long bone or the distance between two anatomical landmarks, taken as one cun, as a unit of measurement for locating points, the same as location of points by skeletal standard	特定の長骨を等分した部分の長さ、または2つの解剖学的標識点間の距離を1寸として、経穴の位置の測定単位とした定位法。location of points by skeletal standardと同義。
骨度法	骨度折量定位法;<br>骨度分寸定位法;<br>骨度法	5.1.80	location of points by bone standard	the length of equally divided portions of a certain long bone or the distance between two anatomical landmarks, taken as one cun, as a unit of measurement for locating points, the same as location of points by skeletal standard	特定の長骨を等分した部分の長さ、または2つの解剖学的標識点間の距離を1寸として、経穴の位置の測定単位とした定位法。location of points by skeletal standardと同義。
骨度分寸		5.1.81	bone proportional cun	the length of equally divided portions of a certain long bone taken as one cun, a unit of measurement, the same as skeletal proportional cun	特定の長骨を等分した部分の長さを1寸として測定単位とした定位法。skeletal proportional cun と同義
指寸定位法		5.1.82	location of point by finger cun	a method of locating points by using the width of the patient' s finger as unit of measurement	患者の指の幅を測定単位とした、経穴の位置決定法。
同身寸		5.1.83	body cun	unit of length for measurement in locating points by dividing a certain part of the patient's body into certain divisions of equal length, B-cun in brief	経穴の位置決定のための長さ測定単位。患者の体の特定の部位を、等しい長さに一定に分割したもの。簡単にB寸とも呼ばれる。
手指同身寸		5.1.84	finger cun	the width of the patient's finger taken as a unit of measurement, F-cun in brief	患者の指の幅を測定の単位としたもの。簡単にF寸とも呼ばれる。
中指同身寸		5.1.85	middle finger cun	the length between the ends of the twisted folds at the radial aspect of the second segment of the patient's middle finger when bent, taken as one cun, a unit of measurement	患者の中指を曲げたときの第1関節と第2関節のしわの間の長さを1寸とした測定単位。
拇指同身寸		5.1.86	thumb cun	the width of the phalangeal joint of the patient's thumb, taken as one cun, a unit of measurement	患者の親指の指関節の幅を1寸とした測定単位。
横指同身寸	横指同身寸;<br>一夫法	5.1.87	finger-breadth cun	the width of the four fingers (namely, the index finger, middle finger, ring finger and little finger) held together at the level of the proximal interphalangeal fold of the middle finger, which is taken as a unit of measurement of 3 cun	中指の近位の指節間のしわの高さの所でくっつけた、4本の指（人差し指、中指、薬指および小指）の幅を3寸とした測定単位。
一夫法	横指同身寸;<br>一夫法	5.1.87	finger-breadth cun	the width of the four fingers (namely, the index finger, middle finger, ring finger and little finger) held together at the level of the proximal interphalangeal fold of the middle finger, which is taken as a unit of measurement of 3 cun	中指の近位の指節間のしわの高さの所でくっつけた、4本の指（人差し指、中指、薬指および小指）の幅を3寸とした測定単位。
對證選穴		5.1.88	selection of points according to pattern/ syndrome	the general principle for point selection, that is, determination of the points used in acupuncture according to the pattern/syndrome	選穴の一般原則。鍼療法で用いる経穴を証に基づいて決定すること。
配穴	配穴;<br>配穴法	5.1.89	point combination	combined use of points in acupuncture for achieving desired therapeutic effect	望ましい治療効果を達成するために、鍼療法用の経穴を組み合わせること。
配穴法	配穴;<br>配穴法	5.1.89	point combination	combined use of points in acupuncture for achieving desired therapeutic effect	望ましい治療効果を達成するために、鍼療法用の経穴を組み合わせること。
上下配穴法		5.1.90	superior-inferior point combination	a method of point combination in which points on the upper limb are paired with points on the lower limb	上肢の経穴を下肢の経穴と組み合わせる配穴法。
左右配穴法		5.1.91	left-right point combination	a method of point combination in which bilateral points of a given meridian/channel are selected	特定の経脈の両側の経穴を選んで配穴する方法。
前後配穴法	前後配穴法;<br>腹背配穴法;<br>腹背陰陽配穴法	5.1.92	anterior-posterior point combination	a method of point combination in which points on the anterior aspect of the trunk are paired with corresponding points on the posterior aspect	体幹の前方にある経穴と、後方にある対応する経穴とを組み合わせる配穴法。
腹背配穴法	前後配穴法;<br>腹背配穴法;<br>腹背陰陽配穴法	5.1.92	anterior-posterior point combination	a method of point combination in which points on the anterior aspect of the trunk are paired with corresponding points on the posterior aspect	体幹の前方にある経穴と、後方にある対応する経穴とを組み合わせる配穴法。
腹背陰陽配穴法	前後配穴法;<br>腹背配穴法;<br>腹背陰陽配穴法	5.1.92	anterior-posterior point combination	a method of point combination in which points on the anterior aspect of the trunk are paired with corresponding points on the posterior aspect	体幹の前方にある経穴と、後方にある対応する経穴とを組み合わせる配穴法。
本經配穴法		5.1.93	point combination of the same meridian	a method of point combination in which only the points of an involved meridian/channel are selected	該当する経脈の経穴のみを選択する配穴法。

検索表示（原表示の複数語を分離）	IST原表示	Code	Term	Definition/Description	定義/記述
表裏配穴法	表裏配穴法; 表裏經配穴法	5.1.94	exterior-interior point combination	a method of point combination based on the exterior-interior relationship of the meridians/channels	経脈の表裏関係に基づく配穴法。
表裏經配穴法	表裏配穴法; 表裏經配穴法	5.1.94	exterior-interior point combination	a method of point combination based on the exterior-interior relationship of the meridians/channels	経脈の表裏関係に基づく配穴法。
原絡配穴法		5.1.95	source-connecting point combination	a method of point combination in which the source point of the corresponding meridian/channel is paired with the connecting point of the interior-exteriorly related meridian/channel	対応する経絡の原穴を、表裏に関連した経絡の絡穴と組み合わせる配穴法。
近部取穴		5.1.96	selection of adjacent points	selecting points close to the disease site	罹患部位に近い経穴を選択すること。
局部取穴		5.1.97	selection of local points	selecting points at the disease site	罹患部位の経穴を選択すること。
遠道取穴		5.1.98	selection of distant points	selecting points far from the disease site	罹患部位から遠い経穴を選択すること。
進鍼		5.1.99	needle insertion	penetration of the skin with the tip of the needle to a certain depth	皮膚に鍼尖を一定の深さで刺入すること。
進鍼法		5.1.100	needle insertion method	technique of inserting the needle through the skin	皮膚に鍼を刺入する技術。
雙手進鍼法		5.1.101	double-handed needle insertion	a needle insertion technique using both the right and left hands in cooperation	右手と左手を一緒に使って鍼を刺入する技術。
指切進鍼法		5.1.102	fingernail-pressing needle insertion	a two-handed needle insertion technique involving the application of pressure with a single nail	両手で行う鍼刺入技術の1つ。1つの爪で圧力を加える。
挾持進鍼法		5.1.103	hand-holding needle insertion	a two-handed needle insertion technique whereby the thumb and index finger of the pressing hand holds a sterilized cotton ball with which the shaft of the needle is wrapped	両手で行う鍼刺入技術の1つ。押手の親指と人差し指で、鍼体を包み込んだ滅菌済みコットンボールを持つ。
提捏進鍼法		5.1.104	pinching needle insertion	a two-handed needle insertion technique whereby the needle is inserted while the other hand pinches and lifts the flesh	両手で行う鍼刺入技術の1つ。鍼を刺入し、その間にもう一方の手で肉をつまんで持ち上げる。
舒張進鍼法		5.1.105	skin-spreading needle insertion	a two-hand needle insertion technique whereby the skin is stretched to facilitate needle insertion	両手で行う鍼刺入技術の1つ。皮膚を引っ張って鍼を刺入しやすくする。
單手進鍼法		5.1.106	single-handed needle insertion	a needle insertion technique using one hand only	片手のみで行う鍼刺入技術。
管鍼進鍼法		5.1.107	insertion of needle with tube	a needle insertion technique of using a fine tube as a guide for the needle	鍼のガイドとして細い管を用いる鍼刺入技術。
刺手		5.1.108	needling hand	the practitioner’s hand that holds and inserts the needle	施術者の、鍼を持ち刺入する方の手。
押手		5.1.109	pressing hand	the practitioner’s hand that presses the puncturing area with one or two fingers to facilitate needle insertion	施術者の、鍼を刺す部位を1～2本の指で圧迫して鍼を刺入しやすくする方の手。
押手法		5.1.110	hand-pressing method	a needle insertion technique whereby finger pressing is applied close to the acupuncture site in concert with puncturing	鍼刺入に合わせて、鍼療法部位の近傍を指で圧迫する鍼刺入技術。
鍼刺角度		5.1.111	angle of needle insertion	the angle formed between the shaft of needle and the skin punctured while the needle is being inserted	鍼を刺入する時の、鍼体と、鍼を刺入する皮膚との角度。
直刺		5.1.112	perpendicular insertion	needle insertion at a 90º angle to the skin	皮膚に対して90ºの角度で鍼を刺入すること。
橫刺	橫刺; 平刺; 沿皮刺	5.1.113	transverse insertion	needle insertion at a 15º angle to the skin	皮膚に対して15º の角度で鍼を刺入すること。
平刺	橫刺; 平刺; 沿皮刺	5.1.113	transverse insertion	needle insertion at a 15º angle to the skin	皮膚に対して15º の角度で鍼を刺入すること。
沿皮刺	橫刺; 平刺; 沿皮刺	5.1.113	transverse insertion	needle insertion at a 15º angle to the skin	皮膚に対して15º の角度で鍼を刺入すること。
斜刺		5.1.114	oblique insertion	needle insertion at a 45º angle to the skin	皮膚に対して 45º の角度で鍼を刺入すること。
鍼感	鍼感; 鍼響	5.1.115	needle sensation	the patient’s feeling of soreness, numbness, distension or heaviness around the point or feeling like an electric shock while needling	鍼療法中に患者が感じる、経穴周囲の痛みやしびれ、膨満感 、だるさ、あるいは電気ショックのような感じのこと。
鍼響	鍼感; 鍼響	5.1.115	needle sensation	the patient’s feeling of soreness, numbness, distension or heaviness around the point or feeling like an electric shock while needling	鍼療法中に患者が感じる、経穴周囲の痛みやしびれ、膨満感 、だるさ、あるいは電気ショックのような感じのこと。
氣至		5.1.116	qi arrival	the qi arrival feeling sensed by practitioners during needling	施術者が鍼療法中に感じる、気が到達した感じのこと。
得氣		5.1.117	obtaining qi	causing the acupuncture needle to elicit the patient’s feeling of soreness, numbness, distension, heaviness, or even sensation like an electric shock around the point together with the practitioner’s feeling of tenseness around the needle	鍼療法により、患者には経穴周囲の痛みやしびれ、膨満感、だるさ、あるいは電気ショックのような感覚を起こさせ、施術者には鍼周囲の緊張した感覚を起こさせること。
候氣		5.1.118	awaiting qi	performing manipulation to elicit needle sensation after insertion	刺入後に鍼感を引き起こす操作を行うこと。
催氣		5.1.119	hastening qi	promoting the arrival of needle sensation during acupuncture	鍼療法中に鍼感の到達を促進すること。
經絡現象		5.1.120	meridian phenomenon	physiological and pathological phenomena appearing along the course of meridians/channels	経絡に沿って現れる、生理的・病的現象のこと。
經脈循行		5.1.121	course of the meridian	the direction and route of the meridians/channels	経絡の方向および経路。
循經感傳		5.1.122	transmission of sensation along meridian	transmission of the needle sensation, the feeling of soreness, numbness, distension and heaviness along the meridian/channel	経絡に沿った鍼感（痛み、しびれ、膨満感およびだるさの感じ）の伝達。
隱性感傳		5.1.123	latent meridian transmission	the meridian/channel phenomenon that shows itself only after certain additional stimulation	特定の付加的な刺激の後にのみ現れる経絡現象。

検索表示（原表示の複数語を分離）	IST原表示	Code	Term	Definition/Description	定義/記述
經絡治療		5.1.124	meridian treatment	any treatment performed by applying stimulus to the related meridians/channels, also known as meridian therapy	関連した経絡に刺激を与えることにより行われるすべての治療。meridian therapyとしても知られる。
行鍼	行鍼;運鍼	5.1.125	needle manipulation	manipulating the needle after insertion to produce the desired effect	望ましい効果を起こすために、刺入後に鍼を操作すること。
運鍼	行鍼;運鍼	5.1.125	needle manipulation	manipulating the needle after insertion to produce the desired effect	望ましい効果を起こすために、刺入後に鍼を操作すること。
捻轉法		5.1.126	twirling method	a needle manipulation involving twirling the needle	鍼をくるくる回す鍼の操作。
提插法		5.1.127	lifting-thrusting method	a needle manipulation involving lifting and thrusting the needle	鍼を持ち上げて押し込む鍼の操作。
刮柄法		5.1.128	handle-scraping method	a needle manipulation involving scraping the handle of the needle with a finger nail as an auxiliary method of promoting the needle sensation	鍼感を促進する補助的手段として、指の爪で鍼柄をこする鍼の操作。
搓柄法		5.1.129	handle-twisting method	a needle manipulation involving twisting the handle of the needle as an auxiliary method of promoting the needle sensation	鍼感を促進する補助的手段として、鍼柄をねじる鍼の操作。
搖柄法		5.1.130	handle-waggling method	a needle manipulation involving shaking the handle of the needle as an auxiliary method of promoting the needle sensation	鍼感を促進する補助的手段として、鍼柄を振動させる鍼の操作。
彈柄法		5.1.131	handle-flicking method	a needle manipulation involving flicking the handle of the needle as an auxiliary method of promoting the needle sensation	鍼感を促進する補助的手段として、鍼柄をはじく鍼の操作。
震顫法		5.1.132	trembling method	a manipulation for promoting the needle sensation in which the practitioner lifts, thrusts and twists the needle at a high frequency and small amplitude to make it tremble	鍼感を促進する操作。施術者が、高頻度で小さな振幅で鍼を持ち上げ、突っ込み、ひねって、鍼を振動させる。
循法		5.1.133	massage along meridian	a method of hastening qi, in which the practitioner gives massage with fingers along the meridian/ channel after inserting the needle	気を促進する方法。鍼刺入後に 施術者が経脈に沿って指でマッサージを行う。
補瀉		5.1.134	supplementation and draining	supplementation means to activate and restore a decreased function to normal, while draining means to expel pathogenic factors and thus to restore hyperactivity to normal, the same as reinforcement and reduction	低下した機能を補法で活性化させて正常に回復させながら、瀉法で病邪を排除し、活動亢進を正常に回復させること。reinforcement and reduction と同義。
捻轉補瀉		5.1.135	twirling supplementation and draining	reinforcement or reduction achieved by rotating the needle after the qi is obtained, the same as twirling reinforcement and reduction	気が得られた後、鍼を回すことにより、補瀉を行うこと。twirling reinforcement and reductionと同義。
捻轉補瀉法		5.1.136	twirling supplementation and draining method	the manipulation of rotating the needle for achieving reinforcement or reduction, the same as the twirling reinforcement and reduction method	補瀉を行うために、鍼を回す操作。 twirling reinforcement and reduction methodと同義。
提插補瀉		5.1.137	lifting-thrusting supplementation and draining	reinforcement or reduction achieved by lifting and thrusting the needle after the qi is obtained, the same as lifting-thrusting reinforcement and reduction	気が得られた後、鍼を持ち上げて押し込むことにより補瀉を行うこと。lifting-thrusting reinforcement and reductionと同義。
提插補瀉法		5.1.138	lifting-thrusting supplementation and draining method	the manipulation of lifting and thrusting the needle for achieving reinforcement or reduction, the same as the lifting-thrusting reinforcement and reduction method	補瀉を行うために、鍼を持ち上げて押し込む操作。 lifting-thrusting reinforcement and reduction methodと同義
迎隨補瀉		5.1.139	directional supplementation and draining	reinforcement or reduction achieved by inserting the needle with the direction or against the direction of the meridian/channel course, the same as directional reinforcement and reduction	経絡の経路と同じ方向または逆の方向に鍼を刺入することで行う補瀉。directional reinforcement and reductionと同義。
迎隨補瀉法		5.1.140	directional supplementation and draining method	the manipulation of directing the needle with or against the meridian/channel course for achieving reinforcement or reduction, the same as the directional reinforcement and reduction method	補瀉を行うために、経絡の経路と同じ方向または逆の方向に鍼を向ける操作。directional reinforcement and reduction method と同義。
疾徐補瀉	疾徐補瀉;徐疾補瀉	5.1.141	quick-slow supplementation and draining	reinforcement or reduction achieved by varying the relative speed of insertion and extraction of the needle, the same as quick-slow reinforcement and reduction	鍼の刺入と抜去の相対速度を変えることによって行う補瀉。quick-slow reinforcement and reductionと同義。
徐疾補瀉	疾徐補瀉;徐疾補瀉	5.1.141	quick-slow supplementation and draining	reinforcement or reduction achieved by varying the relative speed of insertion and extraction of the needle, the same as quick-slow reinforcement and reduction	鍼の刺入と抜去の相対速度を変えることによって行う補瀉。quick-slow reinforcement and reductionと同義。
疾徐補瀉法	疾徐補瀉法;徐疾補瀉法	5.1.142	quick-slow supplementation and draining method	the manipulation of varying the relative speed of insertion and extraction of the needle for achieving reinforcement or reduction, the same as the quick-slow reinforcement and reduction method	補瀉を行うために、鍼の刺入 と抜去の相対速度を変える操作。quick-slow reinforcement and reduction method と同義。
徐疾補瀉法	疾徐補瀉法;徐疾補瀉法	5.1.142	quick-slow supplementation and draining method	the manipulation of varying the relative speed of insertion and extraction of the needle for achieving reinforcement or reduction, the same as the quick-slow reinforcement and reduction method	補瀉を行うために、鍼の刺入 と抜去の相対速度を変える操作。quick-slow reinforcement and reduction method と同義。
呼吸補瀉		5.1.143	respiratory supplementation and draining	reinforcement or reduction achieved by inserting and extracting the needle in coordination with the patient's respiration, the same as respiratory reinforcement and reduction	患者の呼吸に合わせて鍼を刺入・抜去することで達成される補瀉。respiratory reinforcement and reductionと同義。
呼吸補瀉法		5.1.144	respiratory supplementation and draining method	the manipulation of inserting and extracting the needle in coordination with the patient's respiration for achieving reinforcement or reduction, the same as the respiratory reinforcement and reduction method	補瀉を行うために、患者の呼吸に合わせて鍼を刺入・抜去する操作。respiratory reinforcement and reduction methodと同義。
開闔補瀉		5.1.145	open-closed supplementation and draining	reinforcement or reduction achieved by opening or closing the insertion hole after withdrawal of the needle, the same as open-closed reinforcement and reduction	鍼の抜去後、刺入穴を開けるまたは閉じることにより行う補瀉。open-closed reinforcement and reduction と同義。
開闔補瀉法		5.1.146	open-closed supplementation and draining method	the manipulation of opening or closing the insertion hole after needle withdrawal for achieving reinforcement or reduction, the same as the open-closed reinforcement and reduction method	補瀉を行うために、鍼の抜去後、刺入穴を開けるまたは閉じる操作。open-closed reinforcement and reduction methodと同義。

検索表示（原表示の複数語を分離）	IST原表示	Code	Term	Definition/Description	定義/記述
平補平瀉		5.1.147	neutral supplementation and draining	equal reinforcement and reduction by lifting and thrusting evenly with the same amplitude or rotation at a favorable angle, the same as neutral reinforcement and reduction	都合の良い角度で、同じ振幅または回転で均等に持ち上げ、押し込むことによる平等な補瀉。neutral reinforcement and reductionと同義。
平補平瀉法		5.1.148	neutral supplementation and draining method	a form of needle manipulation with equal reinforcement and reduction indicated in a case with combined excess and deficiency or no distinct excess or deficiency, the same as the neutral reinforcement and reduction method	平等に補法と瀉法を行う鍼操作の1つ。実と虚が組み合わせっている症例または虚実がはっきりしない症例に適用される。neutral reinforcement and reduction method と同義。
燒山火	燒山火(法)	5.1.149	mountain-burning fire (method)	a complex needle manipulation to achieve reinforcement with a local or generalized feeling of intense heat	局所的なまたは全身の強い熱感を強化するために行う複合的な鍼操作。
燒山火法	燒山火(法)	5.1.149	mountain-burning fire (method)	a complex needle manipulation to achieve reinforcement with a local or generalized feeling of intense heat	局所的なまたは全身の強い熱感を強化するために行う複合的な鍼操作。
透天凉	透天凉(法)	5.1.150	heaven-penetrating cooling (method)	a complex needle manipulation to achieve reduction with a local or generalized feeling of cooling	局所的なまたは全身の涼感を強化するために行う複合的な鍼操作。
透天凉法	透天凉(法)	5.1.150	heaven-penetrating cooling (method)	a complex needle manipulation to achieve reduction with a local or generalized feeling of cooling	局所的なまたは全身の涼感を強化するために行う複合的な鍼操作。
補母瀉子法		5.1.151	mother-supplementing/reinforcing child-draining/reducing method	a therapeutic procedure in which the mother-child relation is determined according to the five-phases attributions of the treated meridian/channel and the transport point, and then the points are selected according to the rule of reinforcing the mother in deficiency conditions and reducing the child in excess cond itions,the same as the mother-reinforcing child-reducing method	治療する経絡および輸穴の五行属性にしたがって母子関係が決定され、経穴を、虚ならば母を補い、実ならば子を瀉すという法則に基づいて選択する治療法。mother-reinforcing child-reducing methodと同義。
子午流注		5.1.152	midnight-midday ebb flow	an ancient acupuncture theory which maintains the concept that the state of qi and blood in the various meridians/channels and the receptivity of different points varies in time and the most effective needling time can be calculated in terms of heavenly stems and earthly branches	古代の鍼療法理論の1つ。経絡の気と血の状態および異なる経穴の感受性が時間によって異なり、最も有効な鍼刺入の時間が天干地支の観点から算出できるという概念を維持している。
子午流注鍼法		5.1.153	midnight-midday ebb flow acupuncture	acupuncture performed in accordance with the midnight-midday ebb flow calculation	子午流注の算出に従って行う鍼療法
納甲法	納甲法;納干法	5.1.154	day-prescription of points	determination of the points that are opening on a given day according to the heavenly stems in correspondence with the viscera and meridians/ channels	臓および経絡に対応した天干に従って、特定の日に開く経穴を決定すること。
納干法	納甲法;納干法	5.1.154	day-prescription of points	determination of the points that are opening on a given day according to the heavenly stems in correspondence with the viscera and meridians/ channels	臓および経絡に対応した天干に従って、特定の日に開く経穴を決定すること。
納支法	納子法;納支法	5.1.155	hour-prescription of points	determination of the points that are opening according to the two-hour periods in correspondence with viscera	臓と一致した、2時間ごとに開く経穴を決定すること。
納子法	納子法;納支法	5.1.155	hour-prescription of points	determination of the points that are opening according to the two-hour periods in correspondence with viscera	臓と一致した、2時間ごとに開く経穴を決定すること。
靈龜八法		5.1.156	eightfold method of the sacred tortoise	a method of selecting confluence points of the eight vessels in accordance with the variations of the eight trigrams, nine squares, heavenly stems and earthly branches	八卦、九宮、天干および地支の変化に従い、八脈交会穴を選択する方法。
刺法		5.1.157	needling method	a traditional therapy in which qi and blood of the meridians/channels are activated and the functions of the body are regulated by stimulating certain sites on the body with needles	体の特定の部位を鍼で刺激することにより、経絡の気と血を活性化し、体の機能を調節する、伝統的な治療法。
五刺		5.1.158	five needling (methods)	a collective term for five ancient needling techniques used in accordance with the pathological changes of the five viscera, i.e., half needling, leopard-spot needling, joint needling, join valley needling and transport point needling	五臓の病的変化に基づく5つの古代の鍼技術の総称。すなわち、半刺、豹文刺、関刺、合谷刺および輸刺。
半刺		5.1.159	half needling	one of the five needling techniques characterized by shallow insertion and swift withdrawal of the needle, also called shallow needling	五刺の1つ。浅く刺入し、速やかに抜去するのが特徴。shallow needlingとも呼ばれる。
豹文刺		5.1.160	leopard-spot needling	one of the five needling techniques characterized by pricking with a three-edged needle around the point	五刺の1つ。経穴の周囲に三稜鍼を刺すのが特徴。
關刺		5.1.161	joint needling	one of the five needling techniques by puncturing the tendon close to the joint	五刺の1つ。関節付近の腱を刺す。
合谷刺		5.1.162	join valley needling	one of the five needling techniques for treating numbness and pains of muscles by puncturing the muscles of the affected region directly with the needle going obliquely right and left just like the claws of a chicken, also called multi-direction needling	五刺の1つ。罹患領域の筋肉に、ニワトリの鉤爪のように斜方向に左右に鍼を直接刺すことにより、筋肉のしびれと疼痛を治療する。multi-direction needlingとも呼ばれる。
輸刺		5.1.163	transport point needling	an ancient needling method characterized by deep perpendicular puncture to the bone	古代の鍼療法。骨に向かって深く垂直に刺入するのが特徴。
九刺		5.1.164	nine needling (methods)	a collective term for nine ancient techniques of needling used for treating nine types of syndrome, i.e., transport point needling, distant needling, meridian/channel needling, collateral/network needling, intermuscular needling, great drainage needling, skin needling, red-hot needling and contralateral meridian/channel needling	9タイプの証の治療に用いられる9つの古代の鍼技術の総称。すなわち、輸刺、遠道刺、経刺、絡刺、分刺、大瀉刺、毛刺、焠刺および巨刺。
遠道刺		5.1.165	distant needling	an ancient needling method in which the needling point is selected at the lower body along the meridian/ channel distant from the disease site of the upper body	古代の鍼刺入法の1つ。上半身の疾患部位から遠い、下半身の経絡に沿って、鍼を刺す経穴を選択する。
經刺		5.1.166	meridian needling	an ancient needling method by puncturing the site of meridian/channel where nodulation or blood stasis appears	古代の鍼刺入法の1つ。結節や血瘀が現れている経絡の部位に鍼を刺す。
絡刺		5.1.167	collateral needling	an ancient needling method for bloodletting by pricking the small vessels with a three-edged needle	古代の鍼刺入法の1つ。小さな血管を三稜鍼で刺すことにより瀉血する。
分刺		5.1.168	intermuscular needling	an ancient needling method by puncturing directly into the muscle	古代の鍼刺入法の1つ。筋肉を直接刺す。
大瀉刺		5.1.169	great drainage needling	an ancient needling method referring to incision and drainage of pus and blood	古代の鍼刺入法の1つ。膿および血を切開し排出する。

検索表示（原表示の複数語を分離）	IST原表示	Code	Term	Definition/Description	定義/記述
毛刺		5.1.170	skin needling	an ancient needling method characterized by shallow puncture of the skin	古代の鍼刺入法の1つ。皮膚を浅く刺すのが特徴。
焠刺		5.1.171	red-hot needling	an ancient needling method involving the swift pricking with a red hot needle	古代の鍼刺入法の1つ。赤く焼いた鍼で速やかに刺す。
巨刺		5.1.172	contralateral meridian needling	an ancient needling method by puncturing the point of the meridian/channel contralateral to the diseased side, also called opposing needling	古代の鍼刺入法の1つ。 罹患した側と対側の経絡の経穴を刺す。opposing needlingとも呼ばれる。
繆刺		5.1.173	contralateral collateral needling	an ancient needling method characterized by needling collateral/network contralateral to the diseased side, also called contralateral insertion	古代の鍼刺入法の1つ。罹患した側と対側の絡脈に鍼を刺すのが特徴。contralateral insertion とも呼ばれる。
偶刺		5.1.174	paired needling	an ancient needling method by puncturing with a pair of needles, one anterior to and the other posterior to the disease site	古代の鍼刺入法の1つ。一対の鍼（1本は罹患部位の前方、もう1本は後方）を刺す。
報刺		5.1.175	successive trigger needling	an ancient needling method: while needling directly at the tender point , searching for other tender points over the surrounding area and needling in succession	古代の鍼刺入法の1つ。痛点に直接鍼を刺し、周囲の他の痛点を探して、連続して鍼を刺入する。
恢刺		5.1.176	relaxing needling	an ancient needling method: inserting the needle from the side and then puncturing the contracted muscle in different directions to induce relaxation	古代の鍼刺入法の1つ。横から鍼を刺入し、次に収縮した筋肉に別の方向から刺して、弛緩させる。
齊刺		5.1.177	triple needling	an ancient needling method involving one perpendicular needling with two more needling by its side	古代の鍼刺入法の1つ。直鍼刺を1回行い、その横からさらに2本の鍼を刺入する。
揚刺		5.1.178	shallow surround needling	an ancient needling method: needling the center of a point with additional needling anterior, posterior, right and left to the center of the point	古代の鍼刺入法の1つ。経穴の中央に鍼を刺入し、さらに経穴の中央の前後左右に別の鍼を刺入する。
直鍼刺		5.1.179	perpendicular needling	an ancient needling method by inserting the needle beneath the lifted skin	古代の鍼刺入法の1つ。持ち上げた皮膚の真下に鍼を刺入する。
短刺		5.1.180	short thrust needling	an ancient needling method: inserting the needle deep to the bone while gently shaking the handle, followed by short and swift lift and thrust	古代の鍼刺入法の1つ。鍼柄を穏やかに振動させながら骨に向かって深く鍼を刺入し、短時間速やかに持ち上げて押し込む。
浮刺		5.1.181	superficial needling	an ancient needling method characterized by shallow oblique puncturing	古代の鍼刺入法の1つ。浅く斜めに刺すのが特徴。
陰刺		5.1.182	yin needling	an ancient needling method in which bilateral points are selected for puncture	古代の鍼刺入法の1つ。両側の経穴を選択して刺す。
傍鍼刺		5.1.183	proximate needling	an ancient needling method involving a perpendicular needling followed by two oblique needling in the adjacent area	古代の鍼刺入法の1つ。直鍼刺の後、近接した領域に2本斜めに鍼を刺入する。
贊刺		5.1.184	repeated shallow needling	an ancient needling method characterized by multiple shallow needle insertions causing bleeding	古代の鍼刺入法の1つ。出血を引き起こす複数の浅い鍼刺入が特徴。
留鍼	留鍼; 置鍼	5.1.185	needle retention	retaining the needle in the point for a period of time to maintain and prolong the effect	経穴に鍼をしばらく保持して、効果を維持し長引かせること。
置鍼	留鍼; 置鍼	5.1.185	needle retention	retaining the needle in the point for a period of time to maintain and prolong the effect	経穴に鍼をしばらく保持して、効果を維持し長引かせること。
皮下留鍼法		5.1.186	subcutaneous needle retention method	a manipulation whereby the needle is retained under the skin for an extended period of time	皮下に鍼を長時間留置する手技。
埋鍼法		5.1.187	needle-embedding method	a treatment for relieving or curing illness by embedding in the skin a small needle or needles at certain points	特定の経穴に小型の鍼を1本または複数本埋め込み、疾患を緩和または治療する方法。
散刺法		5.1.188	scattered needling method	a method of treatment by pricking with a three-edged needle around the disease site	罹患部位の周囲を三稜鍼で刺す治療法。
挑刺法		5.1.189	piercing method	a method involving picking out a small piece of fibrous substance with a needle and squeezing out a small amount of fluid	線維状の物質の小片を鍼でつつきだし、少量の津を絞り出す方法。
點刺		5.1.190	pricking	a fast piercing method in acupuncture, usually with a three-edged needle	素早く刺し通す鍼療法の方法。通常三稜鍼で行う。
點刺療法		5.1.191	pricking therapy	a therapy by applying pricking method	點刺による治療。
刺絡法		5.1.192	pricking bloodletting method	a therapeutic method whereby a superficial vein is pierced with a three-edged needle to let out a small amount of blood	浅静脈を三稜鍼で突き通し、少量の血を流出させる治療法。
刺絡療法	刺絡療法; 刺絡法	5.1.193	collateral vessel pricking therapy	a method of treatment performed by pricking the small vessels for bloodletting	小さい血管を刺して瀉血させる治療法。
刺絡法	刺絡療法; 刺絡法	5.1.193	collateral vessel pricking therapy	a method of treatment performed by pricking the small vessels for bloodletting	小さい血管を刺して瀉血させる治療法。
出鍼		5.1.194	needle withdrawal	removal of an acupuncture needle from the body	体から鍼を抜去すること。
出鍼法		5.1.195	needle withdrawal method	the method of removing the acupuncture needle from the body	体から鍼を抜去する方法。
暈鍼		5.1.196	faint during cupuncture treatment	an adverse reaction to acupuncture; a feeling of faintness, dizziness, nausea and cold sweating during and/or after needling, also called needle sickness	鍼療法に対する有害反応。鍼療法中や鍼療法後の、失神、めまい、悪心、冷汗。needle sicknessとも呼ばれる。
彎鍼		5.1.197	bending of the needle	an abnormal condition occurring during acupuncture, in which the needle becomes bent after insertion in the body	鍼療法中に生じる異常な状態。体に刺入した後、鍼が曲がること。
折鍼	折鍼; 斷鍼	5.1.198	needle breakage	breaking of an needle below the skin when applying acupuncture treatment	鍼療法を行った際に、皮下で鍼が折れること。
斷鍼	折鍼; 斷鍼	5.1.198	needle breakage	breaking of an needle below the skin when applying acupuncture treatment	鍼療法を行った際に、皮下で鍼が折れること。
滯鍼		5.1.199	stuck needle	an accidental condition occurring during needling, in which the needle is impossible to rotate, lift or thrust	鍼療法中に生じる事故。鍼を曲げたり、持ち上げたり、押し込んだりできなくなること。
鍼適應症		5.1.200	acupuncture indications	the conditions in which acupuncture is advisable or necessary	鍼療法が推奨されるまたは必要な状態。
鍼禁忌症		5.1.201	acupuncture contraindications	any condition which renders acupuncture inappropriate or undesirable	鍼療法が不適切なまたは望ましくないすべての状態。

検索表示（原表示の複数語を分離）	IST原表示	Code	Term	Definition/Description	定義/記述
鍼不適應症		5.1.202	non-indication of acupuncture	the conditions in which acupuncture is not advisable	鍼療法が推奨されない状態。
禁鍼穴		5.1.203	needling-prohibited point	points to which needling must not be applied	鍼を適用してはならない部位。
刺鍼抵抗		5.1.204	insertion resistance	resistance to needle insertion	鍼刺入に抵抗すること。
鍼敏感人		5.1.205	needling-sensitive person	one who responds easily and quickly to acupuncture	鍼療法に容易に迅速に反応する人。
磁療儀		5.1.206	magneto-therapeutic device	an instrument that applies a magnetic force to acupoints for curing disease	疾患を治癒させるため、経穴に磁力を適用する器具。
電鍼		5.1.207	electro-acupuncture	electric stimulation of the needle following insertion	刺入後に鍼を電気刺激すること。
電鍼儀		5.1.208	electro-acupuncture device	an instrument that applies pulses of electrical current to stimulate the acupuncture site	電流パルスを適用し、鍼療法部位を刺激する器具。
電鍼療法		5.1.209	electro-acupuncture therapy	treatment of disease with electro-acupuncture	電鍼を用いた疾患の治療。
激光鍼		5.1.210	laser acupuncture	a variant of acupuncture in which needling is replaced by laser irradiation on the points	経穴に、鍼の代わりにレーザー照射を行う鍼療法の一種。
穴位激光照射法		5.1.211	laser acupuncture therapy	a therapy performed by laser irradiation on the acupuncture points	経穴にレーザー照射を行う治療。
知熱感度測定器	知熱感度測定器; 溫度感覺測定器	5.1.212	thermesthesiometer	an instrument for measuring heat sensation	熱感を測定する器具。
溫度感覺測定器	知熱感度測定器; 溫度感覺測定器	5.1.212	thermesthesiometer	an instrument for measuring heat sensation	熱感を測定する器具。
藥鍼		5.1.213	herbal acupuncture	injection of medicinal solution into acupuncture points	経穴に薬剤液を注射すること。
藥鍼療法		5.1.214	herbal acupuncture therapy	combined therapy of acupuncture and medication by which medicinal solution is injected into the acupuncture point	経穴に薬剤液を注射する、鍼療法と薬剤療法の併用療法。
電熱鍼		5.1.215	electrothermic needle	an acupuncture instrument that produces a thermal effect by means of electricity	電気により熱効果を起こす鍼療法器具。
微波鍼灸		5.1.216	microwave acumoxa	application of microwave radiation to the inserted needle to produce both acupuncture and moxibustion effects	刺入した鍼にマイクロ波放射を適用し、鍼療法と灸療法の両方の効果を生じさせること。
微波鍼療法		5.1.217	microwave acupuncture therapy	treatment of disease with microwave acumoxa	微波鍼灸を用いた疾患の治療。
蜂鍼		5.1.218	bee venom acupuncture	a special type of acupuncture performed by bee sting (or injection of episin) at a certain point or cutaneous region of the meridian/channel for therapeutic purposes, particularly for pain relief	特定の経穴または経絡の皮膚を、治療目的（特に疼痛緩和）で蜂に刺させる（またはｴﾋﾟｼﾝの注射） 特殊な鍼療法。
穴位注射		5.1.219	acupuncture point injection	injection of liquid medicine into the acupuncture point	経穴に液体の薬剤を注射すること。
穴位注射療法		5.1.220	acupuncture point injection therapy	combined therapy of acupuncture and medication by which liquid medicine is injected into the acupuncture point	鍼療法と経穴に液体の薬剤を注射する薬剤療法の併用療法。
割治療法		5.1.221	incision therapy	a therapy involving incision at a certain point and removal of a small amount of subcutaneous tissue	特定の経穴を切開し、少量の皮下組織を除去する治療。
鍼刺麻醉法		5.1.222	acupuncture anaesthesia	a method of inducing an anesthetic effect through needling for a surgical operation	外科手術のため、鍼により麻酔効果を生じさせる方法。
脊髓分節刺鍼		5.1.223	segmental needling	needling to an area of a spinal segment that is associated with a disordered structure	不調のある構造と関連した脊髄分節の一領域に対する鍼療法。
脊髓分節外刺鍼		5.1.224	non-segmental needling	needling to an area of a spinal segment that is not associated with a disordered structure	不調のある構造と関連のない脊髄分節の一領域に対する鍼療法。
發痛点		5.1.225	trigger point	a sensitive area of the body which produces a reaction elsewhere in the body when stimulated	刺激したときに、体のどこかに反応を起こす、感受性の高い体の領域。
發痛点刺鍼		5.1.226	trigger point needling	a type of acupuncture in which the trigger points are needled for therapeutic purposes	治療目的で発痛点に鍼を刺す、鍼療法の一種。
壓痛点刺鍼		5.1.227	tender point needling	a type of acupuncture in which the tender points are needled for therapeutic purposes	治療目的で壓痛点に鍼を刺す、鍼療法の一種。
筋肉刺鍼		5.1.228	intramuscular stimulation needling	a needle stimulating treatment for muscle shortening in deep muscles, especially effective for chronic pain of neuropathic origin, also known as needling myofascial trigger points	深部の筋肉の筋収縮に対する鍼刺激治療。特に神経因性の慢性疼痛に有効。needling myofascial trigger pointsとしても知られる。
傍神經刺鍼		5.1.229	paraneural needling	a stimulating method in which practitioners insert acupuncture needles into the paraneural tissue	施術者が神経近傍の組織に鍼を刺入する刺激法。
筋鍼通電療法	筋鍼通電療法; 筋pulse療法	5.1.230	muscle electro-needling	a stimulating method in which practitioners insert acupuncture needles into muscle and apply electrodes on the needles, thereby allowing electrical current to pass through needles	施術者が筋肉に鍼を刺入し、鍼に電極を付けて、鍼に電流を流す刺激法。
筋pulse療法	筋鍼通電療法; 筋pulse療法	5.1.230	muscle electro-needling	a stimulating method in which practitioners insert acupuncture needles into muscle and apply electrodes on the needles, thereby allowing electrical current to pass through needles	施術者が筋肉に鍼を刺入し、鍼に電極を付けて、鍼に電流を流す刺激法。

検索表示（原表示の複数語を分離）	IST原表示	Code	Term	Definition/Description	定義/記述
神經鍼通電療法	神經鍼通電療法; 神經pulse療法	5.1.231	nerve electro-needling	a stimulating method in which practitioners insert acupuncture needles close to the nerve and apply electrodes on the needles, thereby allowing electrical current to pass through needles	施術者が神経の近くに鍼を刺入し、鍼に電極を付けて、鍼に電流を流す刺激法。
神經pulse療法	神經鍼通電療法; 神經pulse療法	5.1.231	nerve electro-needling	a stimulating method in which practitioners insert acupuncture needles close to the nerve and apply electrodes on the needles, thereby allowing electrical current to pass through needles	施術者が神経の近くに鍼を刺入し、鍼に電極を付けて、鍼に電流を流す刺激法。
皮下鍼通電療法	皮下鍼通電療法; 皮下pulse療法	5.1.232	subcutaneous electro-needling	a stimulating method in which practitioners insert acupuncture needles into the subcutaneous tissue and apply electrodes on the needles, thereby allowing electrical current to pass through needles	施術者が皮下組織に鍼を刺入し、鍼に電極を付けて、鍼に電流を流す刺激法。
皮下pulse療法	皮下鍼通電療法; 皮下pulse療法	5.1.232	subcutaneous electro-needling	a stimulating method in which practitioners insert acupuncture needles into the subcutaneous tissue and apply electrodes on the needles, thereby allowing electrical current to pass through needles	施術者が皮下組織に鍼を刺入し、鍼に電極を付けて、鍼に電流を流す刺激法。
椎間關節鍼通電療法		5.1.233	facet joint electro-needling	a stimulating method in which practitioners insert the acupuncture needles on the facet joint of the vertebrae, and apply electrodes on the needles, thereby allowing electrical current to pass through needles	施術者が脊椎骨の椎間関節に鍼を刺入し、鍼に電極を付けて、鍼に電流を流す刺激法。
椎間關節pulse療法		5.1.233	facet joint electro-needling	a stimulating method in which practitioners insert the acupuncture needles on the facet joint of the vertebrae, and apply electrodes on the needles, thereby allowing electrical current to pass through needles	施術者が脊椎骨の椎間関節に鍼を刺入し、鍼に電極を付けて、鍼に電流を流す刺激法。
僞鍼		5.1.234	sham acupuncture	(1) mimic needling used as a placebo control in clinical research on the effect of acupuncture; (2) devices to mimic needling	(1) 鍼療法の効果に関する臨床研究において、プラセボ対照として用いる偽の鍼療法 (2) 偽の鍼療法のための道具。
灸(法)		**5.2.0**	**Moxibustion**		
灸	灸(法)	5.2.1	moxibustion	a therapeutic procedure involving ignited material (usually moxa) to apply heat to certain points or areas of the body surface for curing disease through regulation of the function of meridians/channels and visceral organs	点火した物質（通常もぐさ）で特定の経穴または体表の領域に熱を与え、経絡および内臓機能を調節することで疾患を治療する方法。
灸法	灸(法)	5.2.1	moxibustion	a therapeutic procedure involving ignited material (usually moxa) to apply heat to certain points or areas of the body surface for curing disease through regulation of the function of meridians/channels and visceral organs	点火した物質（通常もぐさ）で特定の経穴または体表の領域に熱を与え、経絡および内臓機能を調節することで疾患を治療する方法。
艾		5.2.2	moxa	(1) mugwort; (2) a plant from which moxa floss is prepared	(1) ヨモギ (2) 艾絨の材料となる植物
艾絨		5.2.3	moxa floss	a cotton-like material for moxibustion made from mugwort leaves	ヨモギの葉で作られた、灸療法に使う綿に似た物質。
艾炷		5.2.4	moxa cone	cone-shaped mass made of moxa floss for moxibustion	艾絨で作られた、灸療法用の円錐形の塊。
艾炷灸		5.2.5	moxa cone moxibustion	moxibustion with ignited moxa cone either directly on the skin or indirectly using an insulating medium	断熱物質を用いて、点火した艾炷を皮膚に直接または間接的に適用する灸療法
直接灸		5.2.6	direct moxibustion	moxibustion in which ignited moxa cone is applied directly on the skin surface at the point	点火した艾炷を経穴の皮膚表面に直接適用する灸療法。
麥粒灸		5.2.7	wheat-grain size cone moxibustion	a type of moxa cone moxibustion which uses moxa cones the size of wheat grains	小麦粒の大きさの艾炷を用いる艾炷灸療法の一種。
間接灸	間接灸; 間隔灸; 隔物灸	5.2.8	indirect moxibustion	moxibustion performed by placing some insulating material between the moxa cone and the skin	艾炷と皮膚の間に何らかの断熱物質を置いて行う灸療法。
間隔灸	間接灸; 間隔灸; 隔物灸	5.2.8	indirect moxibustion	moxibustion performed by placing some insulating material between the moxa cone and the skin	艾炷と皮膚の間に何らかの断熱物質を置いて行う灸療法。
隔物灸	間接灸; 間隔灸; 隔物灸	5.2.8	indirect moxibustion	moxibustion performed by placing some insulating material between the moxa cone and the skin	艾炷と皮膚の間に何らかの断熱物質を置いて行う灸療法。
隔薑灸		5.2.9	moxibustion on ginger	the application of moxibustion on a piece of fresh ginger as an insulating medium, also called ginger moxibustion	断熱物質として、一片の新鮮なショウガの上で灸療法を行うこと。ginger moxibustion とも呼ばれる。
隔鹽灸; 鹽灸		5.2.10	moxibustion on salt	the application of moxibustion on salt as an insulating medium, also called salt moxibustion	断熱物質として、塩の上で灸療法を行うこと。salt moxibustionとも呼ばれる。
隔蒜灸	隔蒜灸; 大蒜灸; 隔蒜灸	5.2.11	moxibustion on garlic	the application of moxibustion on a slice of fresh garlic as an insulating medium, also called garlic moxibustion	断熱物質として、新鮮なニンニクのスライスの上で灸療法を行うこと。garlic moxibustionとも呼ばれる。
大蒜灸	隔蒜灸; 大蒜灸; 隔蒜灸	5.2.11	moxibustion on garlic	the application of moxibustion on a slice of fresh garlic as an insulating medium, also called garlic moxibustion	断熱物質として、新鮮なニンニクのスライスの上で灸療法を行うこと。garlic moxibustionとも呼ばれる。
隔蒜灸	隔蒜灸; 大蒜灸; 隔蒜灸	5.2.11	moxibustion on garlic	the application of moxibustion on a slice of fresh garlic as an insulating medium, also called garlic moxibustion	断熱物質として、新鮮なニンニクのスライスの上で灸療法を行うこと。garlic moxibustionとも呼ばれる。
艾條		5.2.12	moxa stick	a round long stick made of moxa floss, also called moxa roll	艾絨で作られた丸くて長い棒。moxa roll とも呼ばれる。
艾條灸		5.2.13	moxa stick moxibustion	moxibustion with ignited moxa stick	点火した艾條を用いた灸療法。
艾條灸療法		5.2.14	moxa stick moxibustion therapy	a therapy by applying moxibustion with ignited moxa stick	点火した艾條を用いて灸療法を行うこと。
艾卷		5.2.15	moxa roll moxibustion	a long cylindrical roll of moxa floss wrapped tightly in paper	紙でぴったりと包んだ、長い筒状の巻いた艾絨。

検索表示（原表示の複数語を分離）	IST原表示	Code	Term	Definition/Description	定義/記述
艾卷灸		5.2.16	moxa roll moxibustion	moxibustion with ignited moxa roll, the same as moxa-stick moxibustion	点火した艾巻を用いた灸療法。艾條灸と同義。
溫和灸		5.2.17	gentle moxibustion	a type of moxa-stick moxibustion, performed by holding an ignited moxa stick at a certain distance above the patient's skin, keeping the spot warm and making it reddened but not burnt	艾條灸療法の一種。患者の皮膚から特定の距離離れたところで、点火した艾條を持ち、その点を温め、赤化させるが焼かない。
廻旋灸		5.2.18	circling moxibustion	a type of moxa-stick moxibustion, performed by keeping an ignited moxa stick at a fixed distance from the patient252s skin, but moving it in a circular direction	艾條灸療法の一種。患者の皮膚から一定の距離で、点火した艾條を持ち、艾條を円状に動かす。
雀啄灸	雀啄灸; 雀啄法	5.2.19	pecking sparrow moxibustion	a type of moxa-stick moxibustion, performed by putting an ignited moxa stick near the patient's skin, and moving it up and down like a bird's pecking so as to give strong heat to the applied spot	艾條灸療法の一種。点火した艾條を患者の皮膚の近くに置き、鳥がついばむように上下に動かして、経穴に強い熱を与える。
雀啄法	雀啄灸; 雀啄法	5.2.19	pecking sparrow moxibustion	a type of moxa-stick moxibustion, performed by putting an ignited moxa stick near the patient's skin, and moving it up and down like a bird's pecking so as to give strong heat to the applied spot	艾條灸療法の一種。点火した艾條を患者の皮膚の近くに置き、鳥がついばむように上下に動かして、経穴に強い熱を与える。
懸灸		5.2.20	suspended moxibustion	a type of moxa-stick moxibustion, in which the ignited moxa stick is held above the skin	艾條灸療法の一種。点火した艾條を皮膚の上で持つ。
灸痕		5.2.21	moxibustion scar	burn scar caused by direct moxibustion	直接灸によるやけどの跡。
無瘢痕灸	無瘢痕灸; 無痕灸; 非化膿灸	5.2.22	non-scarring moxibustion	moxibustion in which ignited moxa cone is applied directly on the skin surface at the point, but a burn is prevented and no scar is left	点火した艾炷を直接経穴の皮膚表面に適用するが、やけどさせず、灸痕を残さない灸療法
無痕灸	無瘢痕灸; 無痕灸; 非化膿灸	5.2.22	non-scarring moxibustion	moxibustion in which ignited moxa cone is applied directly on the skin surface at the point, but a burn is prevented and no scar is left	点火した艾炷を直接経穴の皮膚表面に適用するが、やけどさせず、灸痕を残さない灸療法
非化膿灸	無瘢痕灸; 無痕灸; 非化膿灸	5.2.22	non-scarring moxibustion	moxibustion in which ignited moxa cone is applied directly on the skin surface at the point, but a burn is prevented and no scar is left	点火した艾炷を直接経穴の皮膚表面に適用するが、やけどさせず、灸痕を残さない灸療法
瘢痕灸	瘢痕灸; 有痕灸; 化膿灸; 打膿灸	5.2.23	scarring moxibustion	a type of direct moxibustion in which ignited moxa is directly placed on the point until the local skin blisters, causing suppuration and leaving a scar	直接灸の一種。点火したもぐさを直接経穴に置き、皮膚の一部に水ぶくれを作らせ、化膿させて瘢痕を残す。
有痕灸	瘢痕灸; 有痕灸; 化膿灸; 打膿灸	5.2.23	scarring moxibustion	a type of direct moxibustion in which ignited moxa is directly placed on the point until the local skin blisters, causing suppuration and leaving a scar	直接灸の一種。点火したもぐさを直接経穴に置き、皮膚の一部に水ぶくれを作らせ、化膿させて瘢痕を残す。
化膿灸	瘢痕灸; 有痕灸; 化膿灸; 打膿灸	5.2.23	scarring moxibustion	a type of direct moxibustion in which ignited moxa is directly placed on the point until the local skin blisters, causing suppuration and leaving a scar	直接灸の一種。点火したもぐさを直接経穴に置き、皮膚の一部に水ぶくれを作らせ、化膿させて瘢痕を残す。
打膿灸	瘢痕灸; 有痕灸; 化膿灸; 打膿灸	5.2.23	scarring moxibustion	a type of direct moxibustion in which ignited moxa is directly placed on the point until the local skin blisters, causing suppuration and leaving a scar	直接灸の一種。点火したもぐさを直接経穴に置き、皮膚の一部に水ぶくれを作らせ、化膿させて瘢痕を残す。
實按灸		5.2.24	pressing moxibustion	moxibustion performed by placing several layers of cloth or paper on the spot, and then pressing the ignited end of a moxa stick on the cloth or paper	数層の布または紙を経穴に置き、点火した艾條の末端圧を布または紙に押しつけて行う灸療法 。
燈火灸		5.2.25	juncibustion	moxibustion performed by applying a quick momentary touch to the point with a piece of ignited oiled rush	点火した油に浸した1片のイグサで、経穴に素早く瞬間的に触れることにより行う灸療法。
天灸	天灸; 發泡灸	5.2.26	natural moxibustion	moxibustion in which irritants are applied at the points to produce blistering and local congestion, also known as vesiculation moxibustion	刺激薬を経穴に適用し、水ぶくれを作らせ、局所的にうっ血させる灸療法。vesiculation moxibustion としても知られる。
發泡灸	天灸; 發泡灸	5.2.26	natural moxibustion	moxibustion in which irritants are applied at the points to produce blistering and local congestion, also known as vesiculation moxibustion	刺激薬を経穴に適用し、水ぶくれを作らせ、局所的にうっ血させる灸療法。vesiculation moxibustion としても知られる。
太乙神鍼		5.2.27	Taiyi moxa stick moxibustion	a special moxa roll made of sandalwood, notopterygium rhizome, cassia twig, dahurian angelica root and other medicinal herbs, used for the treatment of wind-cold-dampness arthralgia, abdominal pain of cold type and dysmenorrhea	白檀、羌活、桂皮小枝、白芷およびその他の生薬で作られた特別な艾巻。風寒湿関節痛、寒タイプの腹痛、月経困難症の治療に用いられる。
雷火神鍼		5.2.28	thunder-fire wonder moxibustion	a type of medicinal moxa roll including Chinese eagle wood, common aucklandia root, frankincense, and other medicinal herbs, used for treating maladies such as cold and pain in the epigastrium and abdomen, rheumatism and dysmenorrhea	薬用の艾巻の一種。沈香、木香、乳香、およびその他の生薬を含む。上腹部の寒および疼痛、リウマチ、月経困難症などの疾患の治療に用いられる。
藥物灸	藥物灸; 藥調灸	5.2.29	medicinal moxibustion	moxibustion with the moxa cigar made of moxa and various herbal medicines	もぐさとさまざまな生薬で作られた もぐさ薬巻を用いる灸療法。
藥調灸	藥物灸; 藥調灸	5.2.29	medicinal moxibustion	moxibustion with the moxa cigar made of moxa and various herbal medicines	もぐさとさまざまな生薬で作られた もぐさ薬巻を用いる灸療法。
溫鍼療法		5.2.30	warm needling therapy	a therapy involving warm needling moxibustion	温鍼灸療法を用いる治療法。
溫灸器		5.2.31	moxa burner	a receptacle designed to hold burning moxa floss	燃えている艾絨を保持するためにデザインされた容器。
溫灸器灸		5.2.32	moxa burner moxibustion	moxibustion with a moxa burner to hold the ignited moxa floss	もぐさ燃焼器を用いて点火した艾絨を保持する灸療法。

検索表示（原表示の複数語を分離）	IST原表示	Code	Term	Definition/Description	定義/記述
筒灸		5.2.33	moxibustion with moxa tube	a practice performed by placing one end of a thin tube into the external opening of the ear and applying moxibustion to the other end of the tube	耳の穴に細い管の一端を差し込み、管のもう一端に灸をすえる手技。
線香		5.2.34	incense thread	a slender stick of incense used for moxibustion	灸療法に使われる細い香の棒。
壯數		5.2.35	number of cones	that used as the unit of measuring the amount of moxibustion	灸の量を測定する単位。
電氣灸		5.2.36	electro-moxibustion	electrical dermal stimulation used in place of moxa	もぐさの代わりに用いられる、電気的な皮膚の刺激。
灸適應症		5.2.37	moxibustion indications	the conditions in which moxibustion is advisable or necessary	灸療法が推奨されるまたは必要な状態。
灸不適應症		5.2.38	non-indication of moxibustion	the conditions in which moxibustion is not advisable	灸療法が推奨されない状態。
禁灸穴		5.2.39	moxibustion-prohibited point	acupuncture points to which moxibustion must not be applied	灸療法を行ってはならない経穴。
拔罐		**5.3.0**	**Cupping**		
拔罐		5.3.1	cupping	suction by using a vacuumized cup or jar	真空のカップまたはビンを用いた吸引。
拔罐法		5.3.2	cupping method	a therapeutic method involving the application of suction by placing a vacuumized, usually by fire, cup or jar onto the affected or any part of the body surface	通常火により真空にしたカップまたはビンを、罹患部位または体表のさまざまな部位に置いて吸引する治療法。
抽氣罐		5.3.3	suction cup	a cup or jar with a rubber valve through which the air inside can be withdrawn by an aspirator	内部の空気を吸引器で除去するためのゴム栓のついたカップまたはビン。
竹罐		5.3.4	bamboo cup	a cupping instrument made of bamboo	竹で作られた抜罐用器具。
留罐		5.3.5	retained cupping	a common method of cupping in which the cup or jar is kept at the same site for a certain period of time	カップまたはビンを一定時間同じ所に置いておく、一般的な抜罐の方法。
投火法		5.3.6	fire-insertion cupping method	a cupping procedure which involves inserting a piece of ignited alcohol-cotton or paper into a cup and pressing the cup transversely onto the treated area of the lateral side of the body	点火した一片のアルコール綿または紙をカップに入れ、体の側面の治療部位に対して横方向にカップを押しつける抜罐法。
閃火法		5.3.7	flash-fire cupping method	a cupping procedure which involves flashing the fire of a piece of ignited alcohol-cotton once around the cup's interior and pressing the cup onto the treated area immediately after removing the ignited cotton	一片の点火したアルコール綿の火を、カップ内部の周囲で一度ぱっと発火させ、点火した綿を除去してすぐに治療部位にカップを押しつける抜罐法。
貼棉法		5.3.8	cotton-burning cupping method	a cupping procedure performed by placing a thin layer of alcohol-cotton on the lower one third of the cup wall, and pressing the cup onto the treated area after igniting the cotton	アルコール綿の薄い層をカップ壁の下3分の1のところに置き、綿に点火した後、治療部位にカップを押しつける抜罐法。
走罐		5.3.9	slide cupping	a cupping method in which the cup or jar is moved on the skin surface to extend the acting area	皮膚表面上でカップまたはビンを動かして作用部位を広げる抜罐法。
藥罐		5.3.10	medicated cupping	a form of cupping therapy in which the cup or jar is put into boiling medicinal solution before use	抜罐療法の一種で、使用前にカップまたはビンを沸騰した薬物液に入れる。
刺絡拔罐法	刺絡拔罐法;刺血拔罐法	5.3.11	pricking-cupping bloodletting method	a combined method of pricking and cupping in which pricking with a three-edged needle is followed by cupping to increase bloodletting	鍼と抜罐の併用法で、三稜鍼で刺した後、抜罐を行って瀉血を増加させる。
刺血拔罐法	刺絡拔罐法;刺血拔罐法	5.3.11	pricking-cupping bloodletting method	a combined method of pricking and cupping in which pricking with a three-edged needle is followed by cupping to increase bloodletting	鍼と抜罐の併用法で、三稜鍼で刺した後、抜罐を行って瀉血を増加させる。
藥物治療		**6.0.0**	**MEDICINAL TREATMENT**		
藥	藥(物)	**6.1.0**	**Medicinal**	a medicinal substance having or designating healing or curative properties or attributes	治癒力または治療能力を有するか有するとされている薬用の物質。
藥物	藥(物)	**6.1.0**	**Medicinal**	a medicinal substance having or designating healing or curative properties or attributes	治癒力または治療能力を有するか有するとされている薬用の物質。
中藥		6.1.1	Chinese medicinal	usually referring to those medicinal substances recorded in Chinese materia medica	通常、中国の本草書に記録されている薬用の物質。
草藥		6.1.2	herbs/herbal drugs	usually referring to those medicinal substances not recorded in Chinese materia medica or only used in folk medicine, also called medicinal herbs	通常、中国の本草に記録されていない薬用の物質、または民間医療のみで使用されている薬用の物質。薬草とも呼ばれる。
本草		6.1.3	materia medica	that aspect of medical science concerned with the origin and preparation of medicinal agents, their doses and method of administration, also refers to Chinese medicinal herbs	薬剤の起源および調製、用量、投与法に関する医学。Chinese medicinal herbsとも称される。
藥材		6.1.4	medicinal material	crude natural medicinal for processing	加工用の粗天然薬剤。
道地藥材		6.1.5	authentic medicinal	genuine medicinal herbs produced at the original place	起源となる場所で生産された真の薬用植物。
炮製	炮製;修治;修事	6.1.6	processing of medicinals	a general term for treating of medicinal substances by various means before their medical use	医用に使われる前にさまざまな方法で薬用の物質を処理する際の一般的な用語。
修治	炮製;修治;修事	6.1.6	processing of medicinals	a general term for treating of medicinal substances by various means before their medical use	医用に使われる前にさまざまな方法で薬用の物質を処理する際の一般的な用語。
修事	炮製;修治;修事	6.1.6	processing of medicinals	a general term for treating of medicinal substances by various means before their medical use	医用に使われる前にさまざまな方法で薬用の物質を処理する際の一般的な用語。
切	切（製）	6.1.7	cut the medicinal	cut the medicinal into pieces	薬物を切り刻むこと。
切製	切（製）	6.1.7	cut the medicinal	cut the medicinal into pieces	薬物を切り刻むこと。
水製		6.1.8	water processing	processing by utilizing water, including washing, bleaching, soaking, refining with water, etc.	水を用いて処理すること。洗浄、漂白、浸漬、水による精製など。

検索表示（原表示の複数語を分離）	IST原表示	Code	Term	Definition/Description	定義/記述
水飛		6.1.9	water-grind	remove impurities from a powdered medicinal and at the same time obtaining finer powder by mixing it with water in a tank and allowing the supernatant turbid fluid to settle in another tank, and then collecting the deposit	タンク内の水と混ぜることで、粉末の薬物から不純物を除去し、同時により微細な粉末を得て、上層の濁った液体を別のタンクに移し、沈殿物を回収すること。
火製		6.1.10	fire processing	processing by utilizing heat or fire, including stir-baking, baking, calcining, etc.	熱または火を用いて処理すること。攪拌しながら炒る、炒る、焼成など。
清炒		6.1.11	plain stir-bake	bake a medicinal in a pan, with constant stirring and without adding any adjuvant	かき混ぜながら、補助剤を添加せずに鍋で薬物を炒ること。
炒黃		6.1.12	stir-bake to yellow	bake a medicinal in a pan, with constant stirring, till it turns yellow	かき混ぜながら、黄色くなるまで鍋で薬物を炒ること。
炒焦		6.1.13	stir-bake to brown	bake a medicinal in a pan, with constant stirring, till it turns brown	かき混ぜながら、茶色くなるまで鍋で薬物を炒ること。
炒炭		6.1.14	stir-bake to scorch	bake a medicinal in a pan, with constant stirring, till it partly turns to charcoal	かき混ぜながら、一部が炭になるまで鍋で薬物を炒ること。
炙		6.1.15	stir-bake with adjuvant	bake, with stirring, a medicinal together with adjuvant until the latter is infiltrated into the medicinal	薬物に補助剤を加えて、補助剤が薬物に浸潤するまで、かき混ぜながら炒る こと。
煅		6.1.16	calcine	burn a medicinal on a fire to make it crispy, easy to crush	薬物を火で燃やし、容易に砕けるようカリカリにすること。
煨		6.1.17	roast	bake a medicinal wrapped in wet paper or coated with dough in hot ashes till the paper or coat turns black	薬物を、濡れた紙に包むか、熱い灰の生地でコーティングして、紙かコーティングが黒くなるまで炒ること。
烘焙		6.1.18	bake	dry a medicinal over a slow fire	薬物を弱火で乾燥させること。
漂		6.1.19	long-rinse	steep the materials in clean water that is continuously replaced to eliminate impurities	連続的に置換して不純物を除去したきれいな水に、物質を浸すこと。
去火毒		6.1.20	eliminate fire toxin	remove irritants from newly prepared medicinal plasters	新しく調製した膏薬から刺激薬を除去すること。
酒製		6.1.21	processing with wine	processing a medicinal with rice wine or liquor	薬剤を日本酒または蒸留酒で処理すること。
醋製		6.1.22	processing with vinegar	processing, mostly stirring-baking, a medicinal together with vinegar	主として 攪拌しながら炒り、薬剤を酢と共に処理すること。
上品		6.1.23	top grade drug	medicinals that are non-toxic, possesses a rejuvenating effect and can be taken frequently and for a long period of time without harm	毒性がなく、若返り効果を有し、害がなく高頻度で長期間使用できる薬剤
中品		6.1.24	medium grade drug	medicinals that have no or only slight toxic effect and are effective for treating diseases or deficiency conditions	毒性がないまたは軽い毒性しかなく、疾患や欠乏症の治療に有効な薬剤。
下品		6.1.25	low grade drug	medicinals that are effective for expelling pathogens, but are toxic and should not be taken for a long period of time	病原体の排除に有効であるが、毒性があり、長期間は使用できない薬剤。
藥味		6.1.26	flavor of medicinals	taste or flavor of a medicinal, representing the basic action of that medicinal	その薬剤の基本的な作用を示す、薬物の味や風味 。
藥性		6.1.27	nature of medicinals	the basic properties of a medicinal	薬剤の基本的な性質。
氣味		6.1.28	qi and flavor	the property and flavor of a medicinal that represent the main effects of that medicinal	その薬剤の主効果を示す、薬物の性質や風味。
四氣	四氣; 四性	6.1.29	four qi	the four basic properties of medicinals, cold, hot, warmth, and coolness	薬剤の4つの基本的性質。 寒・熱・温・涼。
四性	四氣; 四性	6.1.29	four qi	the four basic properties of medicinals, cold, hot, warmth, and coolness	薬剤の4つの基本的性質。 寒・熱・温・涼。
五味		6.1.30	five flavors	the five tastes of medicinals, pungency, sweetness, sourness, bitterness, and saltiness, representing the basic actions of the medicinals	その薬剤の基本的な作用を示す、薬剤の5種類の味。辛・甘・酸・苦・鹹。
升降浮沈		6.1.31	upbearing, downbearing, floating and sinking	direction of medicinal action, upward, downward, outward, and inward	薬剤の作用の方向。升降出入。
歸經		6.1.32	meridian entry	orientation of the medicinal action according to the meridian/channel on which the therapeutic action is manifested, also called meridian tropism	どの治療作用が発現されるかに対する経絡に基づく薬剤の作用の方向。meridian tropism とも呼ばれる。
相須		6.1.33	mutual reinforcement	two medicinals with similar properties used in combination to reinforce each other’s action	それぞれの作用を増強するために併用される、同様な性質を有する2つの薬剤。
相使		6.1.34	mutual assistance	two or more medicinals used in combination, one being the principal substance while the others play a subsidiary role to reinforce the action of the former	併用して使用される2つ以上の薬剤。1つは主物質であり、もう一方は前者の作用を増強する補助的な役割を果たす。
相畏		6.1.35	mutual restraint	toxicity or side effects of a medicinal being counteracted by another	別の薬剤により中和される、薬剤の毒性または副作用。
相殺		6.1.36	mutual suppression	property of a medicinal to neutralize the toxicity of another	別の薬剤の毒性を中和する薬剤の性質。
相惡		6.1.37	mutual inhibition	property of a medicinal to weaken the action of another	別の薬剤の作用を減弱させる薬剤の性質。
相反		6.1.38	antagonism	antagonistic combination of two medicinals, if used together, one may prevent the therapeutic effect of the other and/or there may be adverse effects	拮抗的な2つの薬剤の組み合わせ。併用すると、1つの薬剤が別の薬剤の治療効果を妨げたり、有害作用を起こしたりする可能性がある。
配伍禁忌		6.1.39	prohibited combination	medicinals whose combined use is prohibited in a prescription	処方で併用が禁止されている薬剤。
十八反		6.1.40	eighteen antagonisms	incompatible medicinals which, if given in combination, are believed to have serious side effects: Radix Glycyrrhizae being antagonistic to Radix Euphorbiae Pekinensis, Flos Genkwa, Radix Euphorbiae Kansui and Sargassum; Radix Aconiti being antagonistic to Bulbus Fritillariae, Fructus Trichosanthis, Rhizoma Pinelliae, Radix Ampelopsis and Rhizoma Bletillae; and Radix Veratri Nigri being antagonistic to Radix Ginseng, Radix S alviae Miltiorrhizae, Radix Adenophorae, Radix Sophorae Flavescentis, Radix Scrophulariae, Herba Asari and Radix Paeoniae	不適合な薬剤。併用すると、重篤な副作用を起こすと考えられている。: 甘草は 京大戟、芫花、甘遂および海藻に対して拮抗作用を示す。烏頭は、貝母、白及、半夏、白斂および括蔞に拮抗作用を示す。 藜蘆は 人参、丹参、沙参、苦参、玄参、細辛および 芍薬に対して拮抗作用を示す。

検索表示（原表示の複数語を分離）	IST原表示	Code	Term	Definition/Description	定義/記述
十九畏		6.1.41	nineteen incompatibilities	medicinals of mutual restraint which, if used in combination, may restrain or neutralize each other's action: sulfur being incompatible with crude sodium sulfate; mercury being incompatible arsenic trioxide; Radix Euphorbiae Ebracteolatae being incompatible with litharge; Semen Crotonis being incompatible with Semen Pharbitidis; Flos Caryophylli being incompatible with Radix Curcumae; crystalline sodium sulfate being incompatible with Rhizoma Sparganii; Radix Aconiti and Radix Aconiti Kuznezoffi being incompatible with Cornu Rhinoceri; Radix Ginseng being incompatible with Faeces Trogopterorum; and Cortex Cinnaraomi being incompatible with Halloysitum Rubrum	相互抑制作用を示す薬剤。併用すると、お互いの作用を抑制または中和する可能性がある。硫黄は朴硝（硫酸ナトリウム）と不適合である。水銀は砒霜（三酸化砒素）と不適合である。狼毒は密陀僧（一酸化鉛）と不適合である。巴豆は牽牛子と不適合である。丁香は鬱金と不適合である。牙硝（結晶硫酸ナトリウム）は三稜と不適合である。川烏および草烏は、犀角と不適合である。人参は、五霊脂と不適合である。肉桂は、赤石脂と不適合である。
禁忌		6.1.42	contraindication	any disease, symptom or circumstance which renders the use of a medicinal or therapeutic procedure inadvisable for an individual	ヒトに薬剤や治療法の使用を推奨できない原因となる疾患、症状または状況。
妊娠禁忌		6.1.43	contraindications during pregnancy	medicinals whose administration is prohibited during pregnancy	妊娠中の投与が禁止されている薬剤。
服藥食忌		6.1.44	dietary contraindication during medication	types of food that should be avoided during the period of medication	薬剤投与中に避けるべき食物の種類。
食忌		6.1.45	dietary contraindications	abbreviation for dietary contraindications during medication	薬剤投与中の食事の禁忌の略語。
劑量		6.1.46	dosage	the measured quantity of a medicinal to be taken	使用すべき薬剤の計量した量。
方寸匕		6.1.47	square-inch-spoon	an ancient instrument for measuring the quantity of medicinals, one square-inch-spoon equal to 2.74 ml, about 2 g of powdered mineral ingredients and 1 g of powdered herbal ingredients	薬剤量を測定するための古来の道具。1方寸匕は、2.74 ml、約2 gの粉末鉱物性成分、および1 gの粉末薬草成分と等しい。
配伍		6.1.48	combination	use various medicinals jointly in a formula or prescription for producing the desired therapeutic effect and reducing toxic or side effects	望ましい治療効果を得て、毒性や副作用を低減するために、1回の処方または方剤でさまざまな薬剤を一緒に使用すること。
解表藥		6.1.49	exterior-releasing medicinal	a category of medicinals that have the effect of dispelling external pathogen from the exterior aspect of the body, usually through sweating	外因性の病原体を体の外表面から、通常発汗により排出する効果を有する薬剤のカテゴリー。
發散風寒藥		6.1.50	wind-cold-dispersing medicinal	a medicinal that has the effect of dispersing wind and cold in the treatment of an exterior pattern/syndrome	表証の治療において、風寒を消散させる効果を有する薬剤。
辛溫解表藥		6.1.51	pungent-warm exterior-releasing medicinal	an exterior-releasing medicinal pungent in flavor and warm in property, used for treating a wind-cold exterior pattern/syndrome	風寒による表証の治療に使われる、風味は辛、性質は温の、解表剤。
發散風熱藥		6.1.52	wind-heat dispersing medicinal	a medicinal that has the effect of dispersing wind and heat in the treatment of an exterior pattern/syndrome	表証の治療において 風熱を消散させる効果を有する薬剤
辛涼解表藥		6.1.53	pungent-cool exterior-releasing medicinal	an exterior-releasing medicinal pungent in flavor and cool in property, used for treating a wind-heat exterior pattern/syndrome	風熱による表証の治療に使われる、風味は辛、性質は涼の、解表剤。
清熱藥		6.1.54	heat-clearing medicinal	a category of medicinals that have the effect of clearing up internal heat in cases of externally contracted febrile diseases or fever due to yin deficiency	外感病による熱性疾患または陰虚による発熱の場合に、内熱を除去する効果を有する薬剤のカテゴリー。
清熱瀉火藥		6.1.55	heat-clearing and fire-purging medicinal	a medicinal that has the effect of clearing heat or purging fire from the interior	内部から熱を取り去る、または火を除去する効果を有する薬剤。
清熱燥濕藥		6.1.56	heat-clearing and dampness-drying medicinal	a medicinal that is effective for eliminating heat and dampness	熱湿を排除するのに有効な薬剤。
清熱解毒藥		6.1.57	heat-clearing and detoxicating medicinal	a medicinal that counteracts heat toxins	熱毒を中和する薬剤。
清熱涼血藥		6.1.58	heat-clearing and blood-cooling medicinal	a medicinal that has the effect of eliminating pathogenic heat from the nutrient and blood aspects in cases of externally contracted febrile diseases	外部拘束性の熱性疾患の場合に、栄養および血から病原性の熱を排除する効果を有する薬剤。
清虛熱藥		6.1.59	deficiency heat-clearing medicinal	a medicinal that clears heat from deficiency conditions	欠乏症から熱を除去する薬剤。
瀉下藥		6.1.60	purgative medicinal	a category of medicinals that promote defecation or even cause diarrhea, not only for relieving constipation, but also for driving stagnant matter, excessive heat and retained fluid out of the body	便秘を緩和するだけでなく、鬱滞した物質、過剰な熱、および滞留した液体を体外に排出するために、排便を促進し、下痢を起こすこともある薬剤のカテゴリー。
攻下藥		6.1.61	offensive purgative medicinal	a medicinal that has a potent purgative effect for moving the bowels and driving away excessive heat and stagnant matter	腸を動かして過剰な熱と停滞した物質を排除するために強力な下剤の効果を有する薬剤。
溫下藥		6.1.62	warm purgative medicinal	a medicinal warm in property, which relieves constipation caused by excessive cold stagnation	過剰な寒鬱滞による便秘を緩和する、性質は温の薬剤。
潤下藥		6.1.63	laxative (medicinal)	a medicinal that lubricates the intestinal tract to facilitate defecation	腸管をなめらかにして排便を促進する薬剤。
峻下逐水藥		6.1.64	drastic (purgative) water-expelling medicinal	a cathartic that causes copious water discharge for reducing accumulated fluid in anasarca, ascites and pleural effusion	全身浮腫、腹水および胸水で蓄積された液体を低減するために、大量の水を排出させる下剤。
祛風濕藥		6.1.65	wind-dampness-dispelling medicinal	a category of medicinals that dispel wind and dampness, mainly for relieving rheumatism and related conditions	主としてリウマチおよびその関連疾患を緩和するために 風湿を排除する薬剤のカテゴリー。
祛風濕散寒藥		6.1.66	wind-dampness-dispelling and cold-dispersing medicinal	a medicinal that dispels wind-dampness and disperses cold, indicated in wind-dampness arthralgia of cold type	風湿を排除し、寒を消散させる薬剤。寒型の風湿関節痛に適用される。

検索表示（原表示の複数語を分離）	IST原表示	Code	Term	Definition/Description	定義/記述
祛風濕清熱藥		6.1.67	wind-dampness-dispelling and heat-clearing medicinal	a medicinal that dispels wind-dampness and clears heat, suitable for treating wind-dampness arthralgia of heat type	風湿を排除し、熱を除去する薬剤。熱型の風湿関節痛の治療に適用される。
化濕藥		6.1.68	dampness-resolving medicinal	a medicinal with fragrant odor, warming and drying, that resolves dampness	温めて乾燥させ、湿邪を消退させる芳香性の薬剤。
利水滲濕藥	利水滲濕藥; 利濕藥	6.1.69	dampness-draining diuretic medicinal	a medicinal that increases urine excretion and water discharge for treating internal retention of dampness	湿邪の内部鬱滞を治療するために、尿排泄および水の排出を増加させる薬剤。
利濕藥	利水滲濕藥; 利濕藥	6.1.69	dampness-draining diuretic medicinal	a medicinal that increases urine excretion and water discharge for treating internal retention of dampness	湿邪の内部鬱滞を治療するために、尿排泄および水の排出を増加させる薬剤。
利水消腫藥		6.1.70	water-draining and swelling-dispersing medicinal	a medicinal that increases urine excretion for treating edema	浮腫を治療するために、尿排泄を増加させる薬剤。
利尿通淋藥		6.1.71	strangury-relieving diuretic medicinal	a medicinal that increases urine excretion and relieves strangury, mainly indicated in the treatment of dampness-heat in the lower energizer with difficult and painful discharge of urine	尿排泄を増加させ、有痛排尿困難を緩和する薬剤。主として、有痛性の排尿困難を有する下焦の湿熱の治療に適用される。
通淋藥		6.1.72	strangury-relieving medicinal	a medicinal that relieves various kinds of strangury	さまざまな有痛排尿困難を緩和する薬剤。
利濕退黃藥		6.1.73	dampness-draining anti-icteric medicinal	a medicinal that drains dampness and relieves icterus, indicated in the treatment of dampness-heat jaundice	湿邪を排除し、黄疸を緩和する薬剤。湿熱黄疸の治療に適用される。
利膽退黃藥		6.1.74	bile-draining anti-icteric medicinal	a medicinal that promotes the discharge of bile to relieve jaundice	胆汁の排出を促進し、黄疸を緩和する薬剤。
溫裏藥		6.1.75	interior-warming medicinal	a medicinal that warms the interior and expels internal cold	内部を温め、内寒を排除する薬剤。
理氣藥		6.1.76	qi-regulating medicinal	a medicinal that regulates qi movement to treat qi stagnation or adverse qi flow	気の動きを調節して、気滞または気逆を治療する薬剤。
消食藥	消食藥; 消導藥	6.1.77	digestant medicinal	a medicinal that aids digestion to eliminate accumulated undigested food	消化を助け、蓄積された未消化の食物をなくす薬剤。
消導藥	消食藥; 消導藥	6.1.77	digestant medicinal	a medicinal that aids digestion to eliminate accumulated undigested food	消化を助け、蓄積された未消化の食物をなくす薬剤。
驅蟲藥		6.1.78	worm-expelling medicinal	a medicinal that expels or kills parasitic worms	寄生虫を駆除するまたは殺す薬剤。
止血藥		6.1.79	hemostatic (medicinal)	a category of medicinals that arrest bleeding, either internal or external	内部または外部の出血を抑える薬剤のカテゴリー。
涼血止血藥		6.1.80	blood-cooling hemostatic medicinal	a medicinal that arrests bleeding by clearing the blood of heat, indicated in the treatment of blood-heat hemorrhage	血から熱を除去することで出血を抑える薬剤。血熱性出血の治療に適用される。
化瘀止血藥		6.1.81	stasis-resolving hemostatic medicinal	a medicinal that arrests bleeding and at the same time removes stagnant blood which may cause further bleeding	出血を抑え、同時に さらなる出血を起こしうる鬱滞した血を除去する薬剤。
收斂止血藥		6.1.82	astringent hemostatic medicinal	a medicinal that arrests bleeding by its astringent action	収斂作用により出血を抑える薬剤。
溫經止血藥		6.1.83	meridian-warming hemostatic medicinal	a medicinal that arrests bleeding by warming the meridians/channels	経絡を温めることで出血を抑える薬剤。
活血化瘀藥		6.1.84	blood-activating and stasis-resolving medicinal	a medicinal that promotes blood flow and resolves blood stasis	血流を促進し、鬱血を解消する 薬剤。
活血祛瘀藥		6.1.85	blood-activating and stasis-dispelling medicinal	a medicinal that promotes blood flow and dispels blood stasis	血流を促進し、鬱血をなくす薬剤。
活血藥		6.1.86	blood-activating medicinal	a category of medicinals used in the treatment of retarded or static blood flow	遅滞または鬱滞した血流の治療に用いられる薬剤のカテゴリー。
化瘀藥		6.1.87	stasis-resolving medicinal	a category of medicinals used in the treatment of blood stasis	鬱血の治療に用いられる薬剤のカテゴリー。
活血止痛藥		6.1.88	blood-activating analgesic medicinal	a medicinal that activates blood, moves qi and alleviates pain, indicated in the treatment of painful conditions caused by stagnation of qi and blood	血を活性化させ、気を動かし、疼痛を軽減する薬剤。気および血の鬱滞による有痛性の病態の治療に適用される。
活血行氣藥		6.1.89	blood-activating and qi-moving medicinal	a medicinal that activates blood and promotes the flow of qi, used primarily in treating stagnation of qi and blood	血を活性化させ、気の流れを促進する薬剤。主として気および血の鬱滞の治療に使用される。
活血調經藥		6.1.90	blood-activating menstruation-regulating medicinal	a medicinal that activates blood and regulates menstruation for treating menstrual disorders, dysmenorrhea, amenorrhea and postpartum blood stagnation	血を活性化させ、月経を調節する薬剤。月経疾患、月経困難症、無月経および産後の鬱血の治療に使用される。
活血療傷藥		6.1.91	blood-activating trauma-curing medicinal	a medicinal that activates blood, reduces swelling, arrests bleeding and promotes the healing of wounds and fractures	血を活性化し、腫脹を軽減し、出血を抑え、創傷や骨折の治癒を促進する薬剤。
破血消癥藥		6.1.92	blood-breaking mass-eliminating medicinal	a medicinal that, acting drastically, breaks up the static blood and eliminates masses	劇的に作用し、鬱血を破壊し、塊をなくす薬剤。

検索表示（原表示の複数語を分離）	IST原表示	Code	Term	Definition/Description	定義/記述
止咳平喘藥		6.1.93	cough-suppressing and panting-calming medicinal	a medicinal that relieves cough and dyspnea	咳嗽および呼吸困難を緩和する薬剤。
安神藥		6.1.94	tranquillizing medicinal	a medicinal that calms the mind and relieves mental tension	心を鎮め、精神的緊張を緩和する薬剤。
重鎭安神藥		6.1.95	settling tranquillizing medicinal	a medicinal, mostly a mineral, fossil bone or shell, that induces tranquilization with its settling action	大半が鉱物、化石骨、または貝殻であり、鎮静作用により安定化させる薬剤。
養心安神藥		6.1.96	heart-nourishing tranquillizing medicinal	a medicinal that nourishes yin-blood of the heart to calm the mind	心臓の陰血に栄養を与え、心を鎮める薬剤。
平肝熄風藥		6.1.97	liver-pacifying and wind-extinguishing medicinal	a medicinal that pacifies the liver, suppresses exuberant yang, extinguishes internal wind and controls spasms or tremors	肝臓を和らげ陽盛を抑制し、内風を消滅させ、痙縮や振戦を制御する薬剤。
開竅藥		6.1.98	orifice-opening medicinal	a medicinal, fragrant in flavor, with a resuscitating effect, used for emergency treatment of loss of consciousness in block pattern/syndrome	風味は芳香性で、蘇生効果がある薬剤。閉證における神昏の緊急治療に使用される。
補益藥		6.1.99	tonifying and replenishing medicinal	a category of medicinals that replenish the healthy qi and strengthen the body resistance, used for treating deficiency conditions	正気を補充し、体の抵抗性を高める薬剤のカテゴリー。欠乏症の治療に使用される。
補氣藥		6.1.100	qi-tonifying medicinal	a medicinal that tonifies the healthy qi, used in treating qi deficiency	正気を強める薬剤。気虚の治療に使用される。
補陽藥		6.1.101	yang-tonifying medicinal	a medicinal that tonifies yang qi, used for treating yang deficiency	陽気を強める薬剤。陽虚の治療に使用される。
補腎陽藥		6.1.102	kidney yang-tonifying medicinal	a medicinal that tonifies the kidney yang, used for treating kidney yang deficiency	腎陽を強める薬剤。腎陽虚の治療に使用される。
補血藥	補血藥;養血藥	6.1.103	blood-tonifying medicinal	a medicinal that tonifies the blood in treating blood deficiency, the same as the blood-nourishing medicinal	血虚治療において血を強める薬剤。養血剤と同義。
養血藥	補血藥;養血藥	6.1.103	blood-tonifying medicinal	a medicinal that tonifies the blood in treating blood deficiency, the same as the blood-nourishing medicinal	血虚治療において血を強める薬剤。補血剤と同義。
柔肝藥		6.1.104	liver-emolliating medicinal	a medicinal that replenishes yin-blood of the liver in treating deficiency of liver yin or blood	肝臓または血の欠乏症の治療において肝臓の陰血を補充する薬剤。
補陰藥	補陰藥;養陰藥;滋陰藥	6.1.105	yin-tonifying medicinal	a medicinal that tonifies and nourishes yin fluid, the same as the yin-nourishing or yin-replenishing medicinal	陰液を強めて栄養を与える薬剤。養陰薬または**滋**陰薬と同義。
養陰藥	補陰藥;養陰藥;滋陰藥	6.1.105	yin-tonifying medicinal	a medicinal that tonifies and nourishes yin fluid, the same as the yin-nourishing or yin-replenishing medicinal	陰液を強めて栄養を与える薬剤。補陰剤または滋陰薬と同義。
滋陰藥	補陰藥;養陰藥;滋陰藥	6.1.105	yin-tonifying medicinal	a medicinal that tonifies and nourishes yin fluid, the same as the yin-nourishing or yin-replenishing medicinal	陰液を強めて栄養を与える薬剤。養陰薬または補陰薬と同義。
收澁藥	收澁藥;固澁藥	6.1.106	astringent medicinal	a variety of medicinals that arrest discharges due to insecure health qi and declined visceral function such as excessive sweating, chronic diarrhea, enuresis, bleeding, leukorrhea and spermatorrhea of deficiency type	不安定な正気、および過剰な発汗、慢性下痢、遺尿症、出血、帯下および欠乏型の精液漏などの内臓機能低下による分泌を抑えるさまざまな薬剤。
固澁藥	收澁藥;固澁藥	6.1.106	astringent medicinal	a variety of medicinals that arrest discharges due to insecure health qi and declined visceral function such as excessive sweating, chronic diarrhea, enuresis, bleeding, leukorrhea and spermatorrhea of deficiency type	不安定な正気、および過剰な発汗、慢性下痢、遺尿症、出血、帯下および欠乏型の精液漏などの内臓機能低下による分泌を抑えるさまざまな薬剤。
固表止汗藥		6.1.107	exterior-securing anhidrotic medicinal	a medicinal that arrests excessive sweating by strengthening the superficies	表面を強化することで過剰な発汗を抑える薬剤。
斂汗固表藥		6.1.108	sweat-constraining exterior-securing medicinal	a medicinal that strengthens the superficies by arresting excessive sweating	過剰な発汗を抑えることで表面を強化する薬剤。
清熱收澁藥		6.1.109	heat-clearing astringent medicinal	a medicinal that clears heat and checks discharge, used in the treatment of bleeding or exudation due to pathogenic heat	熱を除去し、分泌を阻止する薬剤。病原性の熱による出血または滲出の治療に使用される。
斂肺澁腸藥		6.1.110	lung-intestine astringent medicinal	a sour and astringent medicinal that relieves cough and dyspnea, and arrests chronic diarrhea	咳嗽および呼吸困難を緩和し、慢性下痢を抑える酸および収斂薬。
湧吐藥	湧吐藥;催吐藥	6.1.111	emetic medicinal	a medicinal that induces vomiting	嘔吐を起こさせる薬剤。
催吐藥	湧吐藥;催吐藥	6.1.111	emetic medicinal	a medicinal that induces vomiting	嘔吐を起こさせる薬剤。
消散藥		6.1.112	resolving medicinal	a medicinal that promotes subsidence or diminution of sores and ulcers	瘡および潰瘍の軽減または減少を促進する薬剤。
方劑		**6.2.0**	**Formula**		
方劑		6.2.1	formula	prescription, recipe	方剤、レシピ。
經方		6.2.2	classical formula	formulary recorded in the Han dynasty and earlier, especially referring to the formulas recorded in Zhang Zhongjing's works	漢王朝以前に記録された処方書。特に張仲景の書籍に記録されている処方のこと。
漢方藥		6.2.3	Kampo formula	medicinal formula used in Kampo medicine	漢方医学で使用される薬剤の処方。
理法方藥		6.2.4	principles, methods, formulas and medicinals	the four basic steps of diagnosis and treatment: determining the cause, mechanism and location of the disease according to the medical theories and principles, then deciding the treatment principle and method, and finally selecting a formula as well as proper medicinals	診断および治療の4つの基本的なステップ。原因の決定、医学の理論と原理に基づく疾患のメカニズムと位置、そして治療の原理と方法の決定、最後に処方ならびに適切な薬剤の選択。

検索表示（原表示の複数語を分離）	IST原表示	Code	Term	Definition/Description	定義/記述
藥對		6.2.5	couplet medicinals	two medicinals used in pair to increase the therapeutic effect or reduce the toxic effect	治療効果を高めるまたは毒性を軽減するために対で使用される2つの薬剤。
君臣佐使		6.2.6	sovereign, minister, assistant and courier	the ingredients in a formula or prescription that have different roles	異なる役割を有する処方または方剤の成分。
君藥		6.2.7	sovereign medicinal	the ingredient that provides the principal curative action on the main pattern/syndrome or primary symptom	主な証または主要な症状に対して、主要な治癒作用を生じさせる成分。
臣藥		6.2.8	minister medicinal	the ingredient that helps strengthen the principal curative action	主な治癒作用を増強するのに有用な成分。
佐藥		6.2.9	assistant medicinal	the ingredient that treats the combined pattern/ syndrome, relieves secondary symptoms or tempers the action of the sovereign ingredient when the latter is too potent	合併した証を治療し、副次的な症状を緩和する、または強力すぎる場合に特効的な成分の作用を 軽減する成分。
使藥		6.2.10	courier medicinal	the ingredient that directs action to the affected meridian/channel or site	罹患した経絡 または部位への作用を支配する成分。
反佐		6.2.11	counteracting assistant	an assistant ingredient with a property opposite to and a therapeutic effect complementary to the sovereign ingredient	特効的な成分tとは反対の性質および特効的な成分に相補的な治療効果を有する補助的な成分。
大方		6.2.12	major formula	a formula or prescription which consists of many ingredients or large dosage of medicinals, used for treating a serious disease with exuberant pathogens or a disease of the lower energizer	多くの成分または大用量の薬剤から成る処方または方剤。過剰増殖性の病原体を有する重篤な疾患または下焦の疾患の治療に用いられる。
小方		6.2.13	minor formula	a formula or prescription which contains only a few ingredients of small dosage, used for treating a mild illness	2，3種類の成分または小用量の薬剤のみを含む処方または方剤。軽度の疾患の治療に用いられる。
緩方	緩方;<br>緩劑	6.2.14	slow-acting formula	a formula or prescription which is composed of ingredients that act slowly and is indicated in the long-term treatment of chronic cases	緩徐に作用する成分から構成される処方または方剤 。慢性疾患の長期治療に適用される。
緩劑	緩方;<br>緩劑	6.2.14	slow-acting formula	a formula or prescription which is composed of ingredients that act slowly and is indicated in the long-term treatment of chronic cases	緩徐に作用する成分から構成される処方または方剤 。慢性疾患の長期治療に適用される。
急方	急方;<br>峻劑	6.2.15	quick-acting formula	a formula or prescription which is composed of immediate effect in the treatment of emergency or critical cases	緊急または危篤症例の治療において、即時的な効果のある成分から構成される処方 。
峻劑	急方;<br>峻劑	6.2.15	quick-acting formula	a formula or prescription which is composed of immediate effect in the treatment of emergency or critical cases	緊急または危篤症例の治療において、即時的な効果のある成分から構成される処方 。
奇方		6.2.16	odd-numbered formula	a formula or prescription with ingredients odd in number	奇数の成分からなる処方または方剤 。
偶方		6.2.17	even-numbered formula	a formula or prescription with ingredients even in number	偶数の成分からなる処方または方剤 。
複方		6.2.18	compound formula	a formula or prescription that is formed by multiple ingredients or by two or more set formulas	複数の成分または2以上の処方セットから成る処方または方剤。
八陣		6.2.19	eight tactical arrays	a collective term for eight categories of formulas: supplementing array, harmonizing array, attacking array, dissipating array, cold array, heat array, securing array, and causal array	処方の8つのカテゴリーの総称。補陣、和陣、攻陣、散陣、寒陣、熱陣、固陣、および因陣。
主治		6.2.20	indication	major symptom or particular condition that indicate the advisability or necessity of a specific medical treatment or procedure, also called major indication	特定の薬剤投与または治療の勧告または必要性を示す主な症状または特定の病態。major indicationとも呼ばれる。
劑型		6.2.21	preparation form	form of a prepared medicine designed according to the medicinal property, therapeutic purpose and route of administration	薬剤の性質、治療目的、投与経路に基づいてデザインされた、調製された薬剤の形状。
湯劑		6.2.22	decoction (preparation)	a liquid medicine prepared by boiling the ingredients in water, and taken after the dregs are removed	水中で成分を煮沸することにより調製された液体の薬剤。カスを除去した後に使用する。
丸劑		6.2.23	pill preparation	a solid globular mass, coated or uncoated, made of finely powdered medicinals with a suitable excipient or binder	被覆または未被覆の、固形の球状の塊。適切な賦形剤または結合剤と共に微細粉末とした薬剤で作られている。
散劑		6.2.24	powder preparation	a medicated preparation in the form of discrete fine particles, for internal administration or topical application.	分散した微細な粒子の形状をしている製剤。内用または局所用。
蜜丸		6.2.25	honeyed pill	a globular medicated mass, in which processed honey is used as a binder	球状の製剤。処理した蜂蜜が結合剤として使われている。
糊丸		6.2.26	pasted pill	a small globular medicated mass, in which rice-paste or flour-paste is used as a binder	小さい球状の製剤。米のペーストまたは小麦粉のペーストが結合剤として使われている。
蠟丸		6.2.27	waxed pill	a globular medicated mass, in which beeswax is used as a binder	球状の製剤。蜜蝋が結合剤として使われている。
膏劑		6.2.28	paste preparation	a general term for soft extract, ointment and adhesive plaster	軟エキス剤、軟膏 および絆創膏の一般的な用語。
浸膏		6.2.29	extract	a concentrated medicated preparation obtained by extracting the active constituents	有効成分を抽出して得られた濃縮製剤。
流膏		6.2.30	fluid paste	a semiliquid medicated preparation containing the active constituents	有効成分を含む半液体製剤。
軟膏		6.2.31	ointment	an unguent (ointment) for topical application to the skin	皮膚に局所適用するための軟膏。
膏藥		6.2.32	plaster	a medicated dressing that consists of a film (as of cloth or paper) spread with a medicated substance	薬物を塗布した フィルム（布または紙）から構成される包帯。
藥酒	藥酒;<br>酒劑	6.2.33	medicated wine	a preparation made by steeping medicinals in wine or liquor	薬剤を発酵酒または蒸留酒に浸漬して作成した製剤。
酒劑	藥酒;<br>酒劑	6.2.33	medicated wine	a preparation made by steeping medicinals in wine or liquor	薬剤を発酵酒または蒸留酒に浸漬して作成した製剤。
丹劑		6.2.34	pellet	a medicated preparation in the form of small particles, usually made from minerals by sublimation for topical application, but some also for internal administration	小さい粒子の形状をした製剤。通常、昇華による局所適用のため鉱物から成るが、内用のものもある。
茶劑		6.2.35	medicated tea	a medicated preparation made into coarse powder or small cakes, taken as tea after being infused with boiling water or boiled in water	粗い粒子または小型のケーキの形状をした製剤。熱湯と共に煎じるかまたは煮沸し、お茶として使用する。
條劑		6.2.36	medicinal strip	a medicated preparation for external use, made by a twisted gauze coated with or enveloping medicinal powder to be introduced into a wound or fistula	外用の製剤。創傷または瘻孔に適用する薬用の粉末を被覆したまたは包んだ、ねじったガーゼで作られている。

検索表示（原表示の複数語を分離）	IST原表示	Code	Term	Definition/Description	定義/記述
沖劑	沖劑; 顆粒劑	6.2.37	soluble granules	granules made of medicinal extract, usually with sugar, to be dissolved in boiling water before being taken	通常砂糖と共に薬用の抽出物で作られた顆粒剤。使用前に熱湯に溶かす。
顆粒劑	沖劑; 顆粒劑	6.2.37	soluble granules	granules made of medicinal extract, usually with sugar, to be dissolved in boiling water before being taken	通常砂糖と共に薬用の抽出物で作られた顆粒剤。使用前に熱湯に溶かす。
片劑		6.2.38	tablet (preparation)	small flattened pill of compressed powdered medicine or extract of medicine with starch as a formative agent	散剤または薬剤の抽出物を、澱粉を形成剤として圧縮した、小型の平らな丸薬。
飲片		6.2.39	decocting pieces	medicinal substances in small pieces or slices for decocting, mostly after appropriate processing, also called medicinal slices	煎じるための薬用の物質の小片または切片。ほとんど、適切な処理がなされている。medicinal slices とも呼ばれる。
鍼劑		6.2.40	injection	a preparation of medicine intended for injection	注射用の薬剤。
坐藥	坐藥; 栓劑	6.2.41	suppository	a solid but readily meltable cone or cylinder of medicated material for insertion into rectum or agina	固形であるが溶けやすい、円錐状または円筒状の薬物。直腸または膣に挿入する。
栓劑	坐藥; 栓劑	6.2.41	suppository	a solid but readily meltable cone or cylinder of medicated material for insertion into rectum or agina	固形であるが溶けやすい、円錐状または円筒状の薬物。直腸または膣に挿入する。
藥線		6.2.42	medicated thread	a twisted paper like a thread, coated with or enveloping medicated powder	糸のようなねじった紙。散剤で被覆しているまたは散剤を包んでいる。
煎藥法		6.2.43	decoction method	the process of making decoction: to mix the ingredients with an adequate amount of water, boil them for a certain period of time and remove the dregs from the liquid before taking	煎じ薬を作るプロセス。成分を適切な量の水と混合し、適切な時間煮沸し、使用前に液体からカスを除去する。
水煎		6.2.44	decoct with water	boil the medicinals in water to make decoction	水から薬剤を煮沸し、煎じ薬を作ること。
先煎		6.2.45	decoct first	boil certain medicinals before other medicinals are added while making a decoction	煎じ薬を作るとき、他の薬剤を添加する前に特定の薬剤を煮沸すること。
後下		6.2.46	decoct later	add certain medicinals for boiling when the decoction is nearly done	ほぼ煎じ終わったときに、特定の薬剤を煮沸のために加えること。
包煎		6.2.47	wrap-decoct	boil the medicinal wrapped with a piece of cloth or gauze when making the decoction	煎じ薬を作るときに、薬剤を布片またはガーゼで包んで煮沸すること。
另煎	另煎; 單煎	6.2.48	decoct separately	boil the medicinal separately in order to avoid absorption of the extract by the dregs of other medicinals	他の薬剤のカスに抽出物が吸収されないように、別々に薬剤を煮沸すること
單煎	另煎; 單煎	6.2.48	decoct separately	boil the medicinal separately in order to avoid absorption of the extract by the dregs of other medicinals	他の薬剤のカスに抽出物が吸収されないように、別々に薬剤を煮沸すること
武火		6.2.49	strong fire	strong fire used for making decoctions which only allows a short period of boiling, such as pungent diaphoretics	刺激的な発汗剤など短時間の煮沸しかできない煎じ薬を作るときに使われる強火。
文火		6.2.50	slow fire	slow fire used for making decoctions which need a longer period of boiling, such as tonics	強壮剤 など長時間の煮沸が必要な煎じ薬を作るときに使われる弱火。
溶化		6.2.51	dissolve	dissolve a medicinal in water or a decoction before taking	使用前に薬剤または煎じ薬を水に溶かすこと。
沖服		6.2.52	take drenched	take medicine after pouring hot water or hot decoction of other medicinals over it, with stirring	使用前に、熱い湯または熱い他の薬剤の煎じ薬を、その薬剤の上に注いでから混ぜて服用すること。
飯前服		6.2.53	take before meal	to be taken about one hour before meal	食事の約1時間前に服用すること。
飯後服		6.2.54	take after meal	to be taken about one hour after meal	食事の約1時間後に服用すること。
食遠服		6.2.55	take between meals	to be taken at a longer interval from meal	食事の後、長時間おいてから服用すること。
平旦服		6.2.56	take before breakfast	to be taken in the morning on an empty stomach	朝、胃が空の時に服用すること。
臨睡服		6.2.57	take before sleeping	to be taken before bedtime	就寝前に服用すること。
代茶服		6.2.58	take as tea	to be taken in frequent small amounts as drinking tea	高頻度で少量ずつ、お茶と共に服用すること。
頻服		6.2.59	take in frequent small amounts	(decoction) to be taken in small portions at frequent intervals	(煎剤) 高頻度で少量服用すること。
頓服		6.2.60	take in one single dose	(decoction) to be taken all in one single dose	(煎剤) 全量を1回に服用すること。
冷服		6.2.61	take cold	(decoction) to be taken cold	(煎剤) 冷たいまま服用すること。
發表劑		6.2.62	exterior-effusing formula	any formula that is composed of exterior-releasing medicinals, has diaphoretic, muscle-releasing and eruption-promoting effects, and is used to treat exterior patterns/syndromes	解表剤で構成される全ての処方。発汗性の、筋弛緩および発疹促進効果を有し、表証の治療に用いられる。
湧吐劑	湧吐劑; 吐劑	6.2.63	emetic formula	any formula that induces vomiting, used for treating phlegm syncope, food accumulation, and ingestion of poisons	嘔吐を誘発する全ての処方。痰厥、食積、毒物の摂取の治療に用いられる。
吐劑	湧吐劑; 吐劑	6.2.63	emetic formula	any formula that induces vomiting, used for treating phlegm syncope, food accumulation, and ingestion of poisons	嘔吐を誘発する全ての処方。痰厥、食積、毒物の摂取の治療に用いられる。
攻裏劑		6.2.64	interior-attacking formula	any formula mainly composed of purgatives with bowel-moving, heat-purging, accumulation-attacking and water-discharging effects, used for treating interior excess patterns/syndromes	主として腸を動かし、熱を放出させ、蓄積物を破壊し、水を排出する効果を有する下剤 で構成される全ての処方。裏実証の治療に用いられる。
表裏雙解劑		6.2.65	exterior-interior-releasing formula	any formula that eliminates pathogens from both the exterior and the interior parts of the body, indicated in the treatment of dual disease of the exterior and interior	体の外部および内部の病原体を除去する全ての処方。表証および裏証をともにもつ疾患の治療に適用される。
和解劑		6.2.66	harmonizing and releasing formula	any formula that adjusts functions of the body indicated in the treatment of lesser yang disease, liver-spleen disharmony, and cold-heat complex patterns/ syndromes	体の機能の調整を行う全ての処方。少陽病、肝脾不和および寒熱錯雑証の治療に適用される。
理氣劑		6.2.67	qi-regulating formula	any formula that regulates and normalizes the flow of qi	気の流れを調節し正常化させる全ての処方。
理血劑		6.2.68	blood-regulating formula	any formula that corrects blood pathologies, including blood-tonifying formula, blood-activating and stasis-resolving formula, and haemostatic formula	血の異常を正常化させる全ての処方。養血処方、活血および鬱血解消の処方、止血処方を含む。
祛風劑	祛風劑; 治風劑	6.2.69	wind-dispelling formula	any formula that dispels or extinguishes pathogenic wind, either external or internal	外部または内部の風邪を消失させる全ての処方。
治風劑	祛風劑; 治風劑	6.2.69	wind-dispelling formula	any formula that dispels or extinguishes pathogenic wind, either external or internal	外部または内部の風邪を消失させる全ての処方。
祛寒劑	祛寒劑; 溫裏劑	6.2.70	cold-dispelling formula	any formula that warms the interior, used for the treatment of interior cold	内部を温める全ての処方。裏寒の治療に使われる。

検索表示（原表示の複数語を分離）	IST原表示	Code	Term	Definition/Description	定義/記述
溫裏劑	祛寒劑;溫裏劑	6.2.70	cold-dispelling formula	any formula that warms the interior, used for the treatment of interior cold	内部を温める全ての処方。裏寒の治療に使われる。
溫補劑		6.2.71	warm-tonifying formula	any formula with warm-tonifying effect used for treating cold-deficiency conditions	温補効果を有する全ての処方。寒虚証の治療に用いられる。
瀉下劑	瀉下劑;攻下劑	6.2.72	purgative formula	any formula composed of purgatives used for relieving constipation, purging heat, removing accumulation and expelling retained water in the treatment of interior excess patterns/syndromes	下剤で構成される全ての処方。裏実証の治療において、便秘の緩和、熱の放出、蓄積物の除去、および滞留水の放出に用いられる。
攻下劑	瀉下劑;攻下劑	6.2.72	purgative formula	any formula composed of purgatives used for relieving constipation, purging heat, removing accumulation and expelling retained water in the treatment of interior excess patterns/syndromes	下剤で構成される全ての処方。裏実証の治療において、便秘の緩和、熱の放出、蓄積物の除去、および滞留水の放出に用いられる。
寒下劑		6.2.73	cold purgative formula	a purgative formula cold in nature, indicated in the treatment of interior accumulation and stagnation of heat	性質は寒の、下剤の処方 。熱の内部蓄積および鬱滞の治療に適用される。
溫下劑		6.2.74	warm purgative formula	a formula containing yang-warming and cold-dissipating medicinals in combination with purgatives, used for treating interior excess cold patterns/ syndromes	下剤と併用して、陽を温め、寒を消散する薬剤を含む処方。裏実寒証の治療に用いられる。
潤下劑		6.2.75	lubricant laxative formula	formula containing moistening medicinals and purgatives, indicated for relieving constipation caused by insufficiency of intestinal fluid	給湿薬物および下剤を含む処方。腸液の不足による便秘の緩和に適用される。
解表劑		6.2.76	exterior-releasing formula	formula to dispel pathogenic factors from the superficies of the body for the treatment of exterior patterns/syndromes	表証の治療のため、体表から病原体を 除去するための処方。
淸暑劑	淸暑劑;祛暑劑	6.2.77	summerheat-clearing formula	formula that cleans summerheat for treating heat patterns/syndromes occurring in summer	夏に生じる熱証を治療するために暑熱を除去する処方。
祛暑劑	淸暑劑;祛暑劑	6.2.77	summerheat-clearing formula	formula that cleans summerheat for treating heat patterns/syndromes occurring in summer	夏に生じる熱証を治療するために暑熱を除去する処方。
利濕劑		6.2.78	dampness-draining formula	formula composed of dampness-draining diuretics, used to treat exuberant dampness	湿邪を除く利水剤で構成される処方。過増殖性の湿邪の治療に用いられる。
祛濕劑		6.2.79	dampness-dispelling formula	any formula that has the effect of resolving dampness, removing , water, relieving strangury and draining turbidity,for the treatment of water-dampness ailments	湿邪を消退させ、水を除去し、有痛排尿困難を緩和し、濁りを排出する効果を有する全ての処方。湿邪による疾患の治療に用いられる。
潤燥劑		6.2.80	dryness-moistening formula	any formula composed of moistening ingredients, used for treating fluid deficiency and dryness conditions	給湿成分から構成される全ての処方。液体の欠乏症および乾燥状態の治療に用いられる。
治燥劑		6.2.81	dryness-treating formula	any formula that relieves dryness patterns/syndromes, either exogenous or endogenous	外因性または内因性の燥証を緩和する全ての処方。
淸熱劑		6.2.82	heat-clearing formula	any formula that is mainly composed of heat-clearing ingredients and used for clearing heat, purging fire, cooling blood, or counteracting toxins in the treatment of interior heat patterns/syndromes	主として熱を除去する成分で構成される全ての処方。熱を除去し、火を排除し、血を冷却するために用いられる。または内熱証の治療において毒素を中和する。
瀉火劑		6.2.83	fire-draining/reducing formula	a drastic heat-clearing formula or a heat-clearing formula with concurrent purgative effect, used to treat fire patterns/syndromes	劇的に熱を除去する処方または熱を除去する処方。同時に下剤の効果も有する。火証 の治療に用いられる。
祛痰劑	祛痰劑;除痰劑	6.2.84	phlegm-dispelling formula	any formula used for expelling or dissipating phlegm	痰を放出し消散させるために用いる全ての処方。
除痰劑	祛痰劑;除痰劑	6.2.84	phlegm-dispelling formula	any formula used for expelling or dissipating phlegm	痰を放出し消散させるために用いる全ての処方。
消食劑	消食劑;消導劑	6.2.85	digestant formula	formula to promote digestion and disperse stagnant food	痰を放出し消散させるために用いる全ての処方。
消導劑	消食劑;消導劑	6.2.85	digestant formula	formula to promote digestion and disperse stagnant food	痰を放出し消散させるために用いる全ての処方。
開竅劑		6.2.86	orifice-opening formula	any formula chiefly composed of aromatic ingredients with a resuscitating effect, used for emergency treatment of loss of consciousness in block pattern/ syndrome	主として蘇生効果を有する芳香性の成分から構成される全ての処方。閉證における神昏の緊急治療に用いられる。
安神劑		6.2.87	tranquillizing formula	formula that calms or tranquilizes excitement, used for treating disquieted spirit	興奮を鎮めるまたは落ち着かせる処方。落ち着きのない精神の治療に用いられる。
固澁劑		6.2.88	securing and astringent formula	any formula chiefly composed of astringents that arrest exhaustive discharges of qi, blood, essence and fluids	主として、気、血、精、および津液の消耗性の放出を止める収斂剤で構成される全ての処方 。
補益劑		6.2.89	tonifying and replenishing formula	any formula that reinforces yang, replenishes qi, nourishes blood or supplements yin in deficiency conditions	欠乏症において陽を強め、気を補充し、血に栄養を与え、陰を補充する全ての処方。
驅蟲劑		6.2.90	worm-expelling formula	any formula that expels parasitic worms	寄生虫を駆逐する全ての処方。
殺蟲劑		6.2.91	worm-killing formula	any formula that kills parasitic worms	寄生虫を殺す全ての処方。
明目劑		6.2.92	vision-improving formula	a general term for formulas used in the treatment of eye diseases, also called eye-brightening formula	目の疾患の治療に使われる処方の一般的な用語。目を明るくする処方とも呼ばれる。
癰瘍劑		6.2.93	formula for treating abscess and ulcer	a general term for formulas used to treat sores, abscesses and ulcers in external medicine	外用薬で、瘡、膿瘍および潰瘍の治療に使われる処方の一般的な用語。
經産劑		6.2.94	formula for menstruation and childbirth	a general term for formulas used to treat menstrual disorders, leukorrheal ailments and obstetricdiseases	月経疾患、帯下の疾患、および産科の疾患の治療に使われる処方の一般的な用語。
救急劑		6.2.95	emergency formula	any formula used in emergency cases	緊急の場合に使われる全ての処方。
